# Methionine orchestrates the metabolism vulnerability in cisplatin resistant bladder cancer microenvironment

**DOI:** 10.1038/s41419-023-06050-1

**Published:** 2023-08-15

**Authors:** Chen Yang, Yuxi Ou, Quan Zhou, Yingchun Liang, Weijian Li, Yiling Chen, Wensun Chen, Siqi Wu, Yifan Chen, Xiyu Dai, Xinan Chen, Tian Chen, Shengming Jin, Yufei Liu, Limin Zhang, Shenghua Liu, Yun Hu, Lujia Zou, Shanhua Mao, Haowen Jiang

**Affiliations:** 1 Department of Urology, Huashan Hospital, Fudan University, Shanghai, China; 2 Intistute of Urology, Huashan hospital, Fudan University, Shanghai, China; 3 National Clinical Research Center for Aging and Medicine, Huashan Hospital, Fudan University, Shanghai, China; 4 Department of Urology, Fudan University Shanghai Cancer Center, Shanghai, China

**Keywords:** Self-renewal, Cancer microenvironment

## Abstract

Metabolism vulnerability of cisplatin resistance in BCa cells remains to be discovered, which we applied integrated multi-omics analysis to elucidate the metabolism related regulation mechanism in bladder cancer (BCa) microenvironment. Integrated multi-omics analysis of metabolomics and proteomics revealed that MAT2A regulated methionine metabolism contributes to cisplatin resistance in BCa cells. We further validated MAT2A and cancer stem cell markers were up-regulated and circARHGAP10 was down-regulated through the regulation of MAT2A protein stability in cisplatin resistant BCa cells. circARHGAP10 formed a complex with MAT2A and TRIM25 to accelerate the degradation of MAT2A through ubiquitin-proteasome pathway. Knockdown of MAT2A through overexpression of circARHGAP10 and restriction of methionine up-take was sufficient to overcome cisplatin resistance in vivo in immuno-deficiency model but not in immuno-competent model. Tumor-infiltrating CD8^+^ T cells characterized an exhausted phenotype in tumors with low methionine. High expression of SLC7A6 in BCa negatively correlated with expression of CD8. Synergistic inhibition of MAT2A and SLC7A6 could overcome cisplatin resistance in immuno-competent model in vivo. Cisplatin resistant BCa cells rely on methionine for survival and stem cell renewal. circARHGAP10/TRIM25/MAT2A regulation pathway plays an important role in cisplatin resistant BCa cells while circARHGAP10 and SLC7A6 should be evaluated as one of the therapeutic target of cisplatin resistant BCa.

## Introduction

As one of the leading urinary malignancies worldwide, bladder cancer (BCa) caused 17100 mortalities in 2022 [1]. Despite the development of surgical operation as well as chemotherapy and immunotherapy [2], the overall-survival of BCa remains unfavorable because of its high rate of relapse and metastasis [3]. Cisplatin is the first-line adjuvant chemotherapy treatment option for advanced state BCa [4]. Understanding the profound regulatory mechanism of BCa malignant phenotype and chemotherapy resistance is of urgent need to develop promising treatment strategies for BCa.

Metabolic reprogramming was considered as the hallmark of malignant transformed cells to sustain viability under external stress such as chemotherapy [5]. Glycolysis and Glutamine metabolism pathway are both well studied in cisplatin resistance induced metabolic reprogramming [6]. As one of the essential amino acid, methionine is crucial to keep protein metabolic homeostasis which sustains cell growth [7]. Several cancers have been reported to rely on increasing methionine consumption. Coupled with folate cycle, methionine metabolism provided one-carbon unit through methyl donor S-adenosylmethionine (SAM) to support several essential celluar activities as purine biosynthesis, thymidine biosynthesis, redox balance and epigenetic regulation [8].

Methionine adenosyl transferases (MATs) are the key enzymes of methionine metabolism to synthesis S-adenosylmethionine [9]. Methionine adenosyl transferase IIa (MAT IIa, MAT2A) was up-regulated in various human malignancies while silencing MAT2A resulted in inhibition of cell phenotype [10]. MAT2A was universally regulated within cells through ubiquitin-proteasome pathway in response to extracellular stress and nutrition deficiency [11, 12]. However, the intrinsic relationship between MAT2A regulated methionine metabolism and cisplatin resistant BCa remain largely unknown.

Since methionine is crucial to the malignant progression as an essential amino acid, several clinical studies were permitted to study whether restriction of methionine could improve outcome of patients [13, 14]. Methionine restriction was tolerated although limited clinical improvement was observed in multiple clinical trials, while a combination of methionine restriction and chemotherapy received promising results [15]. Previous pre-clinical researches as well as clinical trials of methionine restriction only focus on cancer cells while ignoring the anticipation of immune cells. The function and infiltration of CD8 + T cells into core section of cancer indicate a better prognosis in certain cancer types mainly because CD8 + T cells are the end-point executor of immunological process [16]. Recent works indicated methionine was critical in maintaining CD8 + T cell function [17]. The profound relationship between methionine metabolism and CD8 + T cells in cisplatin resistant BCa microenvironment still remains further discovery.

In this work, integrated analysis of metabolomics and proteomics revealed that cisplatin resistant BCa cells rely on MAT2A regulated methionine metabolism. A methionine metabolism related circRNA circARHGAP10 formed a three component complex with MAT2A and TRIM25 to decrease protein stability of MAT2A through ubiquitin-proteasome pathway. Inhibition of methionine metabolism was sufficient to overcome cisplatin resistance in vivo in immuno-deficiency model. Synergistic inhibition of MAT2A and SLC7A6 could overcome cisplatin resistance in immuno-competent model in vivo. Our work provides a promising therapeutic option based on circARHGAP10/TRIM25/MAT2A and SLC7A6 regulation pathway in cisplatin resistant BCa microenvironment.

## Results

### Characteristics of cisplatin resistant BCa cells

We first cultured human BCa T24 and UMUC-3 cells in complete DMEM medium with increasing cisplatin concentration from 1 μM to 25 μM to induce cisplatin resistant BCa cell lines as previously described [18]. Compared with the parental T24 (IC50 = 1.49 μM) and UMUC-3 (IC50 = 1.07 μM) cells, cisplatin resistant BCa cell T24-CR (IC50 = 21.86 μM) and UMUC-3-CR (IC50 = 27.49 μM) showed significantly increased resistance to cisplatin (Fig. S1A). T24-CR and UMUC-3-CR cells show a similar growth curve compared to T24 and UMUC-3 cells while only cisplatin resistant T24 and UMUC-3 cells preserve viability in 10 μM treatment concentration (Fig. S1B). EdU assays have further proved that proliferation rate of cisplatin resistant state of BCa cell remains the same (Fig. S1C), while apoptosis rate (Fig. S1D) and colony formation ability (Fig. S1E) only sustained in cisplatin resistant BCa cells while treated with cisplatin.

### Integrated multi-omics analysis identified the cisplatin resistant phenotype of BCa cells rely on MAT2A mediated methionine metabolism

**Fig. 1:**
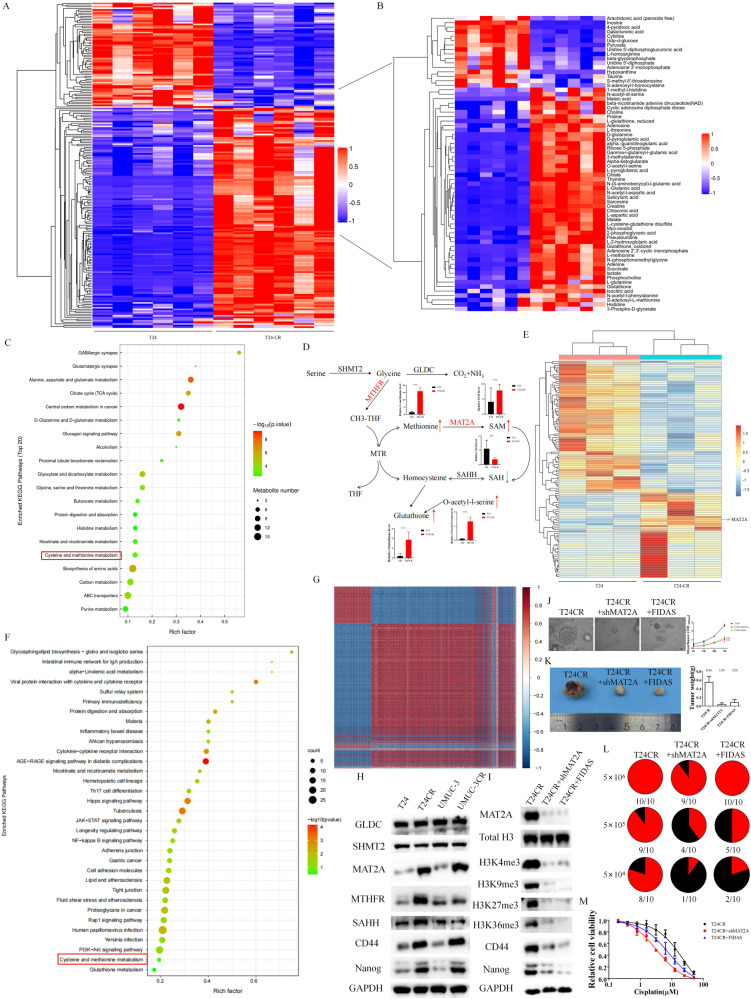
Multi-omics analysis of proteomics and metabolomics identified the cisplatin resistant BCa cells rely on MAT2A mediated methionine metabolism. **A**, **B** Metabolomics were performed in T24 cells versus T24-CR cells. Metabolites related with amino acid metabolism were further selected. **C** KEGG pathway enrichment of the metabolomics was applied to identified the enriched metabonomic pathway between T24 cells and T24-CR cells. **D** The schema of MAT2A regulated methionine metabolism pathway. Differentiated metabolites between T24 cells and T24-CR cells were marked with arrows. ***p < 0.001 versus T24. **E** Proteomics were performed in T24 cells versus T24-CR cells. **F** KEGG pathway enrichment of the proteomics was applied to identified the enriched pathway between T24 cells and T24-CR cells. **G** Integrated analysis of the correlation between the metabolomics and proteomics. **H** Western blot analysis of the enzymes and stem cell markers of the BCa cells. **I** Western blot analysis of the histone methylated markers and stem cell markers of the BCa cells with 5 μM FIDAS treatment or MAT2A silencing. **J** Spheroid assay was performed to analyze spheroid/organoid forming ability of the relatively treated T24-CR cells.Relative cell proliferation was measured through CCK-8 assay. ***p < 0.001 versus T24CR. **K** The tumor volume of 5 × 10^4^ T24-CR cells with relative treatment. The successful rate of tumor formation and weight were calculated (10 mice were enrolled in each treatment group). **L** The extreme limiting dilution experiment was performed to discover the in vivo tumorigenicity of T24-CR cells with relatively treatment. **M** Cell viability of T24-CR cells with different treatments in cisplatin containing medium.

We further carried out metabolomics between T24 cell and T24-CR cells to find differential metabolite after BCa acquire cisplatin resistance. Among 164 significantly dysregulated metabolites (Fig. 1A), we further identified 16 down-regulated metabolities and 50 up-regulated metabolities related with amino acid metabolism in T24-CR cells (Fig. 1B). GSEA analysis further enriched several metabolism pathway in T24-CR cells, while glutamine metabolism [19] and carbon metabolism [20, 21] were previously reported in cisplatin resistance (Fig. 1C). Proteomics was further performed between T24 cells and T24-CR cells while MAT2A was highly expressed in T24-CR cells (Fig. 1D, E). Although several biological processes were enriched in proteomics, we also monitored methionine metabolism was enriched in T24-CR cells (Fig. 1F, Fig. S2A). Glutamine metabolism, Carbon metabolism (Central carbon metabolism and TCA cycle), Glucagon metabolism, Glycine and serine metabolism as well as Histidine metabolism were also enriched in metabolomics data prior to methionine metabolism. However, among these metabolism pathways, only key enzymes in methionine metabolism as MAT2A was up-regulated in T24-CR cells according to proteomics data, which indicates methionine metabolism was crucial to cisplatin resistance in bladder cancer. In the methionine metabolism pathway, MAT2A transfers methionine to generate methyl-donor SAM in mammal cells. As a mean of supplying methionine, homocysteine could be transferred to methionine catalyzed via methionine synthase [22]. In T24-CR cells, methionine and SAM were enriched while SAH was down-regulated, which means the overall methionine cycle and methylation reactions were enhanced in T24-CR cells to provide essential methyl-doner for epigenetic regulation. As a by-products of methionine metabolism, glutathione was also up-regulated in T24-CR cells (Fig. 1D). Consistency analysis of metabolomics and proteomics indicated a relatively preferable correlation in the enriched pathways from metabolomics and proteomics, which indicates a perfect correlation of the two omics in cisplatin resistant bladder cancer cells (Fig. 1G). Through integrated multi-omics analysis of the protein and metabolism, we discovered that methionine metabolism participated in cisplatin resistance of BCa cell.

### Methionine metabolism contributes to cisplatin resistance of BCa through improving cell stemness

Chemotherapy agents as cisplatin could induce and educate cancer cell to gain cell stemness in order to survive the extracelluar stress [23]. Cancer stem cells share the characteristics of resistance to conventional chemotherapy and high rates of metastasis and relapse into more lethal cancer state [24]. Cancer stem cells rely on high exogenous methionine consumption and an active methionine cycle flux, not any other amino acids [25]. We further intend to discover the relationship between cisplatin resistance, methionine consumption and cancer stem cell characteristics. We first validated the protein expression of enzymes involved in methionine metabolism. Only MAT2A was highly expressed in both cisplatin resistant BCa cells, together with stem cell markers CD44 and Nanog (Fig. 1H). As the downstream of MAT2A induced methylation reactions, histone methylated markers were also highly expressed in cisplatin resistant cells (Fig. S2B). We further cultured T24-CR cell in mediums without different amino acids to rule out whether this phenomenon was strict to methionine. Medium without serine and glycine failed to decrease stem cell markers as well as histone methylated markers in cisplatin resistant BCa cells, while medium without glutamine to some extent inhibit these markers (Fig. S2C). We also monitored supplied methionine starvation medium with SAM or recovery cells after 48 h in complete medium sustain stem cell markers and histone methylated markers, not homocysteine (Fig. S2D). These results indicate SAM generated from MAT2A is crucial to cisplatin resistance induced cancer stem cell properties. We further knock down MAT2A or used FIDAS, a small molecular inhibitor of MAT2A, to down-regulate MAT2A as well as downstream pathway in T24-CR cells (Fig. 1I). Adherent cells lose cancer stem cell markers as well as high demands of methionine consumption, while cells maintained in organoids preserved these characteristics. The main phenotype of cancer stem cells is the high potential ability of tumorigenicity. While knocking down MAT2A or inhibiting MAT2A through FIDAS, the spheroid formation ability (Fig. 1J) as well as tumorgenicity ability (Fig. 1K, L) of T24-CR cells decline strikingly. T24-CR-shMAT2A (IC50 = 3.29 μM) and T24-CR-FIDAS (IC50 = 7.92 μM) were also more sensitive to cisplatin than T24-CR cells (Fig. 1M). These finding reinforced that cisplatin resistance of BCa enhanced cell stemness which relied on methionine metabolism.

### circARHGAP10 is a methionine metabolism related circRNA through the regulation of MAT2A in cisplatin resistant BCa cell

**Fig. 2:**
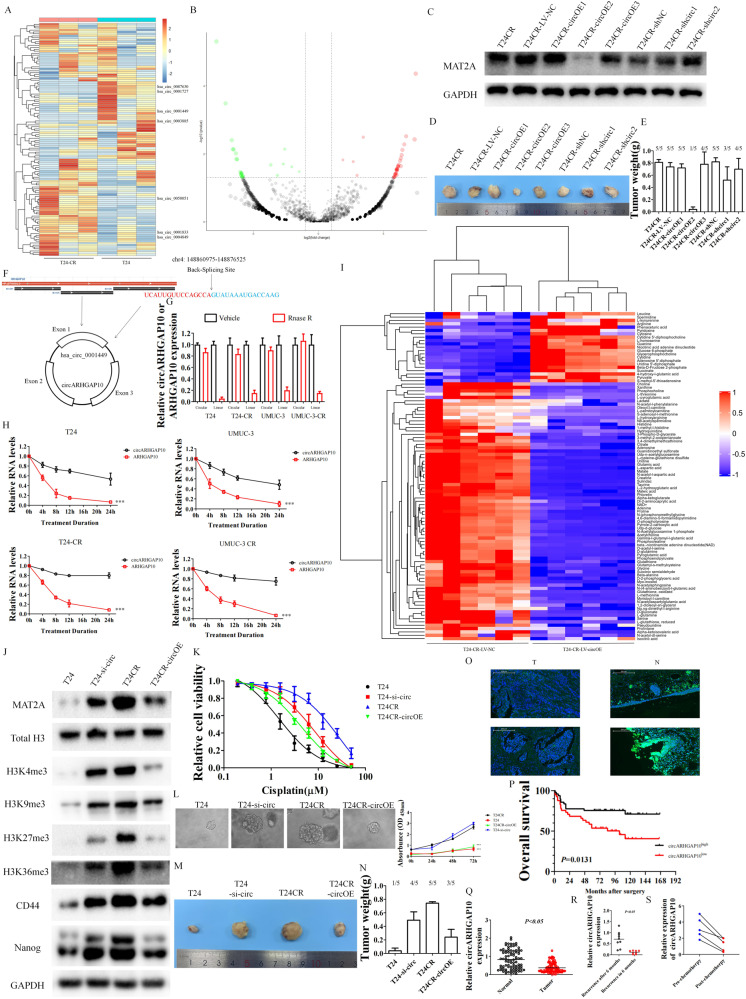
circARHGAP10 was identified as a methionine metabolism related circRNA in cisplatin resistant BCa cell. **A** Relative differential circRNA expression between T24 cells versus T24-CR cells. **B** Volcano plot was performed to reveal the differential expressed circRNAs. **C** Relative MAT2A expression of the T24-CR cells with the indicated circRNA knock down or up-regulated. **D** The tumor volume formed from 5 × 10^4^ T24-CR cells transfected with relative circRNA silencing or over-expression plasmid. **E** The successful rate of tumor formation and weight formed from 5 × 10^4^ T24-CR cells transfected with relative circRNA silencing or over-expression plasmid (5 mice were enrolled in each treatment group). **F** Genomic loci and structure of circARHGAP10. **G** Expression of circular/linear forms of transcripts in BCa cell with RNase R treatment. **H** Expression of circular/linear forms of transcripts in BCa cell after treatment by actinomycin D. ***p < 0.001 versus circARHGAP10 group. **I** Metabolomics were performed in T24-CR-LV-NC cells versus T24-CR-LV-circARHGAP10-OE cells. **J** Western blot analysis of MAT2A, the histone methylated markers and stem cell markers of T24, T24-si-circARHGAP10, T24-CR, T24-CR-LV-circARHGAP10 cells. **K** Cell viability of T24, T24-si-circARHGAP10, T24-CR, T24-CR-LV-circARHGAP10 cells in cisplatin containing medium. **L** Sphere formation assay was used to analyze the spheroid/organoid formation ability of the T24, T24-si-circARHGAP10, T24-CR, T24-CR-LV-circARHGAP10 cells. Relative cell proliferation was measured through CCK-8 assay. ***p < 0.001 versus T24CR. **M** The tumor volume formed from 5×10^4^ T24, T24-si-circARHGAP10, T24-CR, T24-CR-LV-circARHGAP10 cells. **N** The successful rate of tumor formation and weight formed from 5×10^4^ T24, T24-si-circ ARHGAP10, T24-CR, T24-CR-LV-circARHGAP10 cells (5 mice were enrolled in each treatment group). **O** Fluorescence in situ hybridization (FISH) of circARHGAP10 in our BCa tissue microarray (TMA). **P** Kaplan-Meier plot shows the correlation of circARHGAP10 with overall survival. **Q** circARHGAP10 expression levels in normal tissue and tumor tissue of BCa TMA. **R** circARHGAP10 expression in BCa tissue samples from recurrence after 6 months (*n* = 9) or recurrence in 6 months (*n* = 8). **S** Relative circARHGAP10 expression from paired BCa tissue samples from patients received cisplatin based chemotherapy (*n* = 5).

To overcome cisplatin resistance in BCa, targeting MAT2A directly is difficult since MAT2A universally expressed in various tissues. Circular RNAs (circRNAs) are highly expressed in various types of tissues and stable due to the covalent structure without polyA tails which possessed high prospects to be therapeutic target and cancer biomarkers. We performed high-throughput circRNA sequencing (Fig. 2A, B) between T24 and T24-CR cells as well as five pairs of benign and malignant bladder tissue (Fig. S3A, S3B) to discover certain circRNA which is crucial to cisplatin resistance and carcinogenesis in BCa. We identified four down-regulated circRNAs and three up-regulated circRNAs from the intersection of these two data set.

Among which, we further selected three down-regulated and two up-regulated circRNAs which have the same consistency in the normal bladder cells compared to BCa cell lines as well as in the parental BCa cell lines compared with cisplatin resistant cancer cell lines (Fig. S3C, S3D). We further exam the MAT2A expression in these T24-CR cells with either over-expressed or down-regulated certain circRNAs. Only T24-CR over-expressed with circOE2 expressed relatively low MAT2A (Fig. 2C), which indicated circOE2 potentially correlated with MAT2A mediated methionine metabolism. T24-CR-circOE2 cells also possess relatively low tumor formation ability (Fig. 2D, E) which indicates circOE2 cells interfere with cell stemness. The name of circOE2 from circbank is hsa_circ_0001449 (circARHGAP10), a 222nt circRNA originated from chromosome 4 ARHGAP10 gene (Fig. 2F). The circulation of circular RNA from lineal transcript was confirmed from RNase R decreased linear ARHGAP10 levels while did not change circARHGAP10 levels in BCa cells (Fig. 2G) and actinomycin D treatment confirmed that circARHGAP10 was more stable than linear ARHGAP10 in BCa cells (Fig. 2H). Methionine and SAM were down-regulated in T24-CR-circARHGAP10 OE cells (Fig. 2I) as well as several metabolites involved in cisplatin resistance (Fig. 1B, C) as glutamine, histidine and glutathione, which further reinforced that circARGHAP10 was methionine metabolism related circRNA.

We further discovered circARHGAP10 related phenotype of BCa cells. While knocking-down circARHGAP10 in parental BCa cells or over-expression of circARHGAP10 in cisplatin resistant BCa cells show less effect on cell proliferation (Fig. S4A), while the cell viability (Fig. S4A), cloning formation (Fig. S4B) and cell migration ability (Fig. S4C) significantly increased in parental BCa cells with circARHGAP10 silencing and decreased in cisplatin resistant BCa cells with circARHGAP10 over-expression in medium with 10 μM cisplatin. We also observed that MAT2A, stem cell markers as well as histone methylated markers were correlated with circARHGAP10 expression (Fig. 2J) which eventually determined the IC50 of BCa cell during cisplatin treatment (Fig. 2K). The spheroid formation ability (Fig. 2L) and tumorgenicity ability (Fig. 2M, N) of T24 and T24-CR cells also shows a correlation with MAT2A expression mediated via circARHGAP10. We further validated circARHGAP10 expression in 90 pairs of BCa TMA (Fig. 2O). circARHGAP10 was down-regulated in BCa tissue in compared to the paired normal bladder tissue (Fig. 2Q). Indeed, higher expression of circARHGAP10 was correlated with better prognosis (Fig. 2P) and lower tumor burden with tumor grades (Table S1). In addition, we found higher circARHGAP10 expression is related to lower recurrence rate (Fig. 2R). Paired bladder tumors from the same patients from before and after cisplatin based chemotherapy also indicates the down-regulation of circARHGAP10 (Fig. 2S). These results indicated circARHGAP10 is a MAT2A related circRNA which correlates with clinical stage and cisplatin-based chemotherapy in BCa.

### circARHGAP10 interacts with MAT2A and destabilizing MAT2A through ubiquitin/ proteasome dependent degradation

**Fig. 3:**
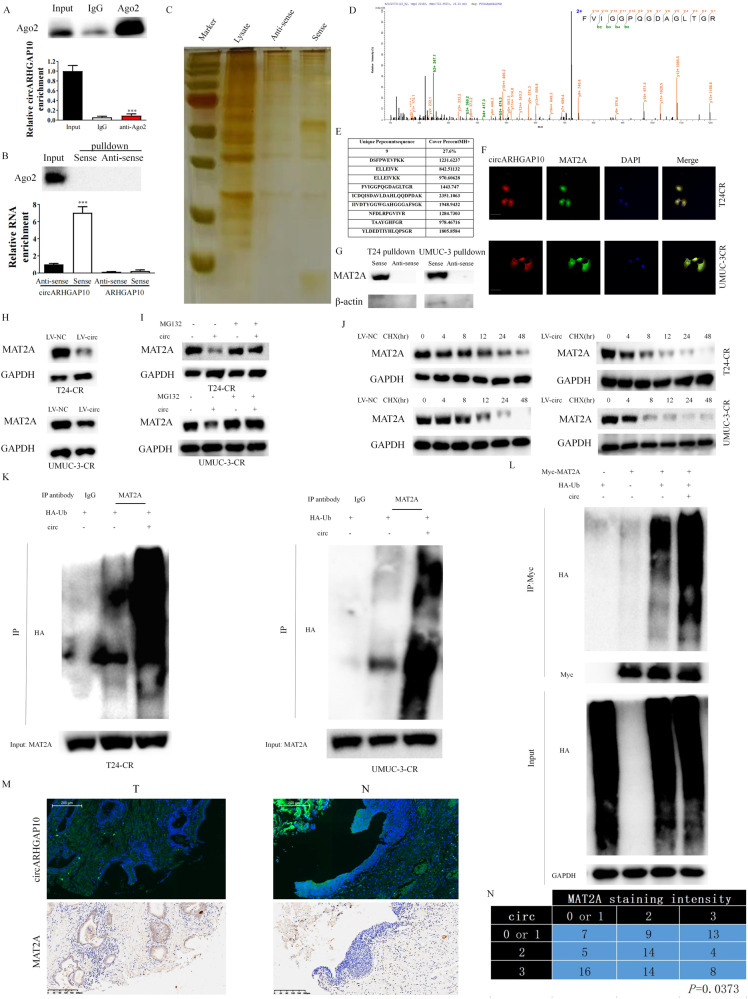
circARHGAP10 destabilizing MAT2A through the direct interaction with MAT2A. **A** RIP assays were performed with an anti-AGO2 antibody in T24-CR cells to analyze circARHGAP10 enrichment. ***p < 0.001 versus input group. (**B**) Analysis for lineal and circular ARHGAP10 enrichment. RNA pull down was performed with sense probe which targeting the back splice junction site of circARHGAP10. ***p < 0.001 versus anti-sense group. **C** Sense probe and antisense probe were incubated with cell lysates of T24-CR -LV-circARHGAP10 cells for silver staining. **D** The peptide segment diagram of MAT2A was identified by mass spectrometry from RNA pulldown. **E** Sequence of identical peptides of MAT2A identified by mass spectrometry. **F** Co-localization of circARHGAP10 with MAT2A in BCa cells. Scale bar = 10 μm. **G** The interaction between MAT2A and circARHGAP10. **H** Protein levels of MAT2A in BCa cells with circARHGAP10 overexpression. **I** Protein levels of MAT2A in circARHGAP10 overexpressed BCa cells with MG132 (20 μM) treatment for 10 h. **J** Relative protein levels of MAT2A and GAPDH in BCa cells treated with 200 μM cycloheximide (CHX) at different time points. **K** Co-IP experiment was performed to determine the ubiquitination modification level of MAT2A in BCa cells. **L** Co-IP experiment was performed to determine the ubiquitination modification level of MAT2A in 293 T cells. **M** Relative expression of MAT2A and circARHGAP10. **N** The contingency of the correlation of the staining intensity of MAT2A and circARHGAP10 analyzed with chi-squared test.

We further began to discover the regulation mechanism between MAT2A and circARHGAP10. RIP assay was applied to test whether circARHGAP10 functions as miRNA sponge in BCa cells. Ago2 was significantly enriched in anti-ago2 while circARHGAP10 was not enriched (Fig. 3A). Since ago2 mediated miRNA process is crucial for miRNA metabolism, no interaction observed between ago2 and circARHGAP10 indicates circARHGAP10 is not a ceRNA. We further used RNA pull-down assay to further discover the protein interacting ability of circARHGAP10. Firstly, we validated sense probe could efficiently and specifically enriched circARHGAP10 (Fig. 3B). The precipitates were separated through SDS-PAGE, silver staining and mass spectrometry analysis (Fig. 3C). Interestingly, we found MAT2A was included in the abundant interacting proteins (Fig. 3D, E), indicating a direct interaction between MAT2A and circARHGAP10. We further identified circARHGAP10 co-localized with MAT2A (Fig. 3F) and the direct interaction between circARHGAP10 with MAT2A (Fig. 3G).

We speculated circARHGAP10 could destabilize the protein stability of MAT2A through direct interaction. circARHGAP10 silencing and over-expression did not change the mRNA of MAT2A in BCa cells (Fig. S5A) which suggests that transcriptional regulation is not involved in MAT2A protein expression. Protein levels of MAT2A were reduced in circARHGAP10 overexpressed BCa cells (Fig. 3H), and this effect was abolished via MG132 treatment (Fig. 3I). Cycloheximide chase assays determined that stability of MAT2A decreased upon circARHGAP10 over-expression (Fig. 3J). These results indicated that circARHGAP10 may destabilize MAT2A via enhancing its ubiquitin/proteasome -dependent degradation through direct interaction. We further validated that circARHGAP10 significantly increased ubiquitination levels of MAT2A in BCa cells (Fig. 3K) and in 293 T cells (Fig. 3L), which indicates this regulation mechanism is universal in human cells. Through IHC staining of MAT2A expression in the tissue microarray (Fig. 3M), we found circARHGAP10 was inversely correlated with MAT2A (Fig. 3N) which further support that circARHGAP10 directly interacts with MAT2A and promote the degradation of MAT2A through ubiquitin dependent pathway.

### circARHGAP10 promotes the interaction between E3 ligase TRIM25 and MAT2A to enhance MAT2A ubiquitination

**Fig. 4:**
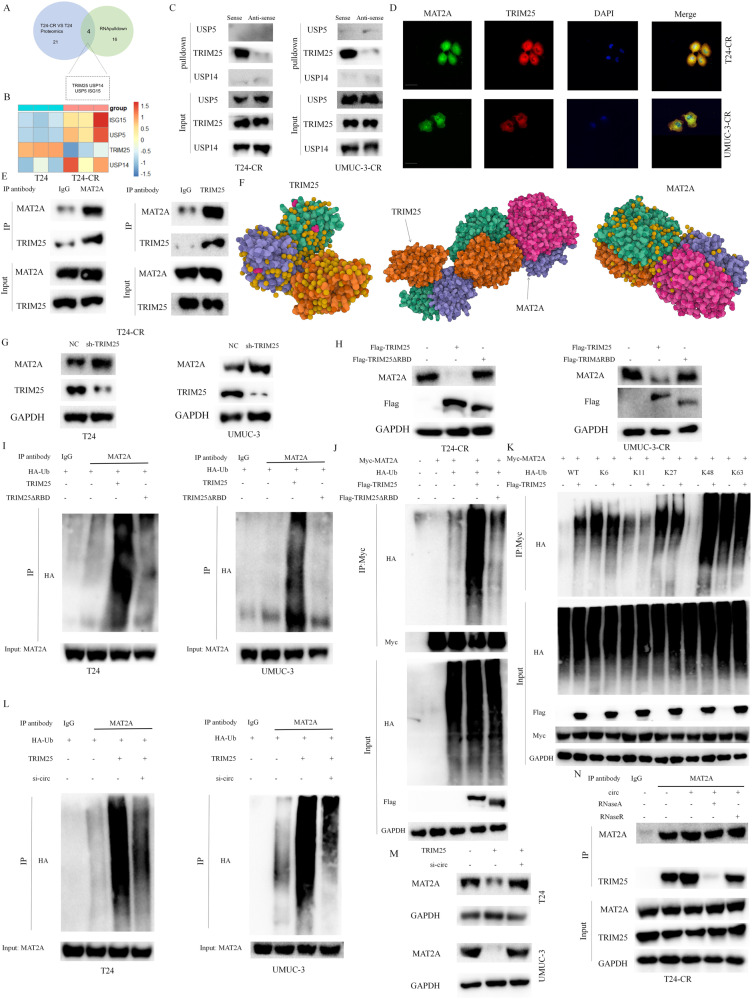
circARHGAP10 act as a scaffold for the interaction between TRIM25 and MAT2A to facilitate ubiquitination of MAT2A. **A** Intersection of the proteomics between T24 and T24-CR cells and the mass spectrometry analysis of circARHGAP10 RNA-pulldown. **B** Differential protein levels of ISG15, USP5, USP14 and TRIM25 of T24 and T24-CR cells identified with proteomics analysis. **C** Binding of circARHGAP10 with TRIM25 in BCa cells. **D** Co-localization of TRIM25 with MAT2A in BCa cells. Scale bar = 10 μm. **E** Co-IP validated TRIM25 binding with MAT2A in T24-CR cells. **F** Molecular docking of the protein structure of TRIM25 and MAT2A. **G** MAT2A protein expression in BCa cells with knockdown of TRIM25. **H** Protein levels of MAT2A in BCa cells with the over-expression of TRIM25 or TRIM25ΔRBD. **I** Co-IP experiment was performed to identify the ubiquitination modification level of MAT2A in BCa cells with the over-expression of TRIM25 or TRIM25ΔRBD. **J** Co-IP experiment was performed to identify the ubiquitination level of MAT2A in 293 T cells with the over-expression of TRIM25 or TRIM25ΔRBD. **K** Co-IP experiment was performed to identify the ubiquitination level of MAT2A in 293 T cells with the over-expression of HA-Ubiquitin or the indicated mutants. **L** Co-IP experiment was performed to identify the ubiquitination level of MAT2A in BCa cells with the over-expression of TRIM25 or the silencing of circARHGAP10. **M** The protein level of MAT2A in BCa cells with the over-expression of TRIM25 or the silencing of circARHGAP10. **N** Co-IP experiment was performed to validate the correlation of circARHGAP10 with binding of TRIM25 with MAT2A in T24-CR cells. RNase A (10 μg/ml) and RNase R (100 U/ml) were the indicated treatment concentration.

We performed the intersection between the proteomics of T24 and T24-CR cells with RNA-pulldown of circARHGAP10 with mass spectrometry analysis to further identify the ubiquitin related proteins for MAT2A ubiquitination. Two E3 ligases, TRIM25 and ISG15, and two ubiquitin specific protease family members, USP5 and USP14 were significantly alternated in both treatment group (Fig. 4A). We further identified that TRIM25 was up-regulated in T24 group. ISG15, USP5 and USP14 were up-regulated in T24-CR group in our proteomics analysis (Fig. 4B). Since the down-regulation of TRIM25 or up-regulation of USP5 and USP14 would decrease the ubiquitin level of relative proteins, we further choose these three ubiquitin-related proteins for further analysis. We then observed that TRIM25, not USP5 or USP14 interacted with circARHGAP10 in cisplatin resistant BCa cells (Fig. 4C) with the further RIP experiment validation (Fig. S5B).

E3 ligase took part in the ubiquitin dependent protein degradation pathway [26]. TRIM25, a tripartite motif family member of E3 ligase, add the poly-ubiquitin chains to the substrates for protein degradation in cells [27]. We further began to discover whether TRIM25 is the E3 ligase of MAT2A through the direct interaction between TRIM25 and MAT2A. co-immunoprecipitation analysis showed that TRIM25 directly interacted with MAT2A (Fig. 4D, Fig. S5C) and immunofluorescence (IF) confirms the co-localization between TRIM25 and MAT2A (Fig. 4E). Molecular docking of the three dimensional structure of TRIM25 and MAT2A also indicates a direct interaction between TRIM25 and MAT2A (Fig. 4F).

The RBP ability of TRIM25 is crucial to exert its E3 ligase activity. To further discover the regulation mechanism between TRIM25 and MAT2A, the FLAG-tagged RNA-binding domain (RBD) of TRIM25 mutant (without residues 470–508, named as TRIM25ΔRBD) [28] was constructed and the deletion of RBD broke the interaction between circARHGAP10 and TRIM25 completely (Fig. S5D). TRIM25 has no effect on the mRNA levels of MAT2A (Fig. S5E), while the MAT2A protein was significantly increased upon TRIM25 silencing (Fig. 4G) and down-regulated when TRIM25 was over-expressed (Fig. 4H). TRIM25ΔRBD has no effect on the protein levels of MAT2A (Fig. 4H), which indicates that the intact RNA-binding domain is crucial for the ubiquitination of MAT2A. The ubiquitination levels of MAT2A were increased with the up-regulation of TRIM25 while over-expression of the TRIM25ΔRBD has no effect on the ubiquitin levels of MAT2A in BCa cells (Fig. 4I) and 293 T cells (Fig. 4J).

We further enrolled K6, K11, K27, K48 and K63 mutation of the HA-Ubi plasmid [29] and found only K48 mutation could induce the up-regulation of ubiquitination levels of MAT2A (Fig. 4K). Since K48 mutation correlates the ubiquitin–proteasome degradation pathway, our results prove that TRIM25 acted as the E3 ligase to promote MAT2A degradation via the ubiquitin–proteasome dependent pathway.

Recent study has indicated TRIM25 enrolled circRNA as scaffold to promote the ubiquitination of target protein to exert the E3 ligase activity [28]. We further explored whether the ubiquitination activity of TRIM25 in promoting the MAT2A degradation depends on circARHGAP10. TRIM25 enhanced the ubiquitination levels of MAT2A while silencing circARHGAP10 abrogate this effect (Fig. 4L) to increase MAT2A in BCa cells (Fig. 4M). Co-IP was performed to verify whether circARHGAP10 worked as a scaffold to enhance the interaction between TRIM25 and MAT2A. The interaction between TRIM25 and MAT2A was enhanced with circARHGAP10 over-expression. We further found that RNA endonuclease RNase A abrogated the interactions between MAT2A and TRIM25 while RNA exonuclease RNase R did not have this effect which mainly due to resistance of circRNA to the degradation of RNA exonuclease (Fig. 4N, Fig. S5F). Taken together, we found that circARHGAP10 worked as the RNA scaffold to mediate the direct interaction between TRIM25 and MAT2A to promote TRIM25-depent ubiquitination of MAT2A.

### circARHGAP10 regulates MAT2A mediated methionine metabolism related stemness phenotype in cisplatin resistant BCa cell

MAT2A mediated methionine metabolism is crucial to cisplatin resistant phenotype of BCa cells and circARHGAP10 reduce the stability of MAT2A through providing a scaffold for the TRIM25 mediated degradation, we hypothesized that circARHGAP10 induced methionine metabolism related phenotype was through mediating the protein level of MAT2A. We found that knocking-down MAT2A significantly decreased the cell viability (Fig. S6A), colony formation (Fig. S6B) and migration ability (Fig. S6C) in BCa cells treated in cisplatin containing medium, while further silencing circARHGAP10 abrogated this effect. The protein levels of MAT2A, stem cell markers as well as histone methylated markers are down-regulated upon MAT2A silencing and up-regulated after further knocking down circARHGAP10 in BCa cells (Fig. S6D). Our results confirmed that MAT2A was the downstream regulator of circARHGAP10 mediated methionine metabolism related phenotype in cisplatin resistant BCa cells.

### Inhibition of methionine metabolism was sufficient to overcome cisplatin resistance in vivo in immuno-deficiency model

**Fig. 5:**
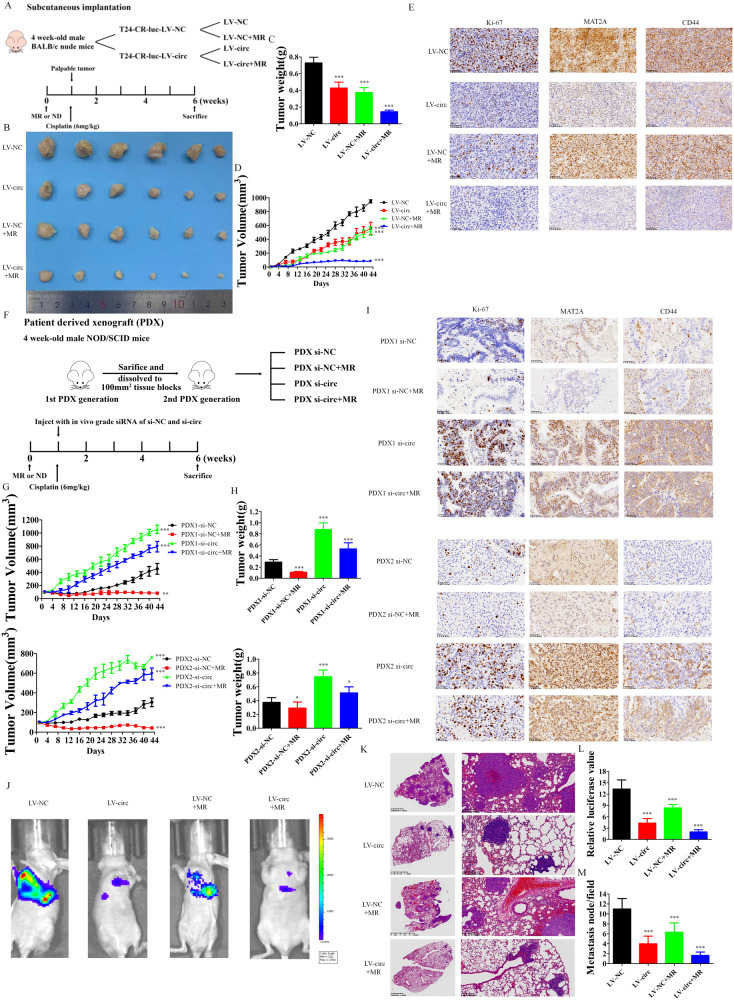
Methionine metabolism inhibition was sufficient to overcome cisplatin resistance in immuno-deficient in vivo model. **A** 1 × 10^7^ T24-CR-luc-LV-NC or T24-CR-luc-LV-circ cells were injected subcutaneously in 4-week-old nude male athymic BALB/c mice fed with control or MR diets under 6 mg/kg cisplatin treatment (six mice were enrolled in each treatment group). **B** Tumor volume was monitored after 6 weeks of treatment. **C** Tumor weight were measured separately in each group. Data are presented as the mean ± SD. ***p < 0.001 versus LV-NC group. **D** Tumor volumes were measured separately in each group. Data are presented as the mean ± SD. ***p < 0.001 versus LV-NC group. **E** IHC staining of the expression of Ki-67, MAT2A and CD44 in cisplatin resistant BCa cell xenograft model. **F** Patient-derived xenograft (PDX) models were established with two fresh fragments of tissue of BCa patients transplanted subcutaneously into 4-week-old NOD/SCID mice. The 2nd PDX generation were injected with 5 nmol in cholesterol-conjugated si-NC or si-circARHGAP10 fed with control or MR diets under 6 mg/kg cisplatin treatment (six mice were enrolled in each treatment group). **G** Tumor volumes were measured separately in each group. Data are presented as the mean ± SD. ***p < 0.001, **p < 0.01 versus PDX-si-NC group. **H** Tumor weights were measured separately in each group. Data are presented as the mean ± SD. ***p < 0.001, *p < 0.05 versus PDX-si-NC group. **I** IHC staining of the expression of Ki-67, MAT2A and CD44 in patient-derived xenograft model. **J** 1 × 10^5^ T24-CR-luc-LV-NC or T24-CR-luc-LV-circ cells were injected intravenously into the tails of 4-week-old nude male athymic BALB/c mice fed with control or MR diets under 6 mg/kg cisplatin treatment (6 mice were enrolled in each treatment group). **K** H&E staining of lung metastatic nodules with indicated treatment. **L** Bioluminescence was used to detect the metastasis ability of T24-CR-luc cells with indicated treatment. Data are presented as mean ± SD. ***p < 0.001 versus LV-NC group. **M** Numbers of lung metastatic nodules with indicated treatment. Data are presented as mean ± SD. ***p < 0.001 versus LV-NC group.

Since methionine could benefit cancer outcome through interfering with the downstream one-carbon metabolism, dietary restriction of methionine (MR) become an experimental therapeutic option which would create vulnerabilities involving redox balance and nucleotide metabolism to gain a synergism therapeutic effect with radiation and chemotherapy [30]. However, long-time methionine restriction in immunocompromised mice could inhibit the growth of tumor with leading the eventually death to animals [31]. Also, little clinical benefits were observed in clinical trials about MR [15, 32, 33]. We further began to discover whether inhibition of methionine metabolism through circARHGAP10 over-expression and restriction of methionine up-take was sufficient to overcome cisplatin resistance in vivo. To further validate whether circARHGAP10 mediated MAT2A expression or methionine restriction regulates cisplatin resistance of BCa in vivo, three immuno-deficiency mouse models were enrolled: 1. subcutaneous cisplatin-resistant T24 cell xenograft model in nude mice. 2. two patient-derived xenograft (PDX) models in NOD-SCID mice. 3. cisplatin-resistant T24 cell lung metastasis in nude mice. We did not observe liver injury and mice body weight lose in all treatment group, which indicates the drug concentration and combination in our preclinical studies are relatively safe (Fig. S7A–D). Over-expression of circARHGAP10 or MR markedly induced the cisplatin sensitivity of T24-CR xenograft (Fig. 5A, B), while these two treatments showed a synergetic effect in reducing T24-CR xenograft tumor weight (Fig. 5C) and volume (Fig. 5D). Immunohistochemistry analysis of MAT2A, stem cell marker CD44 and Ki-67 were also down-regulated after the inhibition of methionine cycle (Fig. 5E). Compared to cisplatin treatment-naive bladder cancer, cisplatin-treated cancer has relatively higher MAT2A expression, lower circARHGAP10 expression and higher histone methylation level(Fig. S7E, F).In two PDX models, knocking down circARHGAP10 with cholesterol-conjugated si-circRNA [34] (Fig. 5F) increased cisplatin resistance of the cisplatin treatment naive BCa tissue (Fig. 5G, H) and the protein expression of MAT2A, CD44 and Ki-67 (Fig. 5I). MR slightly increased the efficacy of cisplatin in PDX models. We also found over-expression of circARHGAP10 or MR hampered the metastasis ability of T24-CR cells through analysis with bioluminescence imaging results (Fig. 5J, L) and H&E staining (Fig. 5K, M) which indicates that interfere with methionine cycle both inhibiting the tumorigenesis and metastasis of BCa cells. In concluding, we validated that down-regulation of methionine metabolism through circARHGAP10 over-expression and restriction of methionine up-take was sufficient to overcome cisplatin resistance in vivo in immuno-deficiency model.

### Methionine metabolism modulated CD8 + T cell function in cisplatin resistant BCa microenvironment

Surprisingly, inhibition of methionine metabolism cycle in immuno-competent model could not achieve the similar in-vivo efficacy to cisplatin resistant BCa as in immuno-deficiency model (Fig. S7G, H). Since the clinical application of methionine restriction (MR) mainly associated with the immuno-competent patient, it is of great value to understand the regulation mechanism for the improvement of the MR based treatment. We first confirmed the above regulation mechanism was preserved in the mouse urothelial carcinoma MB49 cells. The conserved mouse circRNA from human hsa_circ_0001449 (circARHGAP10) is mmu_circ_0014984 (circArhgap10) with an overall 95.5% shared similarity sequences (Fig. S8A). RNase R decreased linear Arhgap10 levels without changing circArhgap10 levels in MB49 cells (Fig. S8B) and circArhgap10 was more stable than linear form in MB49 cells (Fig. S8C). Cisplatin resistant MB49 cells (IC50 = 25.41 μM) were more resistant to cisplatin treatment than MB49 cells (IC50 = 3.45 μM) (Fig. S8D). Treatment with FIDAS [25], a small molecular inhibitor of MAT2A, inhibit the expression of MAT2A mediated cancer stem cell marker and histone methylated marker in MB49-CR cells (Fig. S8E) and decreased the cell viability of MB49-CR cells with cisplatin treatment (Fig. S8F). Silencing TRIM25 in MB49 cells caused the increased MAT2A protein levels (Fig. S8G). Silencing circArhgap10 in MB49 cells or over-expression circArhgap10 in MB49-CR cells significantly increased or decreased the protein levels of MAT2A, H3K4me3 as well as CD44 (Fig. S8H), while cell viability of MB49-CR cells in cisplatin containing medium correlates with the protein levels of MAT2A (Fig. S8I). Silencing MAT2A through sh-MAT2A was abrogated with knocking down circArhgap10 (Fig. S8J), together with the expression of MAT2A regulated protein levels and resistance to cisplatin (Fig. S8K).

TRIM25 enrolled circArhgap10 to increase the ubiquitination levels of MAT2A (Fig. S8L).

**Fig. 6:**
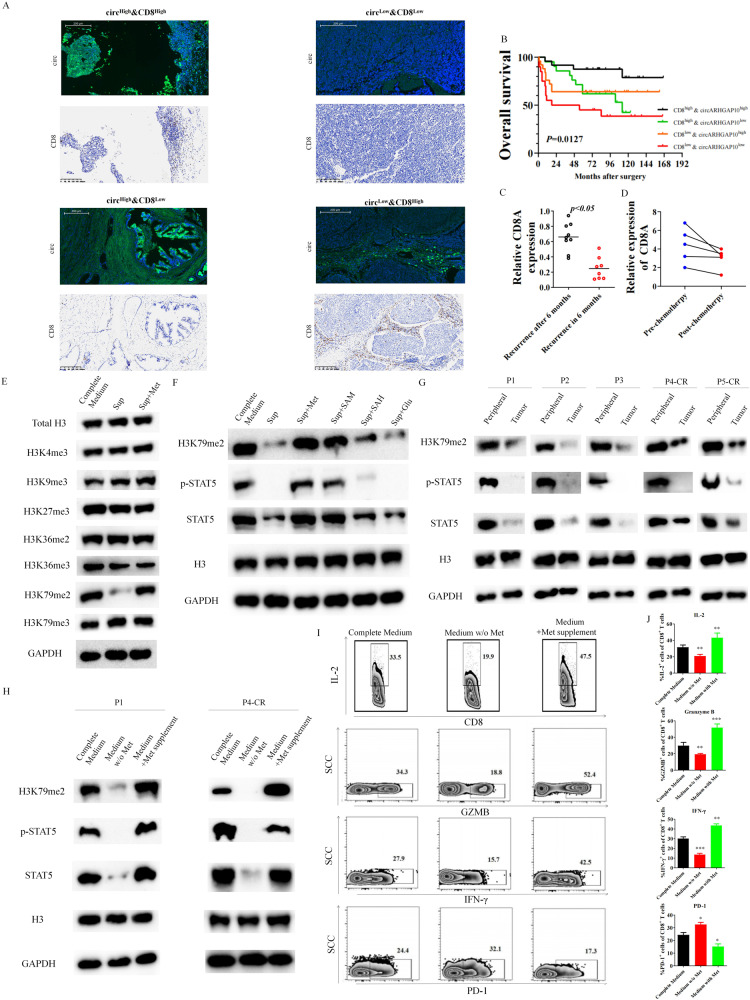
Methionine orchestrated CD8 + T cell function in cisplatin resistant BCa microenvironment. **A** Representative images of circARHGAP10/CD8 in the TMA. **B** Kaplan–Meier of overall survival of BCa patients with the analysis of CD8 + T cells and/or circARHGAP10 expression. **C** CD8A expression in BCa tissue samples from recurrence after 6 months (*n* = 9) or recurrence in 6 months (*n* = 8). **D** Relative CD8A expression from paired BCa tissue samples from patients received cisplatin based chemotherapy (*n* = 5). (E) Effect of supernatant of T24CR cells on histone methylation markers in CD8 + T cells. Sup is the conditional medium of cisplatin resistant BCa cells without any supplements. **F** Effect of methionine metabolism related metabolite supplement on H3K79me2 and STAT5 level in CD8 + T cells. **G** Expression of H3K79me2 as well as STAT5 in peripheral blood or tumor derived CD8 + T cells from patients. **H** Effect of methionine on expression of H3K79me2 as well as STAT5 in CD8 + T cells. **I** Effect of methionine on the expression of IL-2, effector molecules IFN-γ, granzyme B and immune checkpoint PD-1 in CD8 + T cells. **J** Data are presented as the mean ± SD. ***p < 0.001, **p < 0.01, *p < 0.05 versus Complete Medium.

We further realized immuno-related regulation is crucial to the methionine metabolism mediated cisplatin resistance in BCa cells. We analyzed our TMA cohort based on the expression of CD8 and circARHGAP10 levels (Fig. 6A). CD8+ high and circARHGAP10 high groups had the most preferable prognoses, while CD8+ low and circARHGAP 10 low groups indicated the worst survival rates (Fig. 6B). Since circARHGAP10 correlated with MAT2A mediated methionine metabolism, above results suggested that methionine metabolism correlates with the function and infiltration of CD8 + T cell. We also validated that CD8 expression correlated with recurrence of BCa (Fig. 6C) and cisplatin based chemotherapy (Fig. 6D).

CD8 + T cells functioned as the final executor of the anti-tumor response and tumor cells often cause the exhaustion of tumor-infiltrating immune cells [16, 35]. Specifically, previous research indicates malignant cells outcompete immune cells for nutrition as methionine in cancer microenvironment, thereby impairing the function of CD8 + T cell [17]. The restricted intra-cellular methionine level caused low SAM levels and the deficiency of dimethylation at H3K79me2 which eventually hampered STAT5 function to impair T cell related immunity response [36, 37]. We first identified CD45 + /CD3 + /CD8+ immune cells as the selected CD8 + T cells (Fig. S9A). In order to identify which amino acid contributes to the most methionine metabolism regulated CD8 + T cells dysfunction, patient derived CD8 + T cells in supernatant from cultured T24-CR cells was cultured with the supplement of certain amino acids. As previously reported, medium from cultured cisplatin resistant BCa cells resulted in marked T cell dysfunction, while supplemented with methionine significantly rescue the exhausted phenotype of CD8 + T cells (Fig. S9B). We found after cultured with the supernatant from cisplatin resistant BCa cells, only the decrease in H3K79me2 was observed among all the histone methylated markers (Fig. 6E). Only SAM, not SAH or glucose rescued the H3K79me2 and the STAT5 function of CD8 + T cells (Fig. 6F). We further found that CD8 + T cells (Human CD8 + T cells, IC50 = 10.38 μM, Mouse CD8 + T cells, IC50 = 7.08 μM) are more sensitive to methionine starvation than cisplatin resistant BCa cells (T24-CR, IC50 = 0.64 μM, UMUC3-CR, IC50 = 0.87 μM and MB49-CR, IC50 = 2.63 μM) (Fig. S9C), which further supported that the function of CD8 + T cells depends on the methionine levels in BCa microenvironment. We further isolated the T cells derived from peripheral blood and tumor, while blood derived CD8 + T cells preserved a relatively high H3K79me2 level and STAT5 function than CD8 + T cells from tumor tissues (Fig. 6G). CD8 + T cells cultured in culture medium without methionine could be characterized as low H3K79me2 level and STAT5 function (Fig. 6H) as well as deficiency of IL-2, GZMB and IFN-γ expression and high PD-1 expression (Fig. 6I, J), which could be rescued through methionine supplement. CD8 + T cells cultured in conditional supernatant from T24-CR cells and supplemented with methionine showed a similar result (Fig. S9D). Our results indicated that tumor cells consumed methionine in cisplatin resistant microenvironment to hamper CD8 + T cells through methionine metabolism mediated H3K79Me2/STAT5 regulated pathway.

### SLC7A6 mediated methionine metabolism related CD8 + T cell function in cisplatin resistant bladder cancer microenvironment

**Fig. 7:**
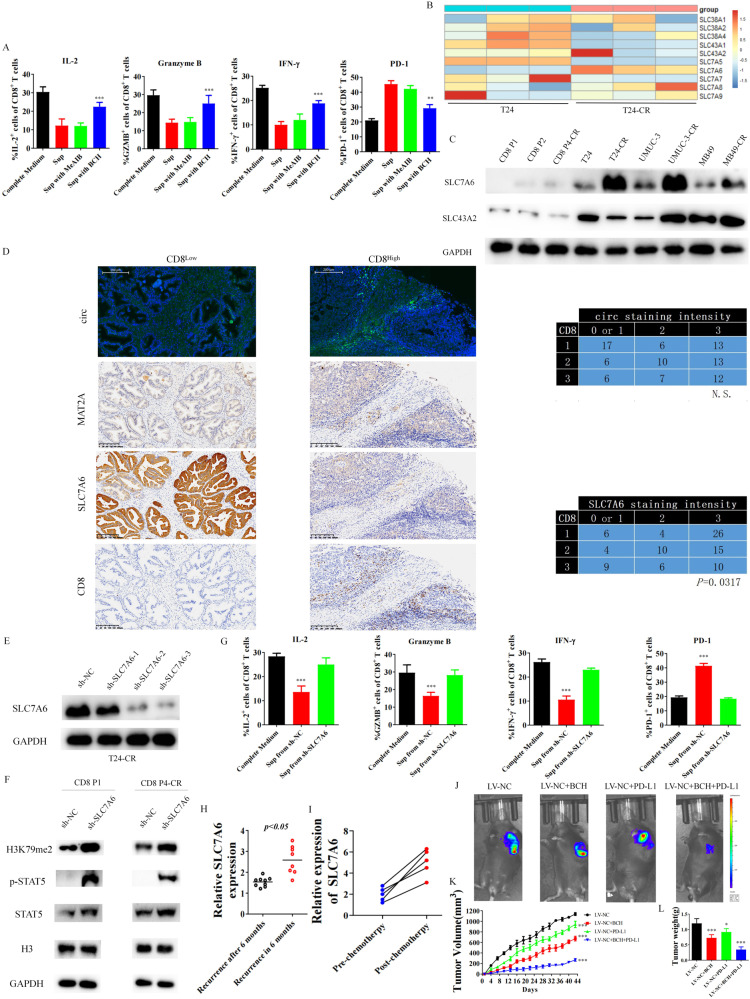
SLC7A6 mediated CD8 + T cell function in cisplatin resistant bladder cancer microenvironment through alteration of methionine absorption. **A** Effects of BCH or MeAIB on supernatant of T24CR cells affected function of CD8 + T cell. Data are presented as the mean ± SD. ***p < 0.001, **p < 0.01 versus Sup. **B** RT-qPCR showed transcripts of SLC transporter in T24 and T24-CR cells. **C** The protein expression of SLC7A6 and SLC43A2 from patient derived CD8 + T cells and BCa cells. **D** Relative expression of MAT2A, circARHGAP10, SLC7A6 and CD8A in TMA. The contingency correlating the staining intensity of relative marker was analyzed with chi-squared test. **E** SLC7A6 knockdown efficiency in T24-CR cells. **F** Effects of expression of H3K79me2/STAT5 in CD8 + T cell with culturing with supernatants from shSLC7A6-T24CR cells. **G** Effects of shSLC7A6-T24CR cells affected function of CD8 + T cell. Data are presented as the mean ± SD. ***p < 0.001 versus Complete Medium. **H** SLC7A6 expression in BCa tissue samples from recurrence after 6 months (*n* = 9) or recurrence in 6 months (*n* = 8). **I** Relative SLC7A6 expression from paired BCa tissue samples from patients received cisplatin based chemotherapy (*n* = 5). **J** Combination of BCH with anti-PD-L1 treatment on MB49-CR cell xenograft model (6 mice were enrolled in each treatment group). **K** Tumor volumes were presented as mean ± SD. ***p < 0.001 versus LV-NC group. **L** Tumor weight were presented as mean ± SD. ***p < 0.001, *p < 0.05 versus LV-NC group.

We did not observe significant improved function of CD8 + T cells after cultured with the supernatant from cisplatin resistant BCa cells with silencing MAT2A or circARHGAP10 over-expression (Fig. S9E), which indicated MAT2A was not in charge of the consumption of methionine in cisplatin resistant BCa cell from tumor microenvironment. Solute carrier (SLC) family, including system solute carrier L and solute carrier A type transporters, is responsible for the transportation of methionine into cells [38]. After cultured T24-CR cells with inhibitor of L type transporters-BCH or A type transporters-MeAIB, CD8 + T cells were cultured with conditional culture medium and analyzed for the function of CD8 + T cells. Only BCH rescued the impaired CD8 + T cell function (Fig. 7A). We also compared the mRNA expression of SLC family transporters between T24 and T24-CR cells while only the L type transporter SLC7A6 of SLC family was highly expressed in cisplatin resistant BCa cells (Fig. 7B). Furthermore, minimum protein expression of SLC7A6 was observed in CD8 + T cells while high SLC7A6 expression in BCa cells (Fig. 7C). We observed that more SLC7A6 expression in resistant BCa cells compared to naive cells, while the previously reported methionine metabolism related SLC43A2 did not expressed highly in cisplatin resistant BCa cells. The differential expression of SLC7A6 in CD8 + T cells, treatment naive BCa cells and cisplatin resistant BCa cells suggest that cisplatin resistance cancer cells may outcompete T cells for the consumption of methionine via SLC7A6 to overcome cisplatin resistance. We further began to figure out the relationship between MAT2A and SLC7A6 in cisplatin resistant BCa cell. Silencing MAT2A caused the slightly over-expression of SLC7A6 in BCa cells while silencing SLC7A6 had no effect on MAT2A (Fig. S10A). Only silencing SLC7A6 could rescue the decreased CD8 + T cells function from cultured with supernatant of BCa cells (Fig. S10B, C). Both silencing MAT2A and SLC7A6 could decreased the cell proliferation rate of BCa cells (Fig. S10D), while MAT2A silencing significantly contributes to the cisplatin sensitivity than SLC7A6 in cisplatin resistant cancer cells (Fig. S10E).

We also validated in our TMA cohort that only SLC7A6 negatively correlated with CD8, indicating that high expression of SLC7A6 in tumor microenvironment restricted infiltration of CD8 + T cells (Fig. 7D). We knock down SLC7A6 in T24-CR cells (Fig. 7E) and cultured CD8 + T cells with the conditional supernatant of T24-CR-shSLC7A6 cells. CD8 + T cell possessed relatively elevated H3K79me2 and pSTAT5 levels (Fig. 7F), with the restoration of the function after knock down SLC7A6 in T24-CR cells (Fig. 7G). Elevated SLC7A6 expression correlated with rapid recurrence of BCa (Fig. 7H) and resistance to cisplatin based chemotherapy (Fig. 7I). Since no small molecular inhibitor of SLC7A6 is available, we treated MB49-CR transplanted mice with BCH in combined with anti-PD-L1 (Fig. 7J). The combination treatment of BCH and PD-L1did not cause potential toxicity in animal model (Fig. S7I, J). Single treatment of BCH or anti-PD-L1 inhibited the growth of MB49-CR xenograft partially and combination treatment showed a synergistic effect (Fig. 7K, L), which further indicated SLC7A6 modulated immune response in cisplatin resistant BCa microenvironment.

### Synergistic inhibition of MAT2A mediated methionine metabolism and SLC7A6 overcame cisplatin resistance of BCa cells in immuno-competent model

**Fig. 8:**
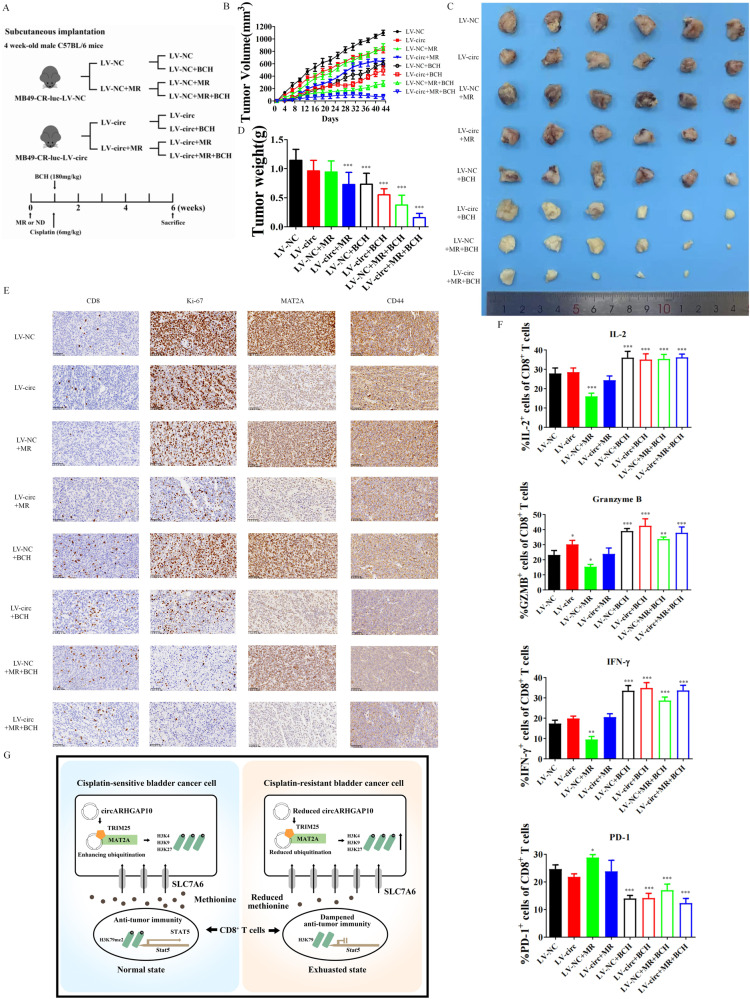
Combined inhibition of MAT2A and SLC7A6 overcame cisplatin resistance of BCa cells in immuno-competent model. **A** 1×107 MB49-CR-luc-LV-NC or MB49-CR-luc-LV-circ cells were injected subcutaneously into four-week-old C57BL/6 male mice treated with control or MR diets with or without 180 mg/kg BCH under 6 mg/kg cisplatin treatment (6 mice were enrolled in each treatment group). **B**, **C** Relative tumor volume was calculated after mice were sacrificed with 6 weeks of treatment. **D** Tumor weight were measured separately in each group. Data are presented as the mean ± SD. ***p < 0.001 versus LV-NC group. **E** IHC analysis of the expression of CD8, Ki-67, MAT2A and CD44 in cisplatin resistant BCa cell xenograft model. **F** Expression of IL-2, IFN-γ, granzyme B and PD-1 in CD8 + T cells from mice xenograft. Data are presented as the mean ± SD. ***p < 0.001, **p < 0.01, *p < 0.05 versus LV-NC. **G** Schematic diagram of the pivotal role of methionine metabolism in cisplatin resistant BCa microenvironments.

We summarized that MAT2A mediated cisplatin resistance in BCa cells, while SLC7A6 was responsible for the methionine consumption of BCa cells to restrict the function of CD8 + T cells. We employed circRNA over-expression, MR and BCH to further discover whether these combined treatments could decrease the tumor burden of cisplatin resistant BCa in immuno-competent model (Fig. 8A). Through the evaluation of the pre-clinical combined treatment, no severe potential toxicity was observed in the MB49-CR xenograft treatment groups (Fig. S7K, L). Up-regulation of circRNA or MR caused slightly decrease of the MB49-CR xenograft. Interestingly, treatment with BCH alone caused the similar reduction of MB49-CR tumor as the combined effect of circRNA over-expression and methionine restriction, indicating the vital role of immuno-regulation in cisplatin resistant microenvironment. Inhibition of SLC7A6 with BCH also showed a synergetic effect with circRNA-overexpression or methionine restriction, while three treatment together exerted the maximum efficacy (Fig. 8B, C, D). MAT2A expression was down-regulated after circRNA over-expression which further determined the down-regulation of CD44 and Ki67. CD8 + T cells infiltration were significantly increased after BCH treatment, while methionine restriction to some extent decreased CD8 + T cells since methionine concentration in BCa microenvironment was decreased (Fig. 8E). We further validated that the tumor volume depended on the CD8 + T cell infiltration and Ki-67 expression. Treatment with circRNA overexpression, BCH and methionine restriction has the most CD8 + T cell infiltration and the lowest amount of Ki-67, which has the most diminished tumor burden. Analysis of the function of tumor infiltration CD8 + T cells also indicated that BCH treatment increased CD8 + T cells function (Fig. 8F).

In concluding, we validated the combination therapy of inhibiting MAT2A regulated methionine metabolism and SLC7A6 mediated CD8 + T cell dysfunction was a promising treatment strategy. The system regulation role of methionine metabolism in the cisplatin resistant BCa microenvironment is presented in Fig. 8G.

## Discussion

The main treatment interventions for the advanced stage of BCa patients are cisplatin based chemotherapy while cisplatin resistance will occur in several months with low efficacy leading to high rate of relapse and metastasis. Thus, the combination therapy of surgical operation with chemotherapy and immunotherapy could only slightly improved the overall-survival of BCa because of the cisplatin resistance [39]. Therefore, it is of urgent need to further discover the underlying mechanisms of cisplatin resistance to discover more effective therapy strategy.

Cancer stem cell, or named as tumor initiating-cells were responsible for the chemotherapy resistance in cisplatin resistant bladder cancer. Previous research has identified that primary cell cultured in organoid forms maintained the cancer ctem cell properties and vulnerability to methionine metabolism, while cells culturing in adherent condition gradually lose the cancer stem cell characteristics [40]. Sustained methionine cycle flux is critical for the induction of cancer stem cells, while methionine starvation for several hours leads to disruption of chemotherapy induced cancer stem cell properties, not any other amino acids [25]. We were further inspired by this finding that disruption of methionine metabolism could overcome cisplatin resistance in bladder cancer cells since the stemness and viability of cancer stem cells rely on methionine metabolism and cisplatin resistant bladder cancer cells inherit a large portion of stemness cells via the selection of cisplatin. Through multi-omics analysis of metabolomics and proteomics of T24 and T24CR cells, we found that MAT2A regulated methionine metabolism is crucial to cisplatin resistance and cancer stem cell properties in BCa cells, mainly though the regulation of production of SAM for methylated histone turnover.

With the research breakthrough of high-throughput technique, a large number of tumor progression related circRNAs was identified and validated in human cells. Our group has identified several BCa related circRNA with distinct regulation pathway [41–44]. In this study, circARHGAP10 was identified as a methionine metabolism related circRNA and was downregulated in BCa tissues. The protein level of MAT2A was frequently regulated via ubiquitin-proteasome pathway. Here, we found that circARHGAP10 functions as the scaffold to promote the E3-ligase TRIM25-mediated MAT2A ubiquitination. TRIM25 exerted its E3-ligase mainly through its RNA-binding regulation. Our work provide a novel regulatory mechanism for MAT2A and circRNAs participating in amino acid metabolism.

Various clinical studies have proven that removal of certain amino acids in daily dietary can benefit the prognosis of cancer [45, 46]. For example, uptake of histidine and asparagine influenced response of patients to the chemotherapy agent methotrexate [47]. Several clinical trials or basic researches have discussed the clinical prospect of methionine restriction. Methionine metabolism contributes to the one-carbon metabolism and consequently create vulnerabilities involving redox or nucleotide metabolism of cancer cells via chemotherapy or radiation therapy [30]. The clinical attempt of methionine restriction was proven safe within patients but could not benefit the long-term survival of cancer patients. Since cisplatin could cause reactive oxygen species aggregated in cancer cells, there is a promising combination therapy effect with methionine restriction for its effect on redox equilibrium. Our group has focused on the circRNA regulation and target-based treatment [48–51]. We further validated that knockdown of MAT2A through overexpression of circARHGAP10 and restriction of methionine up-take was sufficient to overcome cisplatin resistance in vivo in immuno-deficiency model but not in immuno- competent model. This phenomenon has drawn our attention that neglect of the immuno-regulated tumor microenvironment may contribute to the previous low clinical efficacy of methionine restriction.

The activation of CD8 + T cells rely on certain amino acids supplement.

S-adenosylmethionine (SAM) is crucial for the metabolic pathway in cancer stem cells [52]. SAM, converted from methionine, fueled for methyltransferases to generate histone methylation for epigenetic alternation to induce functional CD8 + T cells. Recent research has identified that tumor cells outcompete methionine in tumor microenvironment, thus hamper the methionine metabolism and intracellular levels of methionine of CD8 + T cells, resulting in the down-regulation of H3K79me2 and STAT5 for the eventually impaired T cell function [17]. We further validated that this methionine/SAM/H3K79me2/STAT5 regulation pathway was conserved in CD8 + T cells of cisplatin resistant bladder cancer microenvironment. Cisplatin based chemotherapy plus immunotherapy of immune checkpoint have proven to benefit the outcome in advanced bladder cancer patients. Since the increased methionine consumption in bladder cancer cells can be identified as an immune evasion mechanism, we believed that targeting methionine metabolism would provide new additional approach for the cisplatin resistant bladder cancer. We further validated that SLC7A6 was significantly up-regulated in cisplatin resistant bladder cancer cells versus cisplatin sensitive bladder cancer cells while the expression of SLC7A6 in CD8 + T cells remained relatively at minimum level. Given that SLC7A6 was the only identified SLC family member which has the possibility for methionine transportation, we further discussed the role of SLC7A6 in cisplatin resistant bladder cancer. We found SLC7A6 negatively correlated with CD8 in BCa microenvironment. More importantly, dual inhibition of MAT2A mediated methionine and SLC7A6 activity could overcome cisplatin resistance in immuno-competent model in vivo.

To conclude, our work has introduced the dominant role of methionine in the microenvironment of cisplatin bladder cancer. The combined therapy of down-regulation of methionine flux through MAT2A inhibition or methionine restriction and the inhibition of SLC family SLC7A6 would disrupt the high methionine consumption in cancer cells and preserve the function of CD8 + T cells, which indicates a promising clinical treatment option for cisplatin resistant bladder cancer cell patient.

## Materials and methods

### Cell lines

Human and murine bladder cancer cells SV-HUC-1, RT4, UM-UC-3, T24, 5637, MB49 were obtained and cultured as previously described [44]. For inducing the cisplatin resistant T24, UMUC-3 and MB49 cells, cells were cultured in cisplatin containing medium with the cisplatin concentration increasing from 1 μM to 25 μM for 10 months.

### Tissue microarray and patient samples

The Huashan cohort tissue microarray is composed of 90 pairs of normal and malignant tissues who received radical cystectomy with bladder cancer, between 2007-01 to 2013-01, with a 5-year follow-up [44].

The tissue microarray used in this study came from 90 cases of bladder cancer who received radical cystectomy in our center from January 2007 to January 2013. The inclusion and exclusion criteria are as follows:

1. Inclusion criteria:

- Radical cystectomy was performed at our center, and complete pathological specimens are available. The pathological specimen was confirmed as bladder cancer by two pathologists. The specimen contains tumors and normal tissues.
- Age: 18-80 years old

2. Exclusion criteria:

- The patient’s clinical information is incomplete.
- The quality of pathological samples is unqualified.
- Have a history of bladder perfusion therapy within 3 months before surgery or have adjuvant chemotherapy/radiation therapy within 6 months before surgery.

Written consent was obtained from patients and was approved by the Ethics Committee of Huashan Hospital (approval number KY2011-009, 2022-100). Patient sample for circRNA sequencing and expression analysis were also collected as the same procedure.

### Metabolomics analysis

T24/T24-CR cells and T24-CR-LV-NC/T24-CR-LV-circARHGAP10OE cells were used for metabolomics analysis. Medium was discarded after reaching confluence, and cells were washed with PBS for 3 times. Cells were scraped in 2 mL cooled methanol and lysed to extract metabolites as previously described [44].

### Proteomics analysis

20 µg extracted protein was separated through SDS-PAGE. Protein was digested with trypsin and LC-MS/MS was performed with a timsTOF-Pro spectrometry coupled to Nanoelute (Bruker). Raw protein data was searched with MaxQuant 1.6.14 software for quantitation analysis. Following annotation steps, the proteins were blasted to retrieve their KEGG identifications. Metabolomics and proteomics analysis and the integrated multi-omics analysis of this work were completed in Applied Protein Technology, Shanghai, China.

### RNA-seq

RNA was extracted from five paired of benign and malignant bladder tissues with TRIzol (Invitrogen, USA). 3 mg total RNA was extracted and for further sequencing as previously described [41].

### RNA FISH and immunofluorescence

Probes targeting the junction site of circARHGAP10 were synthesized (GenePharma Biotech, Guangzhou, China). IF and photographed procedure were performed as previously described [41]. All cell were fixed and labeled with DAPI and cell were photographed with the LSM700 confocal microscope (Carl Zeiss, Oberkochen).

### RNase R treatment

Total RNA was extracted and treated with RNase R (Epicenter Technologies, USA). Stability of circRNA and lineal mRNA was analyzed via qRT-PCR [44].

### Actinomycin D assays

2 mg/mL actinomycin D (Sigma) was added to the cells and the BCa cells were collected at indicated time points for further analysis as previously described [41, 44].

### qRT-PCR

RNA was extracted and cDNA was obtained by using the PrimeScriptTMRT Reagent Kit (TaKaRa,Japan) to perform qRT-PCR on an ABI 7900HT machine (Thermo Fisher Scientific, USA). 2-ΔΔCT was used to analyze RNA expressions [44].

### Vector construction and cell transfection

shRNAs sequences were designed to direct target circRNAs, MAT2A, TRIM25 or SLC7A6 were cloned and inserted into pGPU6/GFP/puromycin vector. circRNA sequence was cloned and insert into a plenti-ciR-GFP-T2A vector from IGEbio (Guangzhou, China) to construct circRNA over-expression plasmid. To overexpress MAT2A or SLC7A6, the vector pENTER with identical gene or control was obtained from Vigene (Shanghai, China). Flag-TRIM25, Flag-Trim25ΔRBD, Myc-MAT2A, HA-Ub and the K6, K11, K27, K48 and K63 mutant plasmid was purchased from Vigene (Shanghai, China). The siRNAs were purchased from GenePharma (Shanghai, China).The cell transfection were described as our previous work [43].

### Sphere formation assay

BCa cells were digested and suspended as single cells in the 6-well ultra-low attachment plates, supplemented with 5 ng/mL recombinant EGF (Sangon, China). The sphere formation was observed 12 days later.

### Cell proliferation assay

In CCK-8 assay, cells were digested and seeded at 1,500 cells per well into a 96-wells plate. We detected the OD450 value at various time points after seeding into the wells. For IC50 test, cells were incubated with cisplatin and the relative viability rate was calculated in Graphpad Prism 8 to calculate the IC50 value. For the colony-formation assay, cells were digested and seeded into 6-well plate and incubated for 14 days. The colonies were imaged after stained with 0.2% crystal violet.

### Apoptosis and EdU assay

Apoptosis assay was performed with the apoptosis kit (MultiSciences, China) as our previous work [41]. EdU assay was performed with EdU kit (RiboBio, China) to detect DNA synthesis [44].

### Transwell assays

Cells were stained by 0.2% crystal violet in a Transwell chamber (CoStar, USA). Five randomly selected sections of cells were analyzed for generating statistical analysis.

### Molecular docking

We used Autodock Vina 1.2.2 to analyzed the binding affinities between proteins. The 3D coordinates of the proteins were downloaded from the PDB (http://rcsb.org/PDB). Molecular docking was visualized and performed with AutodockVina 1.2.2 (http://autodock.scripps. edu).

### Co-immunoprecipitation

For co-IP experiment, relative cells were lysed with cooled supplemented with a combination of proteinase and phosphatase inhibitors, with or without RNase inhibitor. The supernatant was incubated with antibody conjunctive or antibody pre-incubated beads. The beads were washed and centrifuged with co-IP buffer for further western blot analysis [53].

### RNA pull-down assay and RIP assay

Streptavidin beads (Life Technologies, USA) were treated with biotin labeled circARHGAP10 probes with oligo probes to perform RNA pull-down assay. The magnetic RIP kit (Millipore) was used to perform the RIP assay according to the instructions of the manufacture described as our previous work [43].

### Western blot analysis

Protein were lysed from cells with RIPA buffer and separated with SDS-PAGE. The blots were blocked and incubated with antibodies overnight. Antibody: GLDC (Abcam, ab232989), SHMT2 (Abcam, ab180786), MAT2A (Proteintech, 55309-1-AP), MTHFR (Abcam, ab203786), SAHH (Abcam, ab151734), CD44 (Abcam, ab243894), Nanog (Abcam, ab109250), H3 (Abcam, ab1791), H3K4me3 (Abcam, ab213224), H3K9me3 (Abcam, ab8898), H3K27me3 (abcam, ab6002), H3K36me2 (Abcam, ab176921), and H3K36me3 (Abcam, ab9050), H3K79me2 (Abcam, ab3594), and H3K79me3 (Abcam, ab2621), Flag/DDDDK tag (Abcam, ab205606), Myc tag (Abcam, ab32), HA tag (Abcam, ab9110), TRIM25(Abcam, ab167154), USP5(Abcam, ab154170), USP14(Abcam, ab192618), p-STAT5(Abcam, ab32364), STAT5(Abcam, ab230670), SLC7A6(Abcam, ab235054), GAPDH (Proteintech, 60004-1-lg) was used as loading control. The signals were visualized using an ECL imaging system (CLiNX, Shanghai) as previously described [44].

### Compounds and reagents

The compounds or enzymes used in this work are as follows: cisplatin (Selleck, USA), FIDAS (Selleck, USA), MeAIB (Selleck, USA), BCH (Selleck, USA), RNase A (Yeason, China), SAM (Sigma, USA), SAH (Sigma, USA) and HCY (Sigma, USA). Medium without certain amino acids were purchased from US Biological,USA. Amino acids were purchased from Sangon, China.

### In vitro CD8 + T cell preparation and activation

Fresh tumor samples were obtained soon after sacrificed of the animals. Tumors were minced and digested with a combination of collagenase I (Sangon,China) and DNase I (Sangon, China). PBMCs were derived from patients with Ficoll (GE Healthcare, USA). The cells were stimulated, stained with membrane markers and applied to MACS column for further analysis.

### Flow cytometry

CD8 + T cells exacted from peripheral blood or cancer tissue were collected. The single-cell suspensions of CD8 + T cells were stained with surface markers with CD45 (BD Biosciences, 557833), CD3 (BD Biosciences, 552852), CD8 (BD Biosciences, 564526) and PD-1 (BD Biosciences, 561272) [44]. BD Cytofifix/Cytoperm Fixation/Permeabilization Solution Kit (BD Biosciences) was used to stain intracellular markers including IFN-g (BD Biosciences, 557643), GZMB (BD Biosciences, 560212), and IL-2(BD Biosciences, 554566). CD8 + T cells were analyzed on the BD FACS Verse Flow Cytometer as we previously described [44].

### Animal study

For the subcutaneous T24-CR cell xenograft model, 4-week-old nude male athymic BALB/c mice (SLARC, Shanghai, China) were maintained in specific pathogen free culture environment. The control and methionine restriction diets were purchased from Xietong (Jiangsu, China) which control diet contained 0.86% methionine nutrition and MR diet contained 0.12% methionine nutrition in our study [30]. The mice were randomly selected into four groups. A total of 1 × 10^7^ T24-CR-luc-LV-NC or T24-CR-luc-LV-circ cells were injected subcutaneously into nude mice fed with control or MR diets. Cisplatin (6 mg/kg) was injected intraperitoneally one week after cell implantation (one injection for every 5 days). Tumor volume was monitored every 3 days with the calculation of tumor volume = width^2^ × length/2. All mice were sacrificed after 6 weeks of drug treatment. The extreme limiting dilution analysis were performed with 5 × 10^6^, 5 × 10^5^ and 5 × 10^4^ T24-CR cells subcutaneously injection into the nude mice to calculate the successful rate of the tumor implantation.

For the patient-derived xenograft (PDX) models, two fresh fragments of tissue of BCa patients were transplanted subcutaneously into 4-week-old NOD/SCID mice (SLARC, Shanghai, China). When the 1^st^ generation of the xenografts reached approximately 100 mm^3^, the PDX tissue block were isolated and mechanically dissolved into 1 mm^3^ tissue blocks for the subcutaneously implantation into NOD/SCID mice for the 2nd PDX generation. The 2nd PDX generation was divided randomly into four groups. The PDX xenografts were injected with 5 nmol in vivo-grade cholesterol-conjugated si-NC or si-circARHGAP5 (RiboBio, Guangzhou, China) fed with control or MR diets. Cisplatin (6 mg/kg) was injected intraperitoneally one week after cell implantation (one injection for every 5 days). Tumor volume was monitored every 3 days with the calculation of tumor volume = (width^2^ × length/2). All mice were sacrificed after 6 weeks of 2nd PDX implantation.

For the lung metastasis model of T24-CR cell, 1 × 10^5^ T24-CR-luc-LV-NC or T24-CR-luc-LV-circ cells were injected intravenously into the tails of 4-week-old nude male athymic BALB/c mice (SLARC, Shanghai, China) fed with control or MR diets. Cisplatin (6 mg/kg) was injected intraperitoneally one week after cell injection (one injection for every 5 days). After 30 days, metastasis of the tumor development was monitored via IVIS following intraperitoneal injection of 200 mg/kg luciferin.

For the subcutaneous MB49-CR cell xenograft, 4-week-old male C57BL/6 mice (SLARC, Shanghai, China). A total of 1 × 10^7^ MB49-CR-LV-NC or MB49-CR-LV-circ cells were injected subcutaneously into mice fed with control or MR diets. Anti-PD-L1 and IgG1 (Bioxcell) (100 μg/mouse) were injected intraperitoneally one week after cell implantation (one injection for every 3 days). BCH (180 mg/kg) was injected intravenously one week after cell implantation (one injection for every 3 days). Cisplatin (6 mg/kg) was injected intraperitoneally one week after cell implantation (one injection for every 5 days). Tumor volume was monitored every 3 days with the calculation of tumor volume = (width^2^ × length/2). All mice were sacrificed after 6 weeks of drug treatment. All animal experiments including in this study were approved with the approve number 202212002 S.

### IHC

Tumor tissue samples and mouse liver tissue were embedded in paraffin while IHC and H&E staining was performed [44, 54]. The primary antibodies were Ki-67 (Abcam, ab270650), MAT2A (Proteintech, 55309-1-AP), SLC7A6(Abcam, ab235054), CD44 (Abcam, ab243894) and CD8A (Abcam, ab217344).

### Statistical analysis

The statistical analysis was analyzed with Student’s *t*-test or the Chi-square test in Graphpad Prism 8 [55]. K-M curve was applied for the overall survival analysis [56, 57]. We determined that *p* < 0.05 was statistically significant.

### Reporting summary

Further information on research design is available in the Nature Research Reporting Summary linked to this article.

## Supplementary information

Supplementary Table 1

Supplementary files

Original Data File

primers

Reporting Summary

## Data Availability

All data used and analyzed in this study are available under reasonable request.
