# Supplementary material for: Methionine orchestrates the metabolism vulnerability in cisplatin resistant bladder cancer microenvironment
Source: Cell Death Dis. 2023 Aug 15;14(8):525. doi: 10.1038/s41419-023-06050-1 (PMC10427658; doi:10.1038/s41419-023-06050-1)
Supplement: Supplementary file 1 — Supplementary Table 1 [file 41419_2023_6050_MOESM1_ESM.doc]

**Table S1** Relationship between circARHGAP10 and clinico-pathological features in bladder cancer tissue microarray

| Characteristics | No. (%) | circARHGAP10 expression | | |
| --- | --- | --- | --- | --- |
|  |  | Low (%) | High (%) | *P*-value |
| **Gender** | | | | |
| Male | 81 (90.0) | 48 (59.3) | 33 (40.7) | 0.667 |
| Female | 9 (10.0) | 6 (66.7) | 3 (33.3) |  |
| **Age** | | | | |
| <65 | 56 (62.2) | 34 (60.7) | 22 (39.3) | 0.859 |
| ≥65 | 34 (37.8) | 20 (58.8) | 14 (41.2) |  |
| **Tumor size** | | | | |
| <3 cm | 38 (42.2) | 17 (44.7) | 21 (55.3) | 0.012 |
| ≥3 cm | 52 (57.8) | 37 (71.2) | 15 (28.8) |  |
| **Clinical stage** | | | | |
| T1 | 44 (48.9) | 19 (43.2) | 25 (56.8) | 0.001 |
| T2-T4 | 46 (51.1) | 35 (76.1) | 11 (23.9) |  |
| Total | | | | |
|  | 90 | 54 | 36 |  |
