# Supplementary material for: Methionine orchestrates the metabolism vulnerability in cisplatin resistant bladder cancer microenvironment
Source: Cell Death Dis. 2023 Aug 15;14(8):525. doi: 10.1038/s41419-023-06050-1 (PMC10427658; doi:10.1038/s41419-023-06050-1)
Supplement: Supplementary file 2 — Supplementary files [file 41419_2023_6050_MOESM2_ESM.docx]

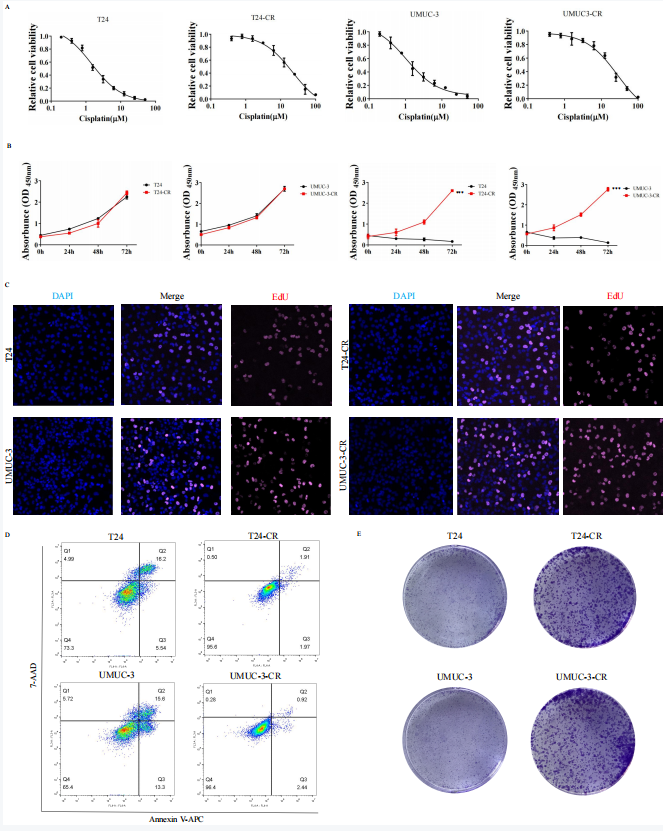


**Figure S1. Characteristic of cisplatin resistant BCa cells.**

(A)Relative cell viability of cisplatin treated BCa cell. (B) Growth curve of BCa cells or 10μM cisplatin treated BCa cell. (C) EdU assay of 10μM cisplatin treated BCa cell. ***p < 0.001 versus T24 or UMUC-3. (D) Cell apoptosis of 10μM cisplatin treated BCa cell. (E) Cloning formation of 10μM cisplatin treated BCa cell.


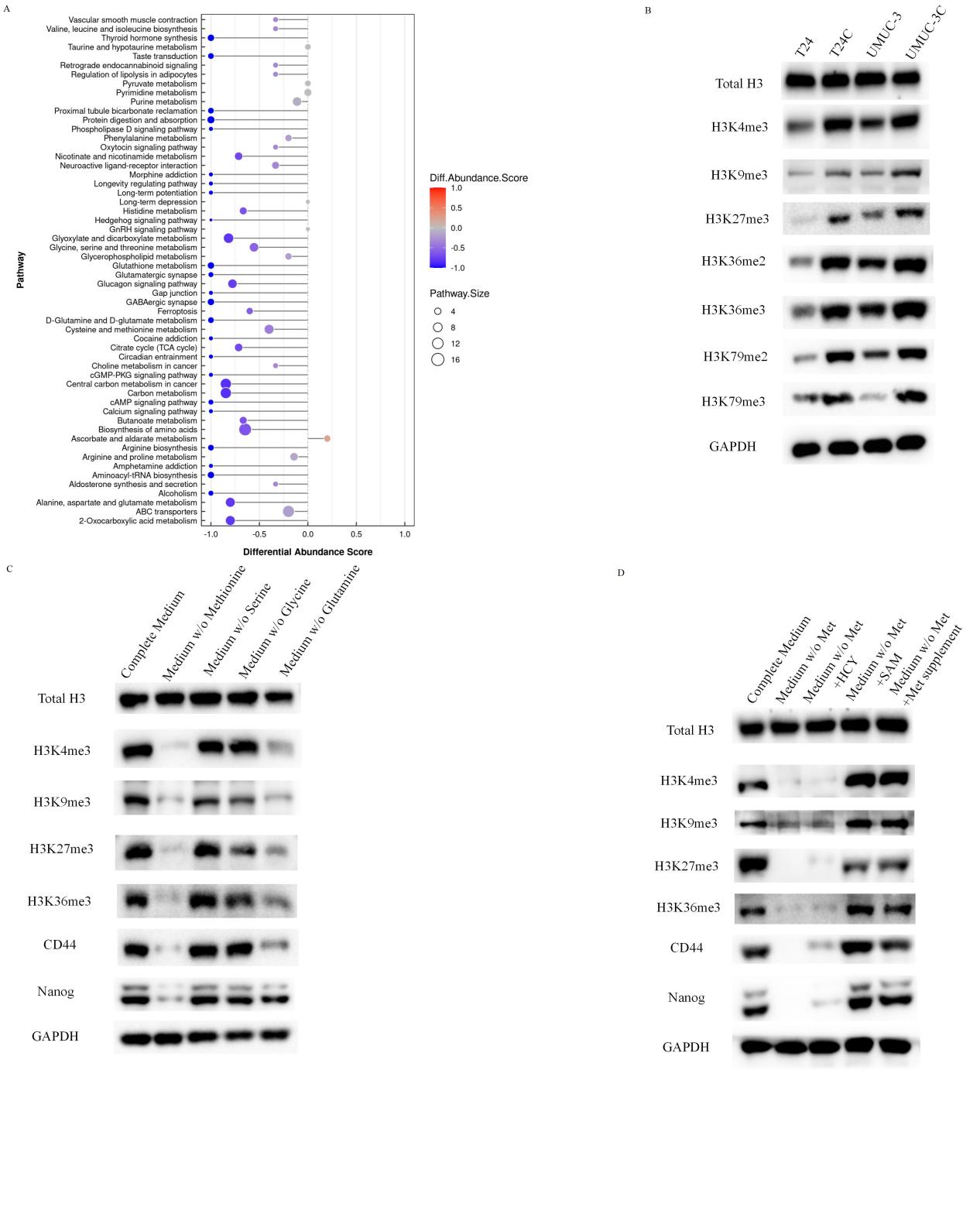


**Figure S2. Methionine metabolism plays a vital role in cisplatin resistant BCa cells.** (A) Enriched pathway analysis of proteomics of T24 versus T24CR cells. (B) Western blot analysis of the histone methylated markers of the BCa cells. (C)Western blot analyses of histone methylated markers and stem cell markers in BCa cells for 48h starvation of indicated metabolite. (D)Western blot analysis of histone methylated markers and stem cell markers in BCa cells with 48 h starvation for methionine and supplemented with 250 µM homocysteine, 500 µM SAM or 48h with complete medium supplement.

**
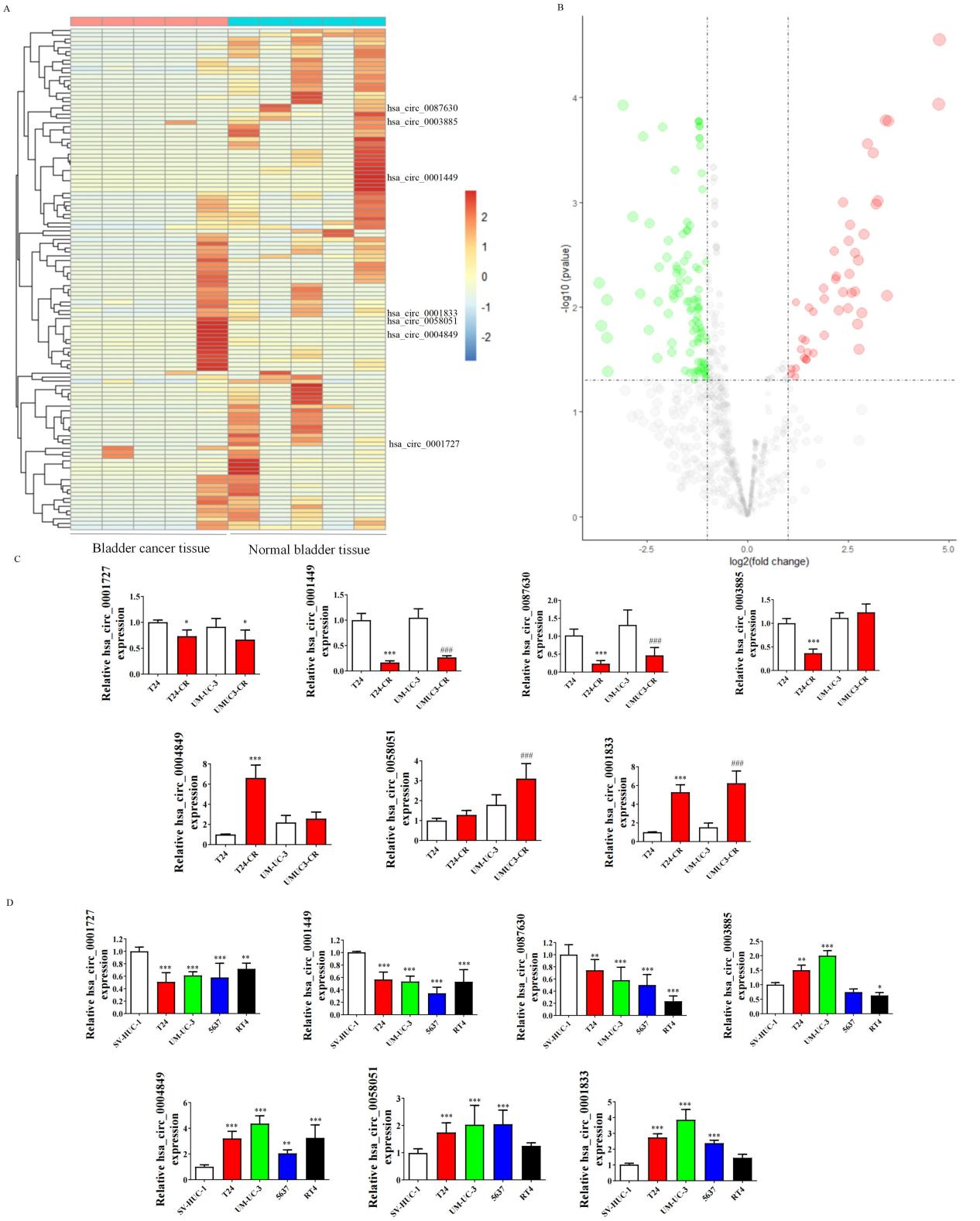
**

**Figure S3. Identification of differential expressed circRNAs between cisplatin resistant cancer cells.** (A and B) Differential circRNA expression in 5 pairs of BCa tissues. (C) Relative circRNA expression in BCa cells and cisplatin BCa cells. ***p < 0.001 versus T24. ^###^p < 0.001 versus UMUC-3. (D) Relative circRNA expression in SV-HUC-1 and BCa cells. ***p < 0.001, **p < 0.01, *p < 0.05 versus SV-HUC-1.

**
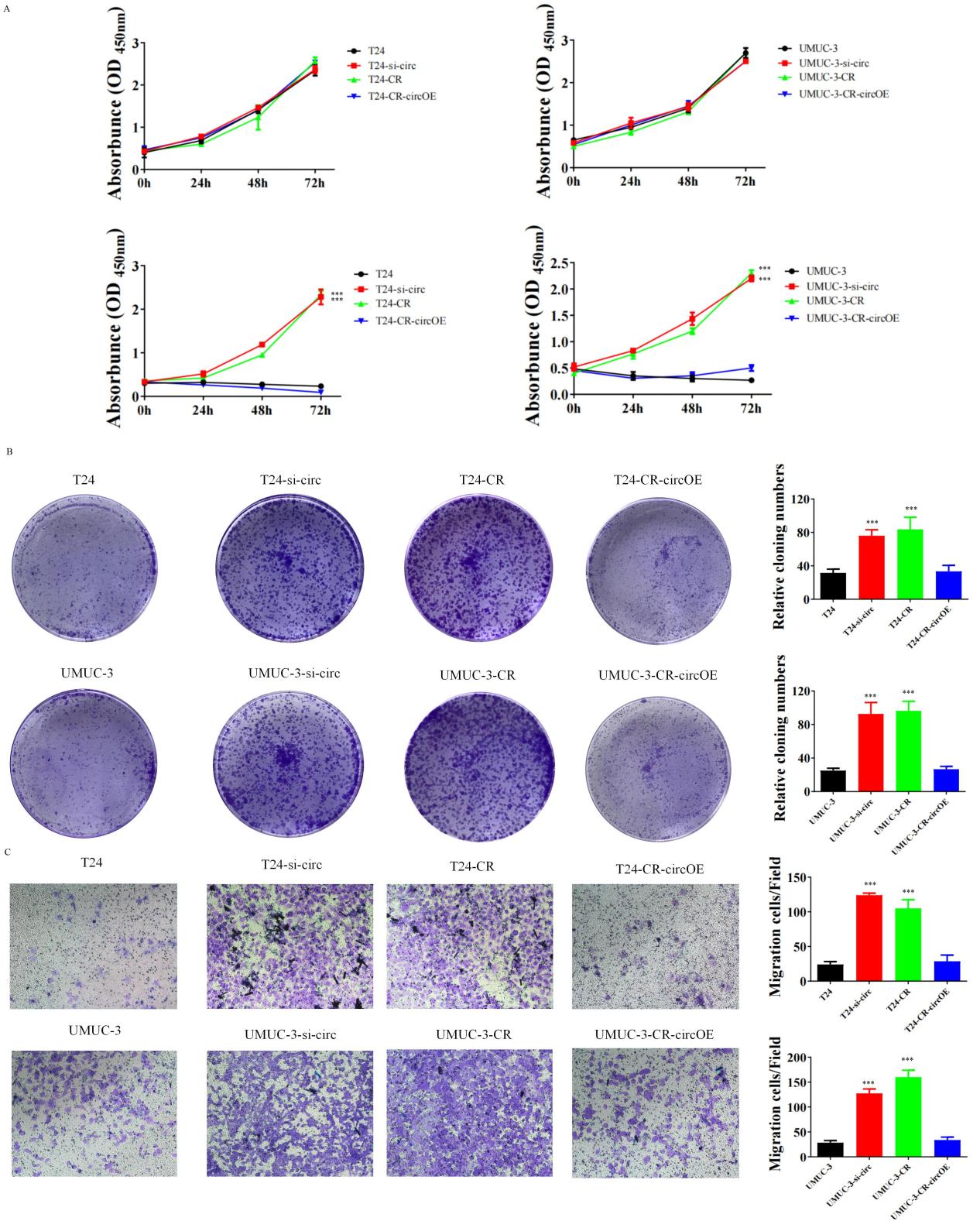
**

**Figure S4. circARHGAP10 influenced the phenotype of cisplatin resistant BCa cells.** (A) Growth curves of BCa cells treated with siRNA of circARHGAP10 or cisplatin BCa cells treated with overexpression of circARHGAP10 in complete medium (upper panel) or 10μM cisplatin containing meium (lower panel). ***p < 0.001 versus T24 or UMUC-3. (B) Cloning formation of BCa cells treated with siRNA of circARHGAP10 or cisplatin resistant BCa cells treated with overexpression of circARHGAP10 in complete medium or 10μM cisplatin containing meium. ***p < 0.001 versus T24 or UMUC-3. (C) Transwell assay of BCa cells treated with siRNA of circARHGAP10 or cisplatin resistant BCa cells treated with overexpression of circARHGAP10 in complete medium or 10μM cisplatin containing medium. ***p < 0.001 versus T24 or UMUC-3.


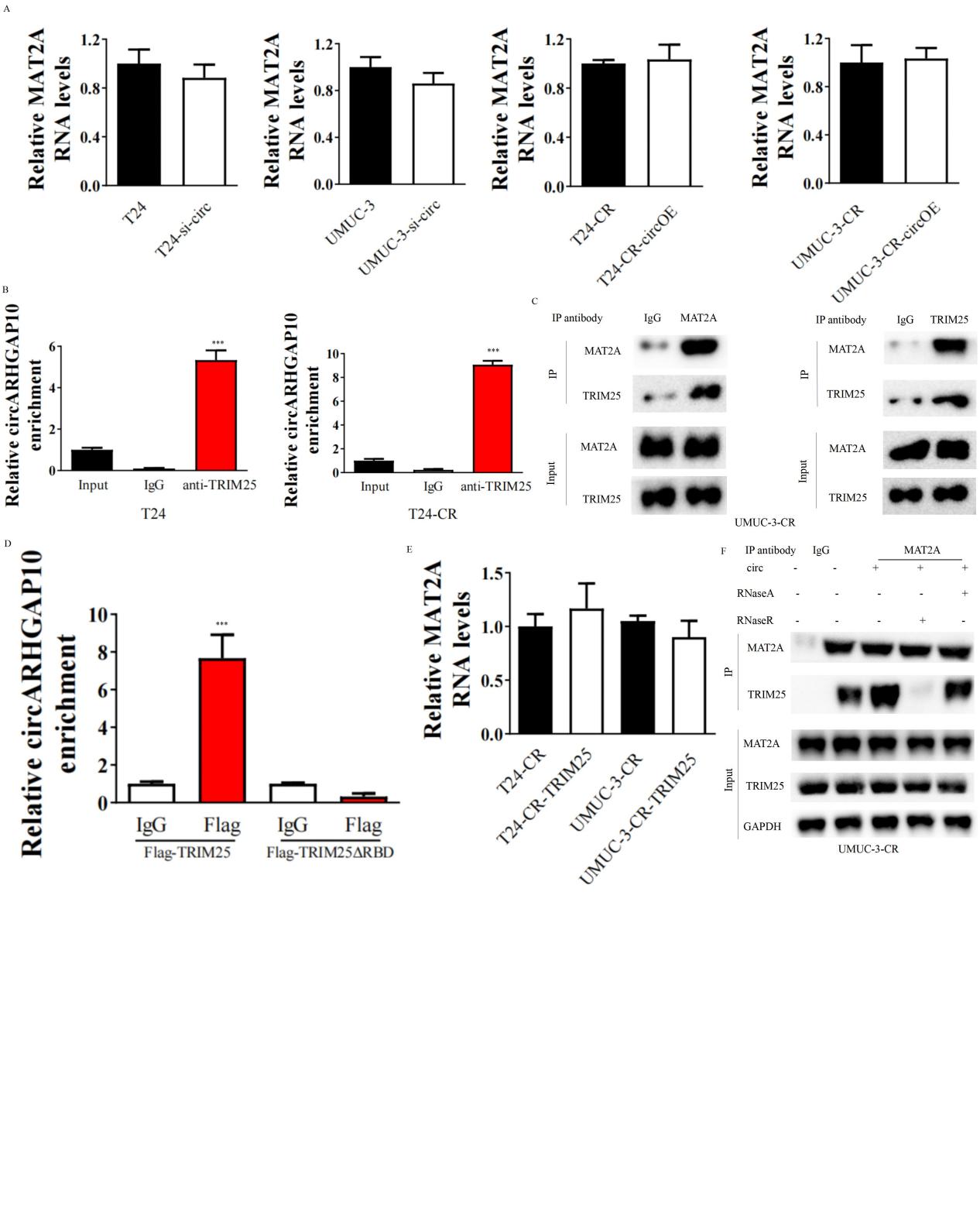


**Figure S5. Interaction of circARHGAP10, MAT2A and TRIM25** (A)Relative MAT2A mRNA expression of BCa cells treated with siRNA of circARHGAP10 or cisplatin BCa cells treated with overexpression of circARHGAP10. (B)Analysis of circARHGAP10 enrichment through RIP assay in BCa cells with anti-TRIM25 antibody. ***p < 0.001 versus input group. (C)Co-IP validated the binding of TRIM25 with MAT2A in UMUC-3-CR cells. (D)Analysis of circARHGAP10 enrichment through RIP assay in BCa cells with anti-FLAG antibody. ***p < 0.001 versus IgG group. (E) Relative MAT2A mRNA expression of cisplatin resistant BCa cells with TRIM25 over-expression. (F) Co-IP experiment was performed to validate the correlation of circARHGAP10 with binding of TRIM25 with MAT2A in UMUC-3-CR cells. RNase A (10 μg/ml) and RNase R (100 U/ml) were the indicated treatment concentration.

**
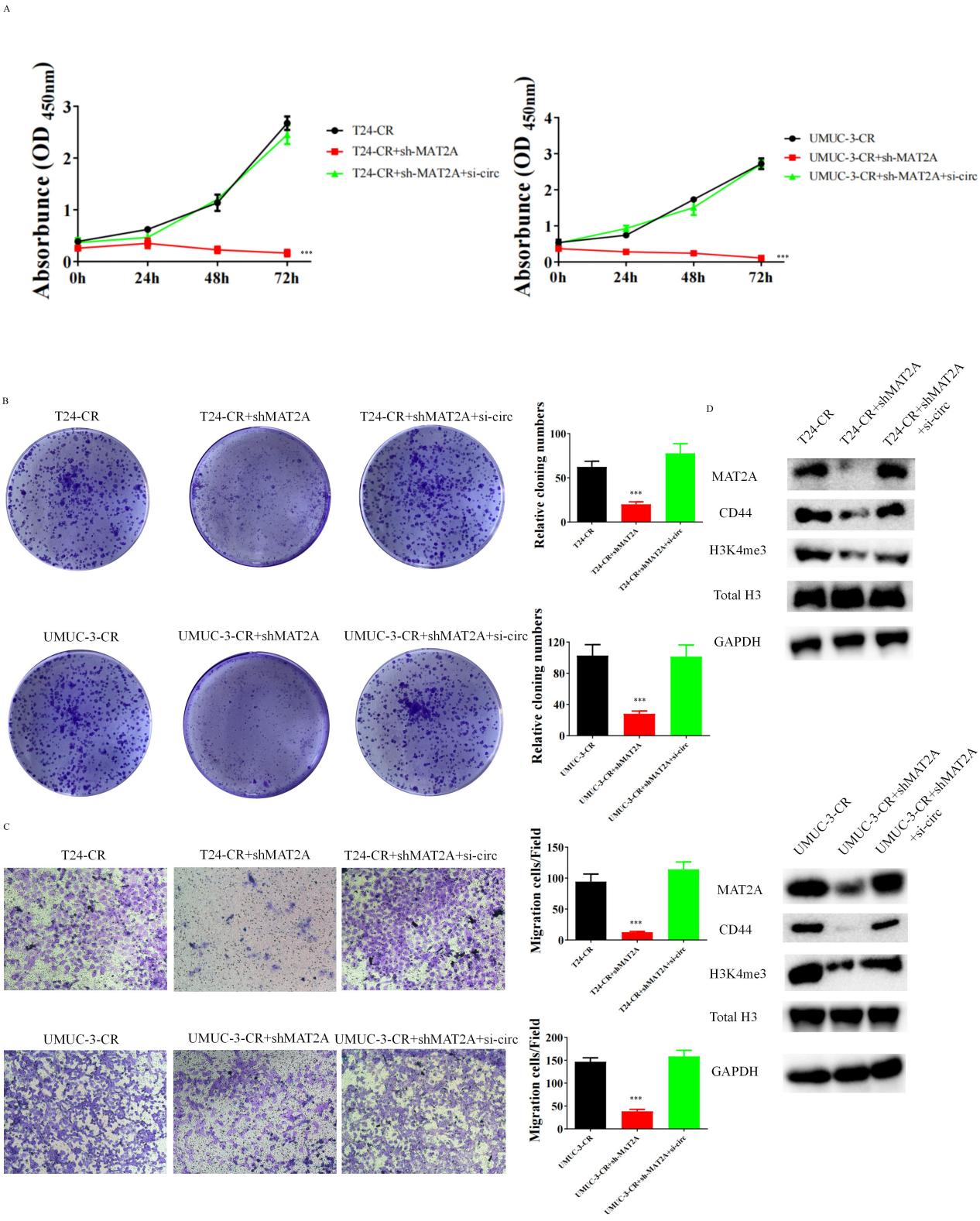
**

**Figure S6. circARHGAP10 modulates cisplatin resistance of BCa cells through MAT2A.** (A) Growth curves of BCa cells treated with sh-MAT2A or with silencing circARHGAP10 with 10μM cisplatin containing medium. ***p < 0.001 versus T24-CR or UMUC-3-CR. (B) Cloning formation of BCa cells treated with sh-MAT2A or with silencing circARHGAP10 with 10μM cisplatin containing medium. ***p < 0.001 versus T24-CR or UMUC-3-CR. (C) Transwell assay of BCa cells treated with sh-MAT2A or with silencing circARHGAP10 with 10μM cisplatin containing medium. ***p < 0.001 versus T24-CR or UMUC-3-CR. (D) Relative protein expression of MAT2A, CD44, H3K4me3 of BCa cells treated with sh-MAT2A or with silencing circARHGAP10 with 10μM cisplatin containing medium.


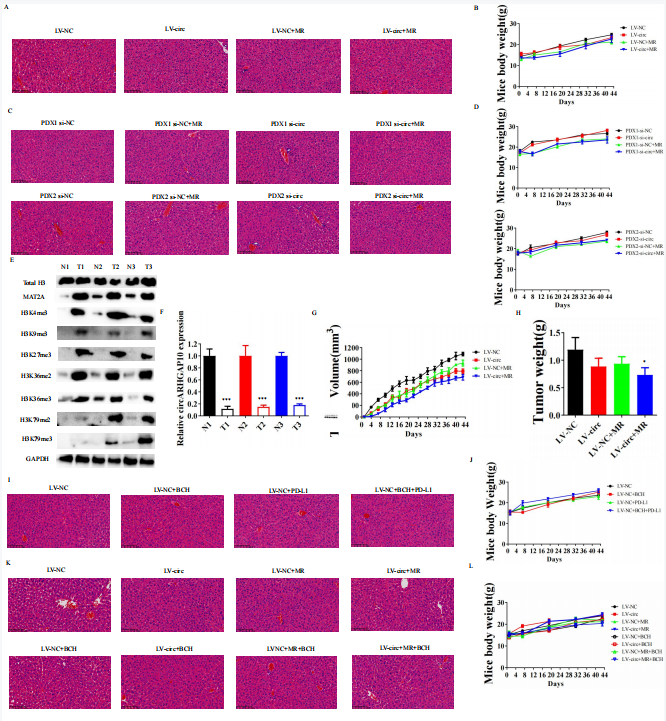


**Figure S7. Potential toxicity of the combination treatment of methionine metabolism inhibition in vivo model.**

(A)H&E staining of the liver tissue of LV-NC, LV-circ, LV-NC+MR and LV-circ+MR from the treatment group in Figure 5A. (B) Mice body weight of the treatment group in Figure 5A. (C)H&E staining of liver of PDX si-NC, si-circ, si-NC+MR and si-circ+MR from the treatment group in Figure 5F. (D) Mice body weight of the treatment group in Figure 5F.

(E)Protein expression of MAT2A and histone methylation level in paired cisplatin treatment naive bladder cancer and cisplatin treated bladder cancer. (F) Relative circARHGAP10 expression in paired cisplatin treatment naive bladder cancer (N) and cisplatin treated bladder cancer (T).Data are presented as the mean ± SD. ***p < 0.001 versus N.(G) 1×10^7^ MB49-CR-luc-LV-NC or MB49-CR-luc-LV-circ cells were injected subcutaneously into 4-week-old male C57BL/6 mice under 6mg/kg cisplatin treatment fed with control or MR diets. (6 mice was enrolled in each treatment group). (H)Tumor weight were measured separately. Data are presented as the mean ± SD. *p < 0.05 versus LV-NC.(I)H&E staining of the liver tissue of LV-NC, LV-NC+PD-L1, LV-NC+BCH and LV-NC+PD-L1+BCH from the treatment group in Figure 7J. (J) Mice body weight of the treatment group in Figure 7J.(K)H&E staining of the liver tissue from the treatment group in Figure 8A. (L) Mice body weight of the treatment group in Figure 8A.

**
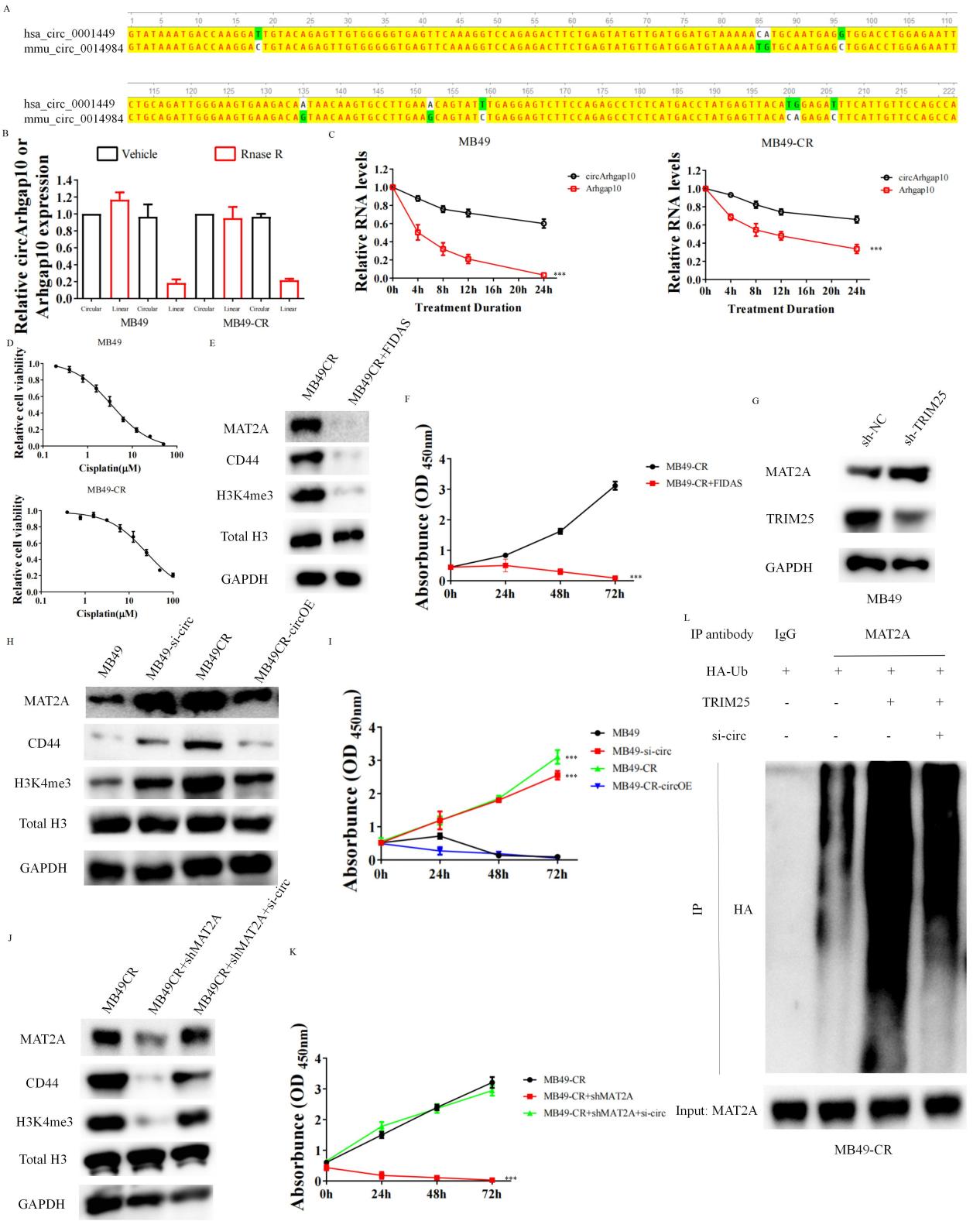
**

**Figure S8. The circARHGAP10/MAT2A/TRIM25 regulation pathway was preserved in cisplatin resistant mouse derived MB49 cells.** (A) The sequence similarity of human circARHGAP10 and conserved mouse circArhgap10. (B) Expression of circular and linear forms of transcripts in MB49 cell with RNase R treatment. (C) Expression of circular and linear forms of transcripts in MB49 cell after treatment by actinomycin D.***p < 0.001 versus circArhgap10. (D) Cell viability of MB49 or MB49-CR cells under 10μM cisplatin treatment. (E) Protein expression of MAT2A, CD44 and H3K4me3 of 5μM FIDAS treatment in MB49CR cells. (F) Growth curve of MB49-CR cells under 10μM cisplatin treatment or with 5μM FIDAS treatment.***p < 0.001 versus MB49-CR. (G) MAT2A expression in MB49 cell with TRIM25 silencing. (H) Western blot analysis of MAT2A, the histone methylated markers and stem cell markers of MB49, MB49-si-circArhgap10, MB49-CR, MB49-CR-LV-circArhgap10 cells. (I) Growth curve of MB49, MB49-si-circArhgap10, MB49-CR, MB49-CR-LV-circArhgap10 cells under 10μM cisplatin treatment.***p < 0.001 versus MB49. (J)Western blot analysis of MAT2A, the histone methylated markers and stem cell markers of MB49-CR cells treated with sh-MAT2A or with silencing circArhgap10 with 10μM cisplatin containing medium. (K) Growth curve of MB49-CR cells treated with sh-MAT2A or with silencing circArhgap10 with 10μM cisplatin containing medium. ***p < 0.001 versus MB49-CR.(L) Co-IP experiment was performed to identify the ubiquitination level of MAT2A in MB49-CR cells with the over-expression of TRIM25 or the silencing of circArhgap10.

**
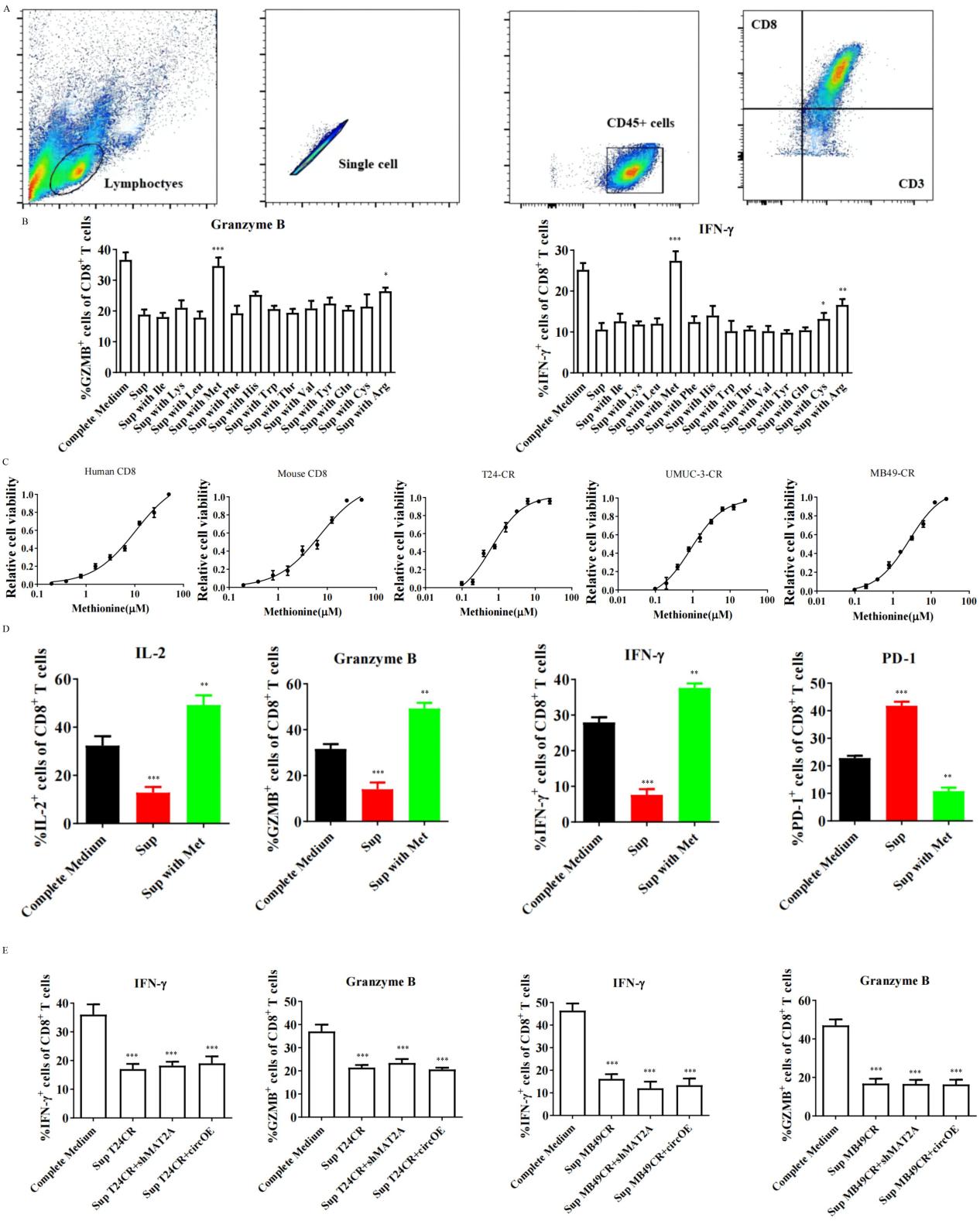
**

**Figure S9.** **BCa cells outcompete CD8+ T cells for methionine in cisplatin resistant bladder cancer microenvironment.** (A)Gating strategy for CD8+ T cells. (B)Function of CD8+ T cells after several amino acid supplementation of supernatant in bladder cancer cell. ***p < 0.001, **p < 0.01,*p < 0.05 versus sup. (C)Relative cell viability of CD8+ T cells and bladder cancer cells in various concentrations of methionine. (D)Function of CD8+ T cells after supplementation of supernatant of T24-CR or with methinone (30μM). ***p < 0.001, **p < 0.01 versus complete medium. (E)Function of CD8+ T cells after supplementation of supernatant of bladder cell with MAT2A silencing or circRNA overexpression. ***p < 0.001 versus complete medium.

**
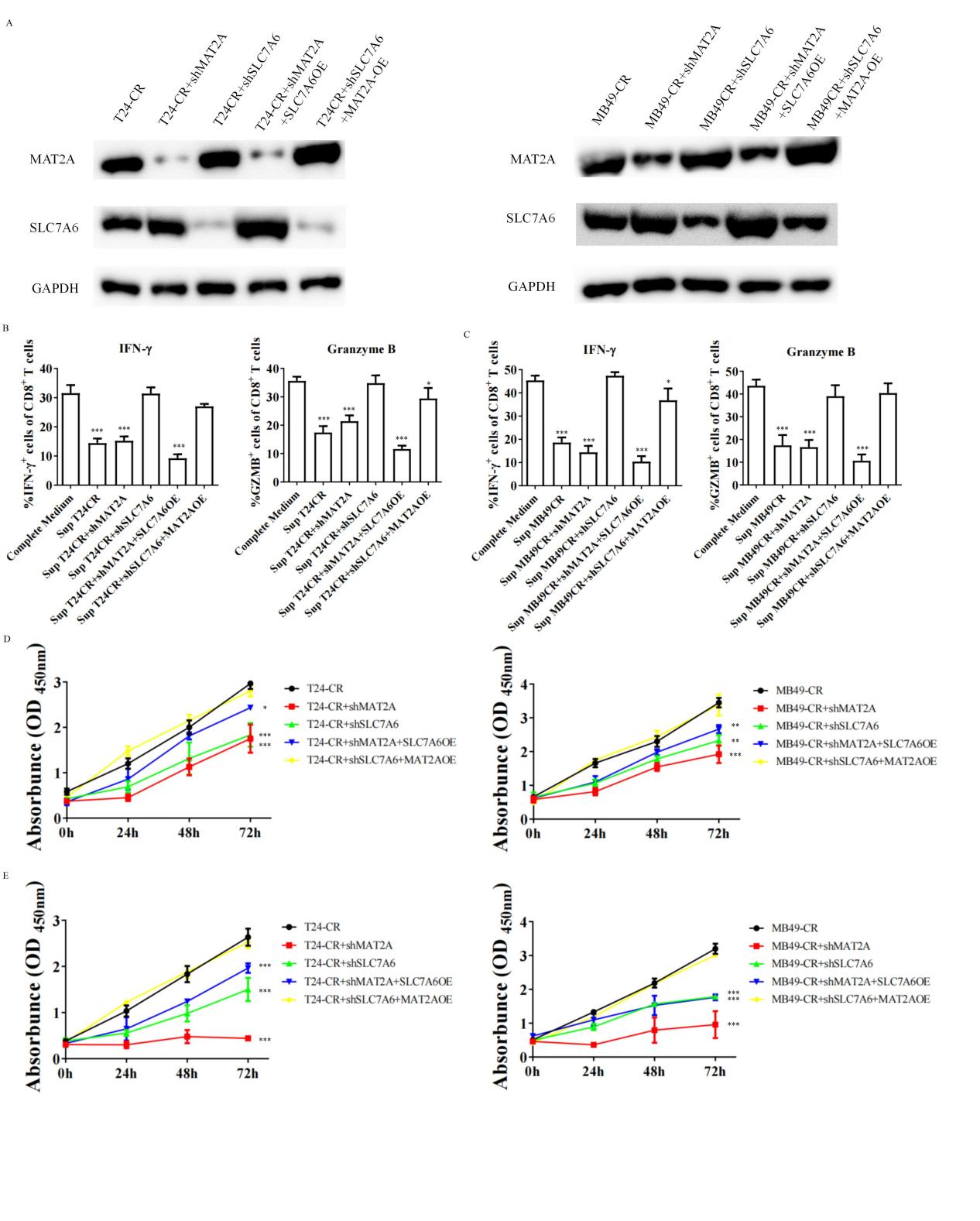
**

**Figure S10. The relationship of MAT2A and SLC7A6 on regulating the viability of BCa cells and function of CD8+ T cells.** (A) Protein expression of MAT2A and SLC7A6 in silencing MAT2A or SLC7A6 and overexpression of SLC7A6 or MAT2A in T24CR or MB49CR cells. (B) Function of CD8+ T cells after supplementation of supernatant of silencing MAT2A or SLC7A6 and overexpression of SLC7A6 or MAT2A in T24CR or MB49CR cells. ***p < 0.001, *p < 0.05 versus complete medium. (C) Growth curve of silencing MAT2A or SLC7A6 and overexpression of SLC7A6 or MAT2A in T24CR or MB49CR cells. ***p < 0.001, **p < 0.01, *p < 0.05 versus T24-CR or MB49-CR cells. (D) Growth curve of silencing MAT2A or SLC7A6 and overexpression of SLC7A6 or MAT2A in T24CR or MB49CR cells with 10μM cisplatin containing medium. ***p < 0.001 versus T24-CR or MB49-CR cells.
