# Supplementary material for: Methionine orchestrates the metabolism vulnerability in cisplatin resistant bladder cancer microenvironment
Source: Cell Death Dis. 2023 Aug 15;14(8):525. doi: 10.1038/s41419-023-06050-1 (PMC10427658; doi:10.1038/s41419-023-06050-1)

Figure 1H SHMT2

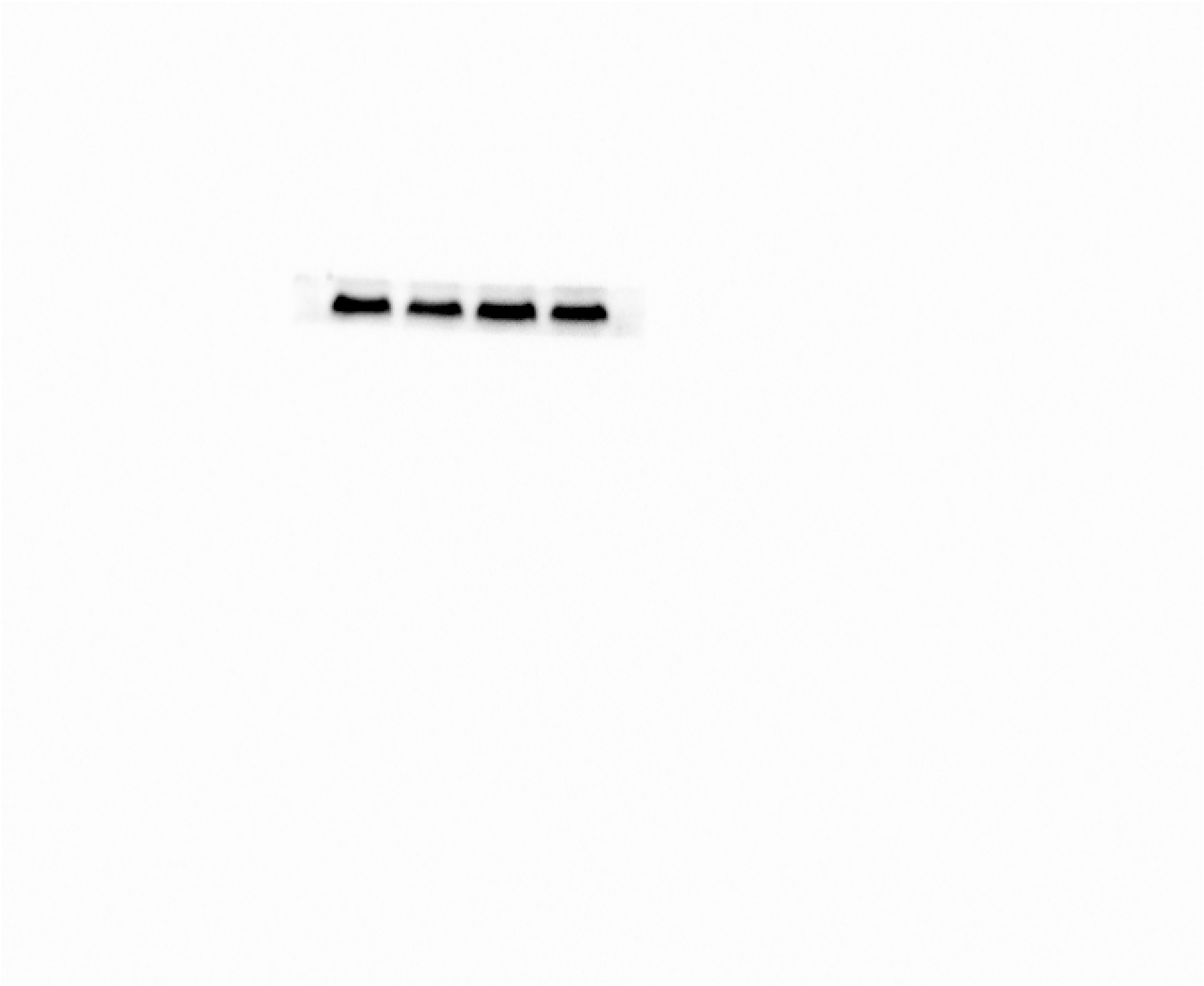

Figure 1H CD44

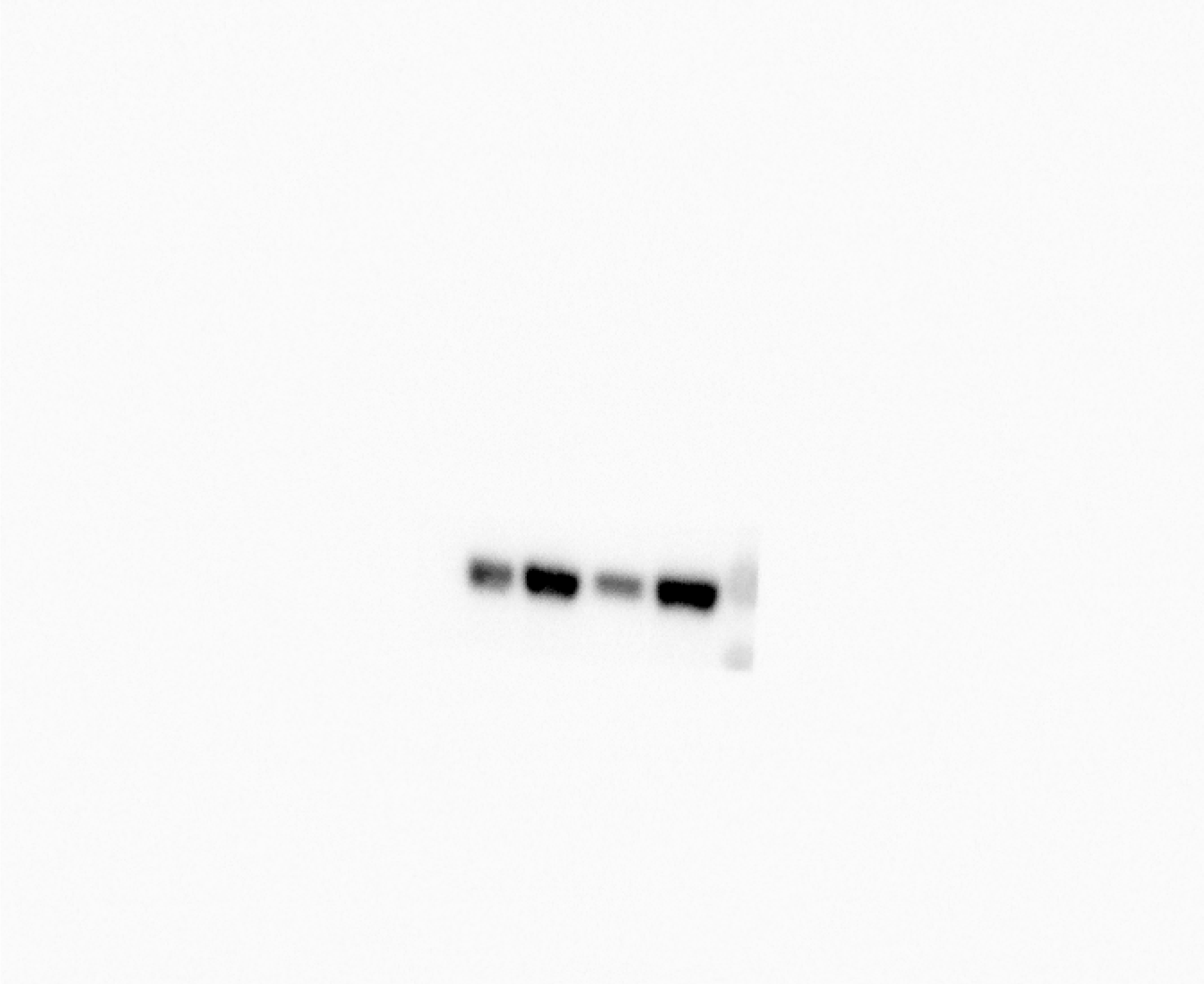

Figure 1H GAPDH

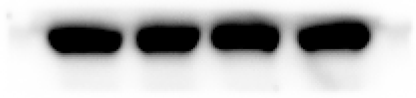

Figure 1H GLDC

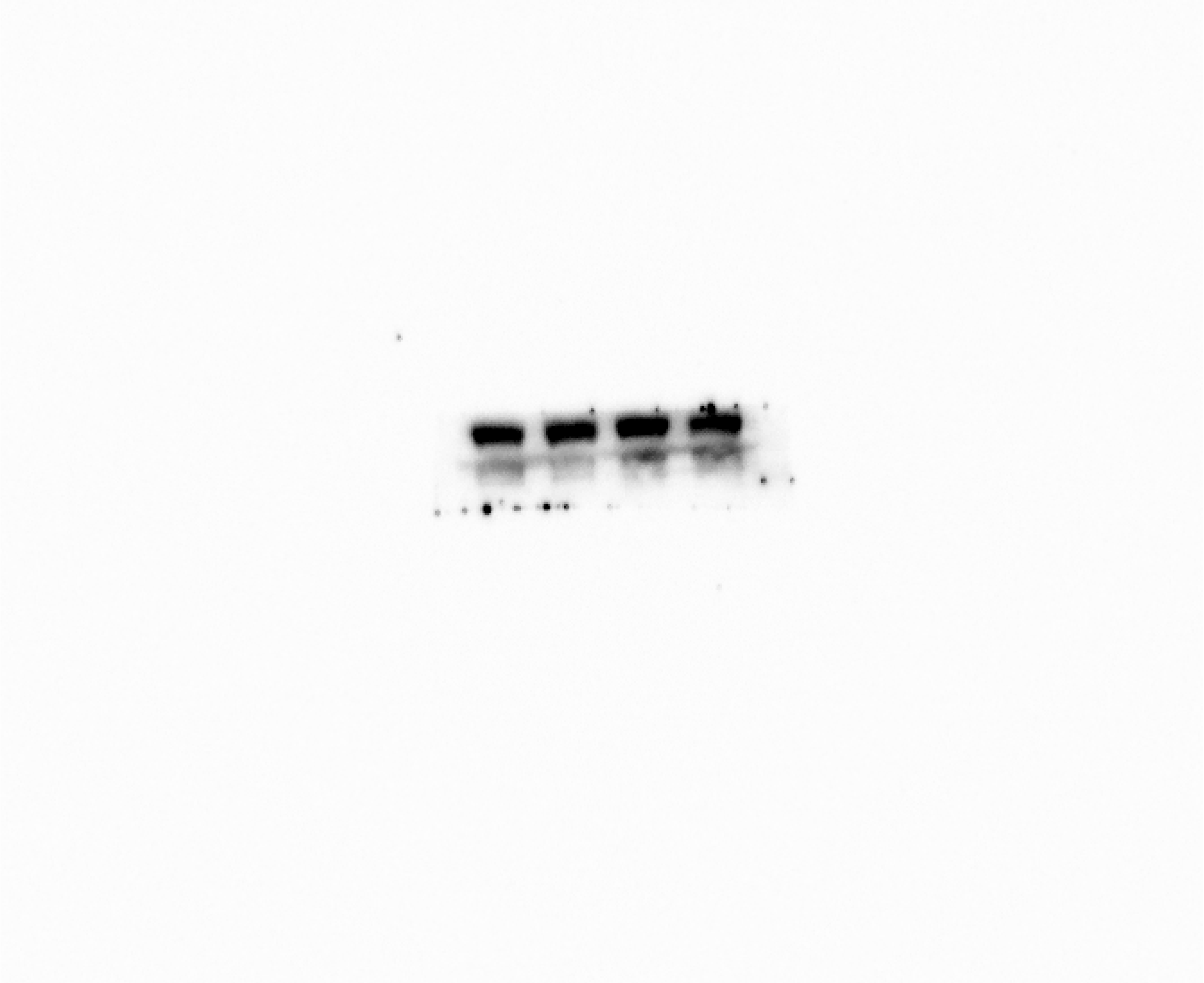

Figure 1H MAT2A

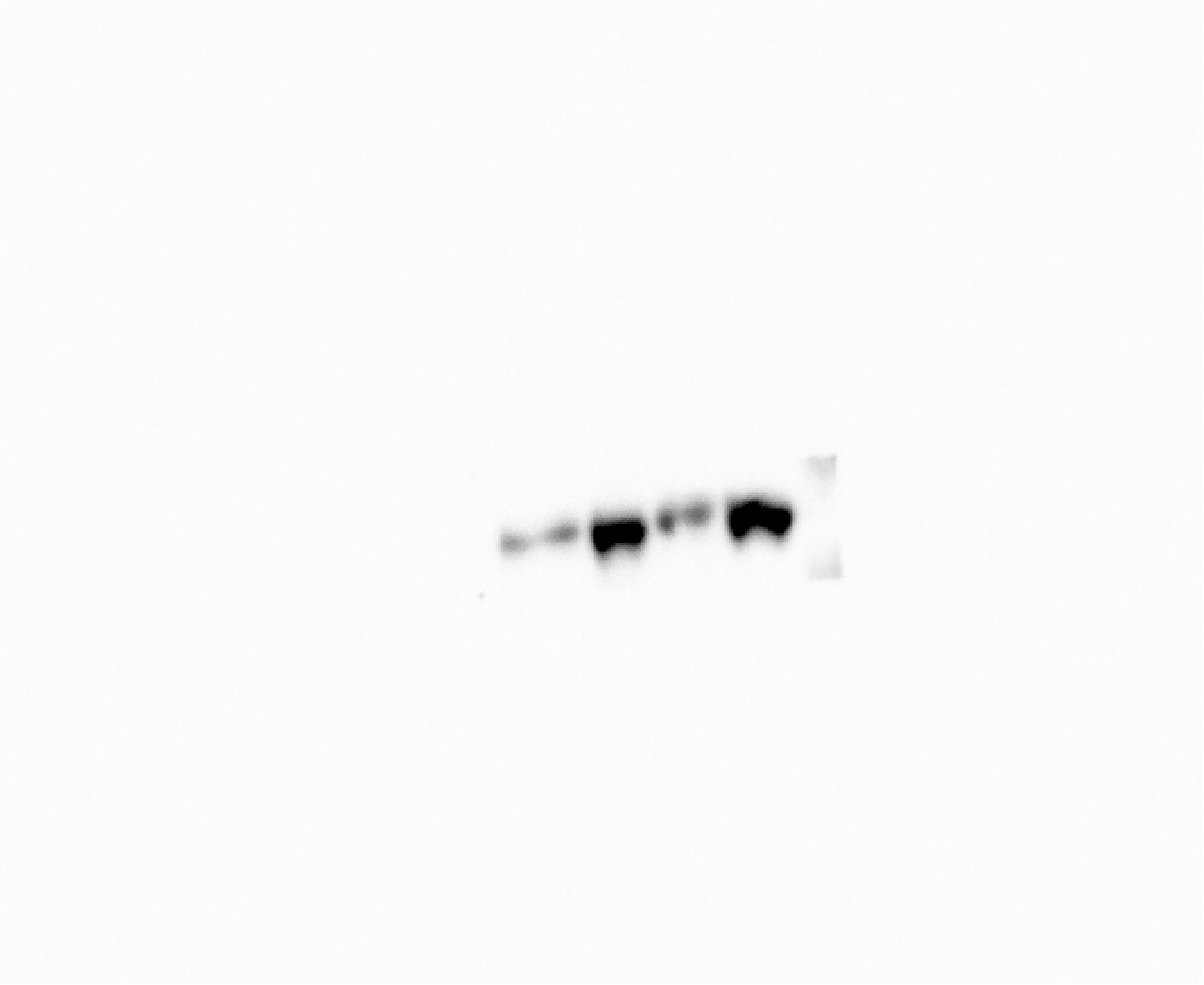

Figure 1H MTHFR

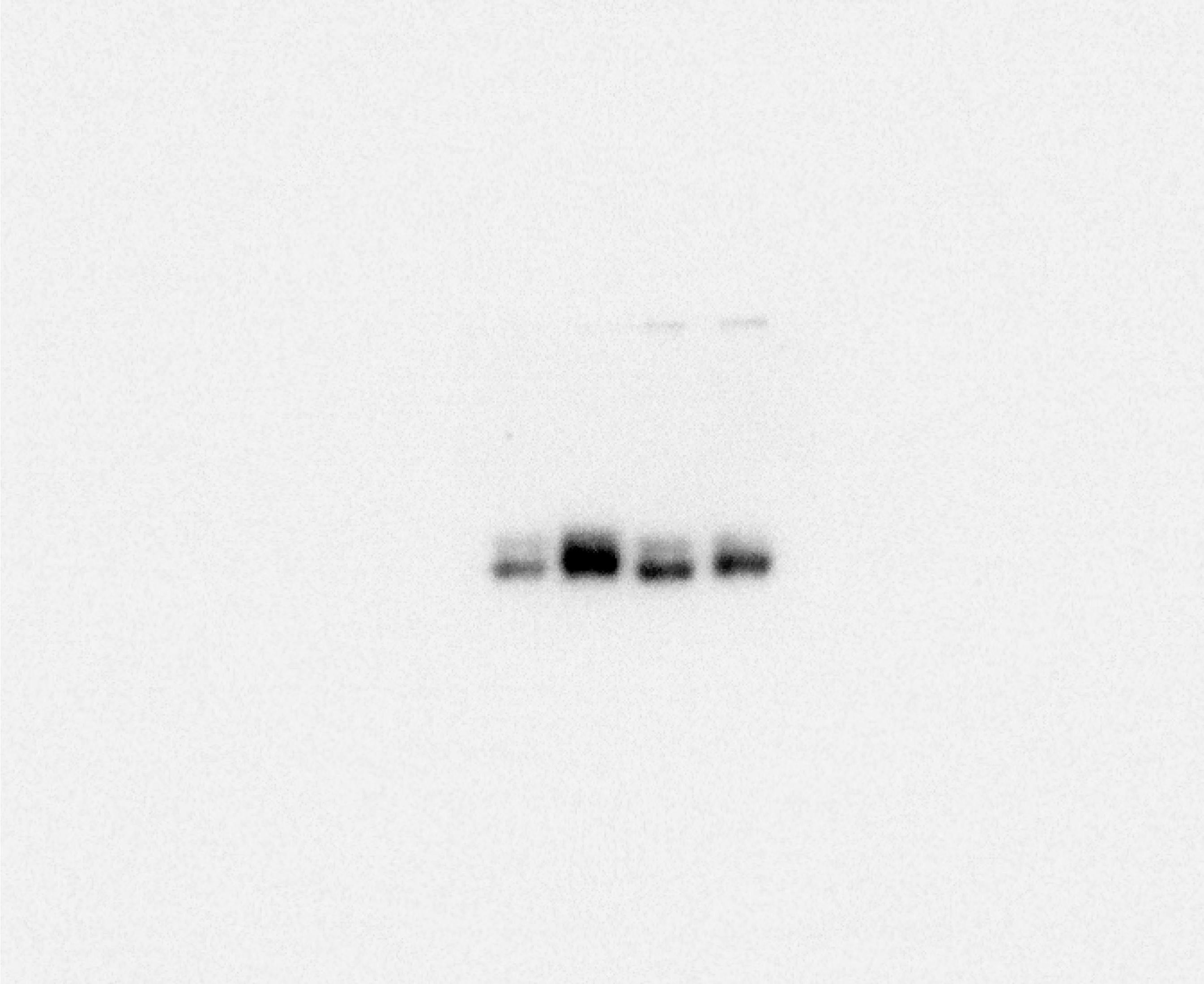

Figure 1H Nanog

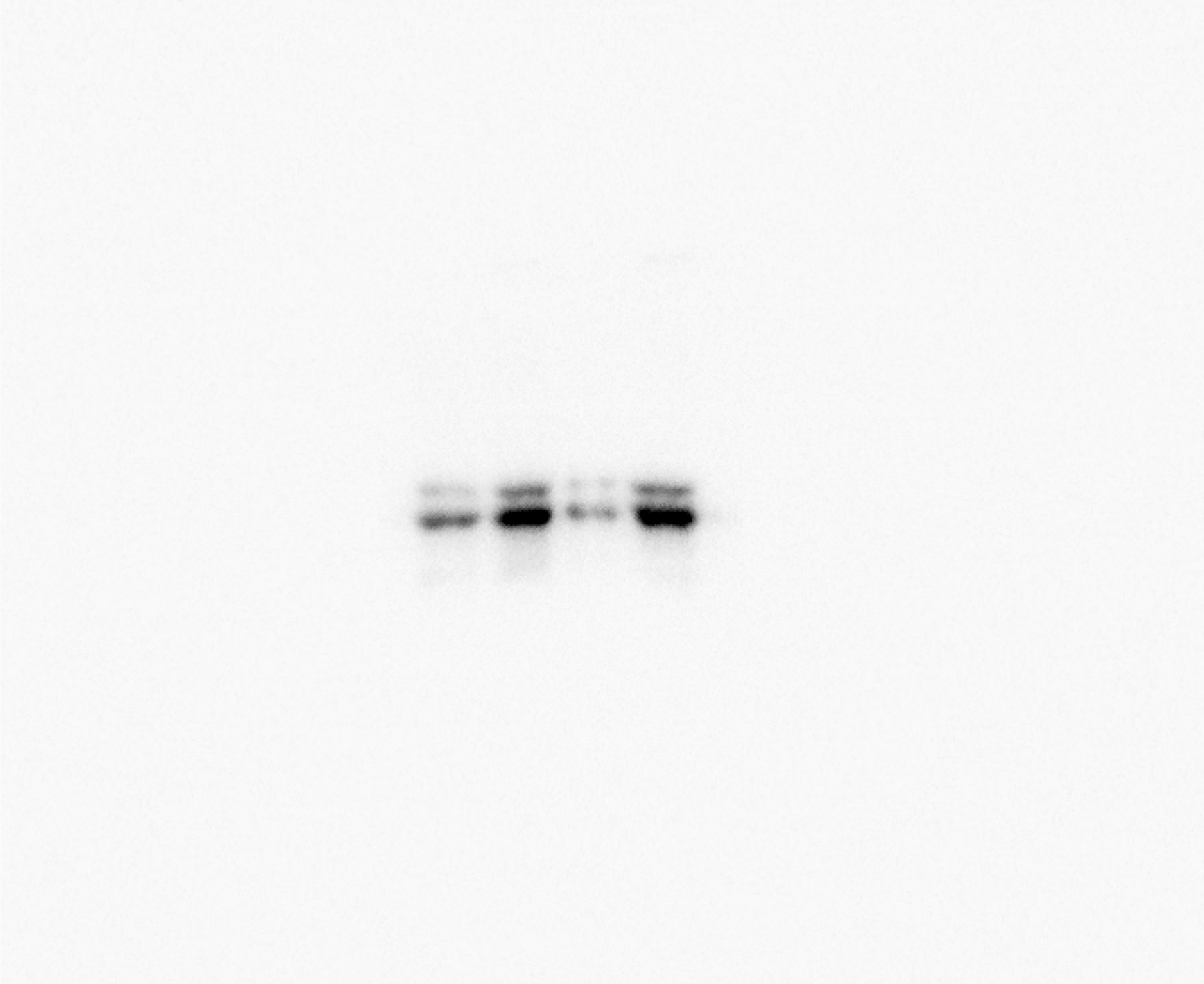

Figure 1H SAHH

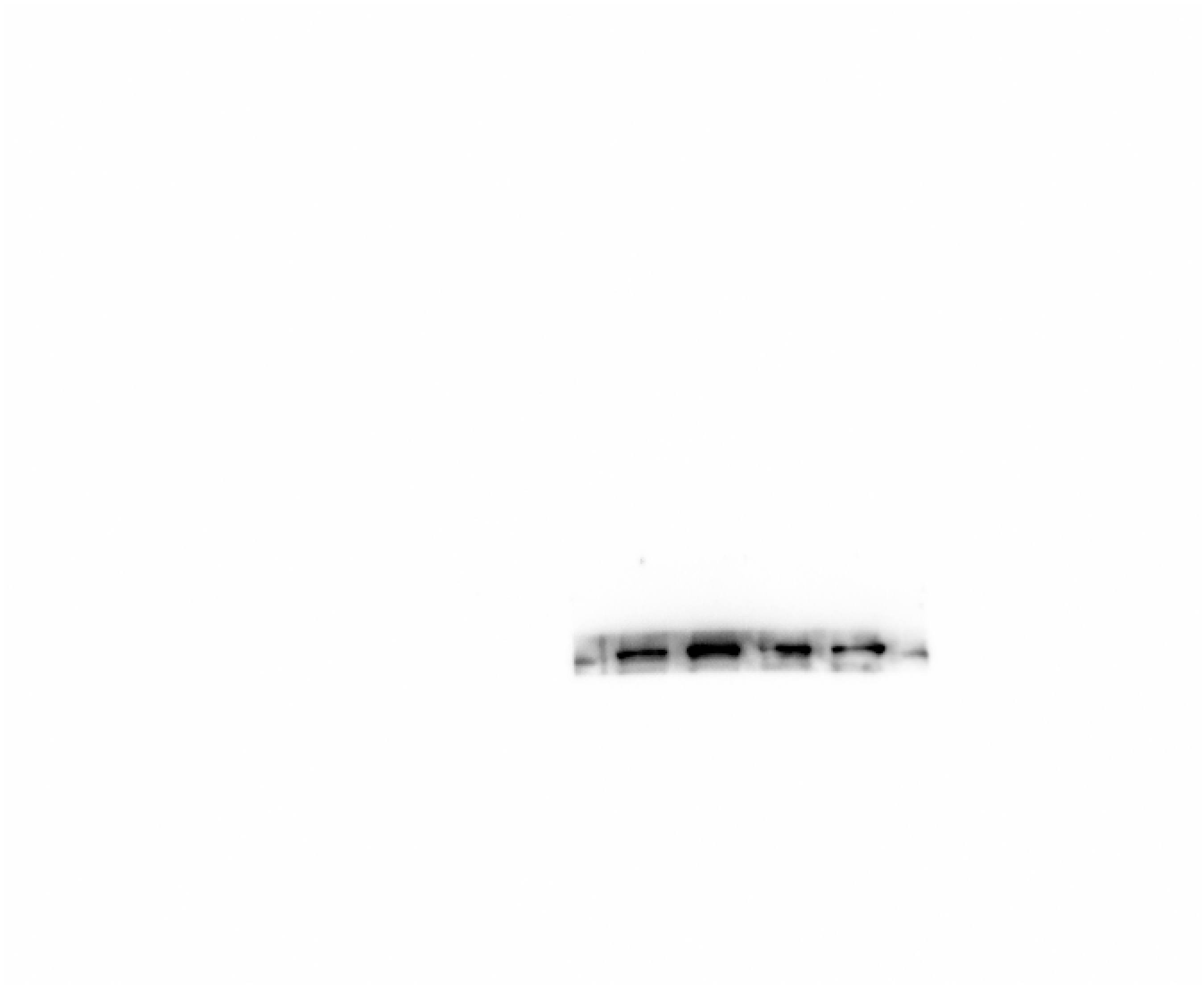

Figure 1I Total H3

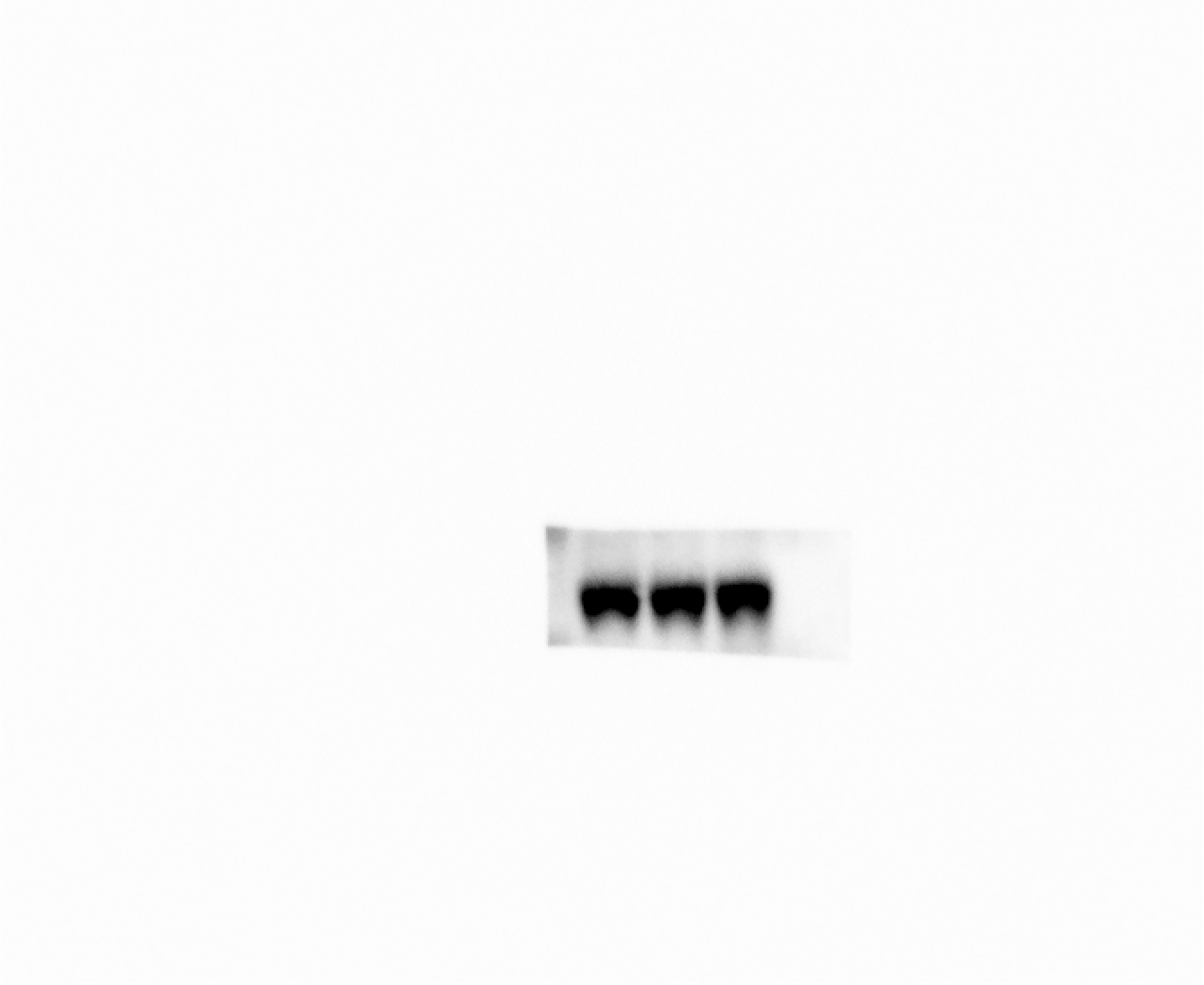

Figure 1I CD44

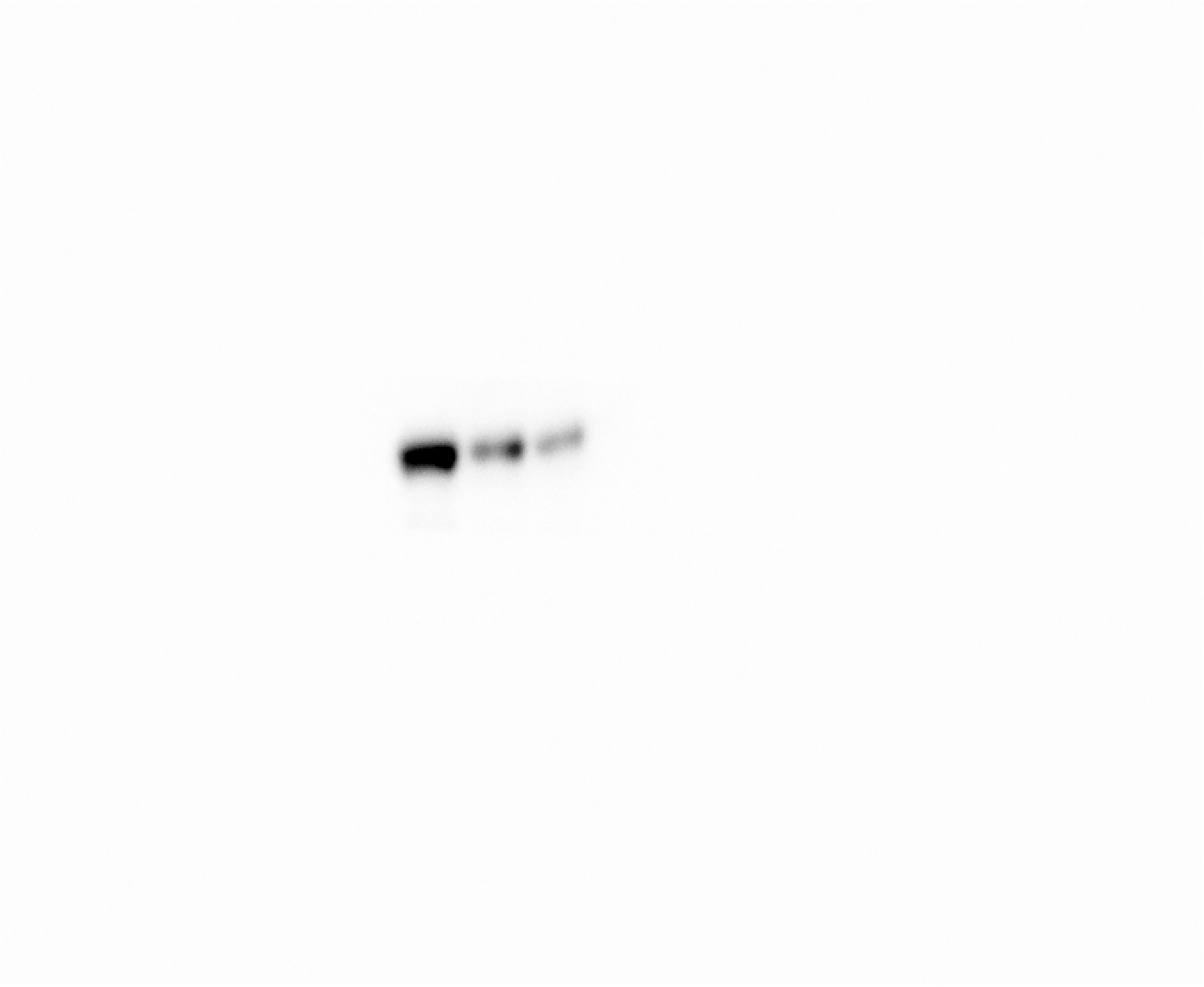

Figure 1I GAPDH

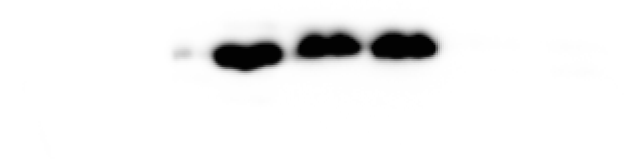

Figure 1I H3K4me3

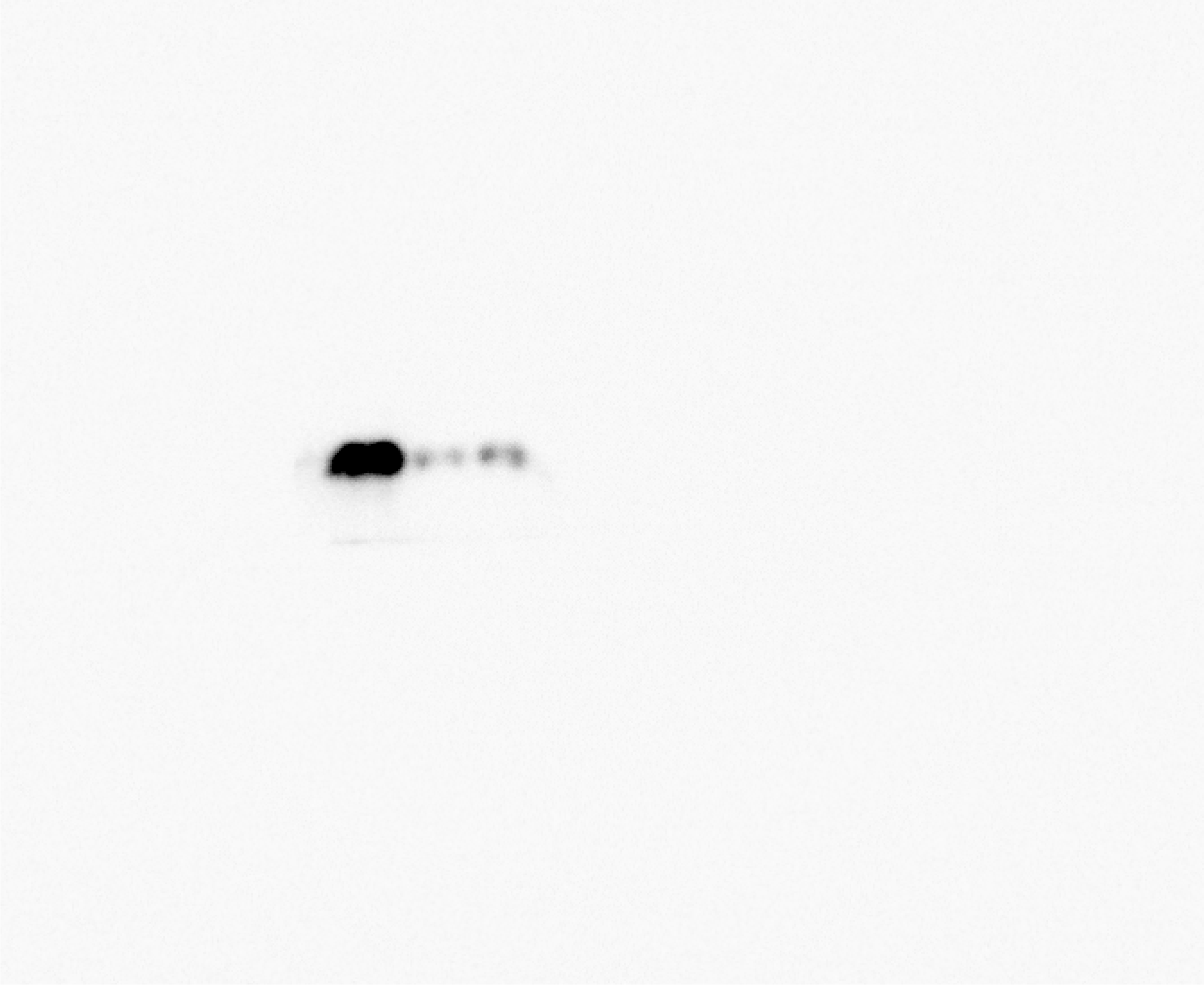

Figure 1I H3K9me3

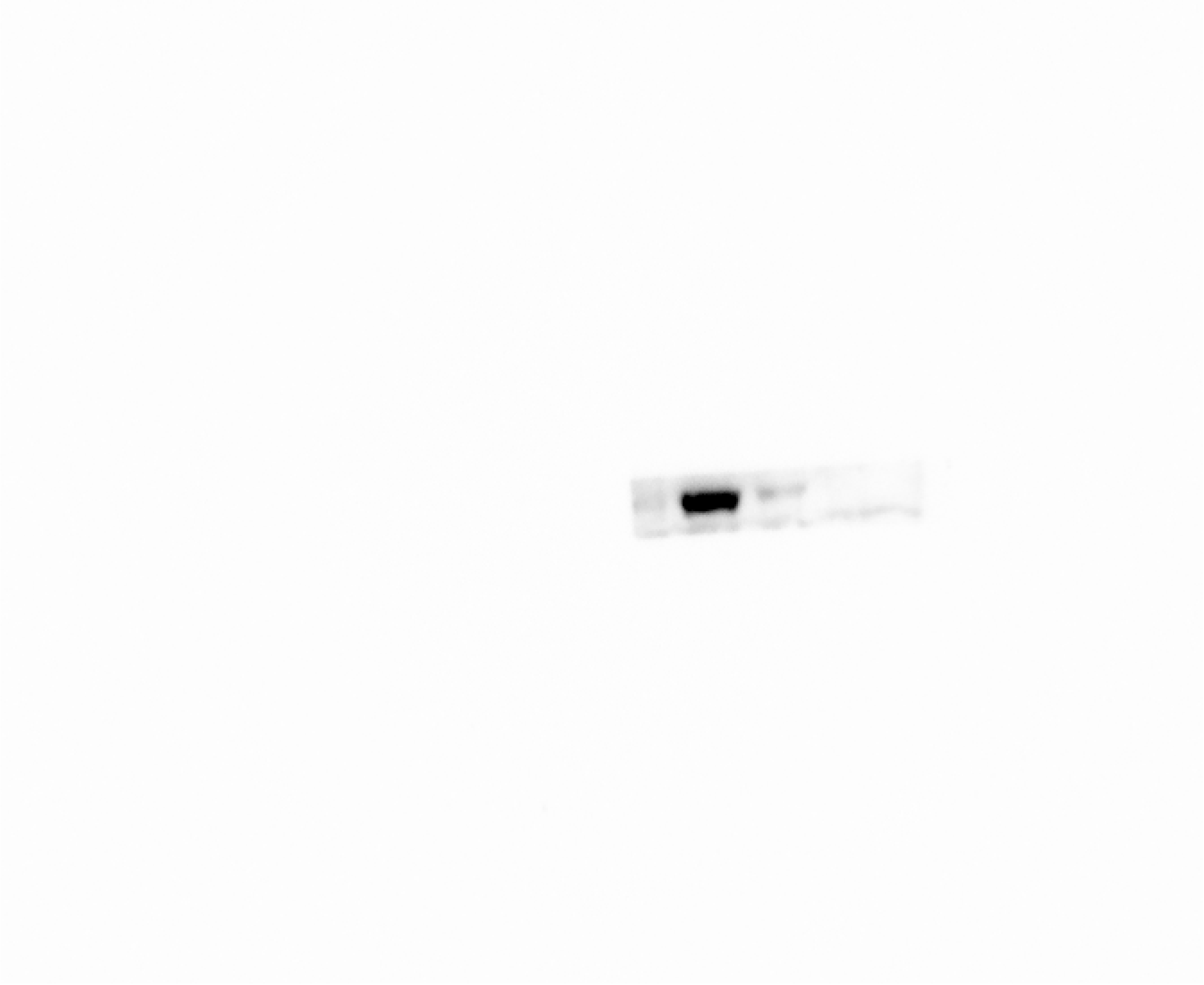

Figure 1I H3K27me3

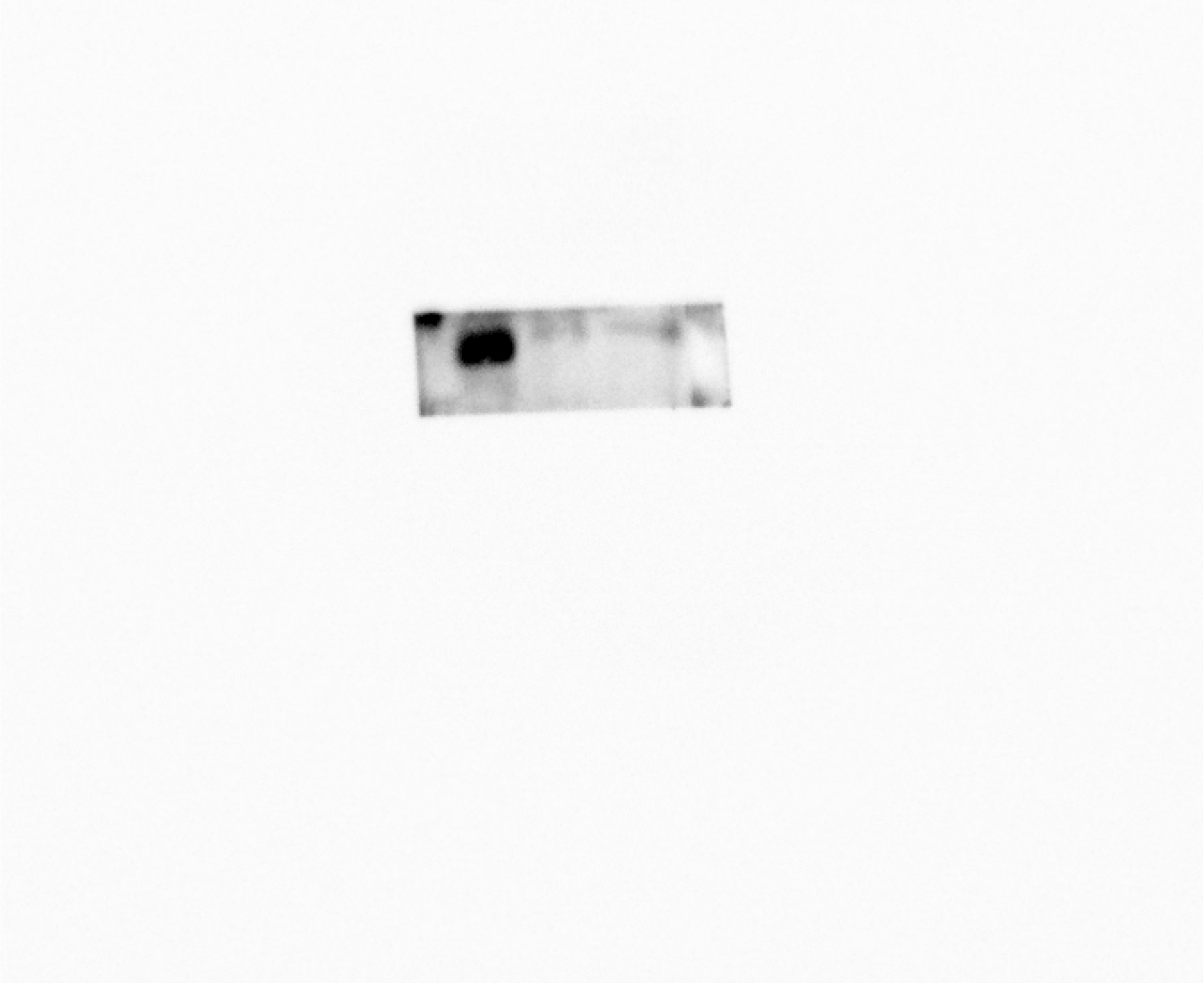

Figure 1I H3K36me3

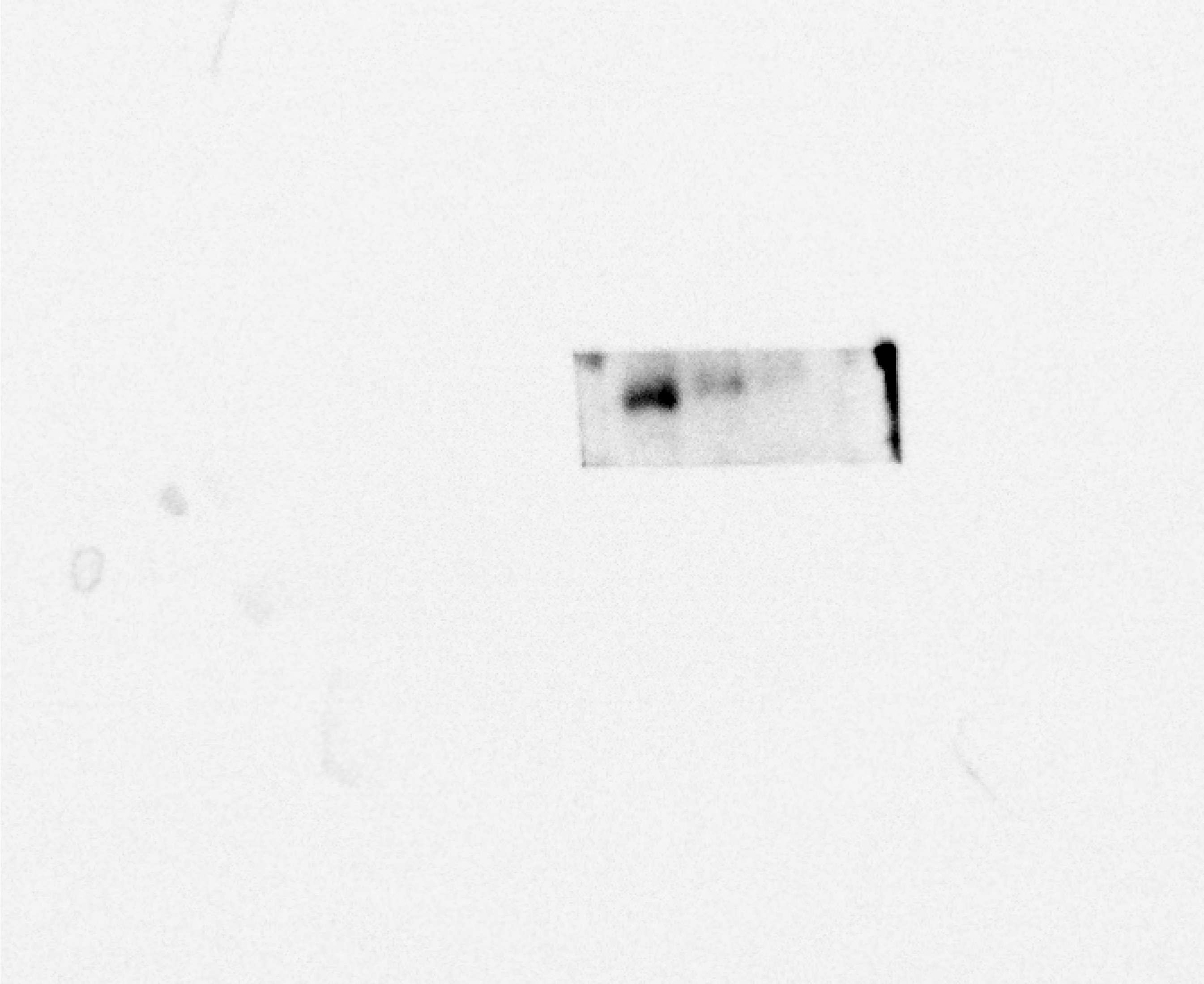

Figure 1I MAT2A

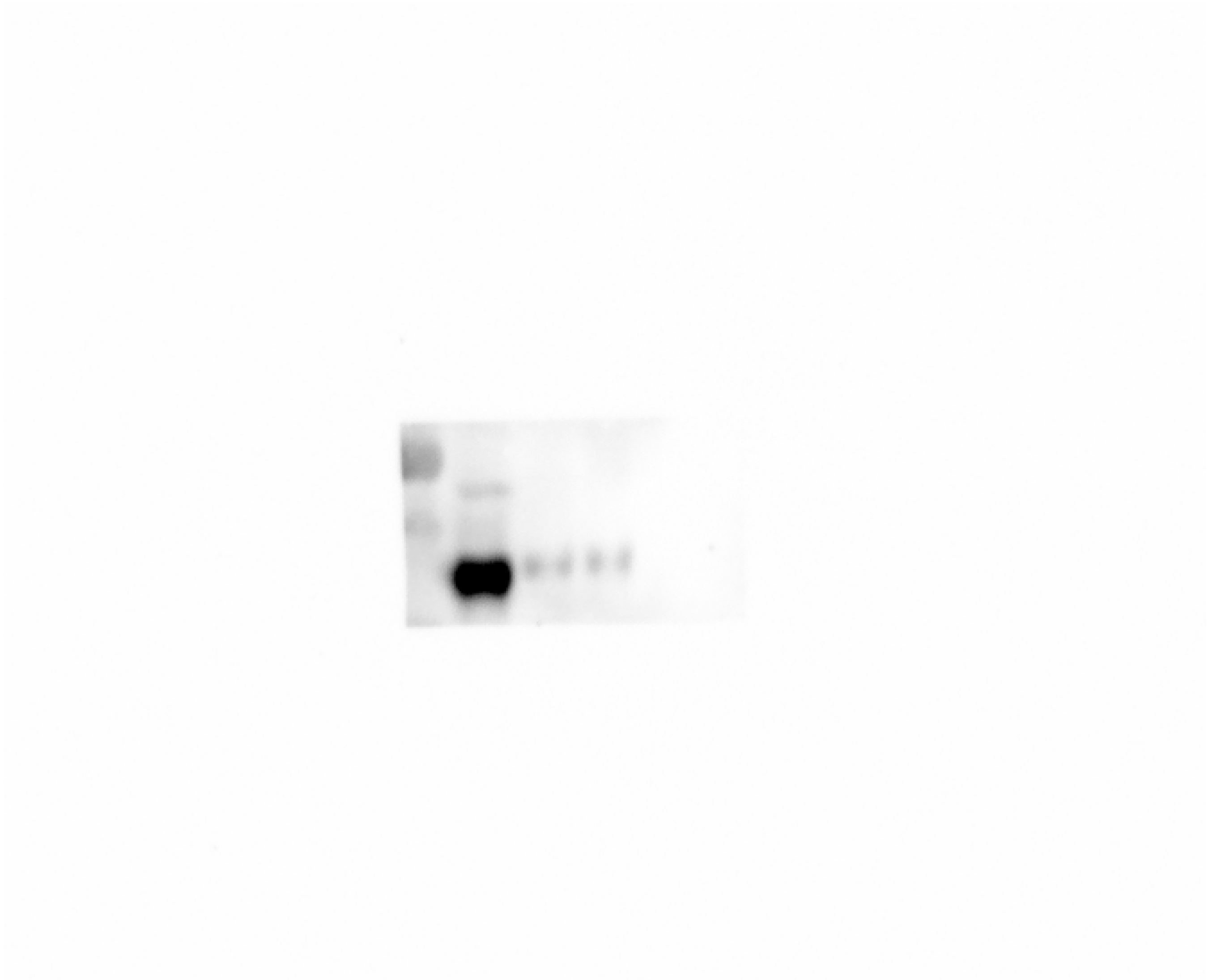

Figure 1I Nanog

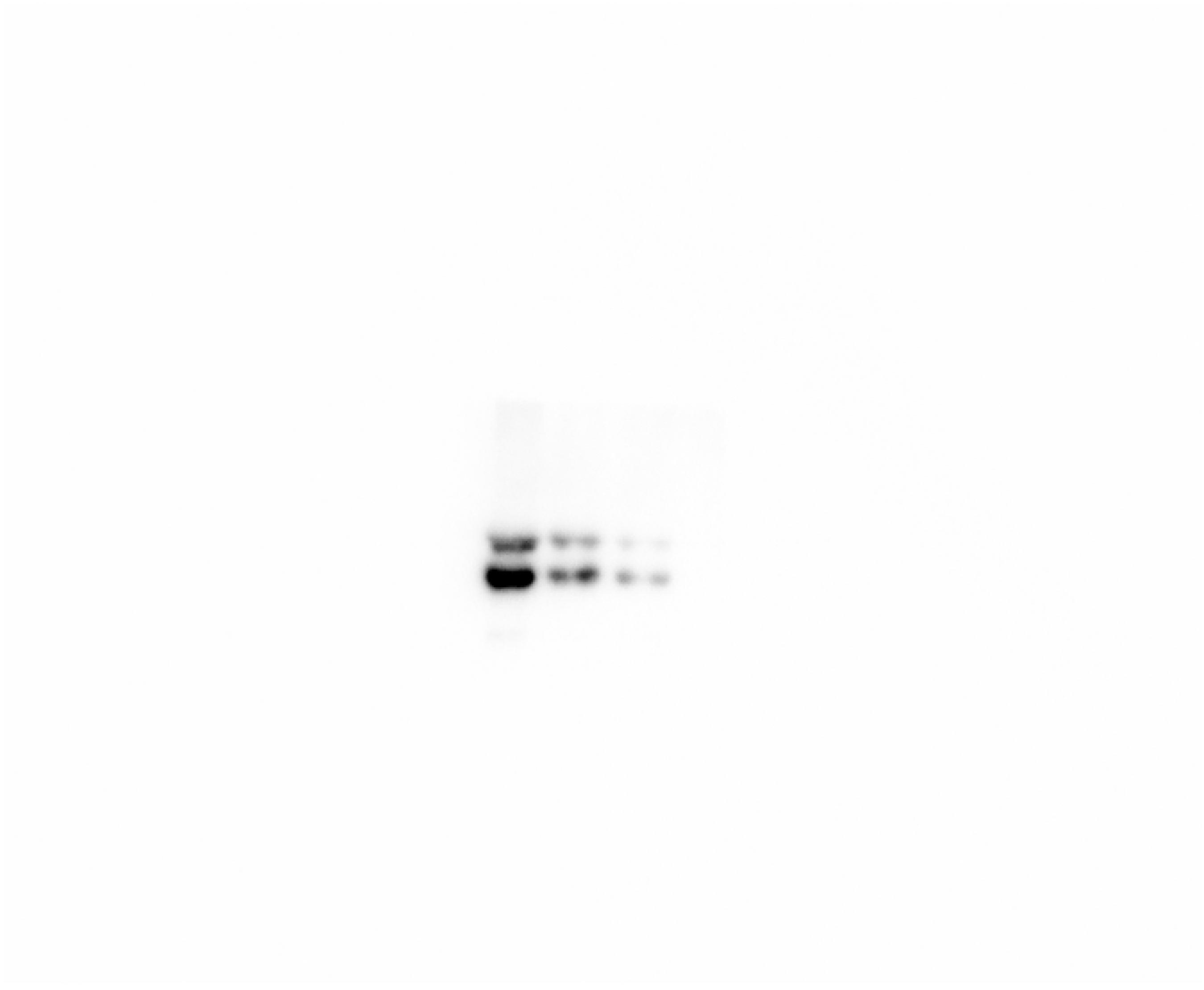

Figure 2C MAT2A

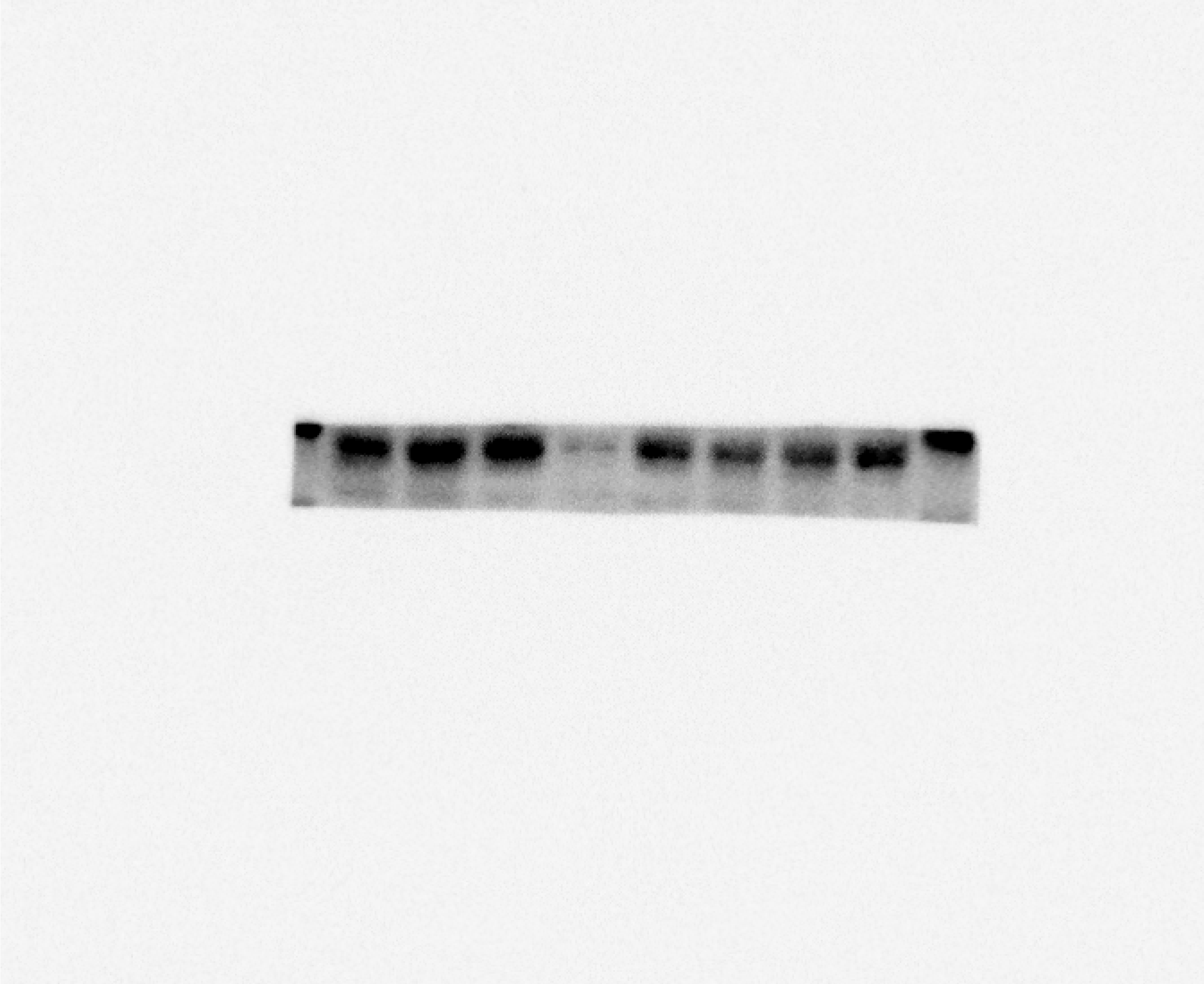

Figure 2C GAPDH

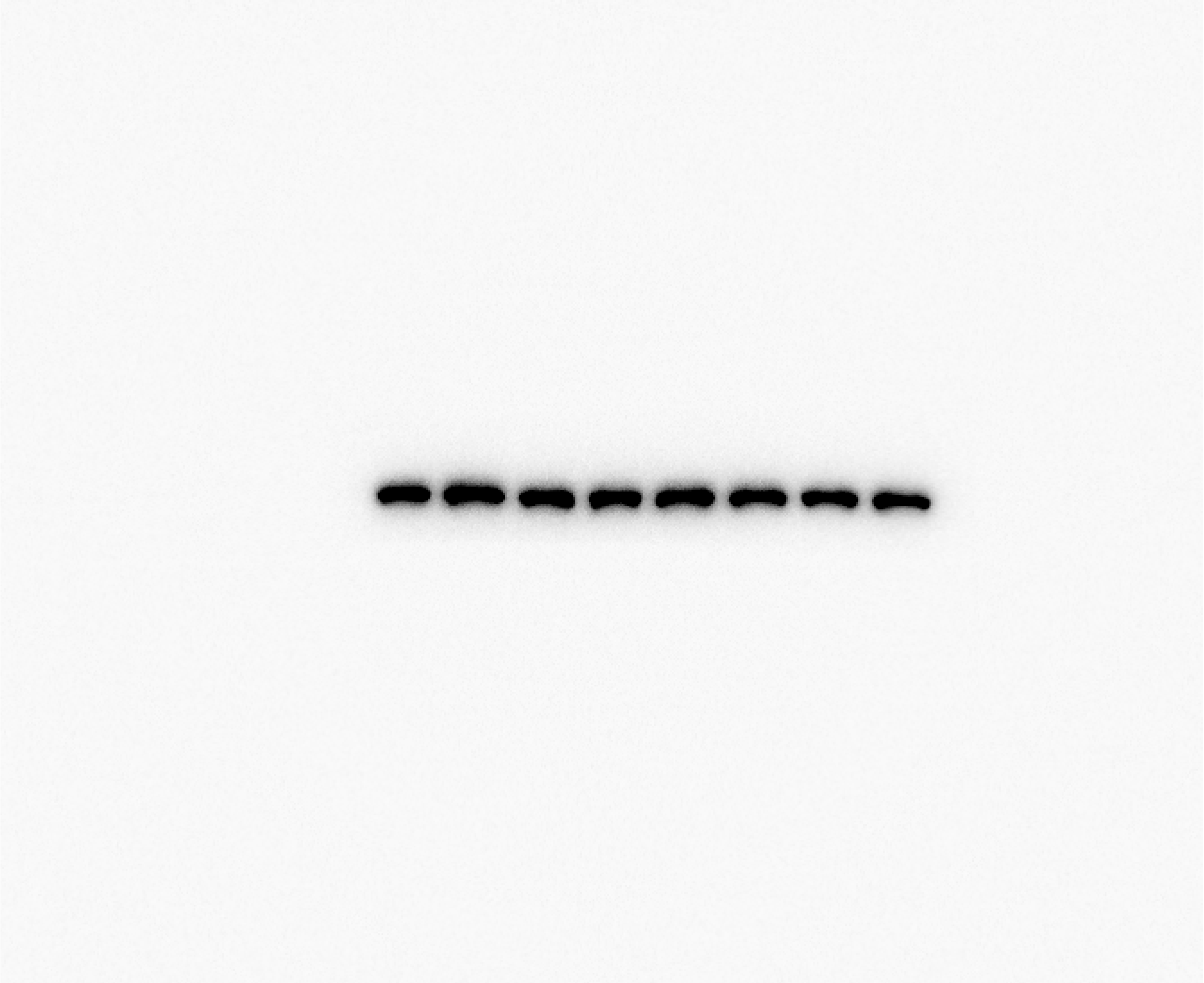

Figure 2J H3K36me3

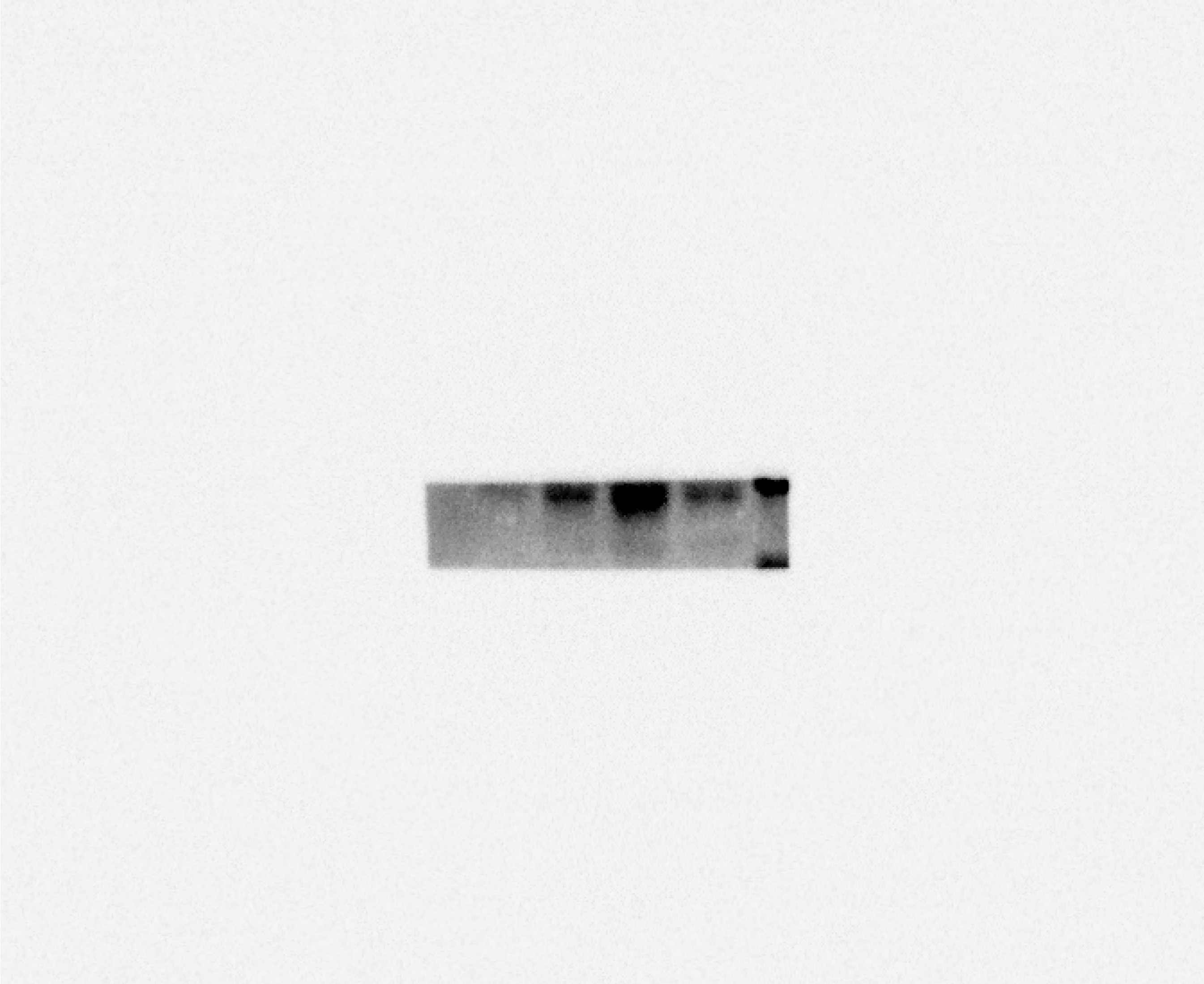

Figure 2J MAT2A

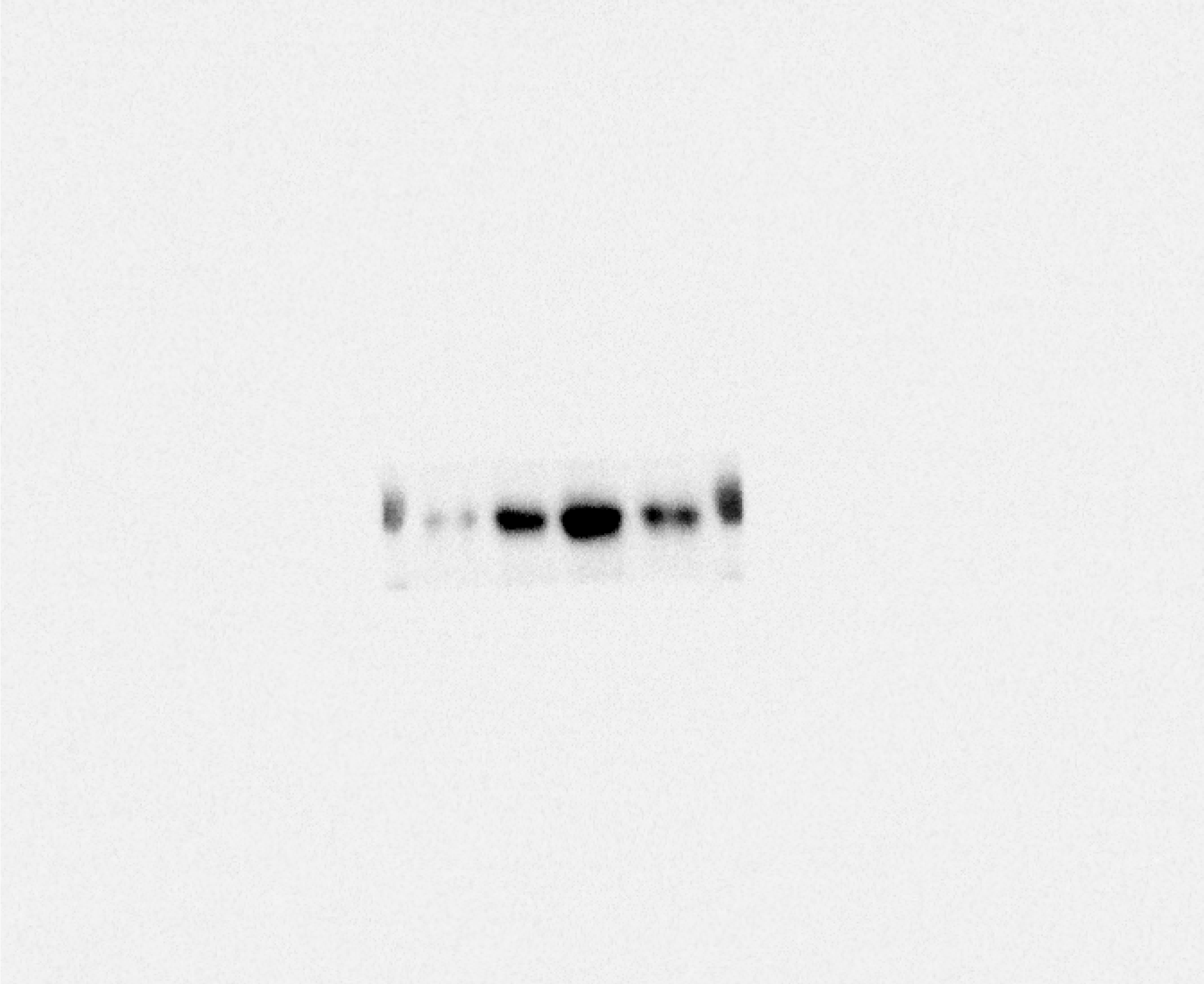

Figure 2J Nanog

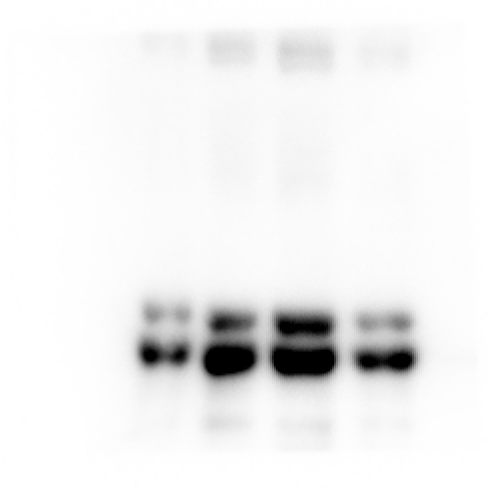

Figure 2J Total H3

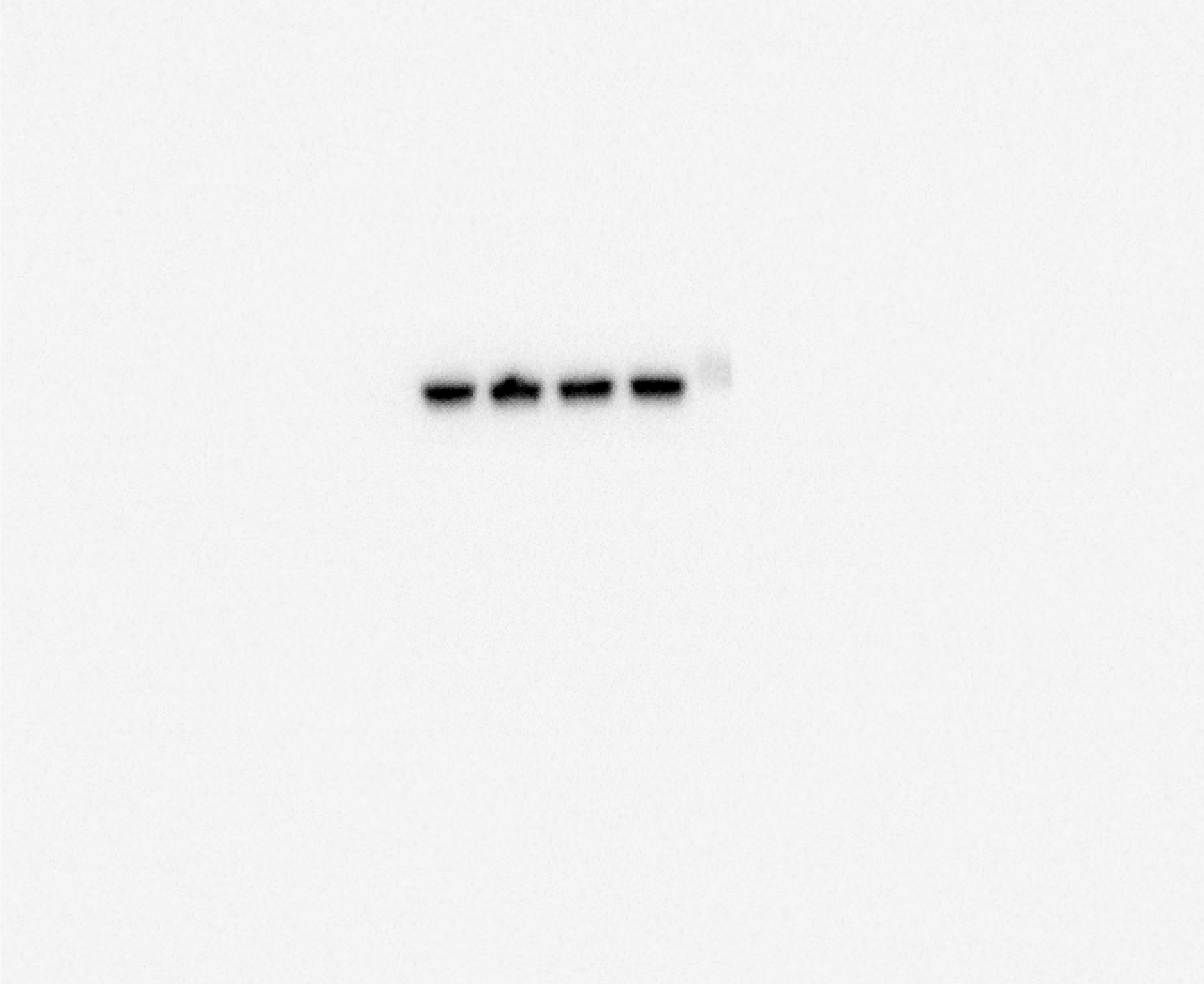

Figure 2J CD44

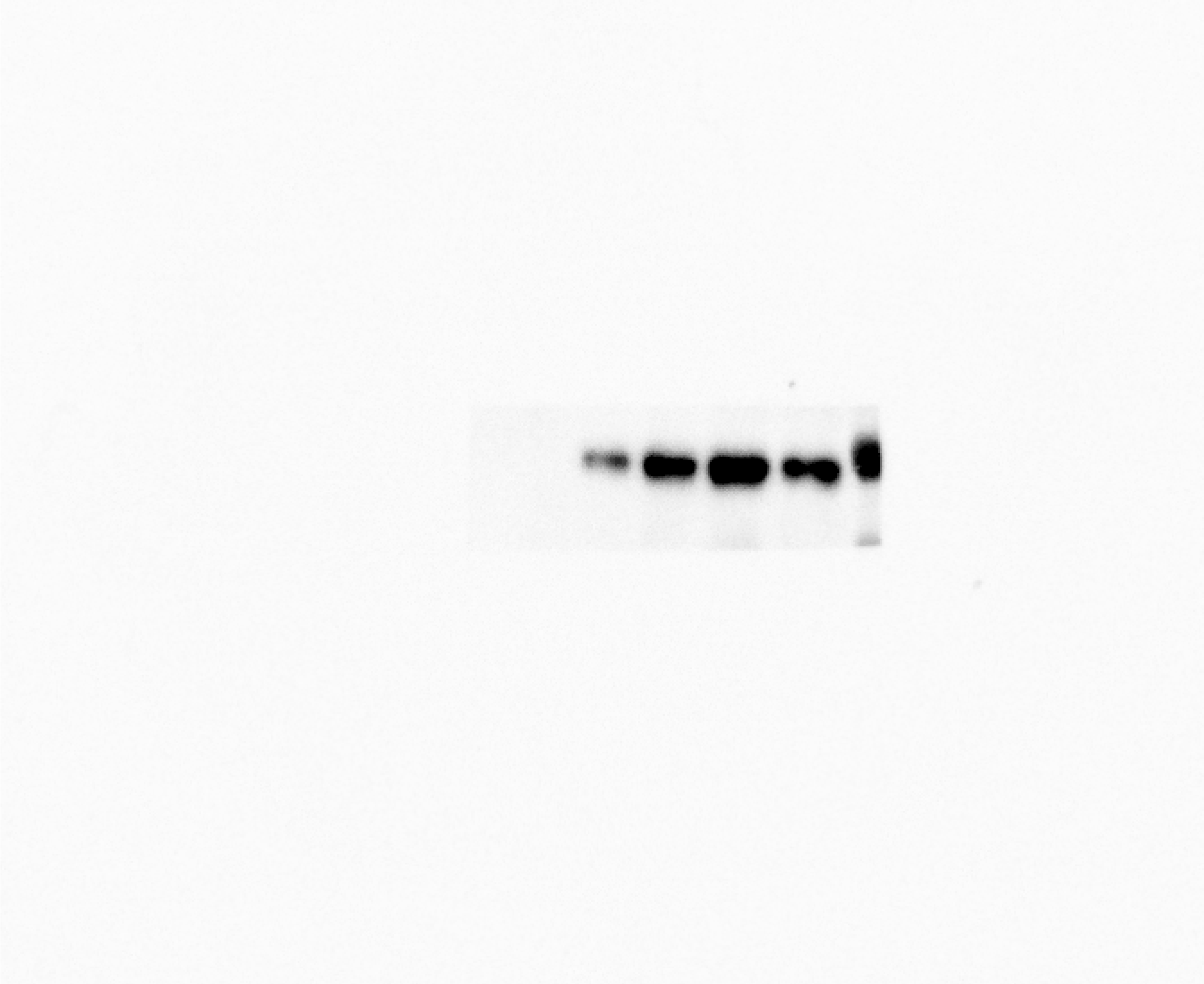

Figure 2J GAPDH

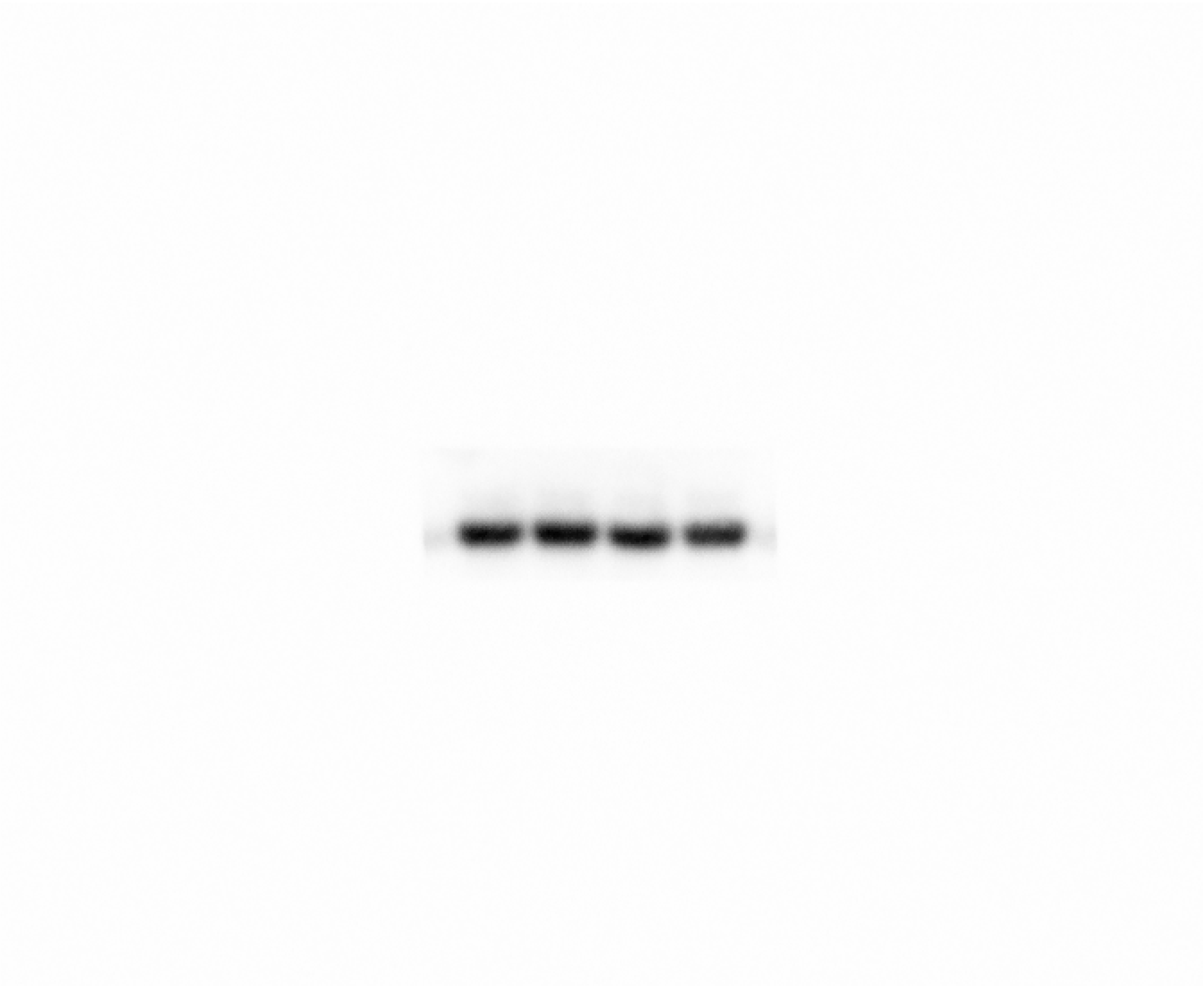

Figure 2J H3K4me3

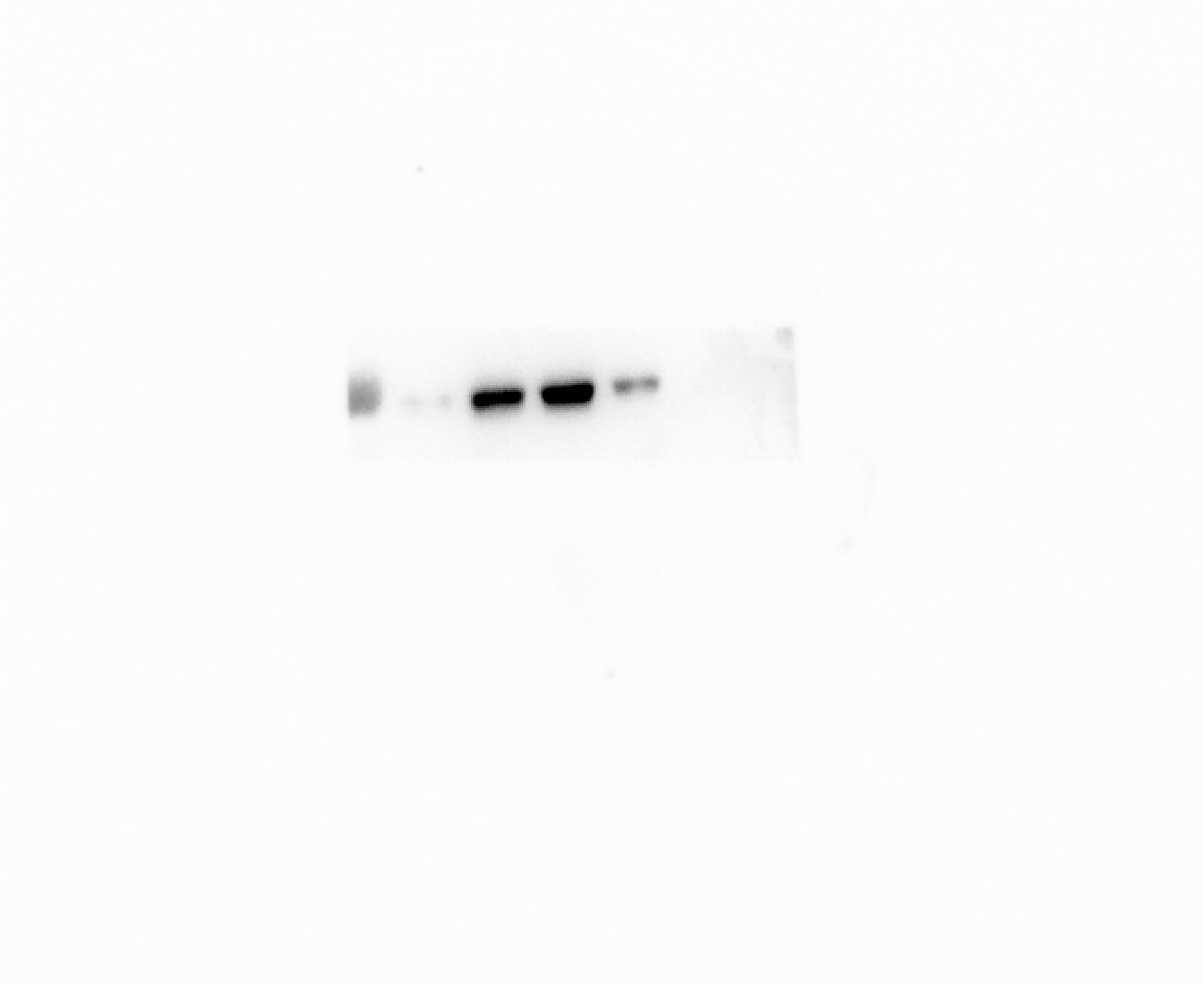

Fgure 2J H3K9me3

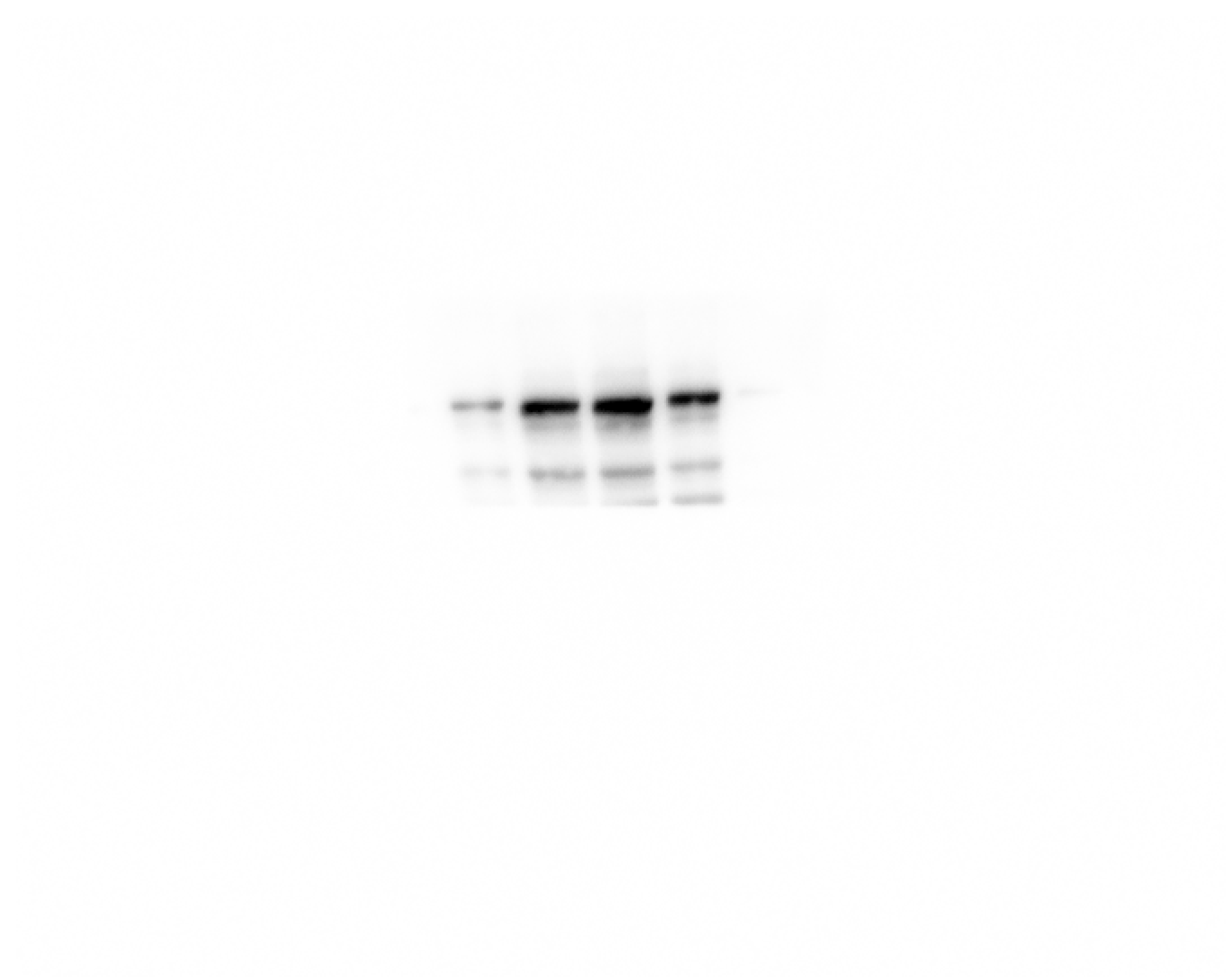

Figure 2J H3K27me3

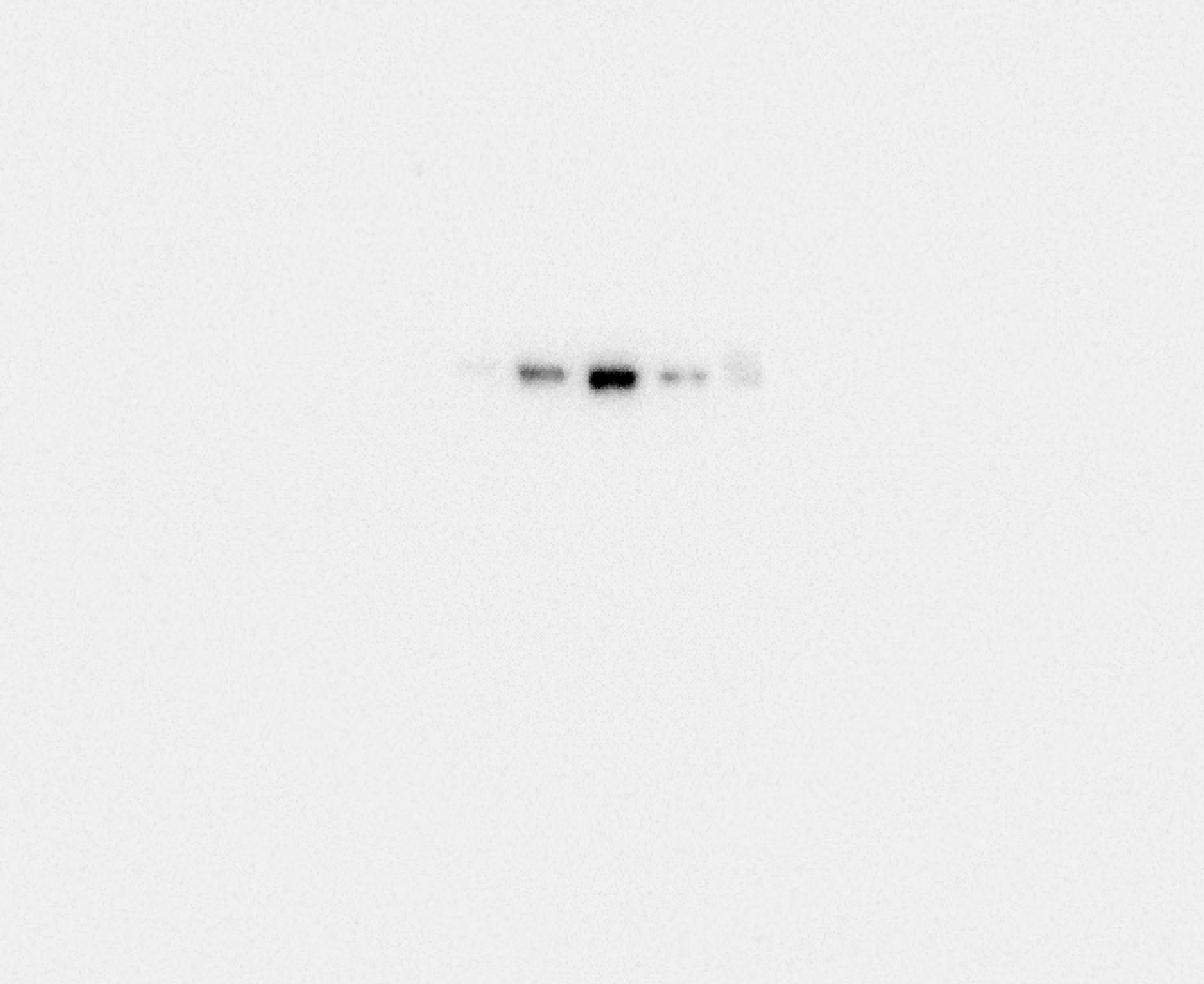

Figure 3G MAT2A UMUC-3

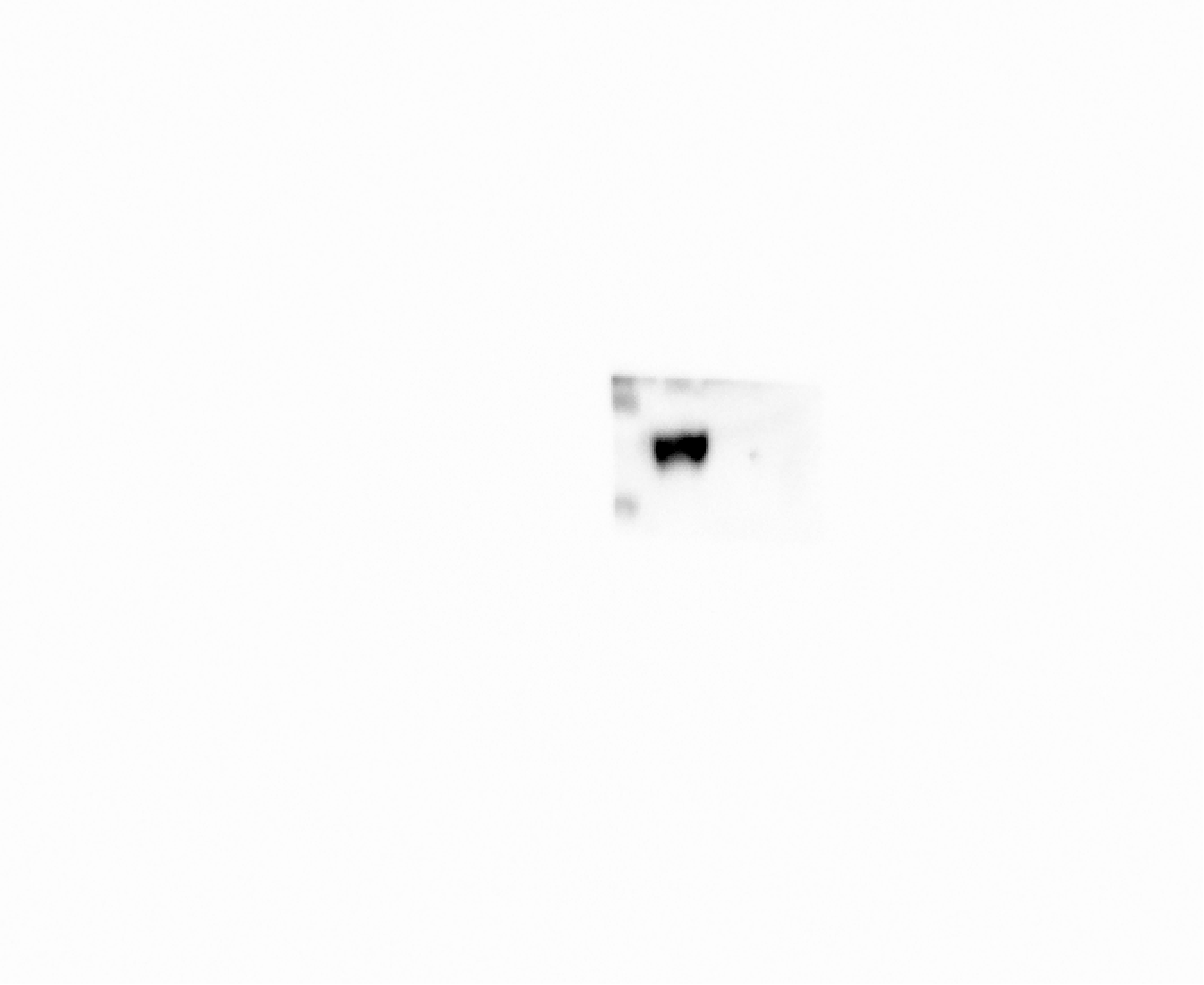

Figure 3G actin T24

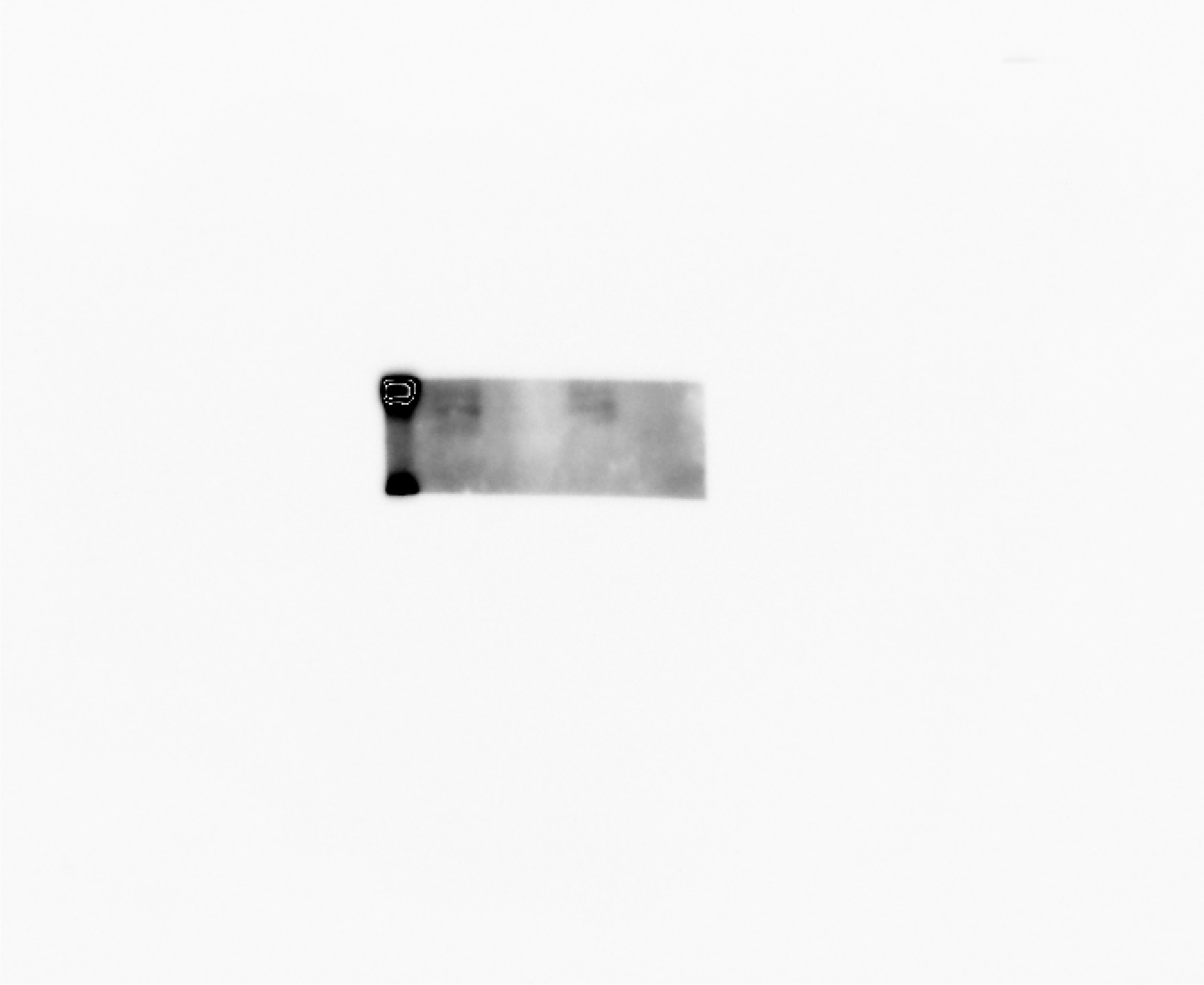

Figure 3G actin UMUC-3

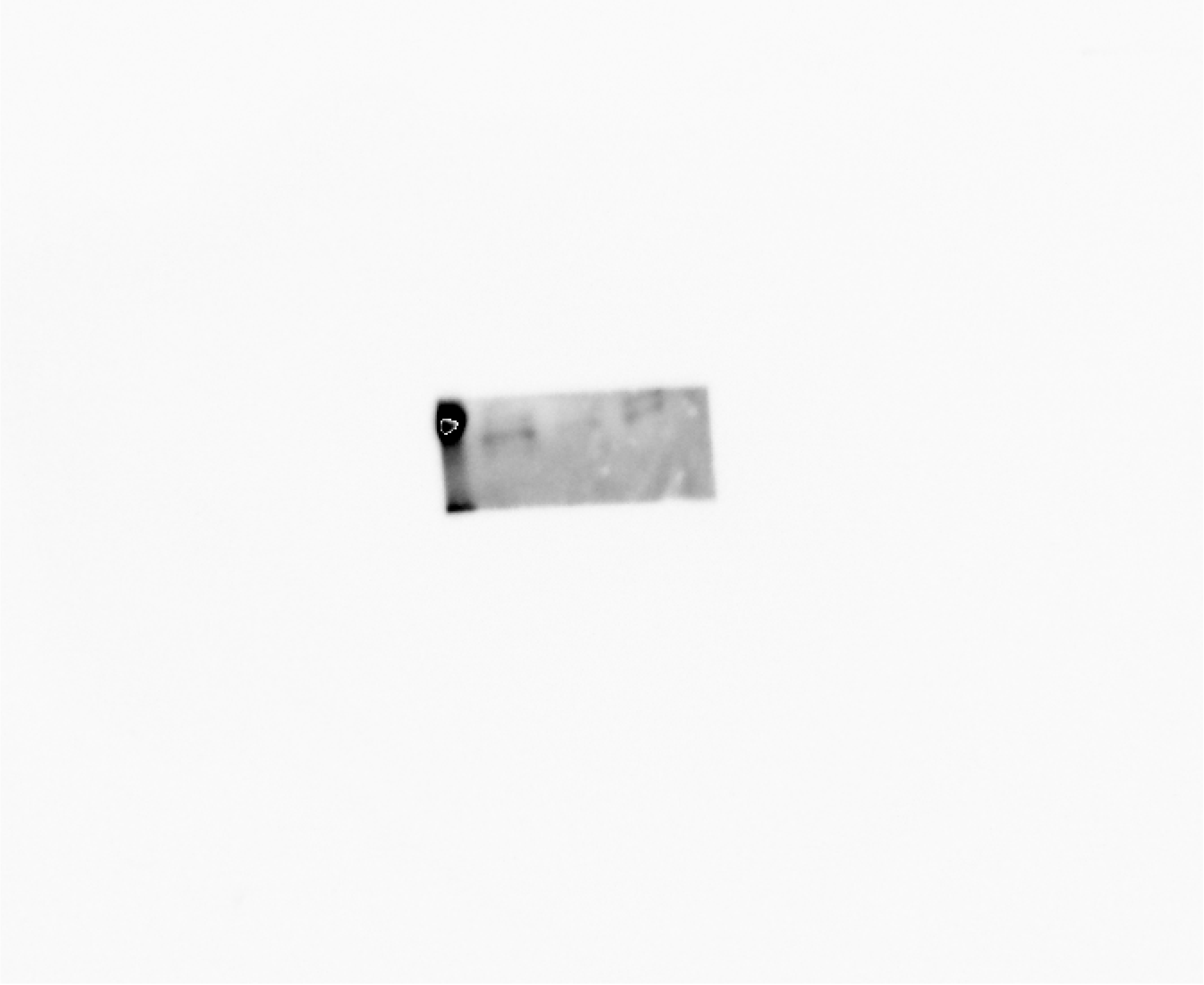

Figure 3A Ago2

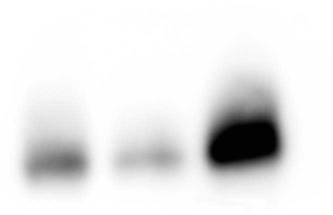

Figure 3B Ago2

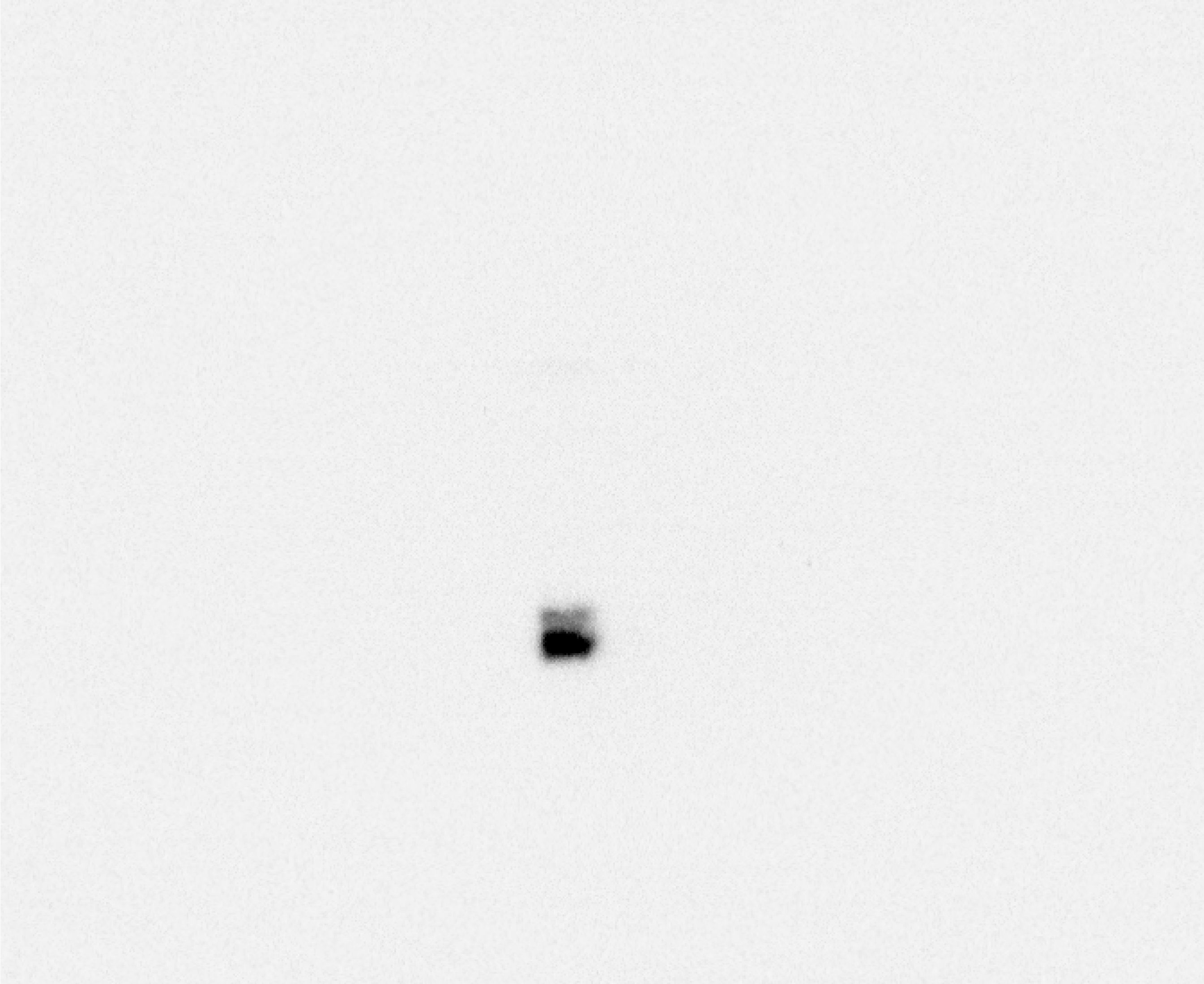

Figure 3G MAT2A T24

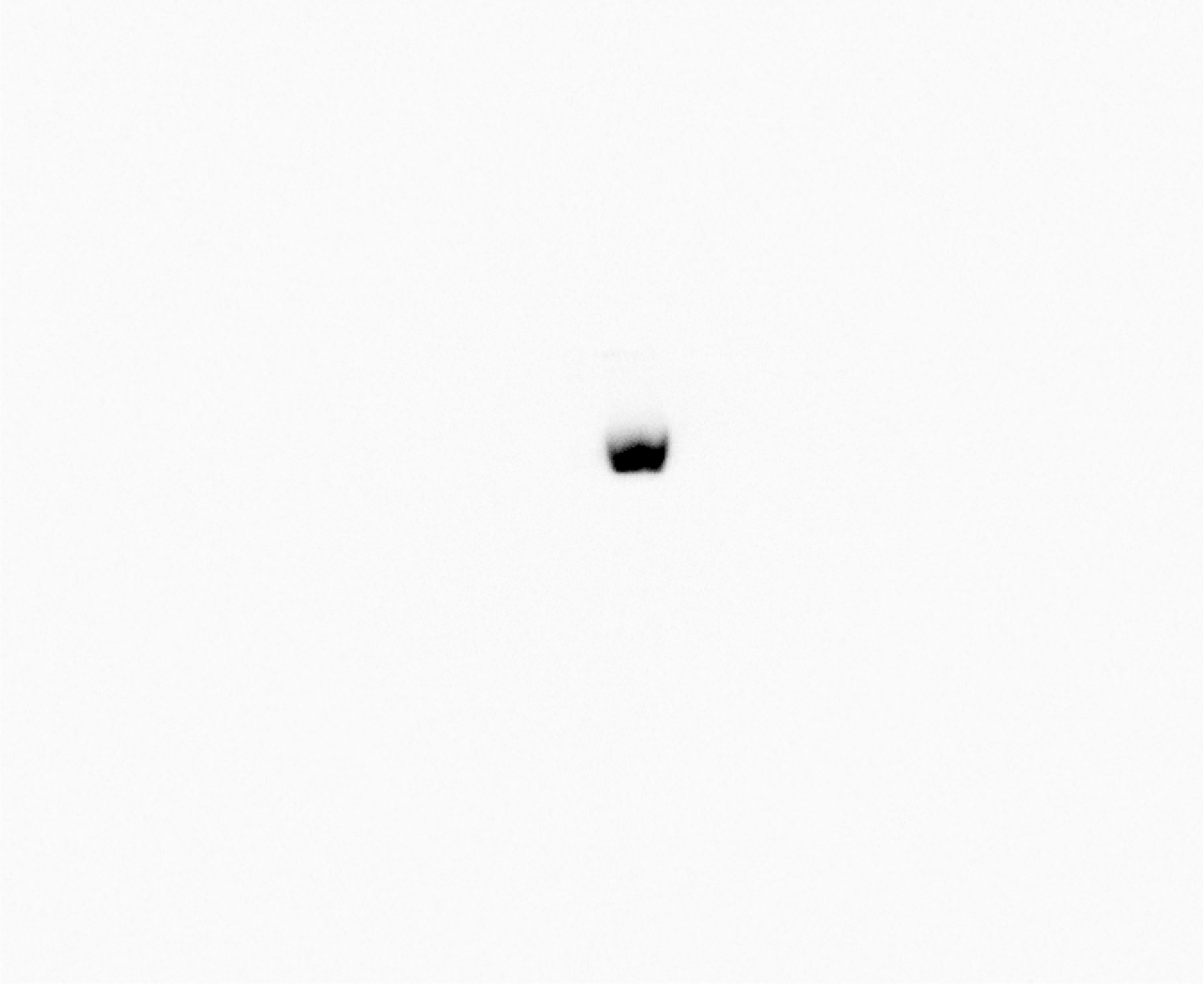

Figure 3H MAT2A UMUC-3

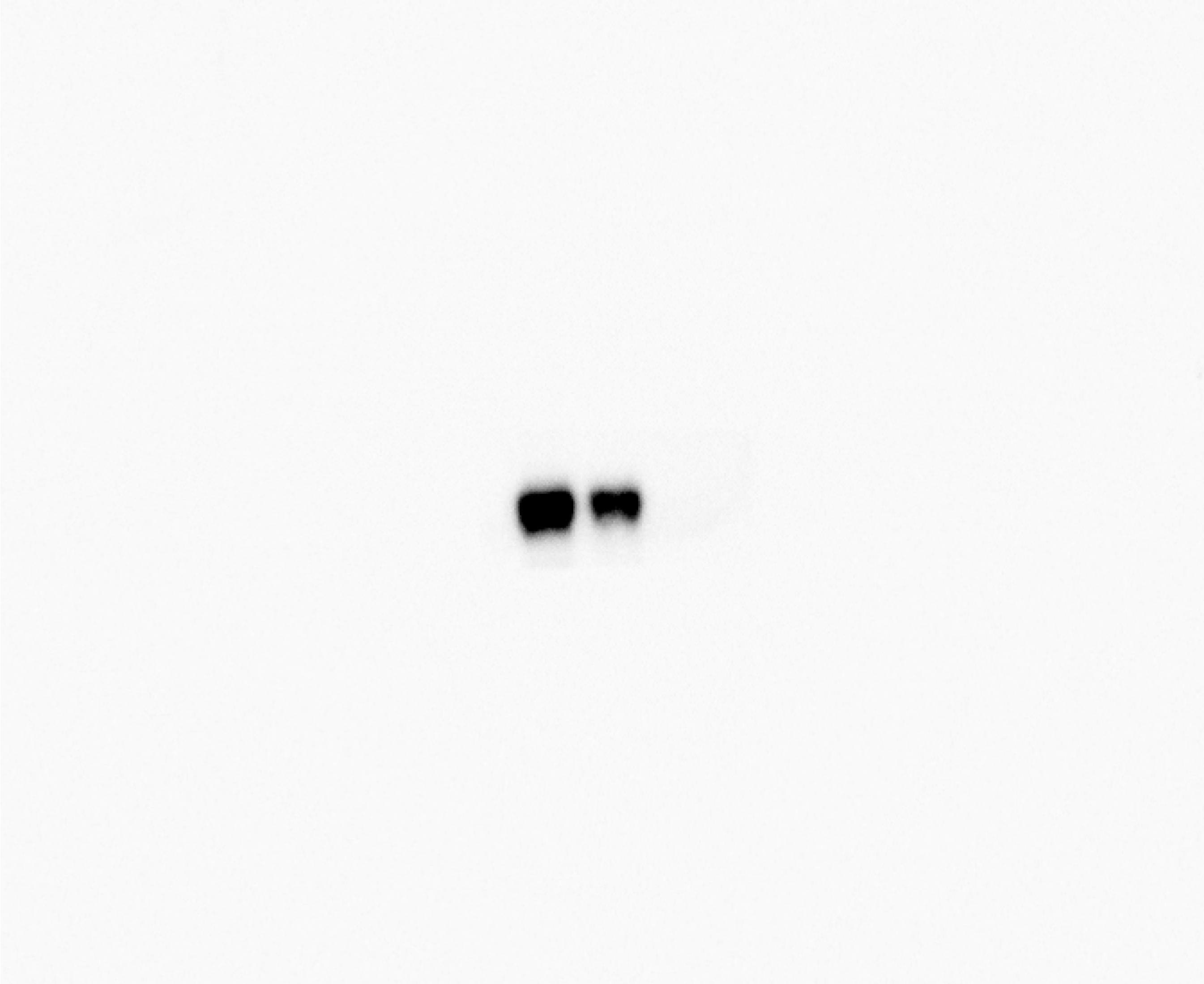

Figure 3H GAPDH T24

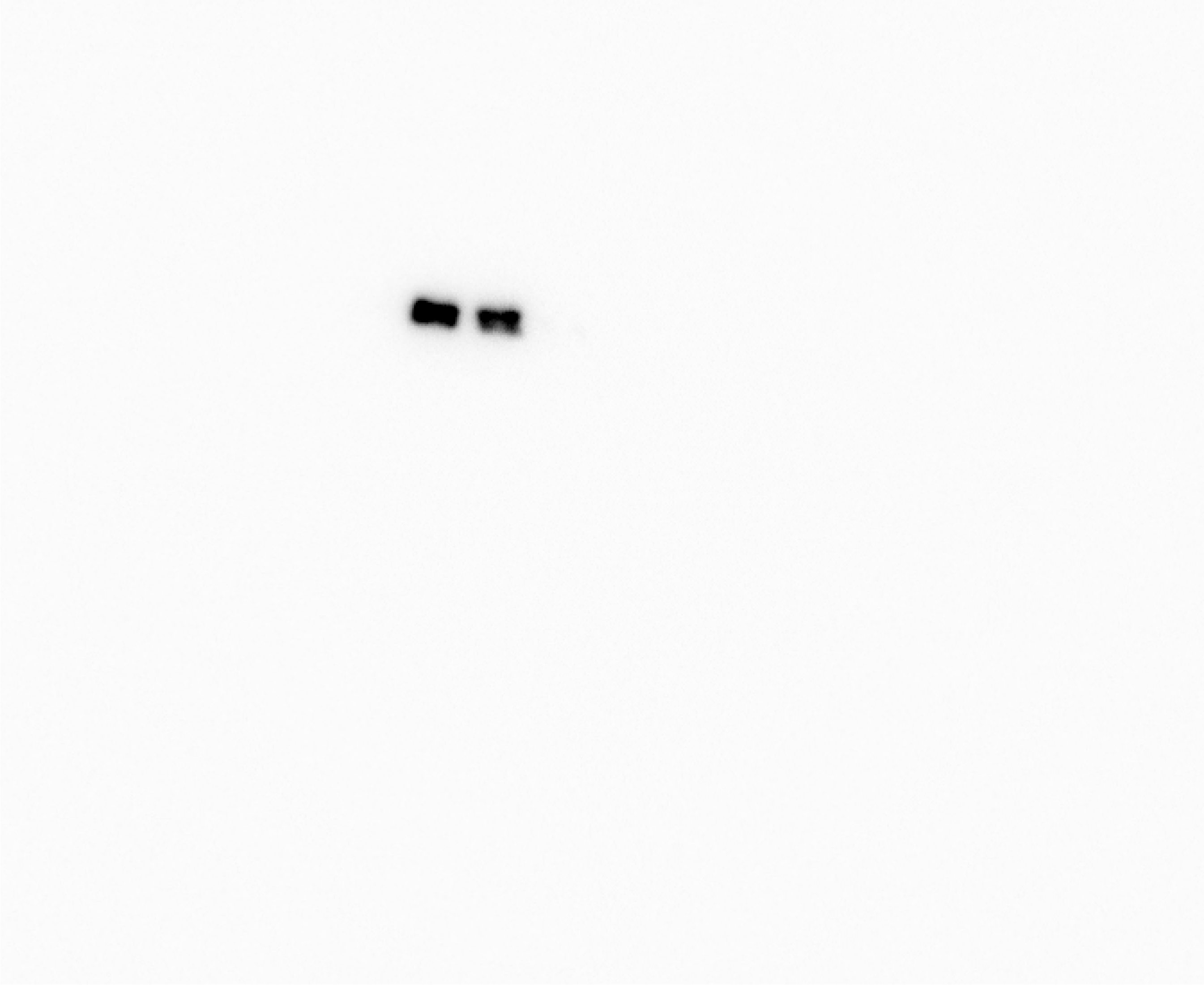

Figure 3H GAPDH UMUC-3

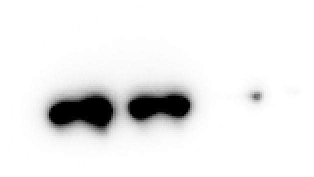

Figure 3H MAT2A T24

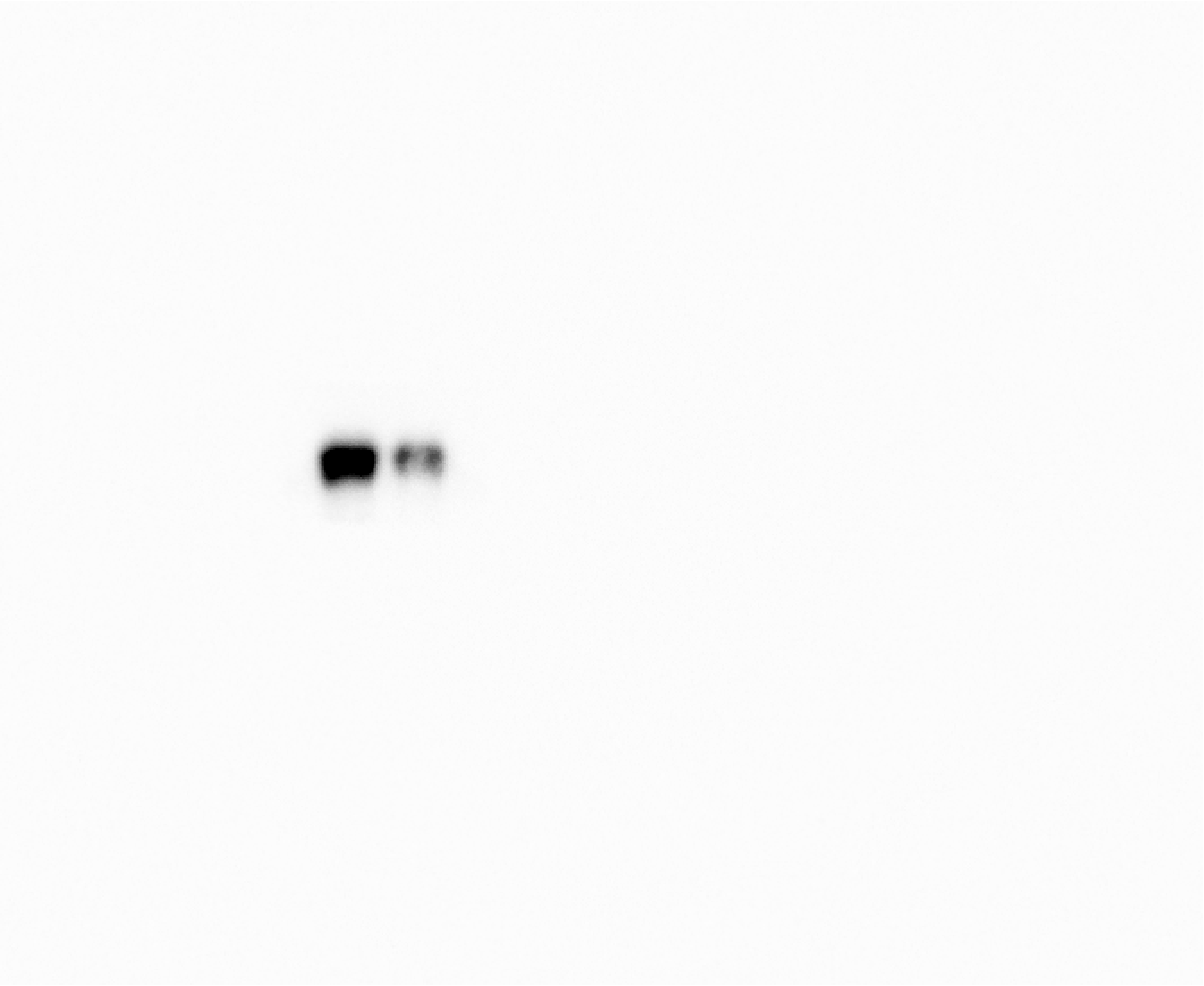

Figure 3I umuc-3 MAT2A

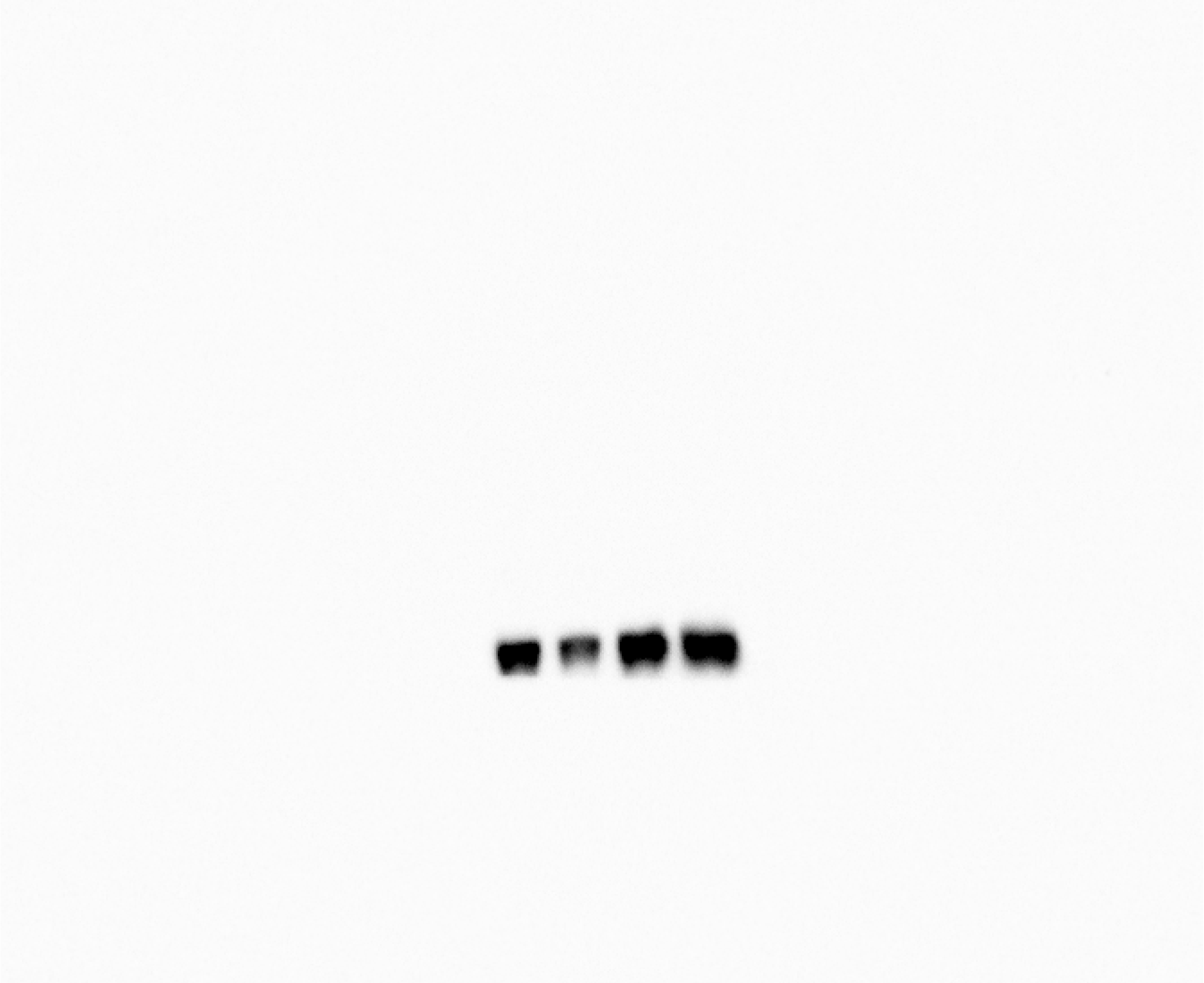

Figure 3I T24 GAPDH

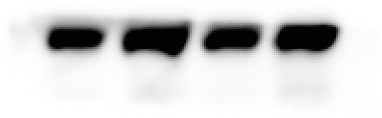

Figure 3I T24 MAT2A

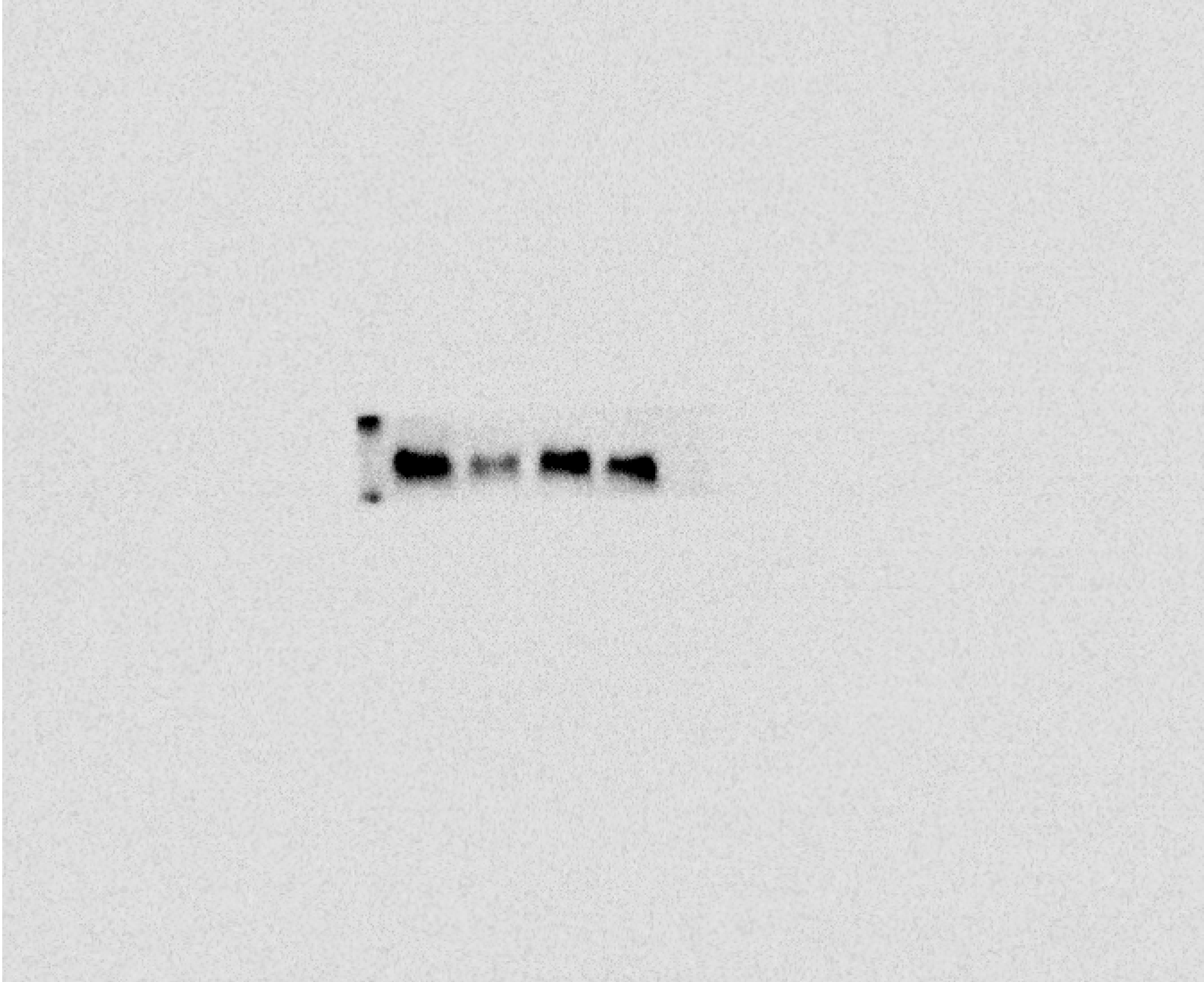

Figure 3I umuc-3 GAPDH

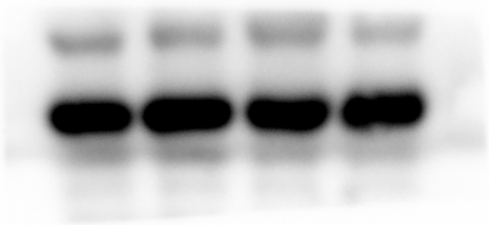

Figure 3J LV-NC MAT2A UMUC-3

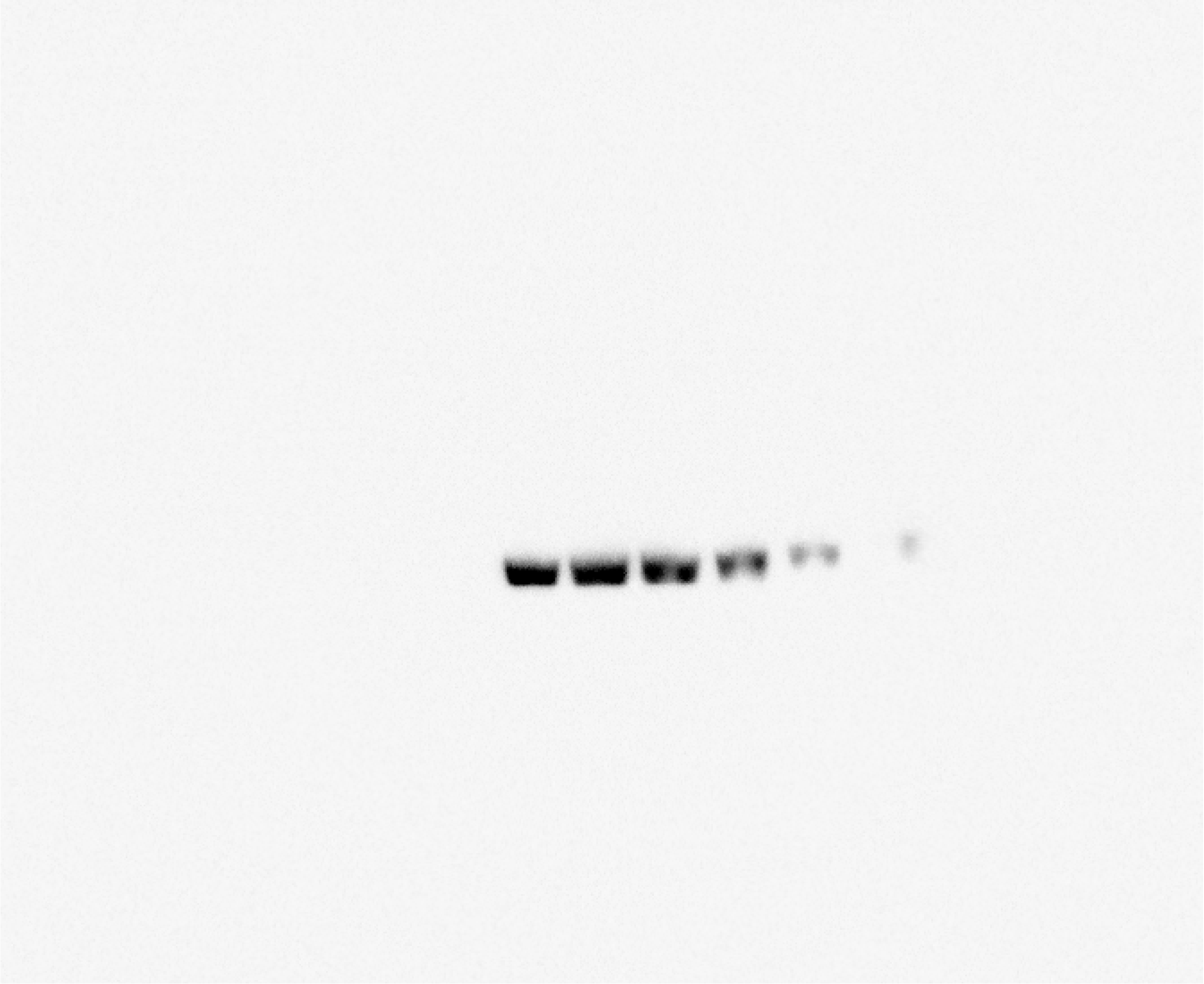

Figure 3J LV-circ GAPDH T24

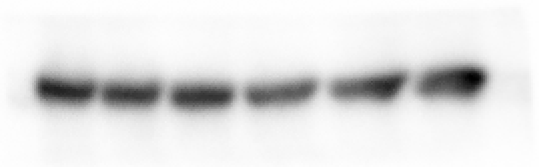

Figure 3J LV-circ GAPDH UMUC-3

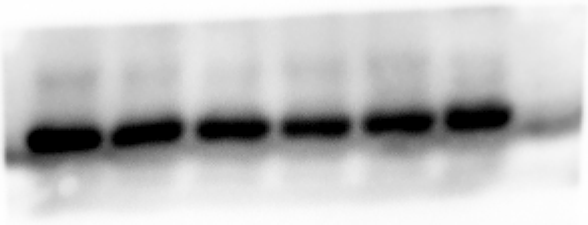

Figure 3J LV-circ MAT2A T24

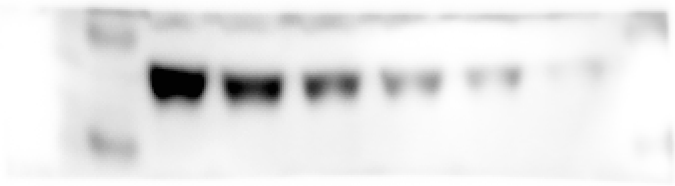

Figure 3J LV-circ MAT2A UMUC-3

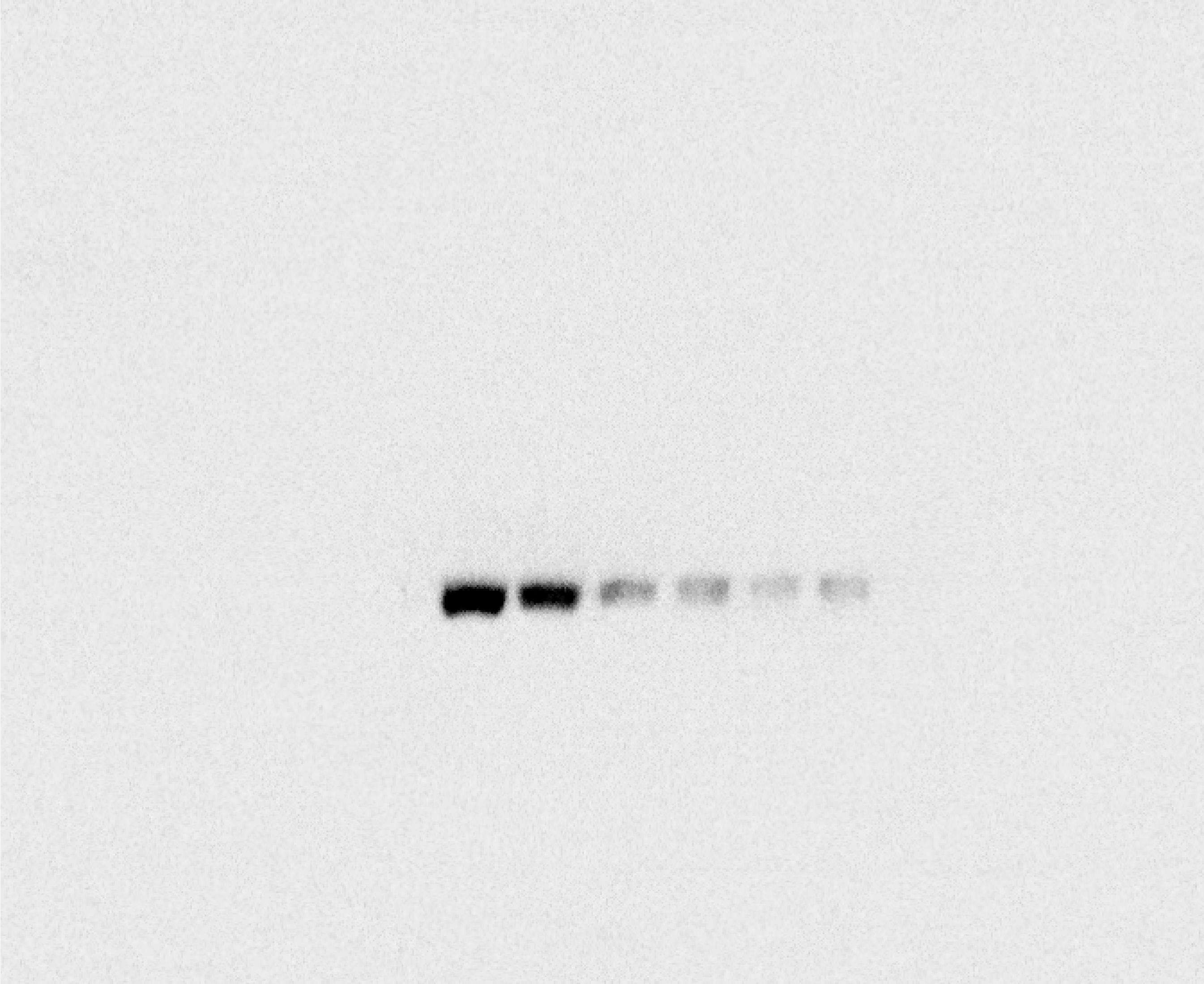

Figure 3J LV-NC GAPDH T24

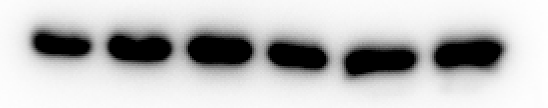

Figure 3J LV-NC GAPDH UMUC-3

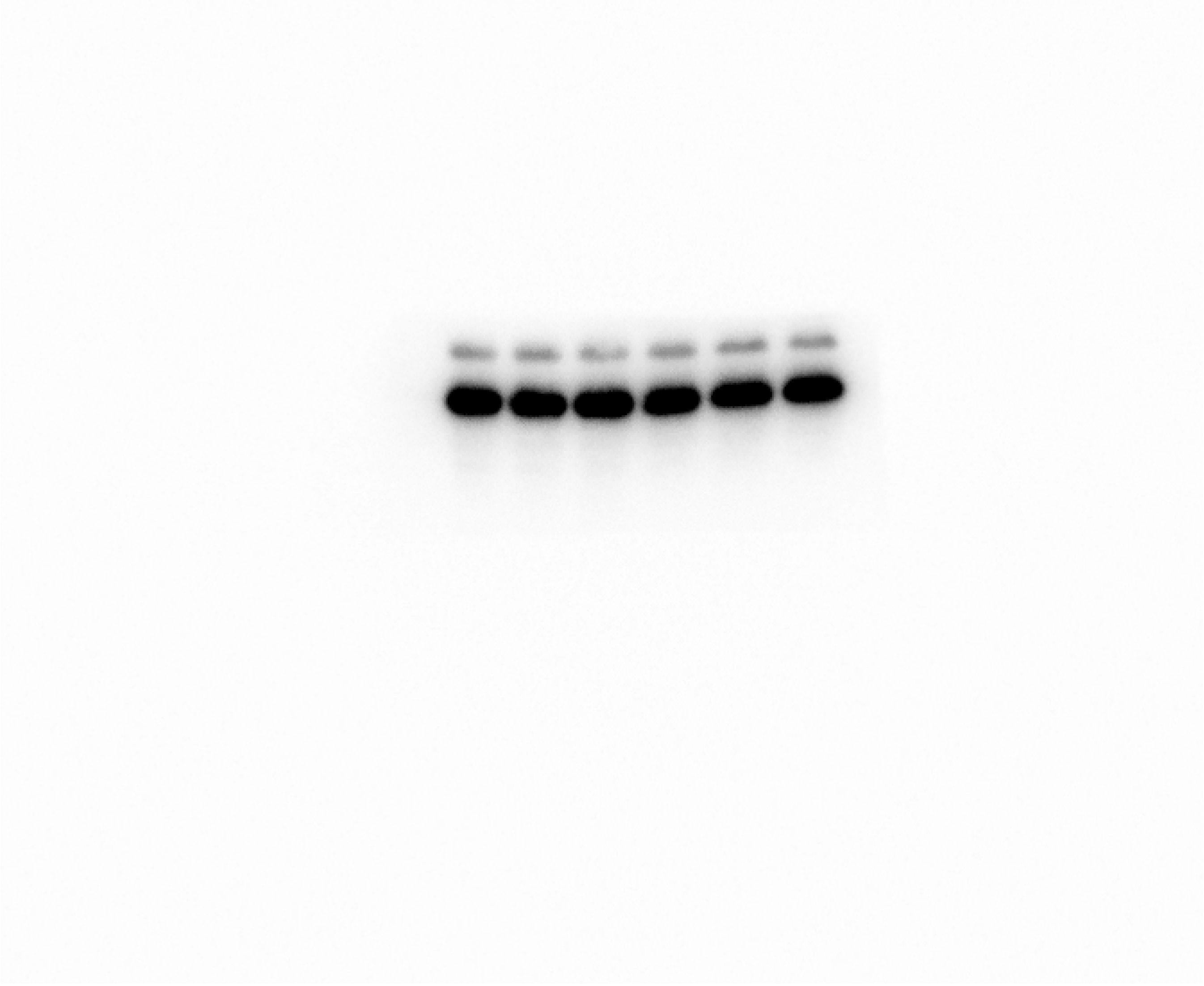

Figure 3J LV-NC MAT2A T24

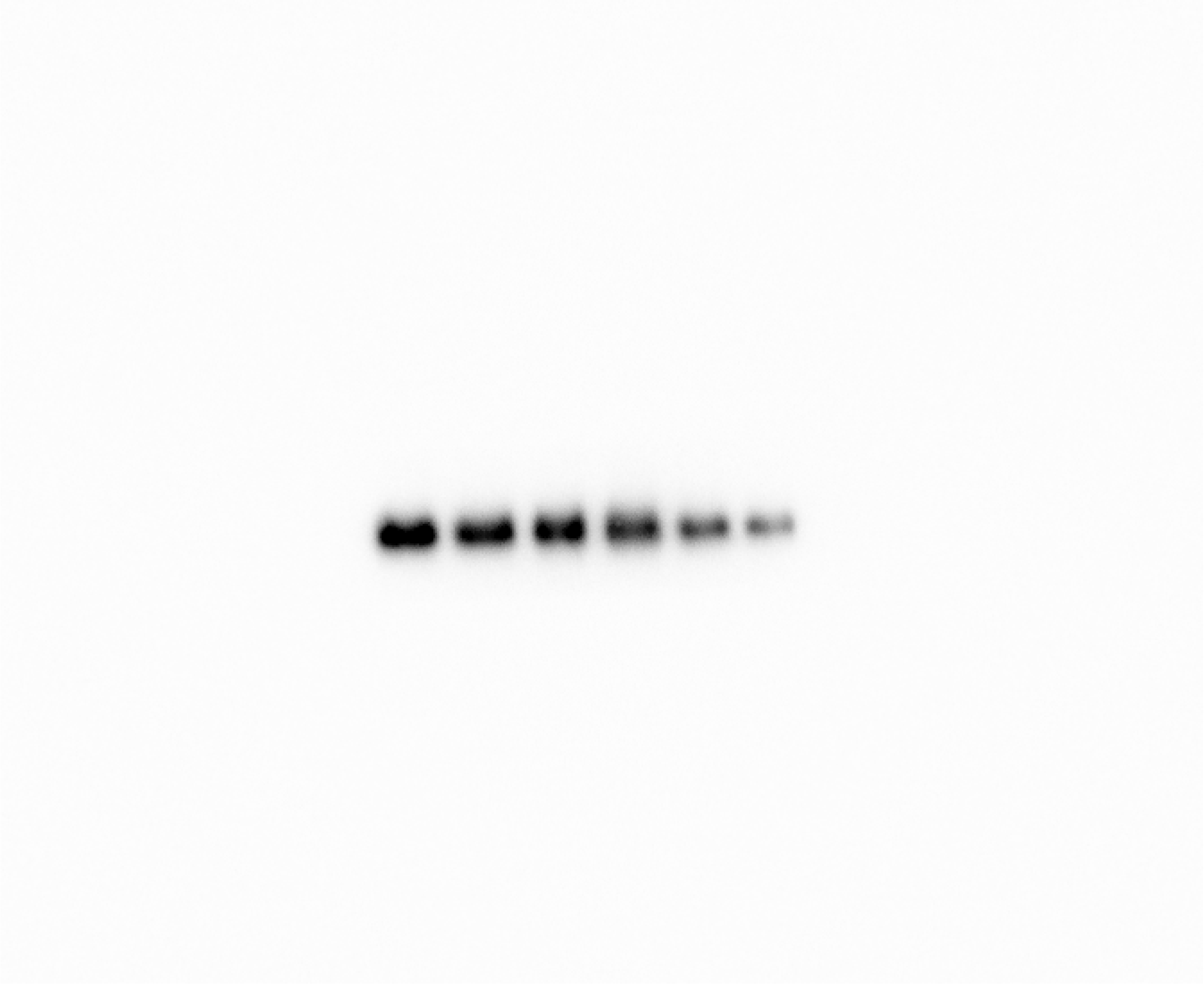

Figure 3K umuc-3 HA

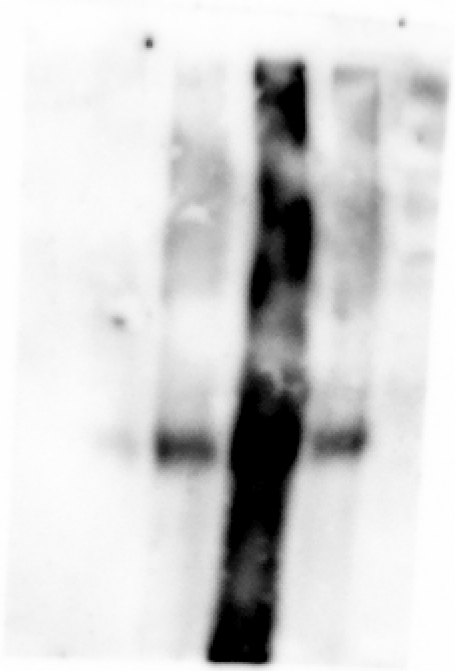

Figure 3K Input T24

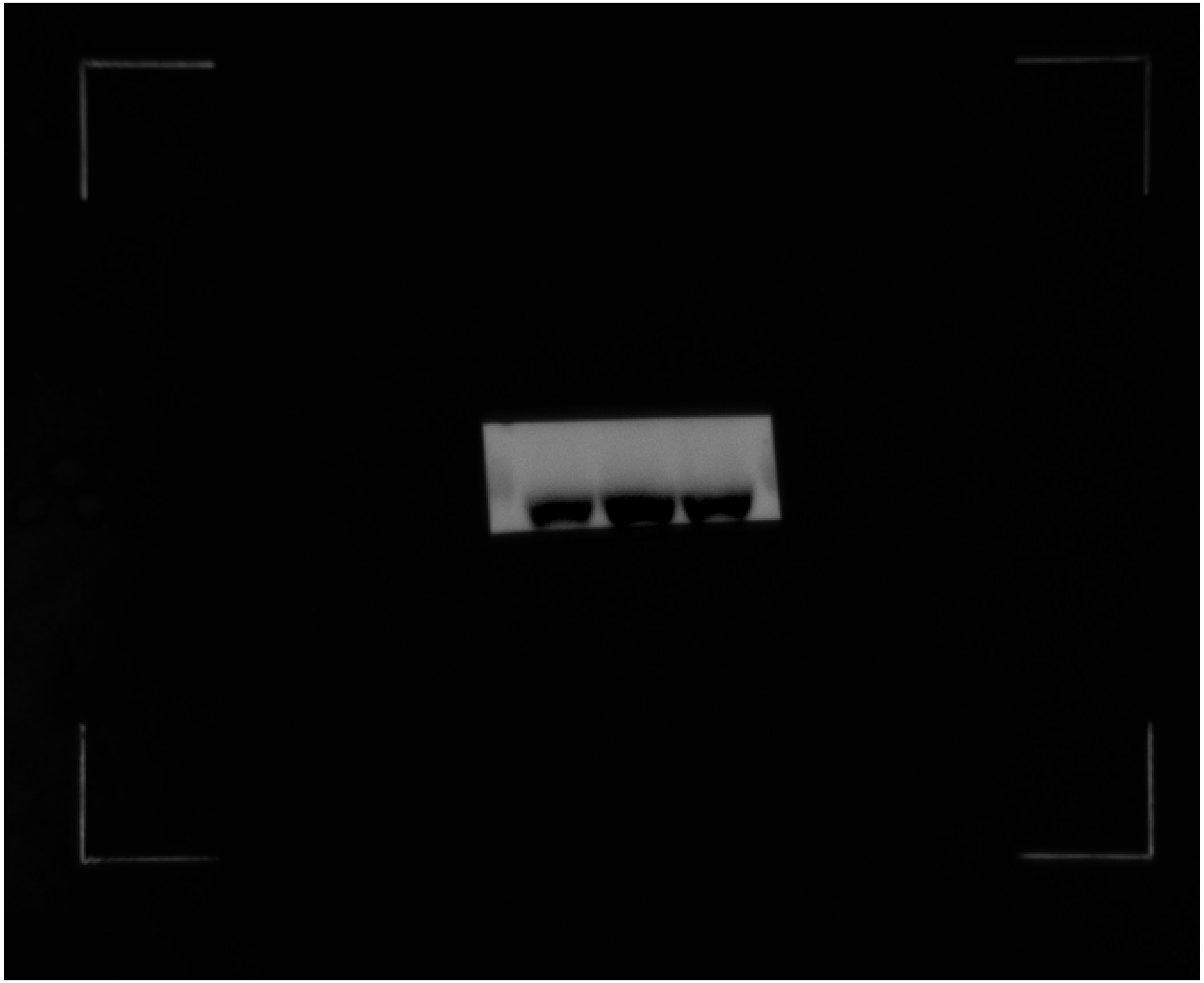

Figure 3K Input umuc-3

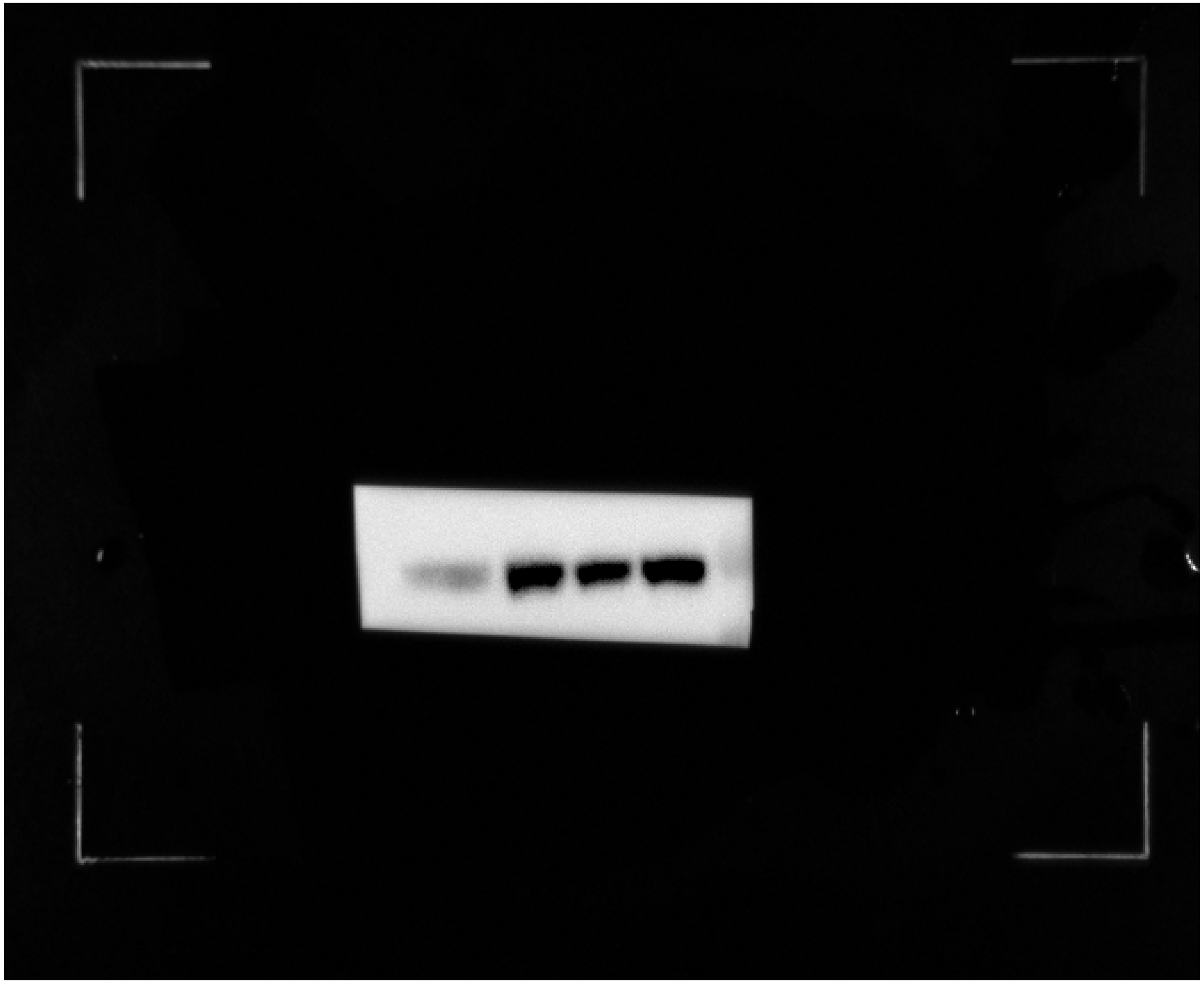

Figure 3K T24 HA

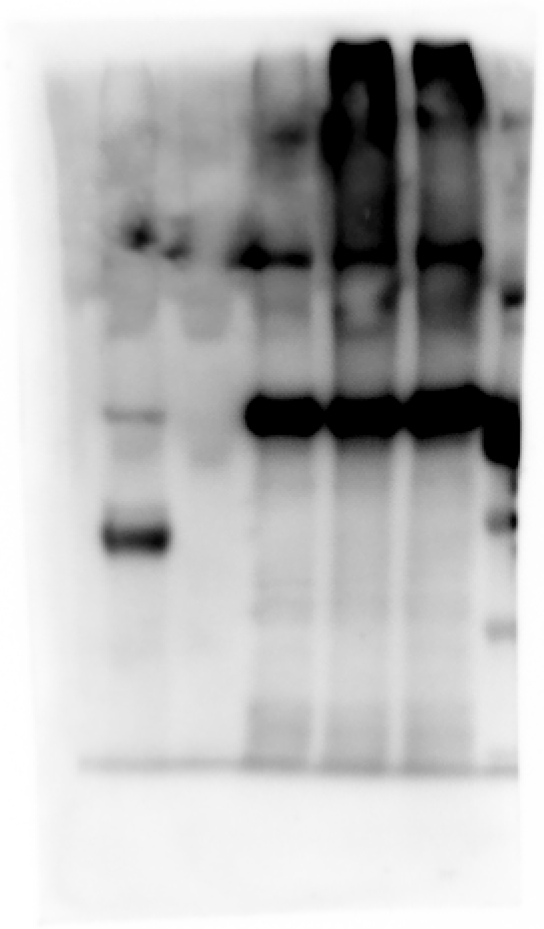

Figure 3L IP myc

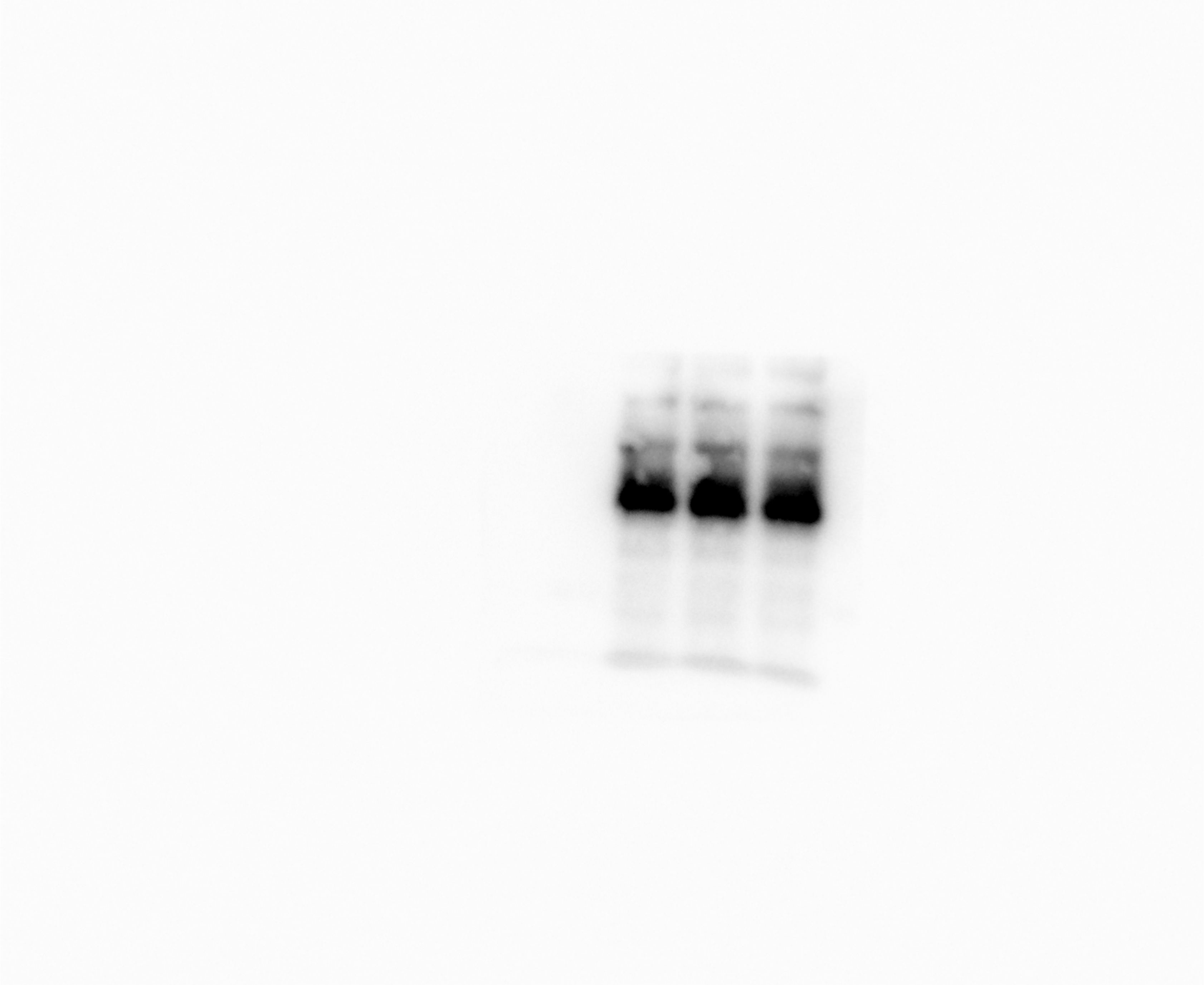

Figure 3L Input GAPDH

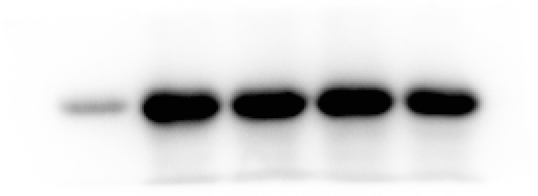

Figure 3L Input HA

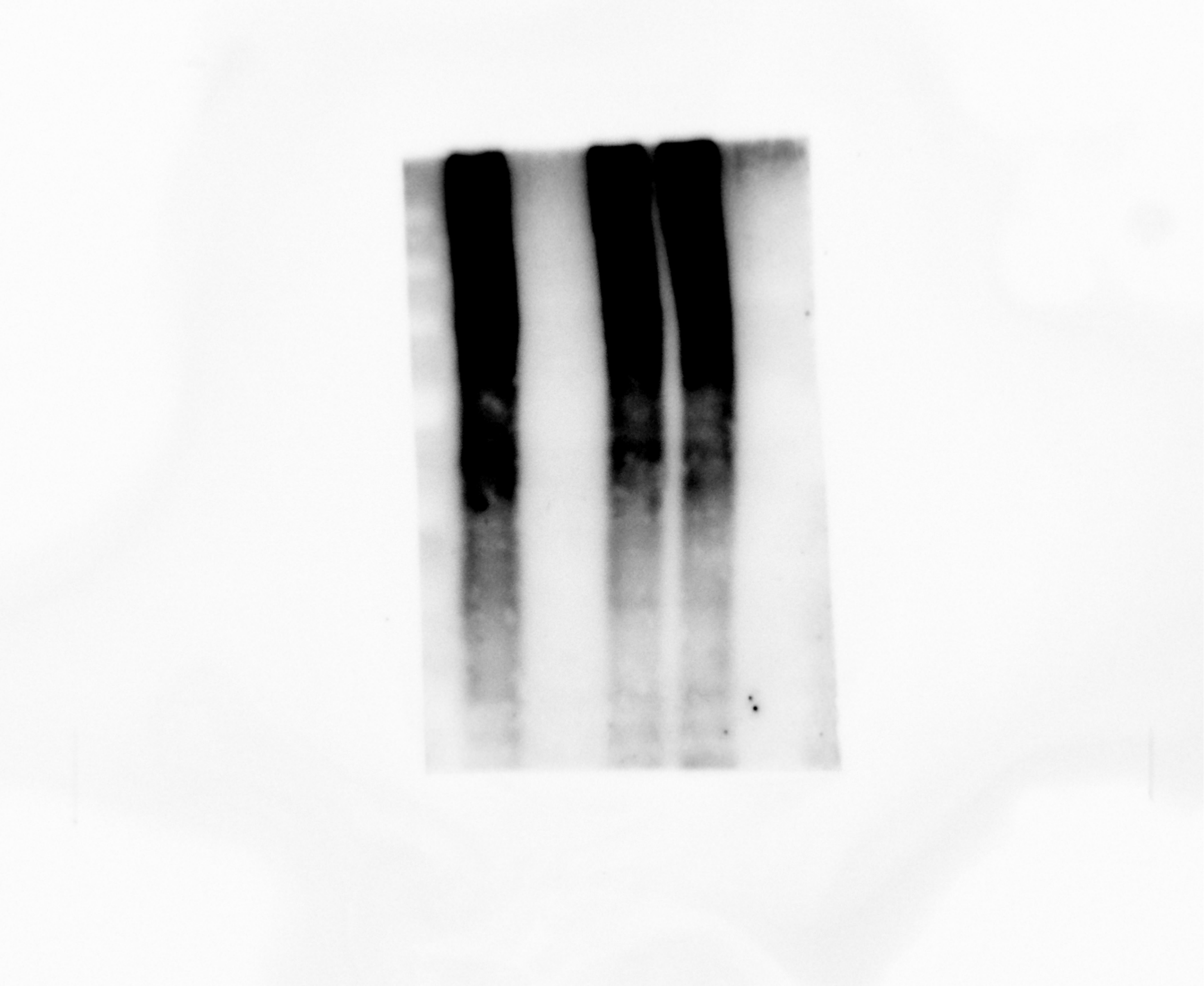

Figure 3L IP HA

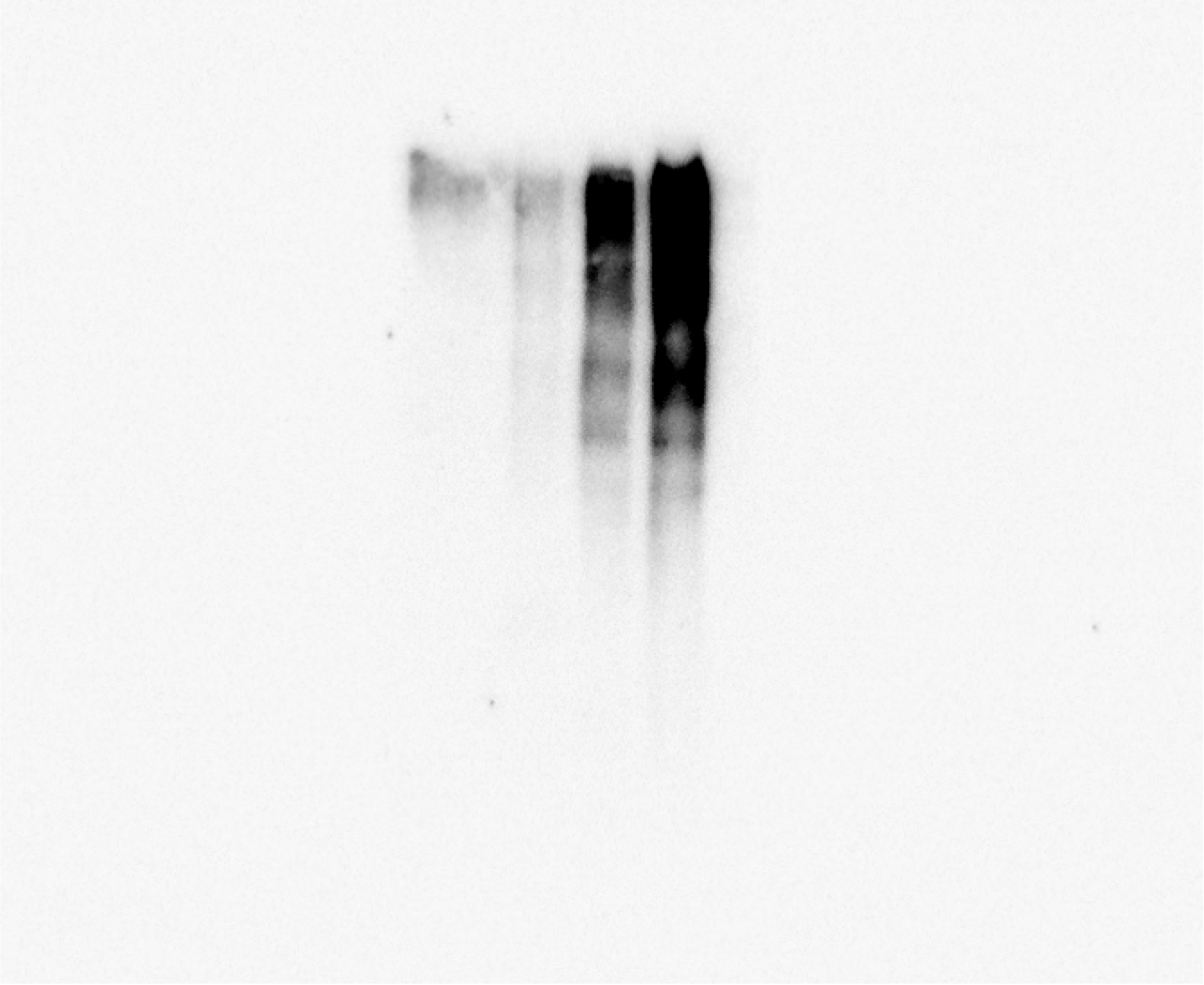

Figure 4C pulldown USP14 UMUC-3

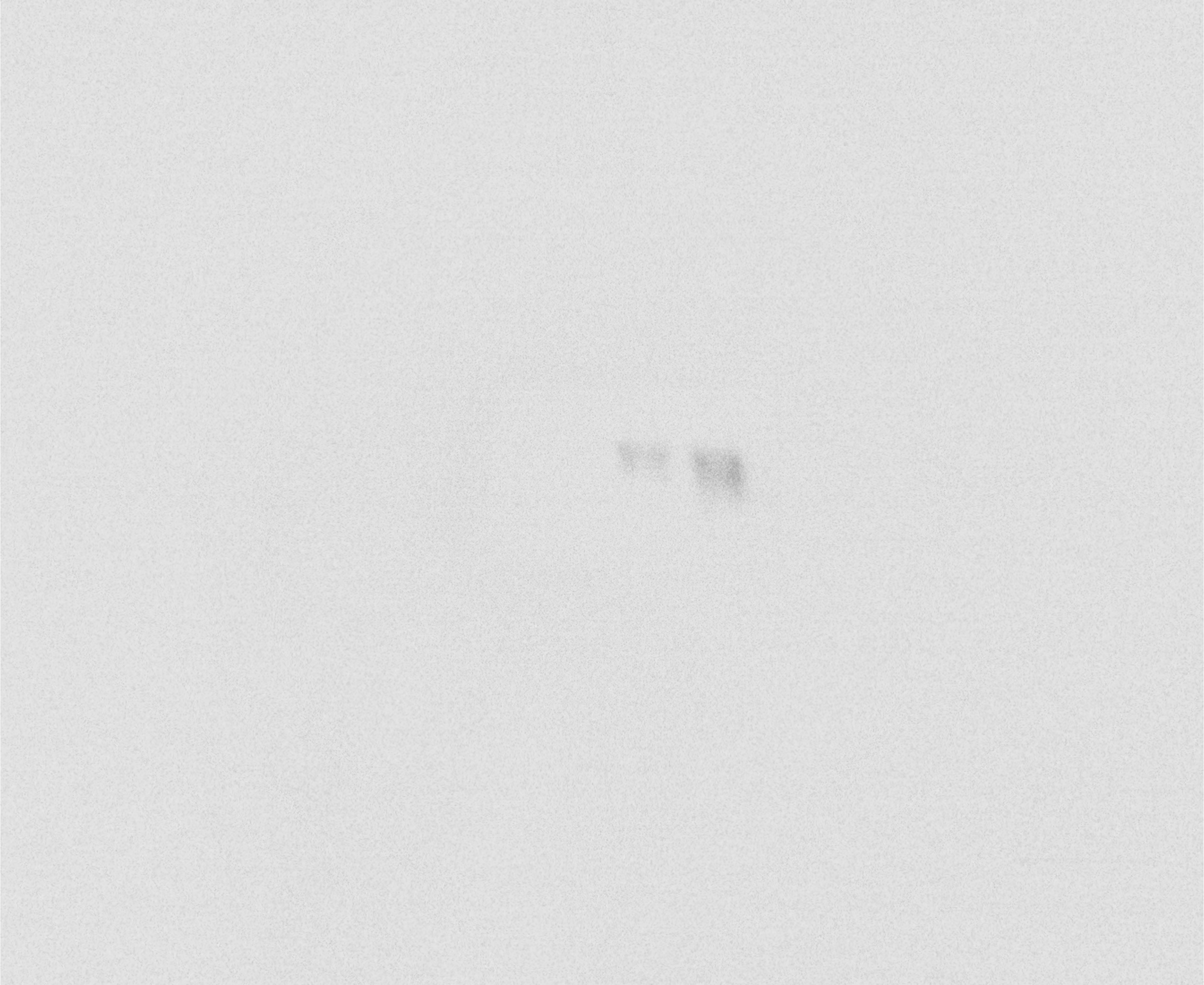

Figure 4C Input TRIM25 T24

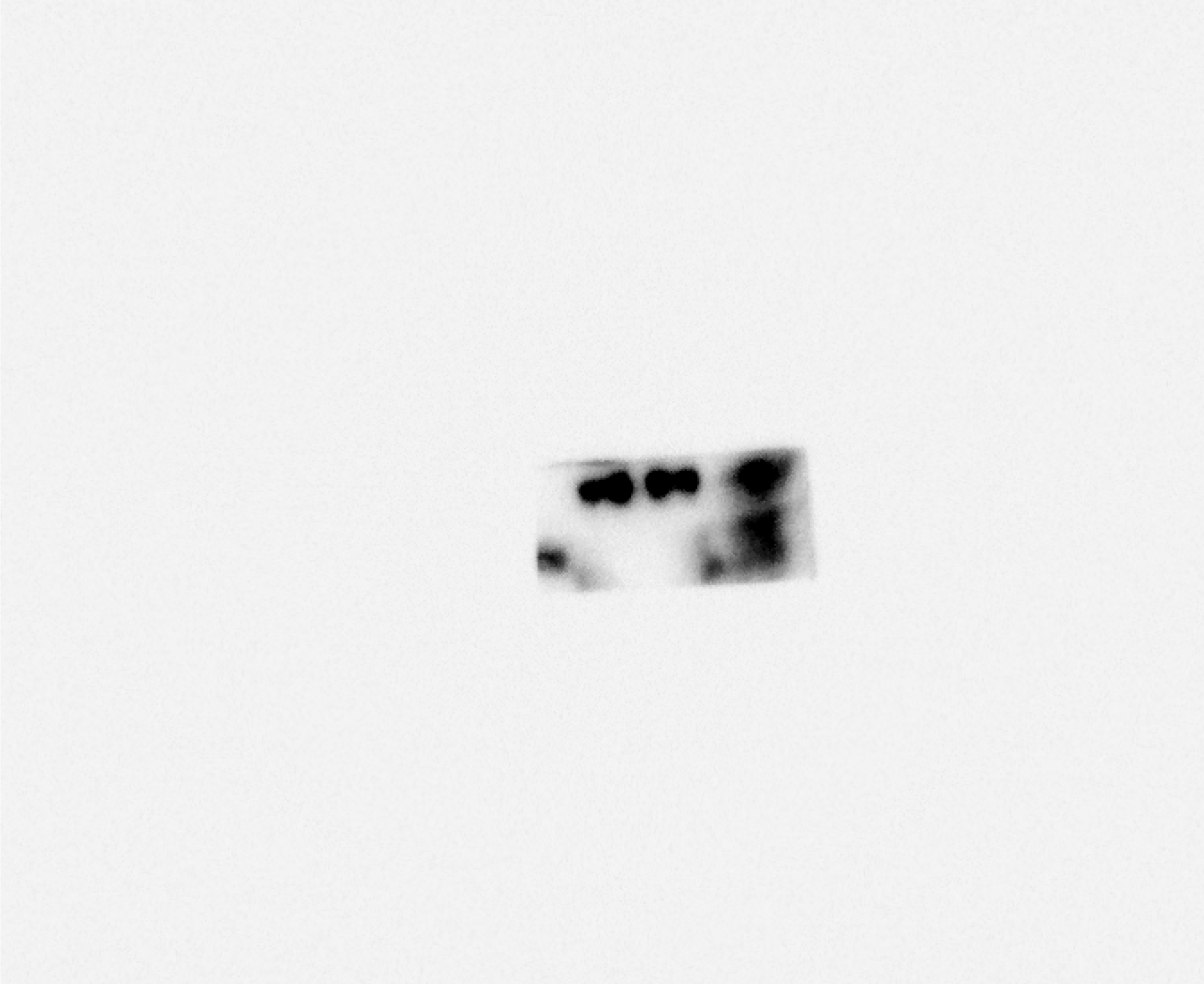

Figure 4C Input TRIM25 UMUC--3

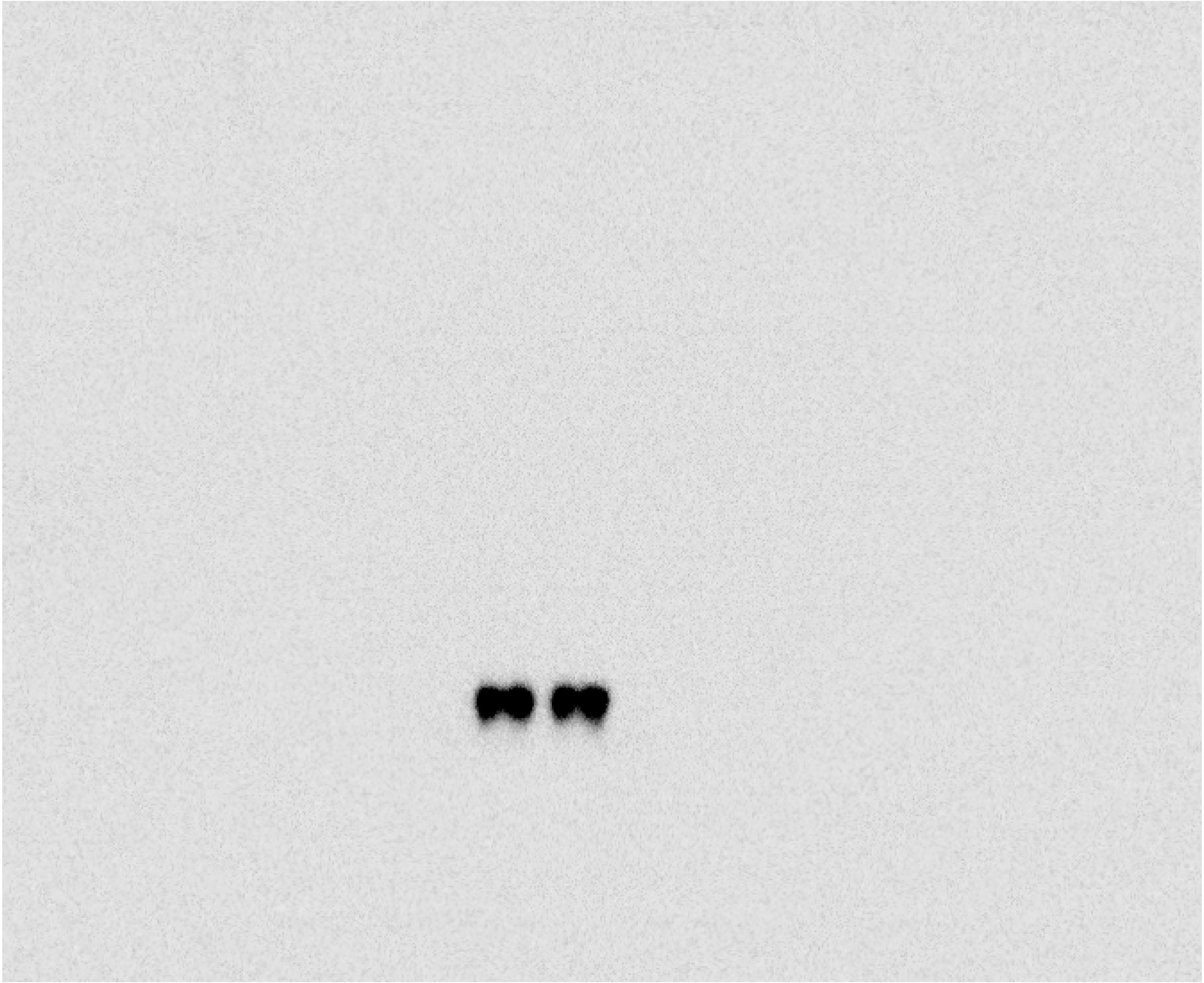

Figure 4C Input USP5 T24

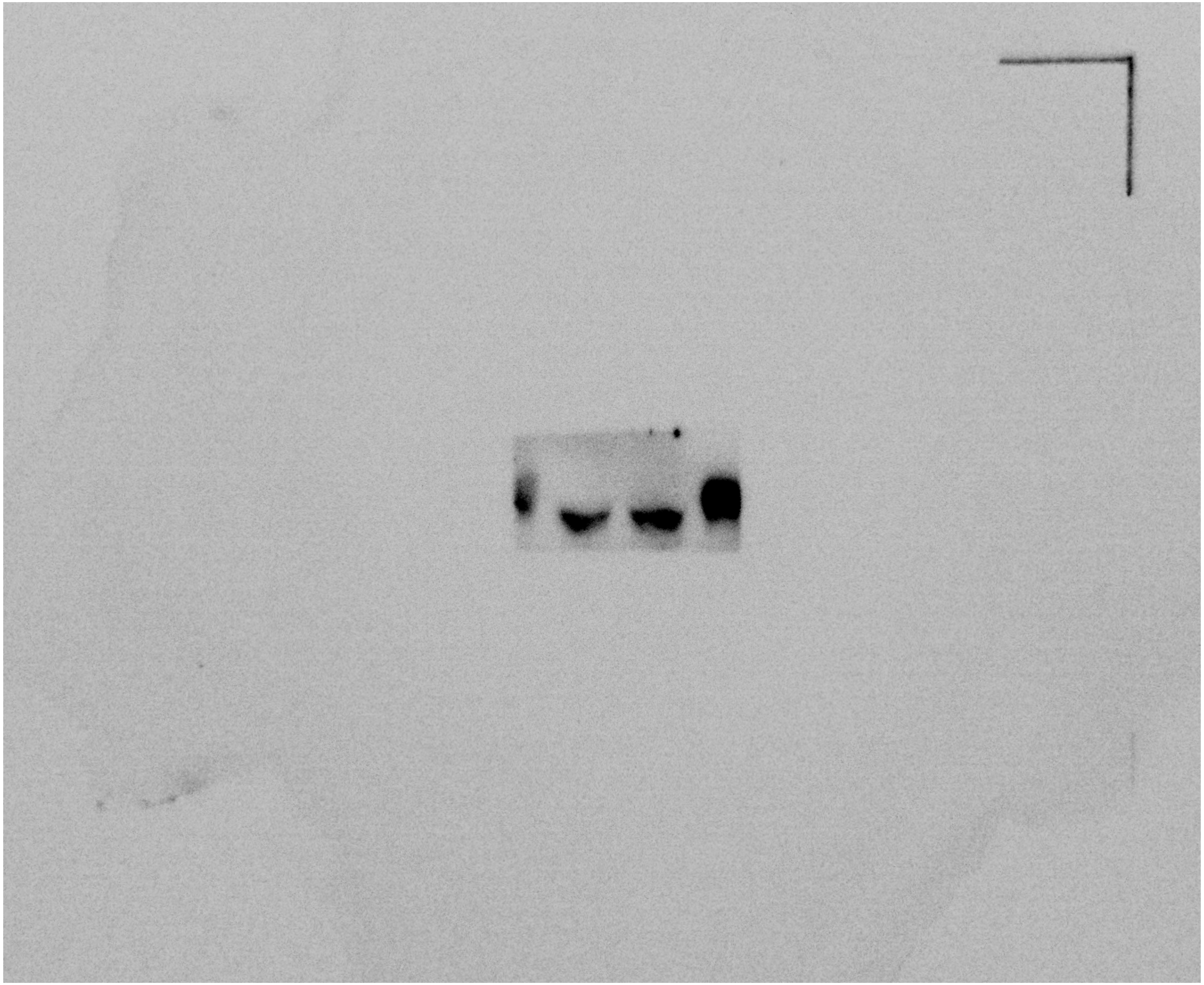

Figure 4C Input USP5 UMUC-3

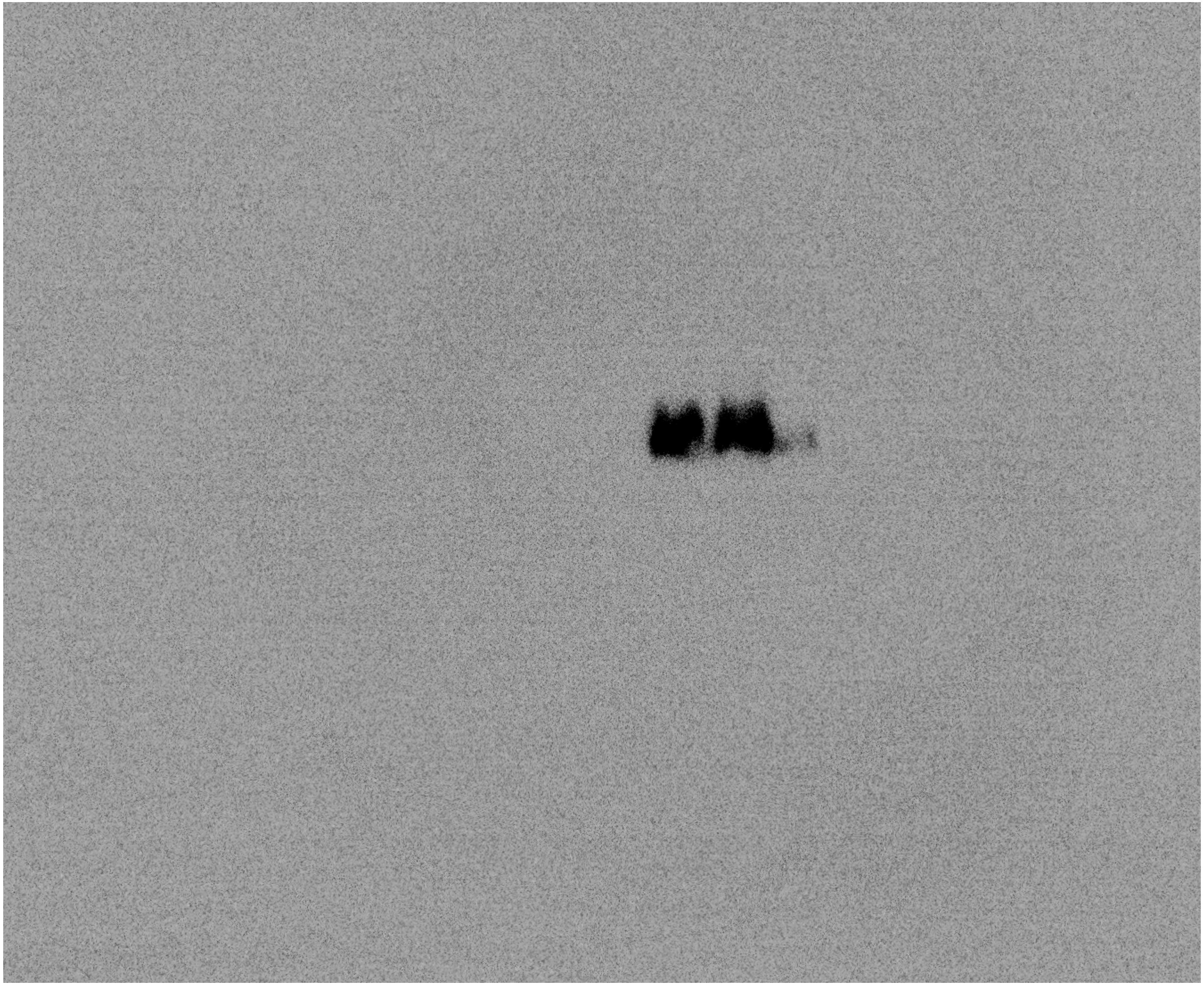

Figure 4C Input USP14 T24

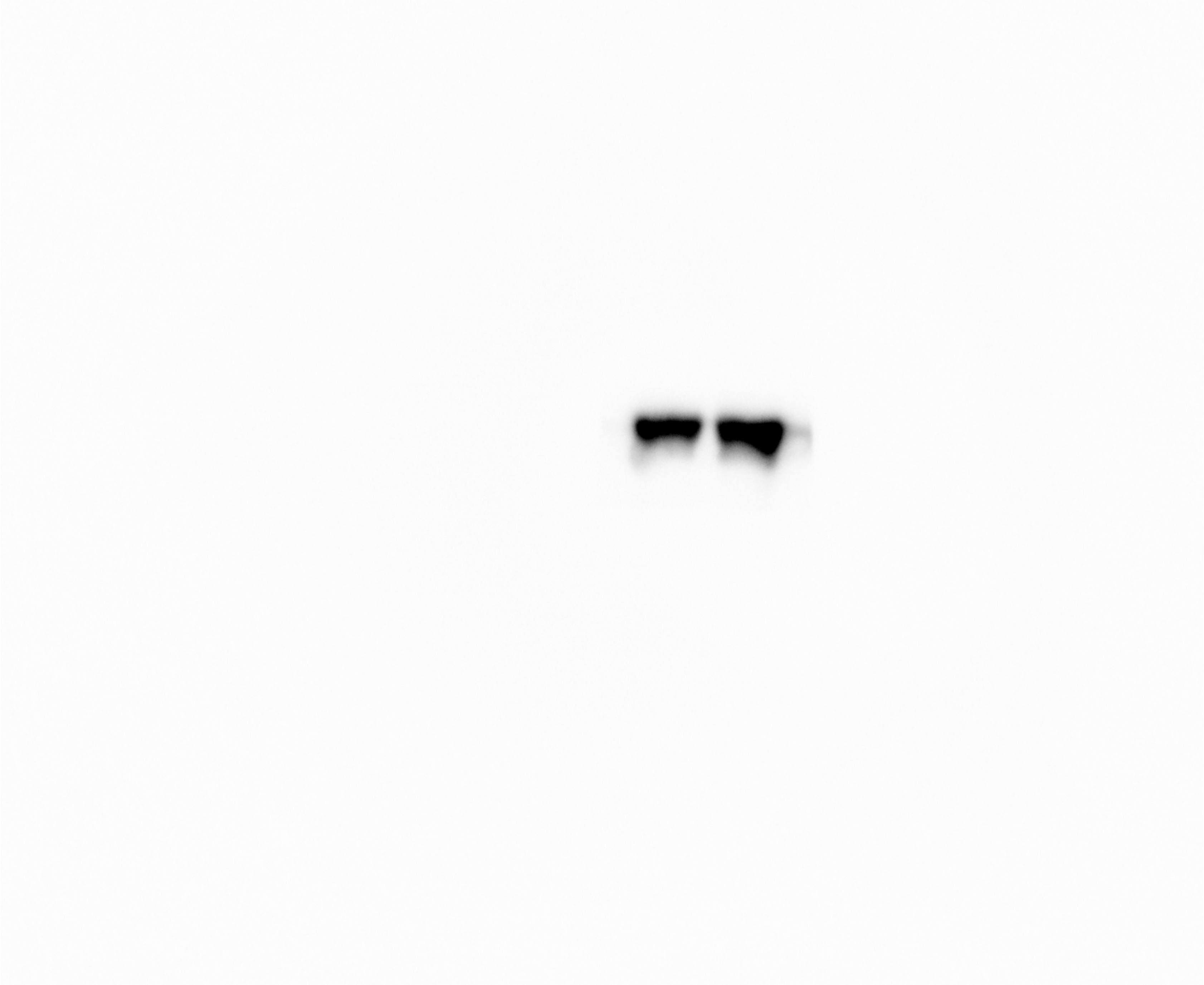

Figure 4C Input USP14 UMUC-3

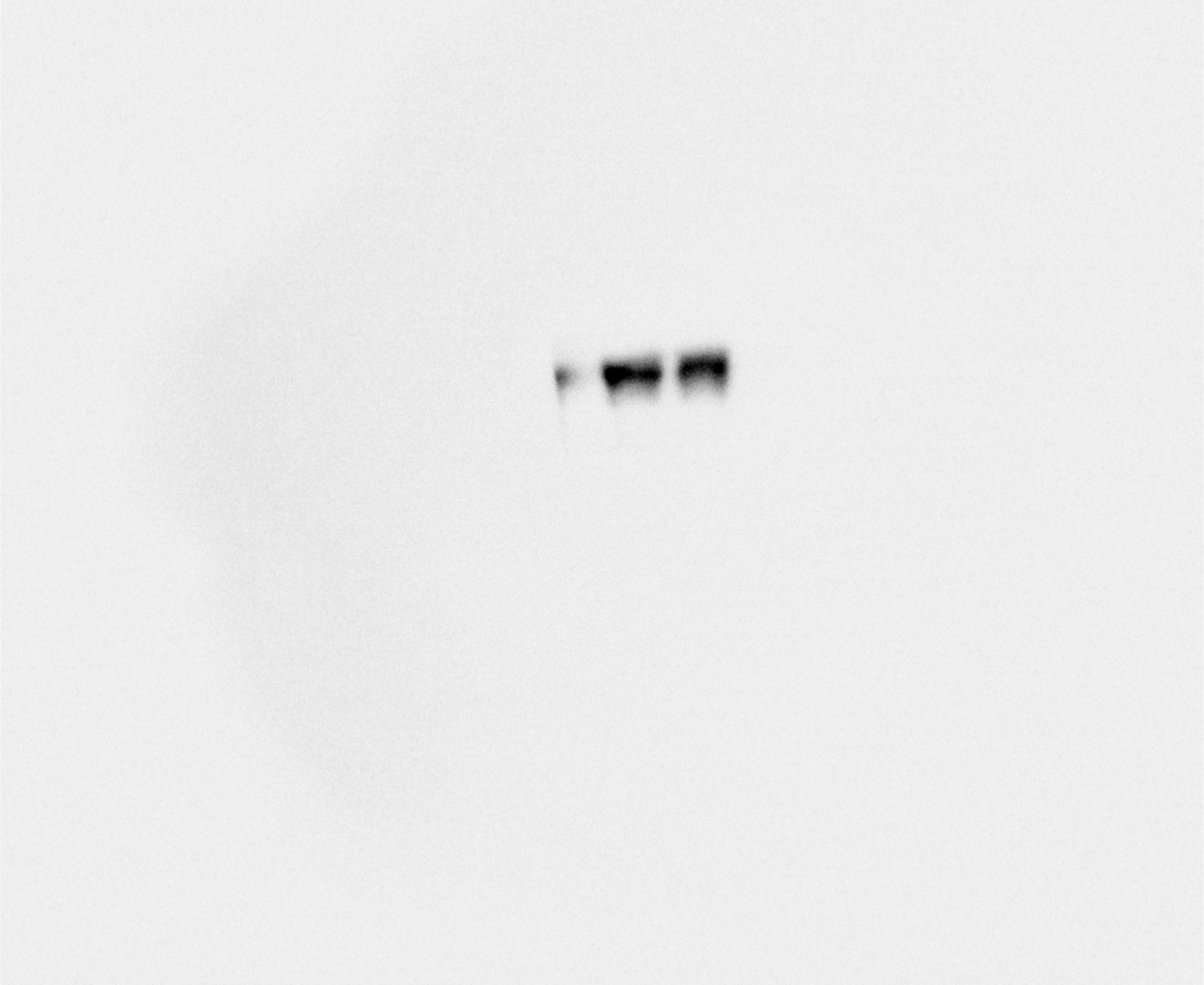

Figure 4C pulldown TRIM25 T24

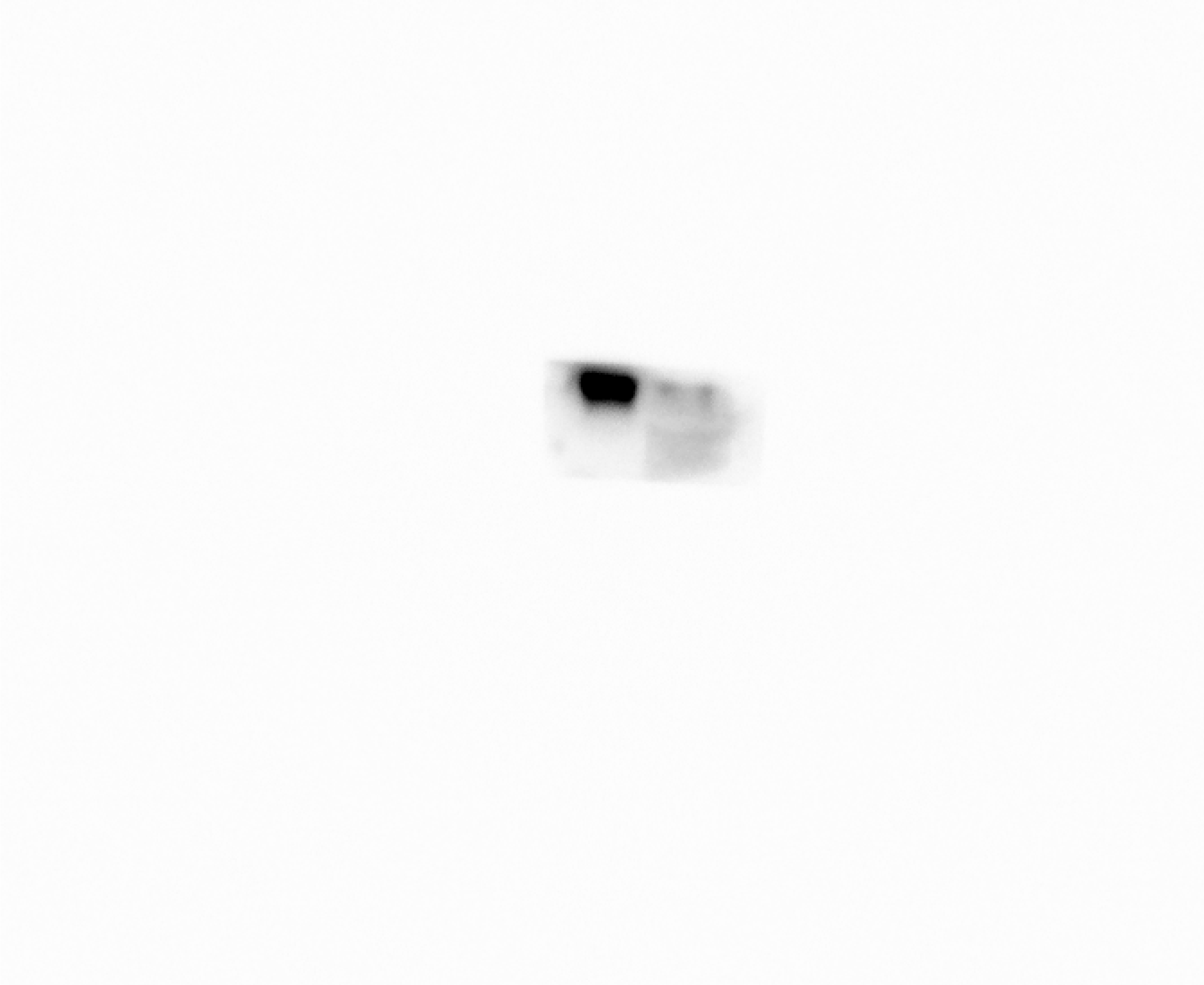

Figure 4C pulldown TRIM25 UMUC-3

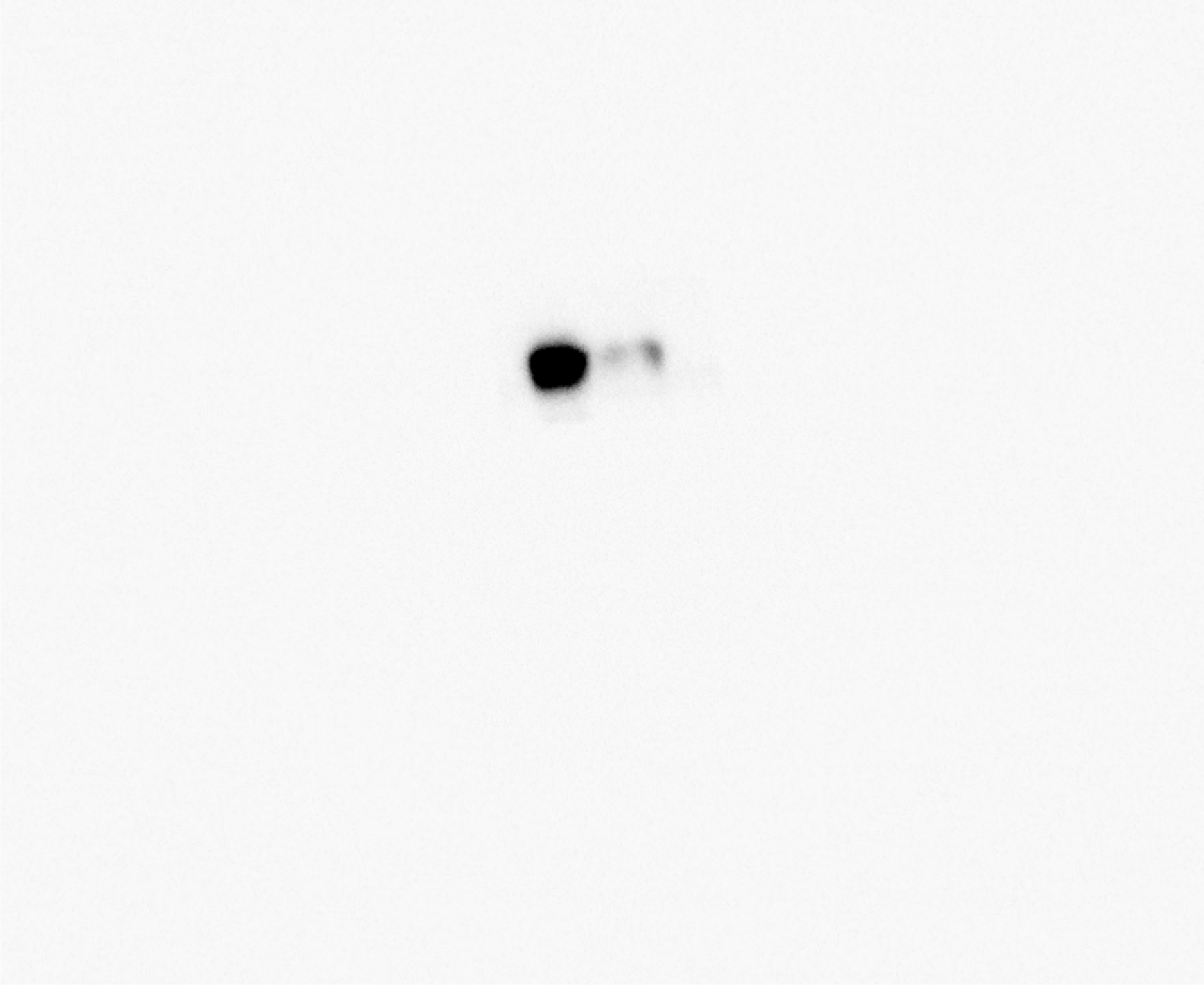

Figure 4C pulldown USP5 T24

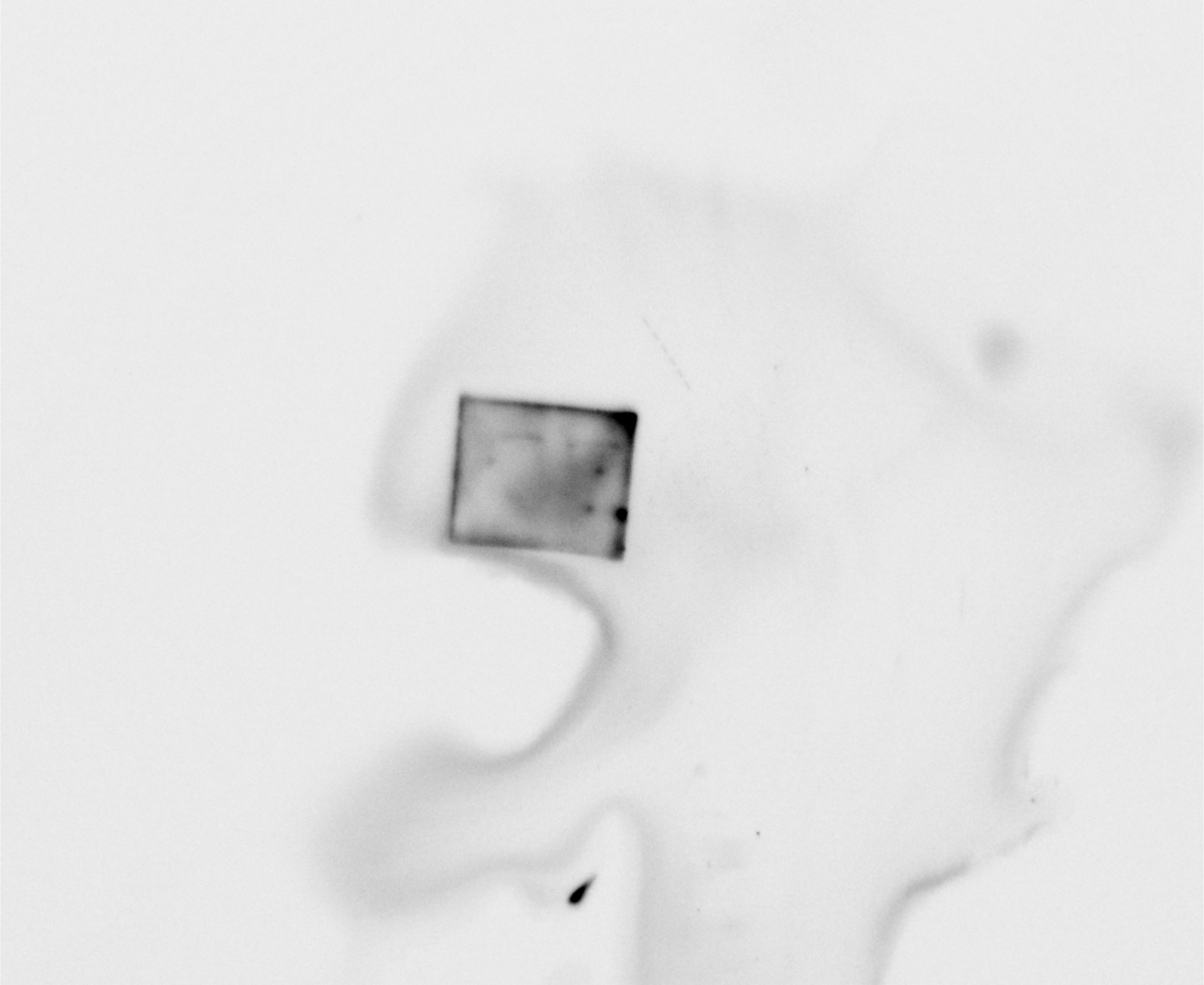

Figure 4C pulldown USP5 UMUC-3

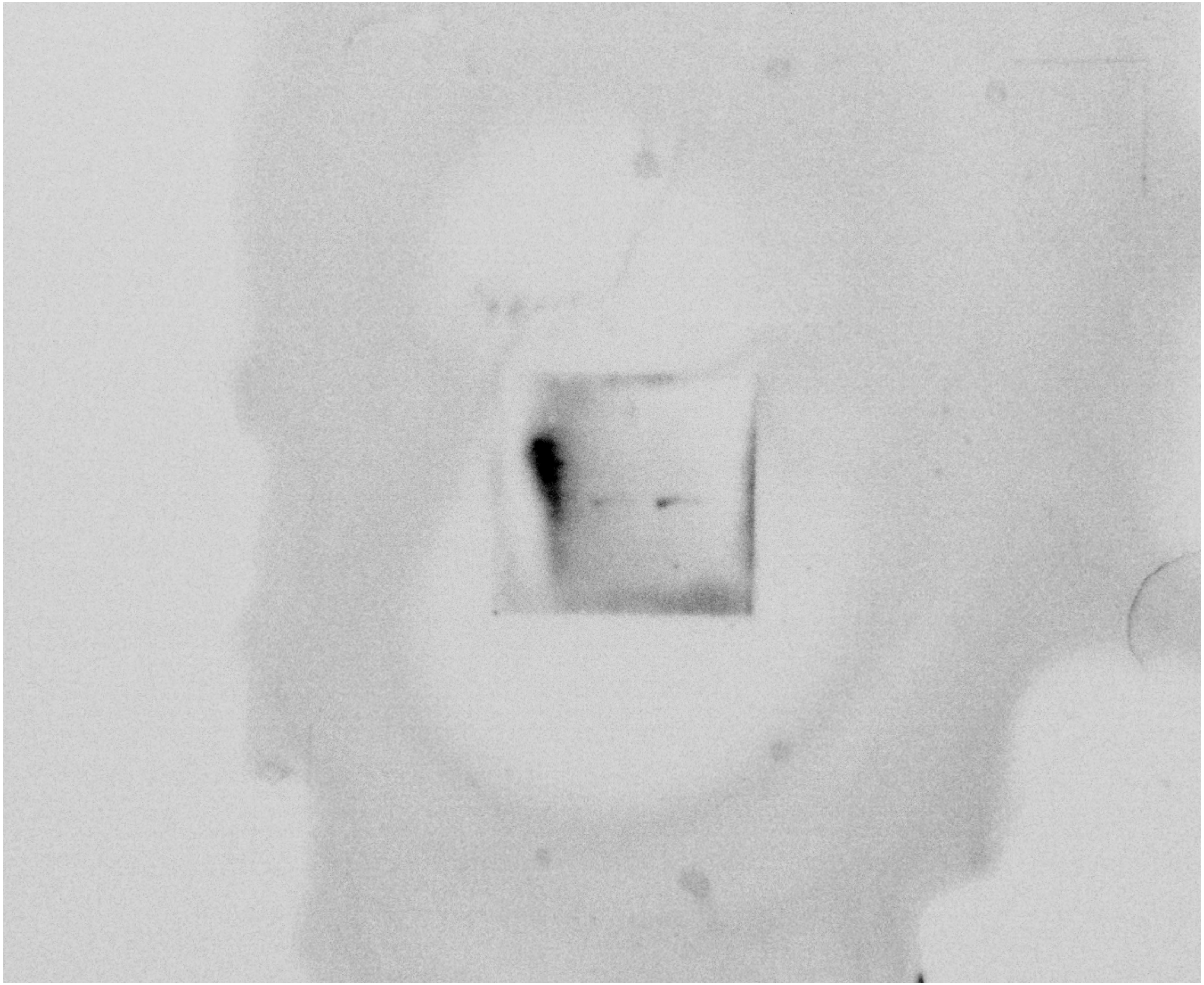

Figure 4C pulldown USP14 T24

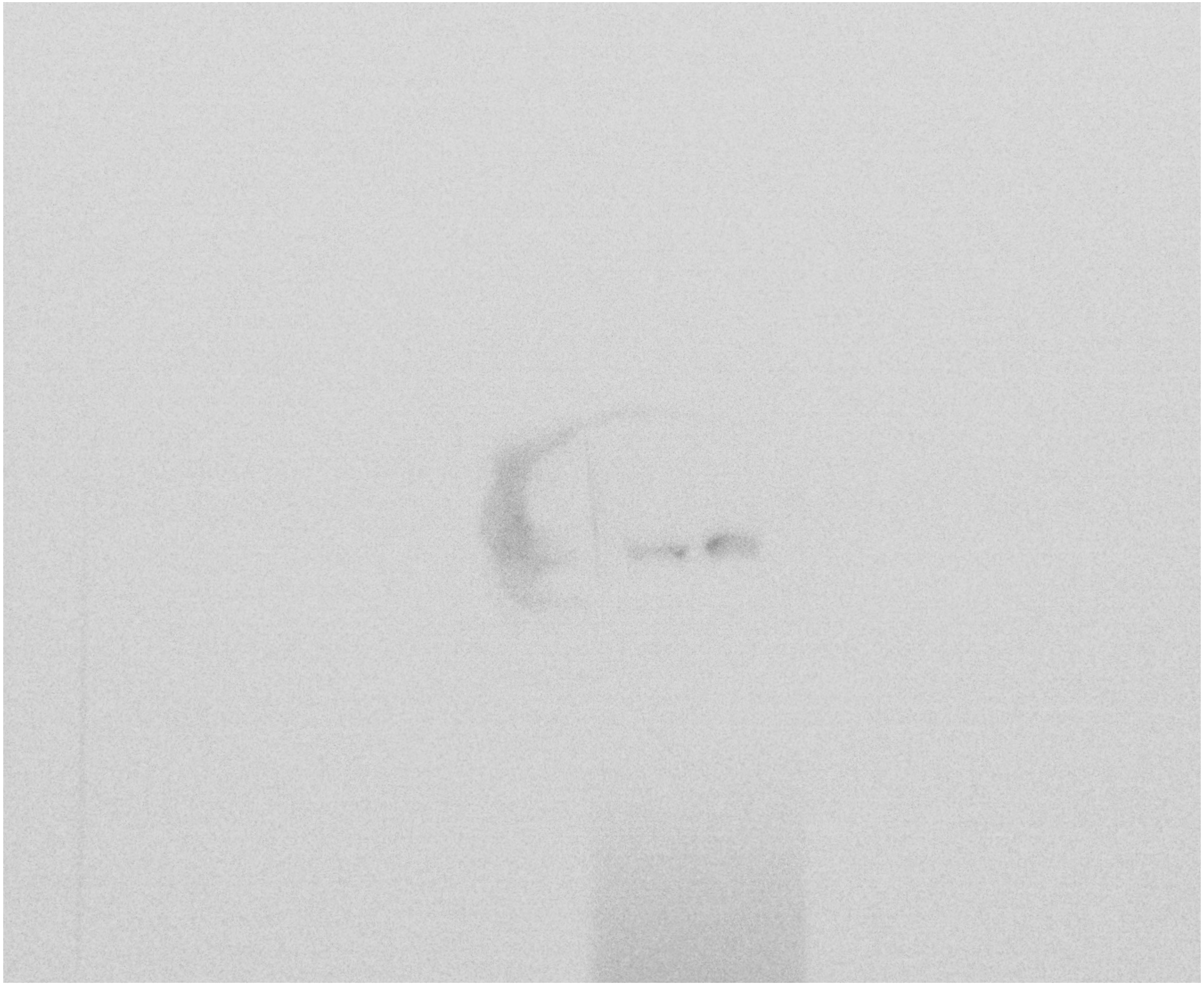

Figure 4E IP TRIM25 IgT

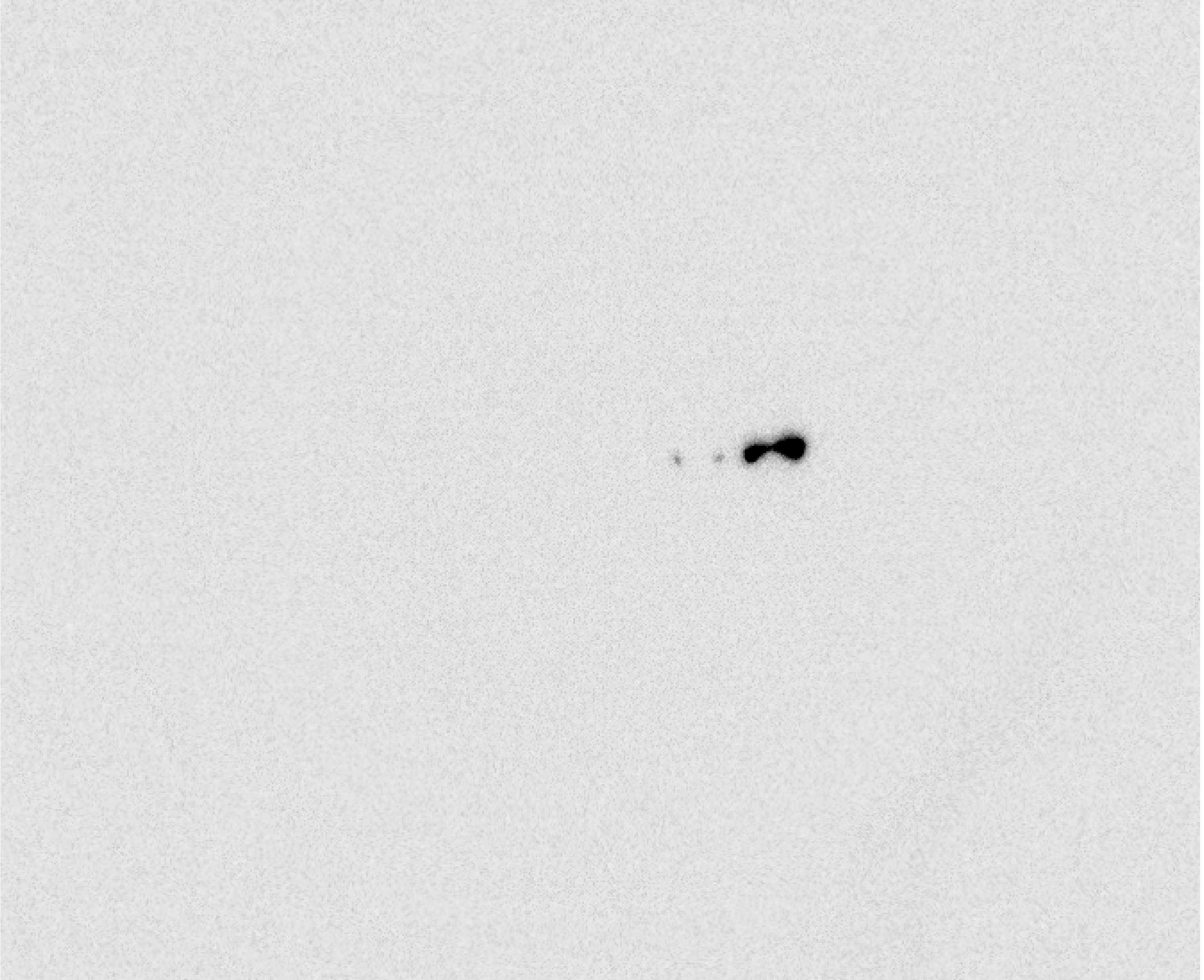

Figure 4E Input MAT2A IgM

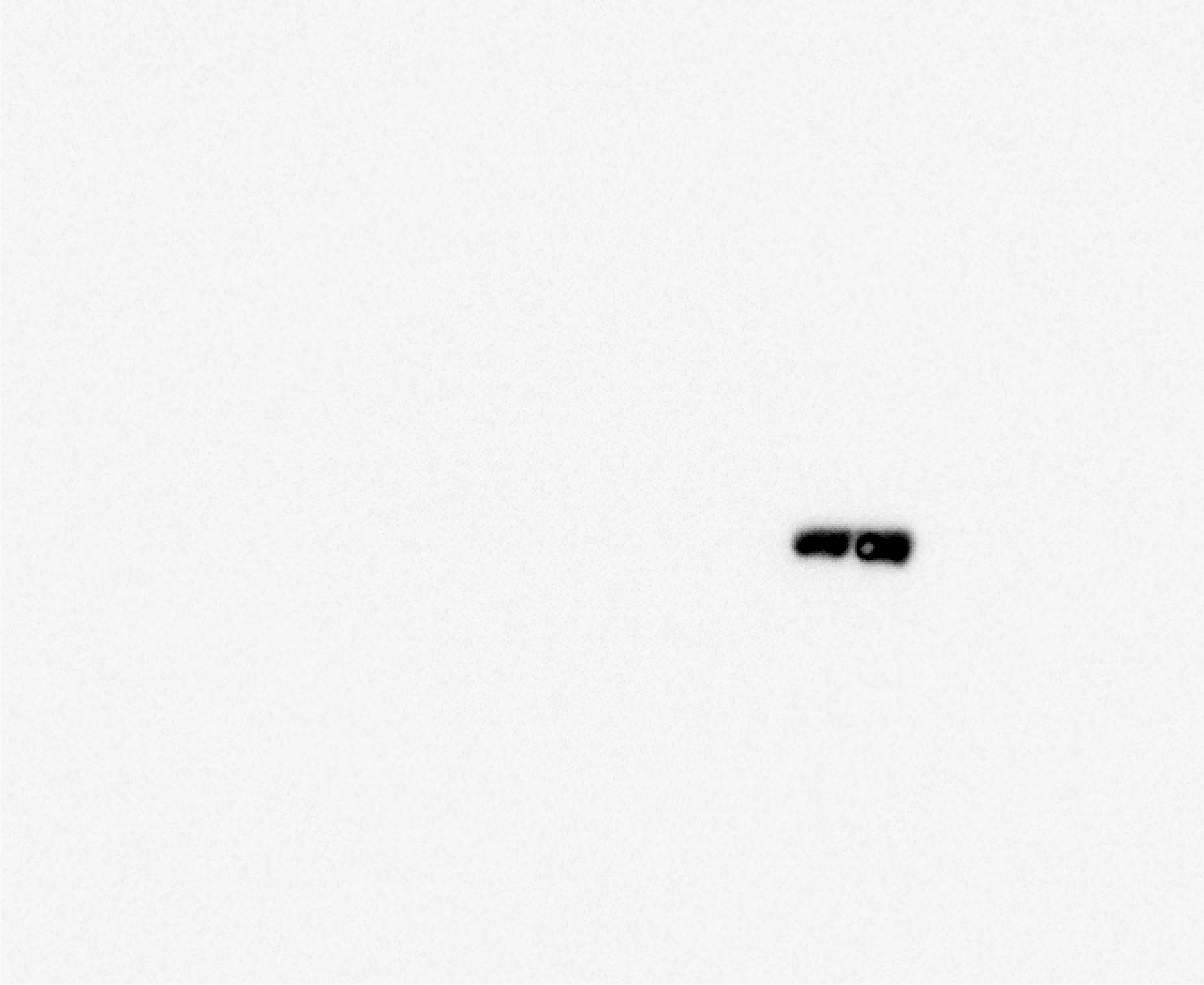

Figure 4E Input MAT2A IgT

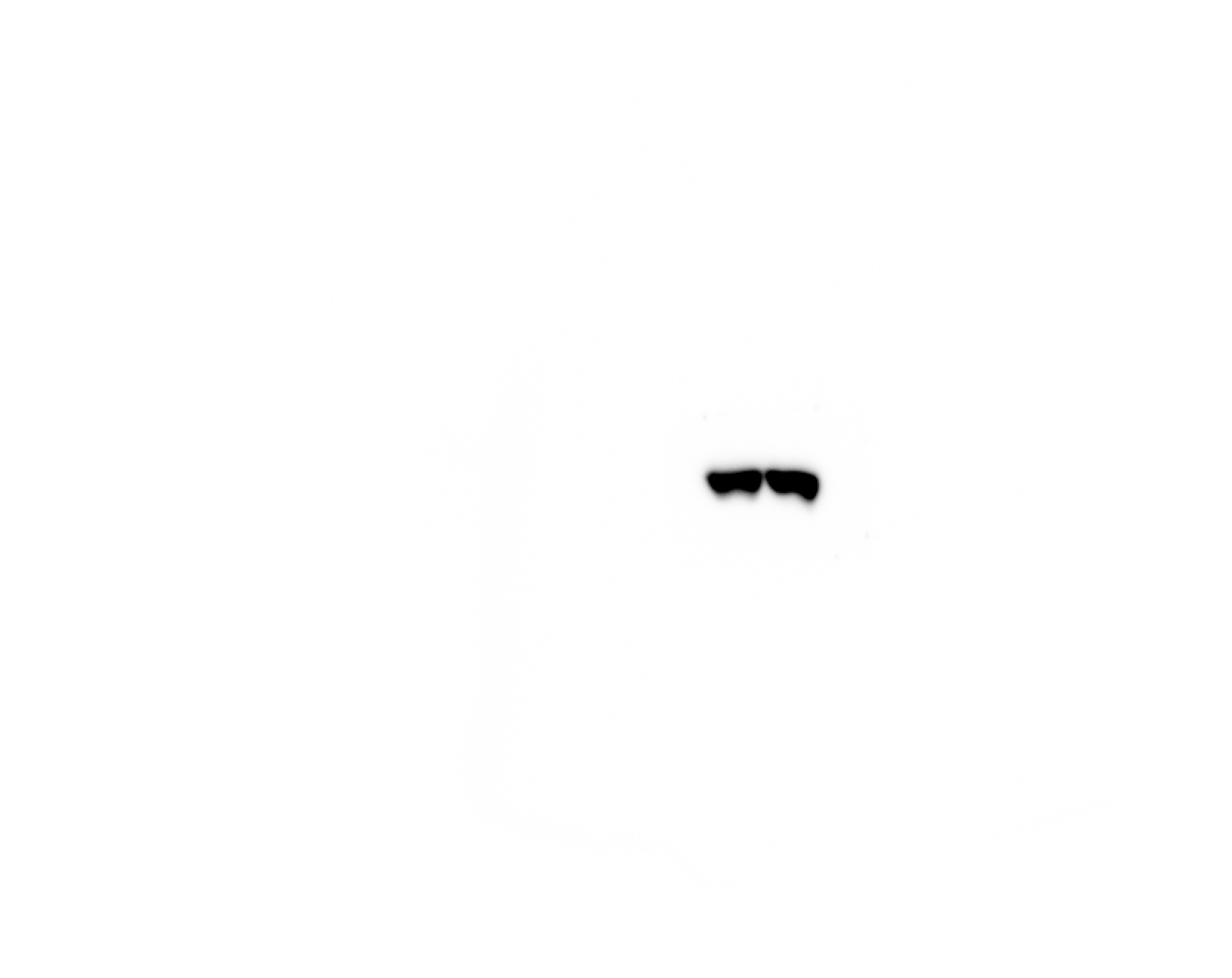

Figure 4E Input TRIM25 IgM

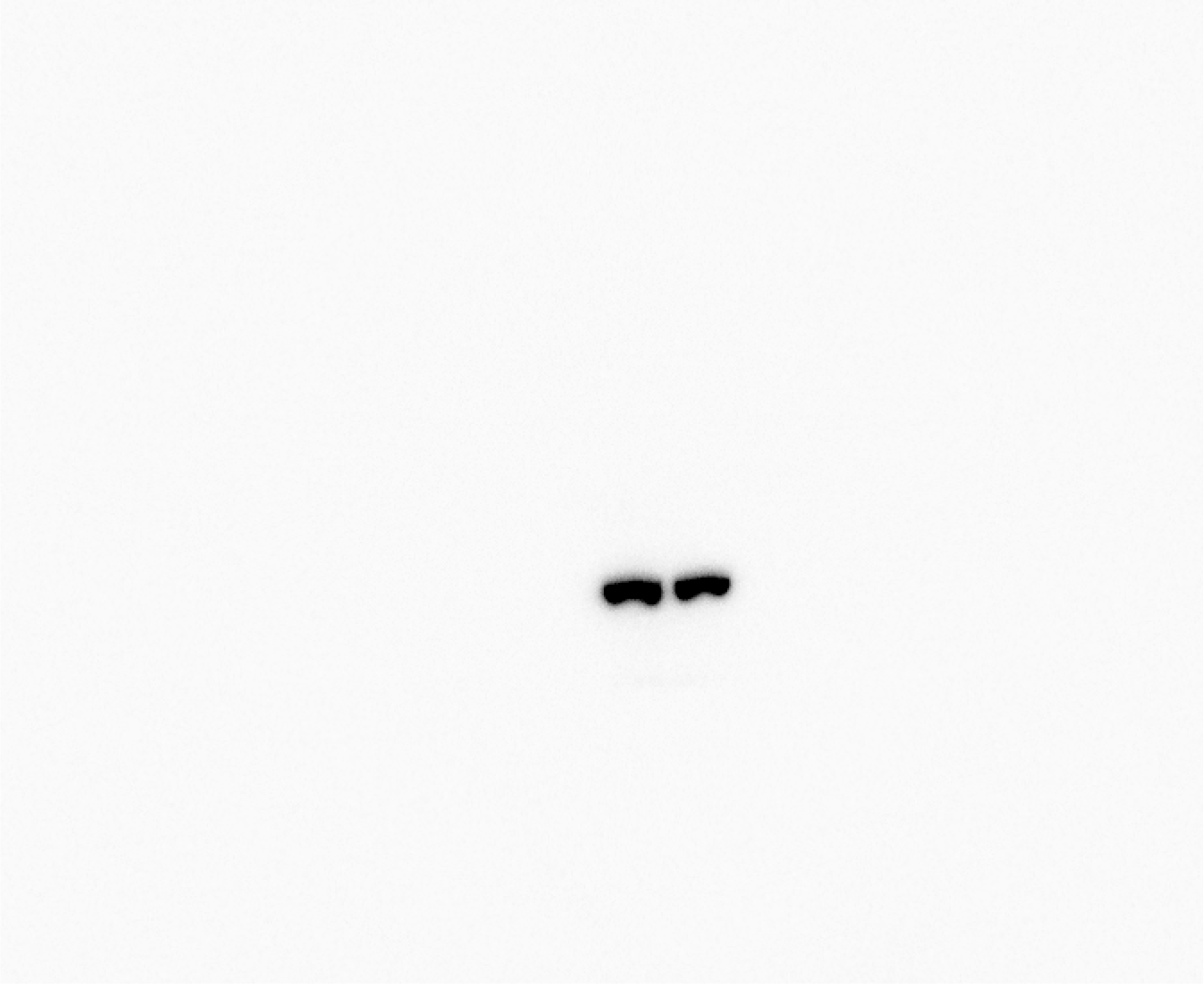

Figure 4E Input TRIM25 IgT

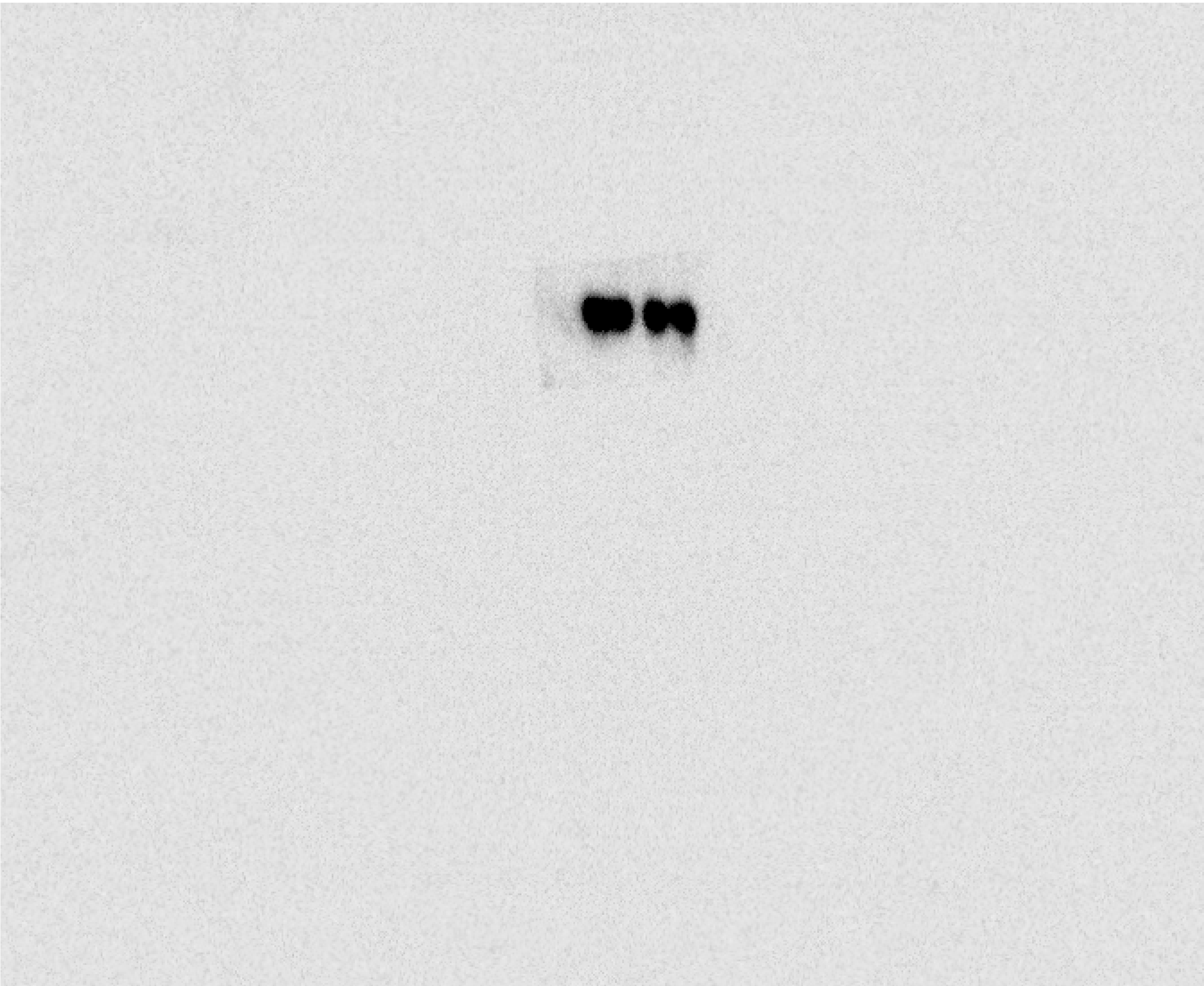

Figure 4E IP MAT2A IgM

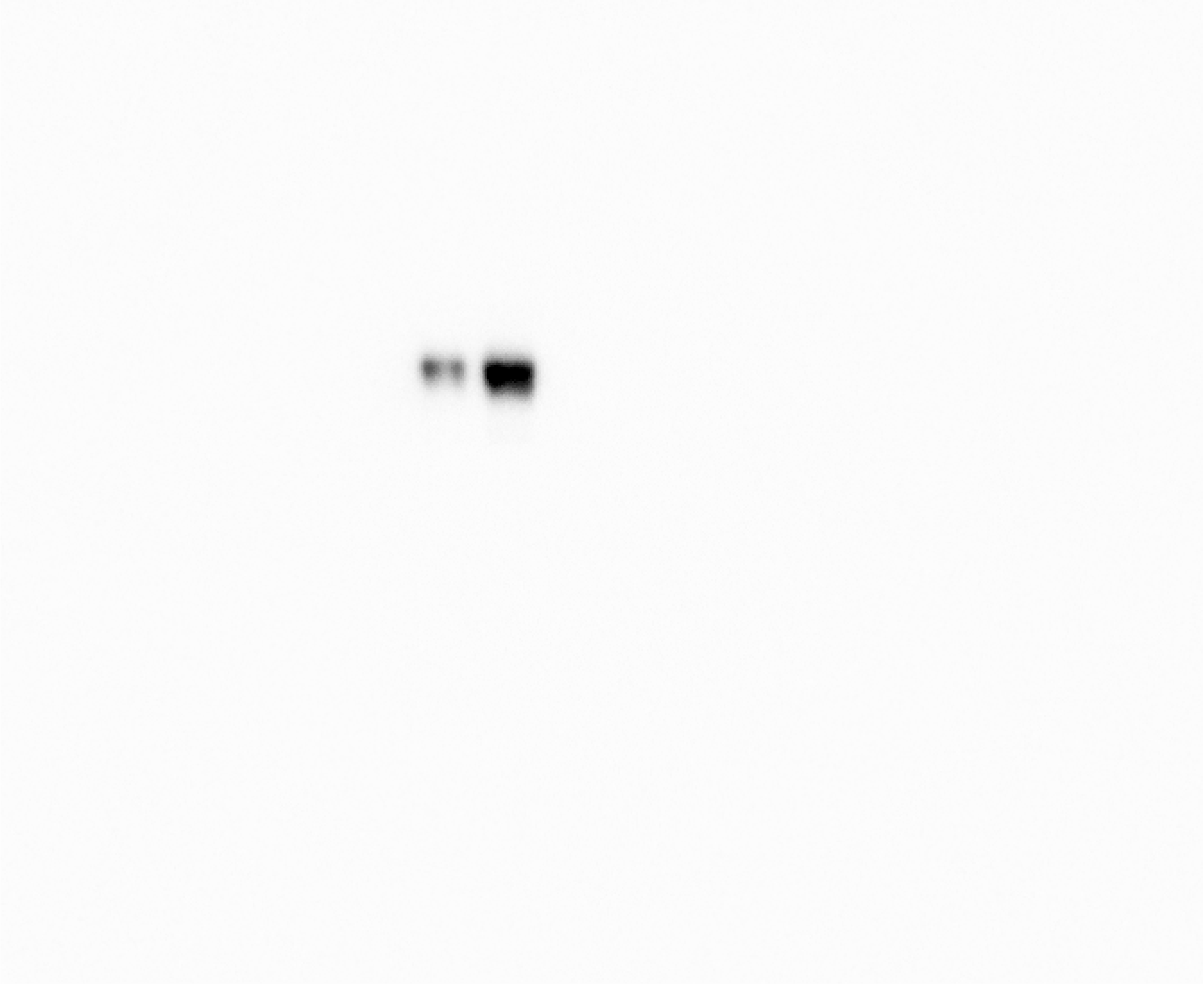

Figure 4E IP MAT2A IgT

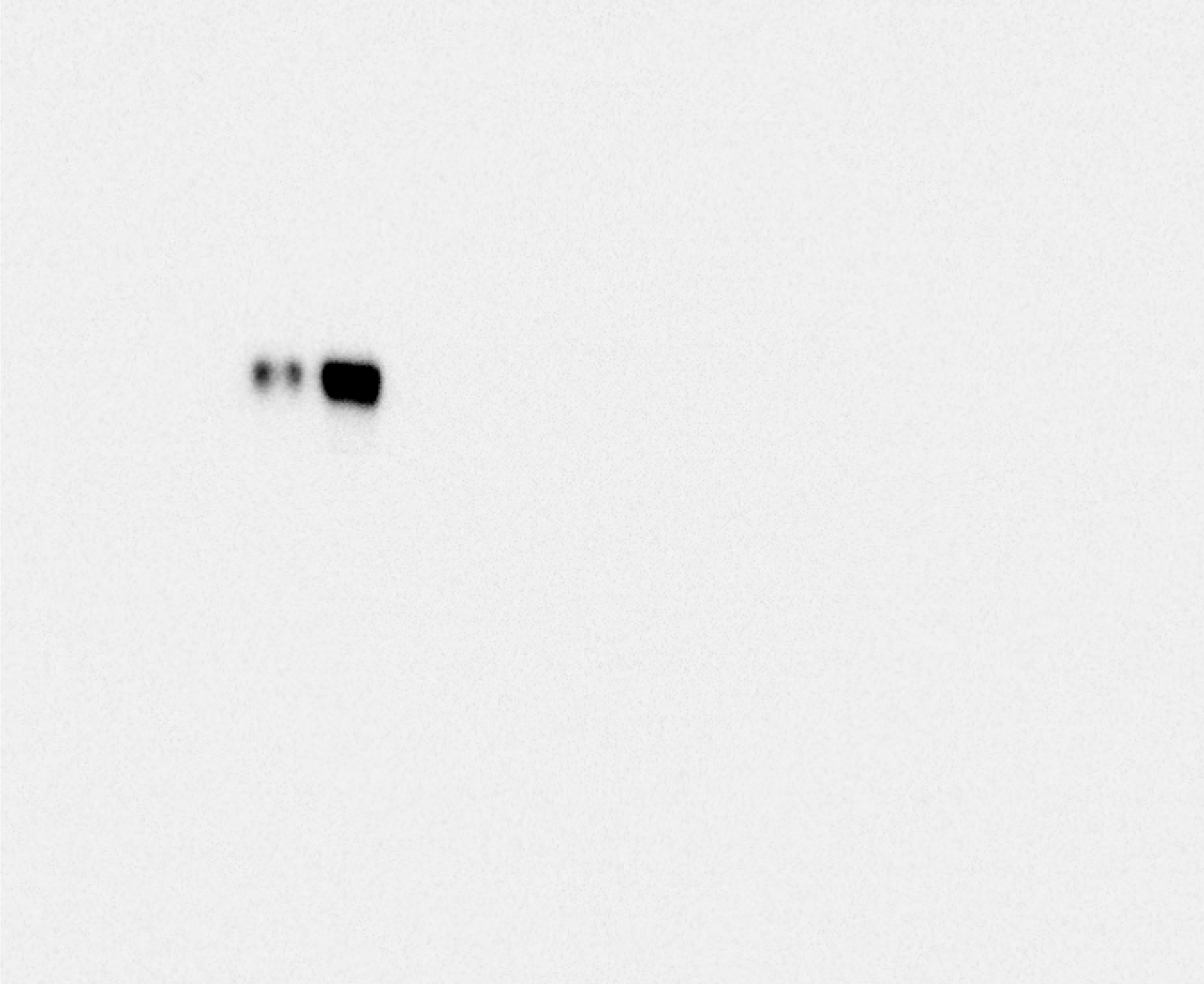

Figure 4E IP TRIM25 IgM

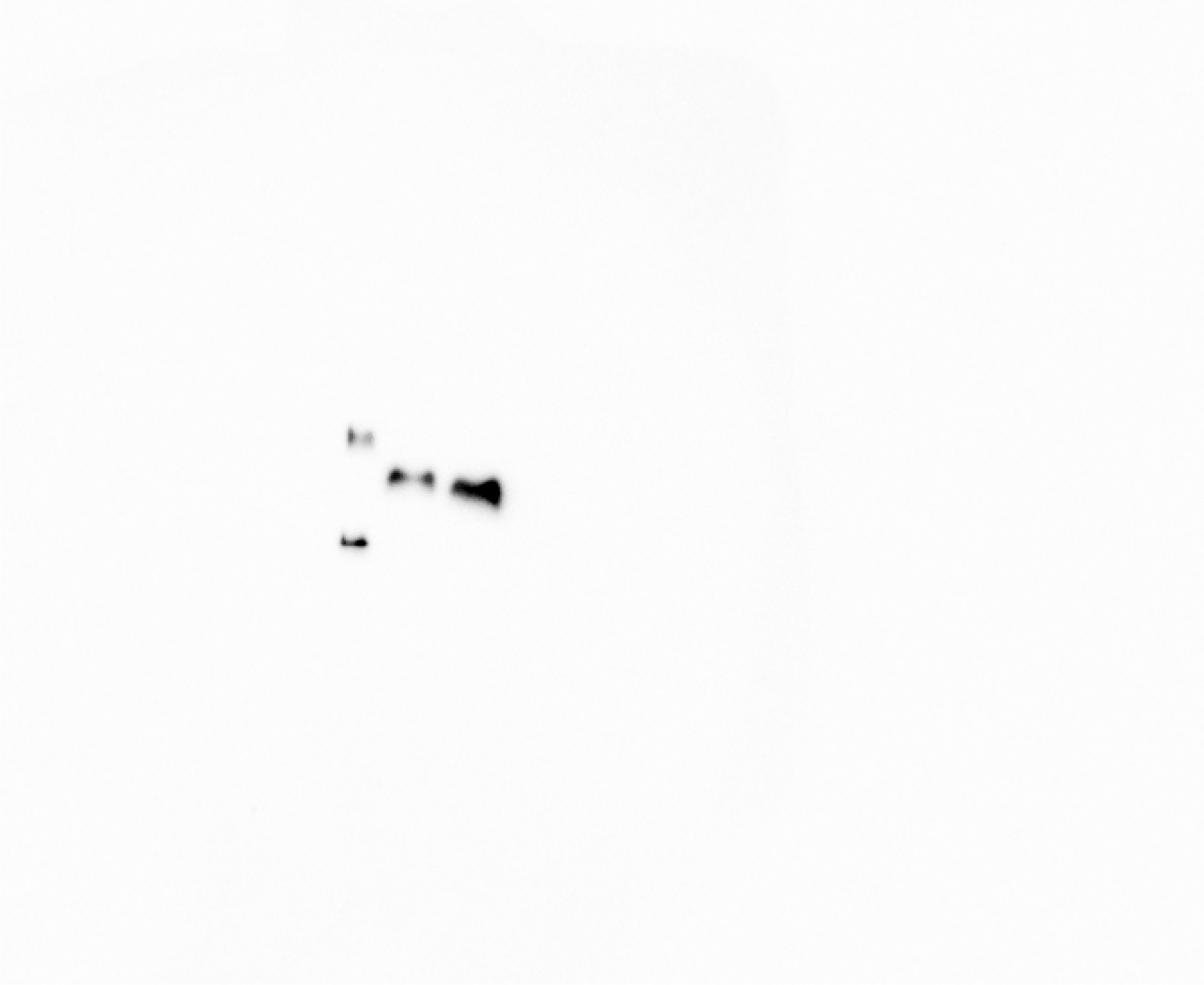

Figure 4G TRIM25 UMUC-3

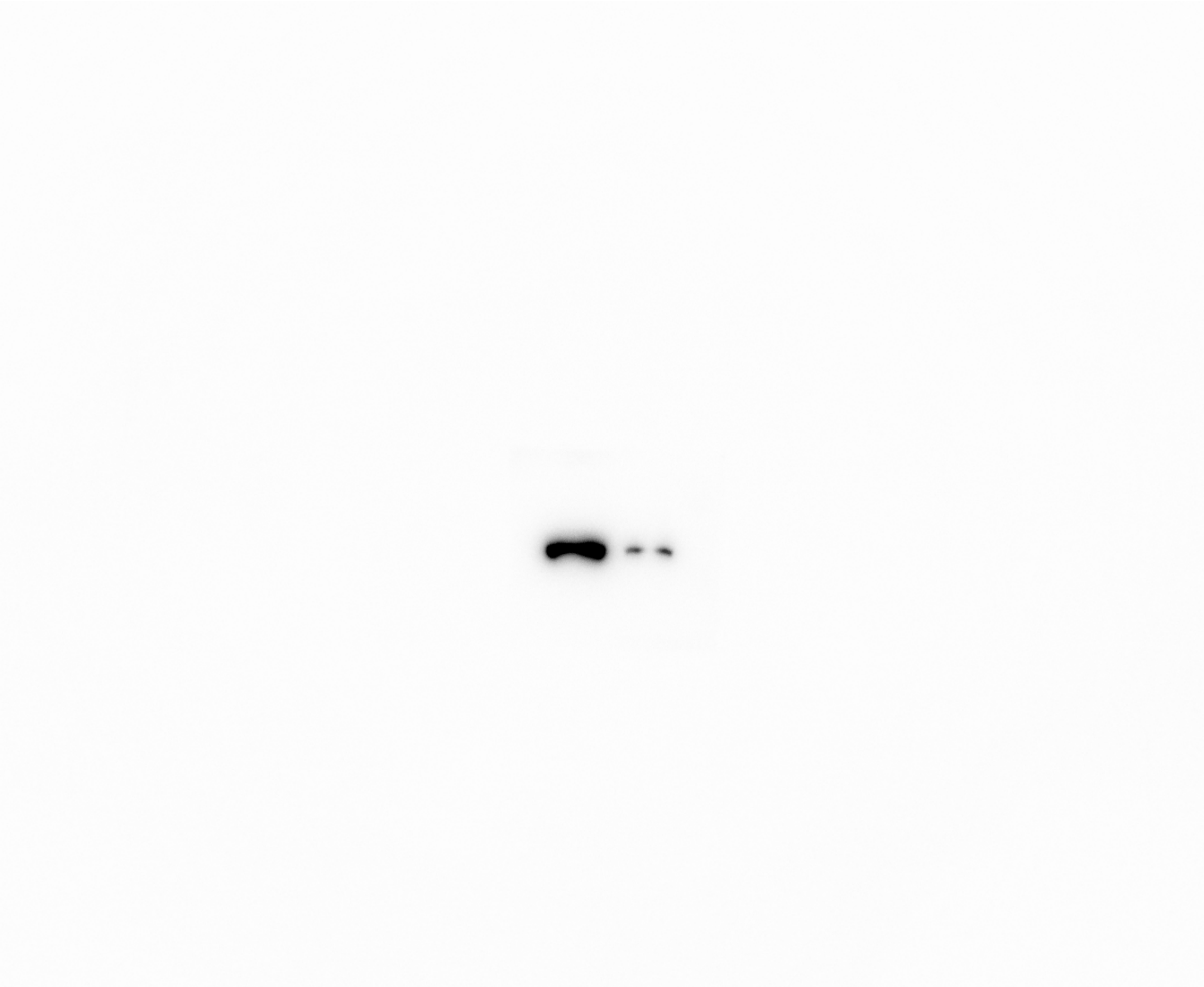

Figure 4G GAPDH T24

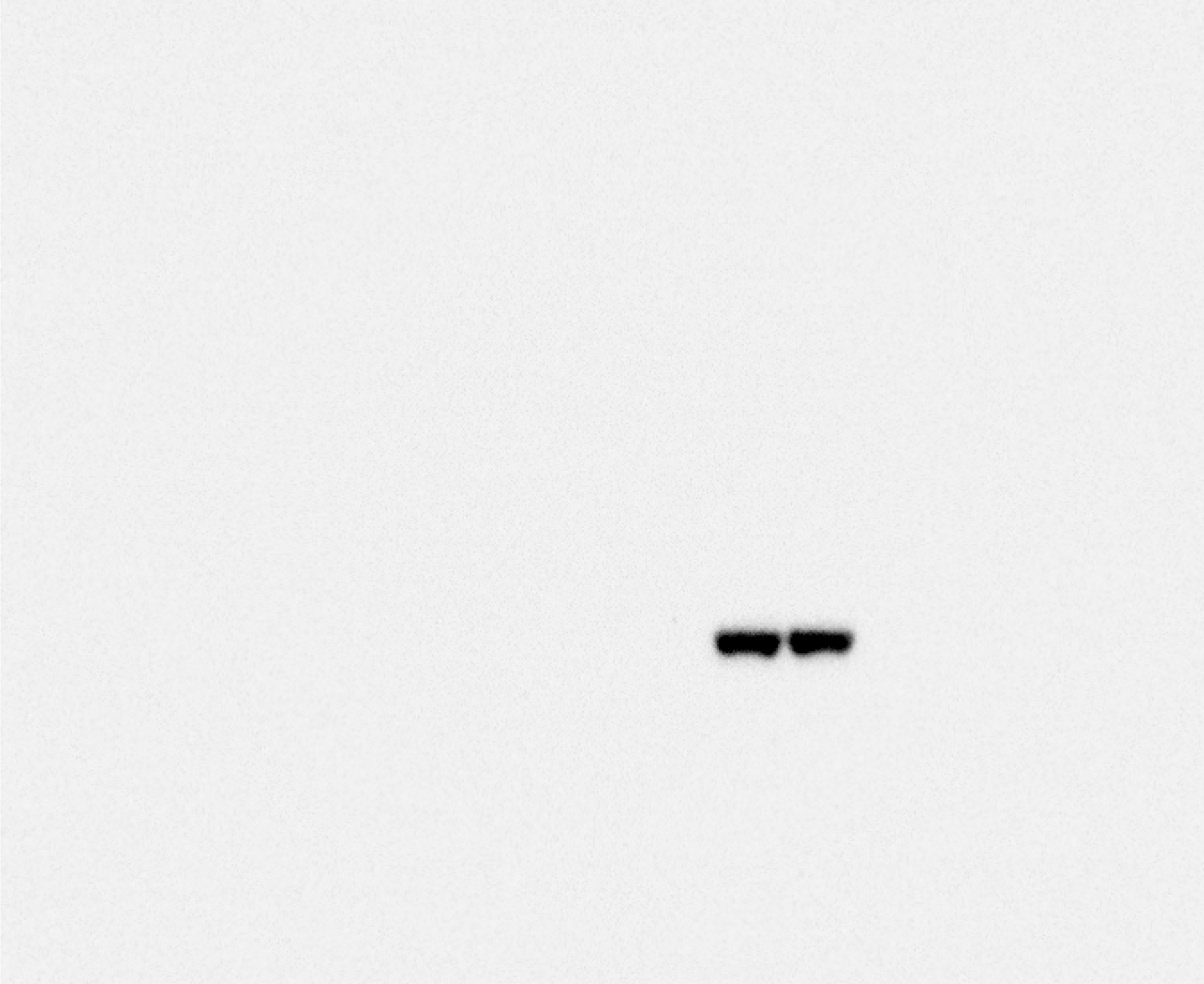

Figure 4G GAPDH UMUC-3

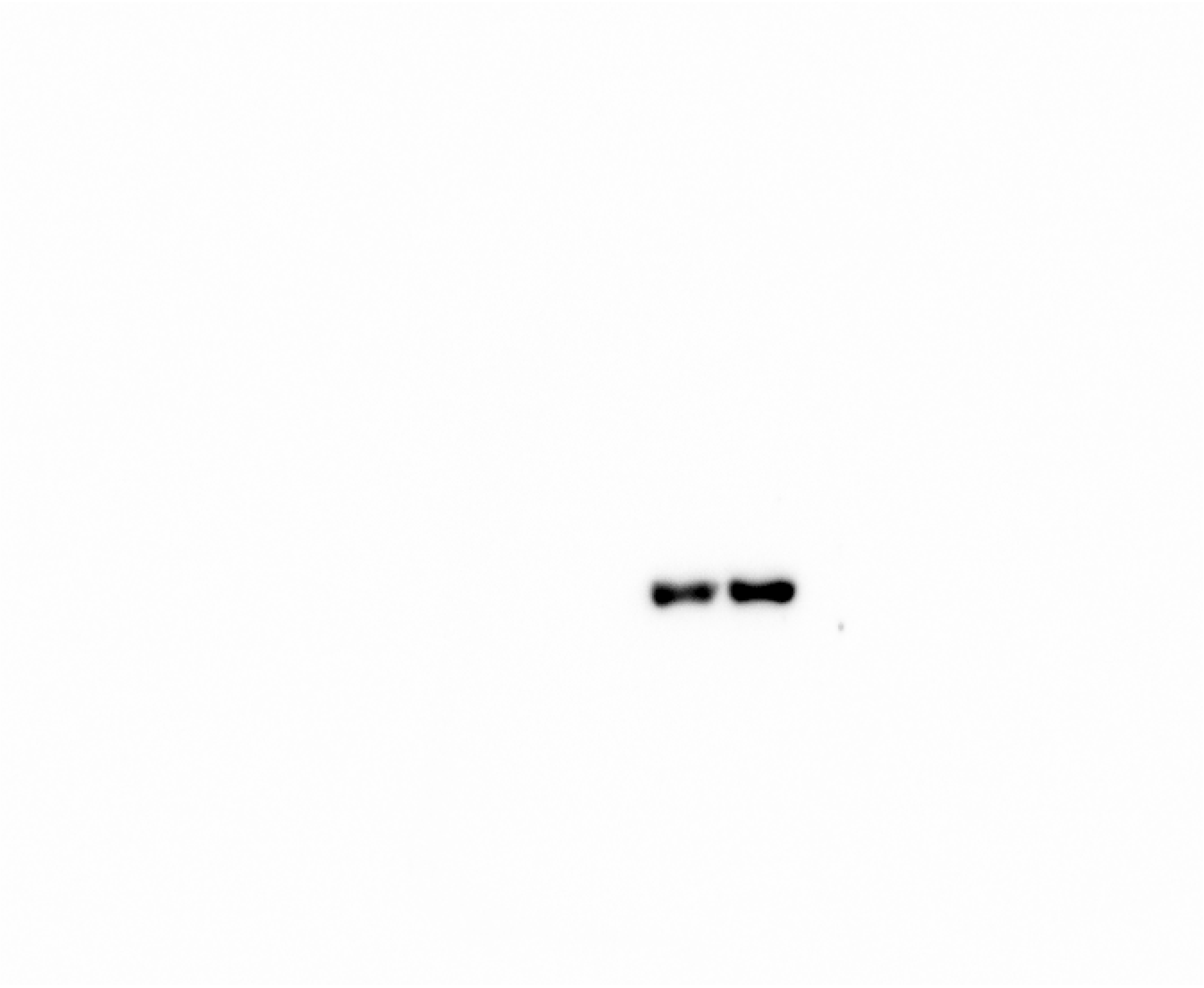

Figure 4G MAT2A T24

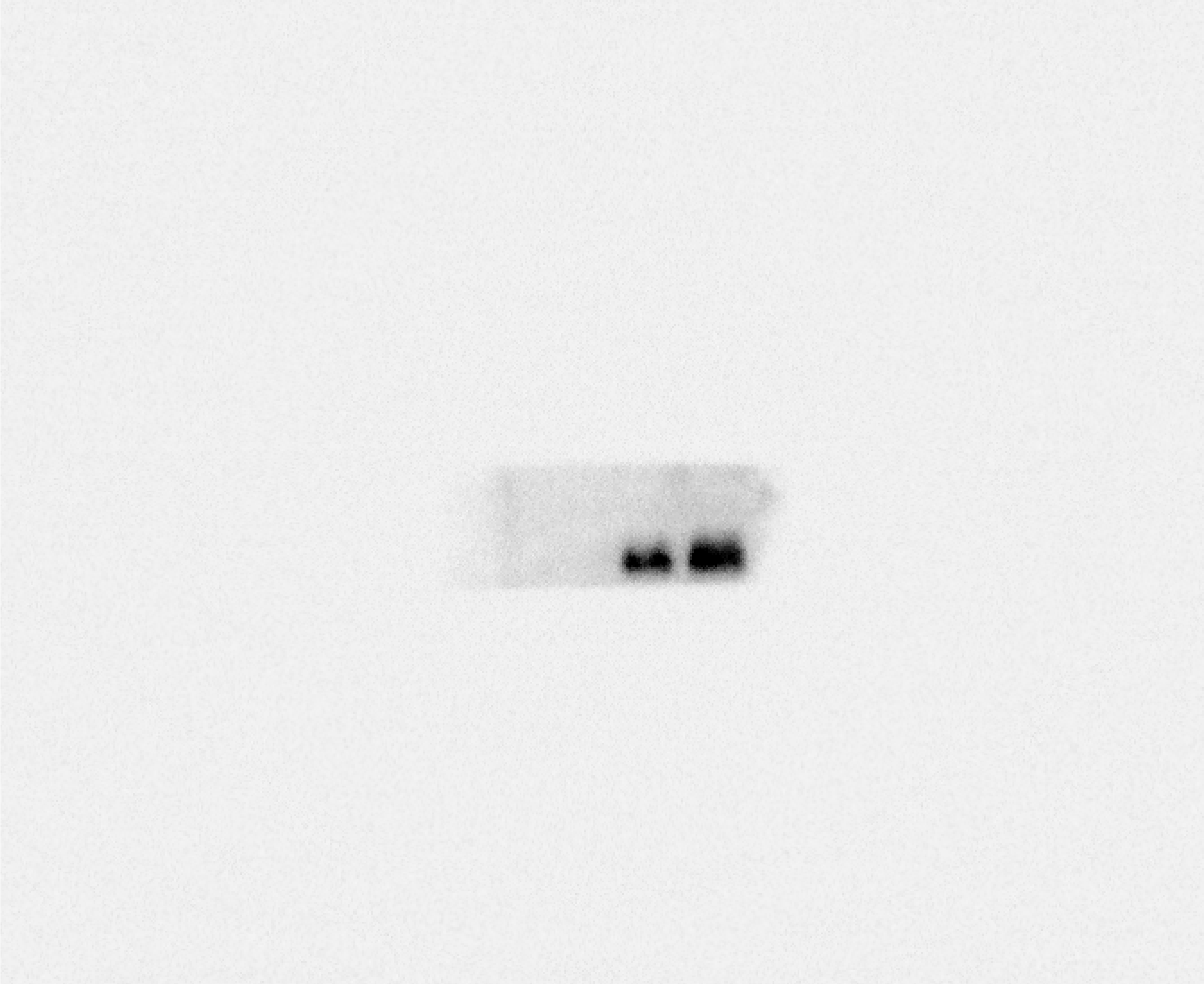

Figure 4G MAT2A UMUC-3

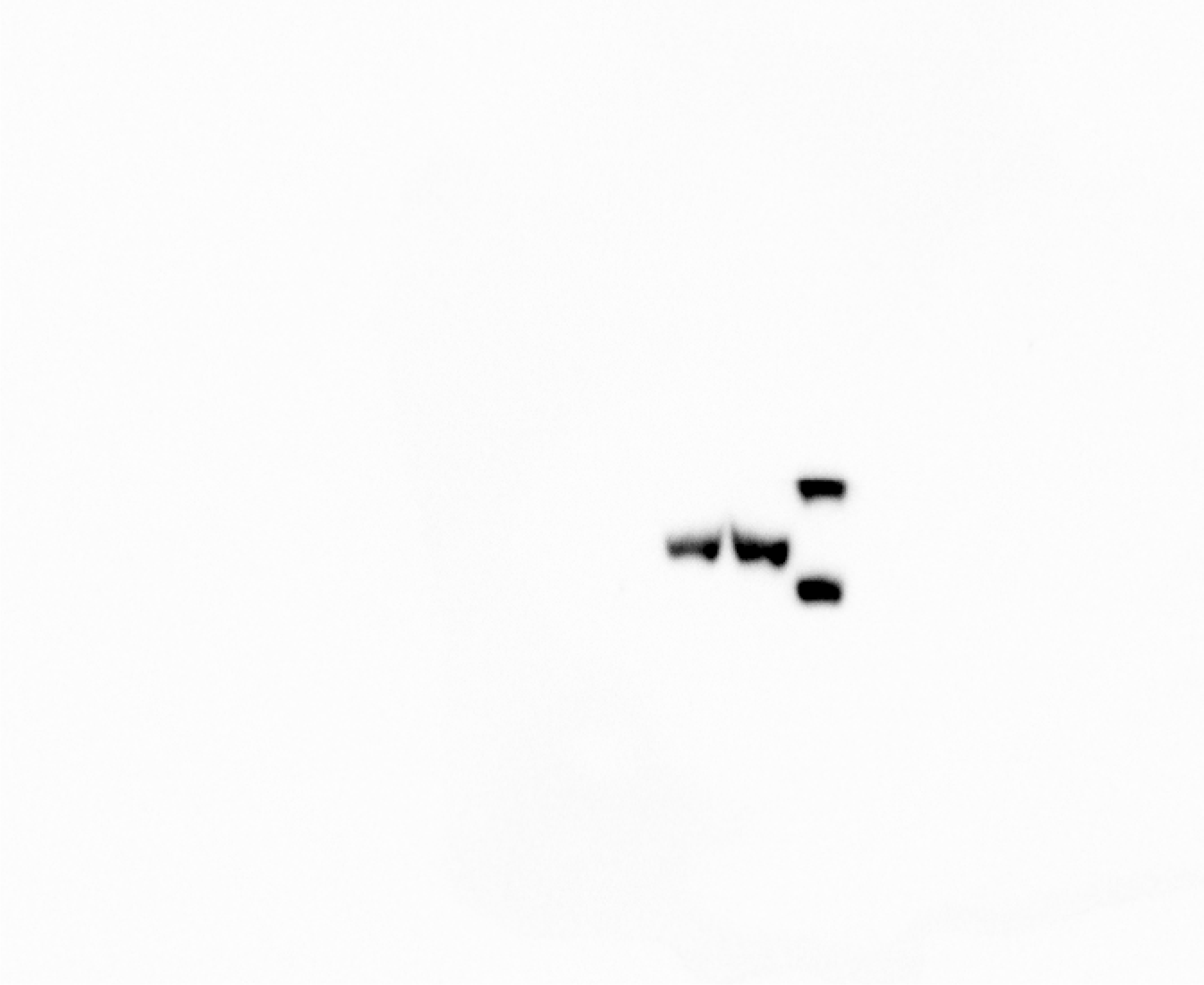

Figure 4G TRIM25 T24

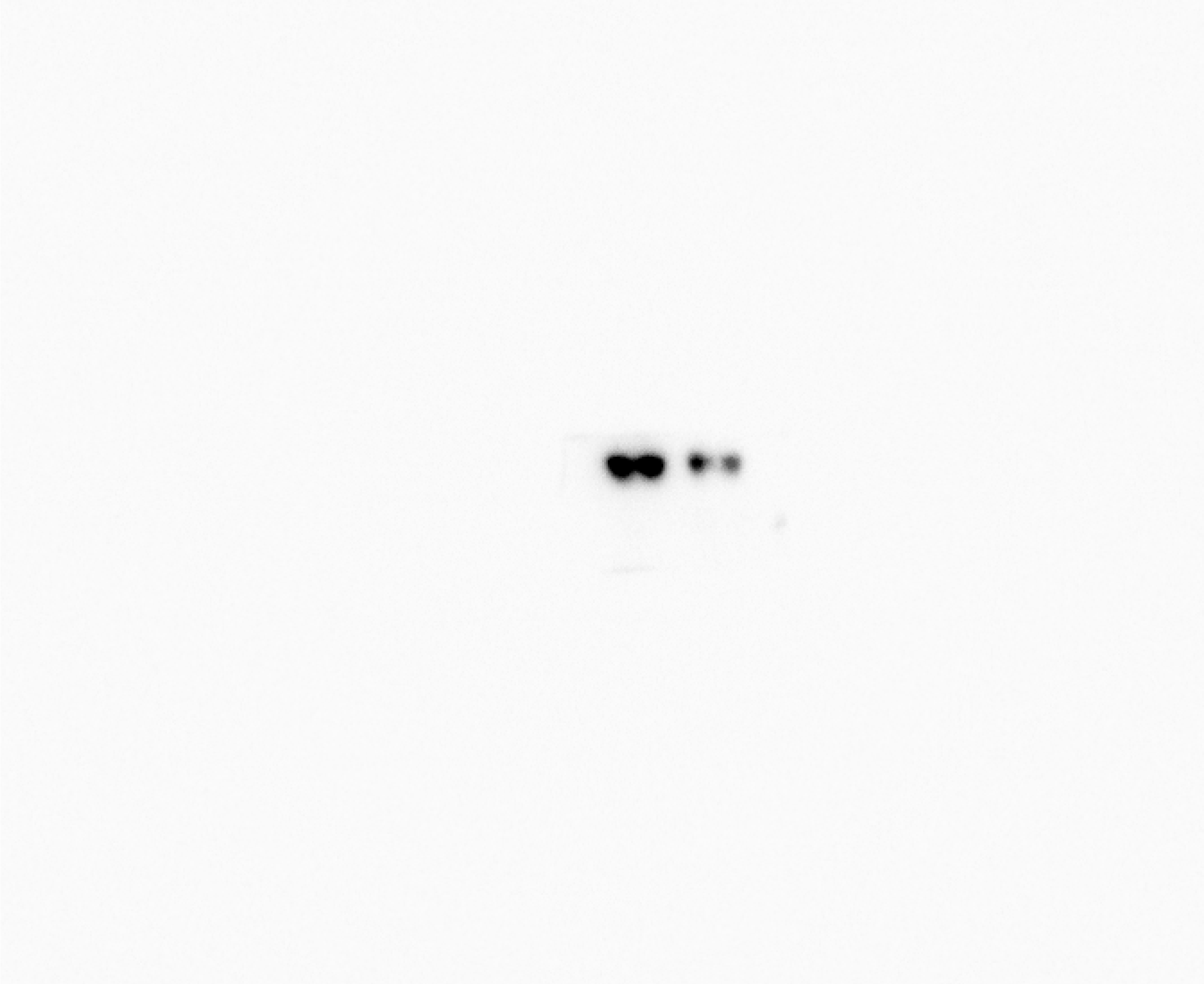

Figure 4H MAT2A UMUC-3

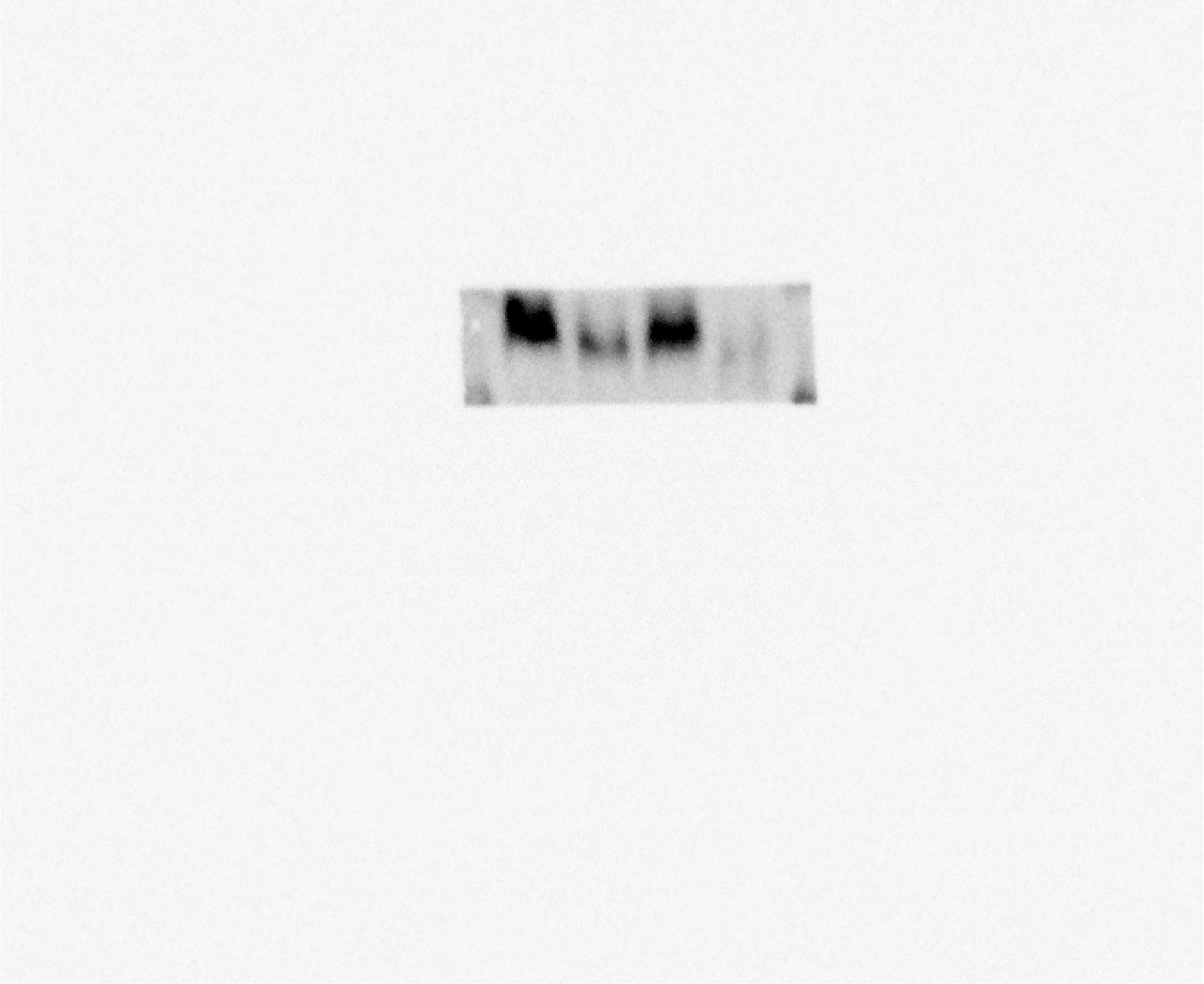

Figure 4H Flag T24

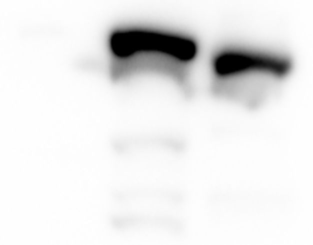

Figure 4H Flag UMUC-3

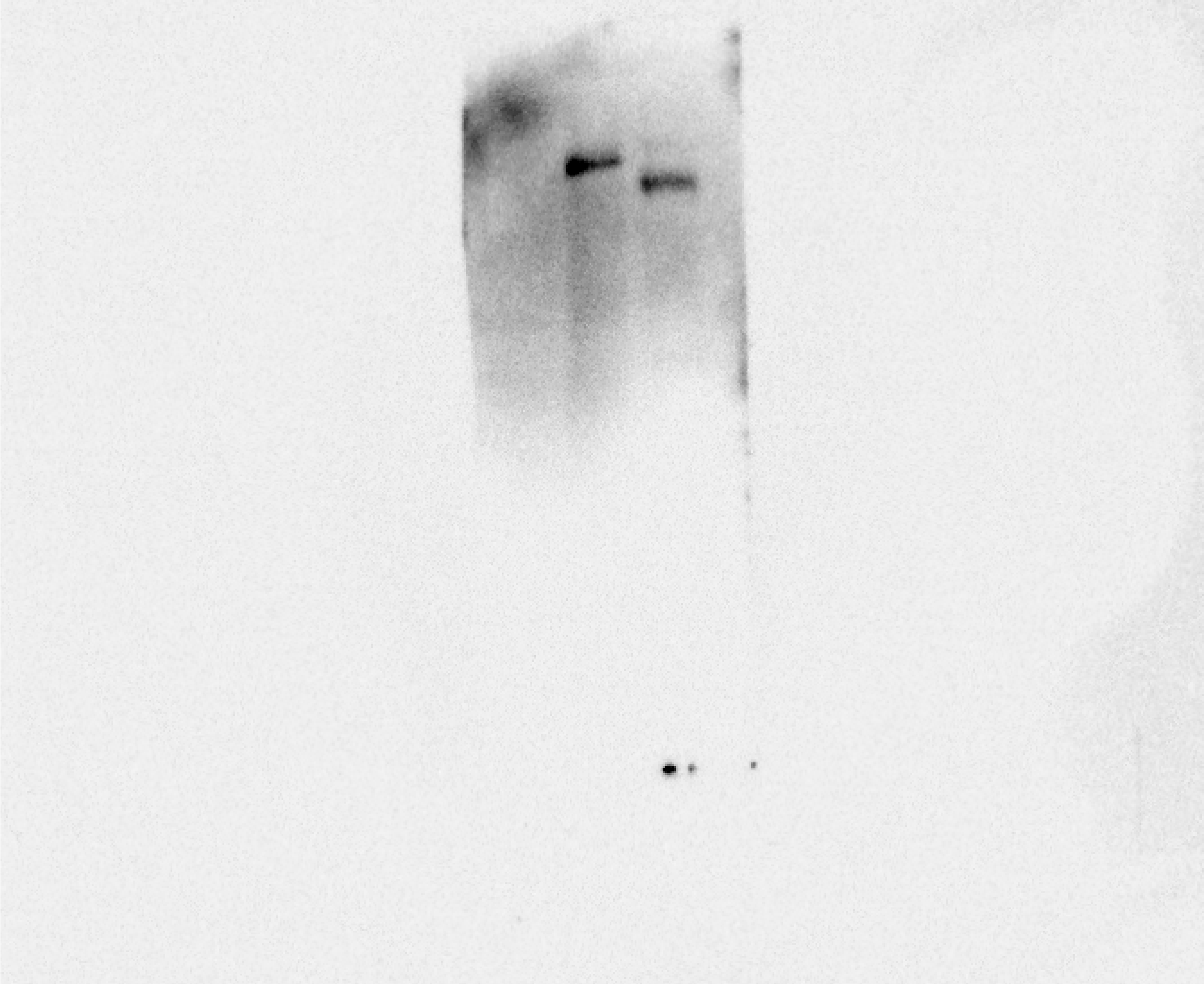

Figure 4H GAPDH T24

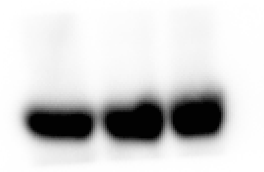

Figure 4H GAPDH UMUC-3

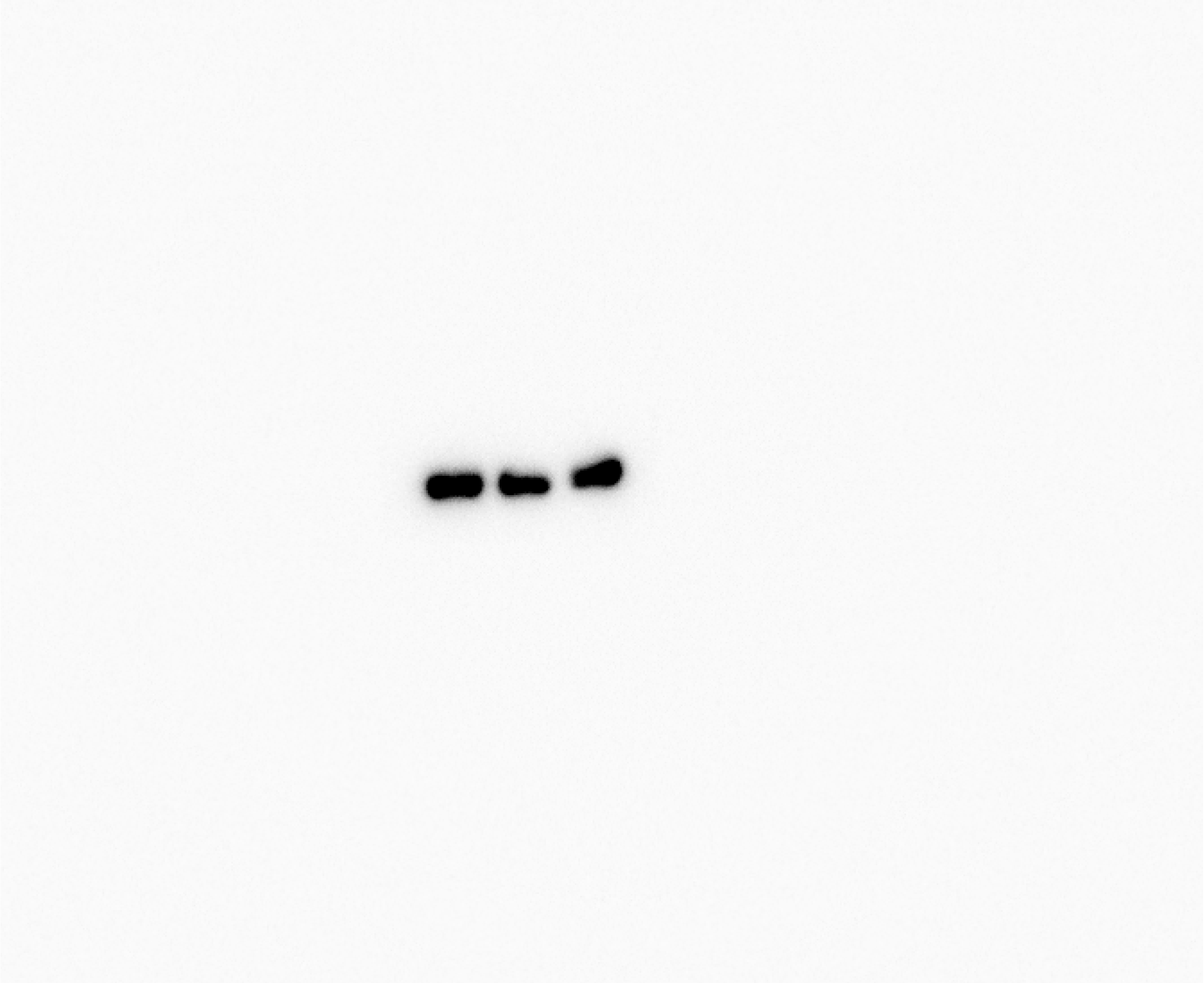

Figure 4H MAT2A T24

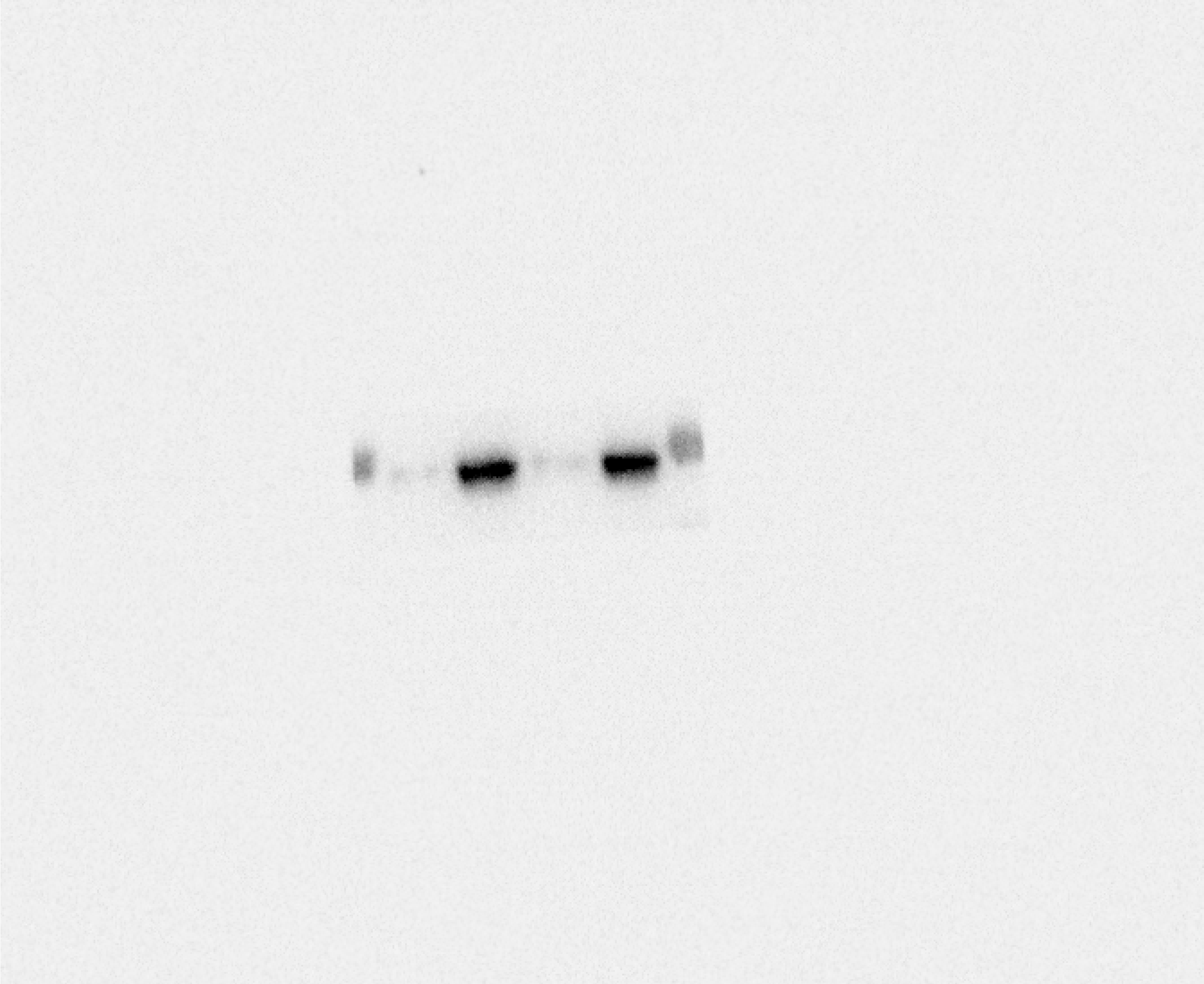

Figure 4I MAT2A Input UMUC-3

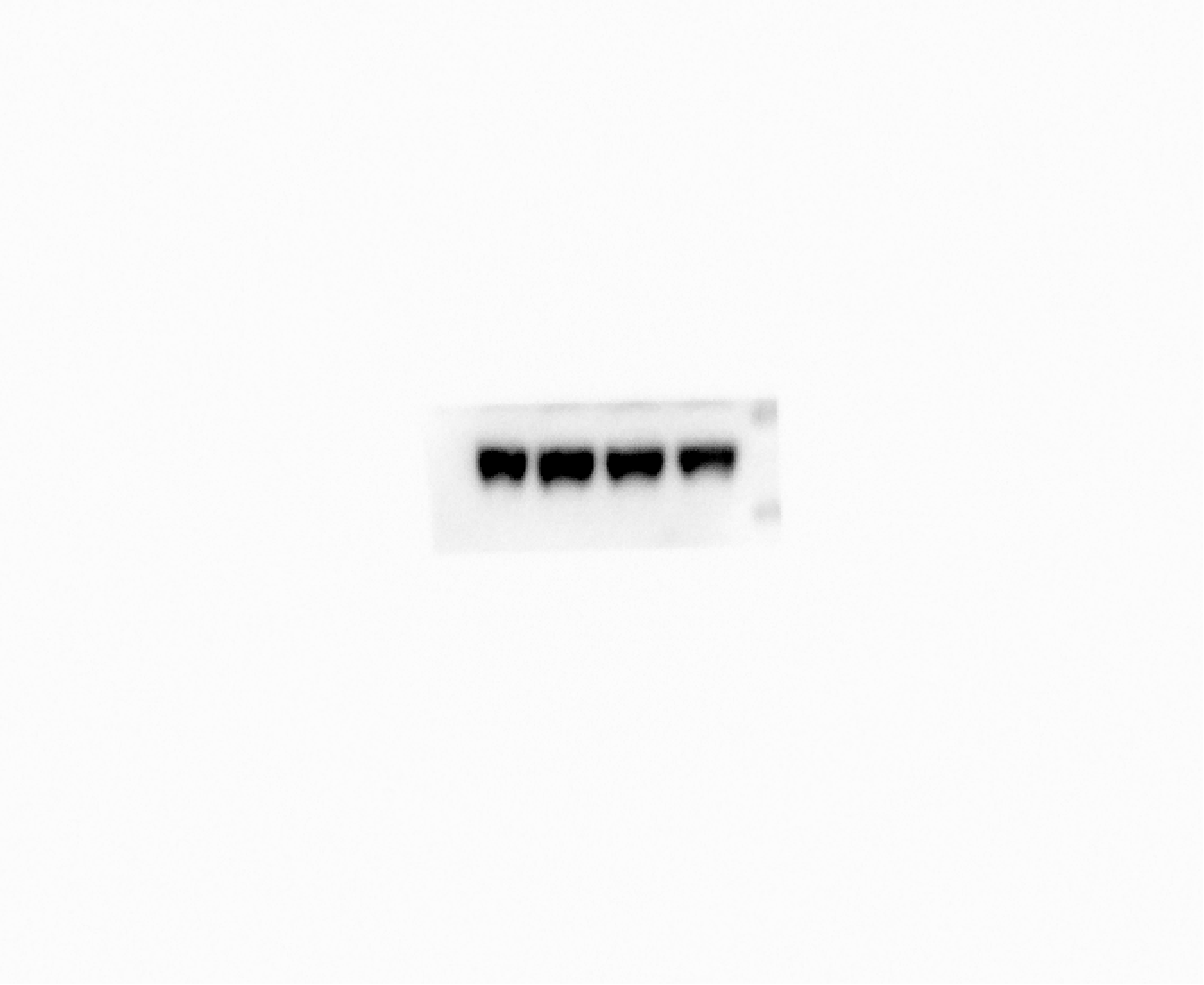

Figure 4I IP HA T24

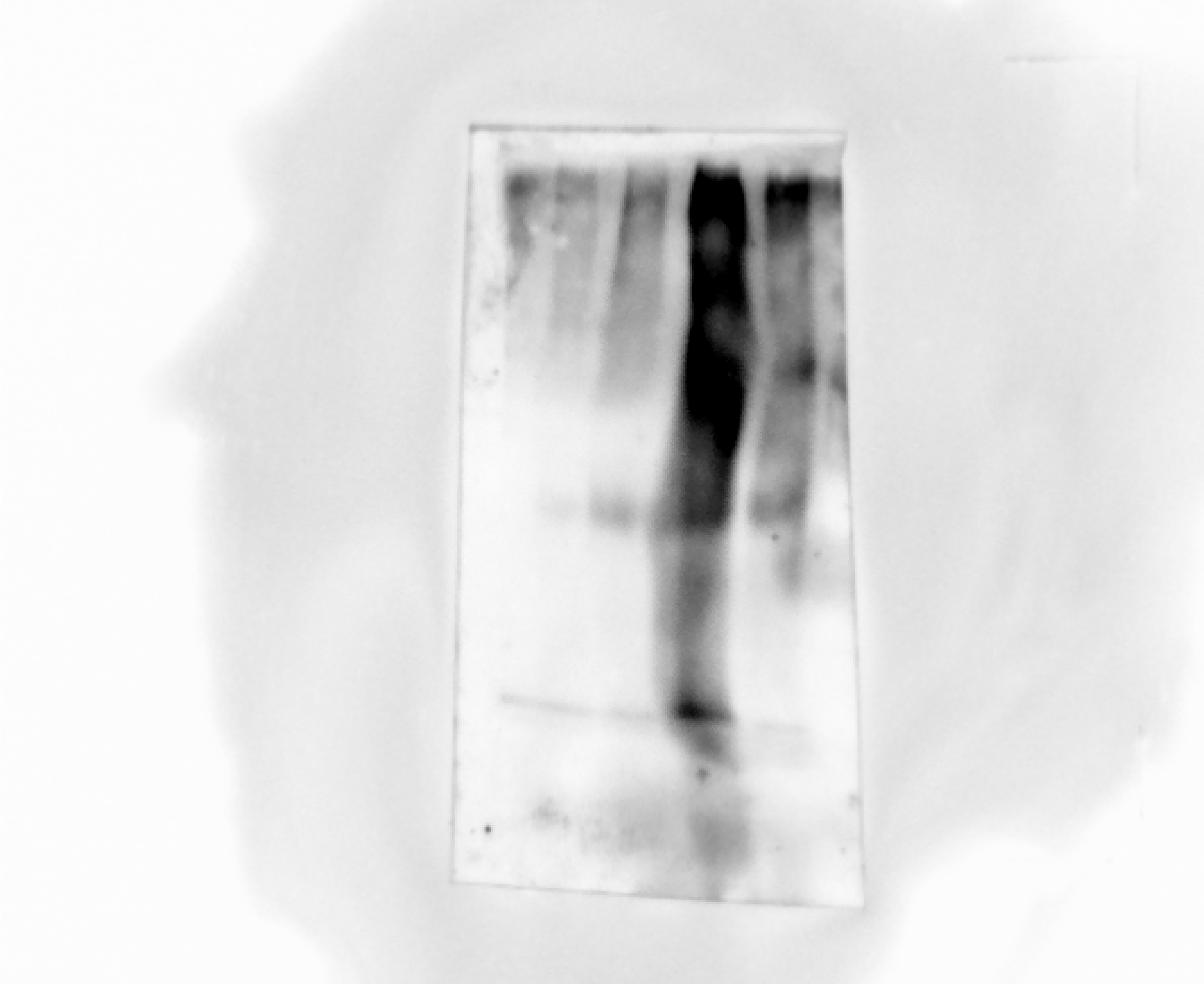

Figure 4I IP HA UMUC-3

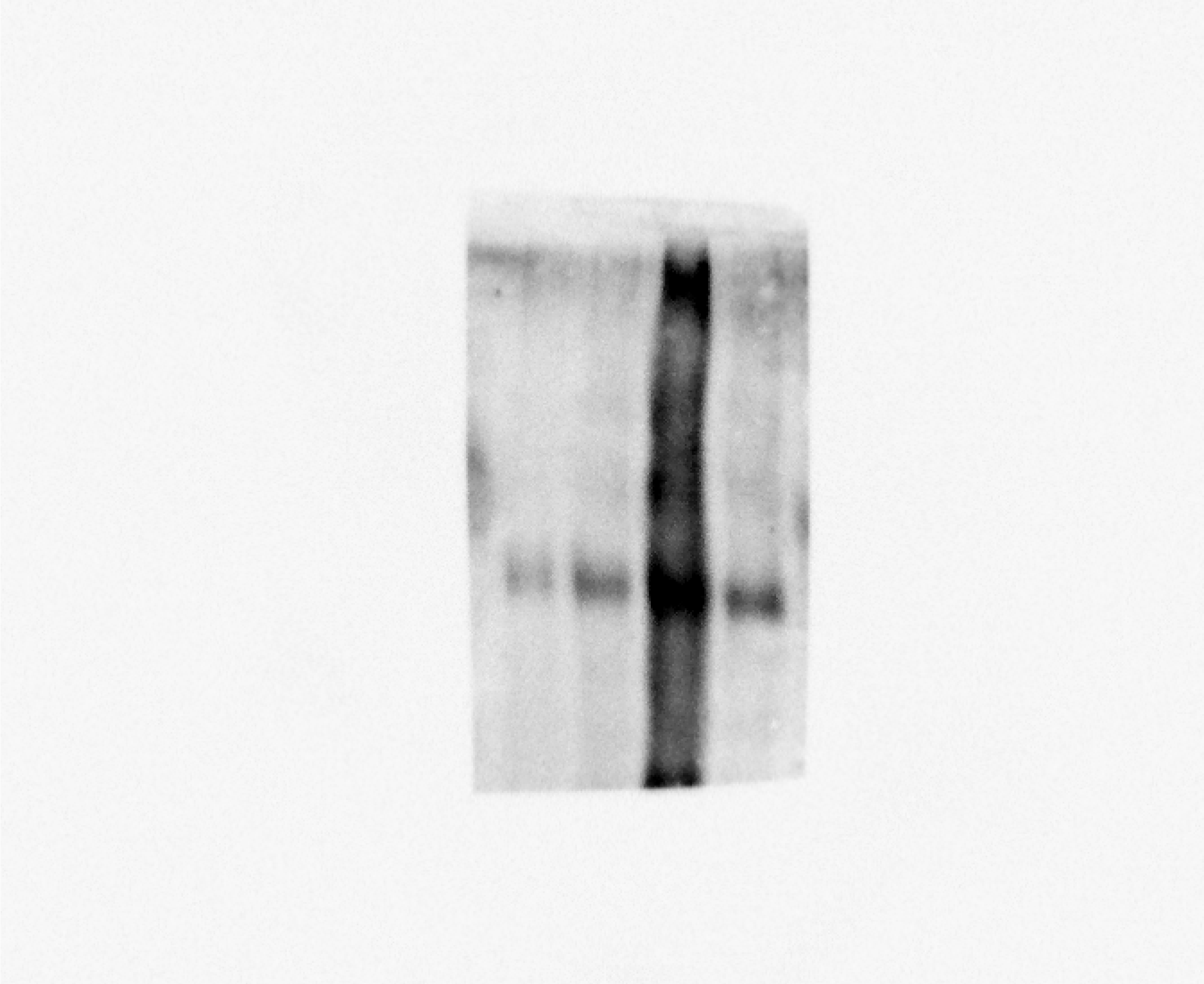

Figure 4I MAT2A Input T24

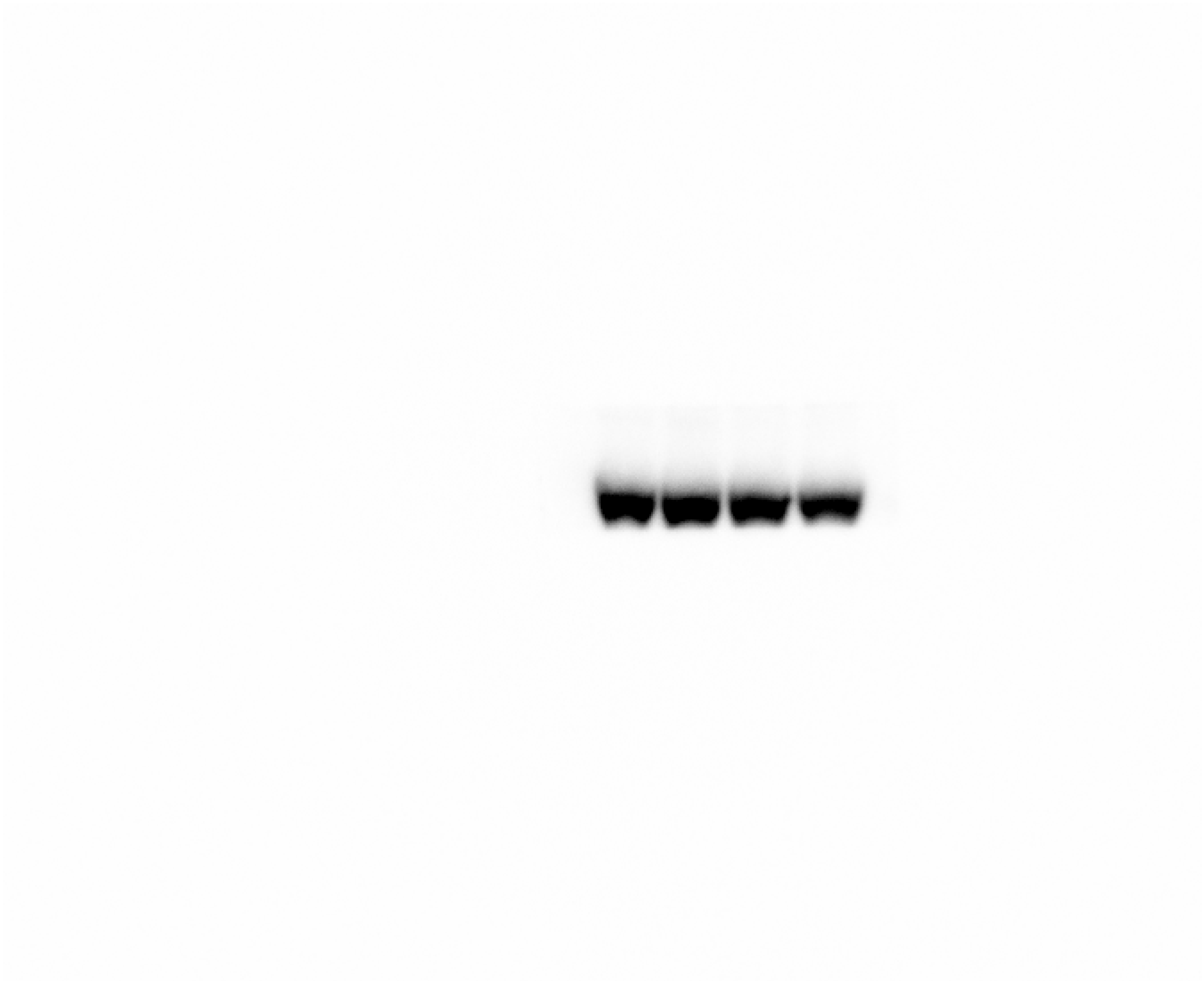

Figure 4J Ip Myc

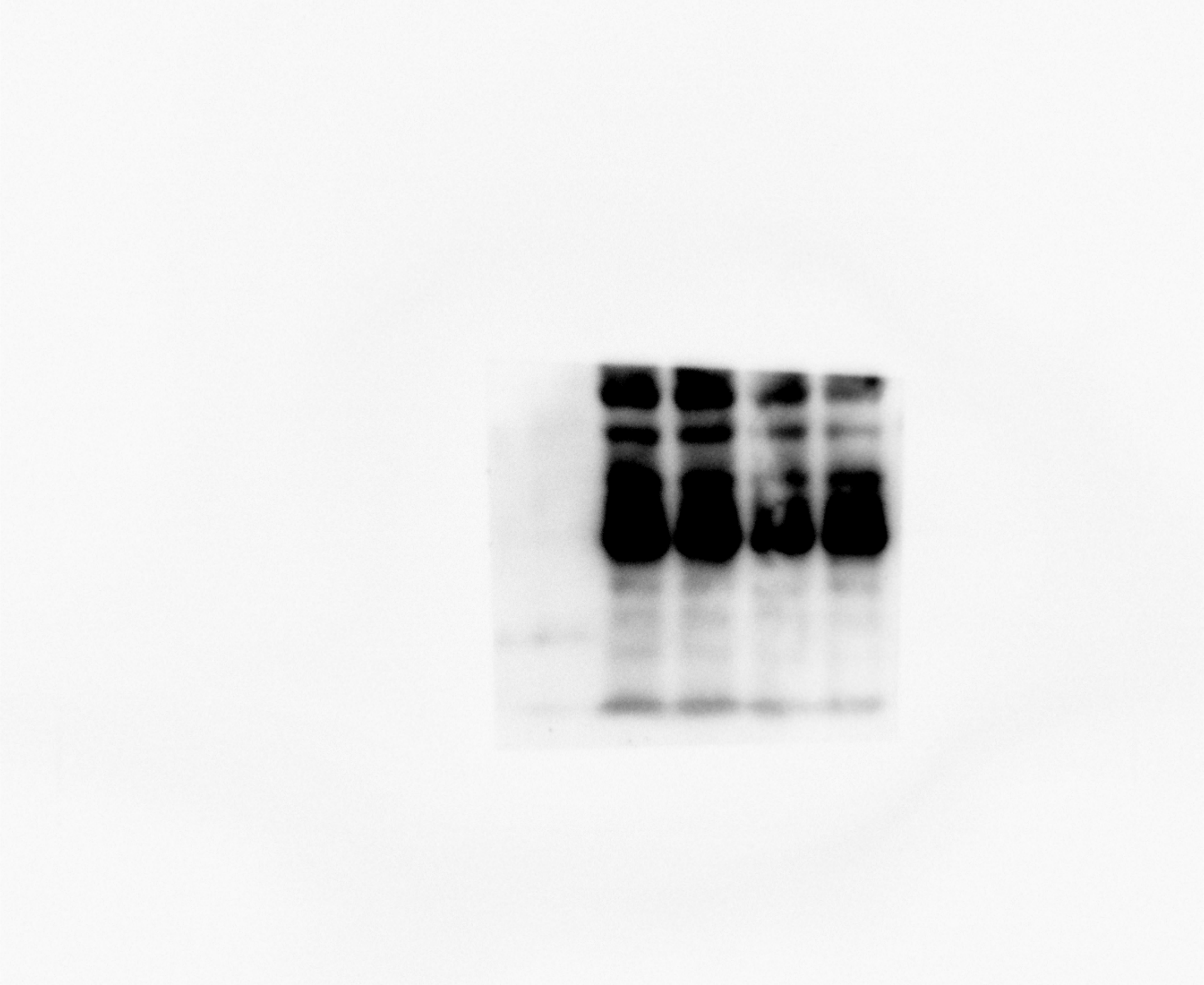

Figure 4J Input Flag

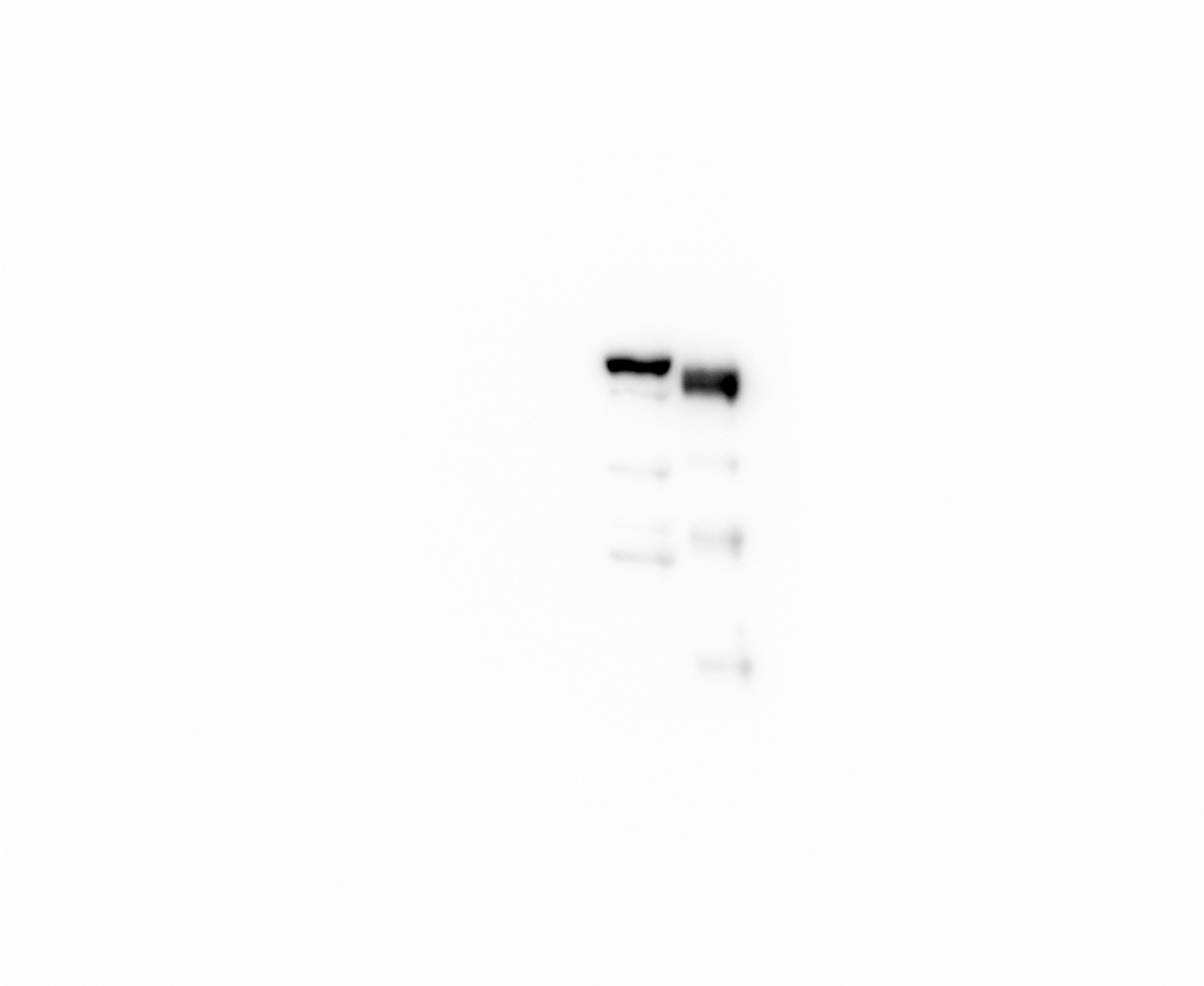

Figure 4J Input GAPDH

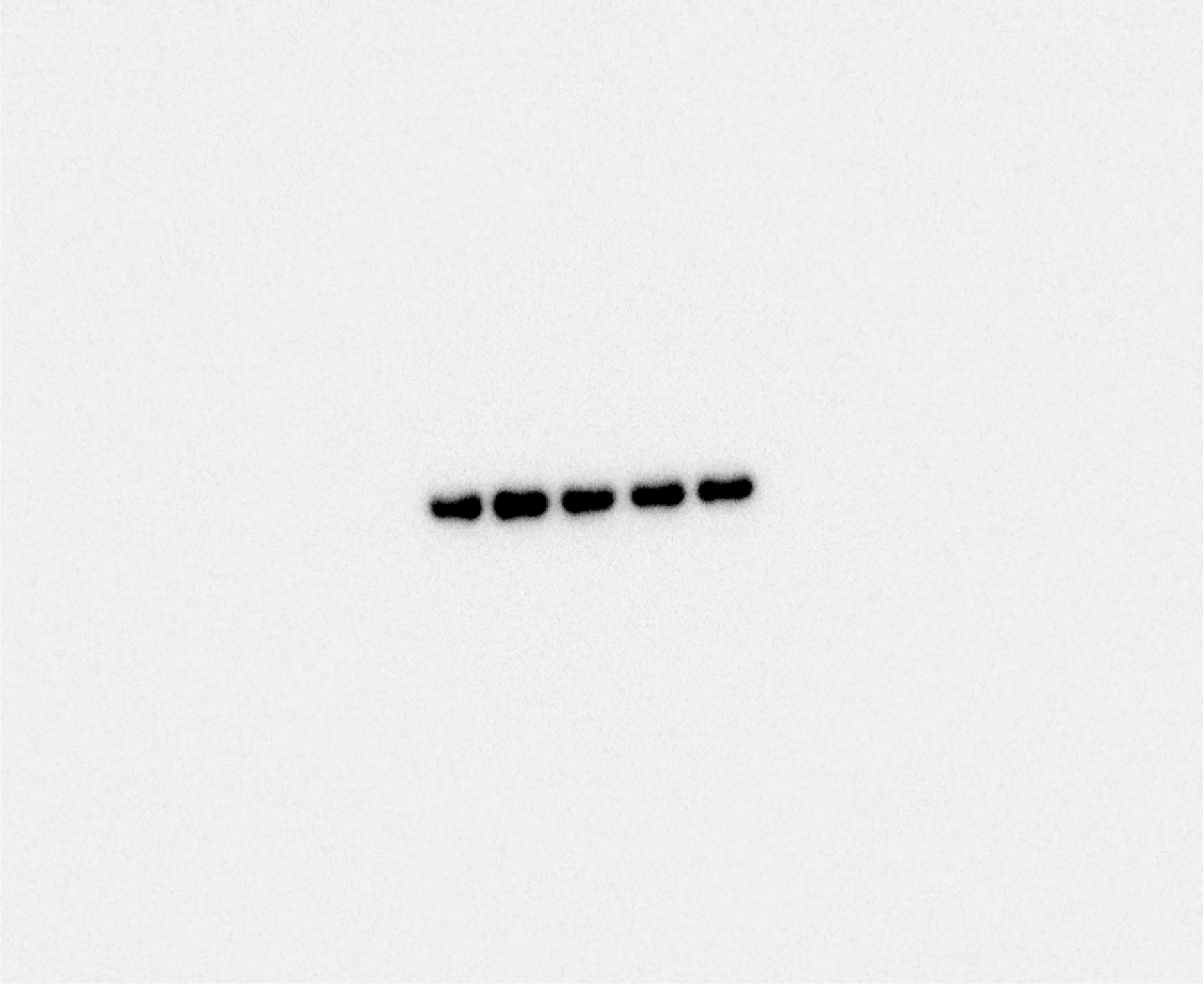

Figure 4J Input HA

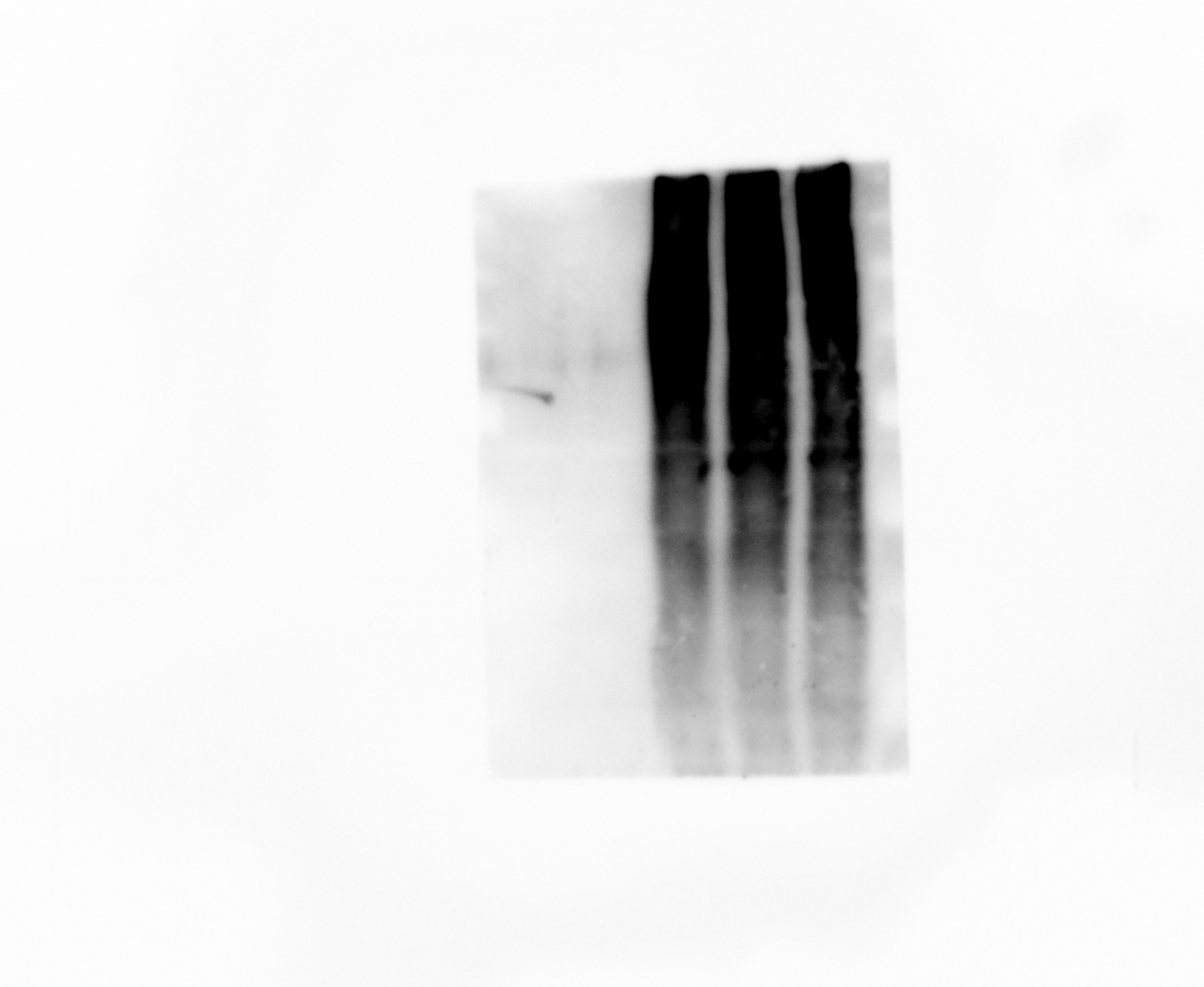

Figure 4J Ip HA

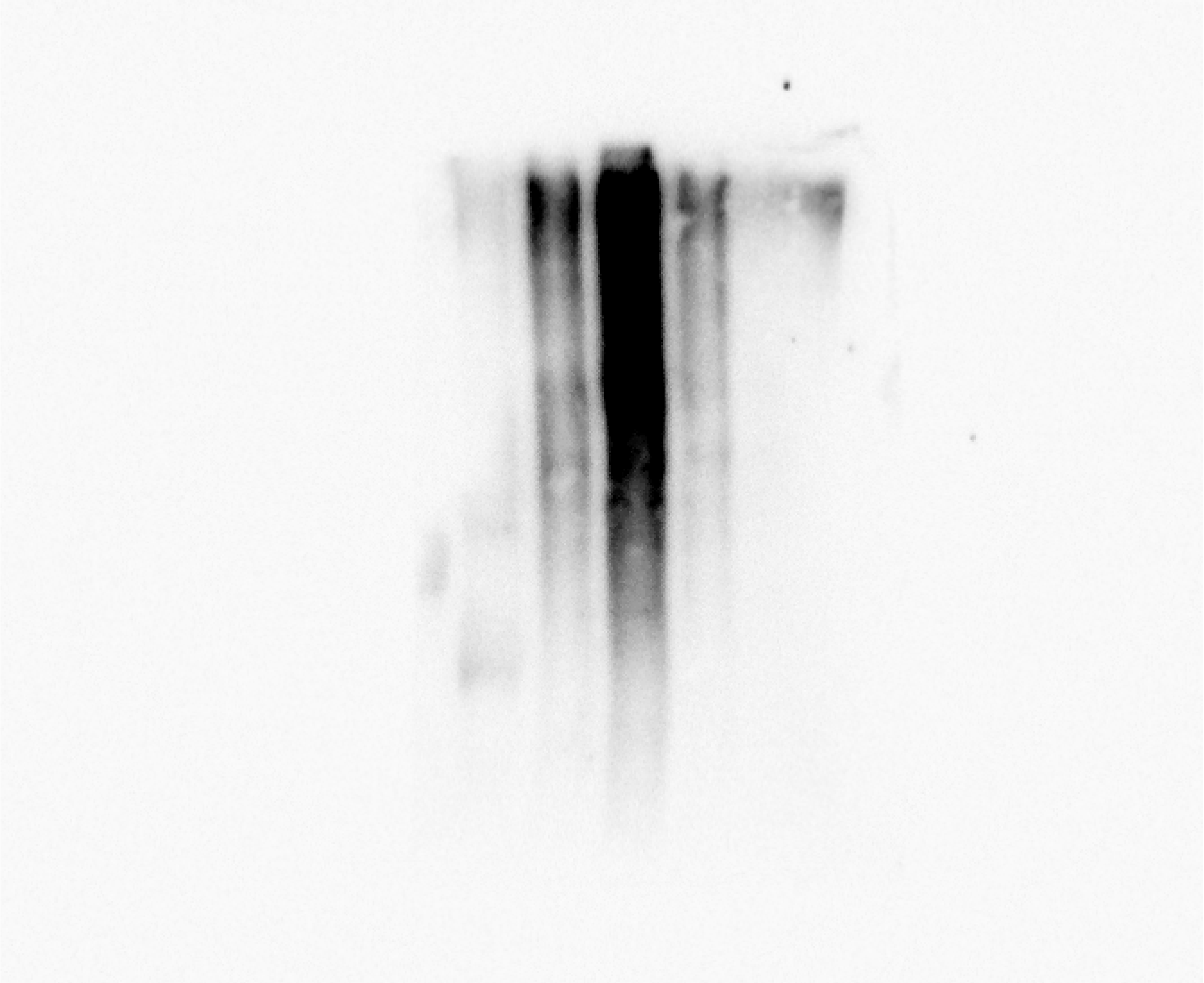

Figure 4K Myc

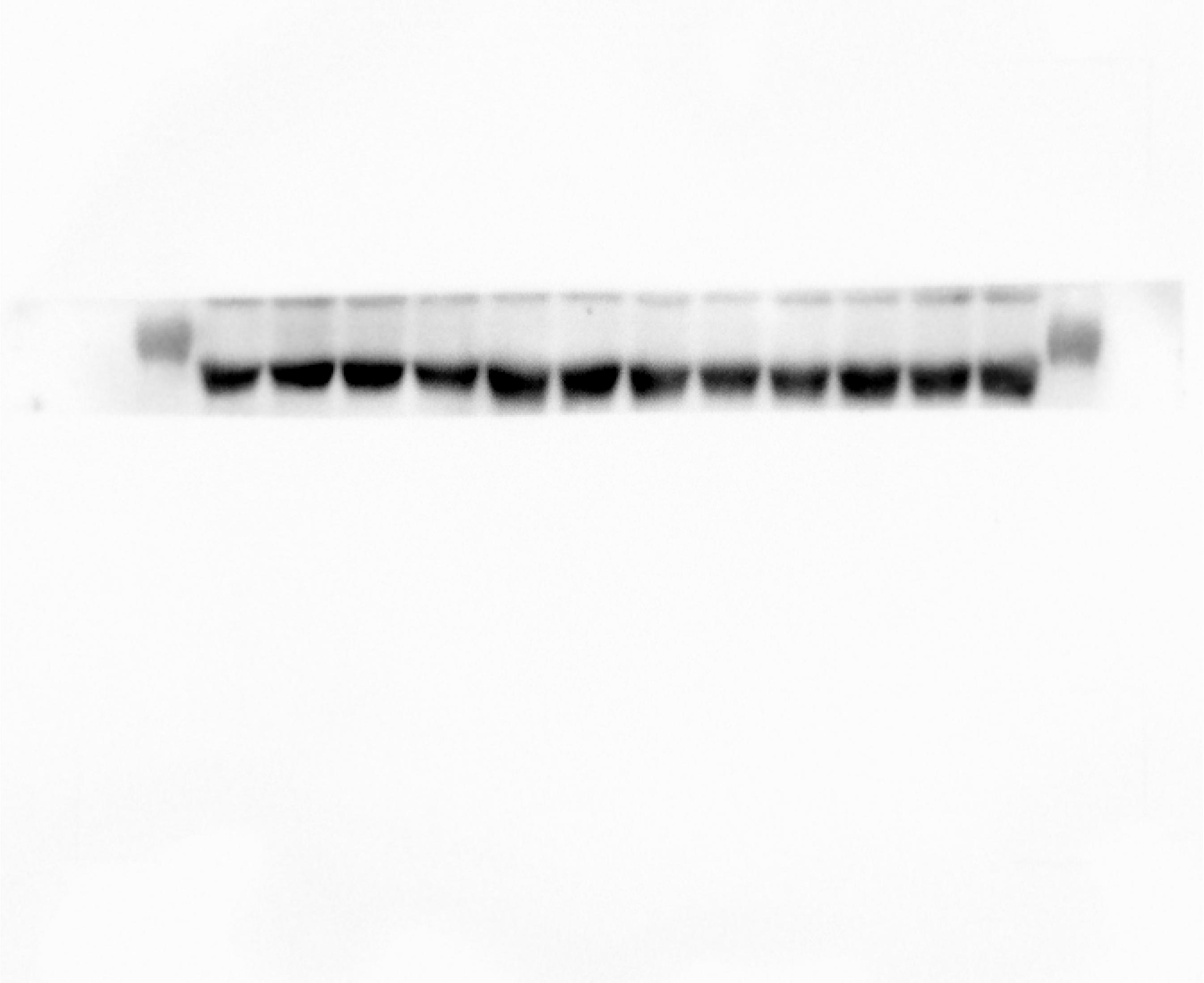

Figure 4K Flag

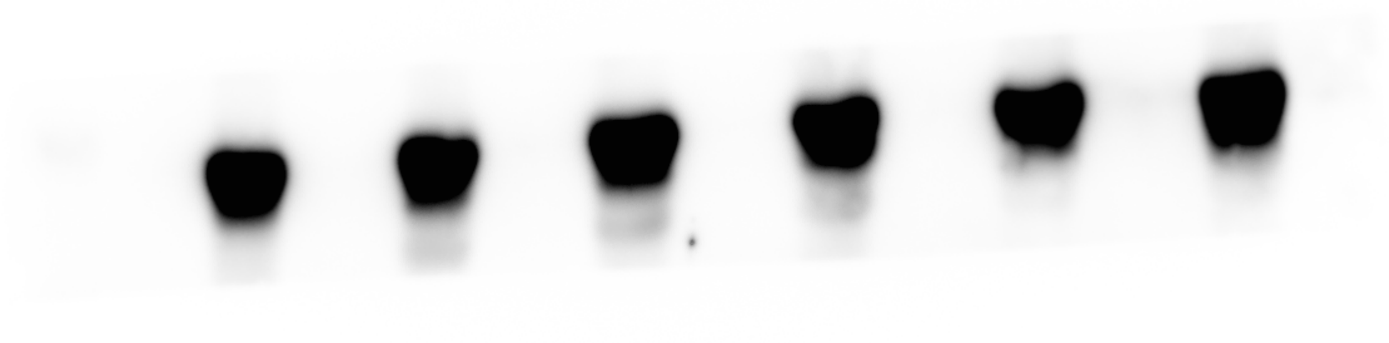

Figure 4K GAPDH

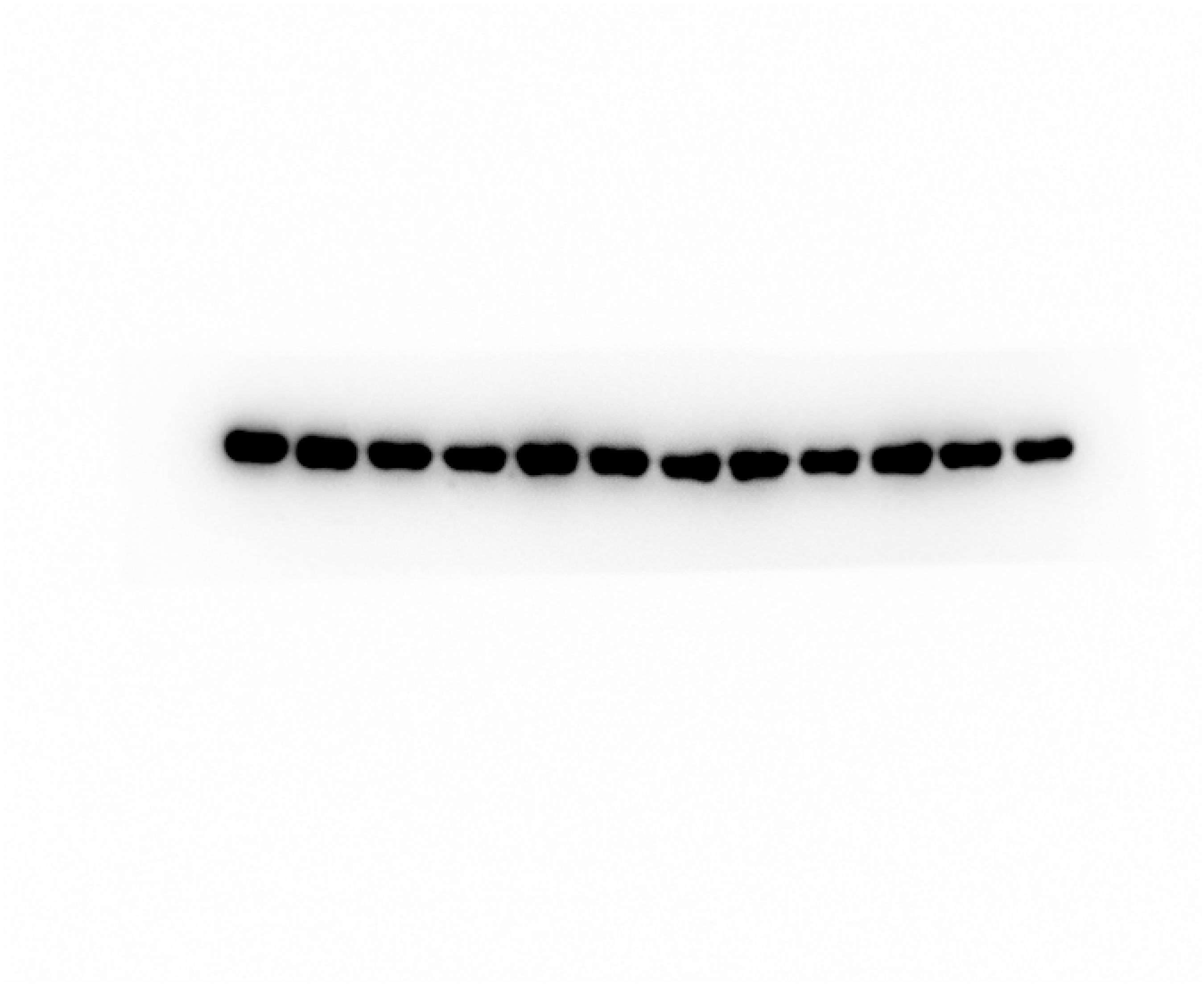

Figure 4K Input HA

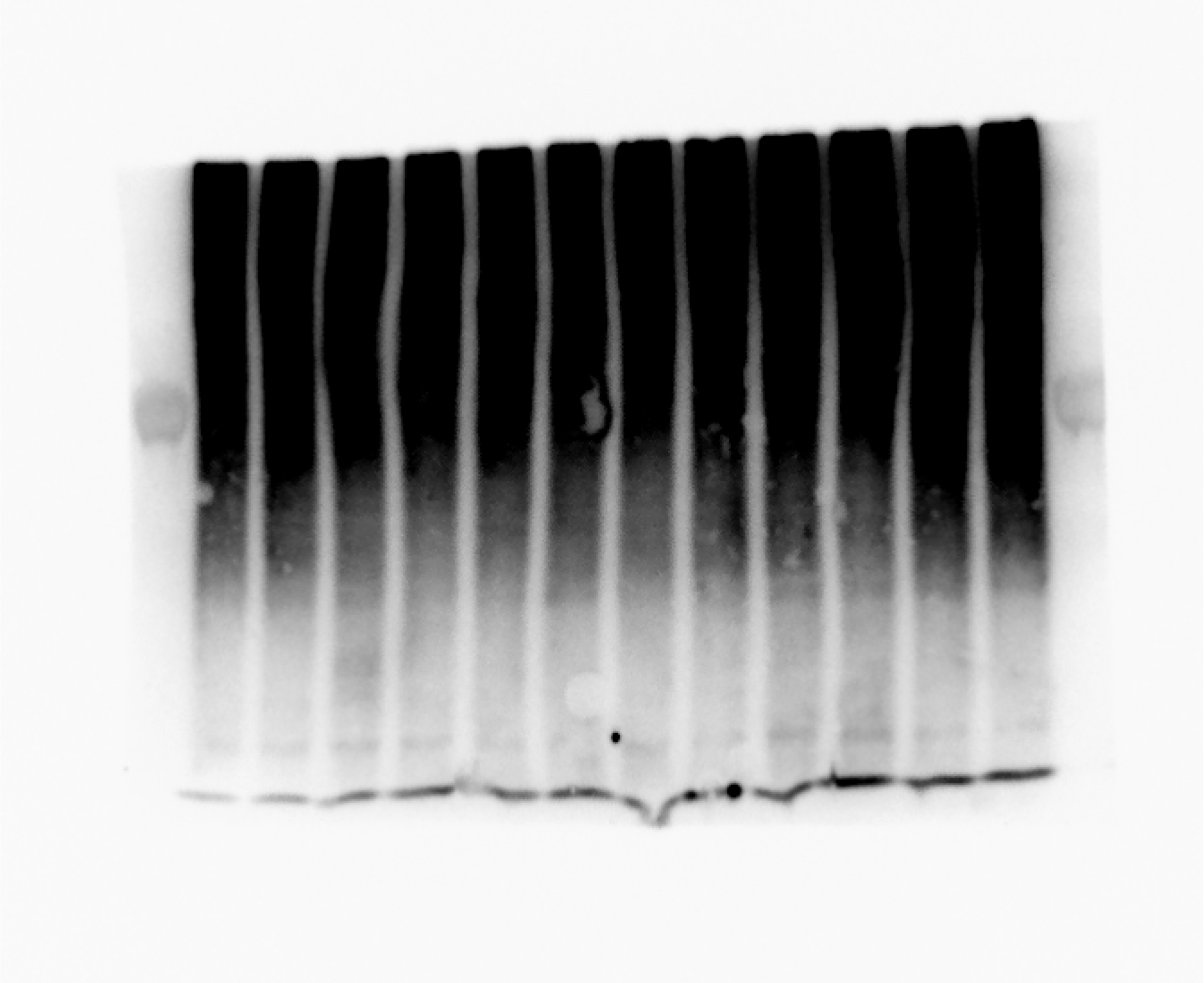

Figure 4K IP HA

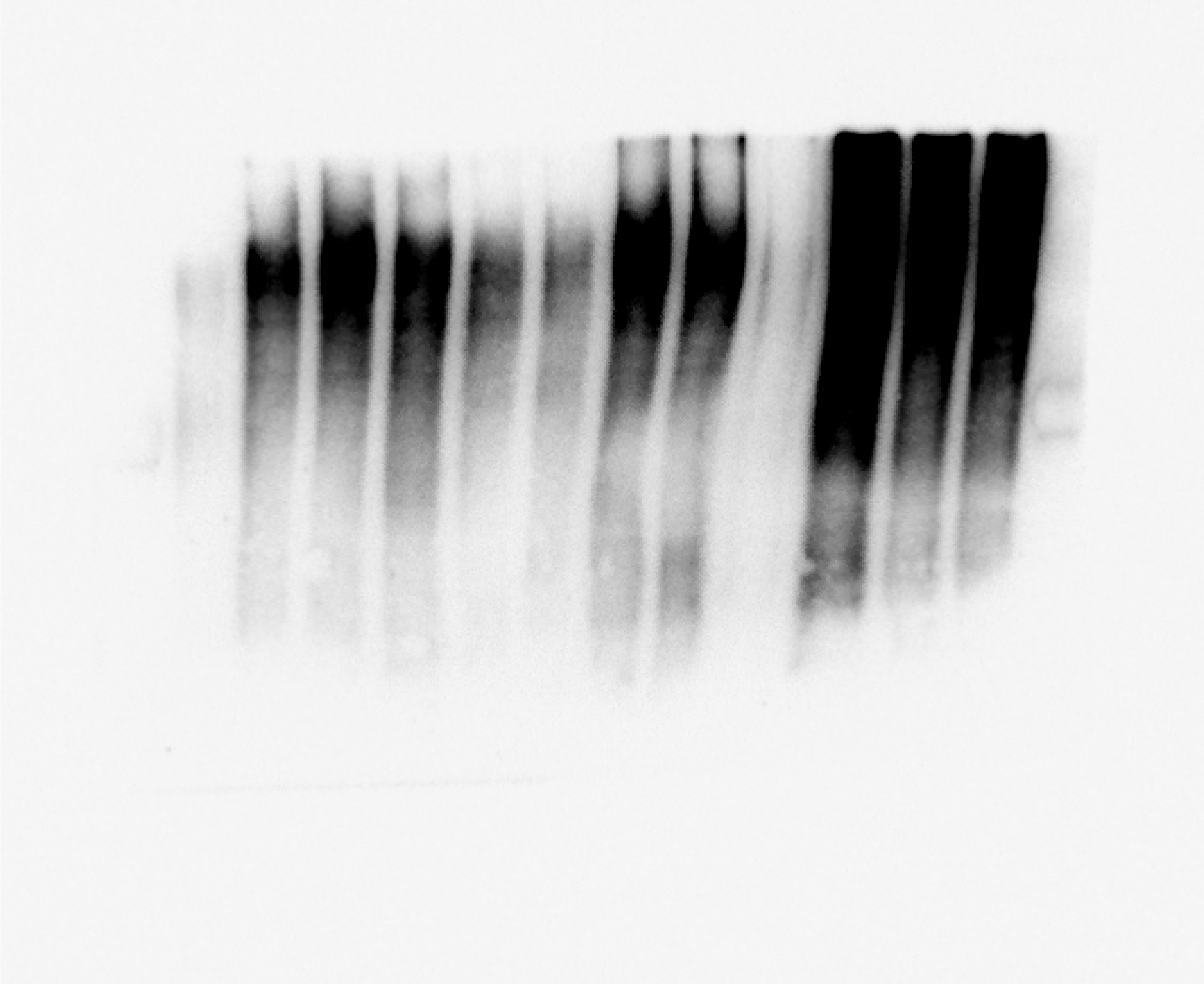

Figure 4L UMUC-3 Input

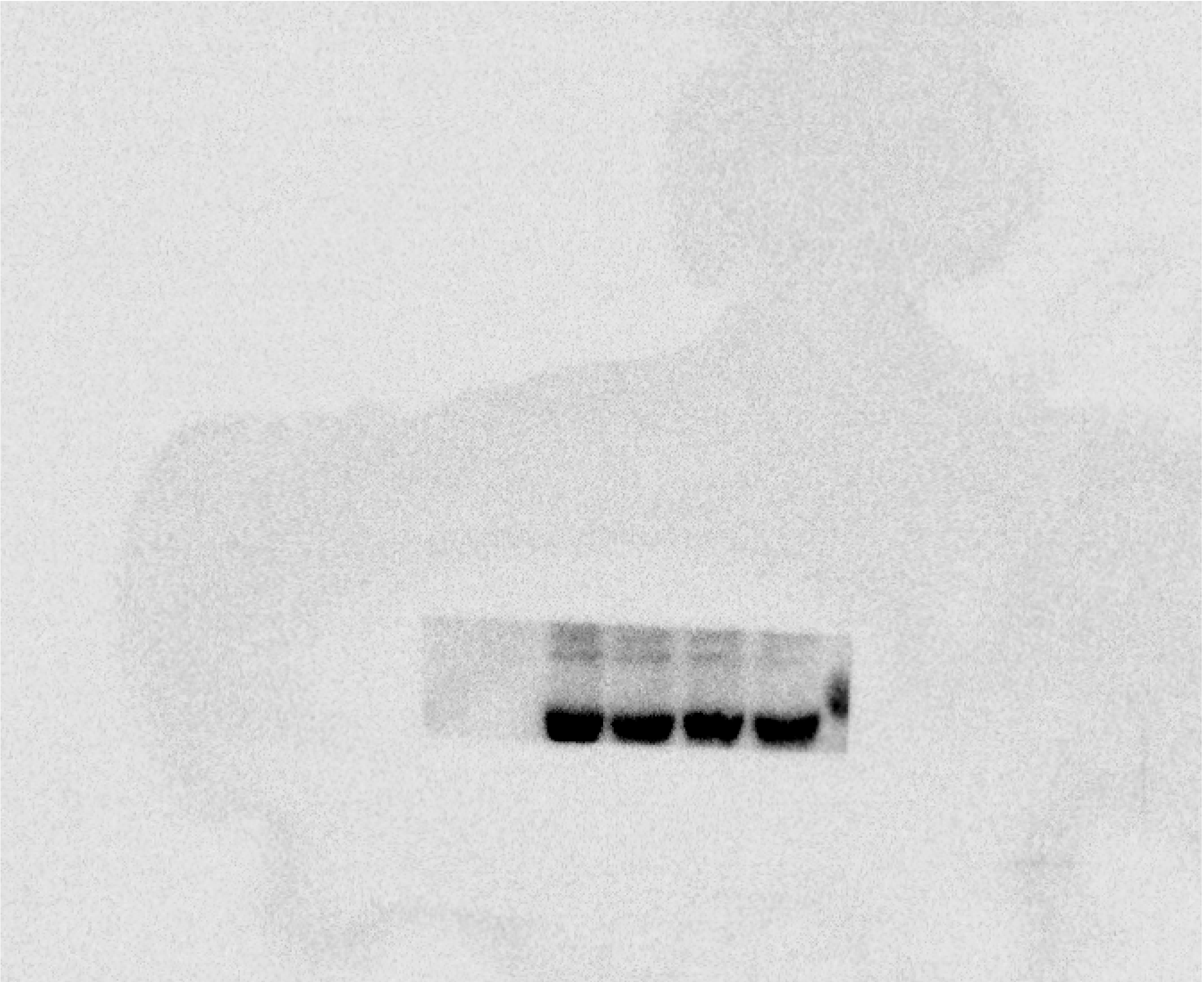

Figure 4L HA T24

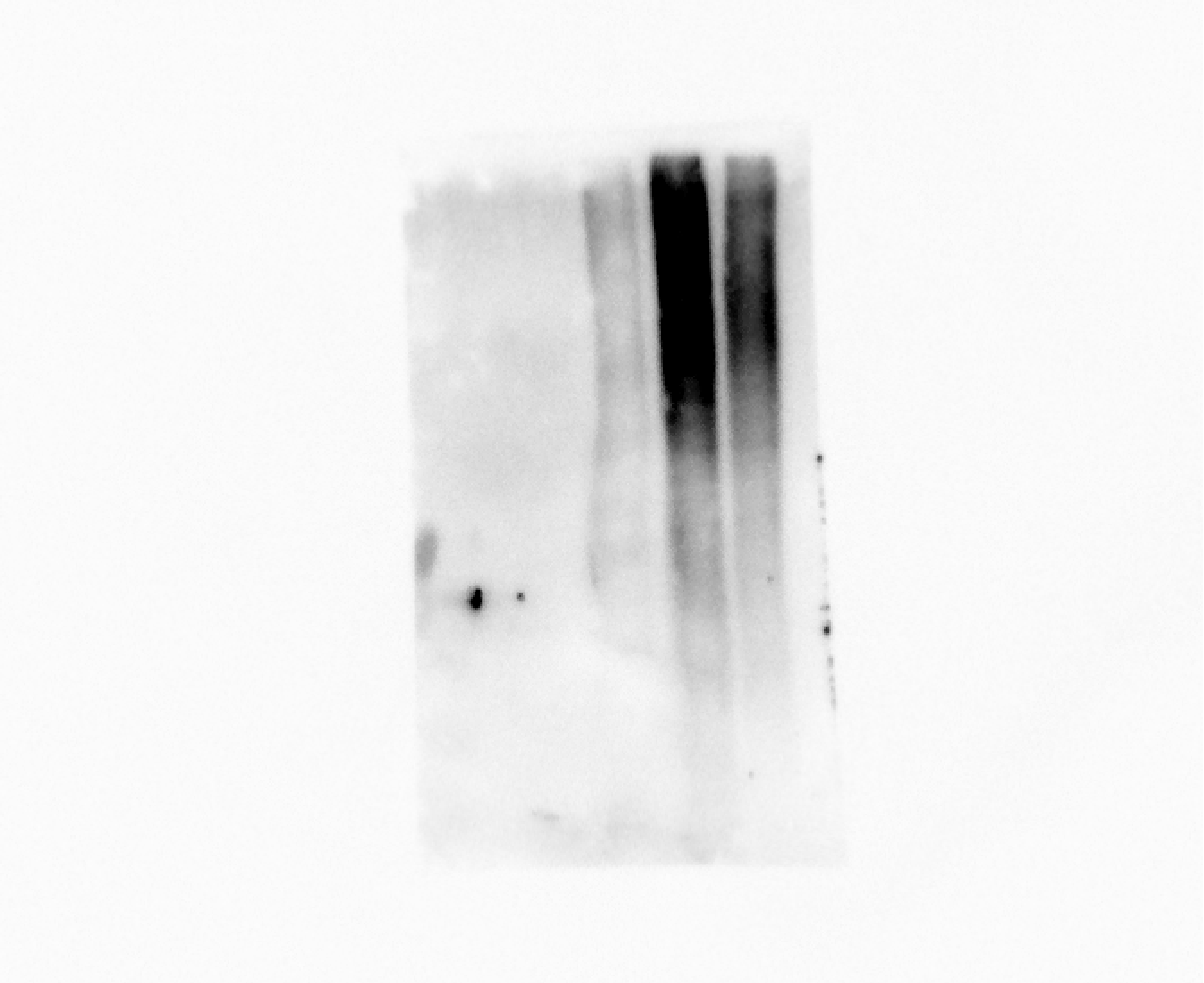

Figure 4L HA UMUC-3

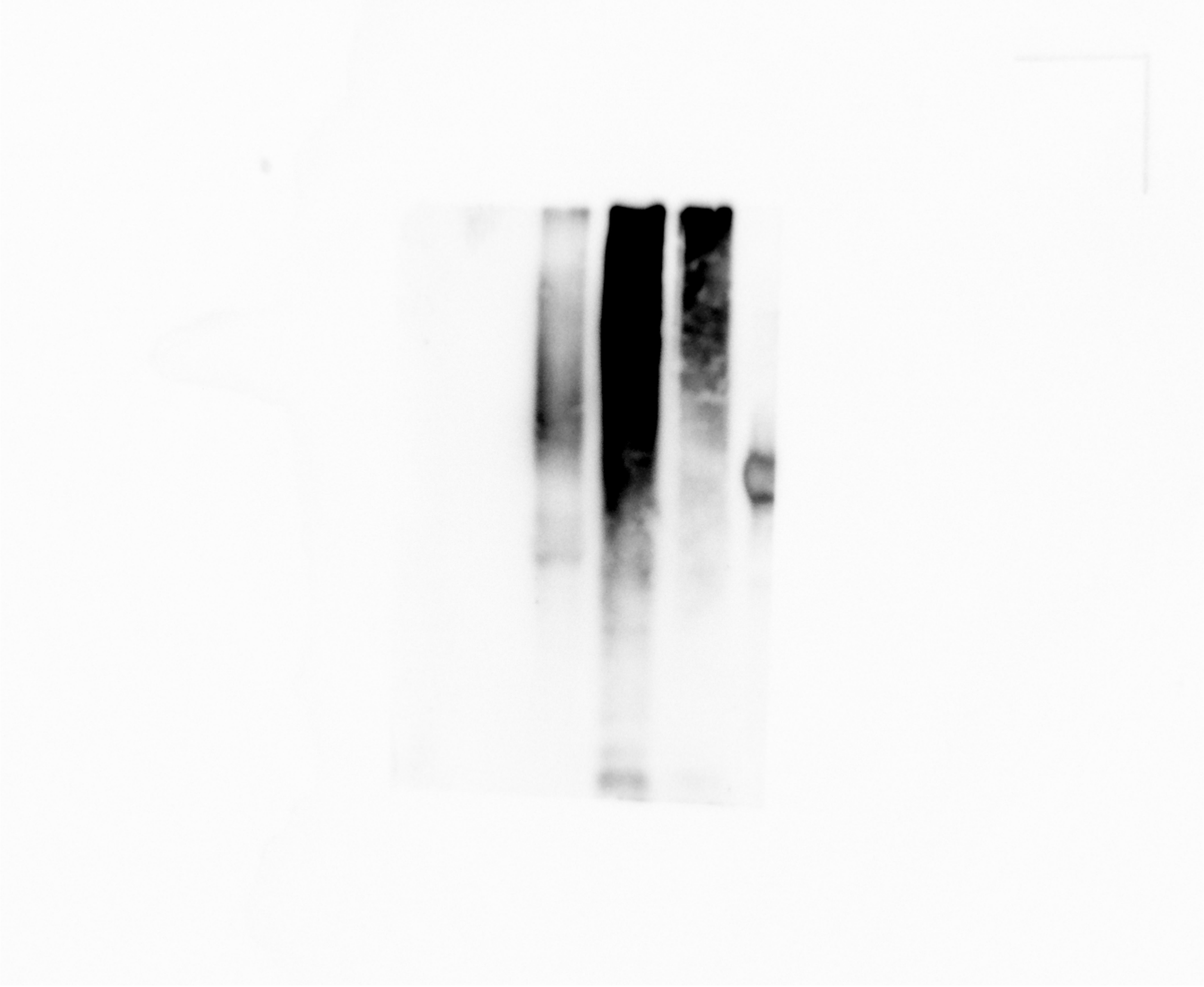

Figure 4L T24 Input

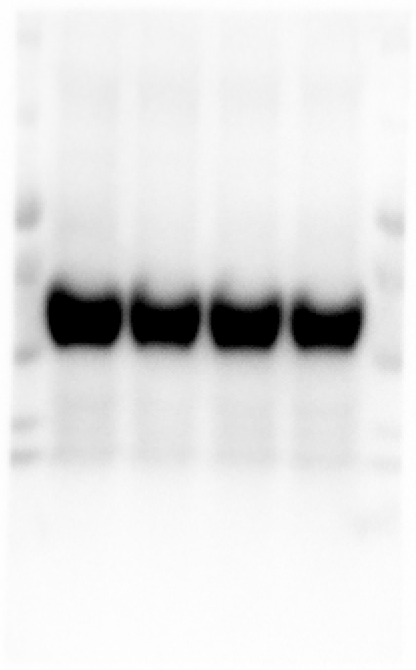

Figure 4M MAT2A UMUC-3

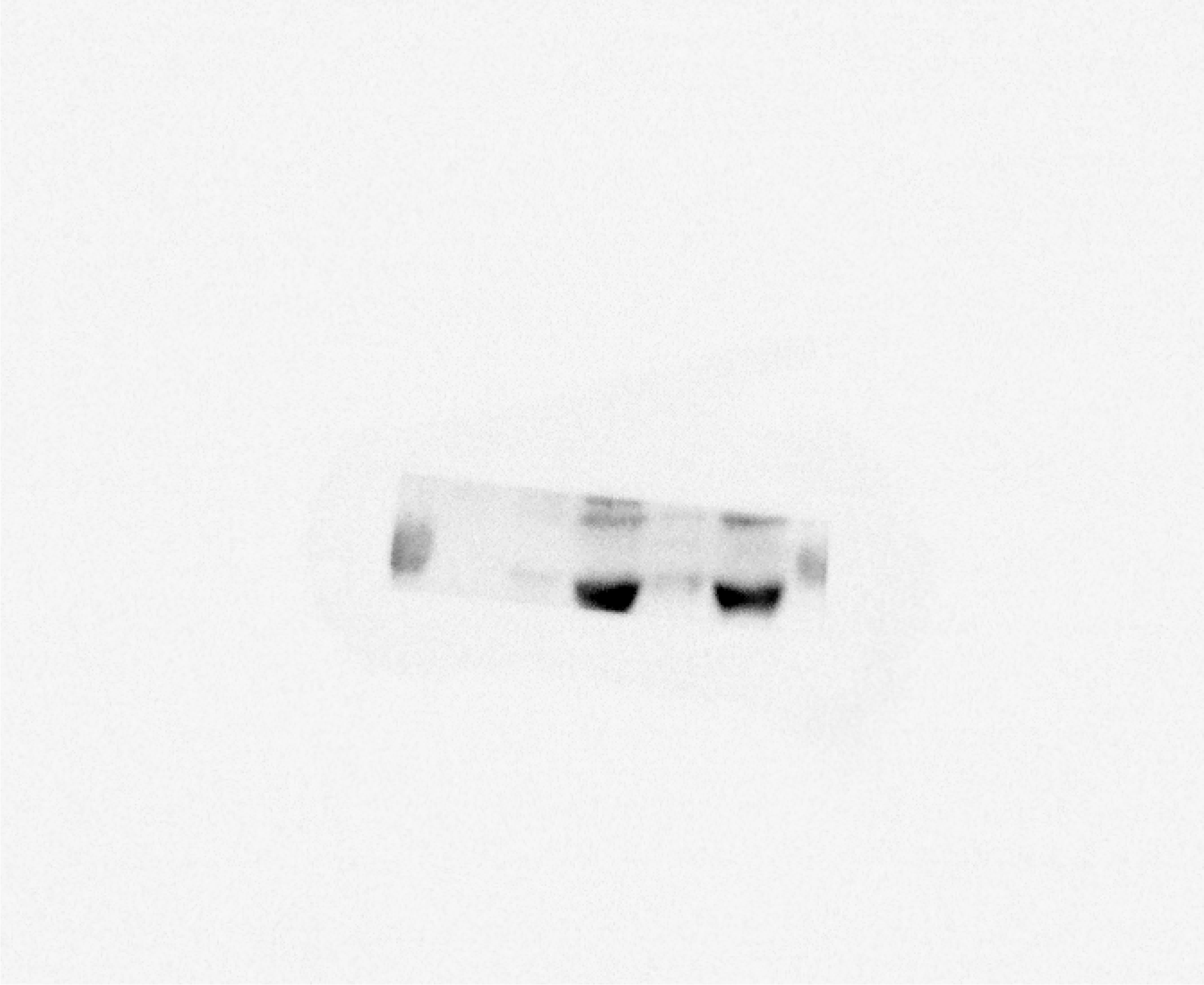

Figure 4M GAPDH T24

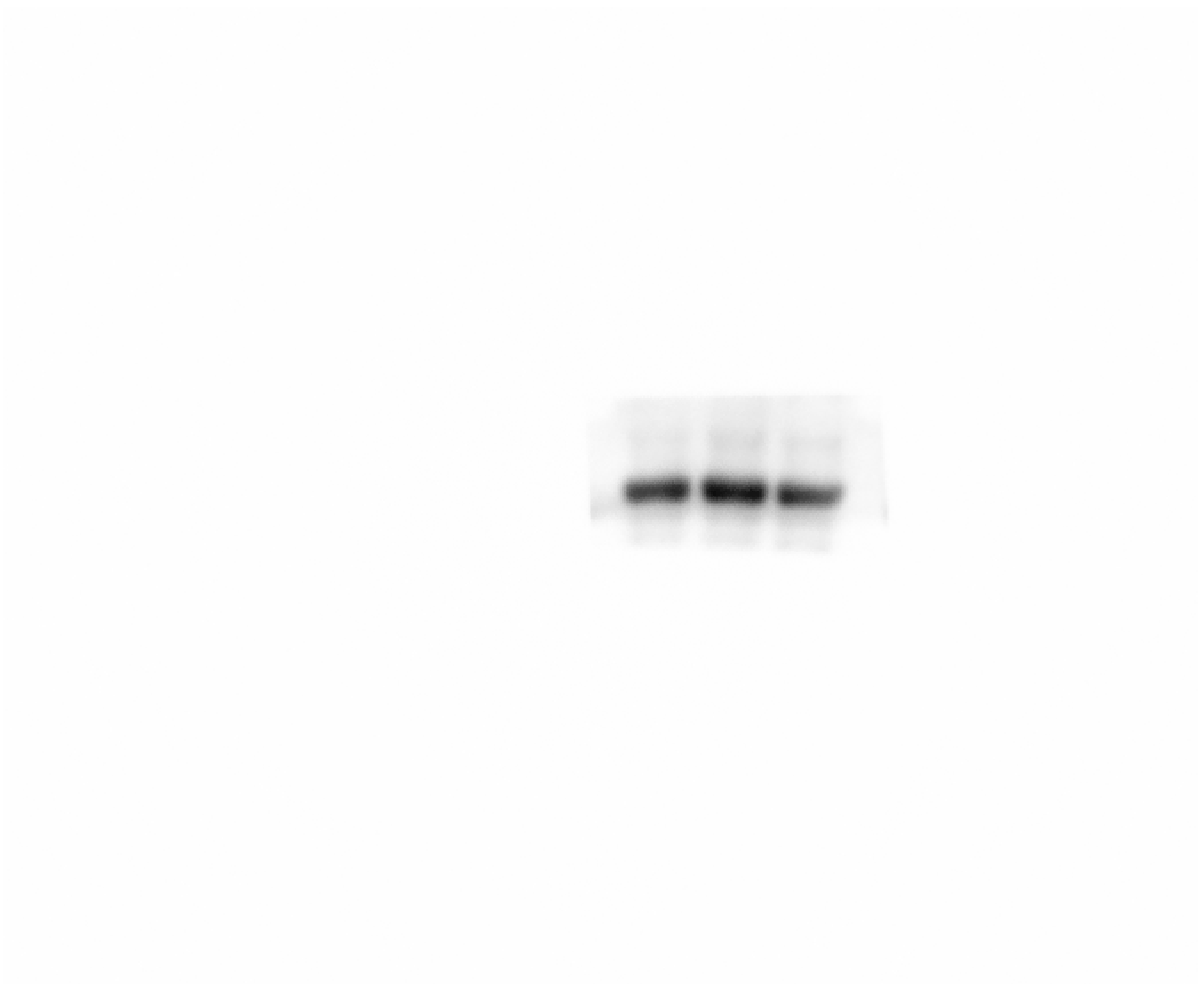

Figure 4M GAPDH UMUC-3

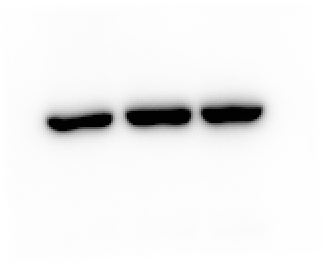

Figure 4M MAT2A T24

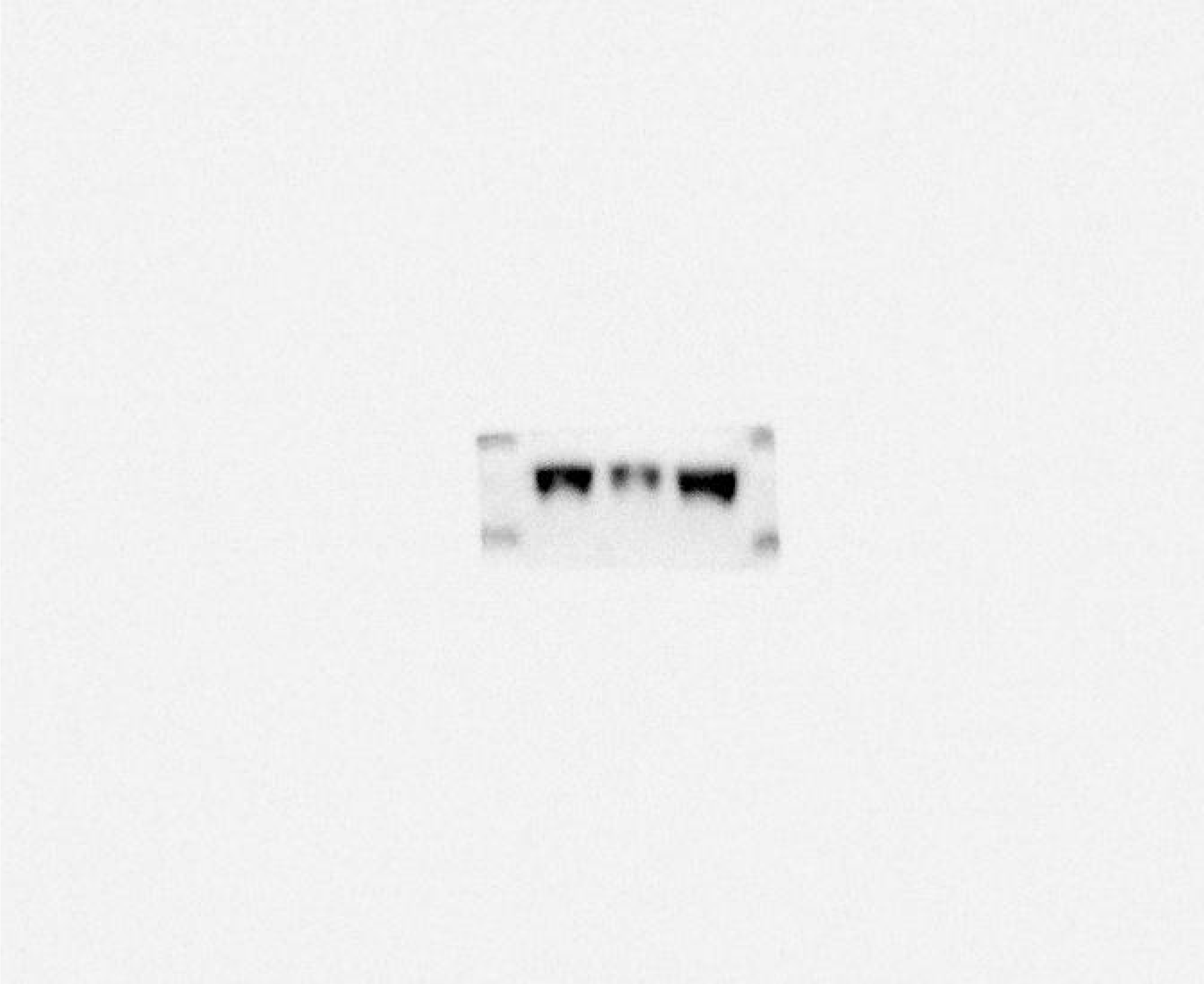

Figure 4N TRIM25

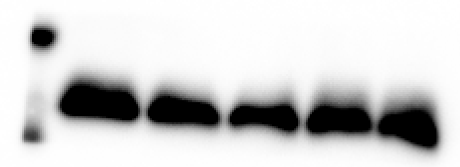

Figure 4N GAPDH

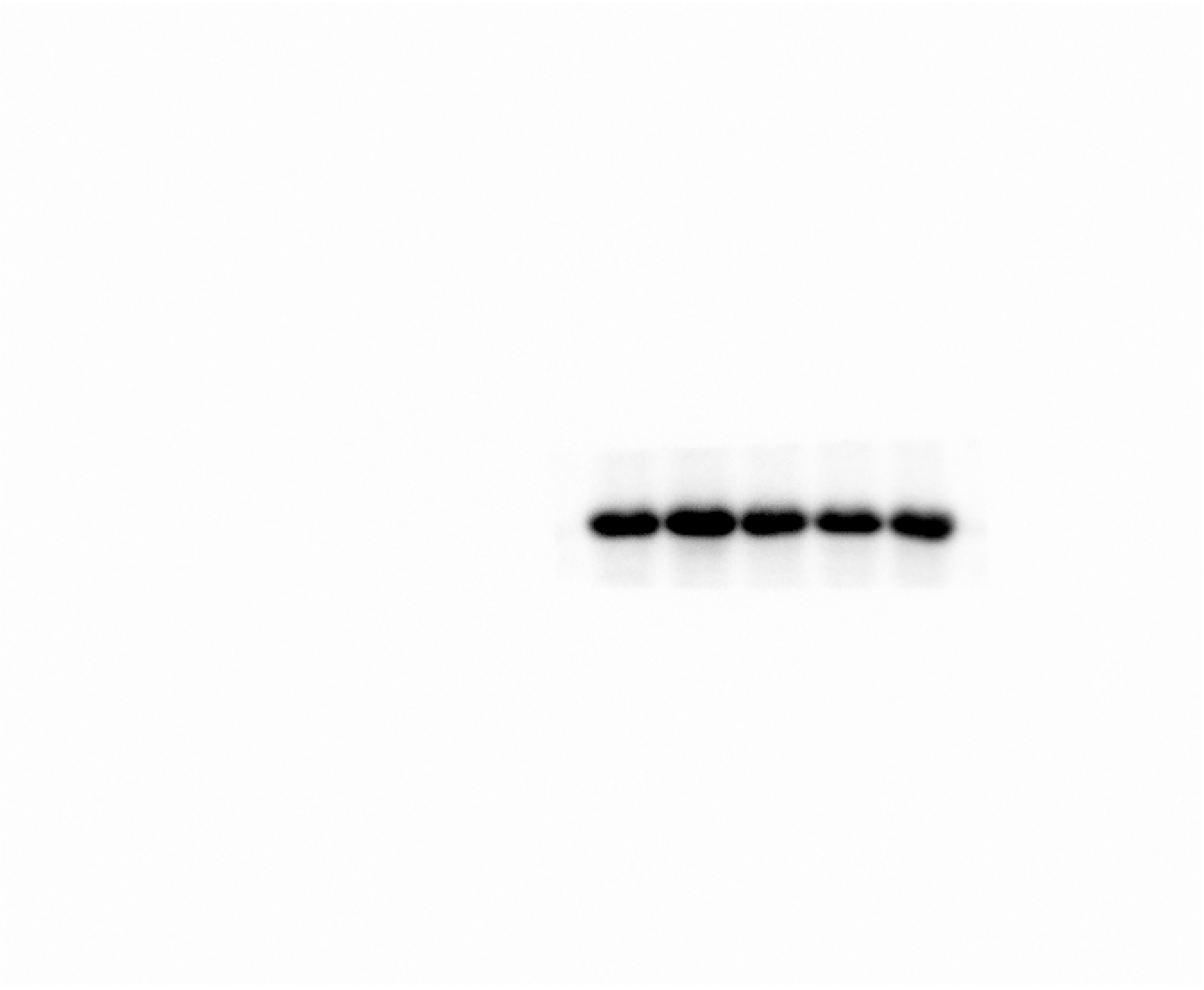

Figure 4N IP MAT2A

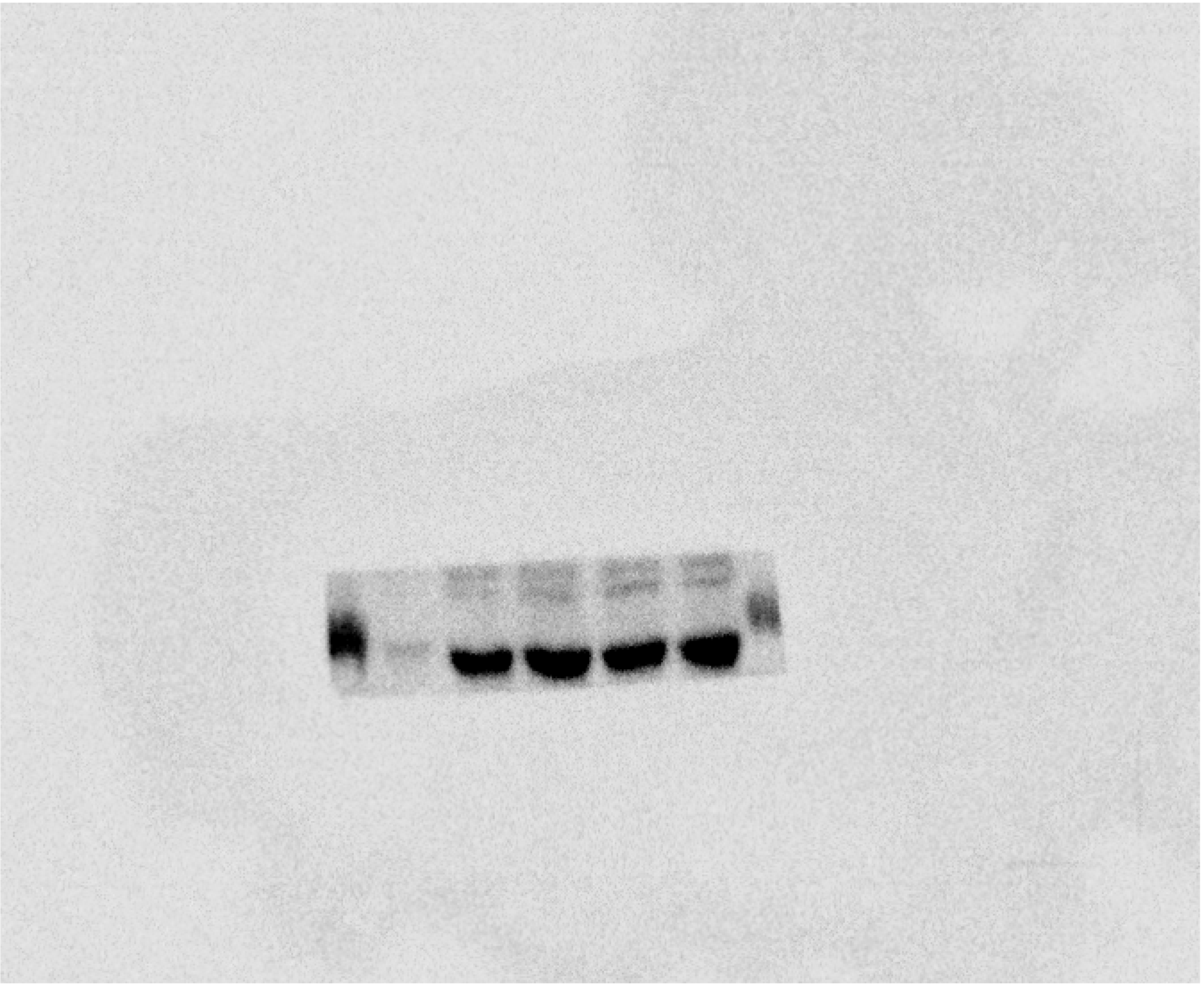

Figure 4N IP TRIM25

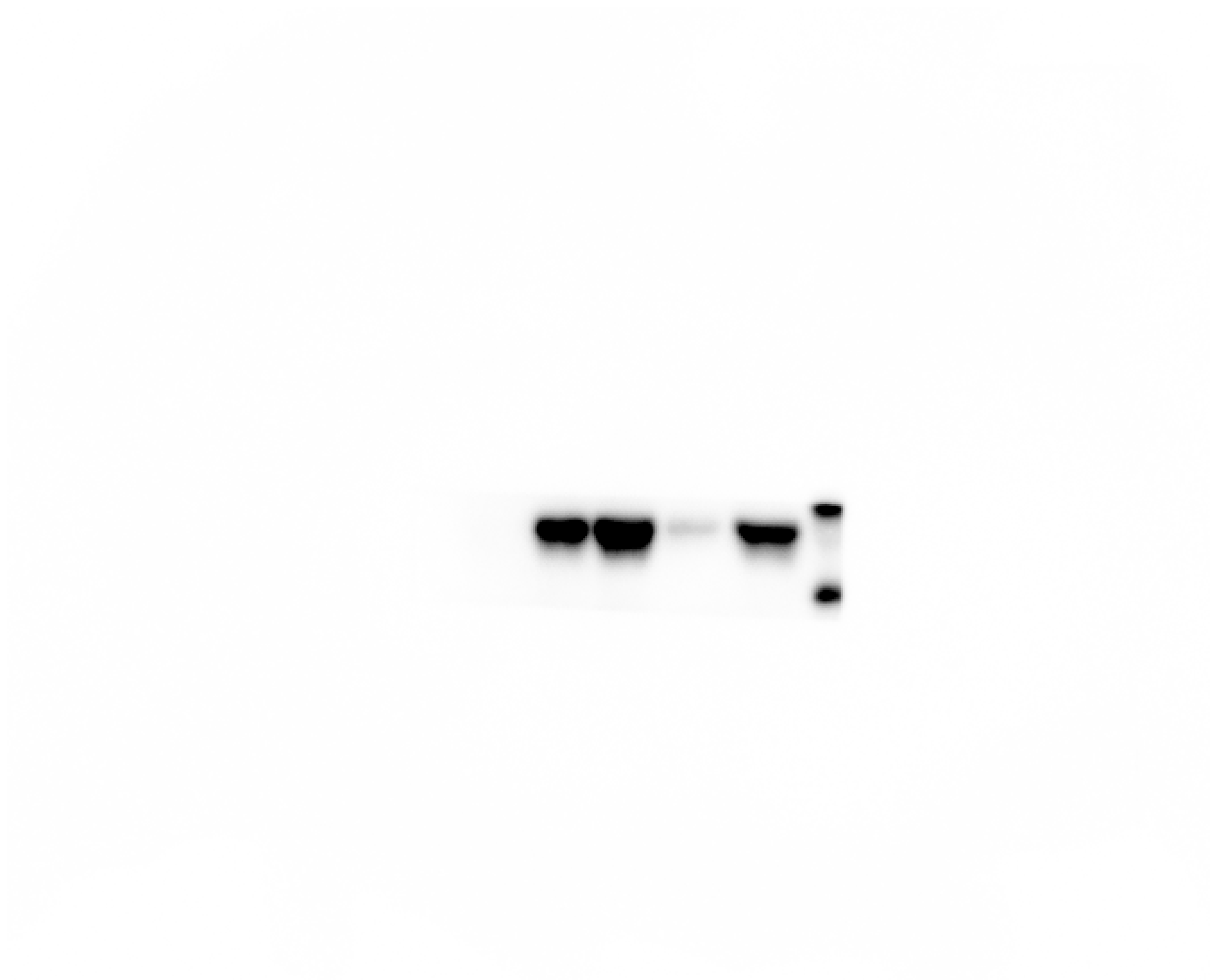

Figure 4N MAT2A

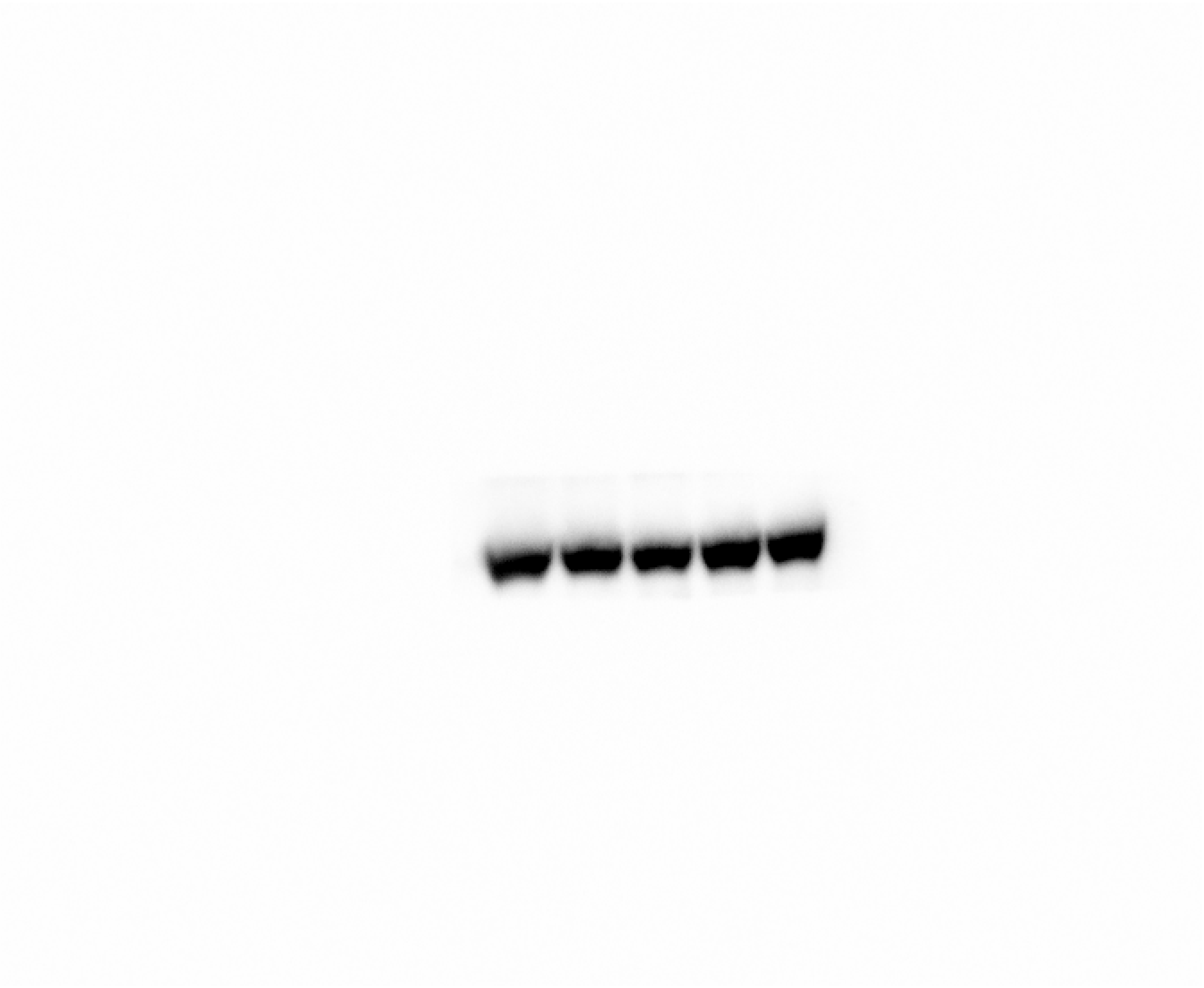

Figure 6E Total H3

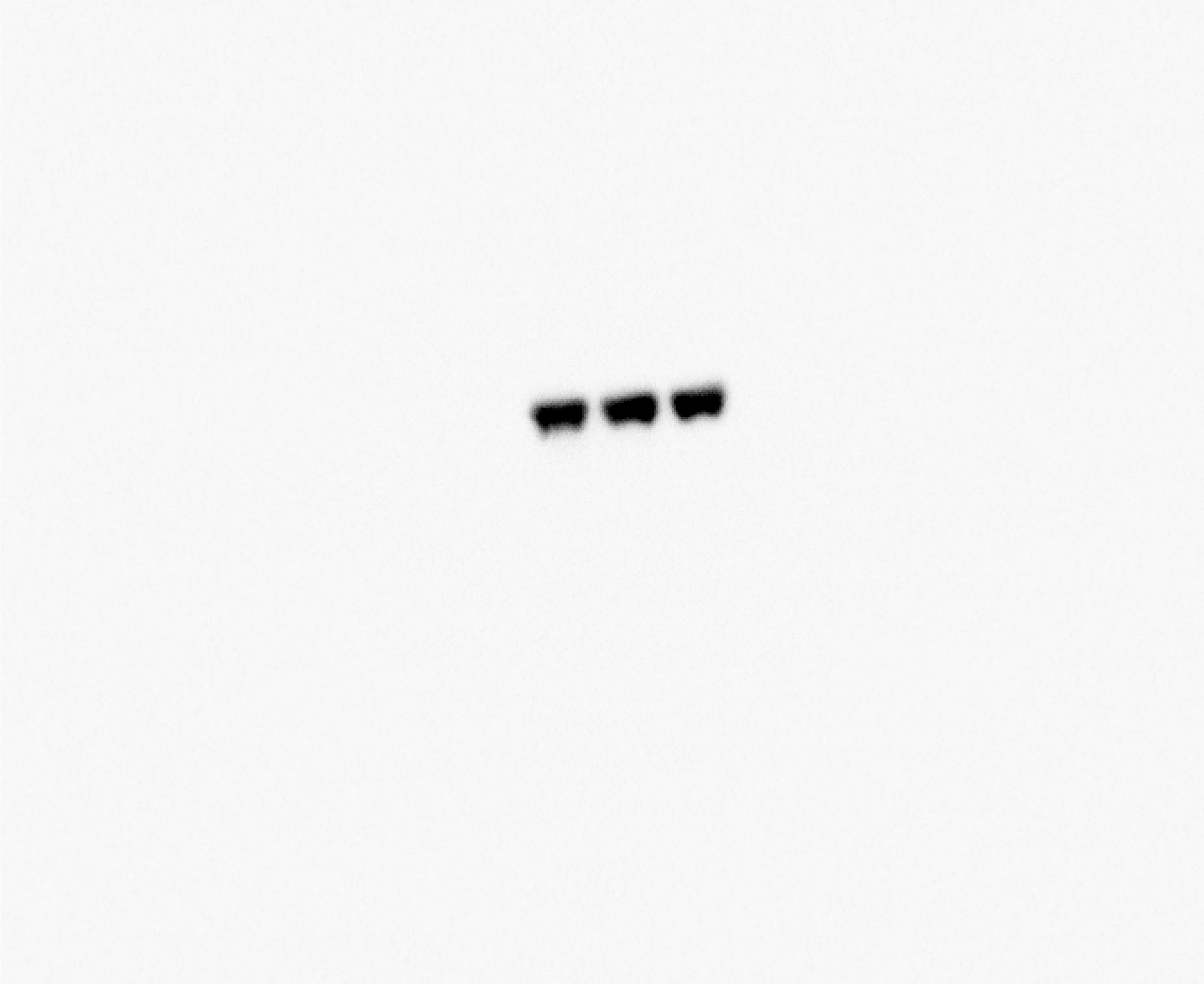

Figure 6E GAPDH

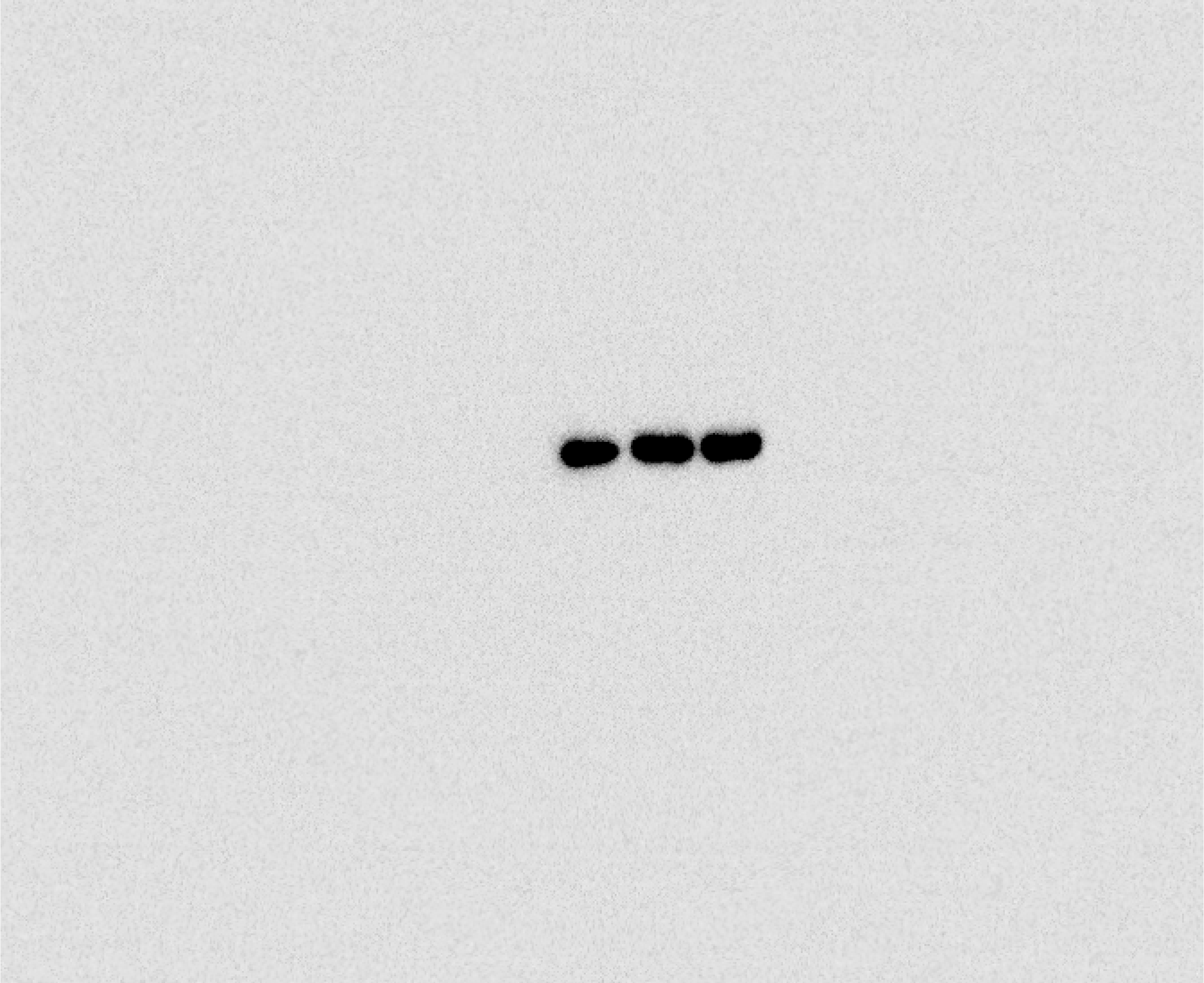

Figure 6E H3K4me3

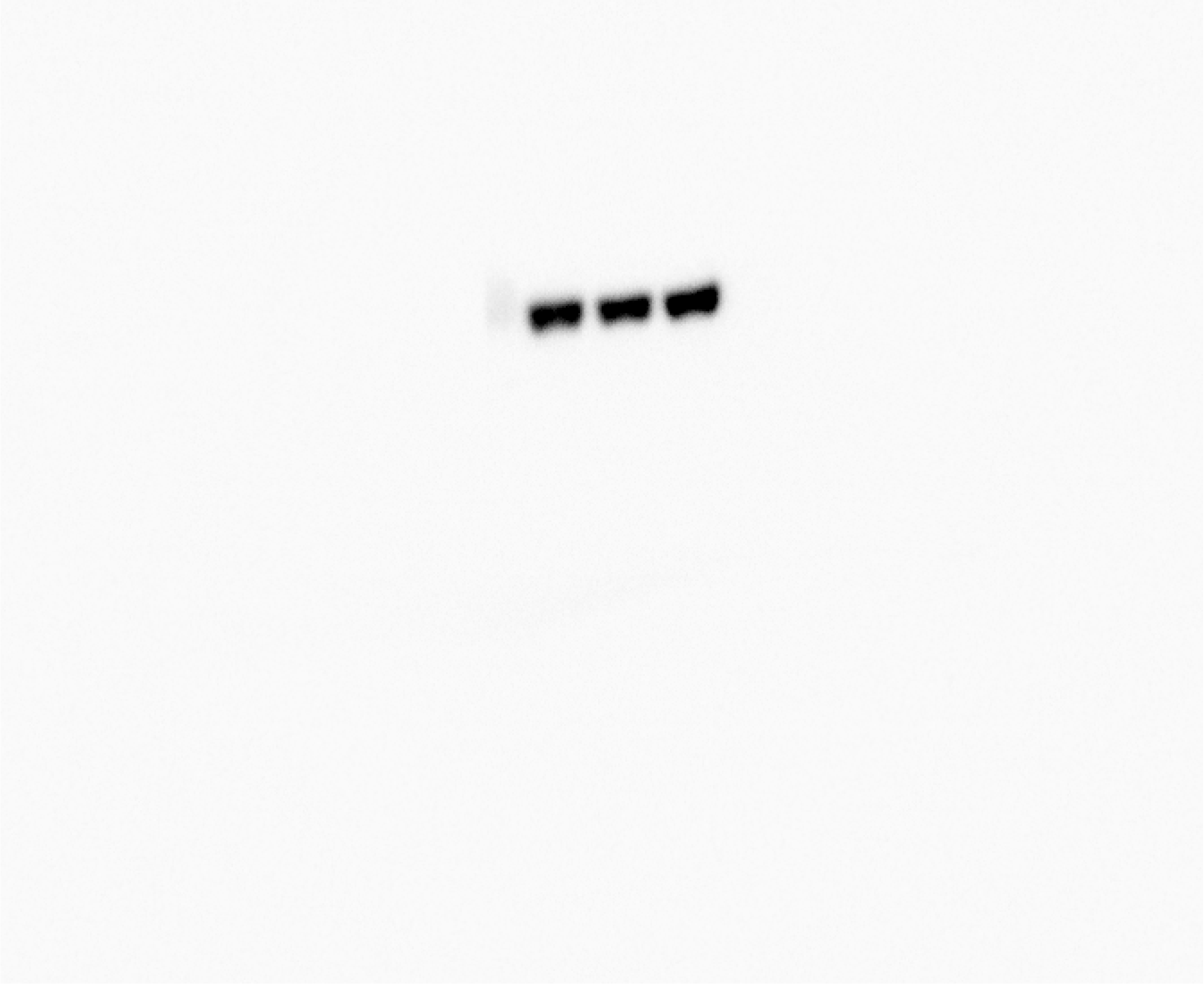

Figure 6E H3K9me3

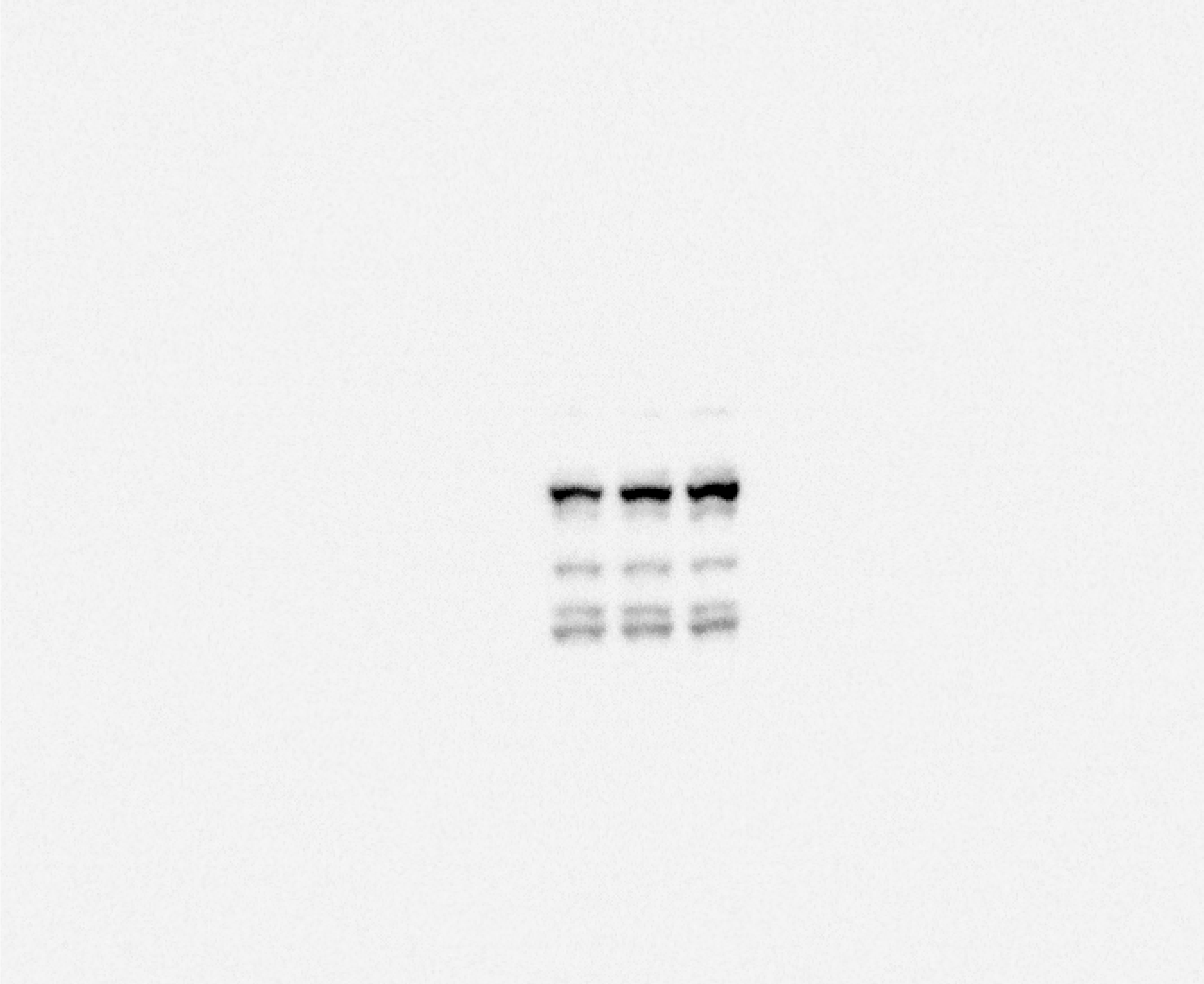

Figure 6E H3K27me3

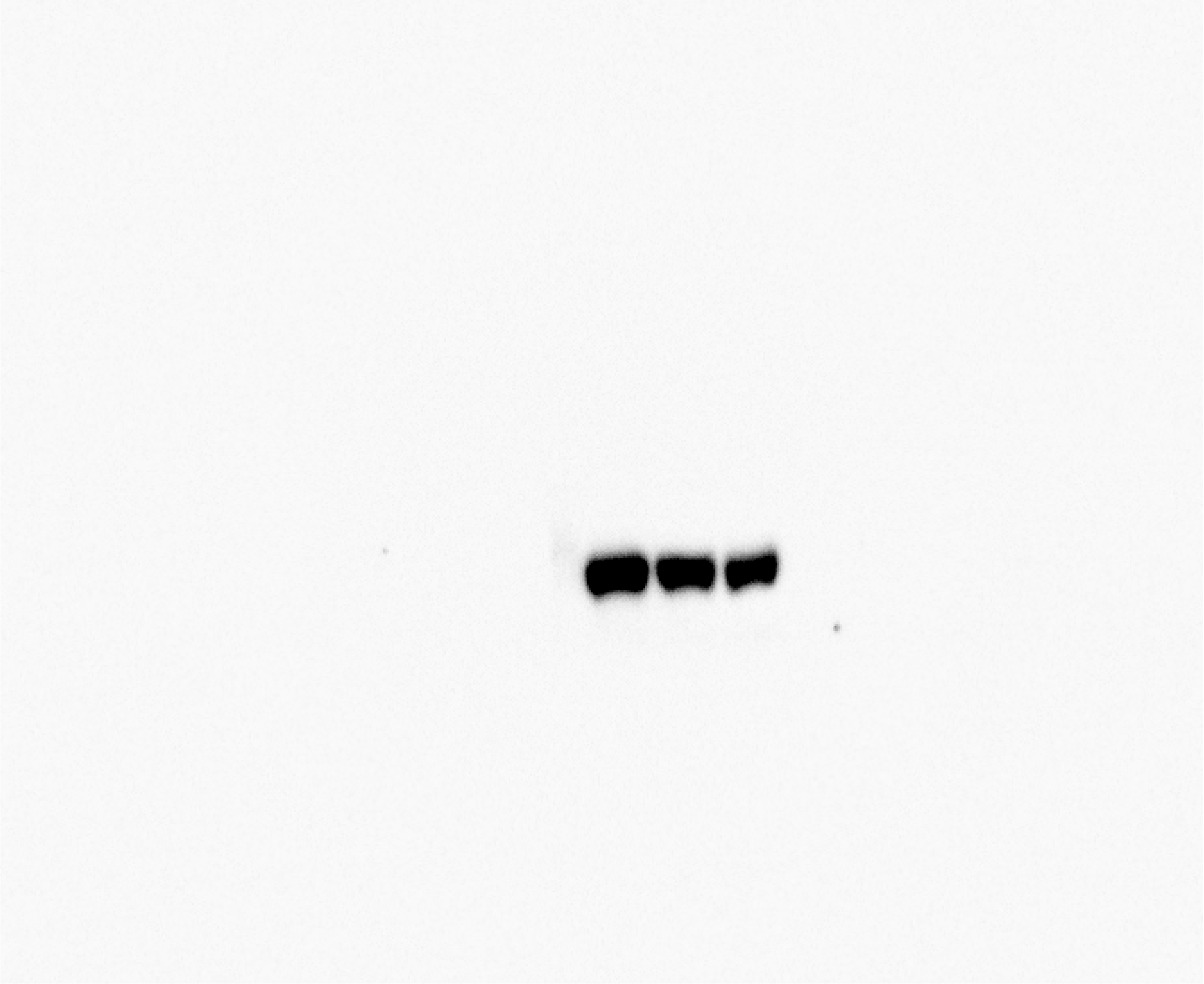

Figure 6E H3K36me2

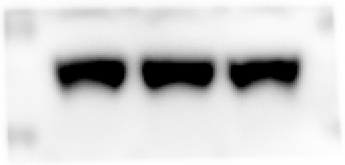

Figure 6E H3K36me3

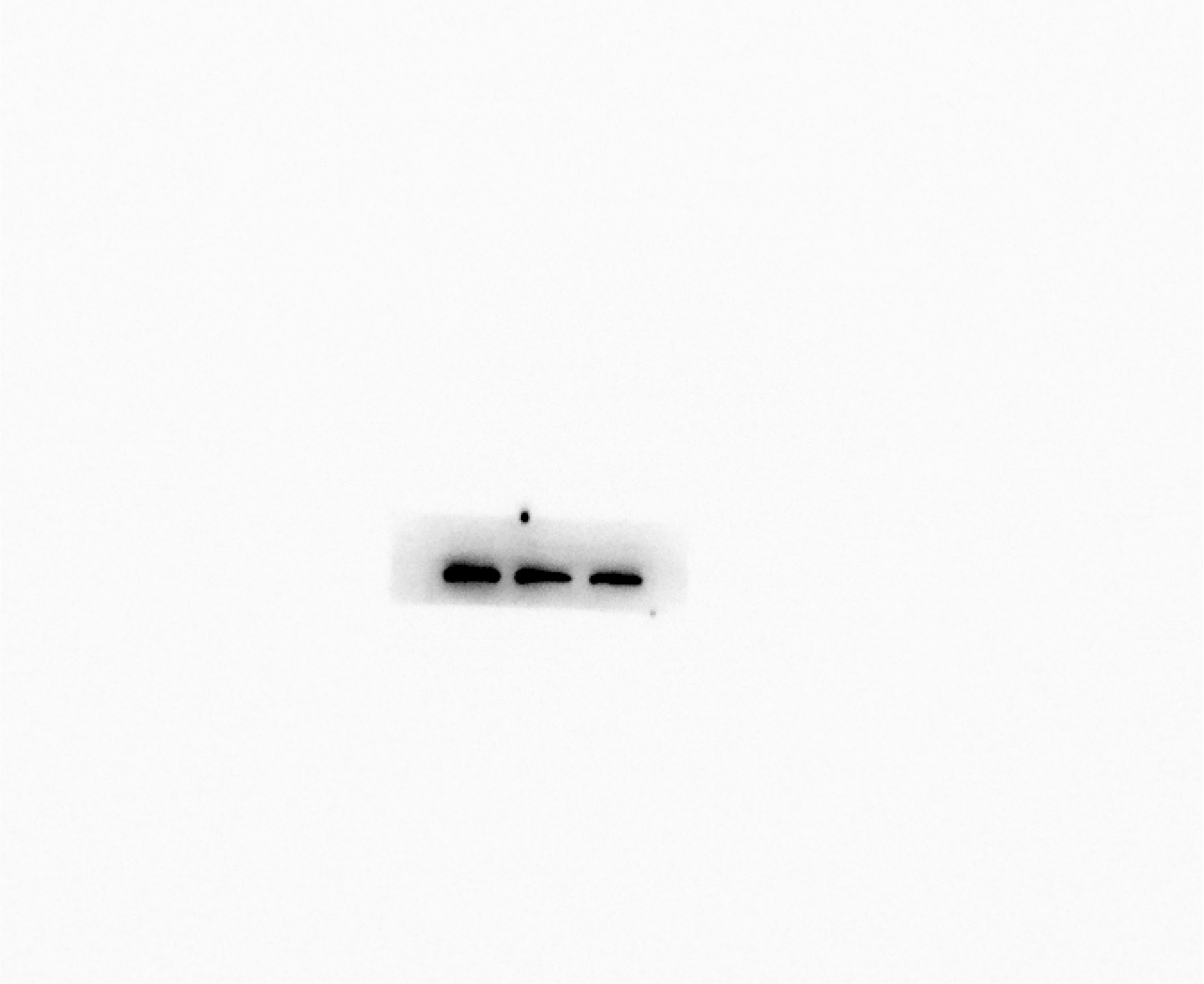

Figure 6E H3K79me2

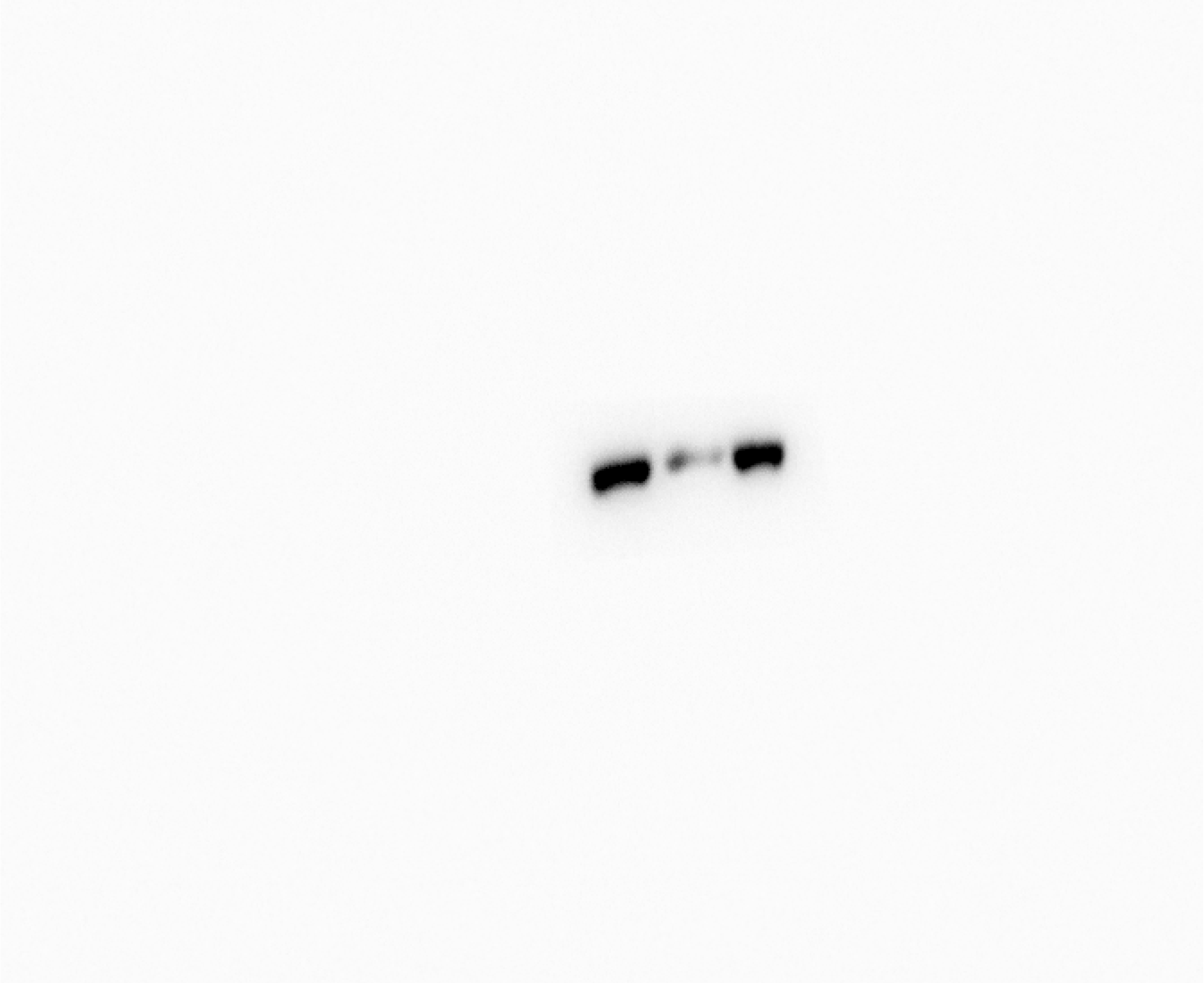

Figure 6E H3K79me3

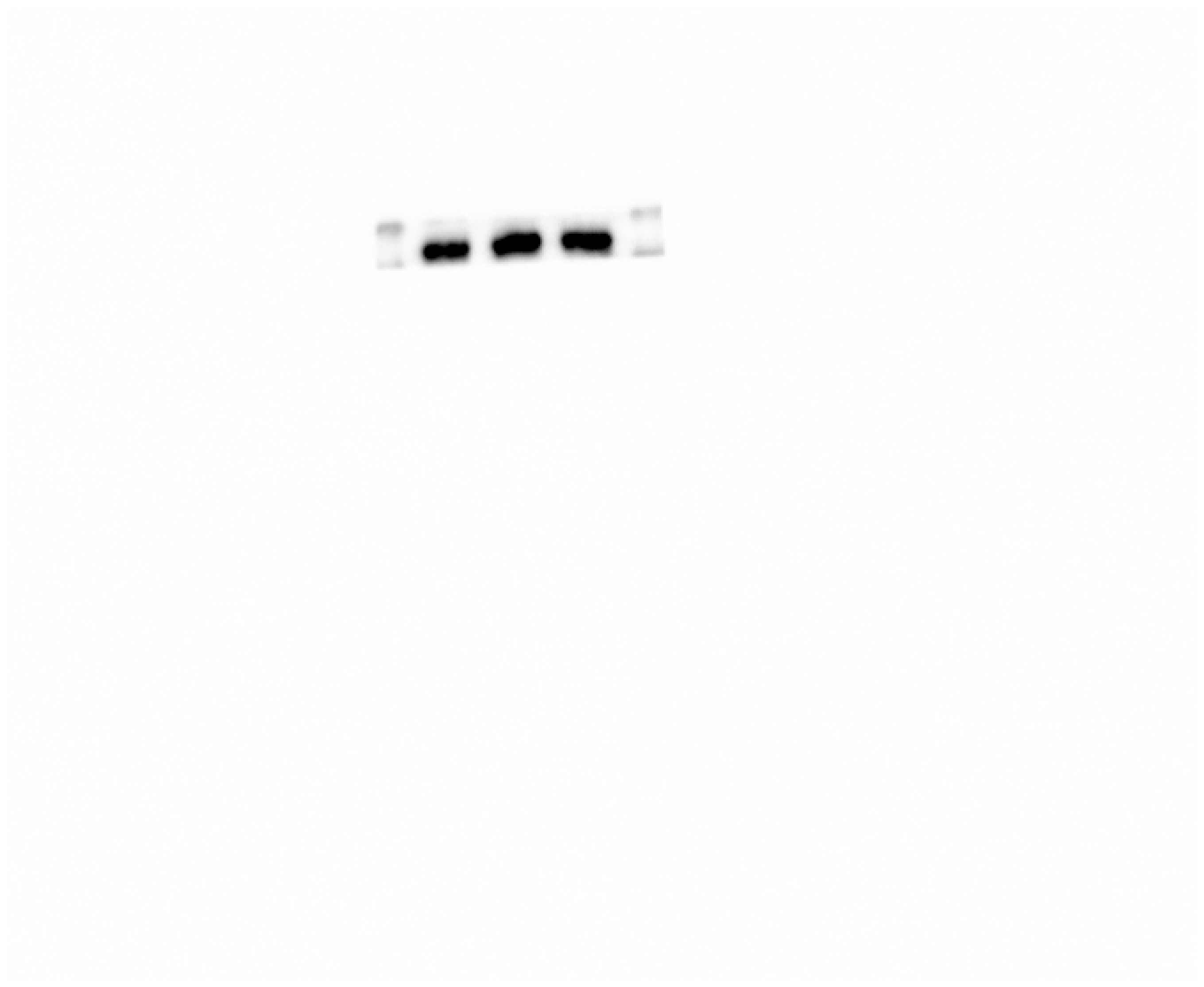

Figure 6F STAT5

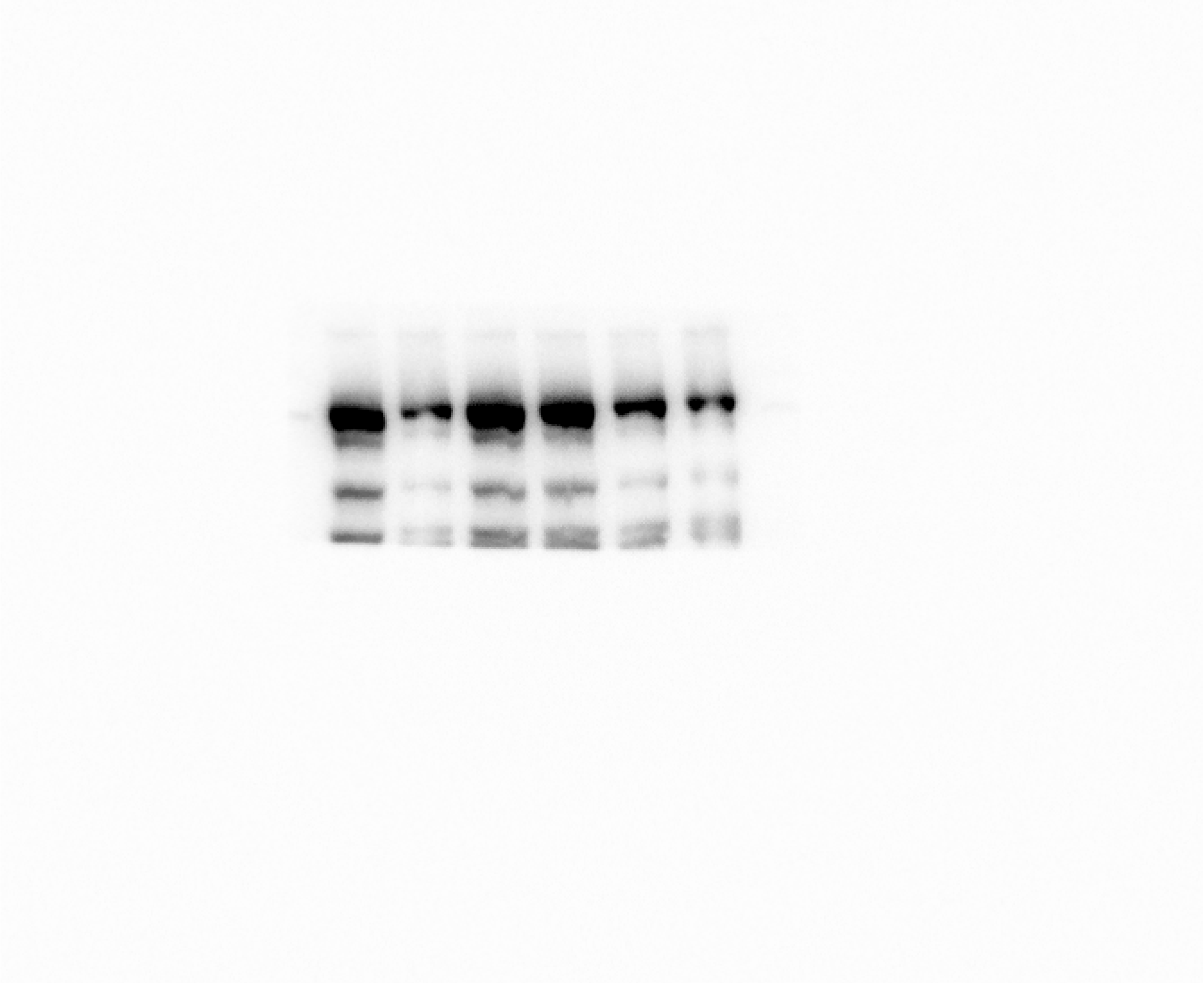

Figure 6F GAPDH

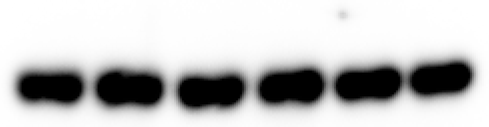

Figure 6F H3

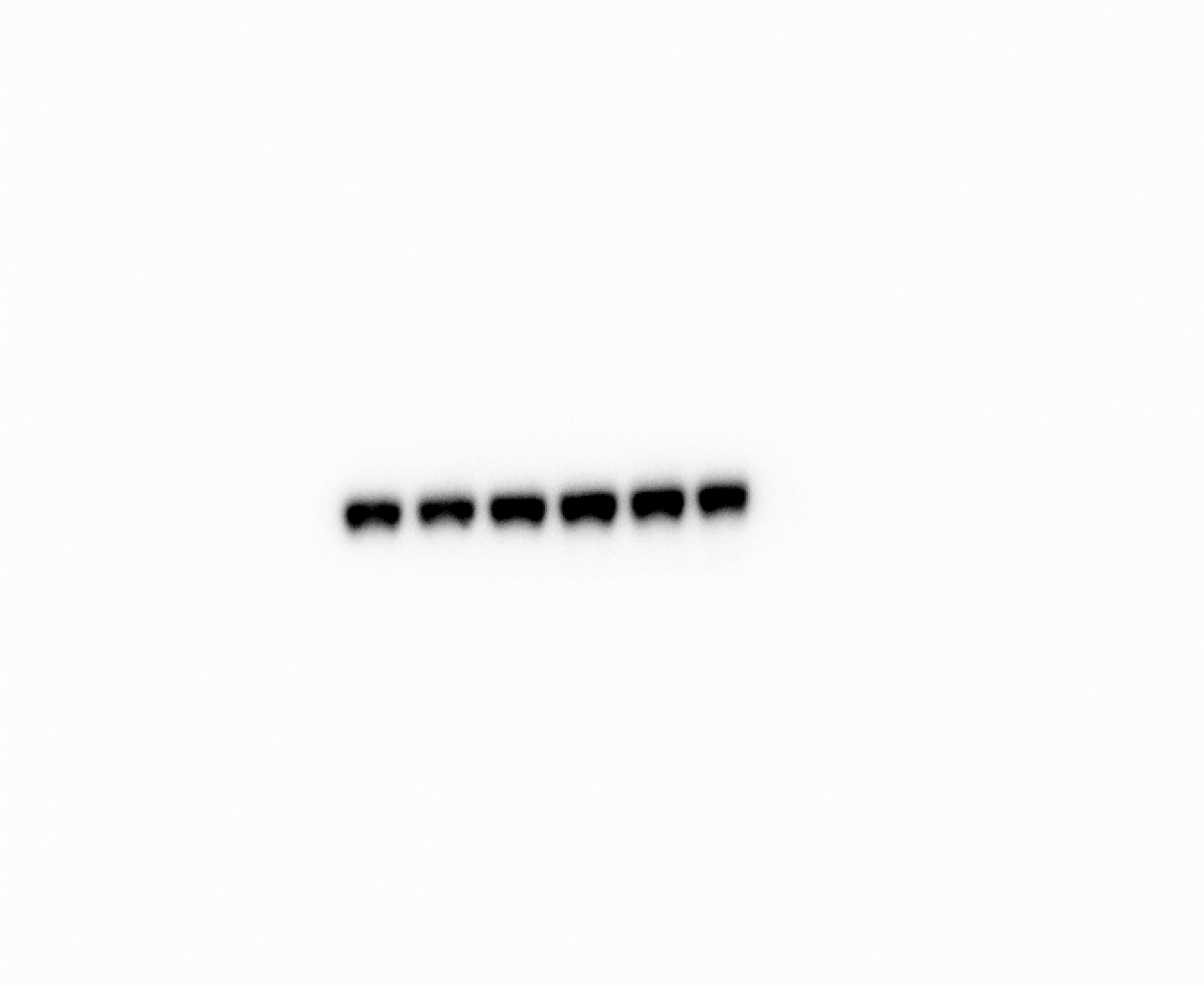

Figure 6F H3K79me2

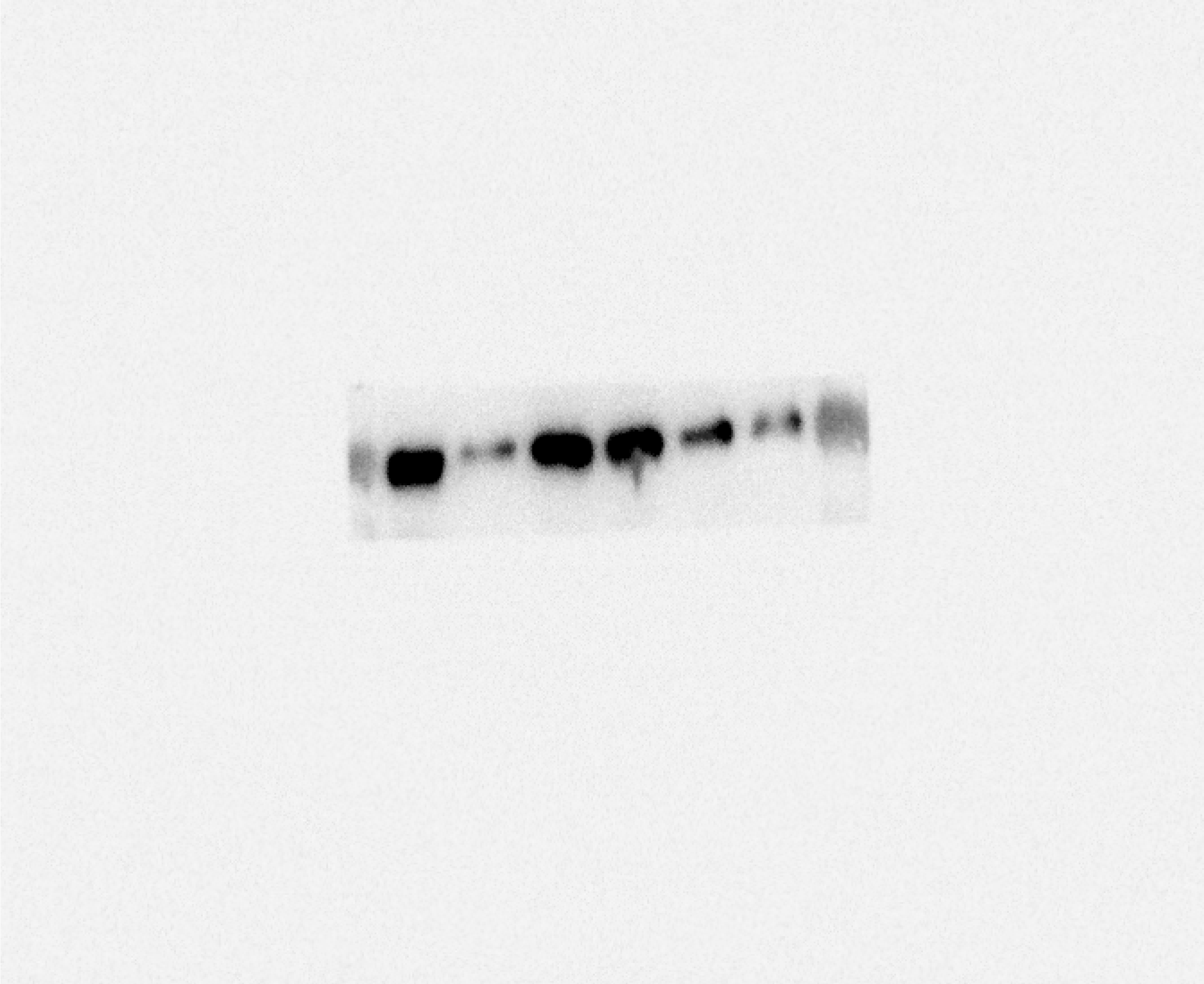

Figure 6G p1 STAT5

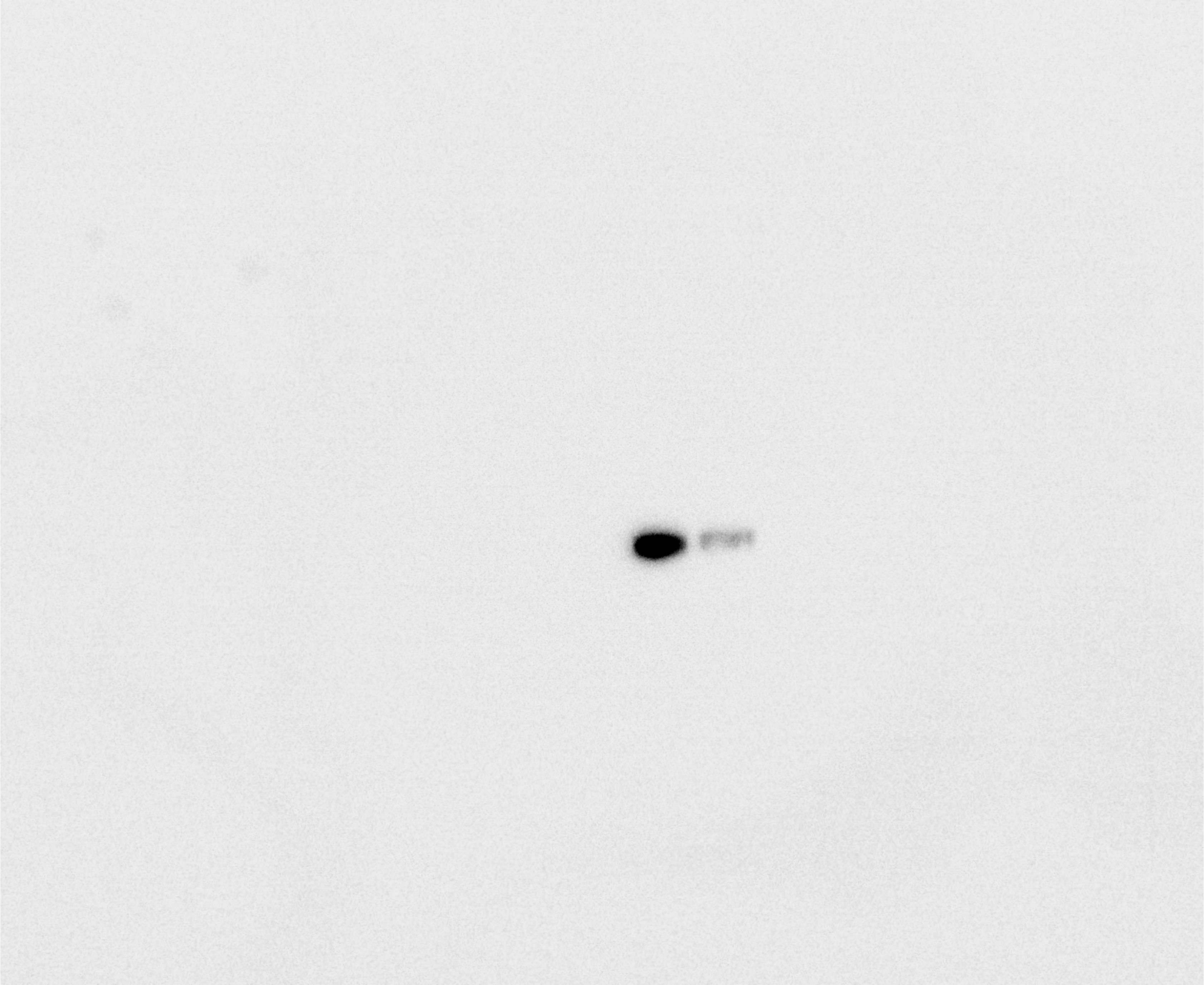

Figure 6G p1 GAPDH

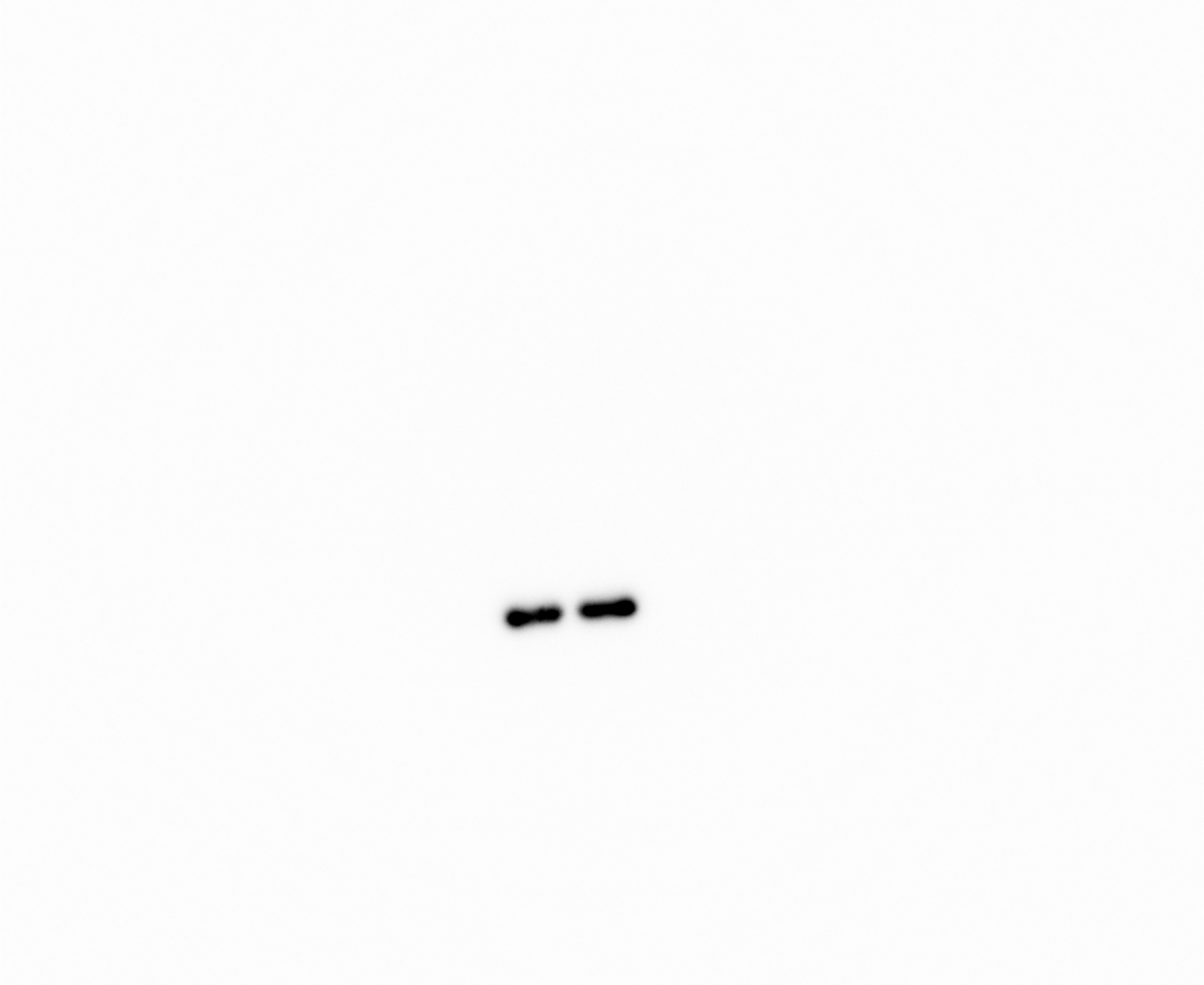

Figure 6G p1 H3

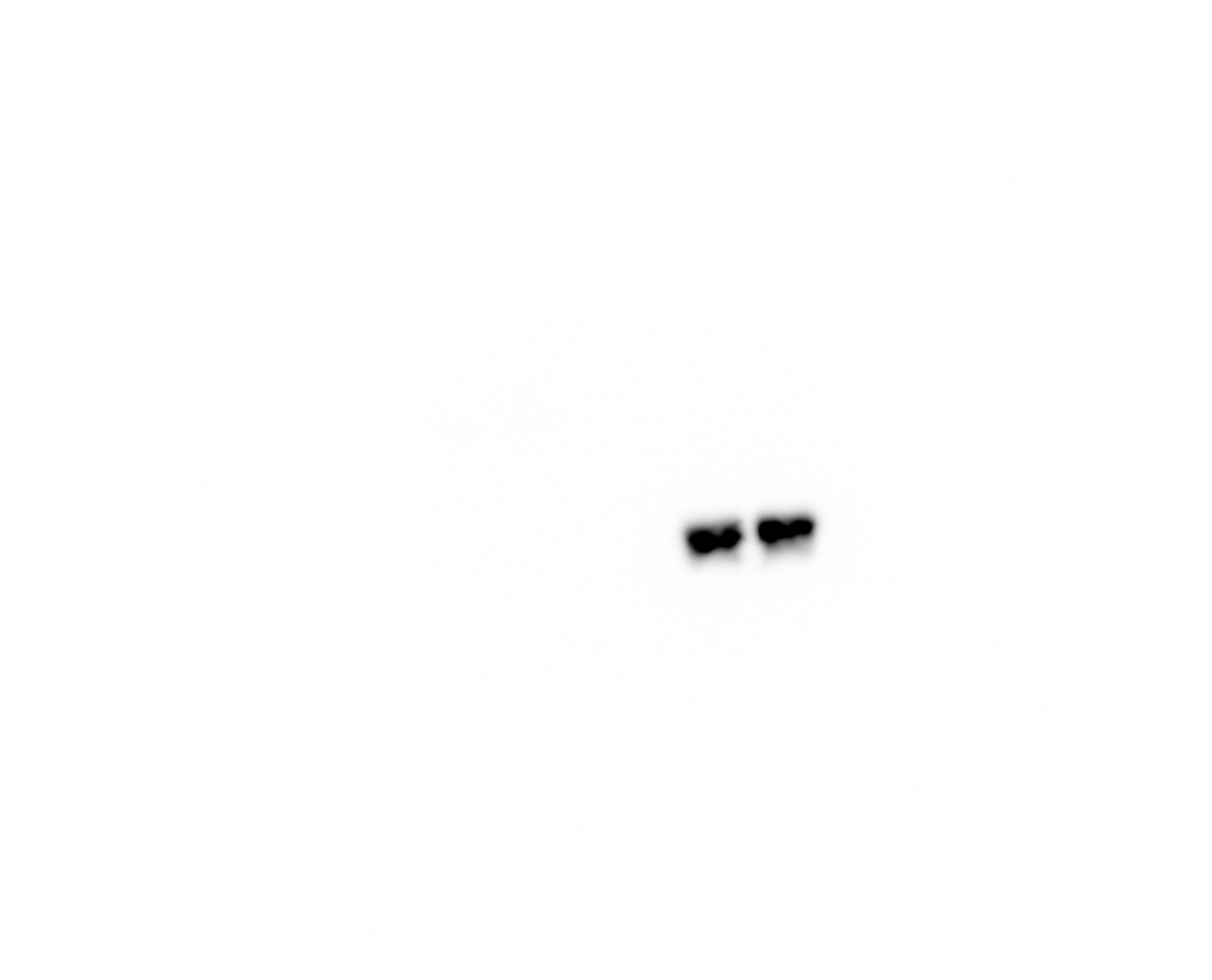

Figure 6G p1 H3K

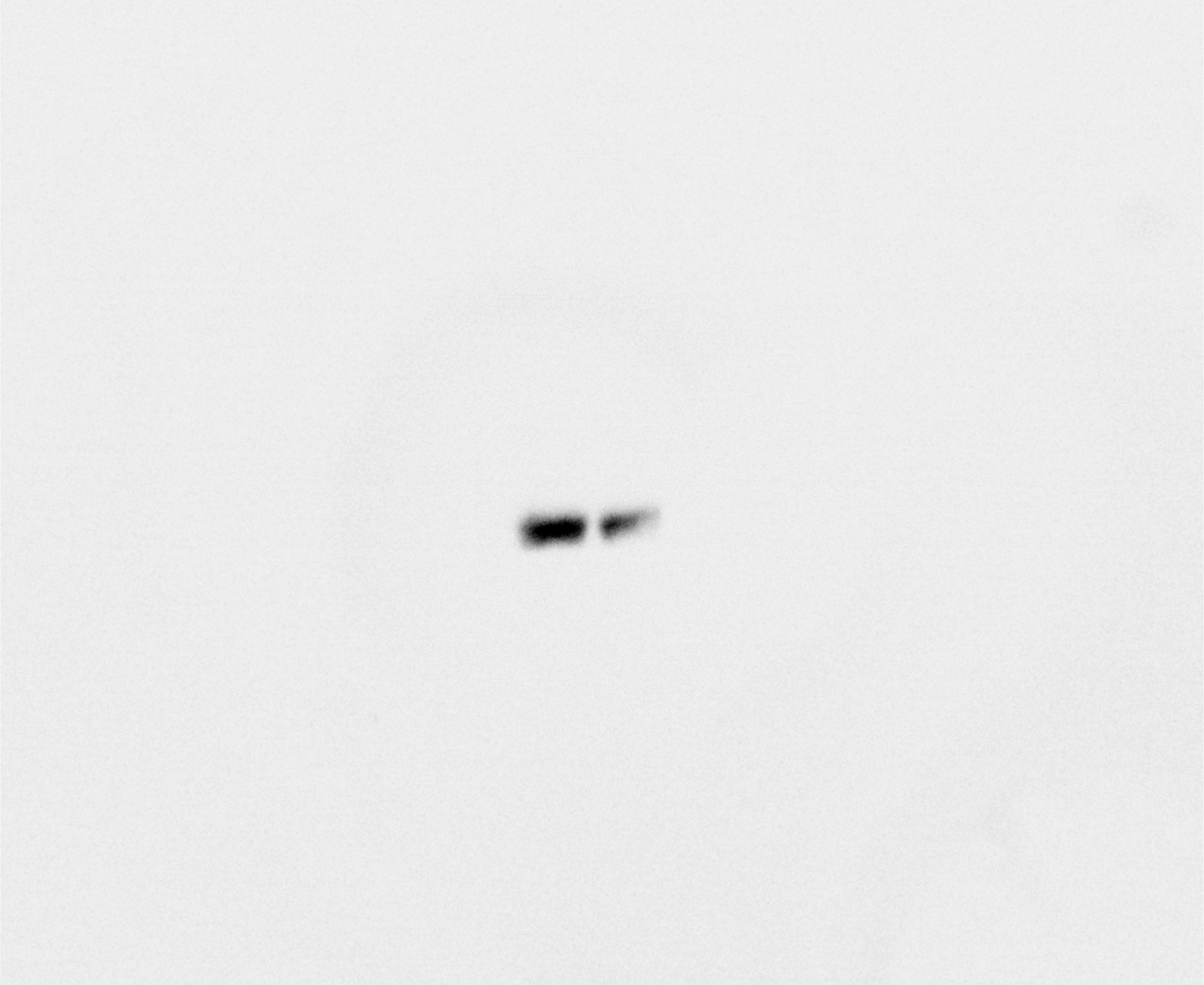

Figure 6G p2 STAT5

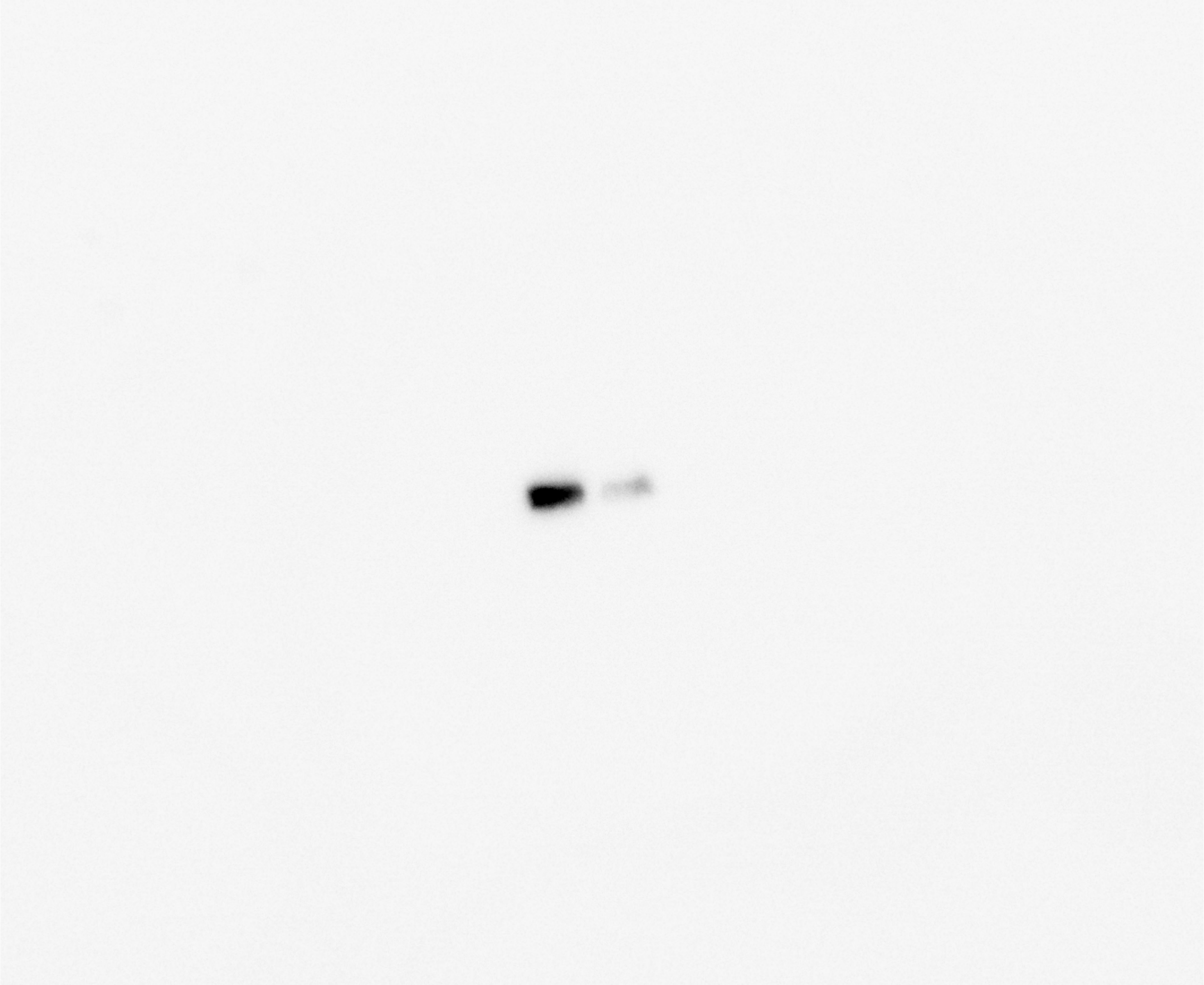

Figure 6G p2 GAPDH

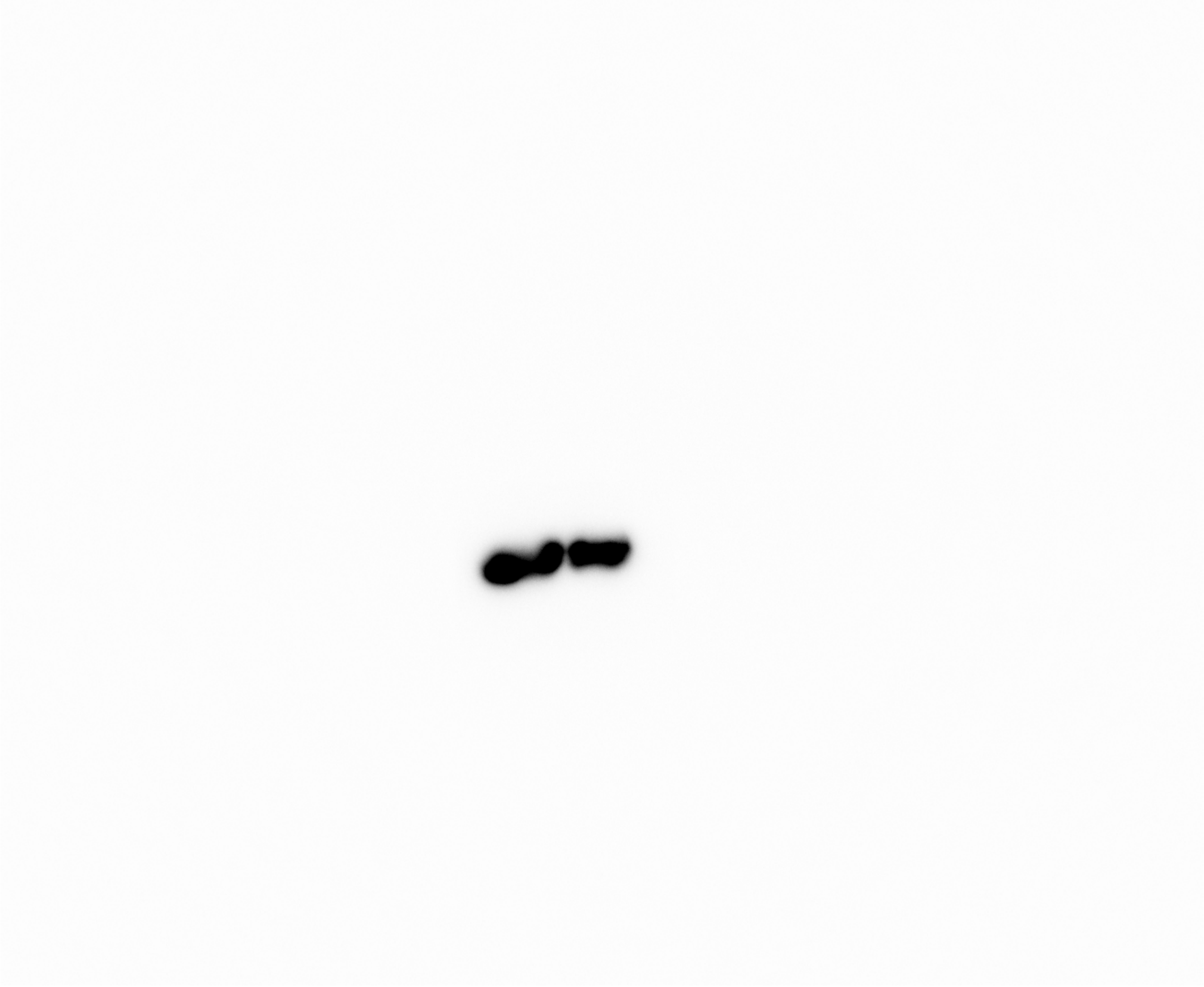

Figure 6G p2 H3

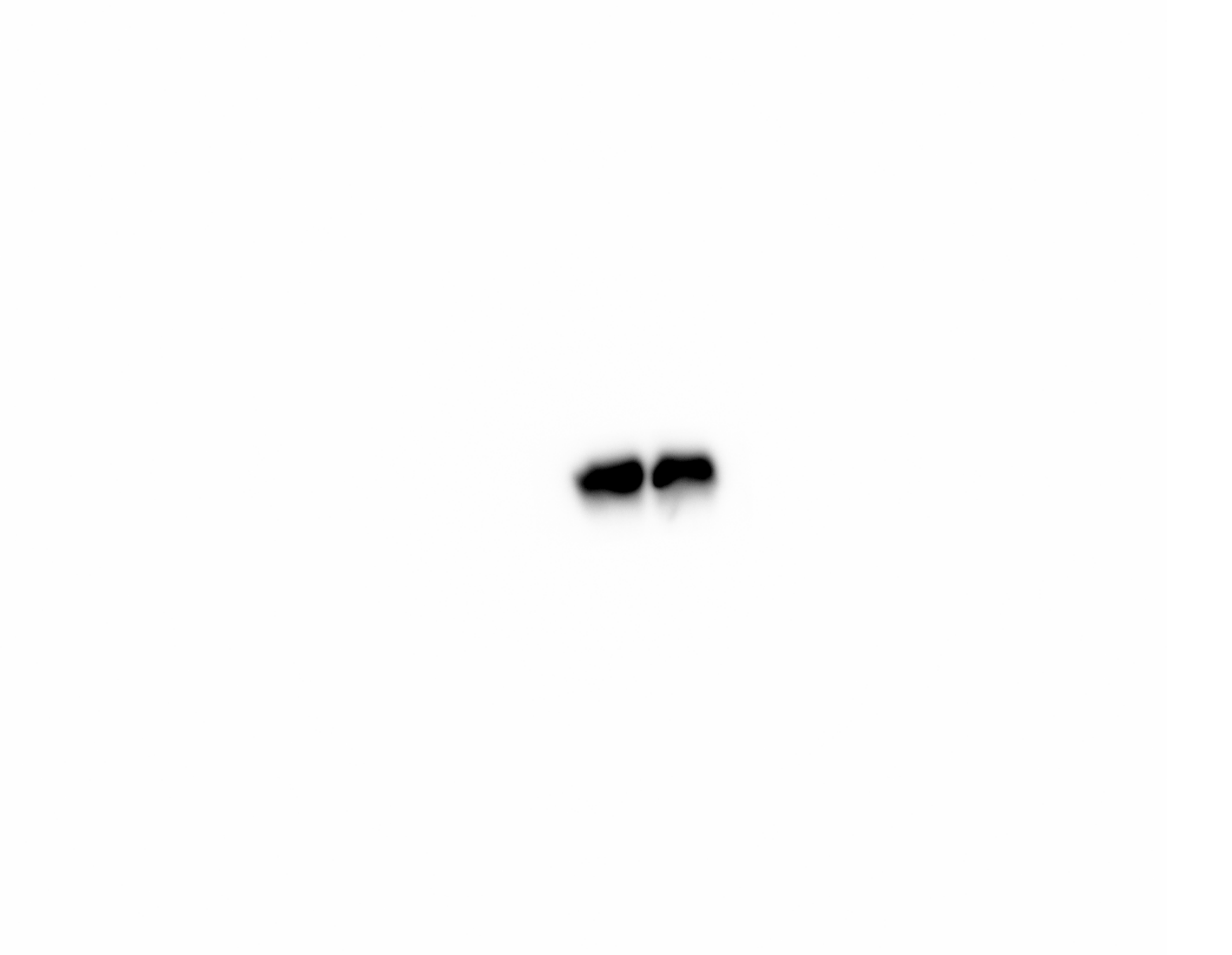

Figure 6G p2 H3K

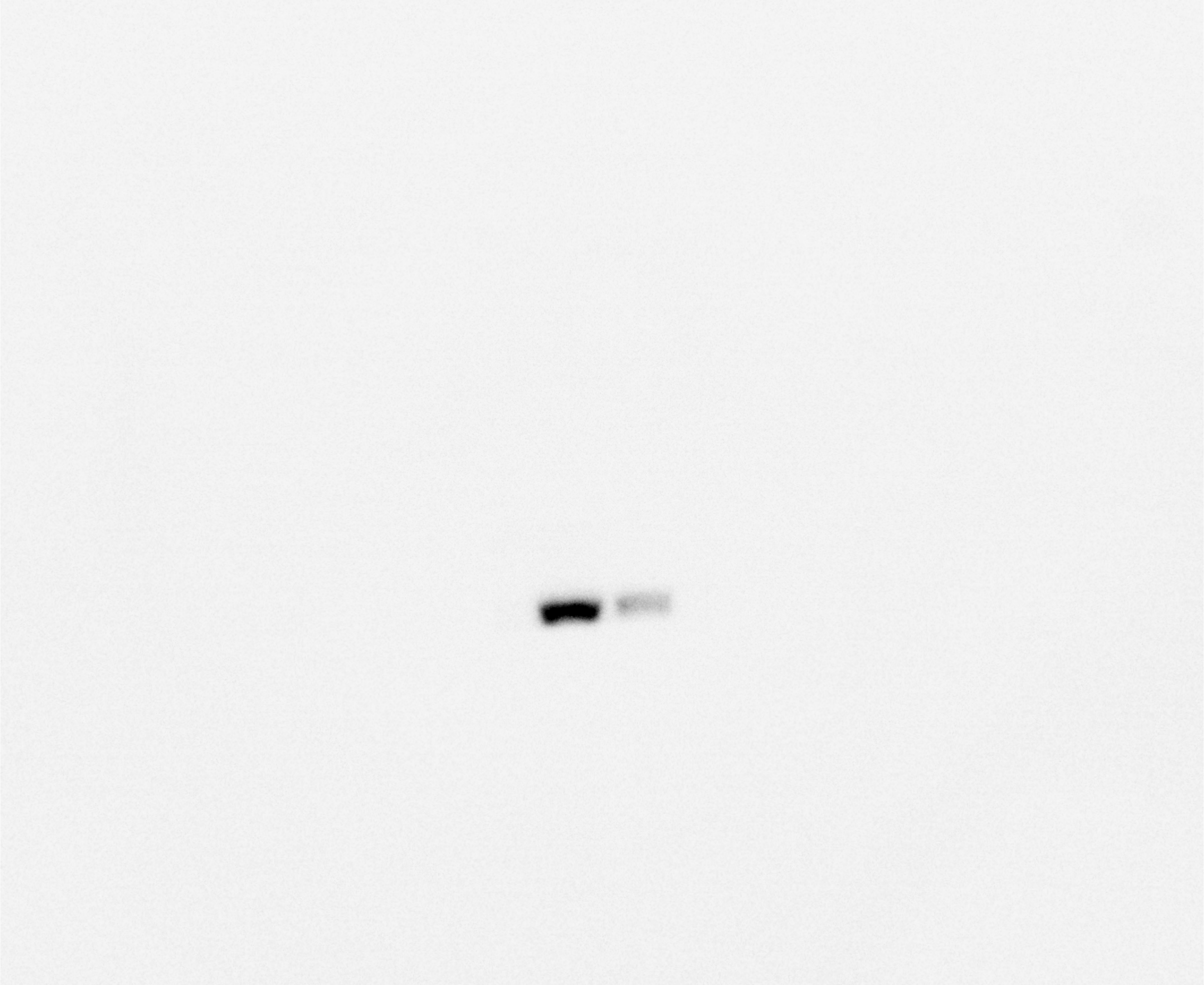

Figure 6G p3 STAT5

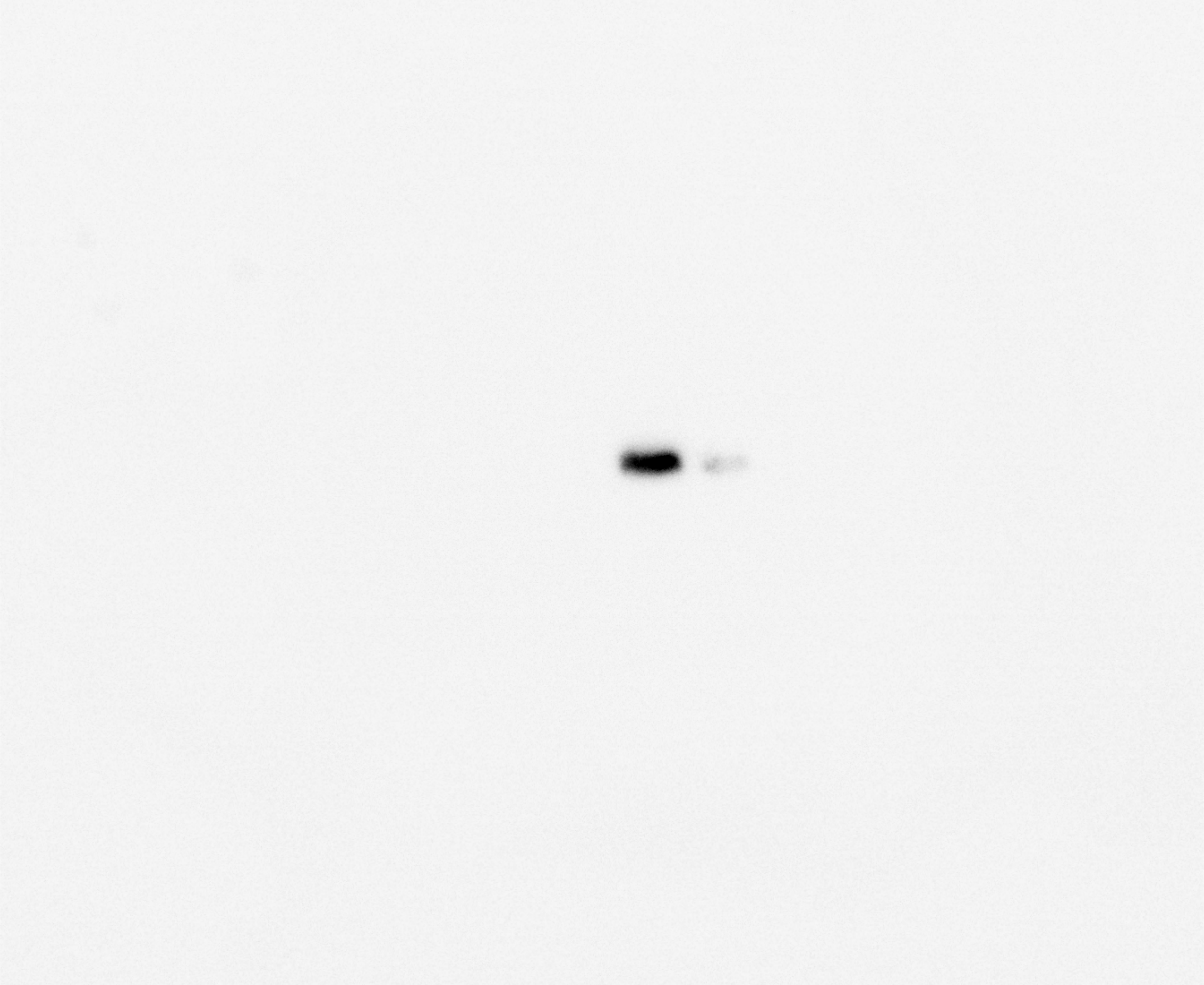

Figure 6G p3 GAPDH

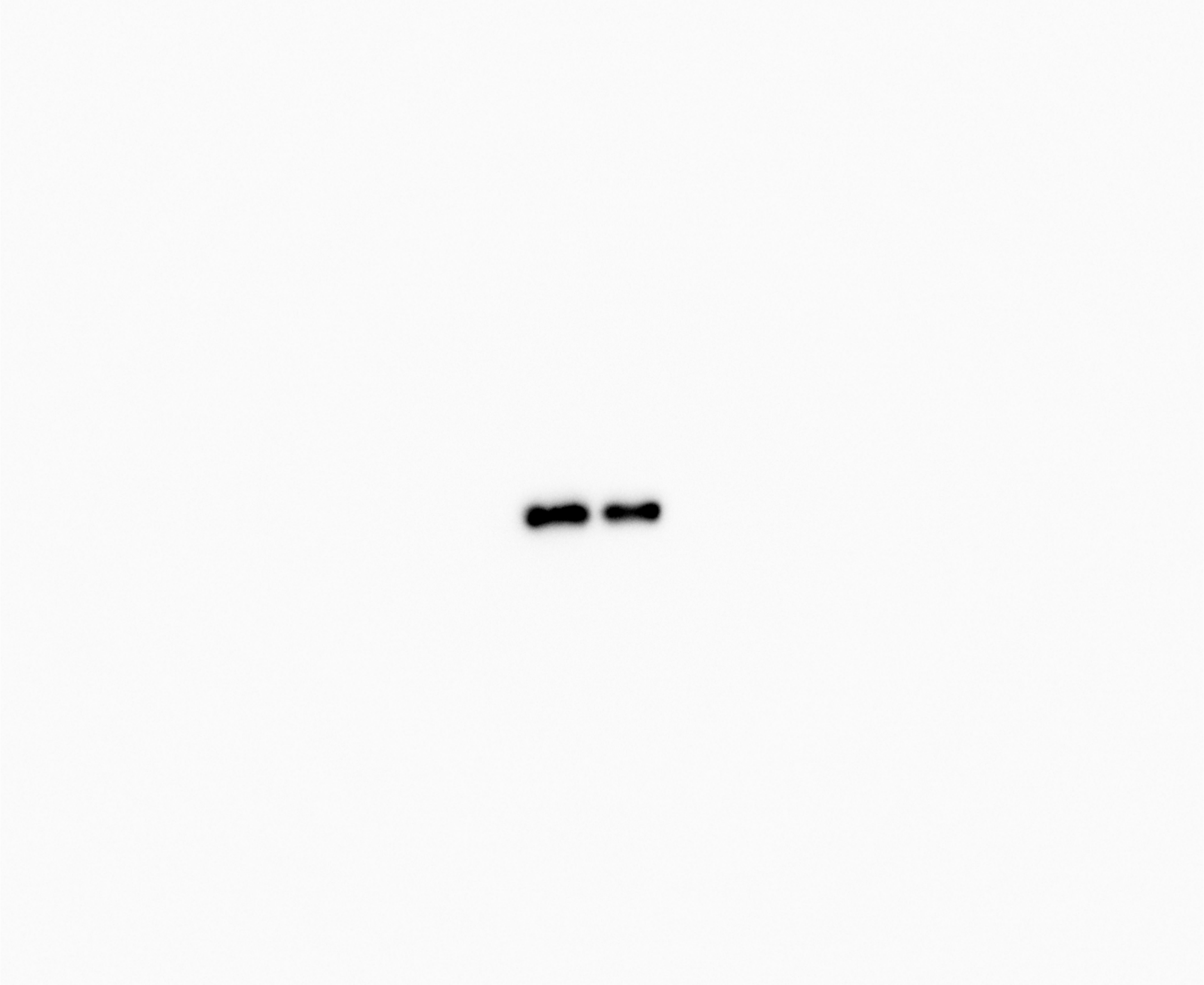

Figure 6G p3 H3

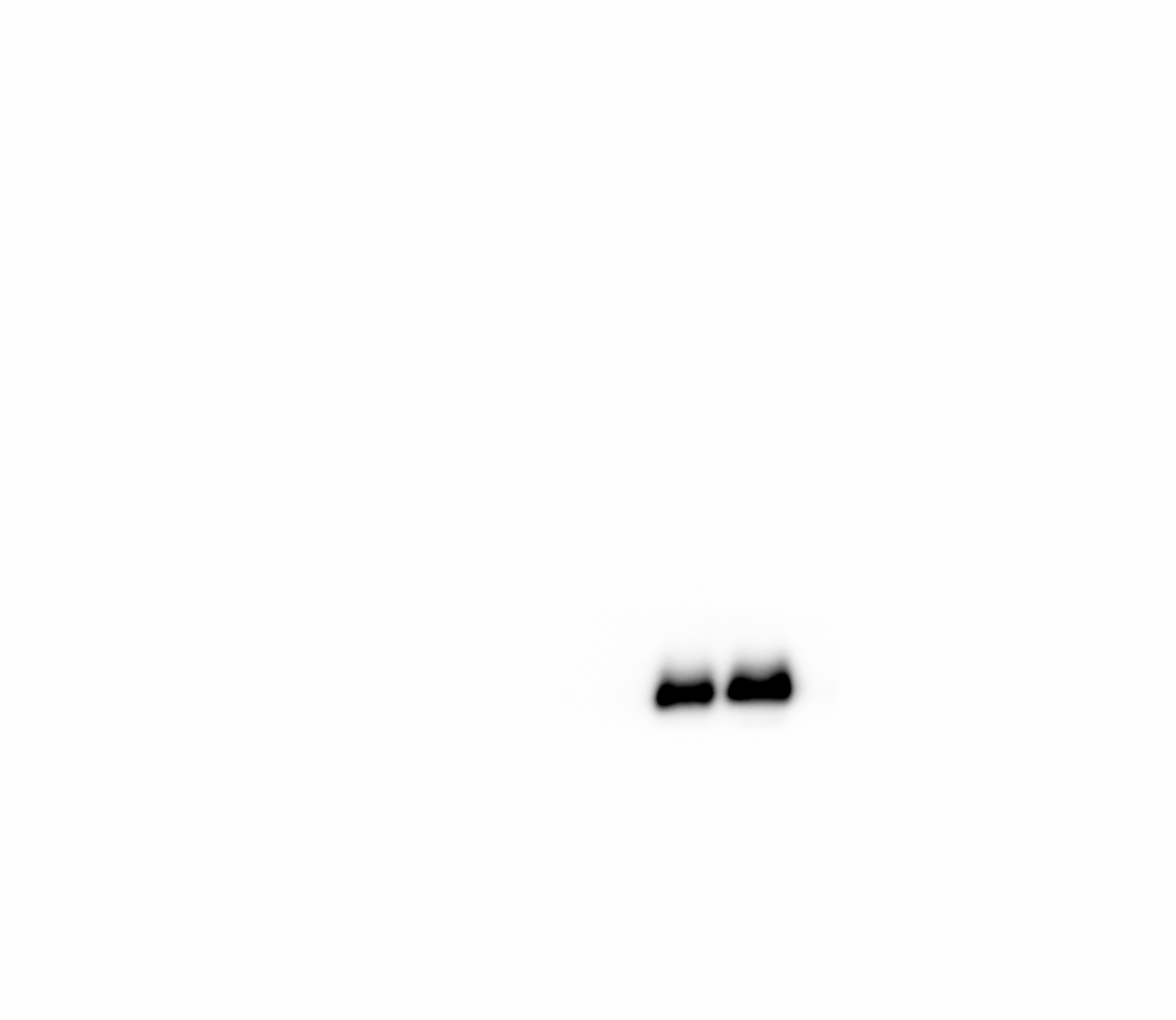

Figure 6G p3 H3K

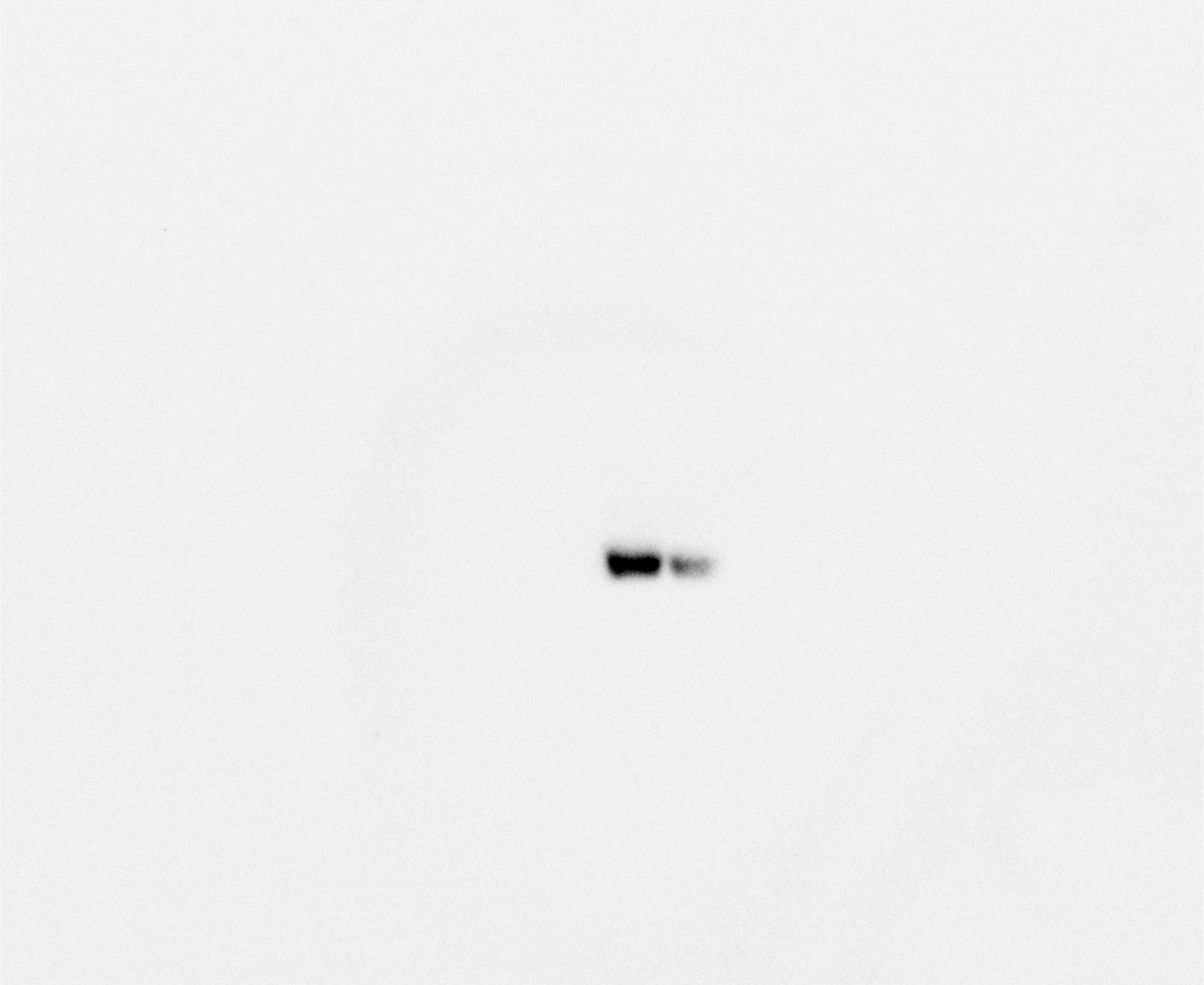

Figure 6G p4 STAT5

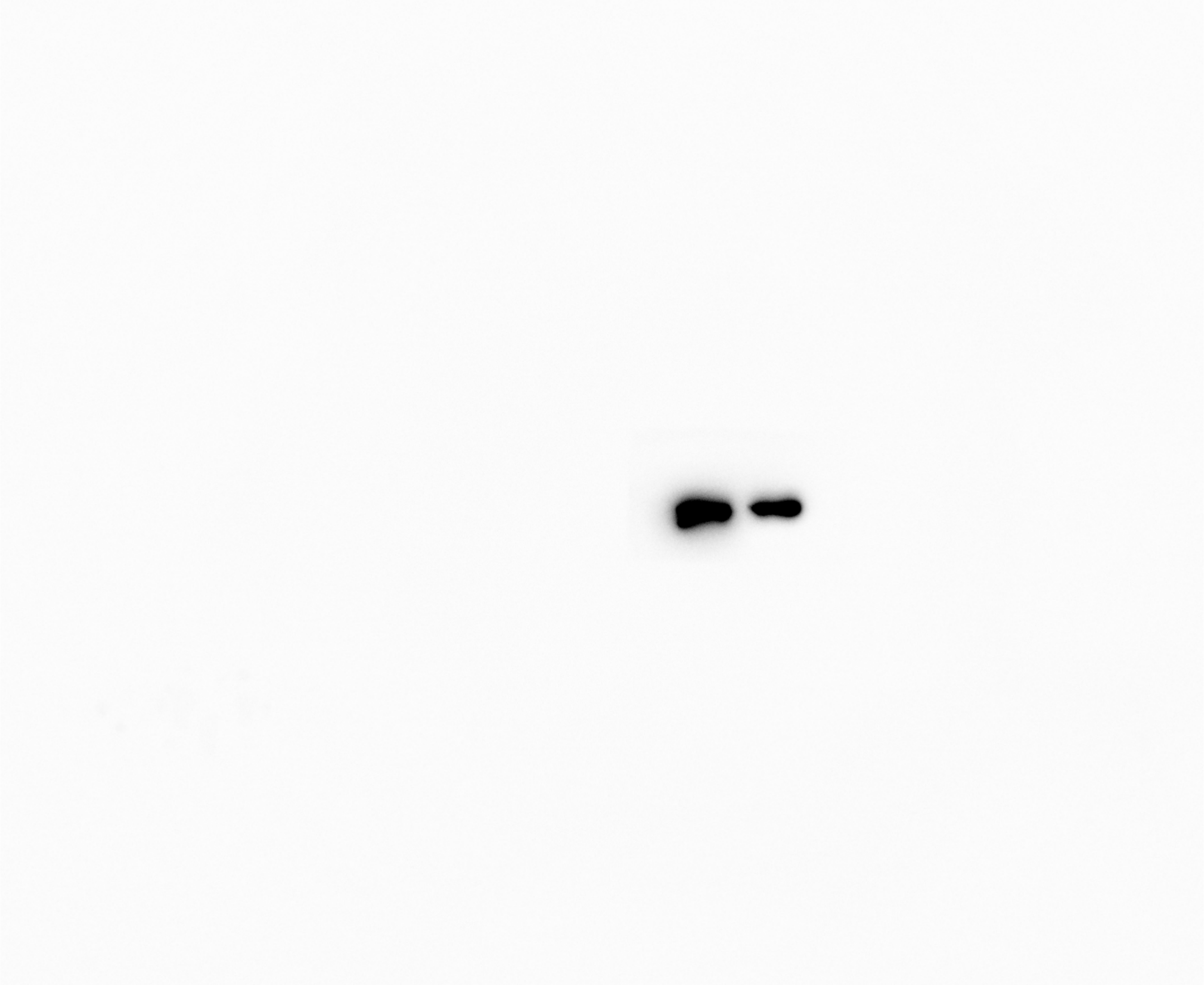

Figure 6G p4 GAPDH

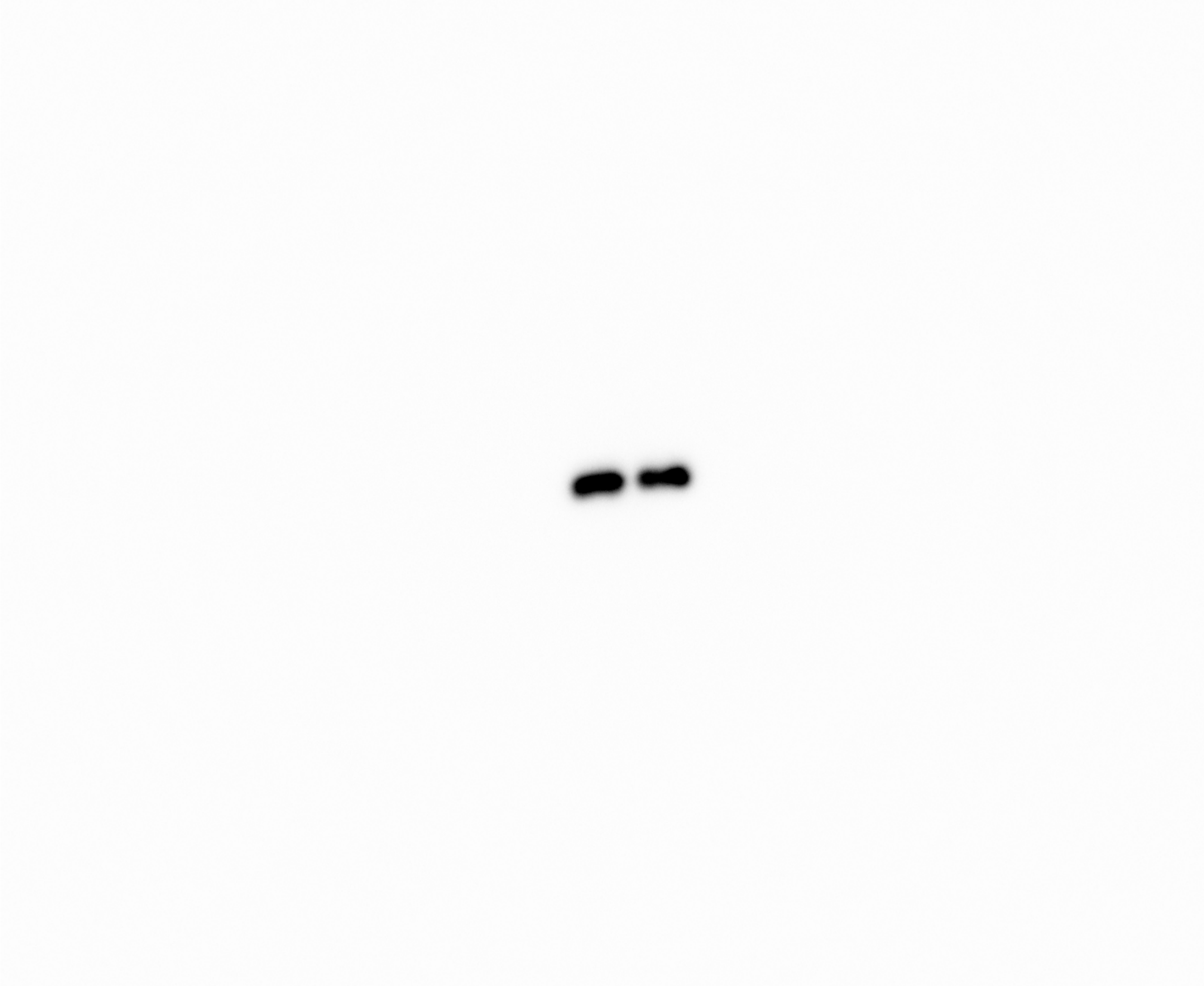

Figure 6G p4 H3

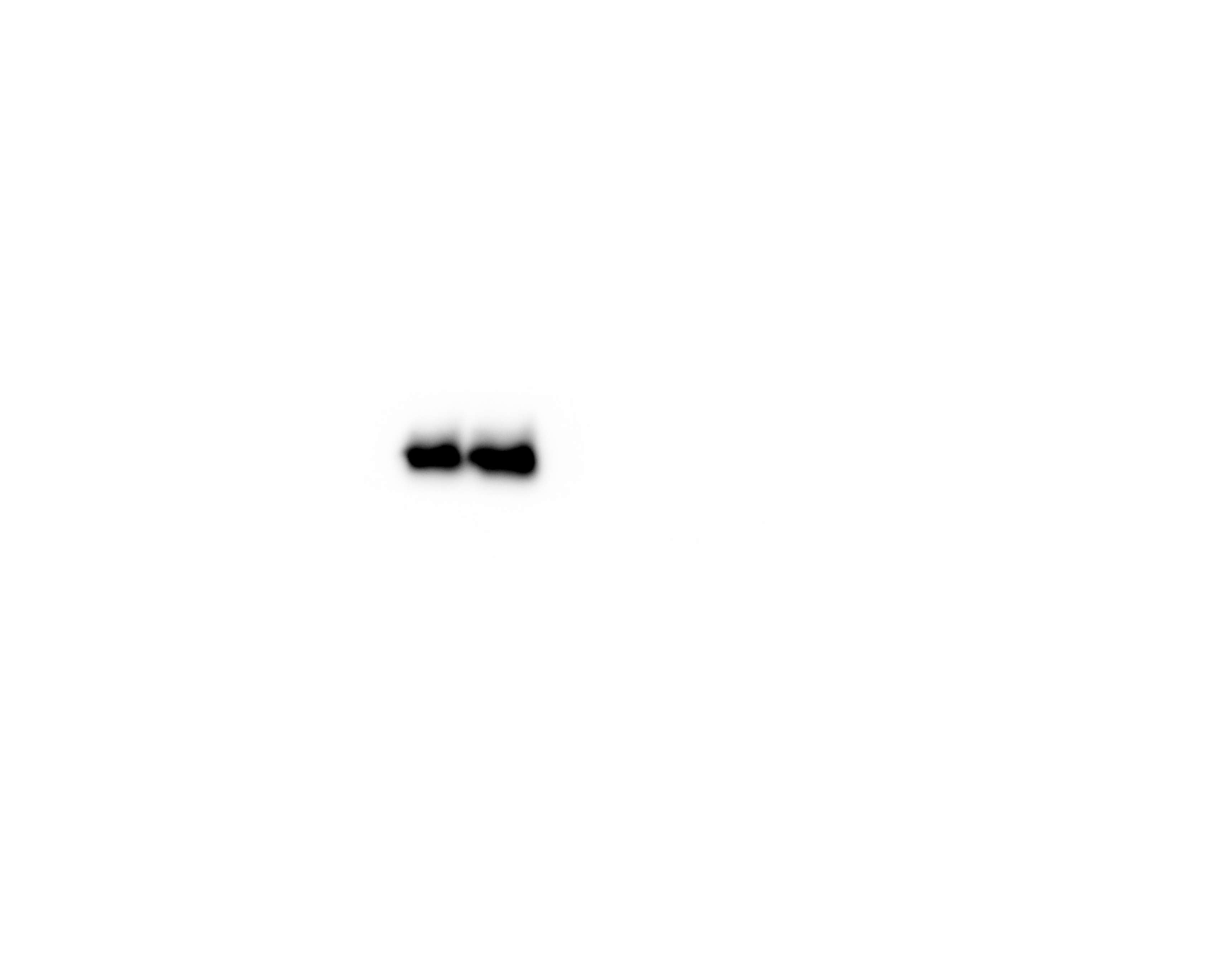

Figure 6G p4 H3K

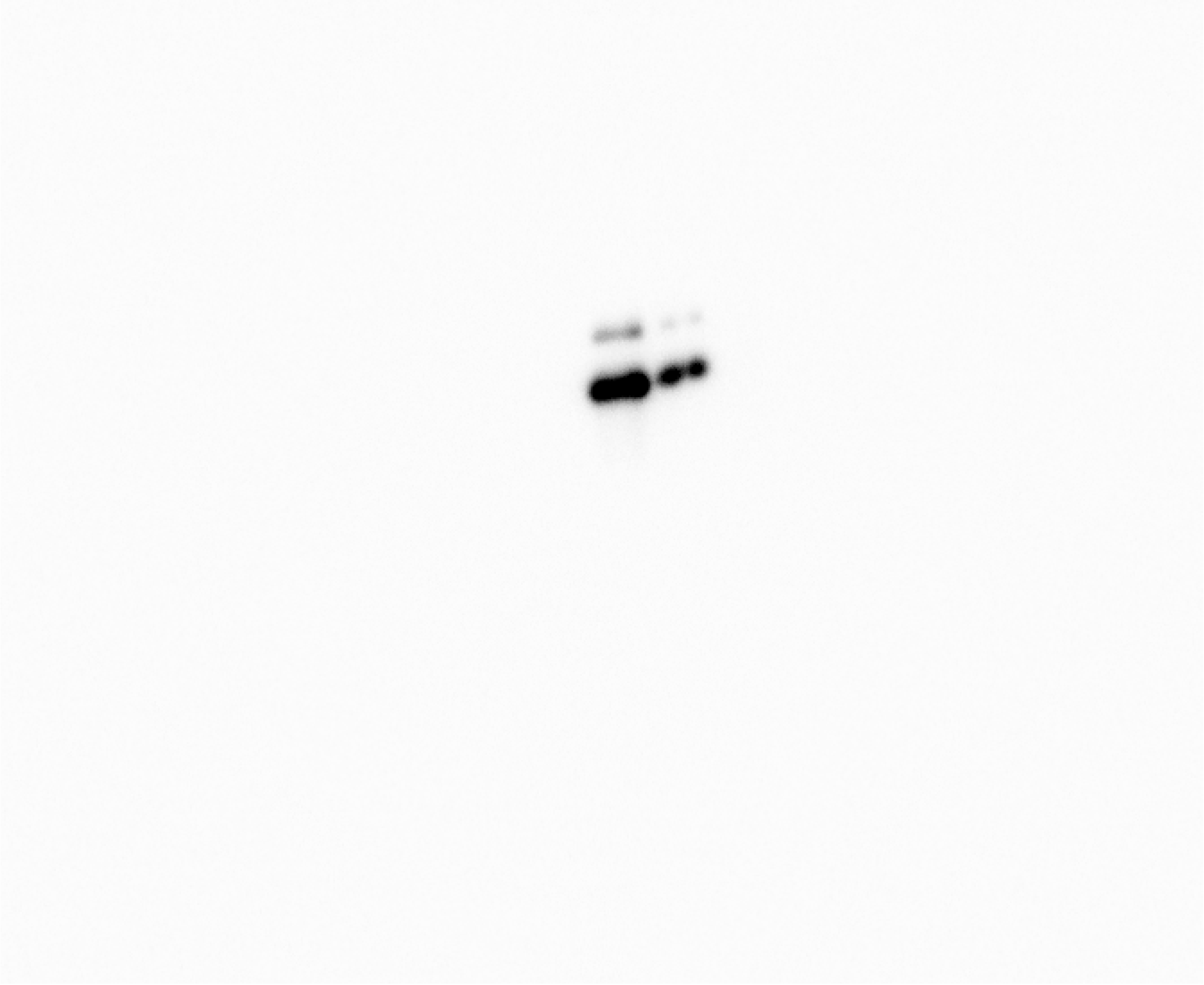

Figure 6G p5 STAT5

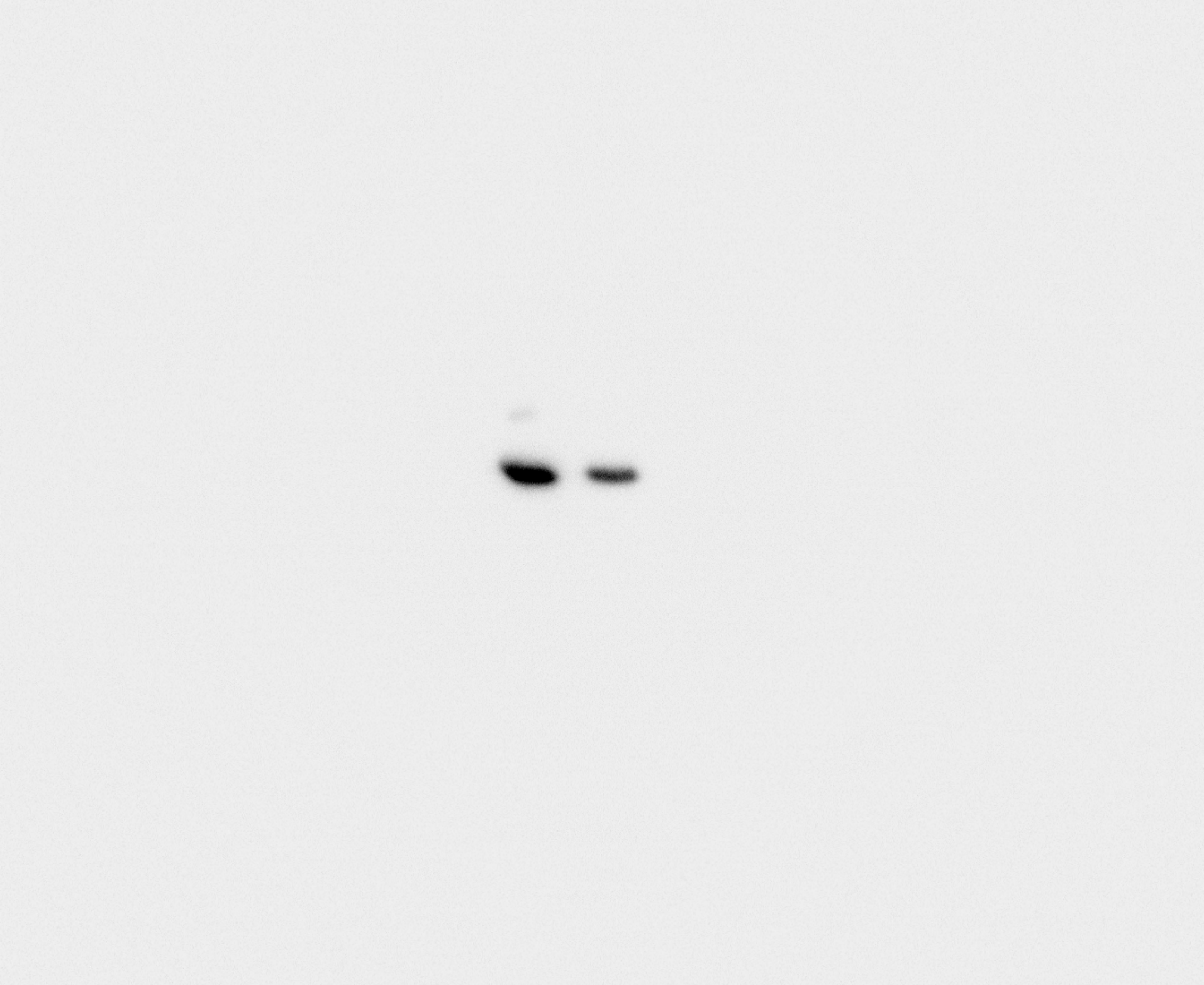

Figure 6G p5 GAPDH

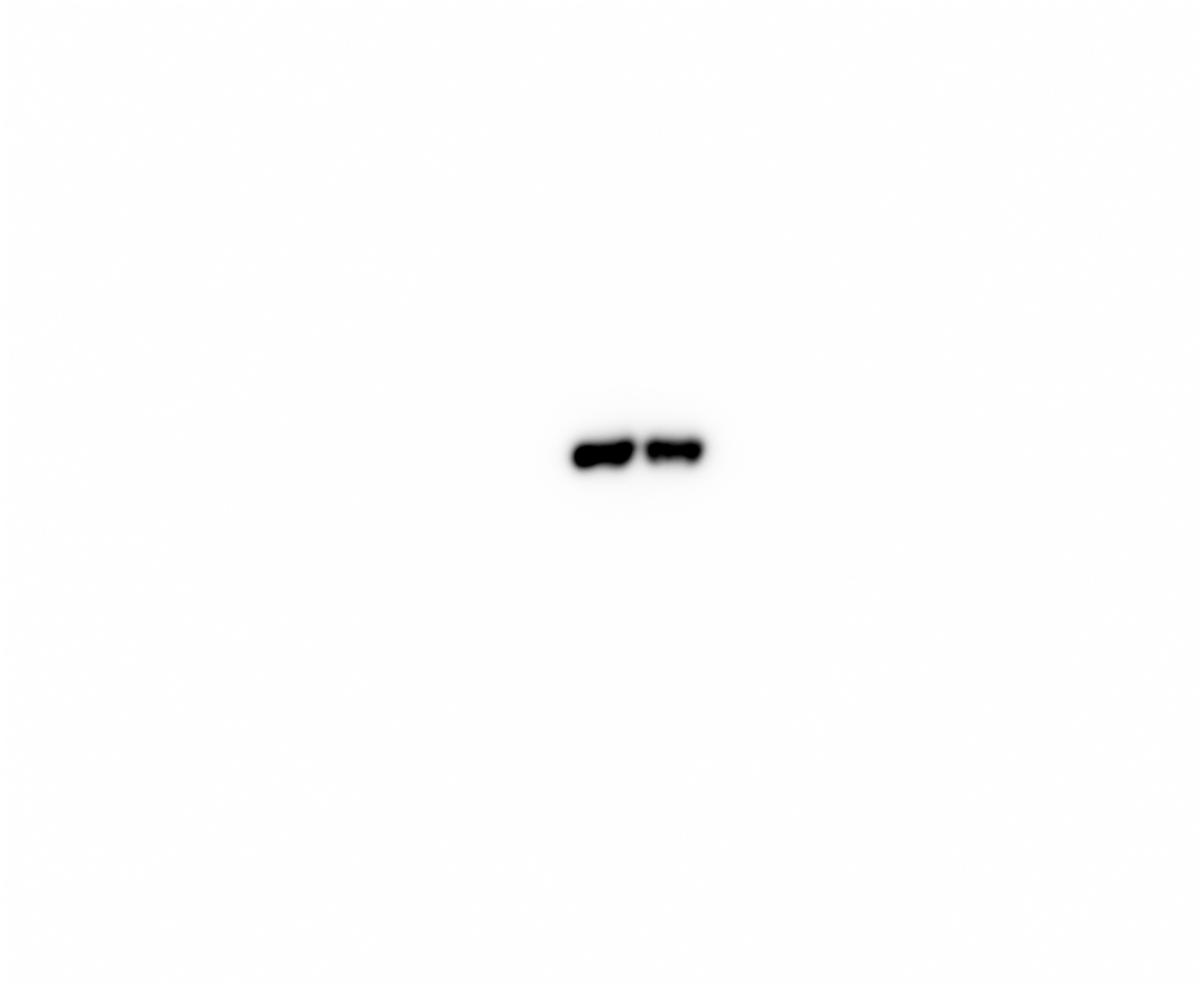

Figure 6G p5 H3

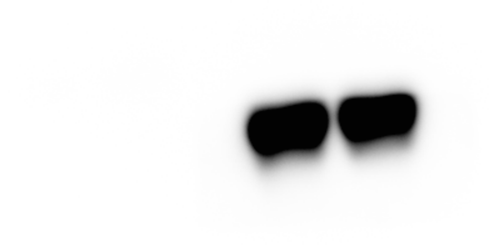

Figure 6G p5 H3K

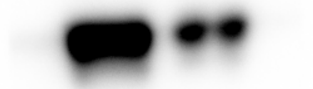

Figure 6H STAT5 p4

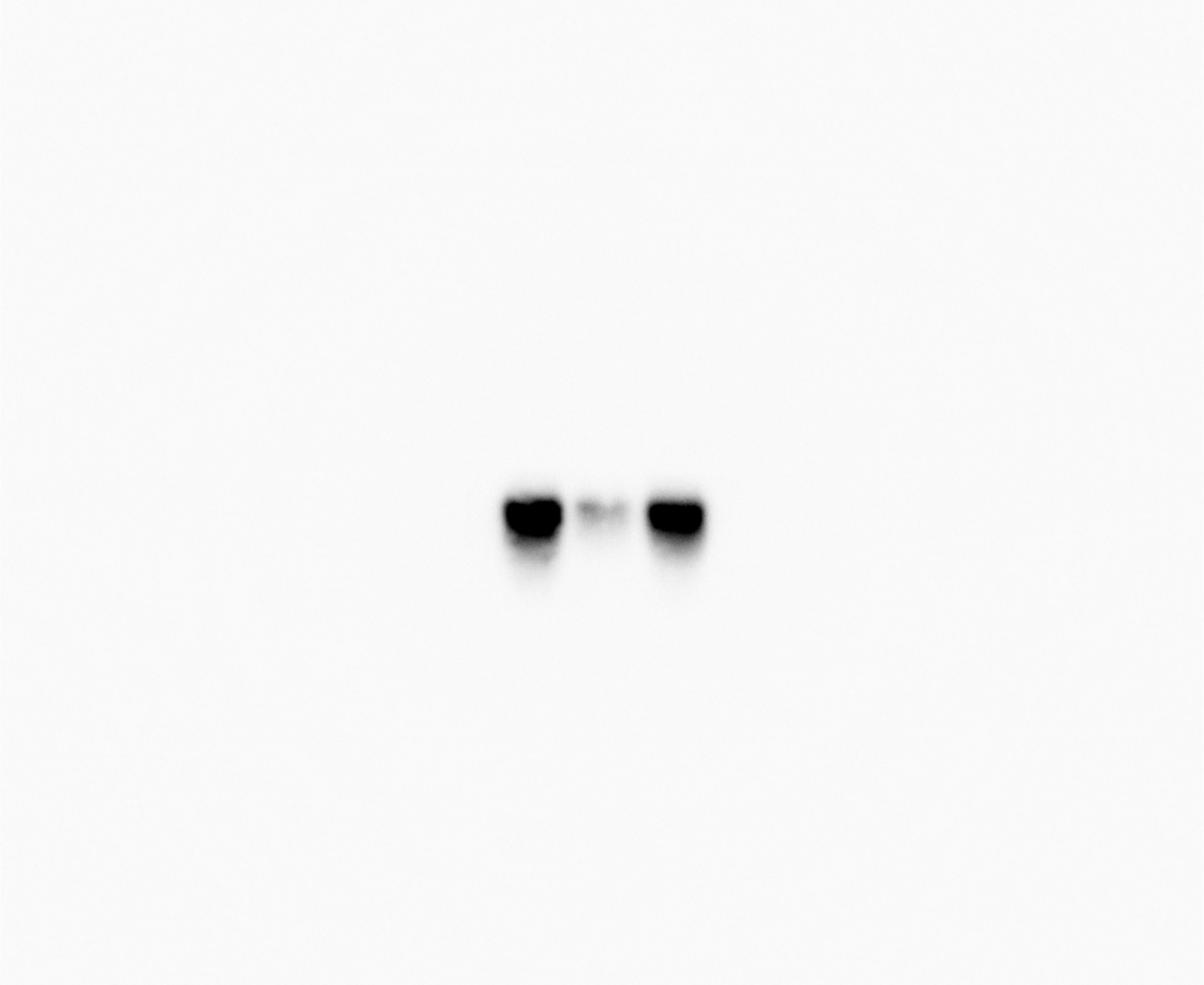

Figure 6H GAPDH p1

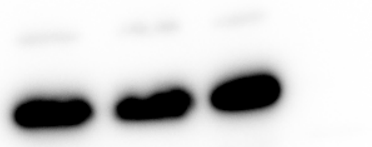

Figure 6H GAPDH p4

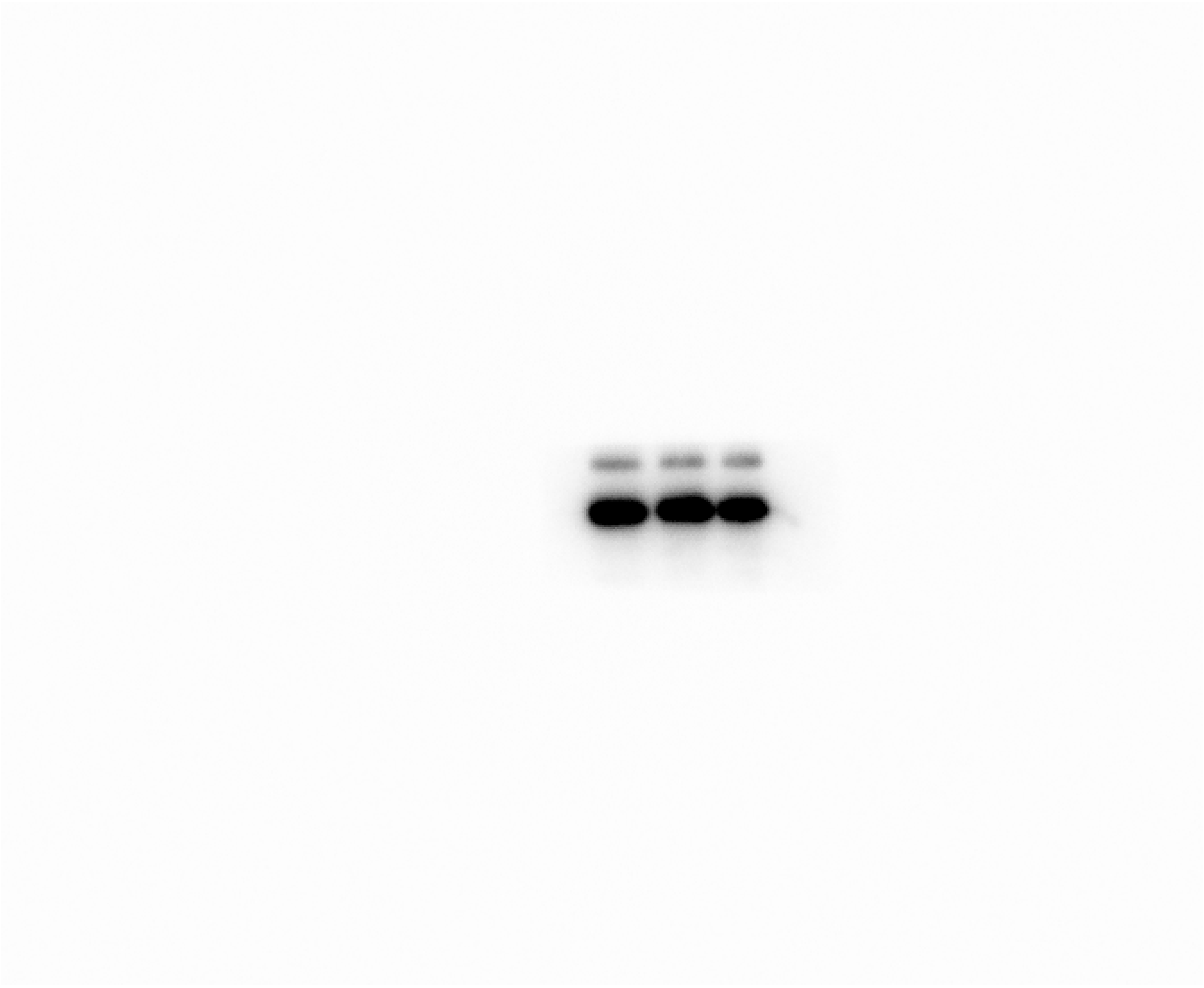

Figure 6H H3 p1

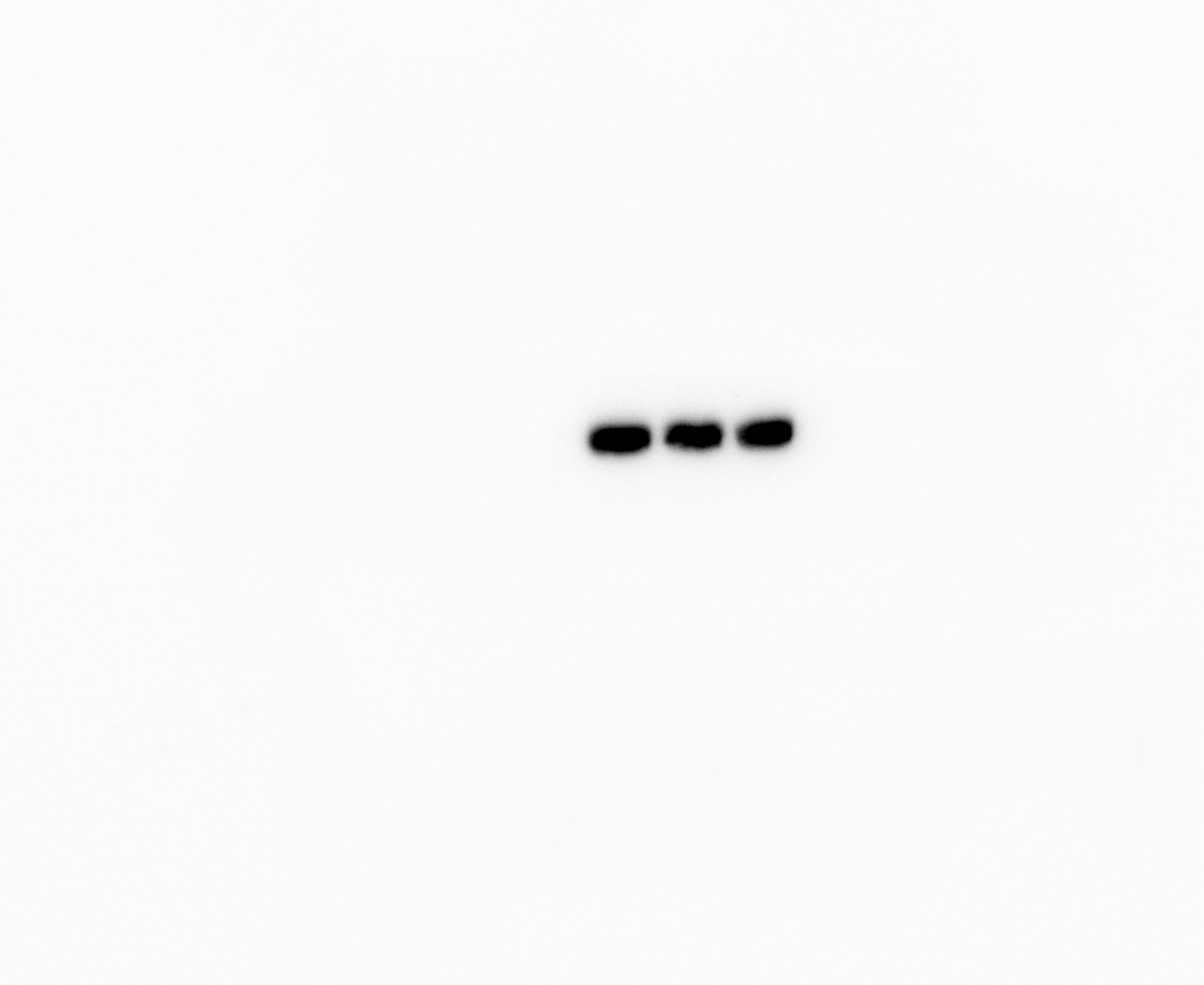

Figure 6H H3 p4

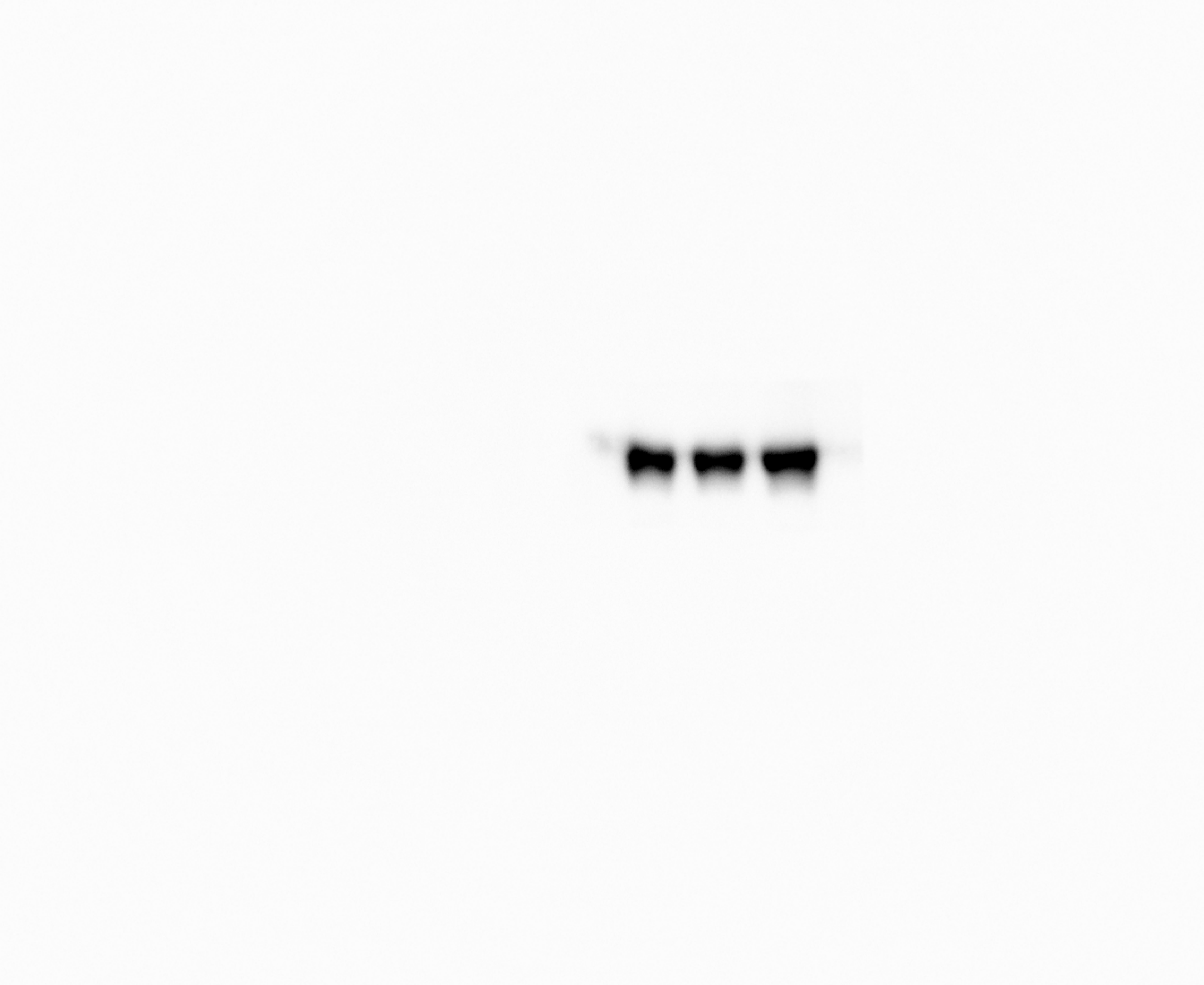

Figure 6H H3K p1

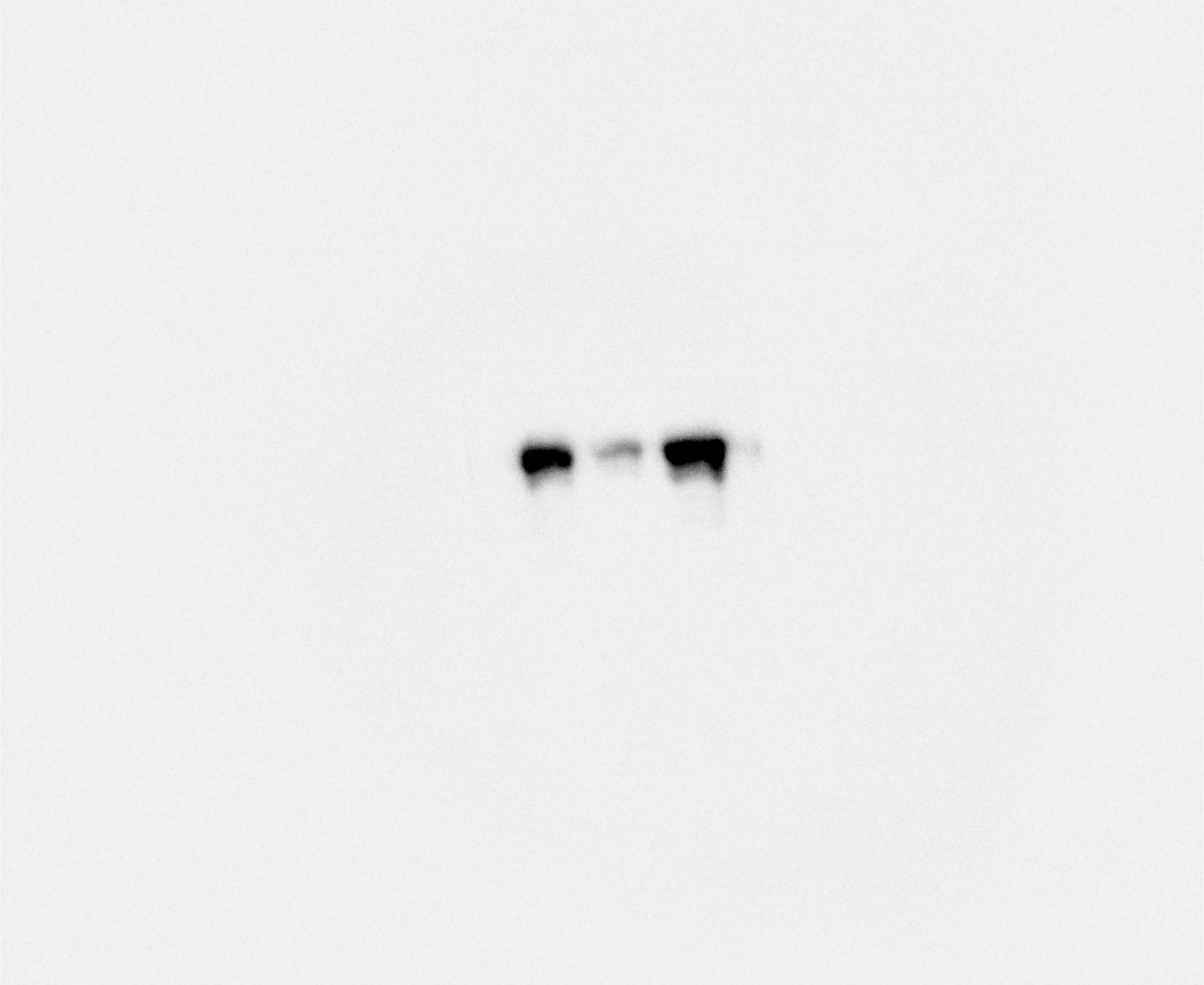

Figure 6H H3K p4

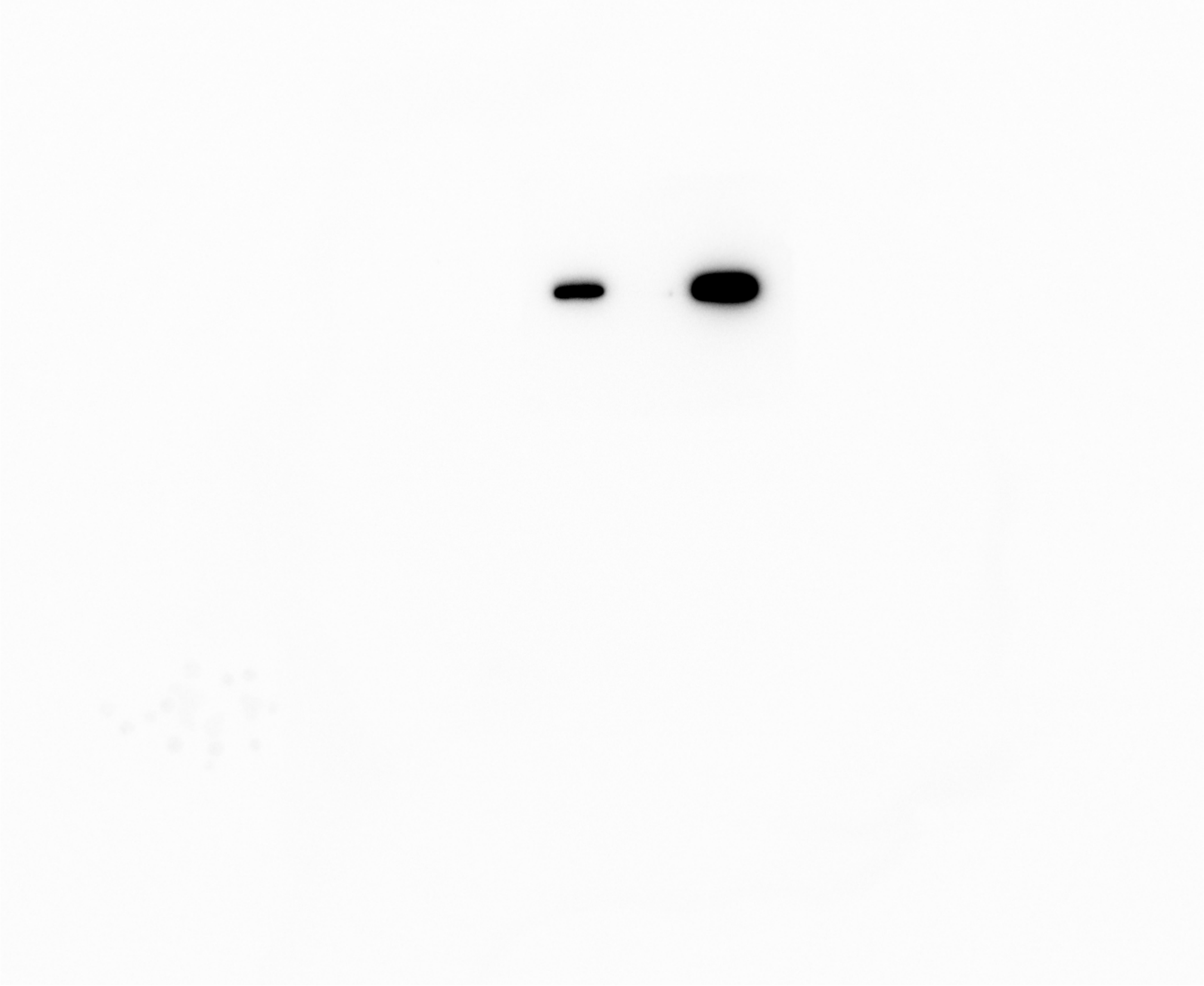

Figure 6H STAT5 p1

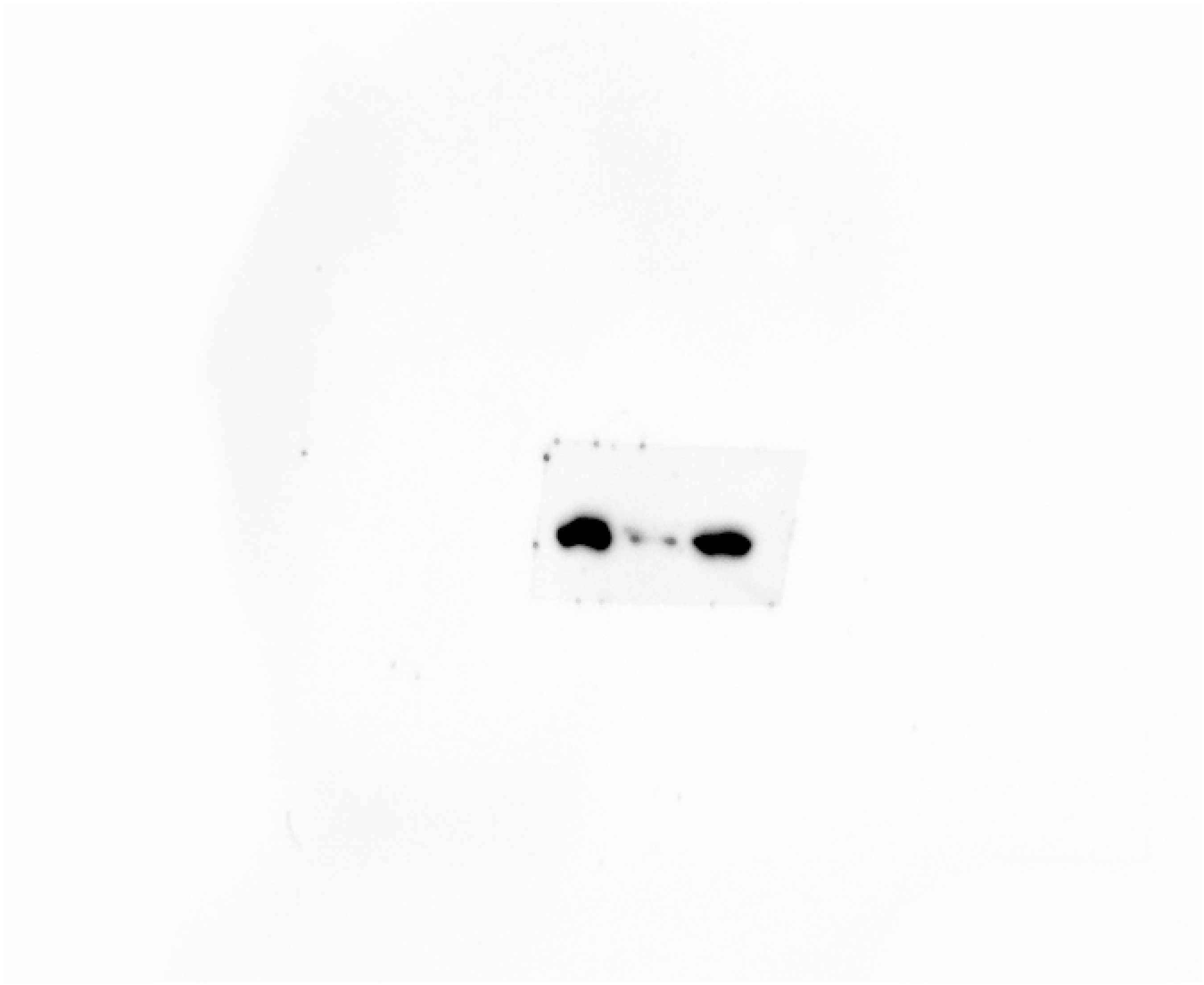

Figure 7C SLC43A2

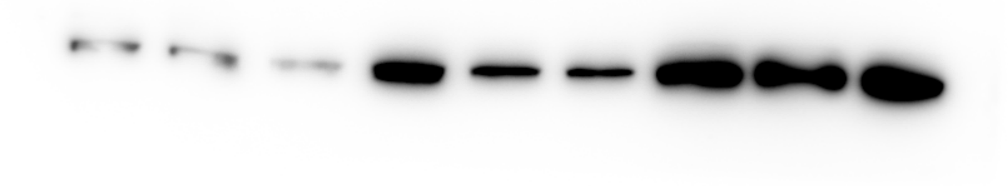

Figure 7C GAPDH

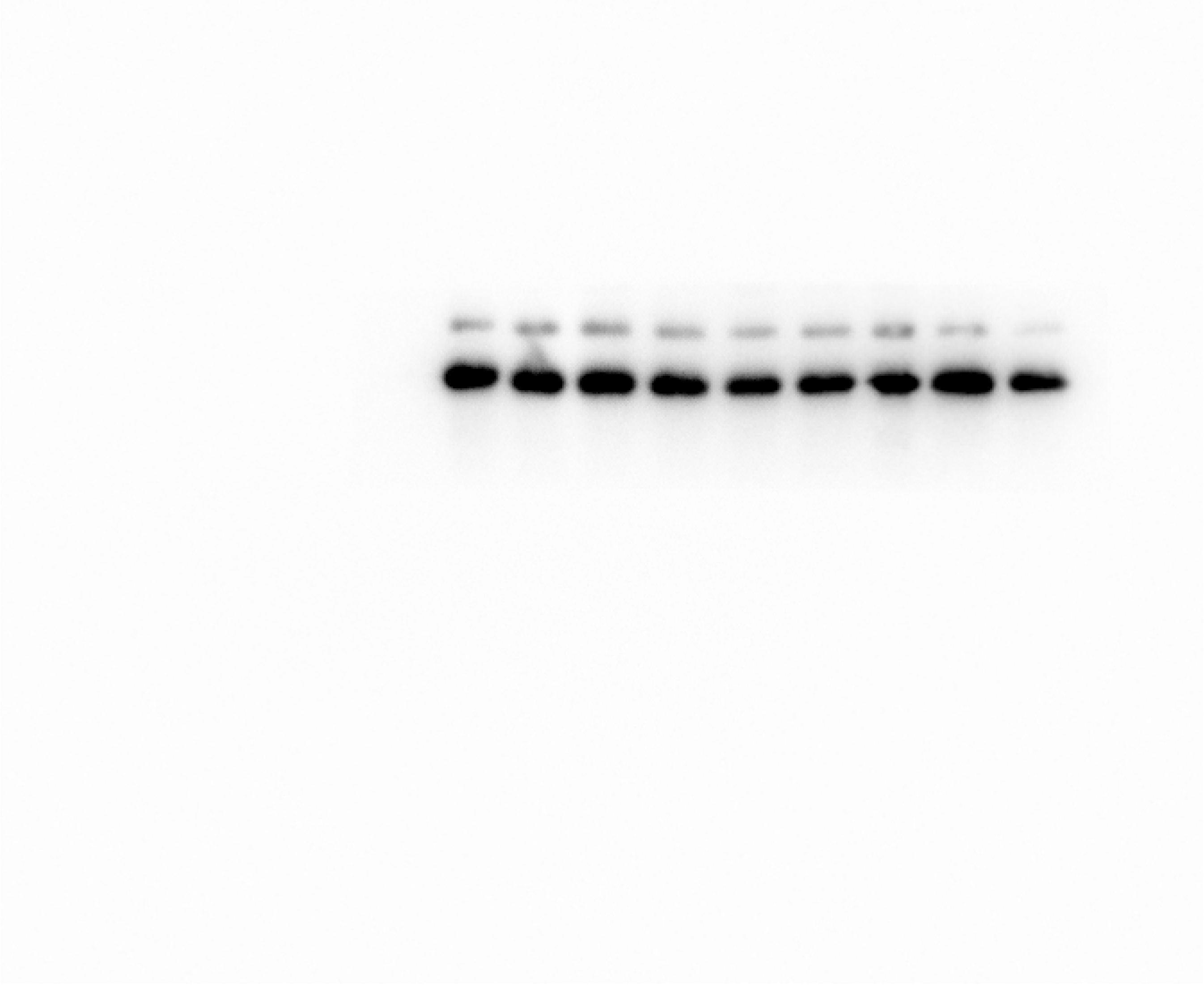

Figure 7C SLC7A6

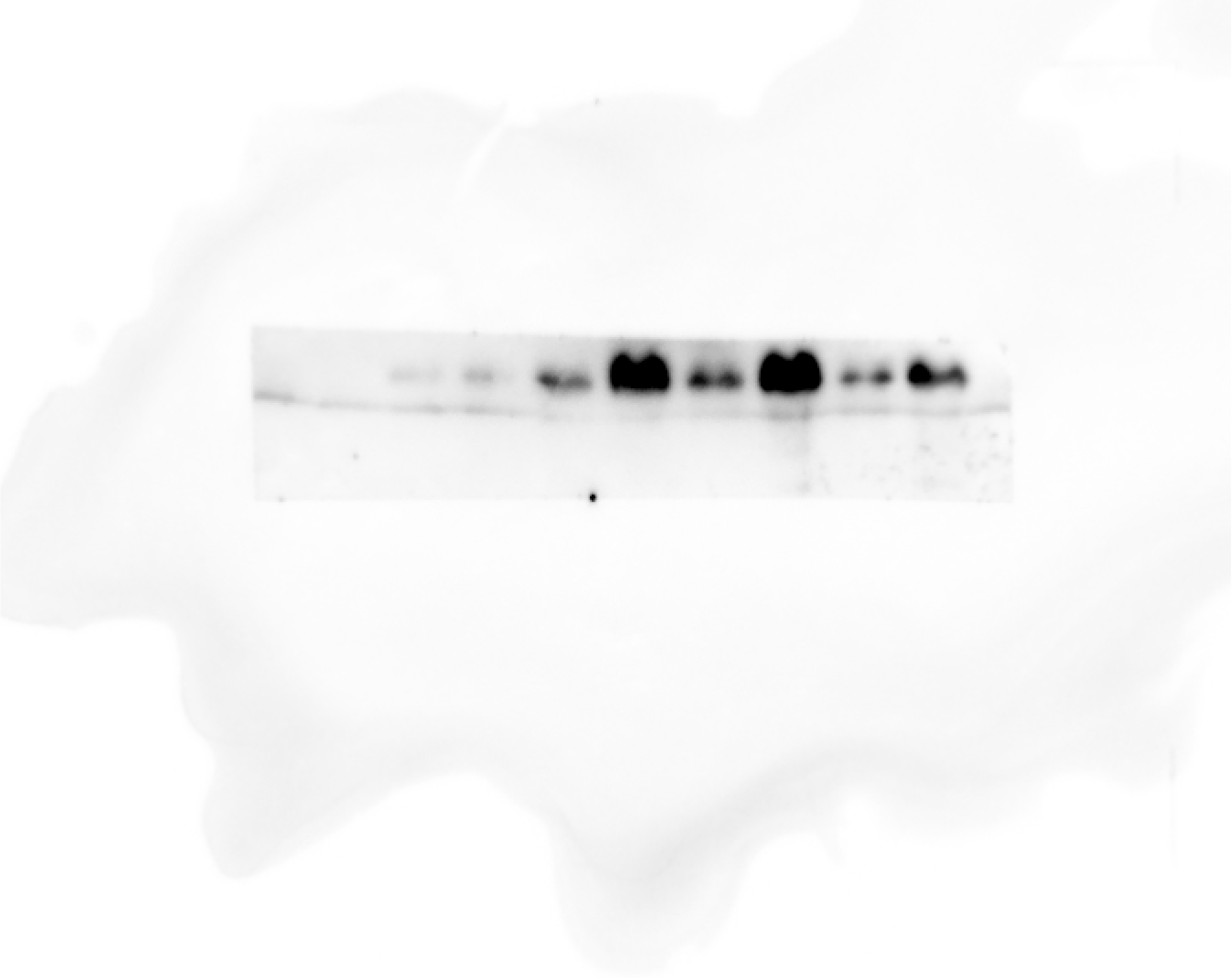

Figure 7E SLC7A6

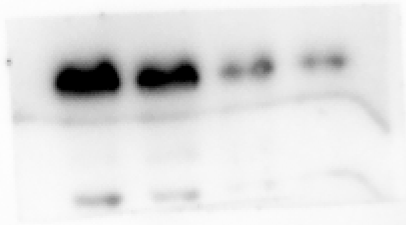

Figure 7E GAPDH

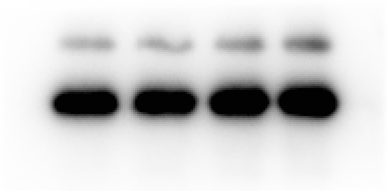

Figure 7F STAT5 p4

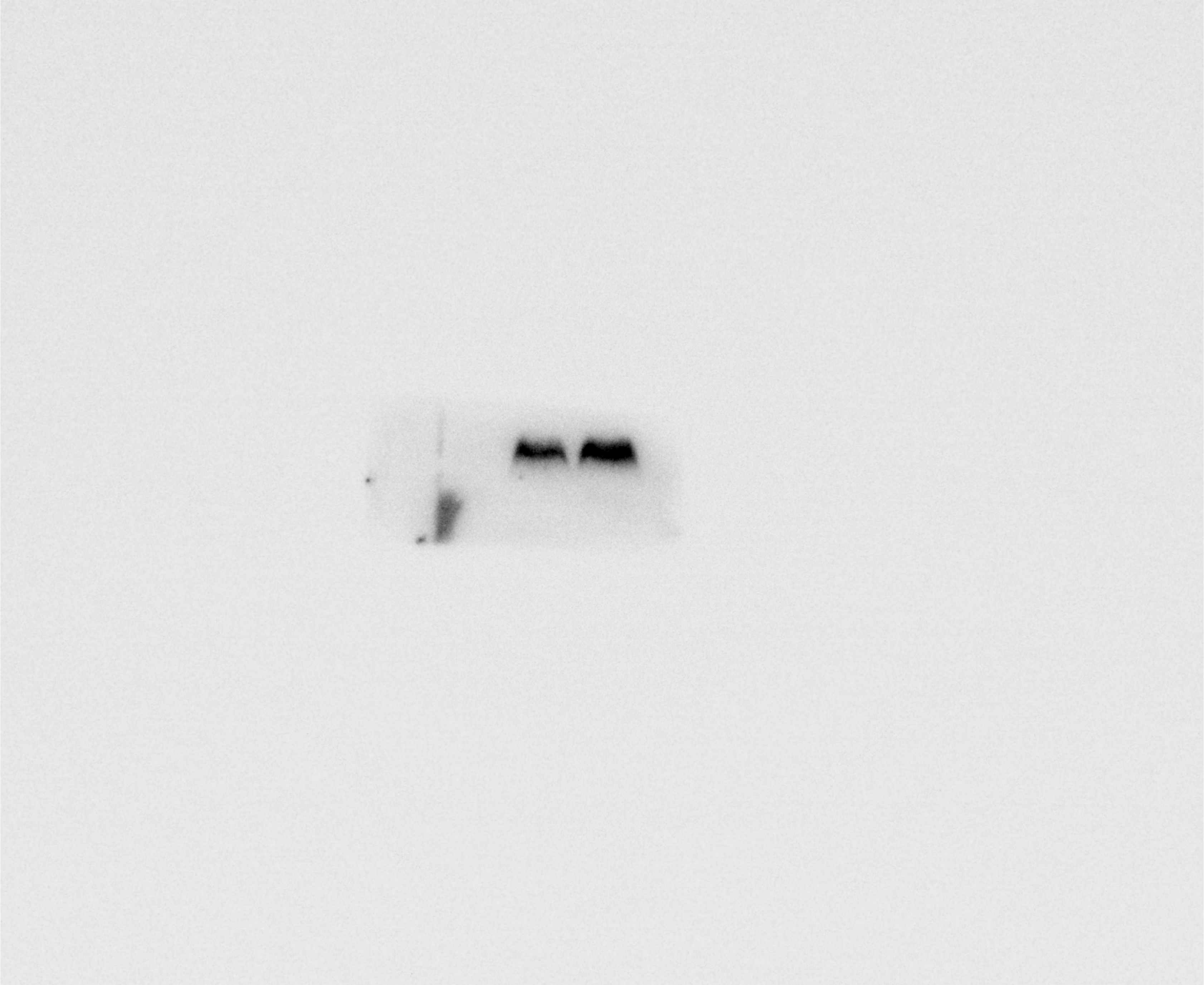

Figure 7F GAPDH p1

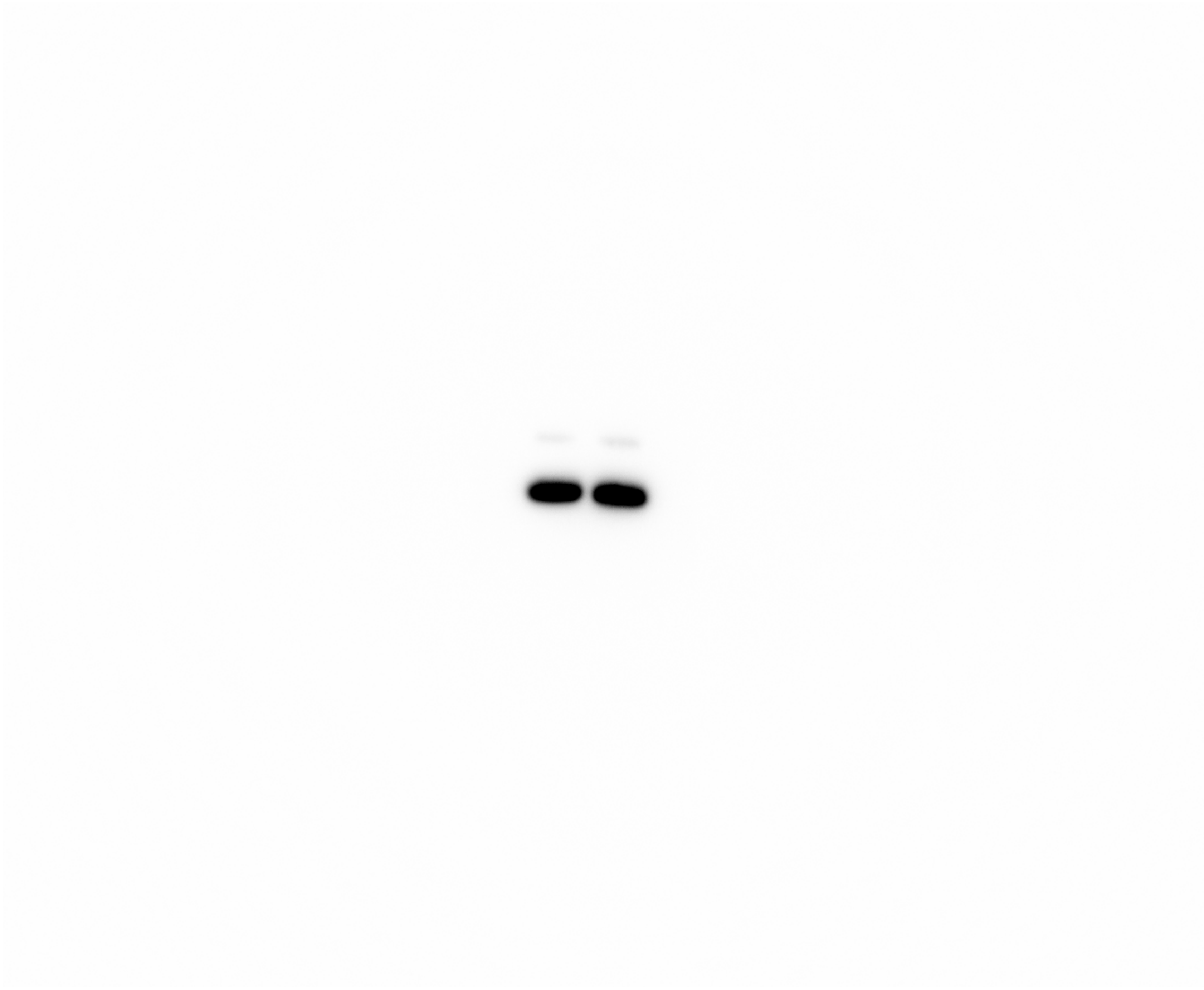

Figure 7F GAPDH p4

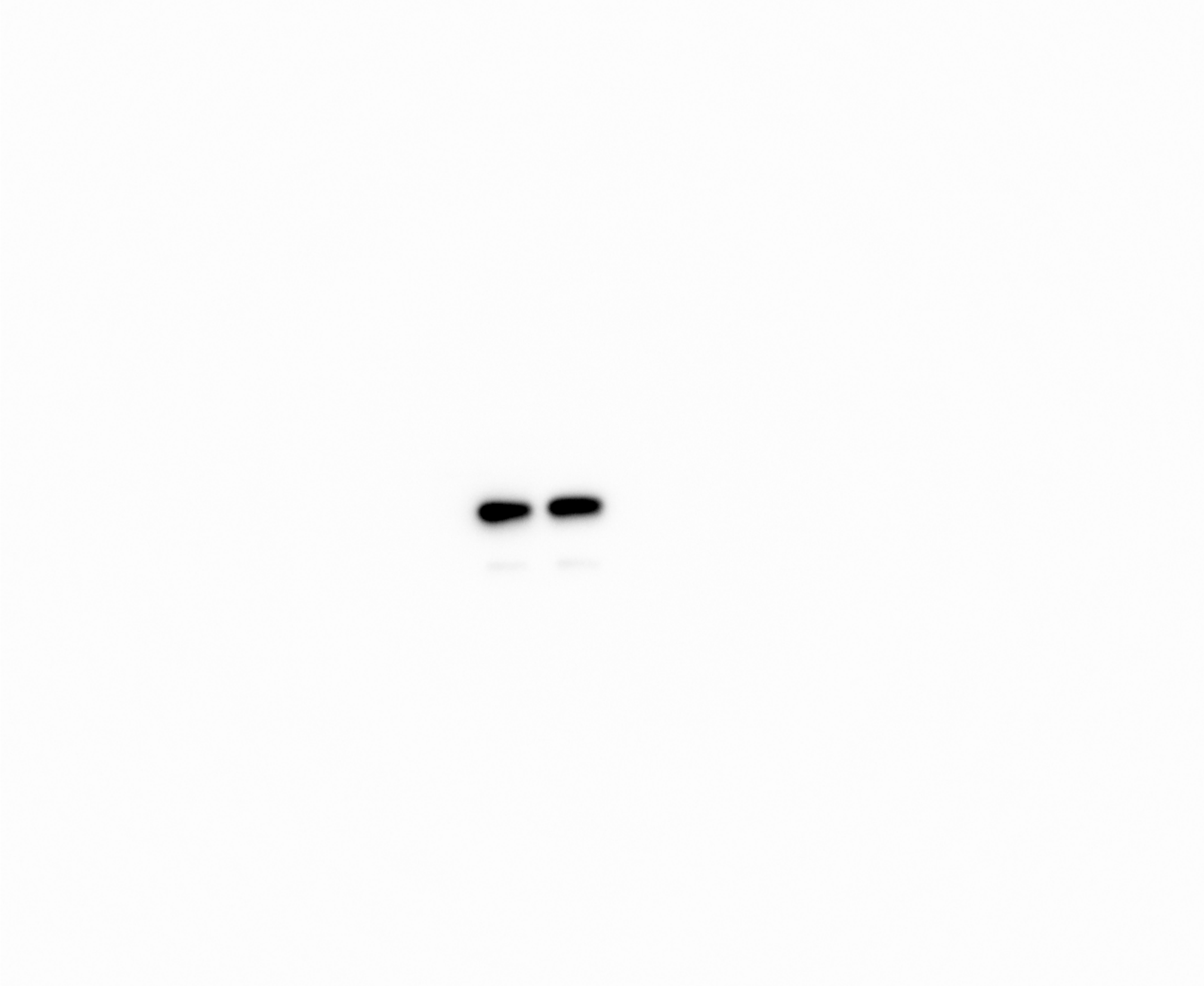

Figure 7F H3 p1

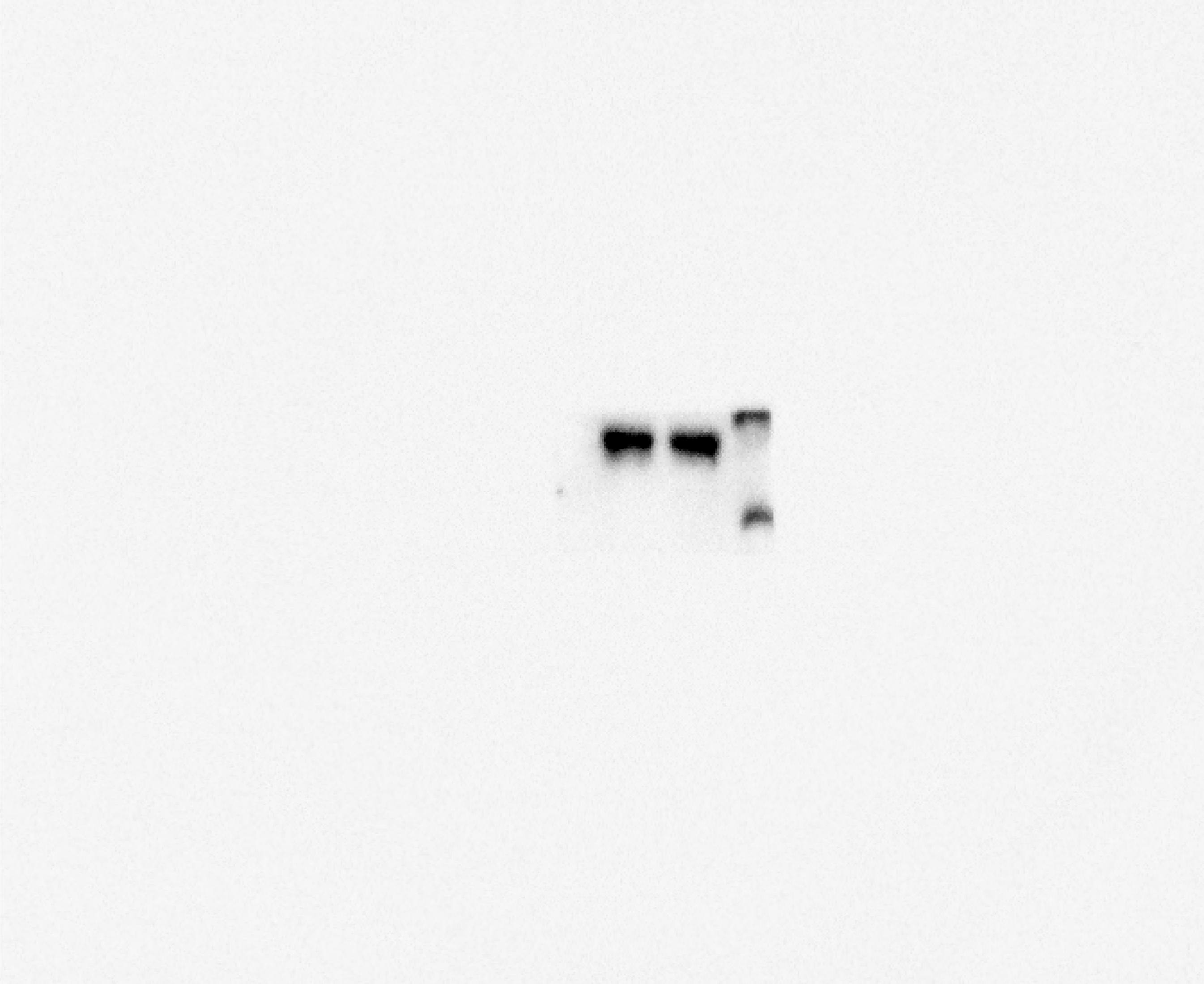

Figure 7F H3 p4

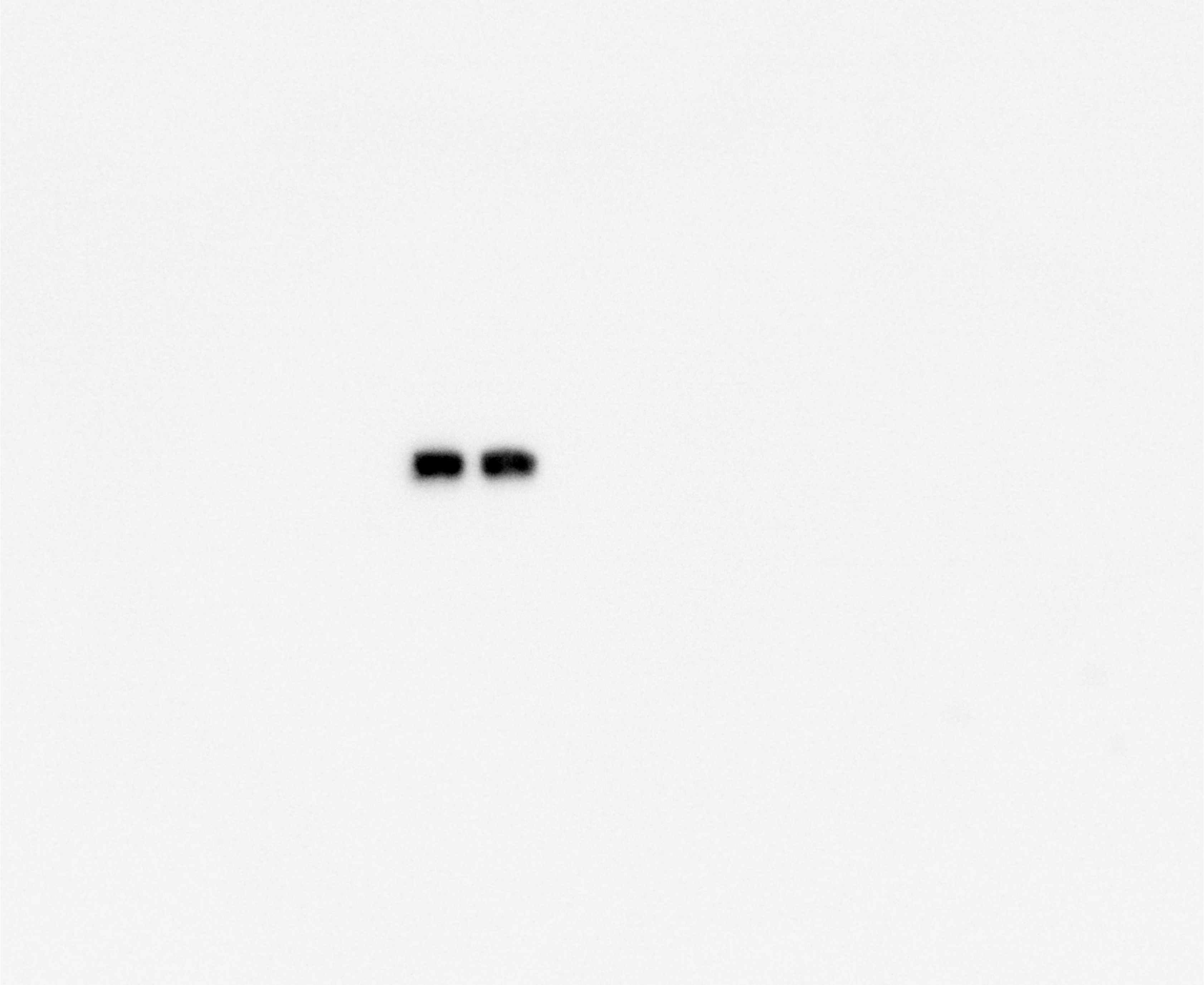

Figure 7F H3K p1

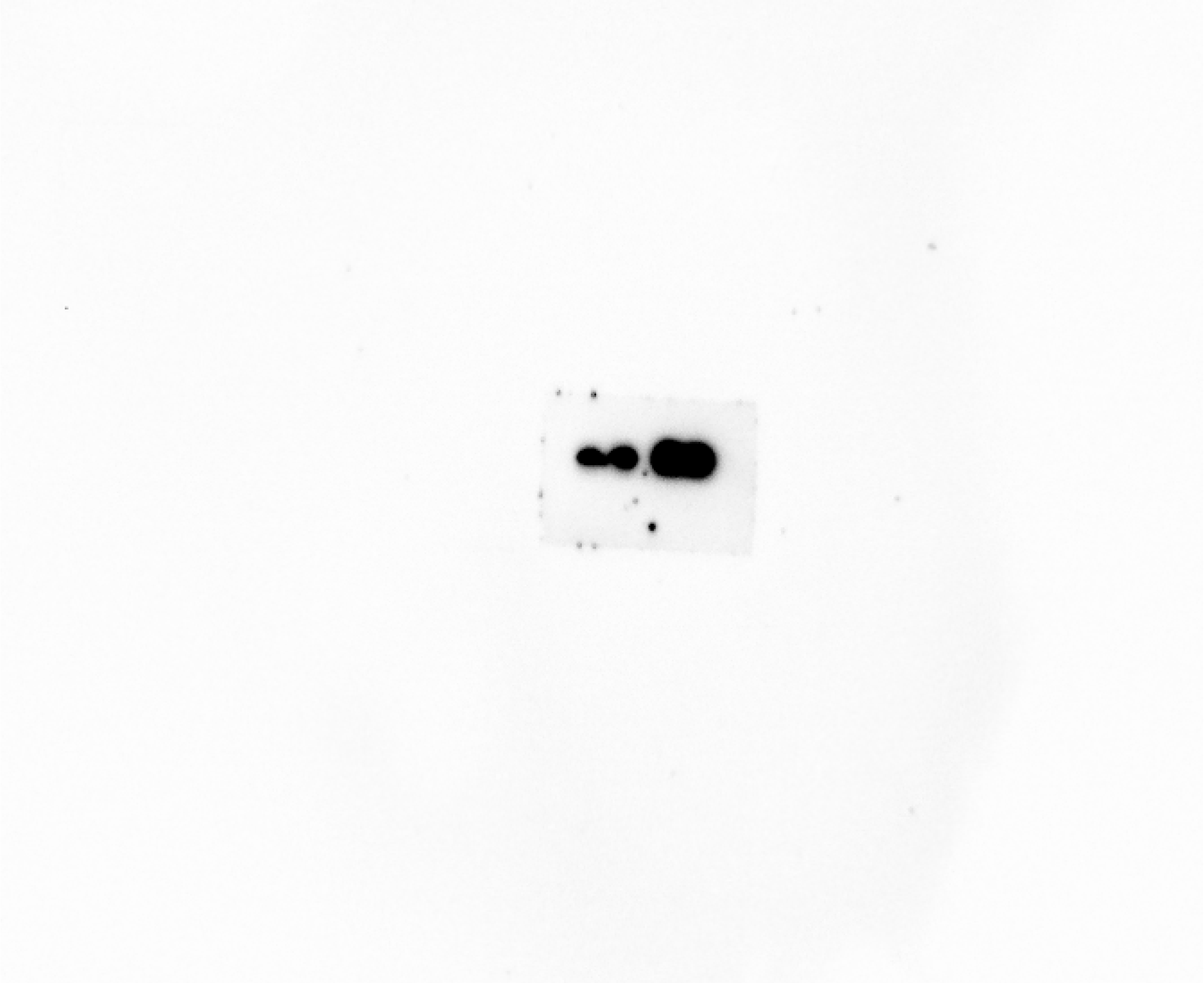

Figure 7F H3K p4

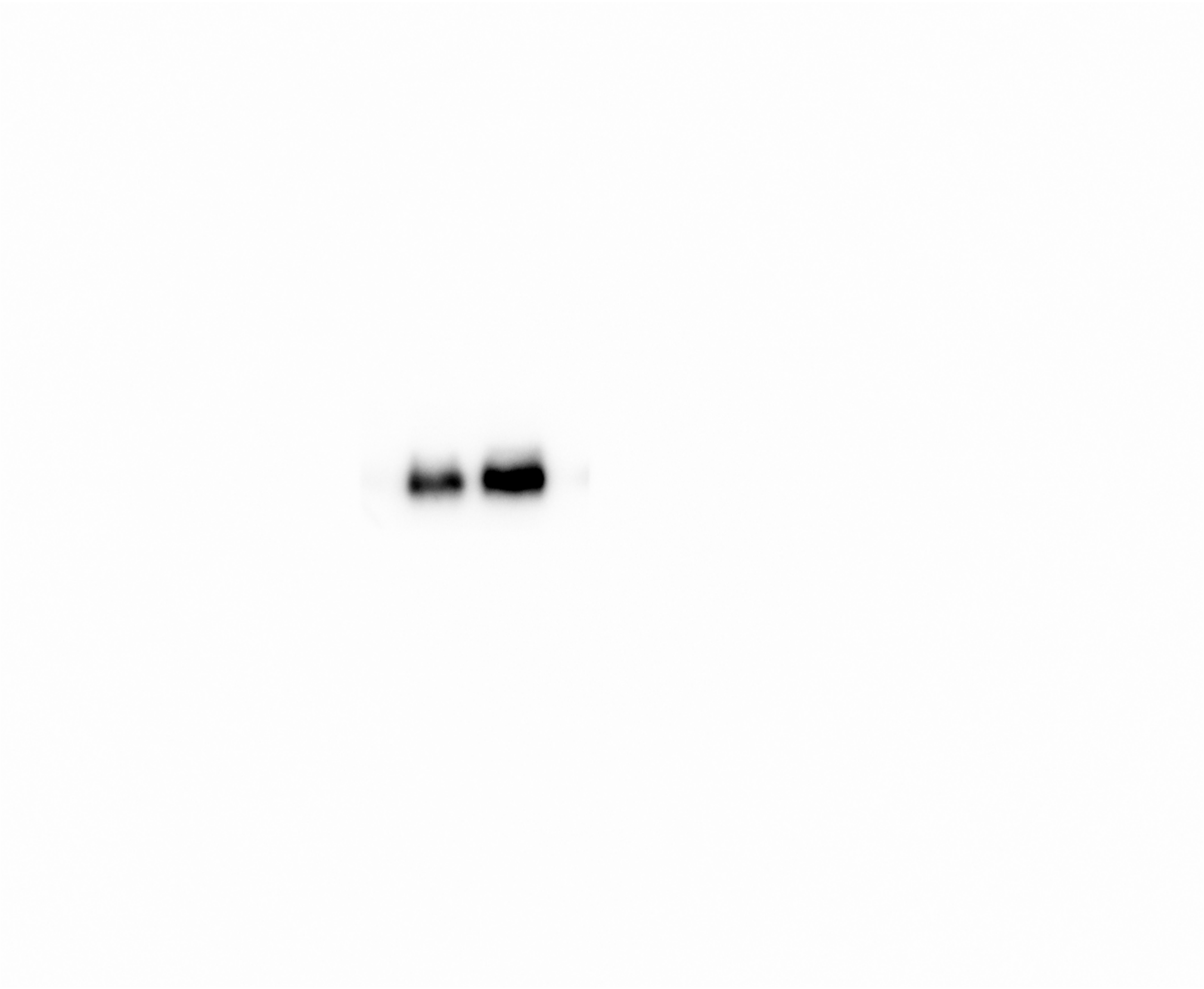

Figure 7F STAT5 p1

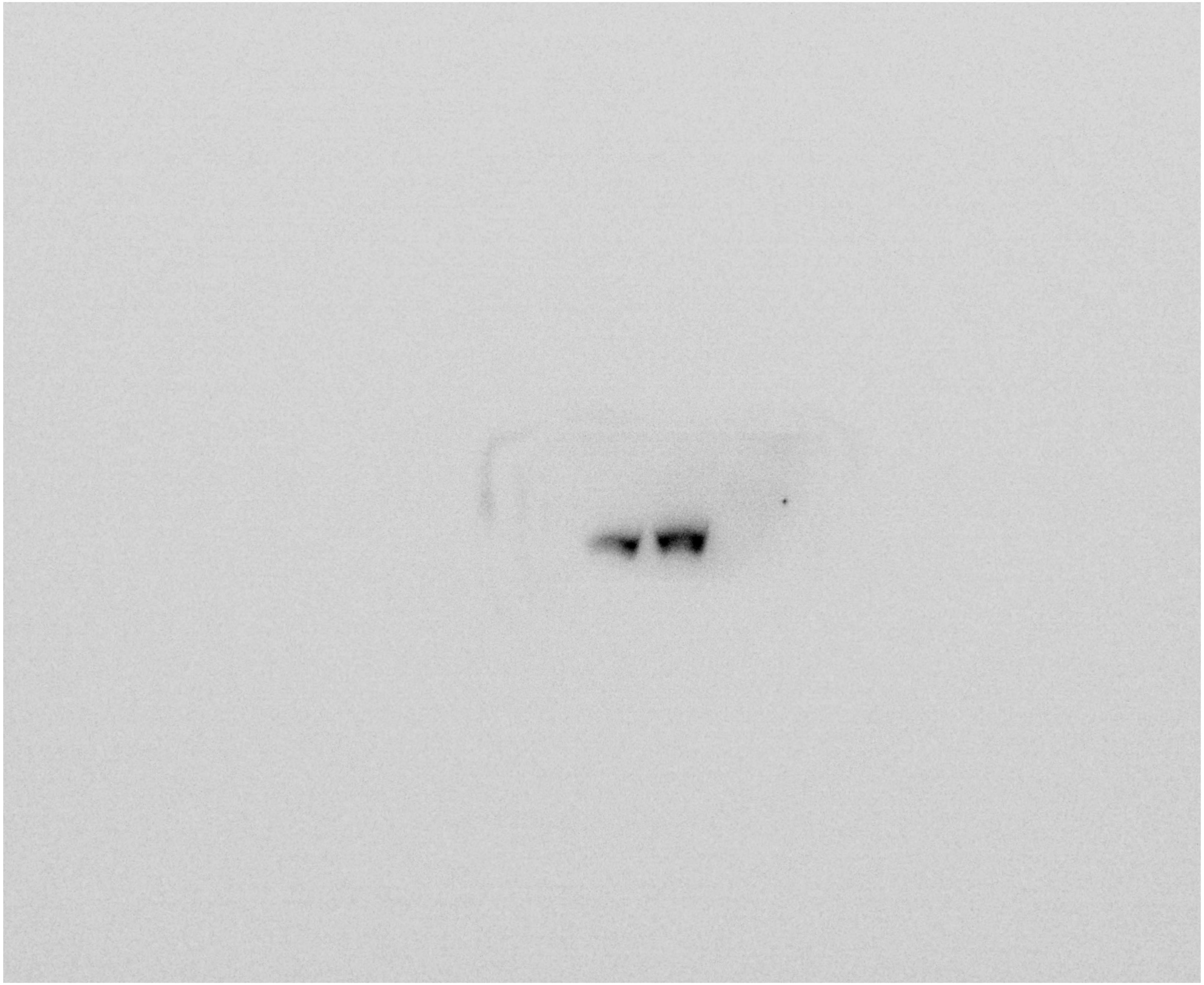

Figure S2B H3K79me3

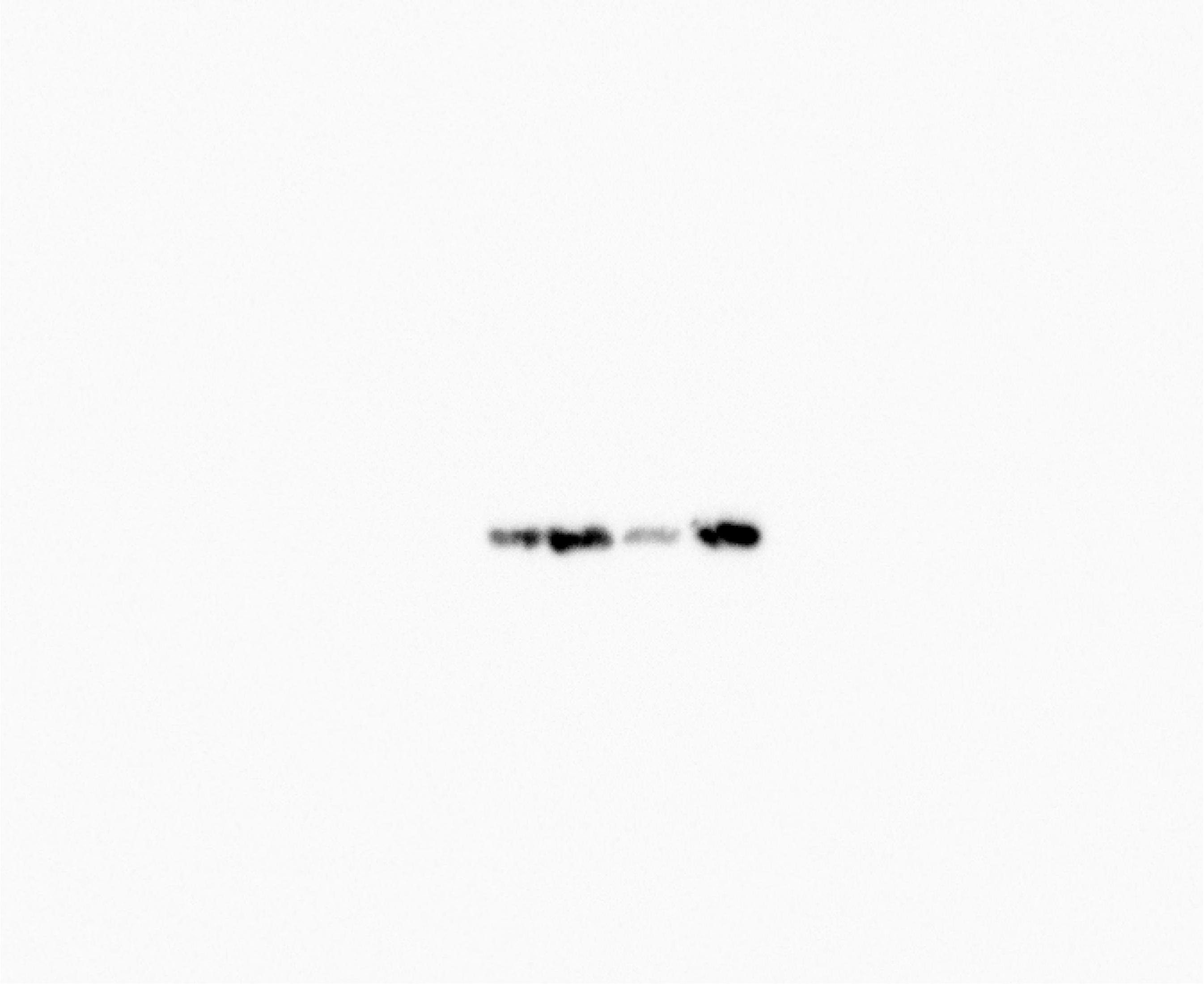

Figure S2B GAPDH

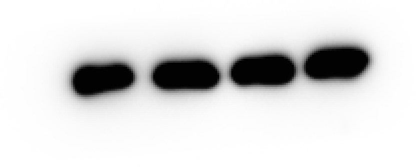

Figure S2B H3

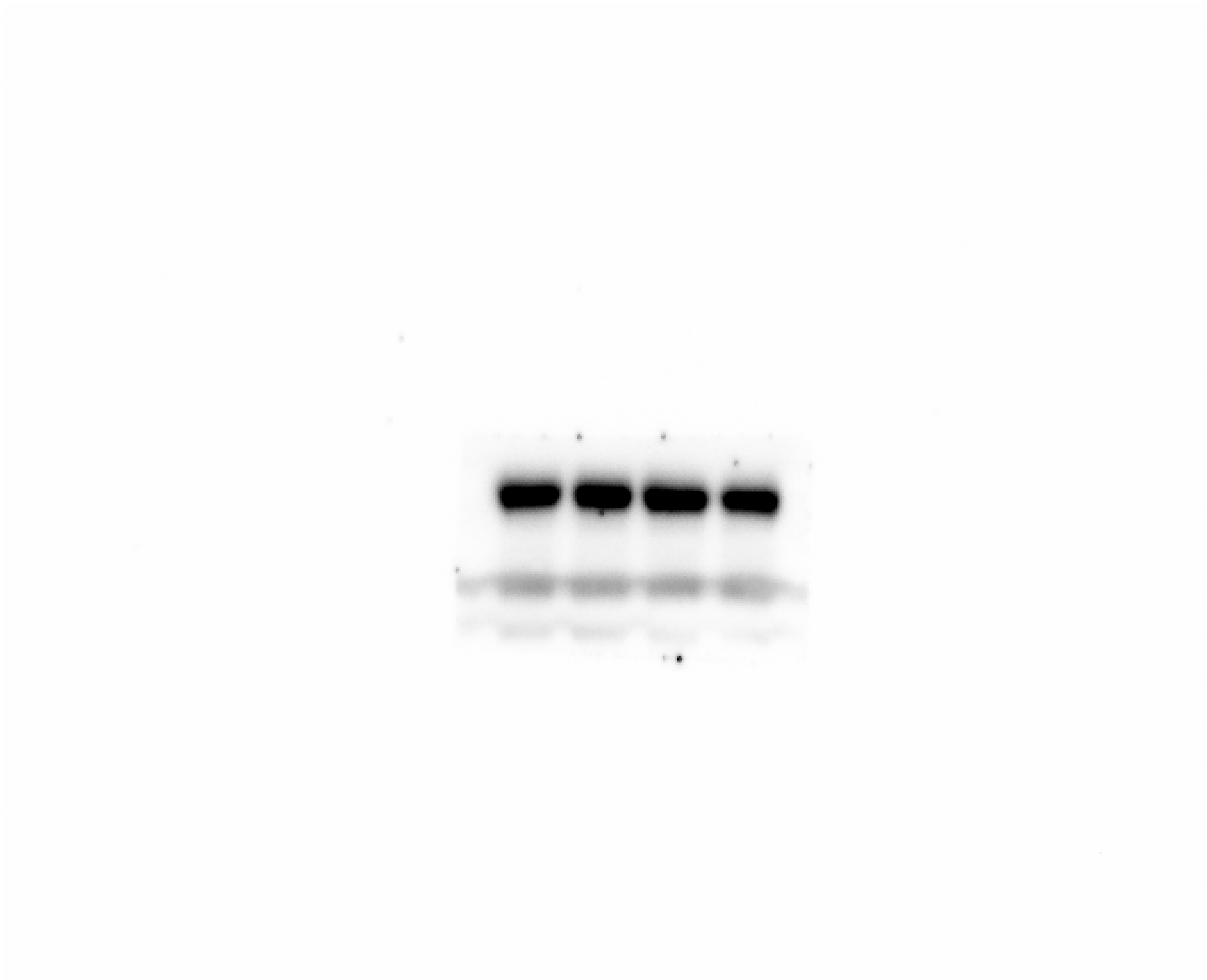

Figure S2B H3K4me3

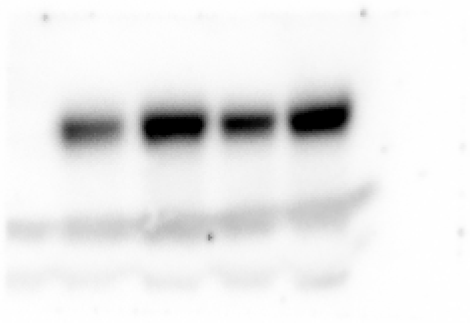

Figure S2B H3K9me3

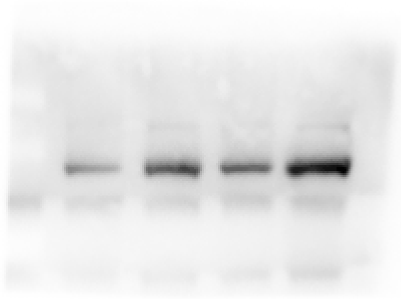

Figure S2B H3K27me3

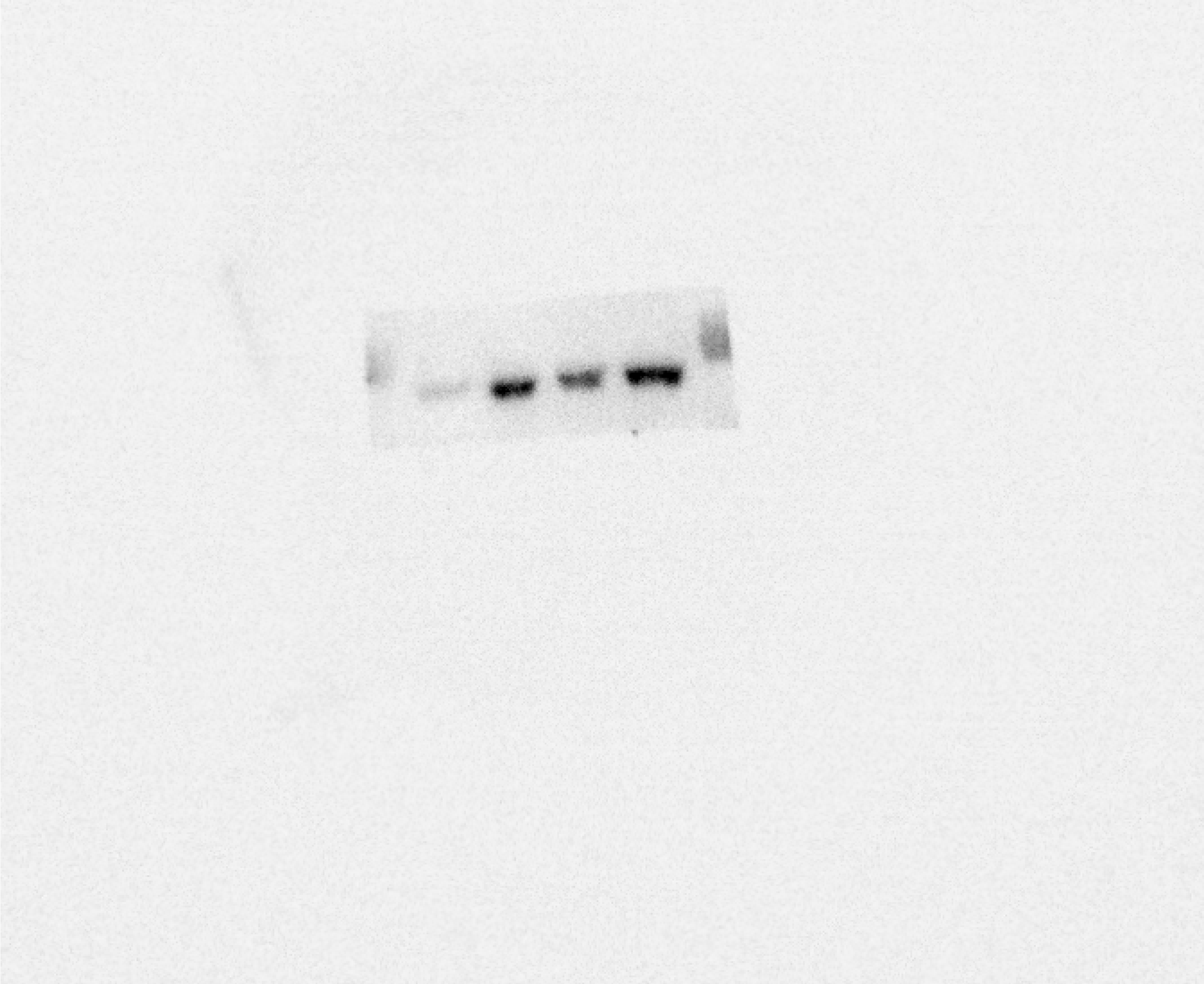

Figure S2B H3K36me2

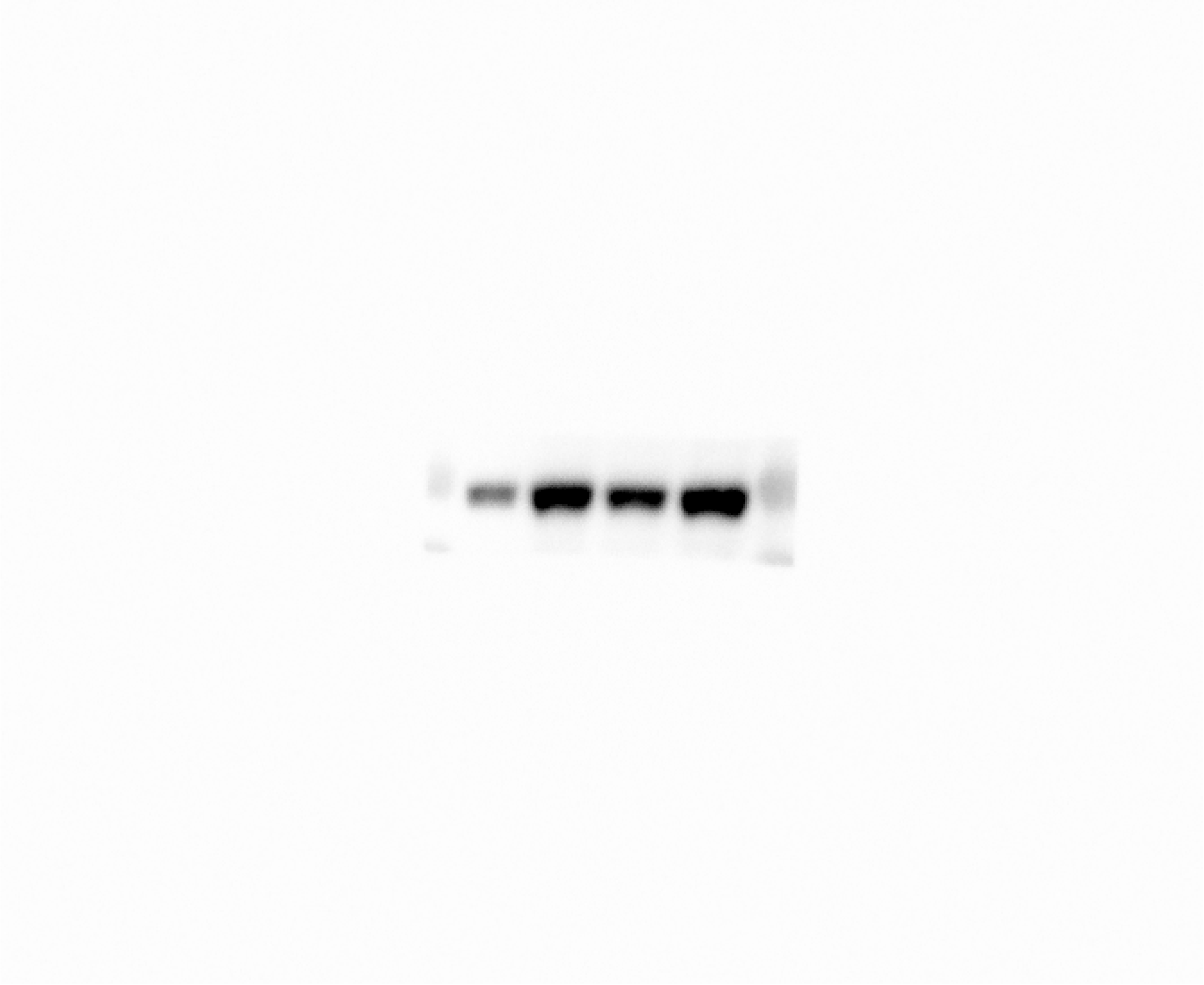

Figure S2B H3K36me3

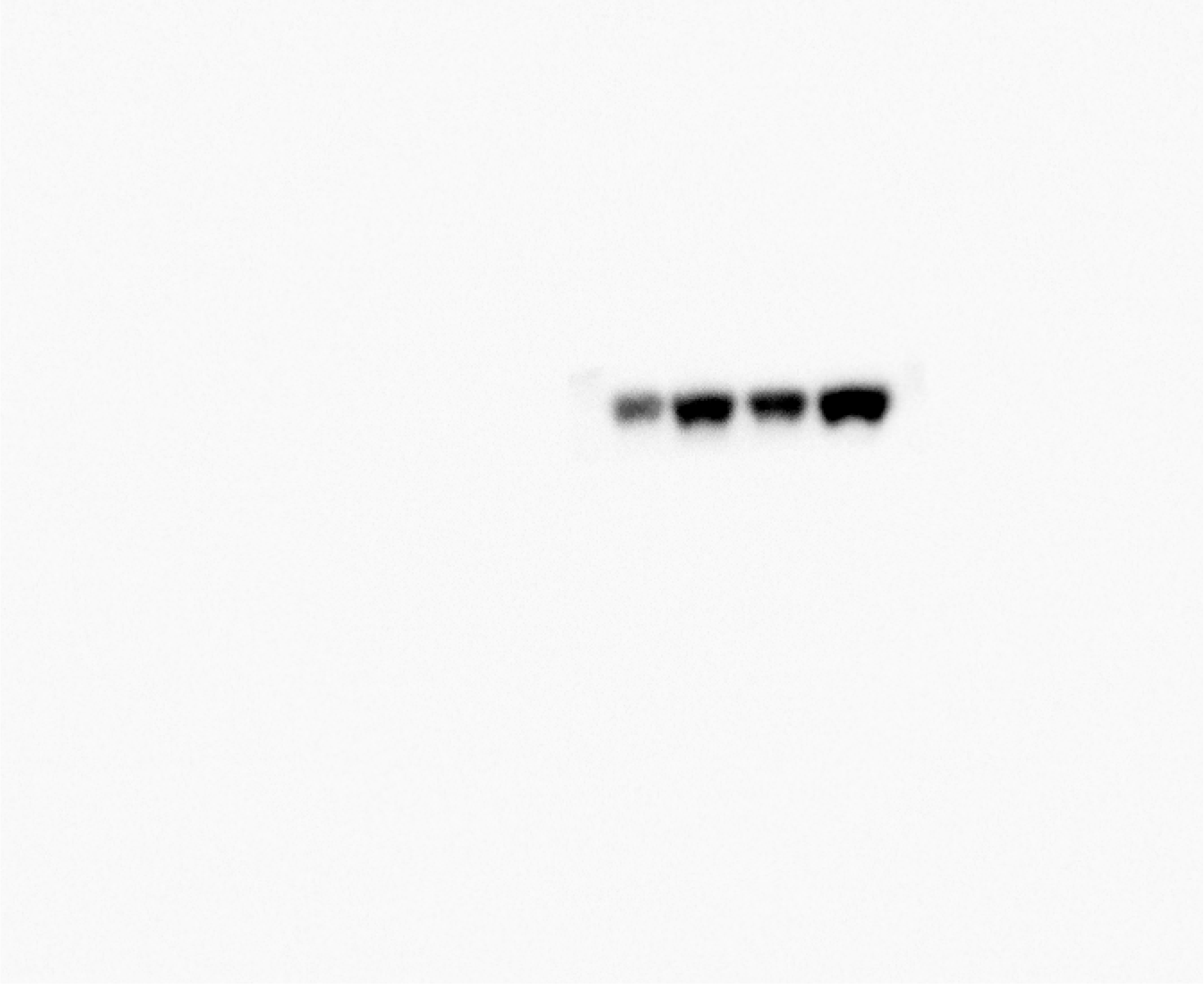

Figure S2B H3K79me2

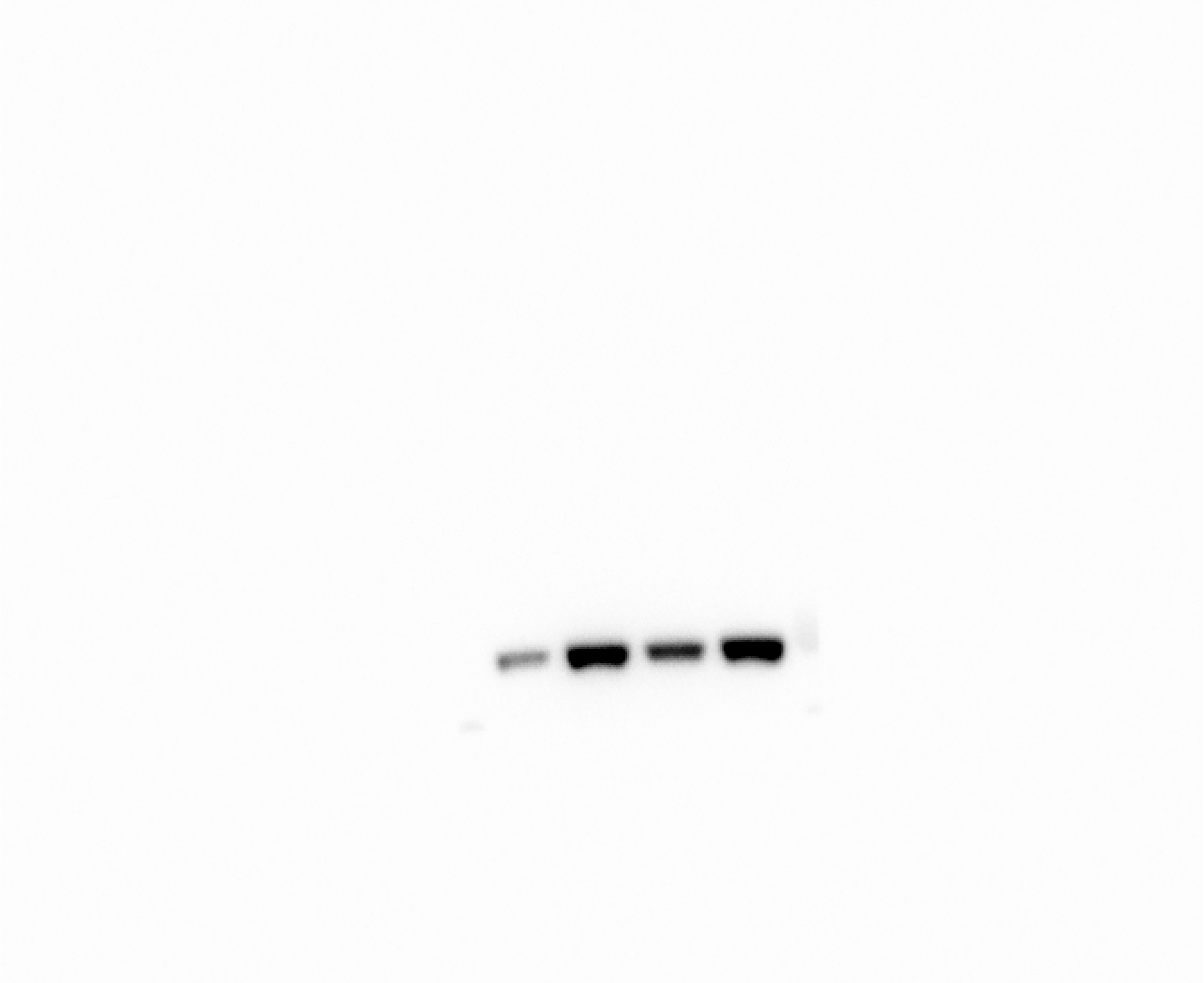

Figure S2C Total H3

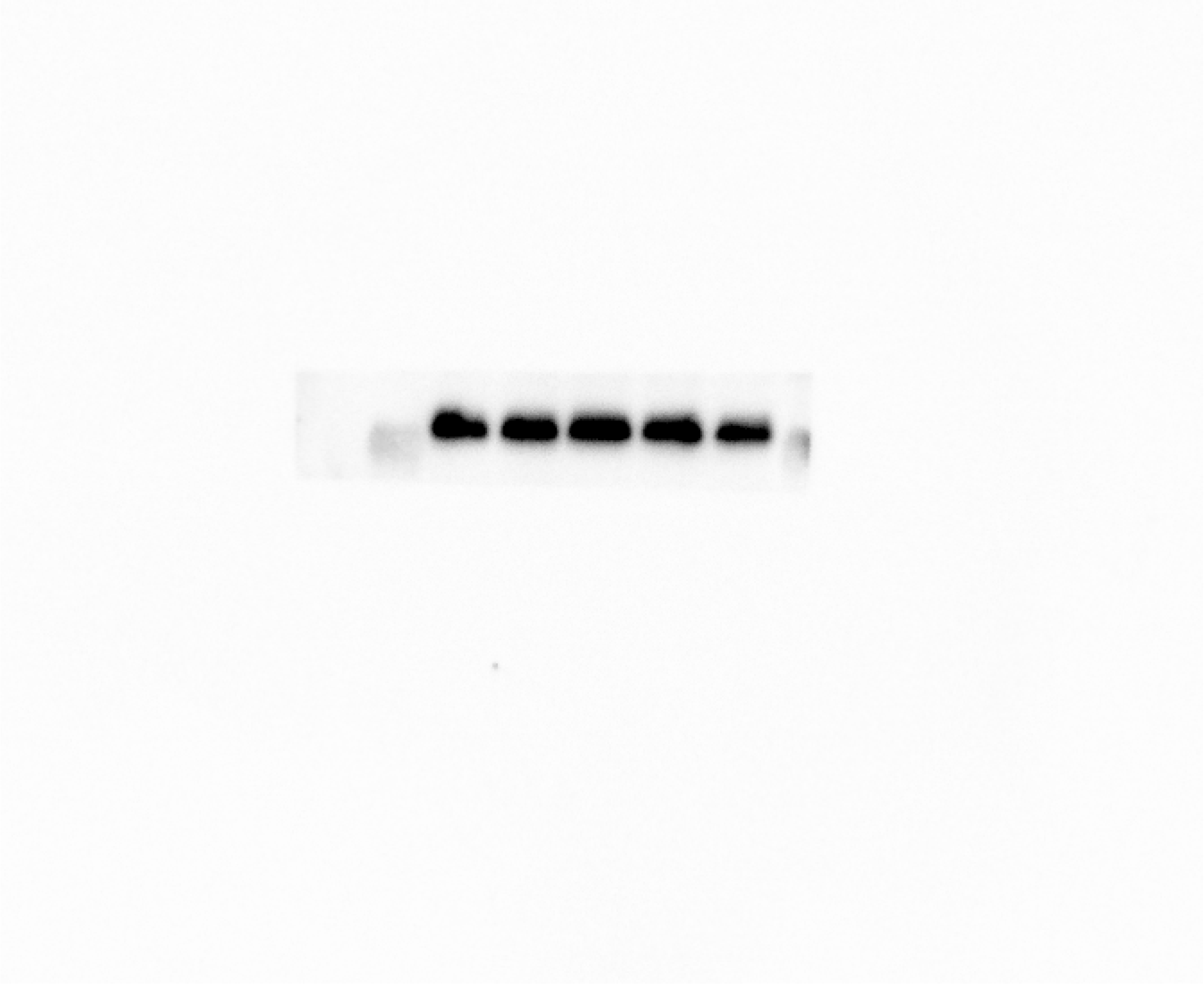

Figure S2C CD44

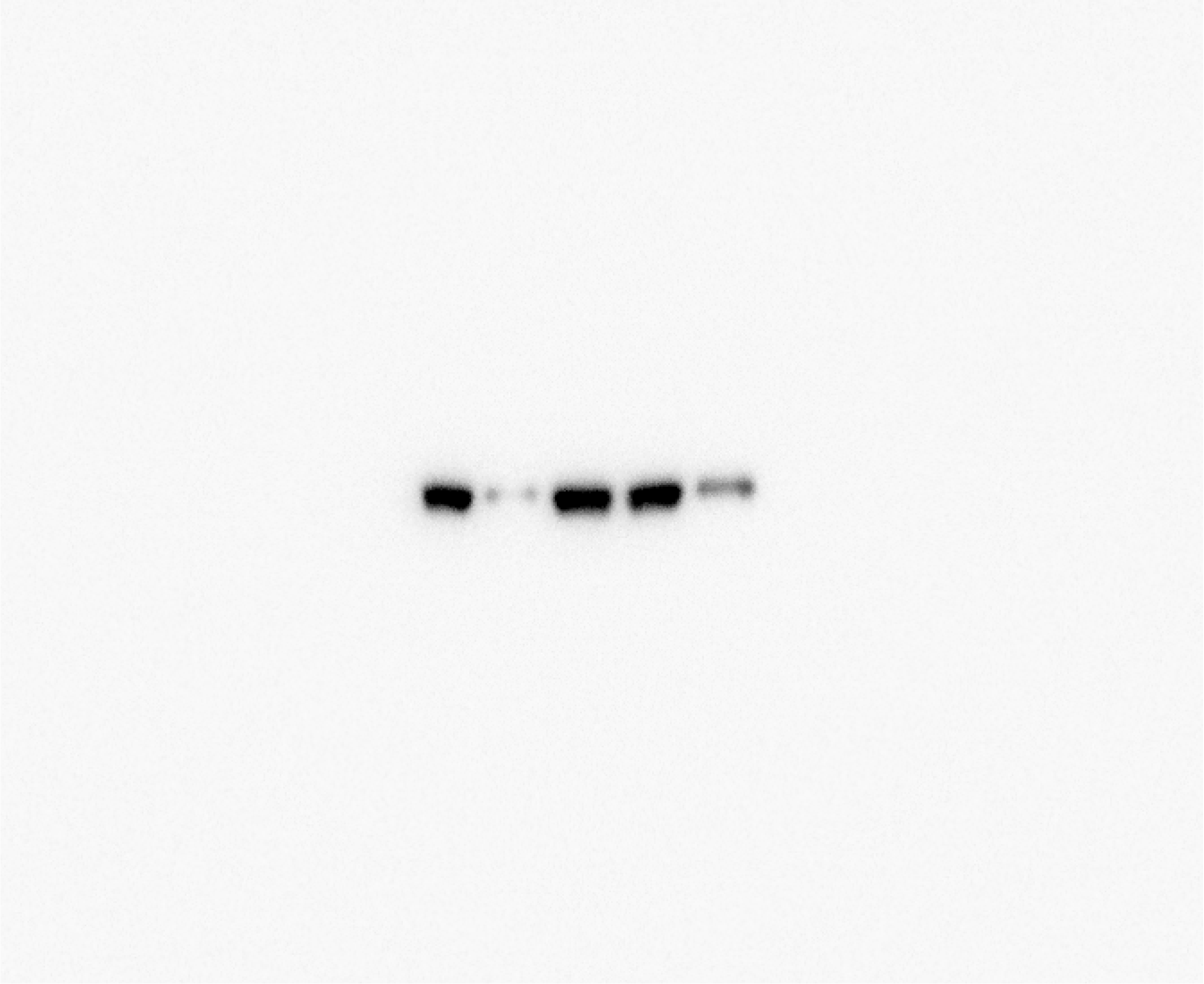

Figure S2C GAPDH

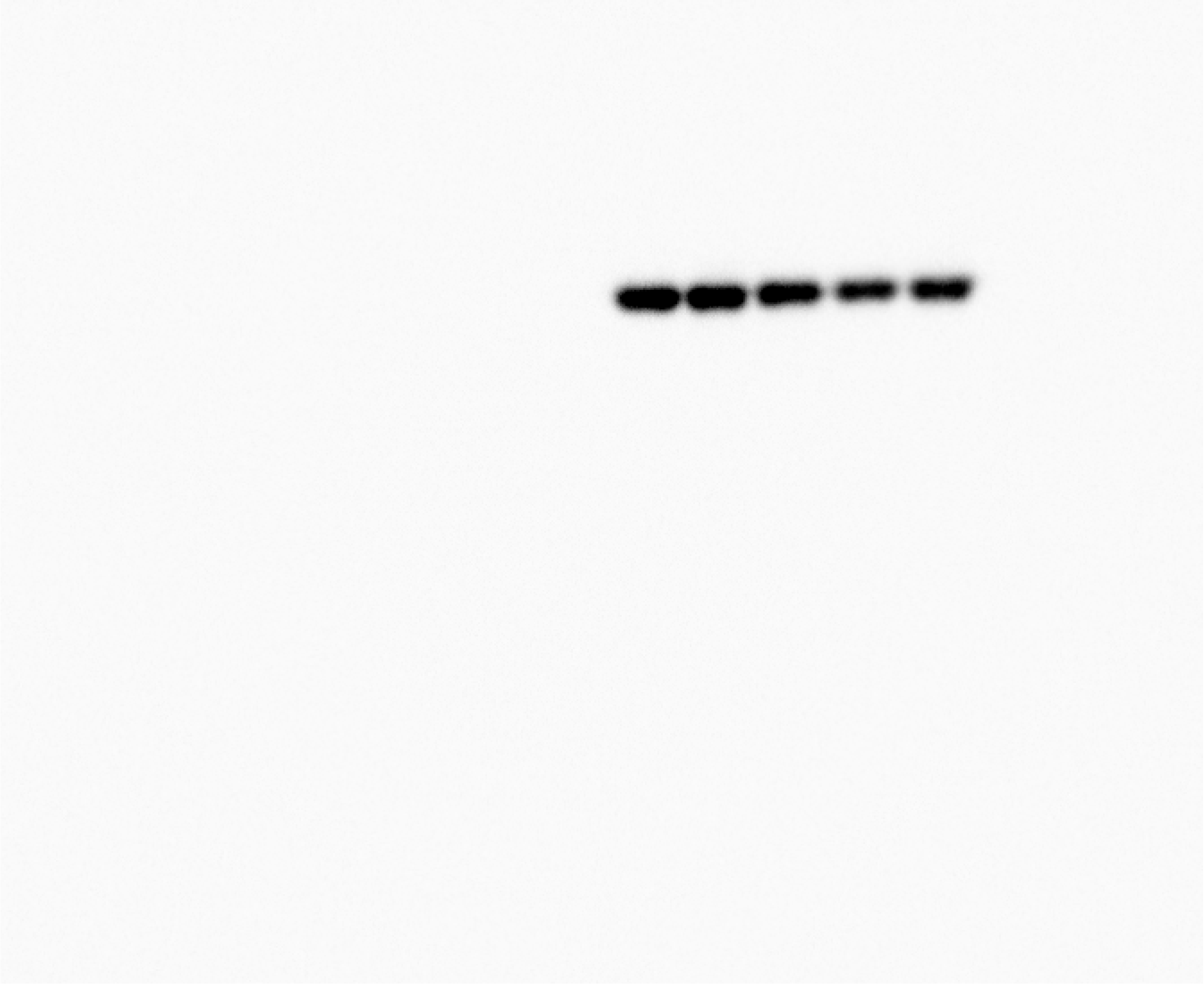

Figure S2C H3K4me3

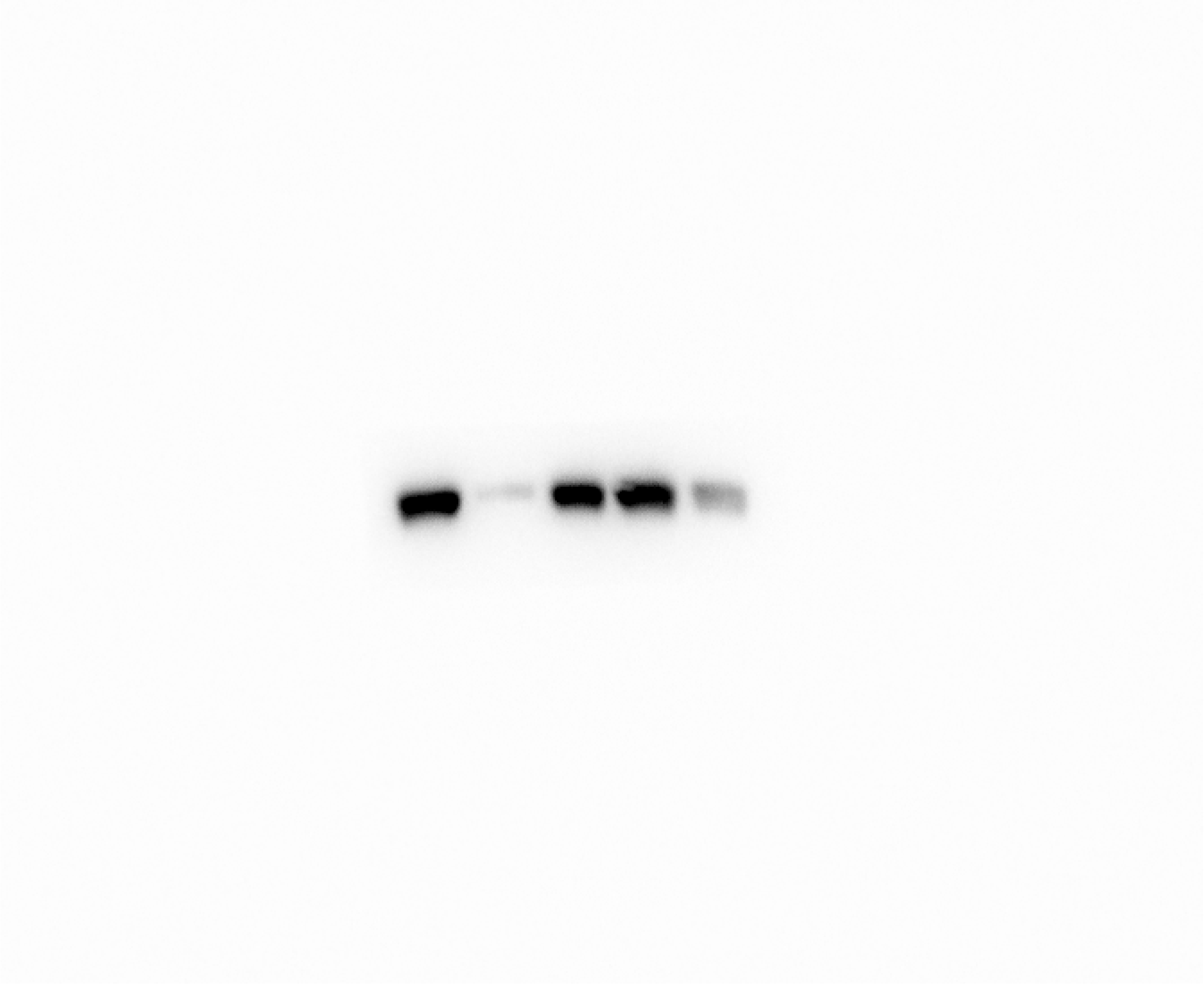

Figure S2C H3K9me3

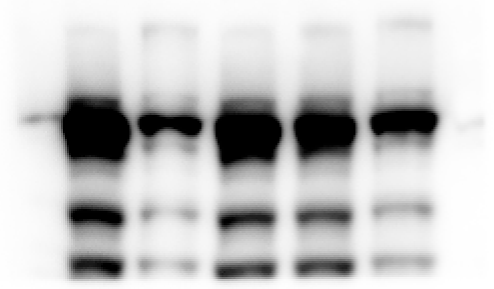

Figure S2C H3K27me3

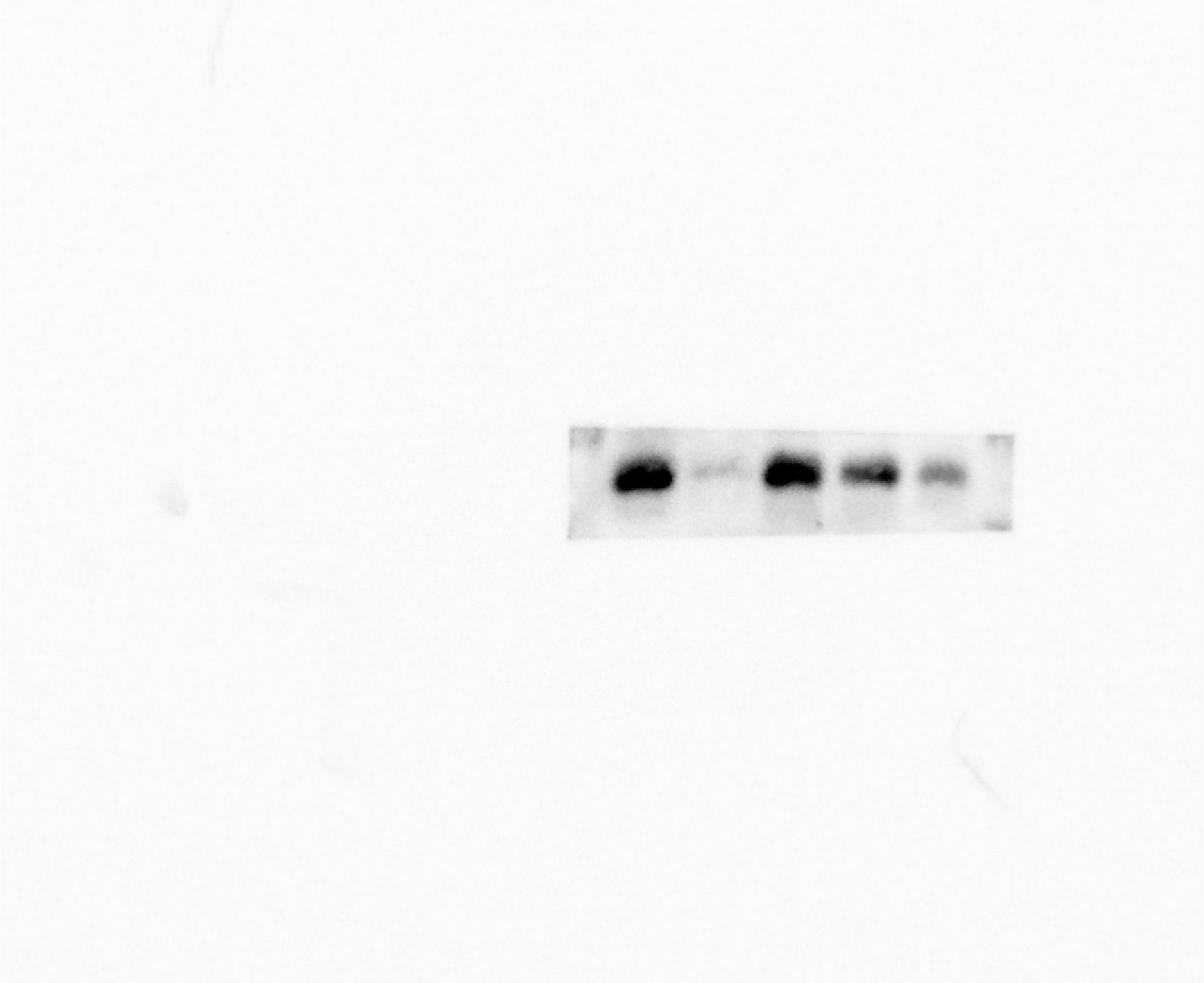

Figure S2C H3K36me3

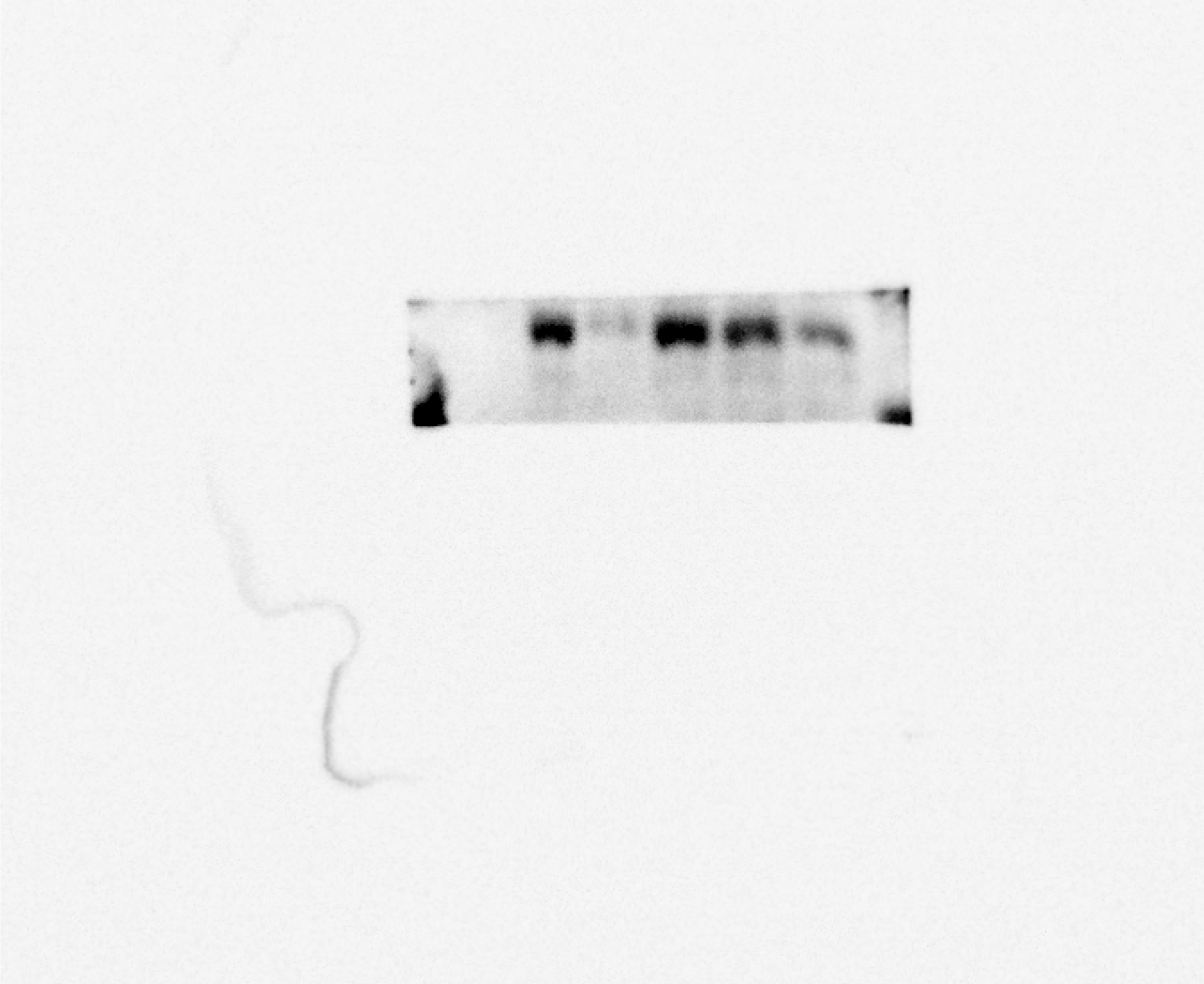

Figure S2C Nanog

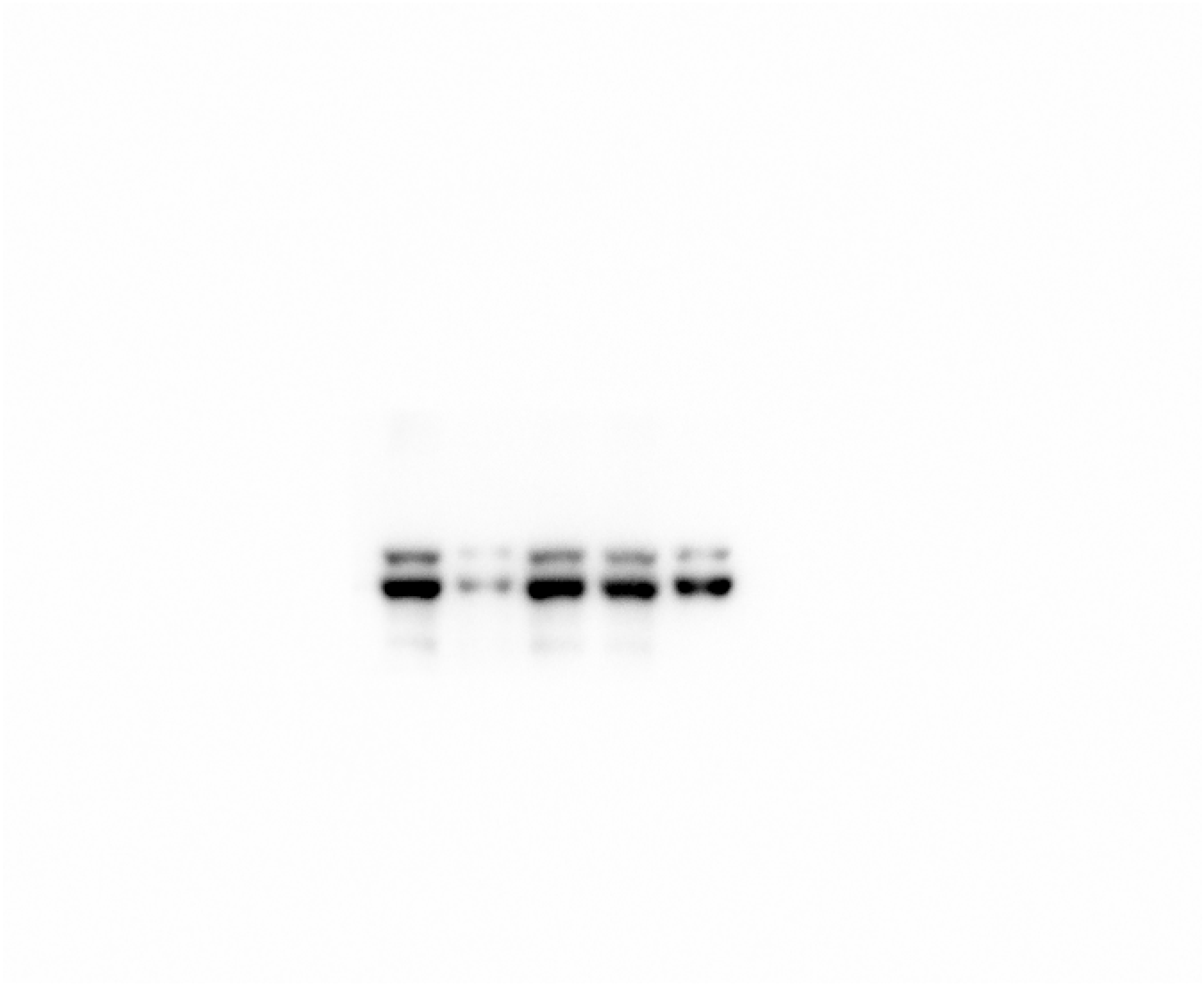

Figure S2D Nanog

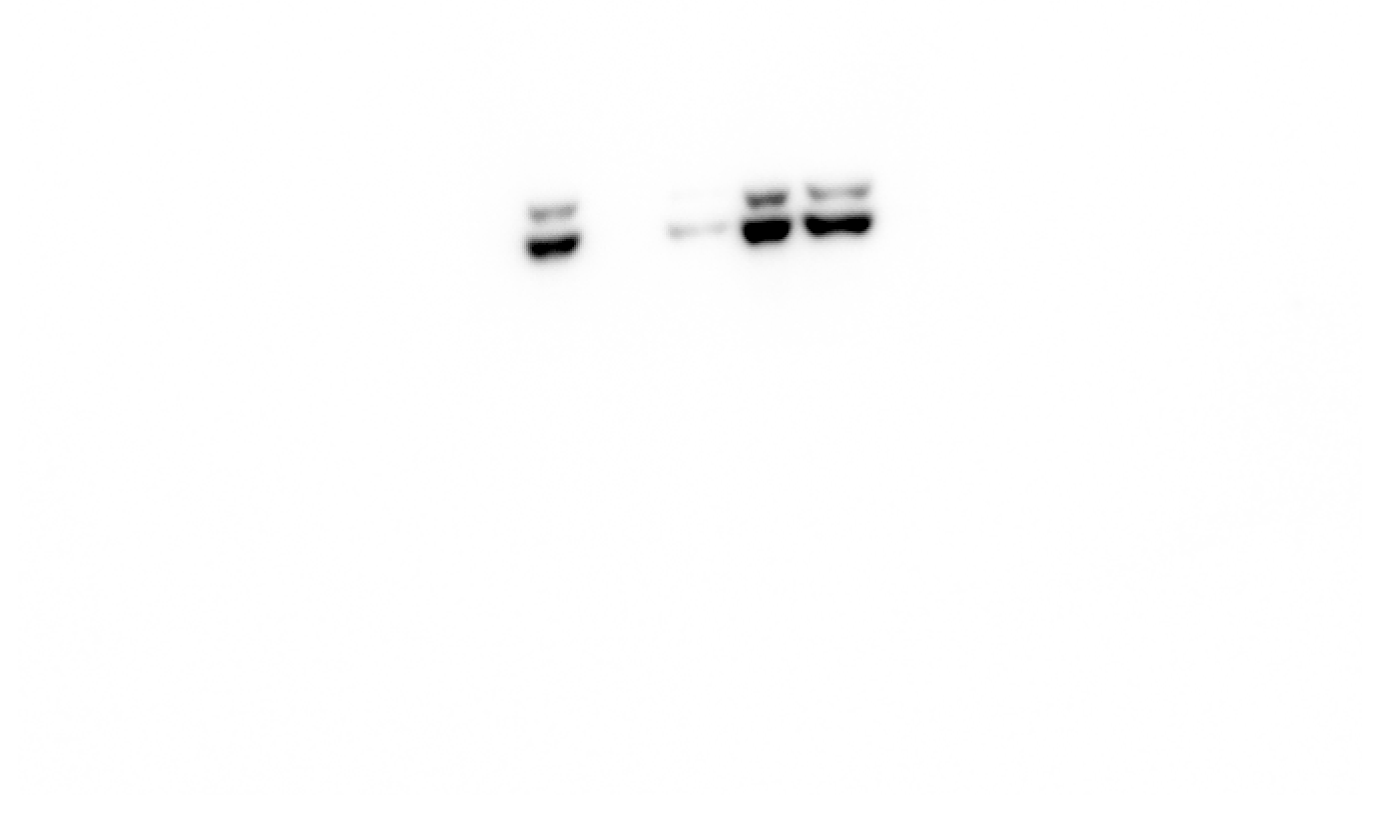

Figure S2D CD44

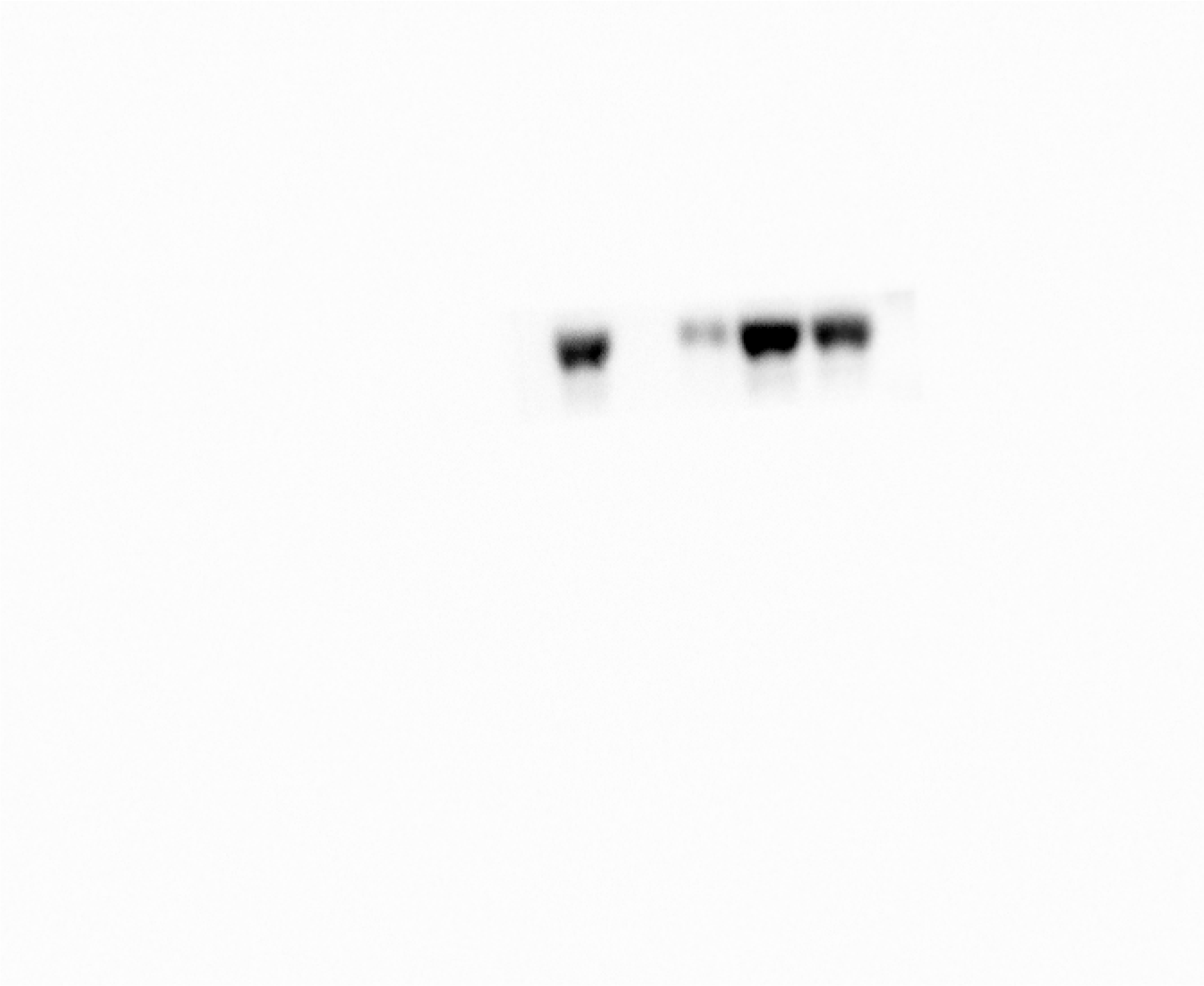

Figure S2D GAPDH

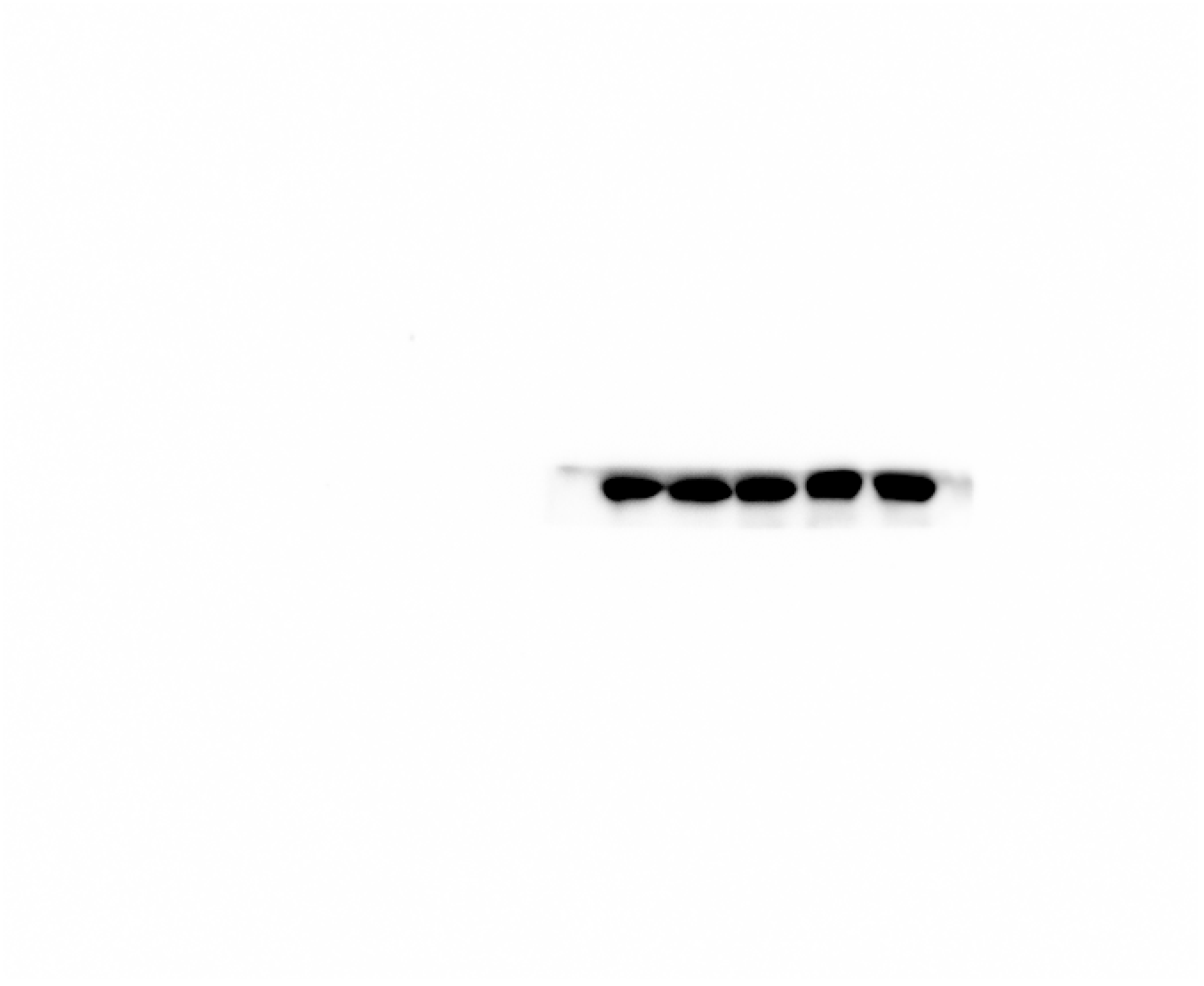

Figure S2D H3

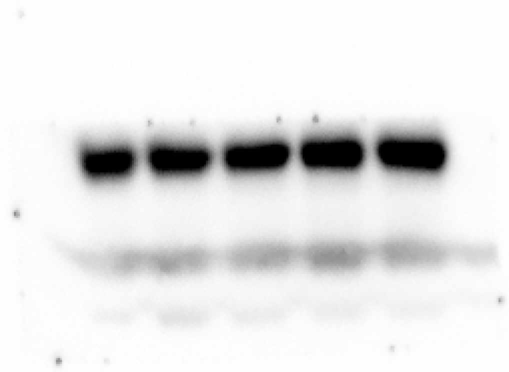

Figure S2D H3K4me3

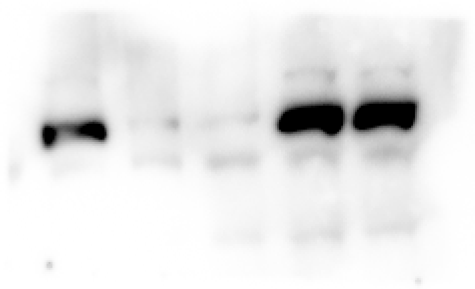

Figure S2D H3K9me3

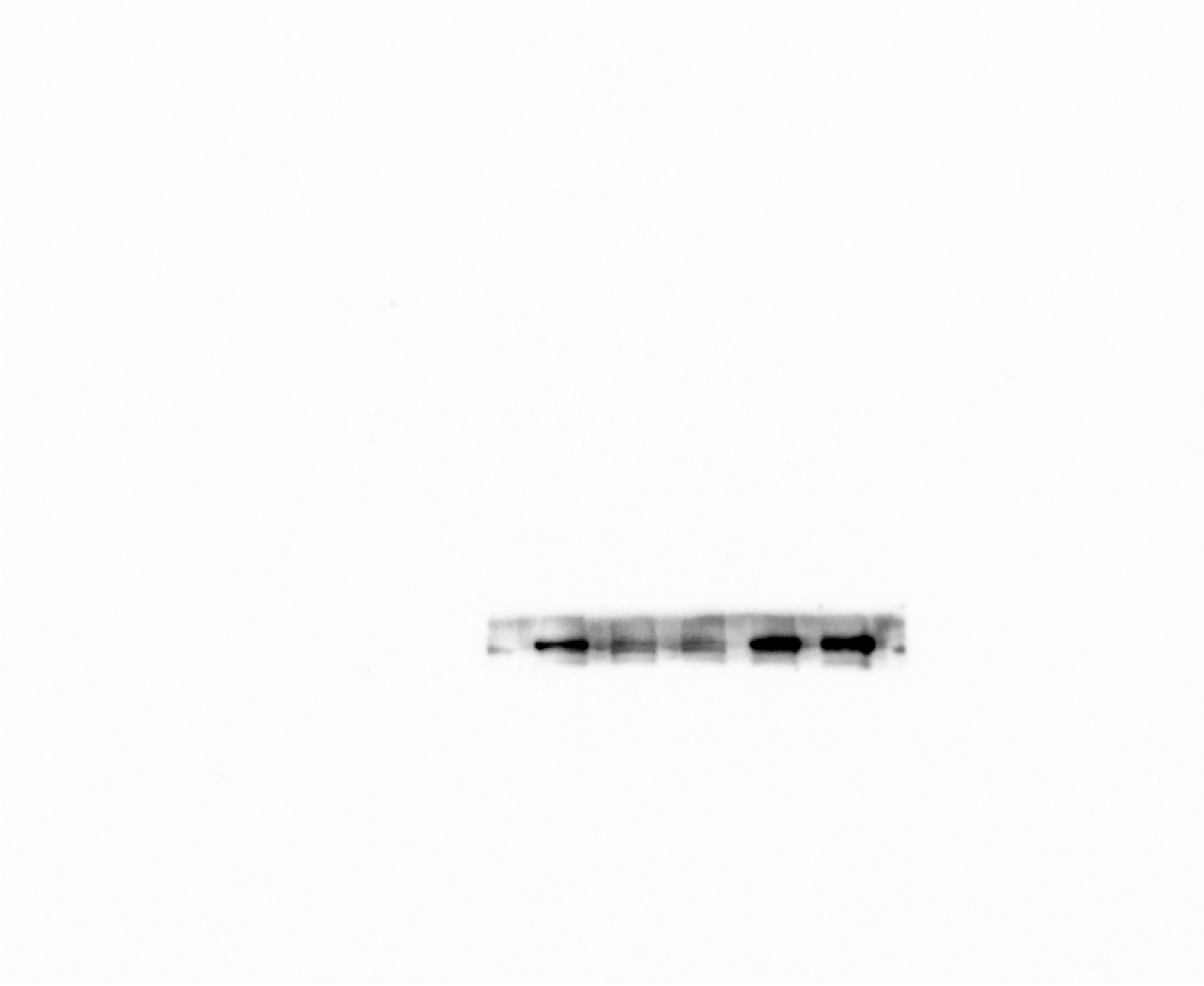

Figure S2D H3K27me3

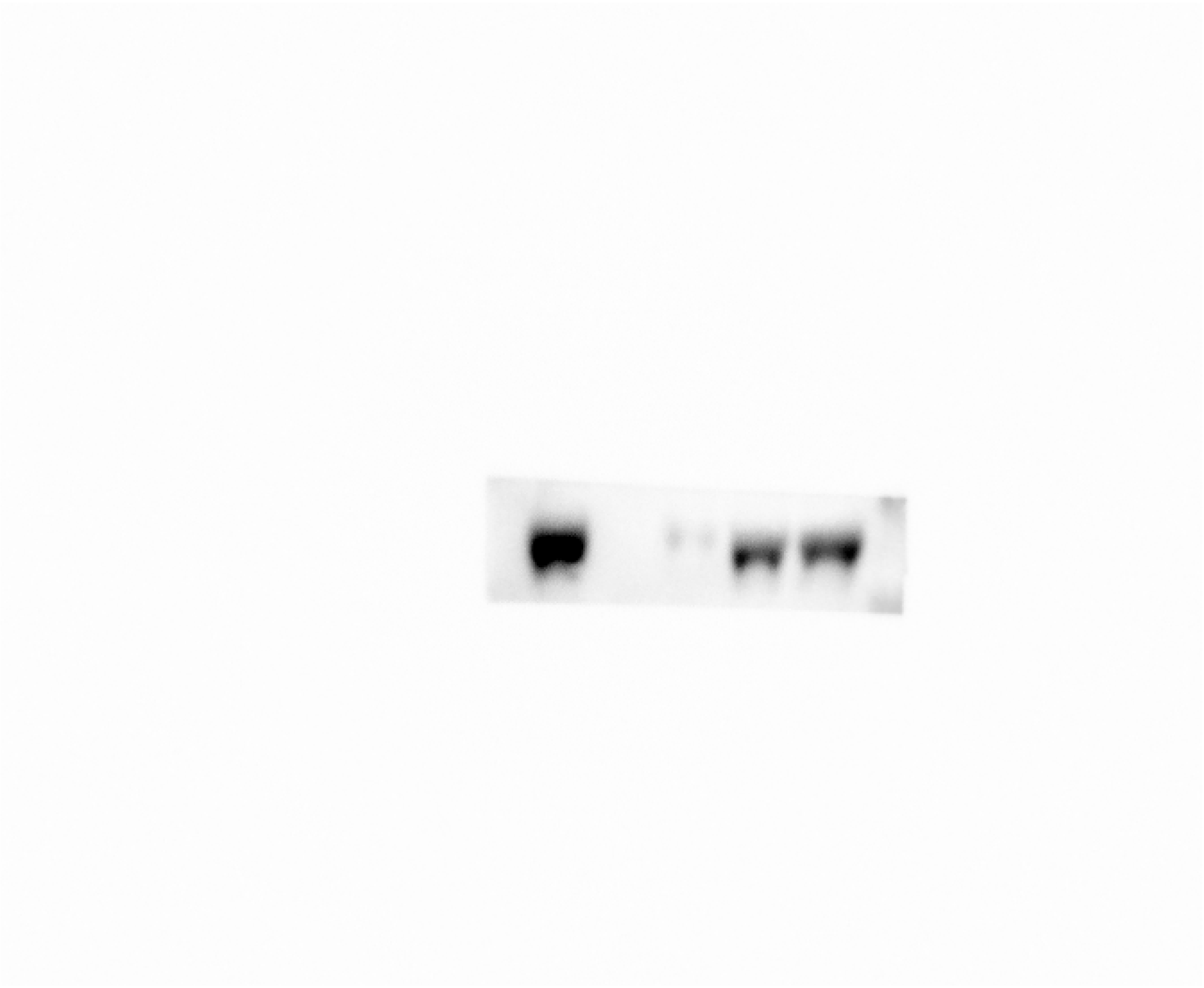

Figure S2D H3K36me3

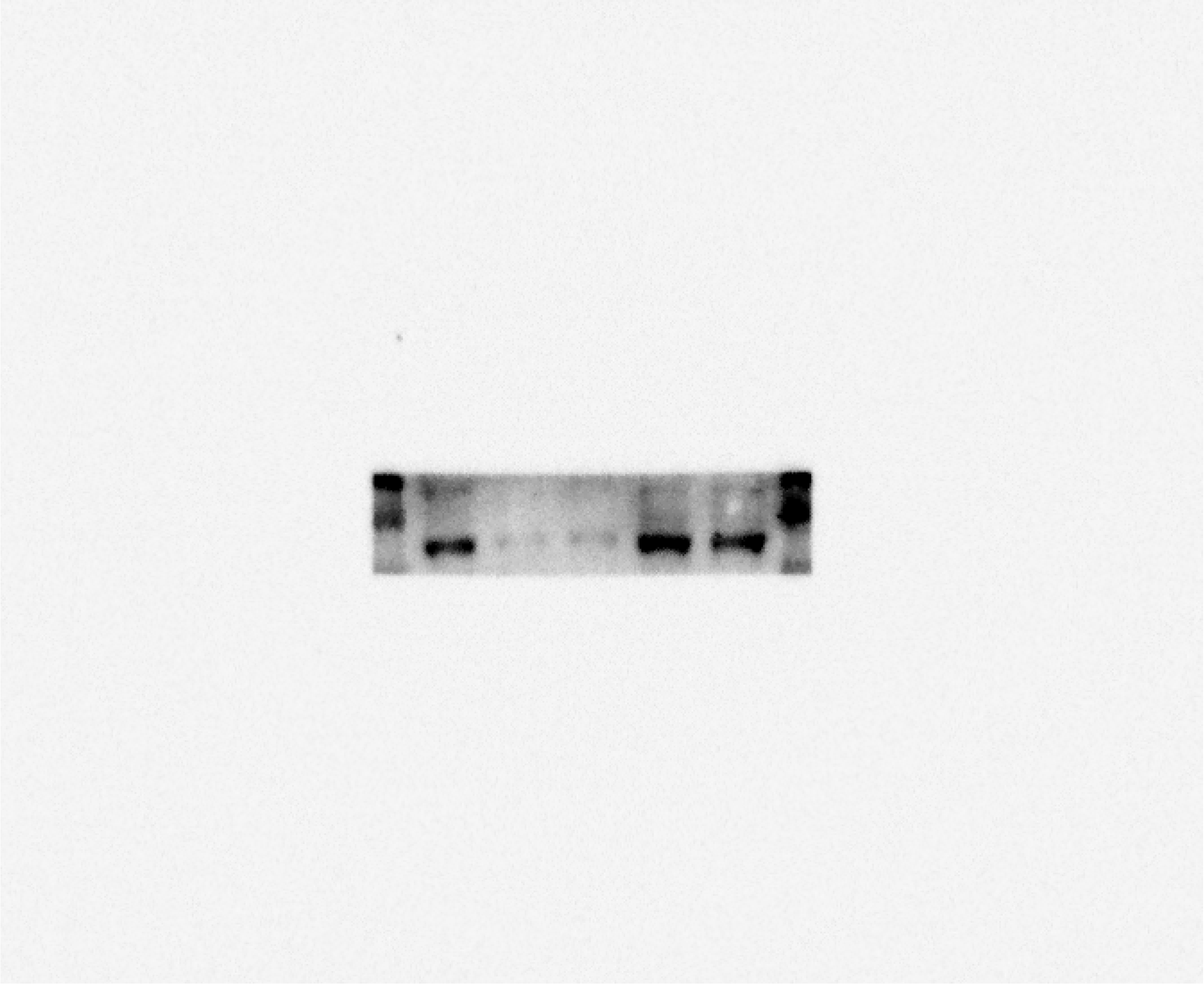

Figure S5C IP TRIM25 IgT

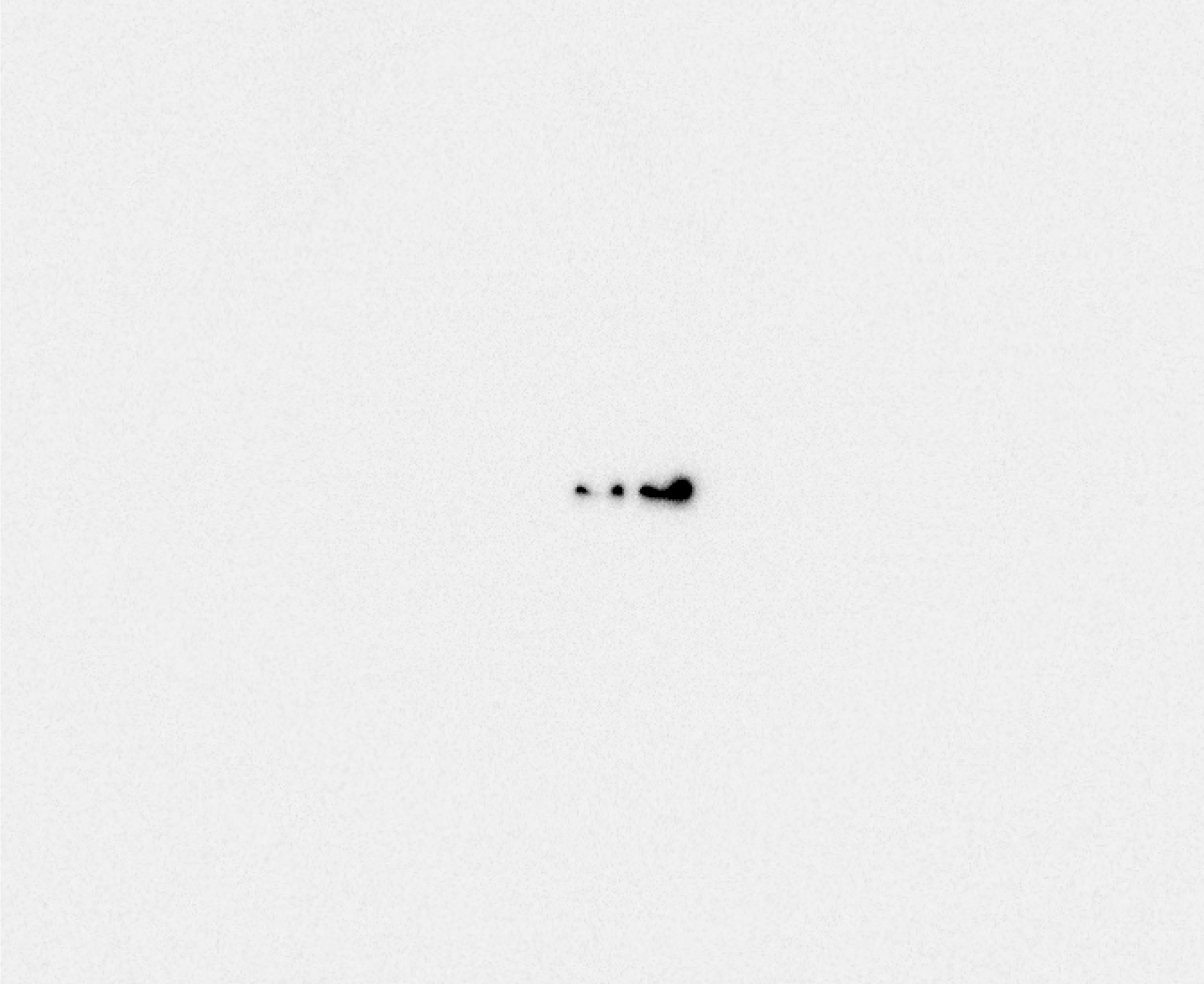

Figure S5C Input MAT2A IgM

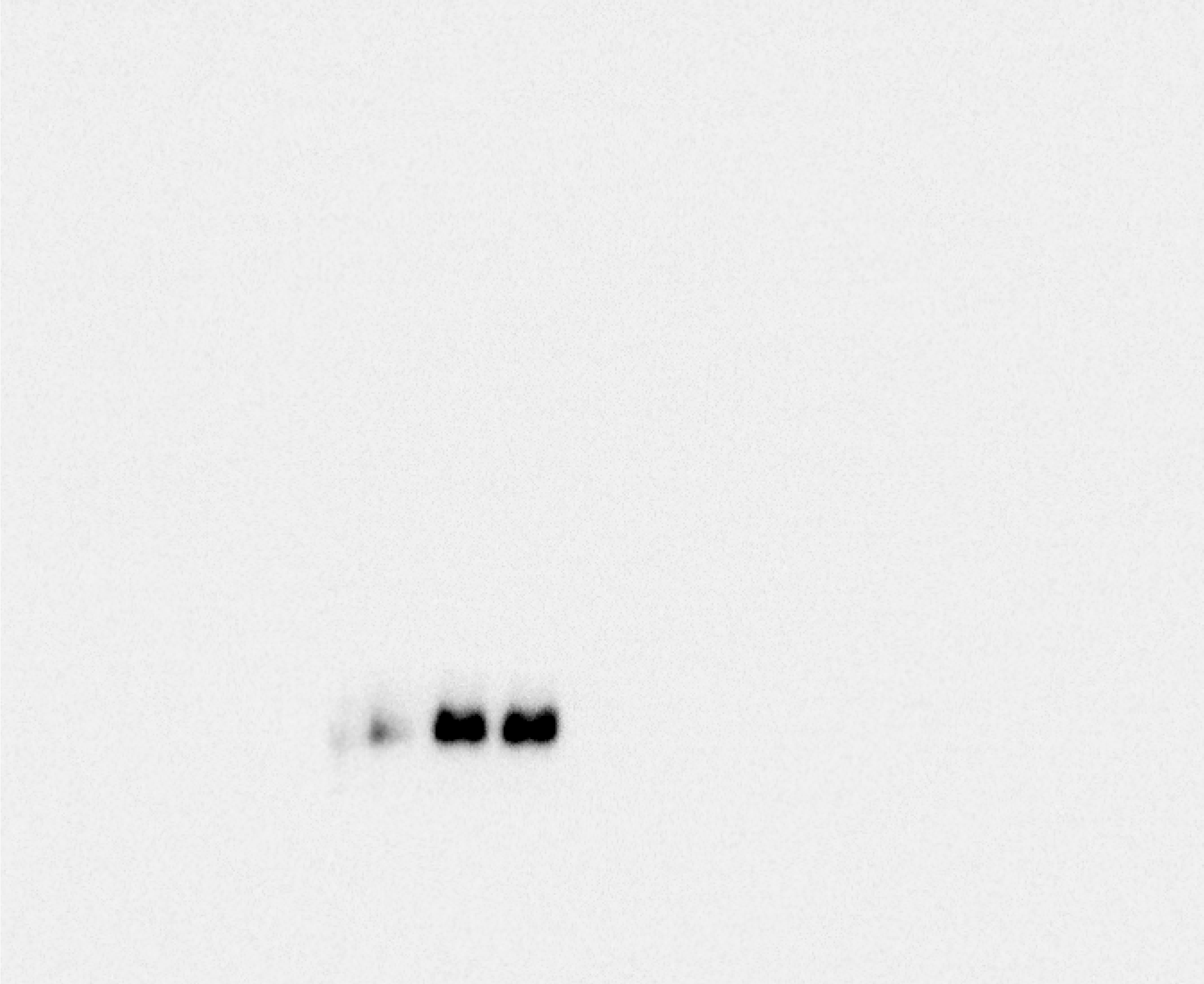

Figure S5C Input MAT2A IgT

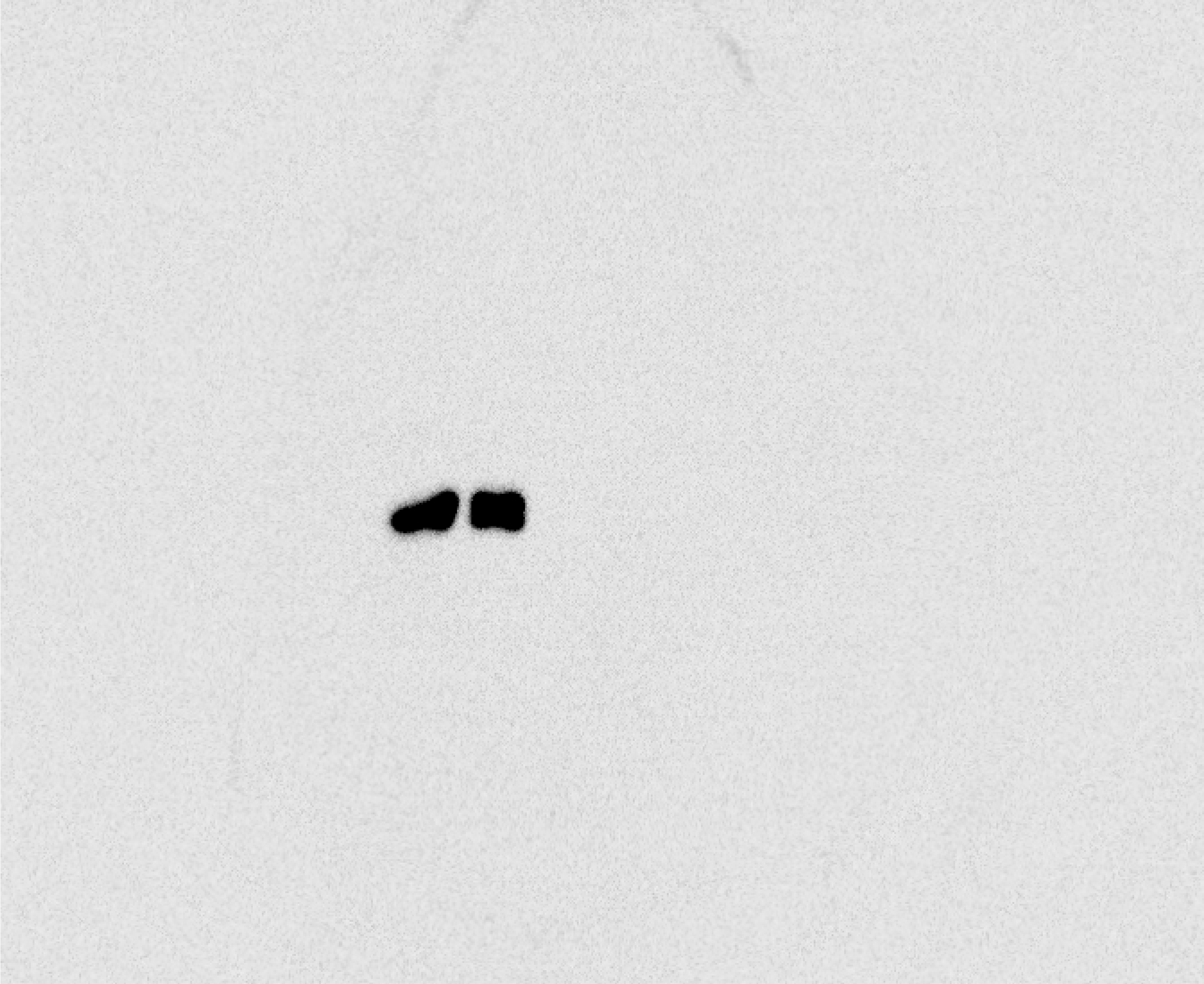

Figure S5C Input TRIM25 IgM

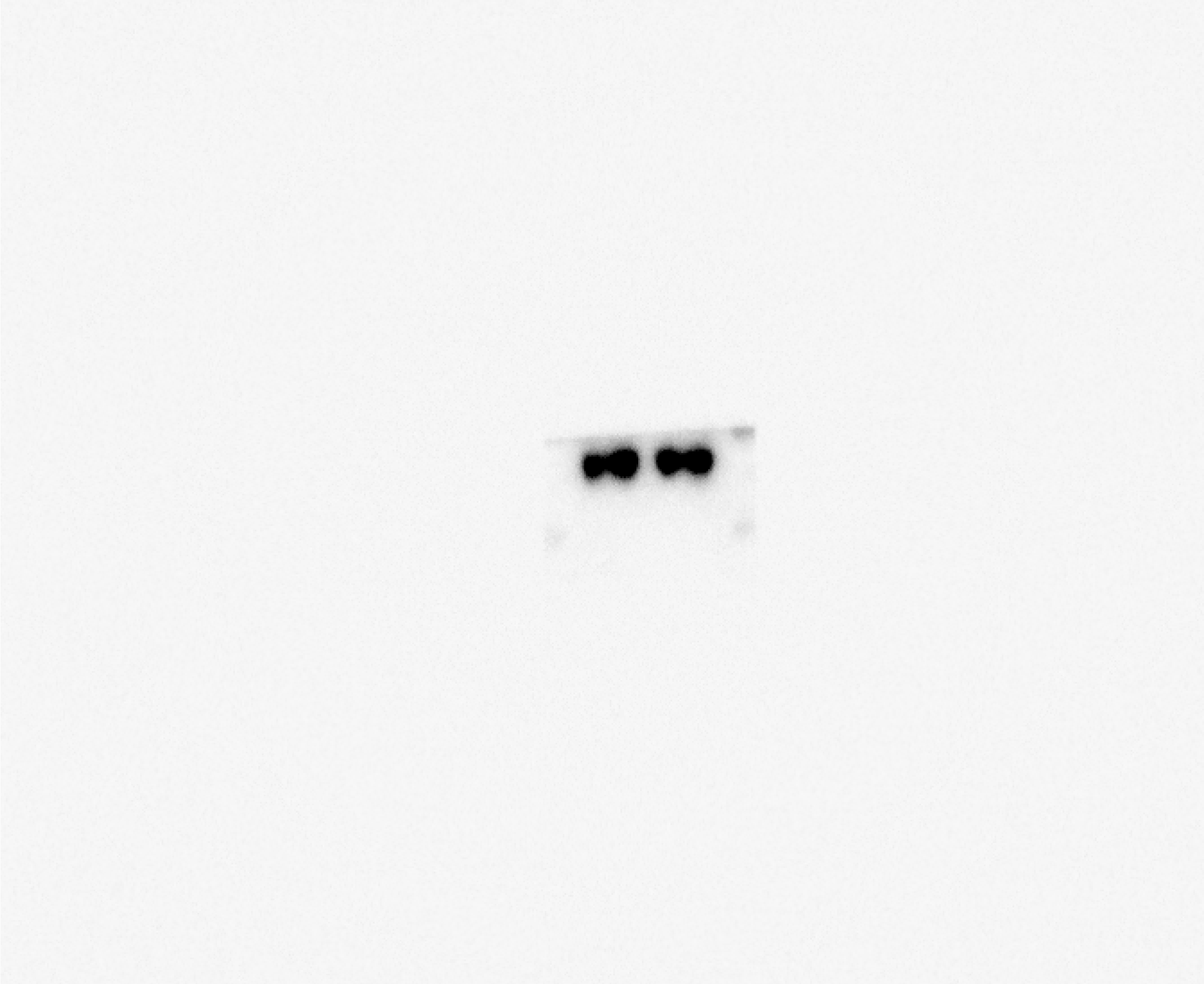

Figure S5C Input TRIM25 IgT

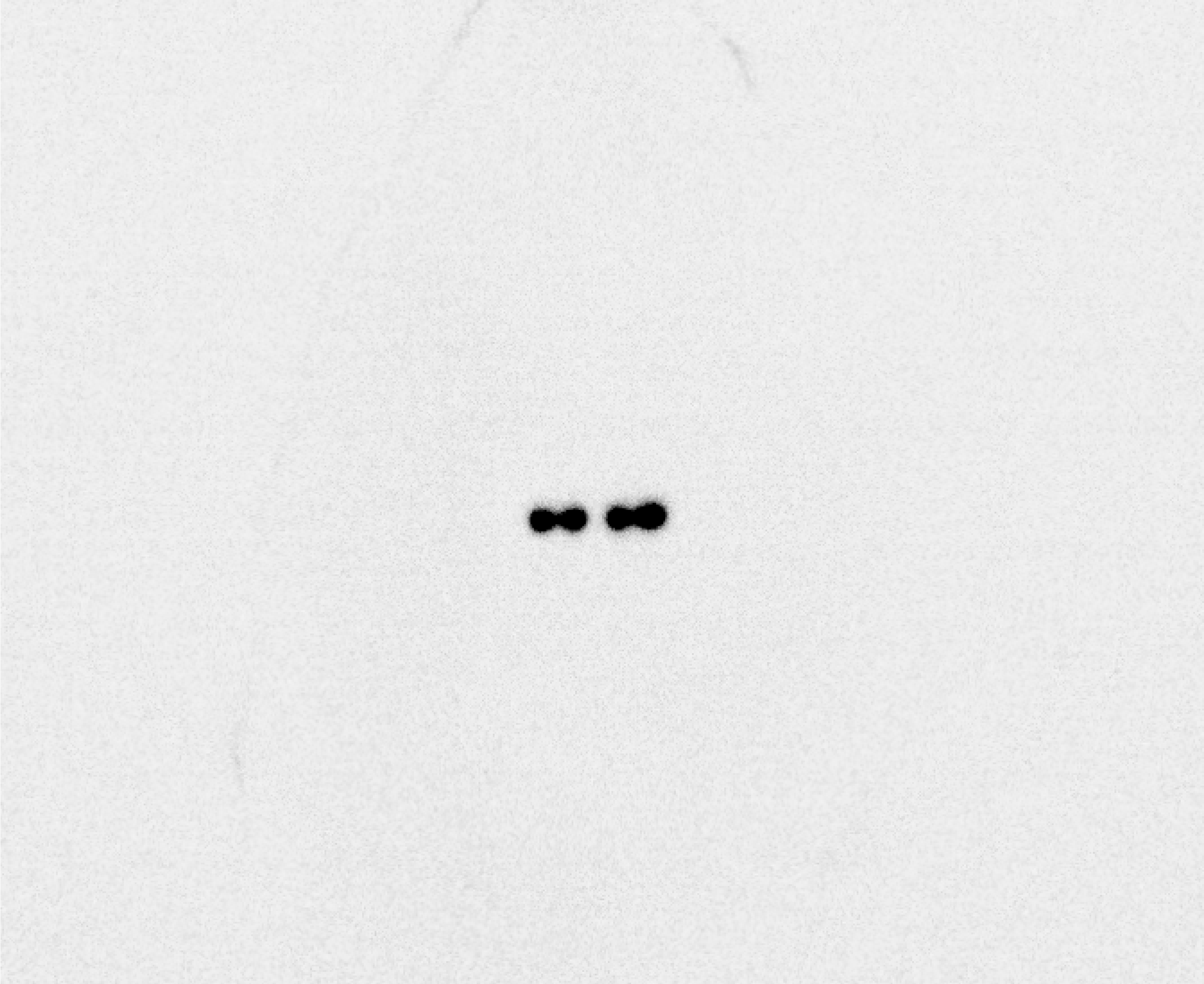

Figure S5C IP MAT2A IgM

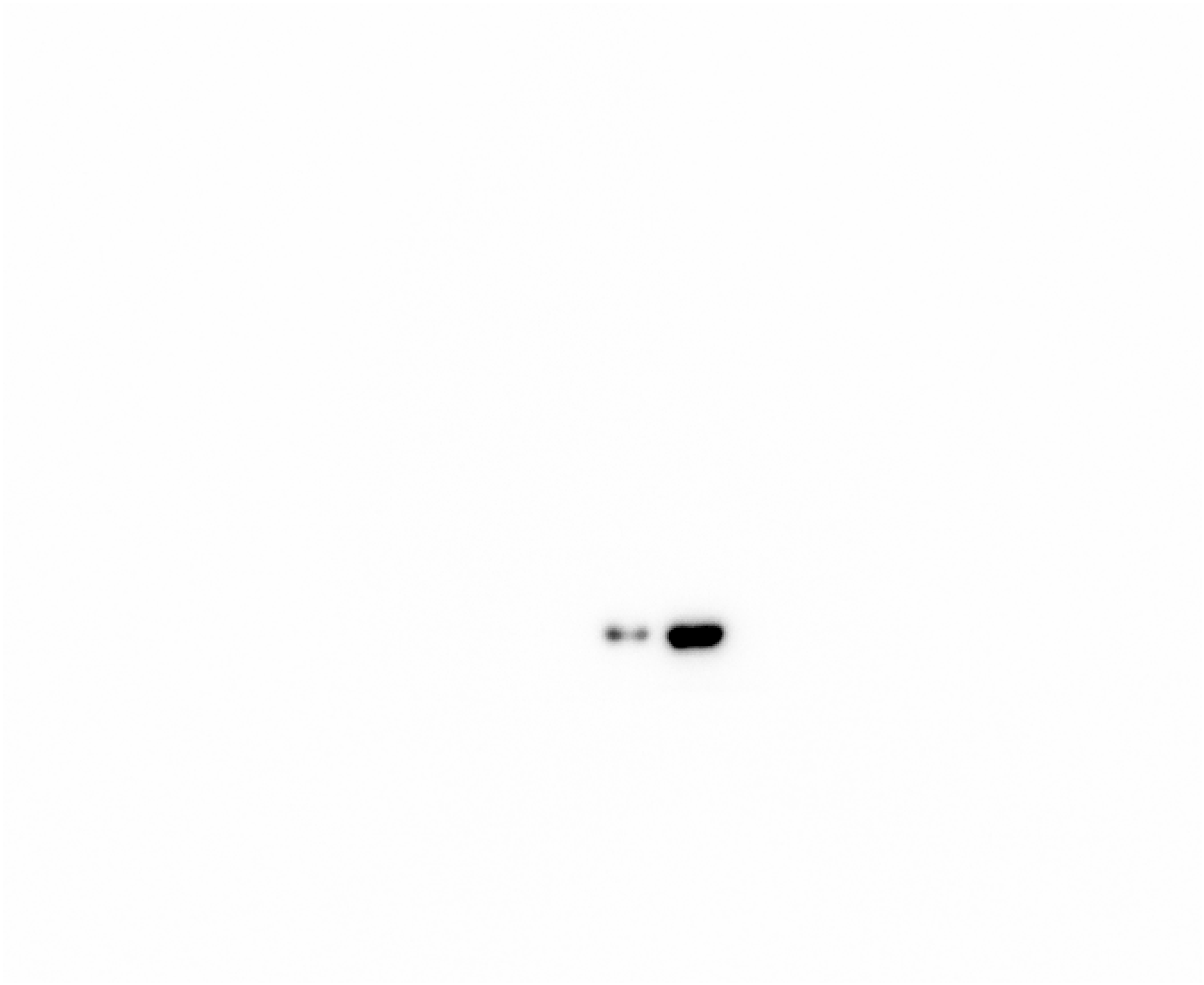

Figure S5C IP MAT2A IgT

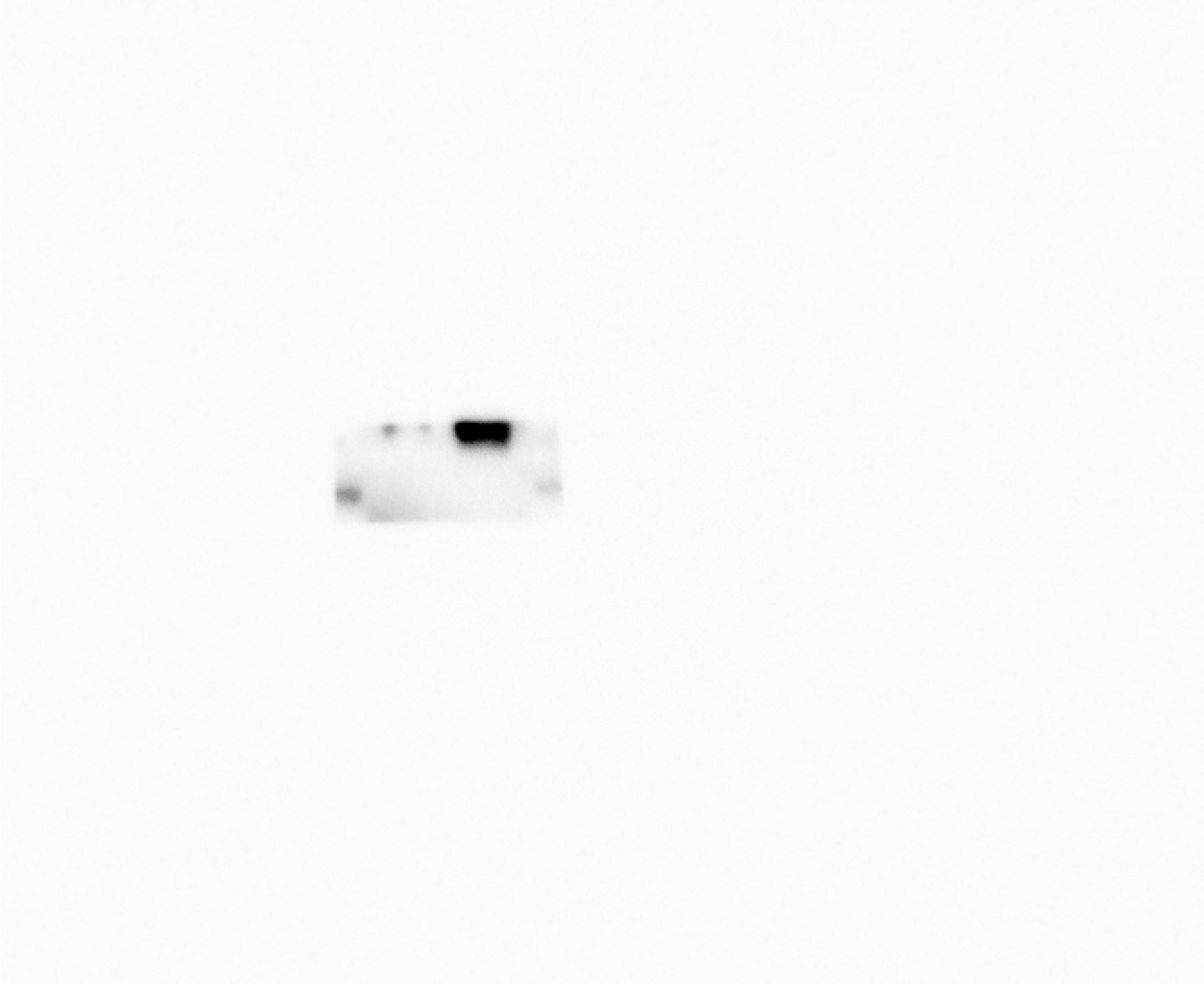

Figure S5C IP TRIM25 IgM

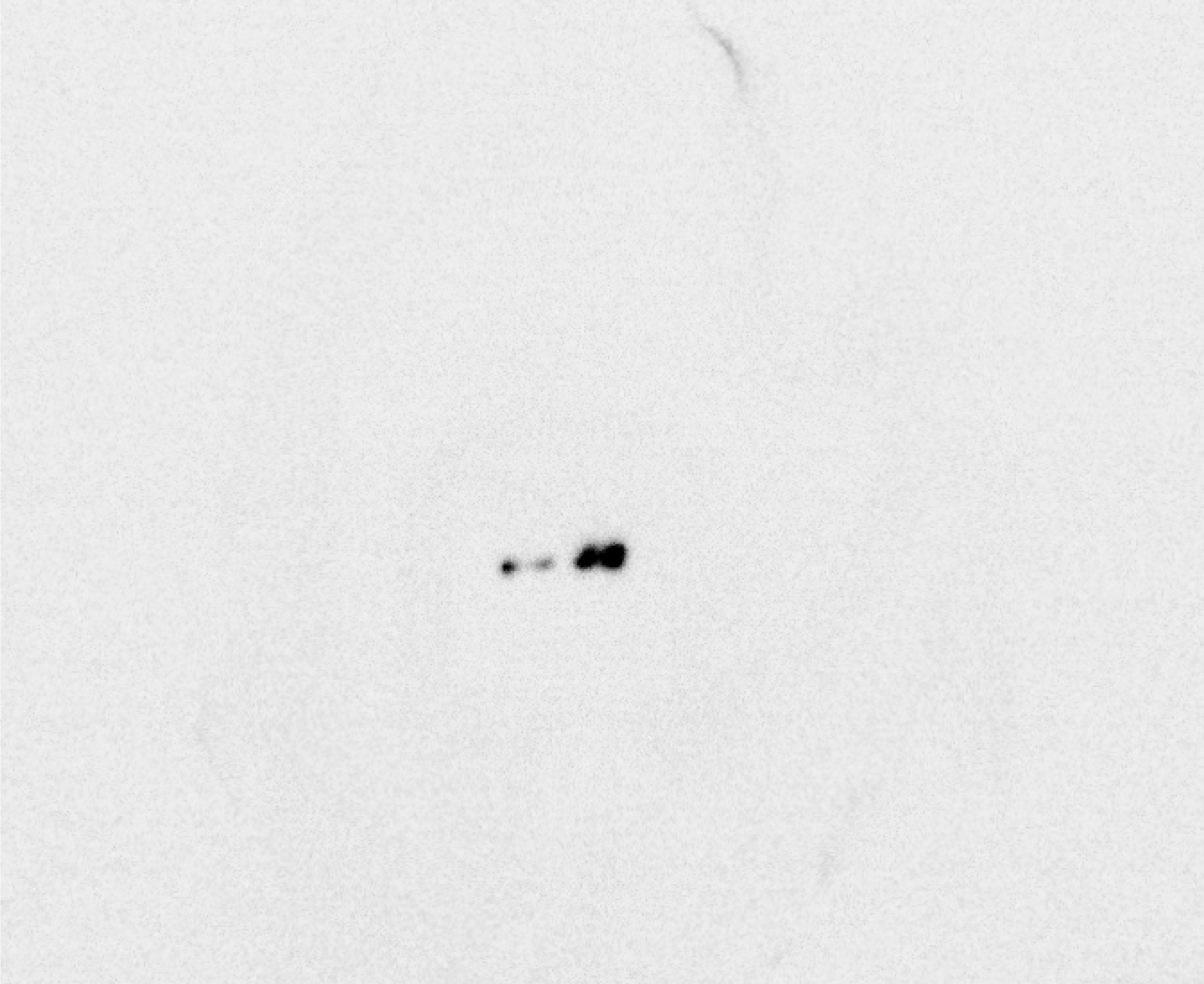

Figure S5F TRIM25 IP

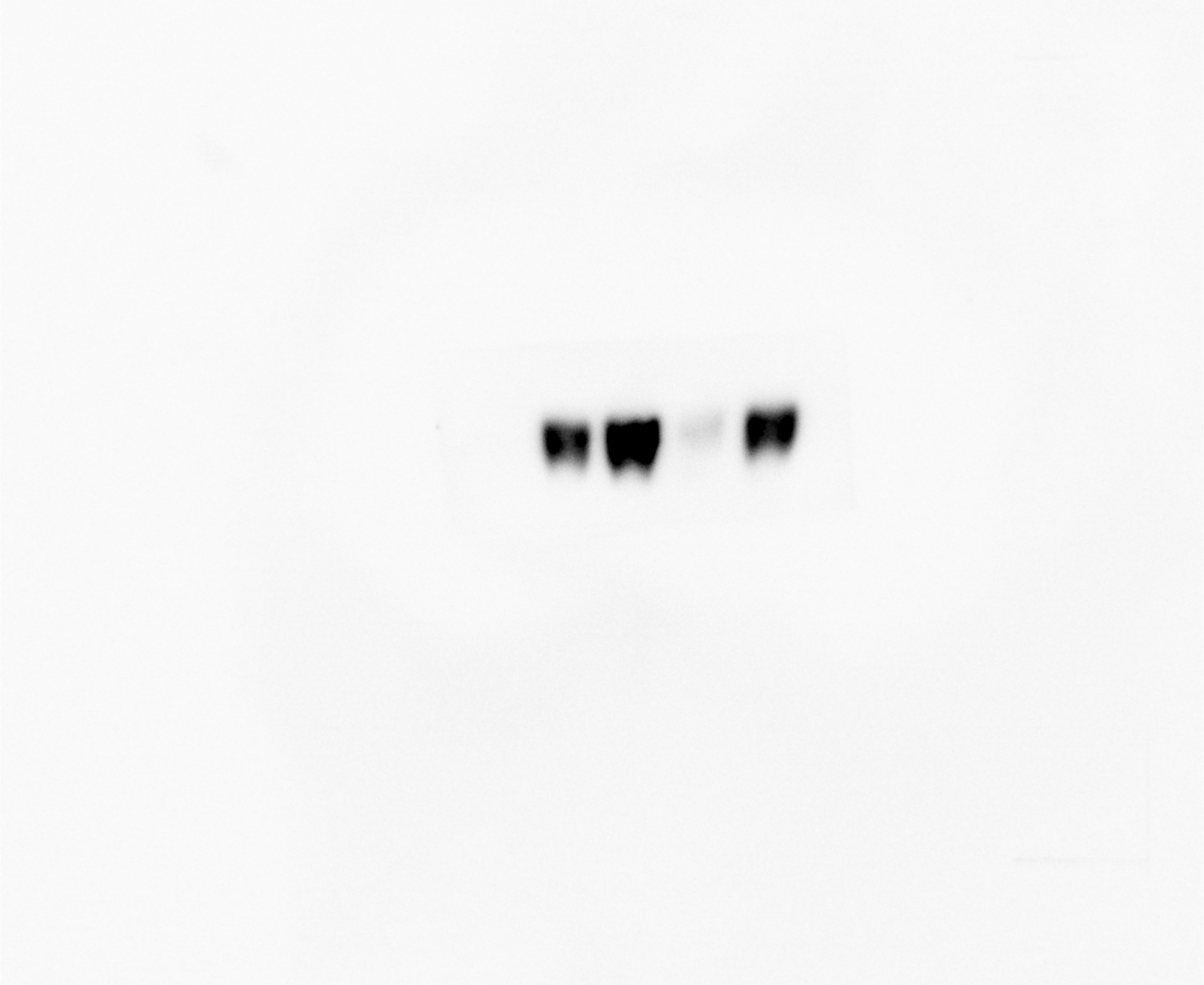

Figure S5F GAPDH

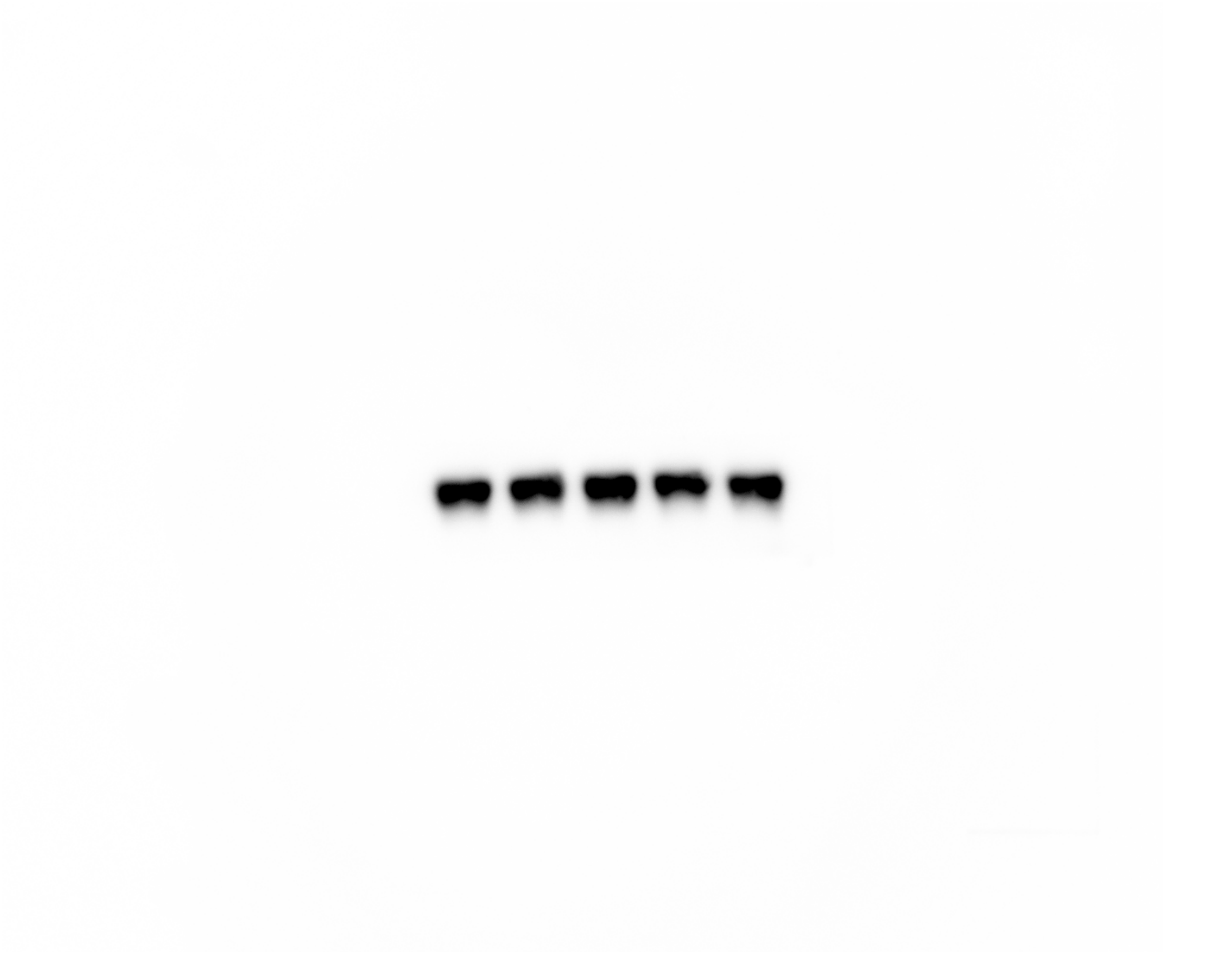

Figure S5F MAT2A Input

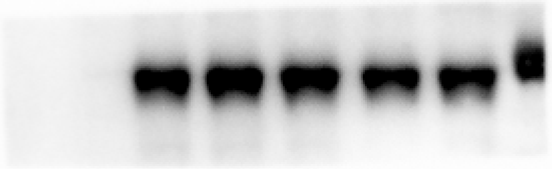

Figure S5F MAT2A IP

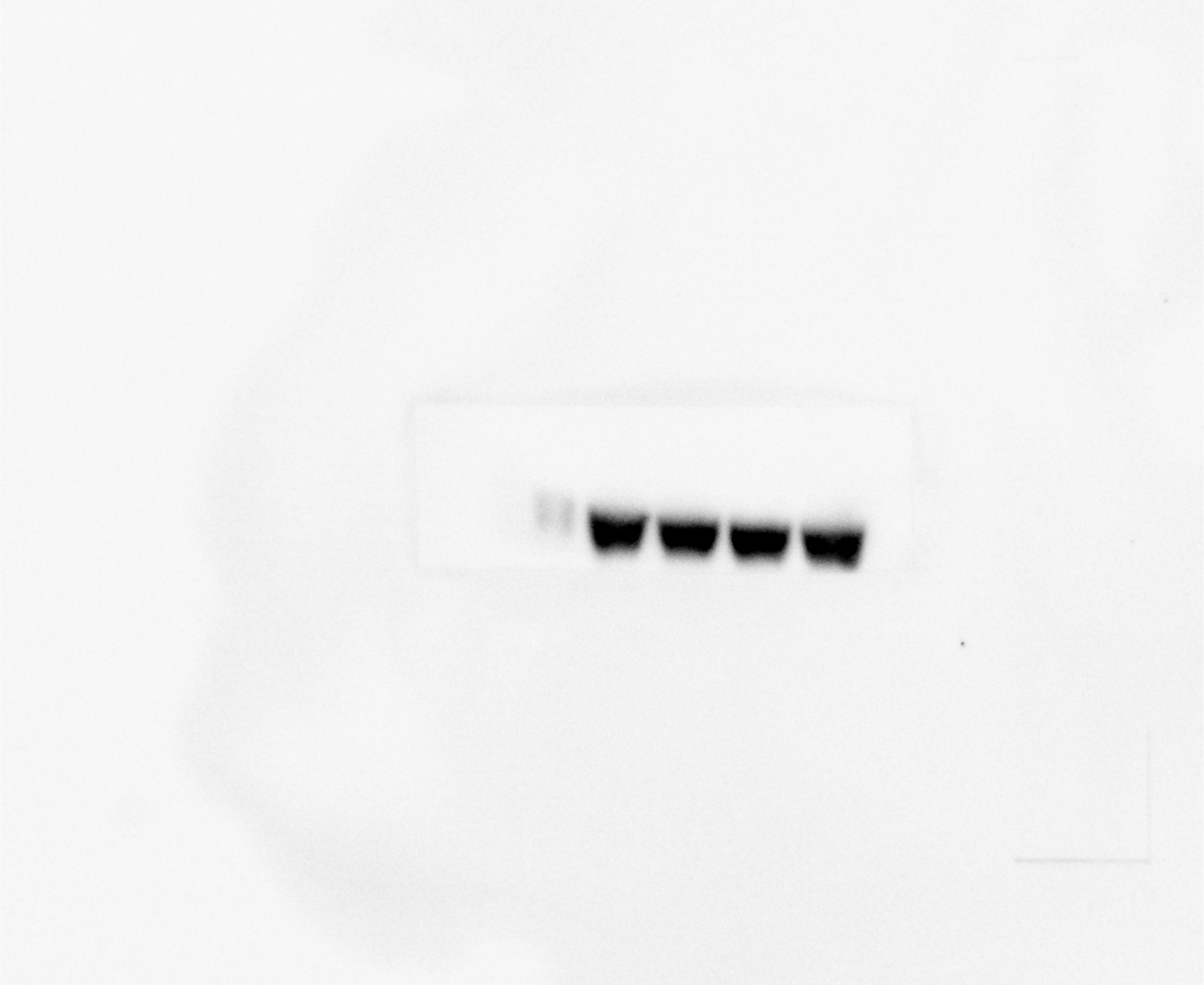

Figure S5F TRIM25 Input

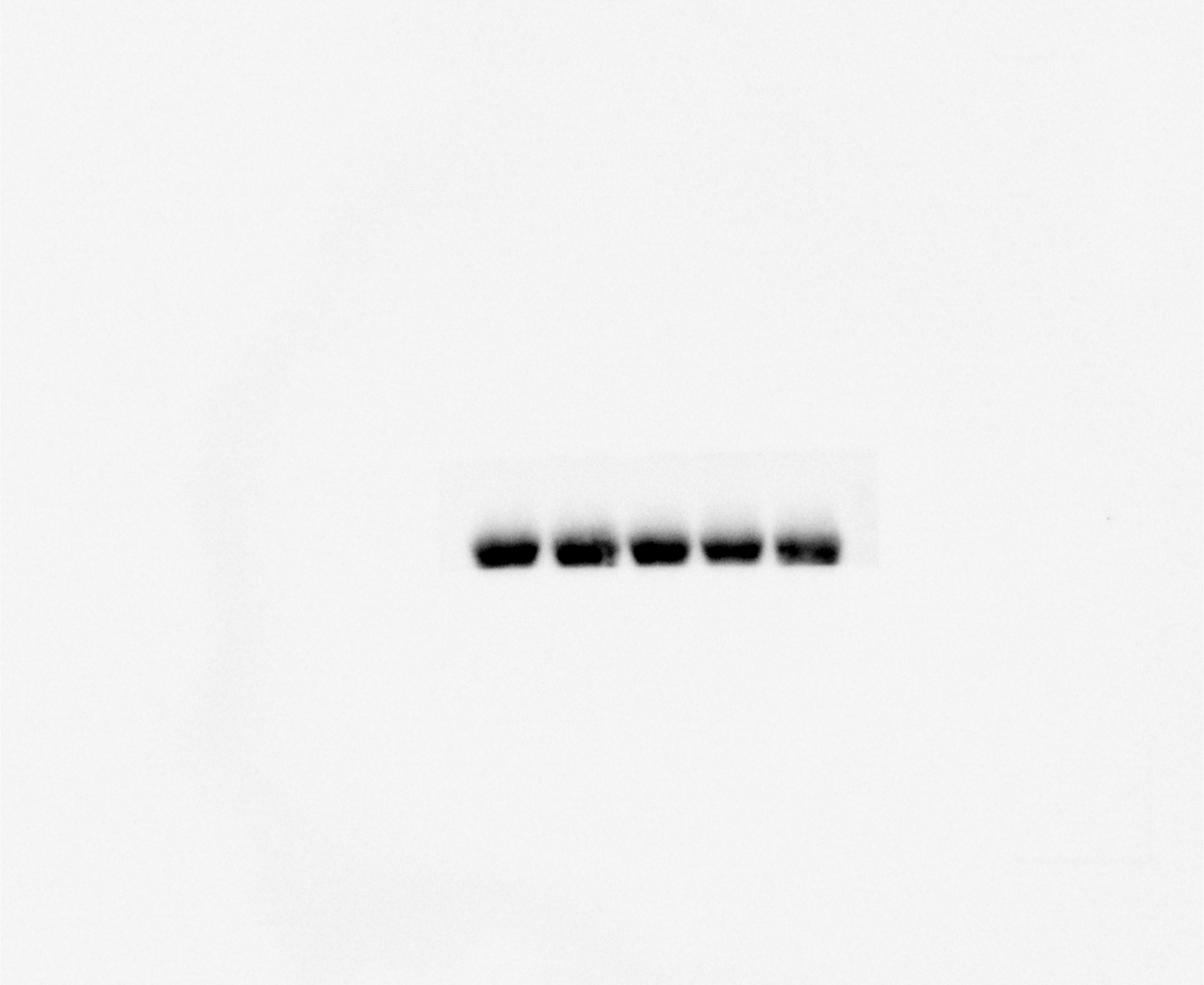

Figure S6 MAT2A UMUC3

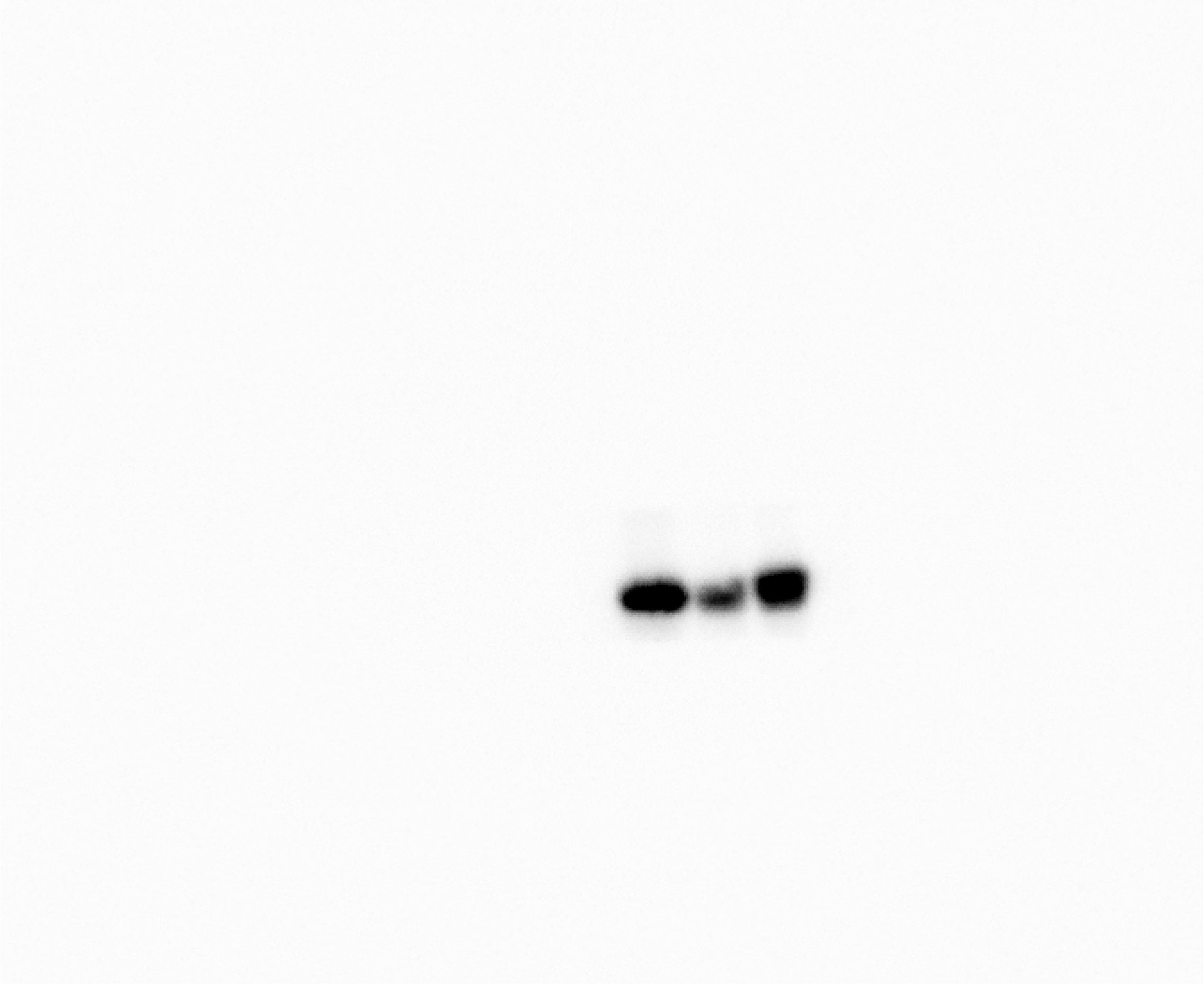

Figure S6 CD44 T24

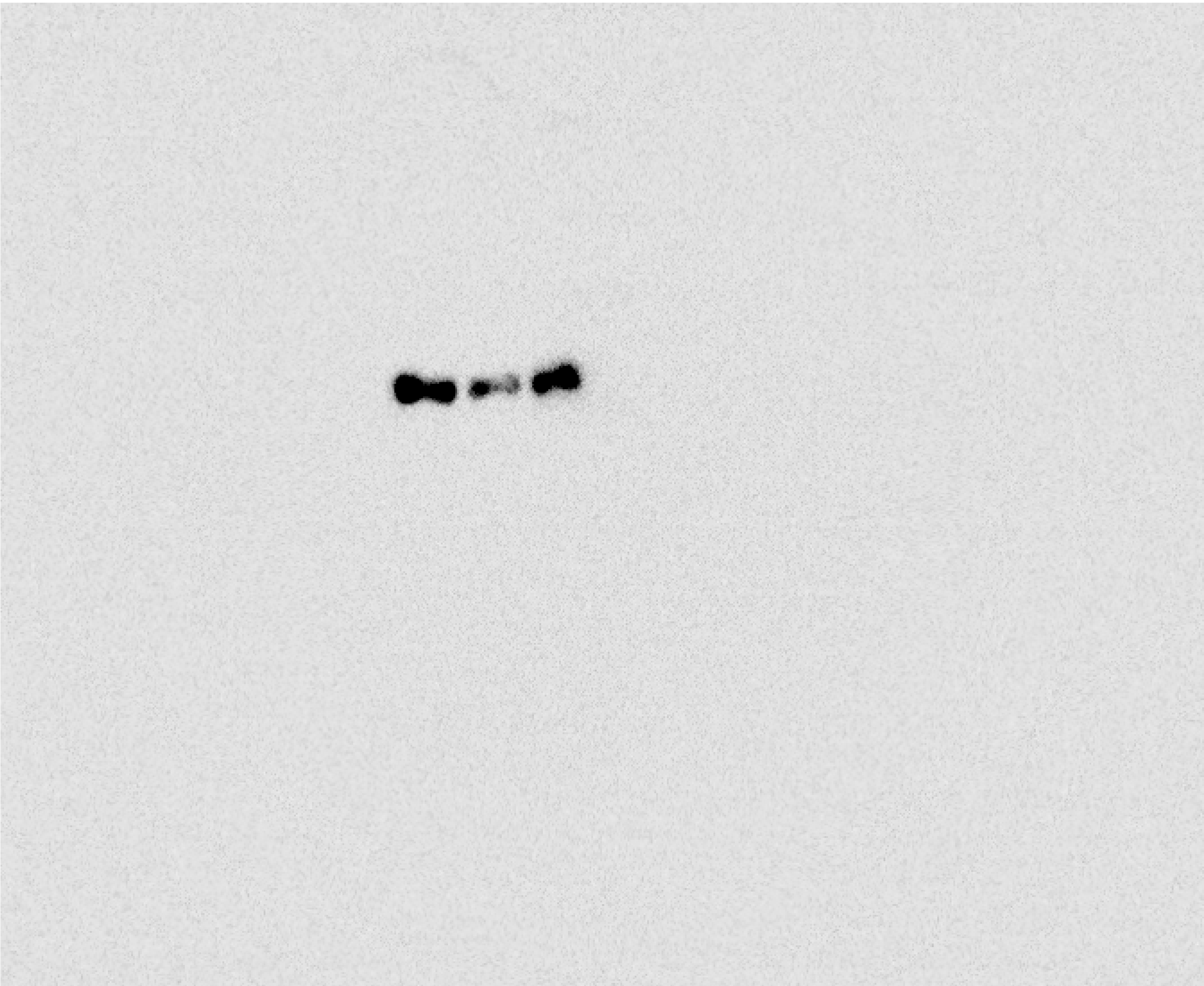

Figure S6 CD44 UMUC3

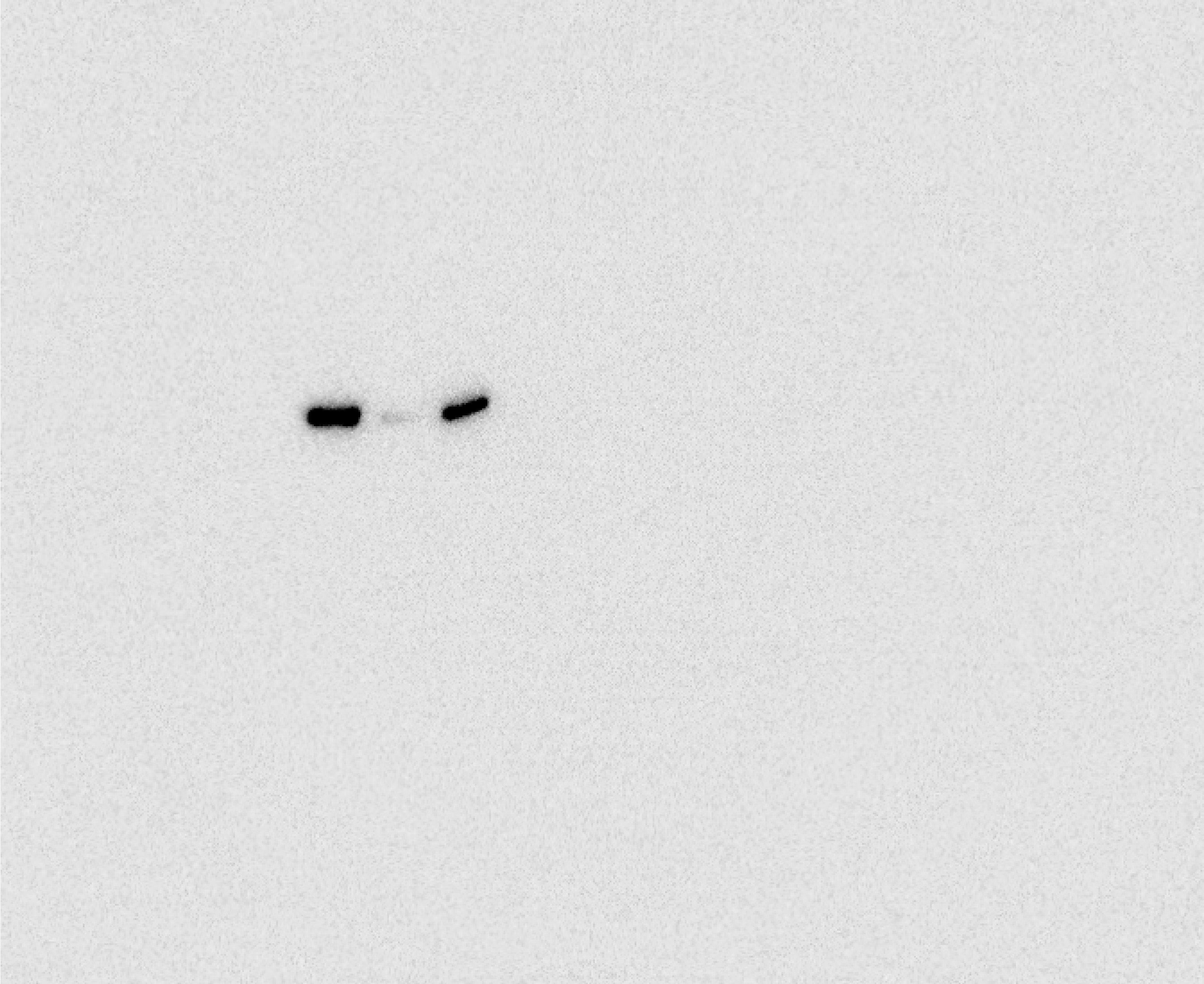

Figure S6 GAPDH T24

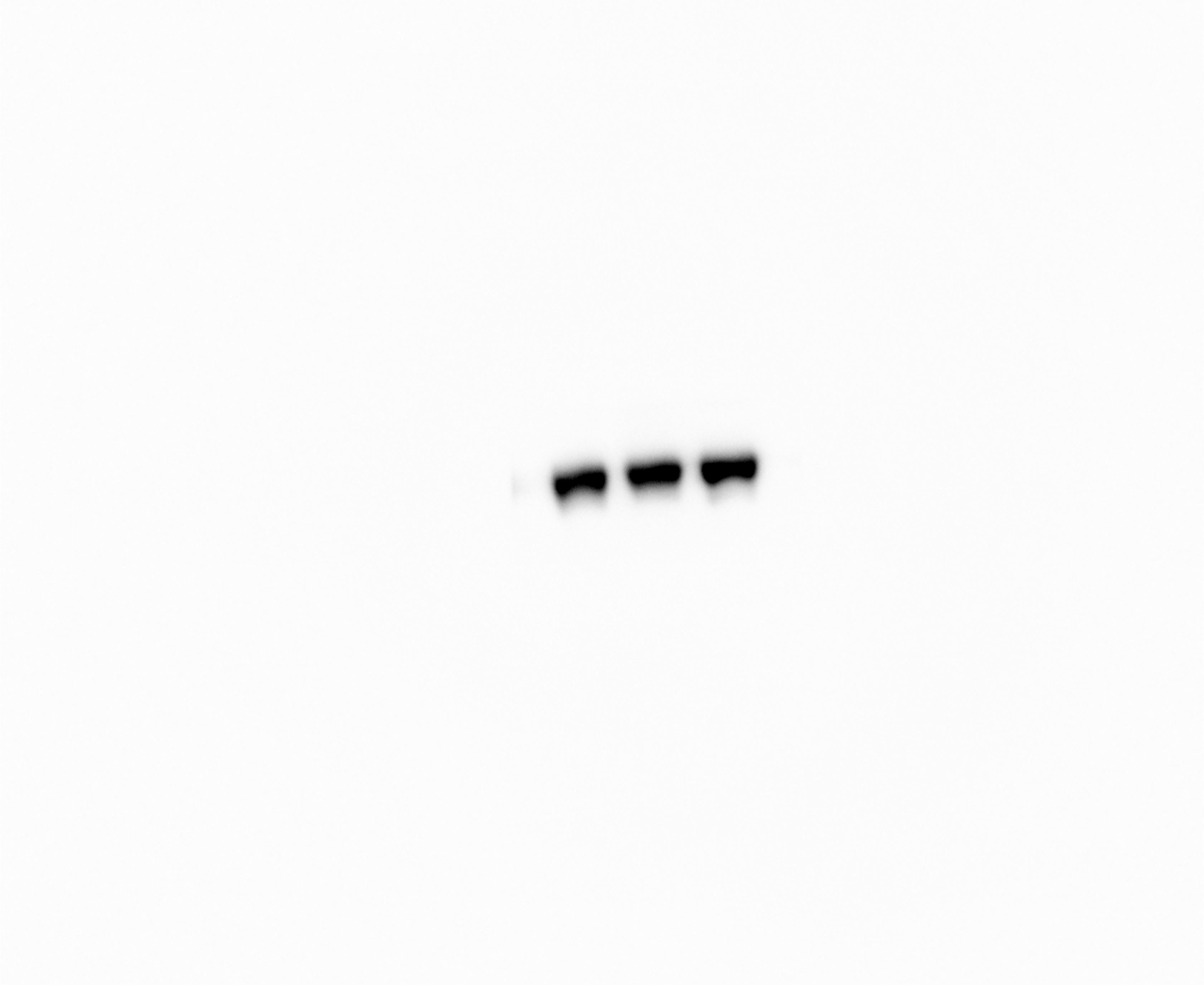

Figure S6 GAPDH UMUC3

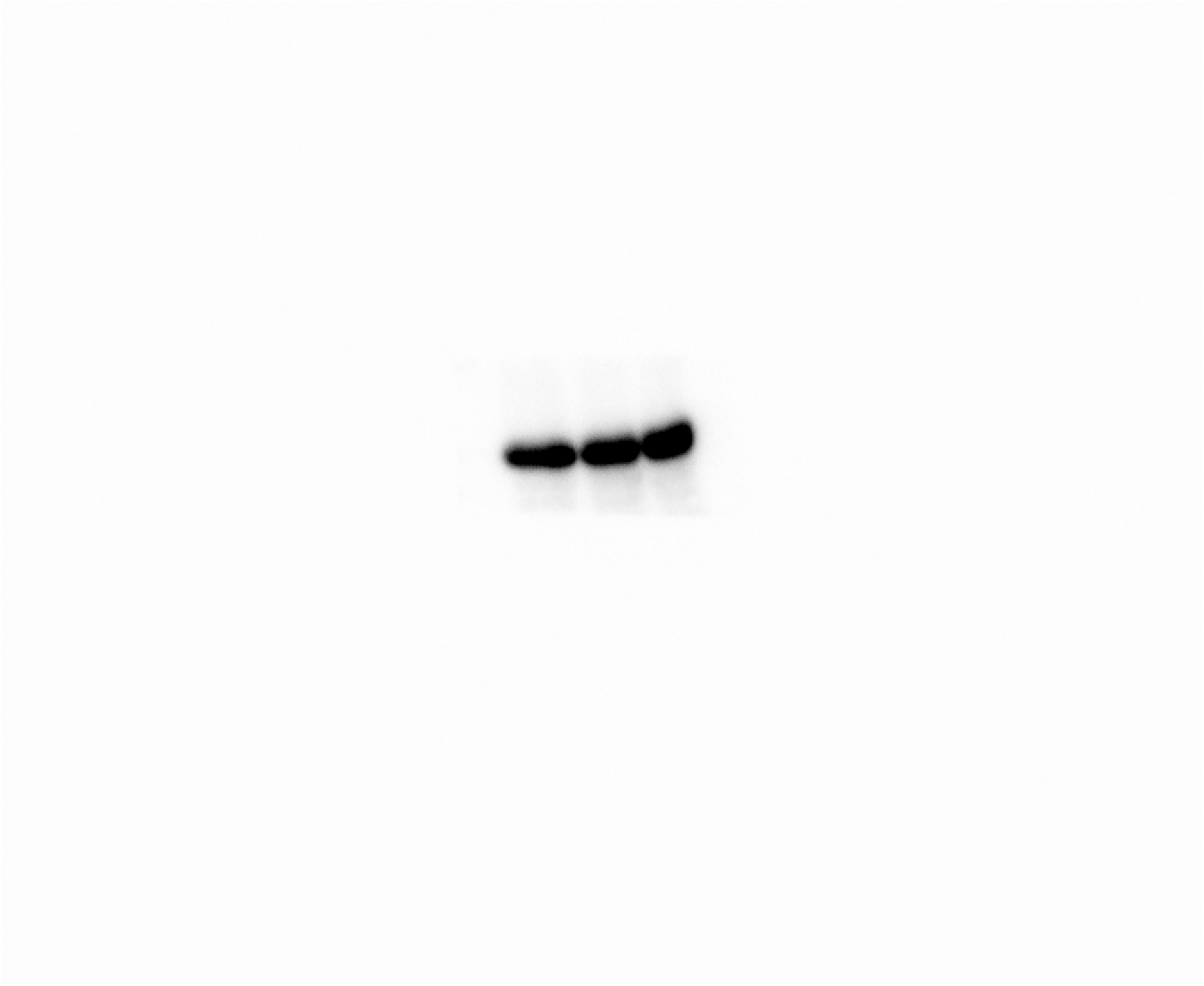

Figure S6 H3 T24

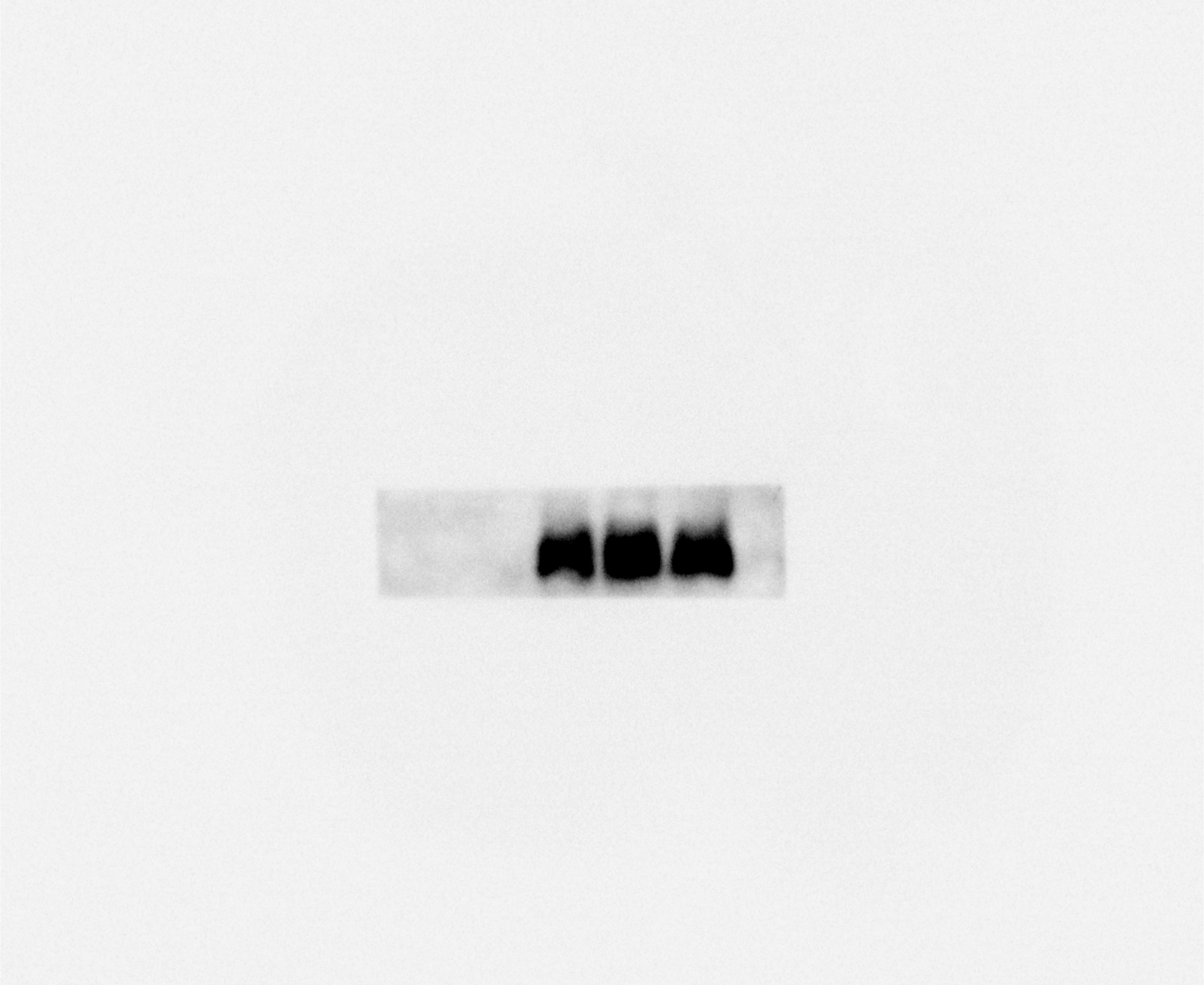

Figure S6 H3 UMUC3

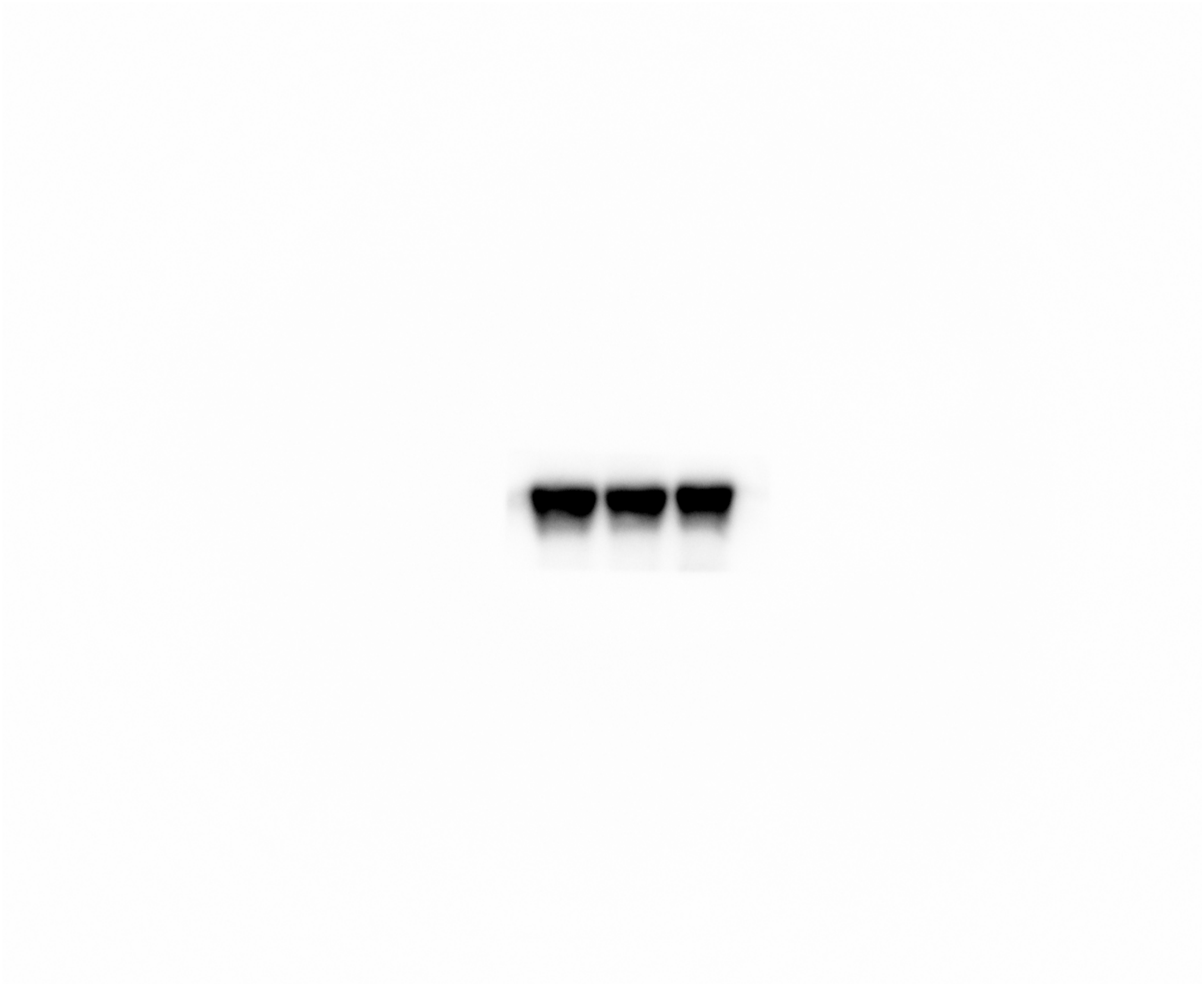

Figure S6 H3K T24

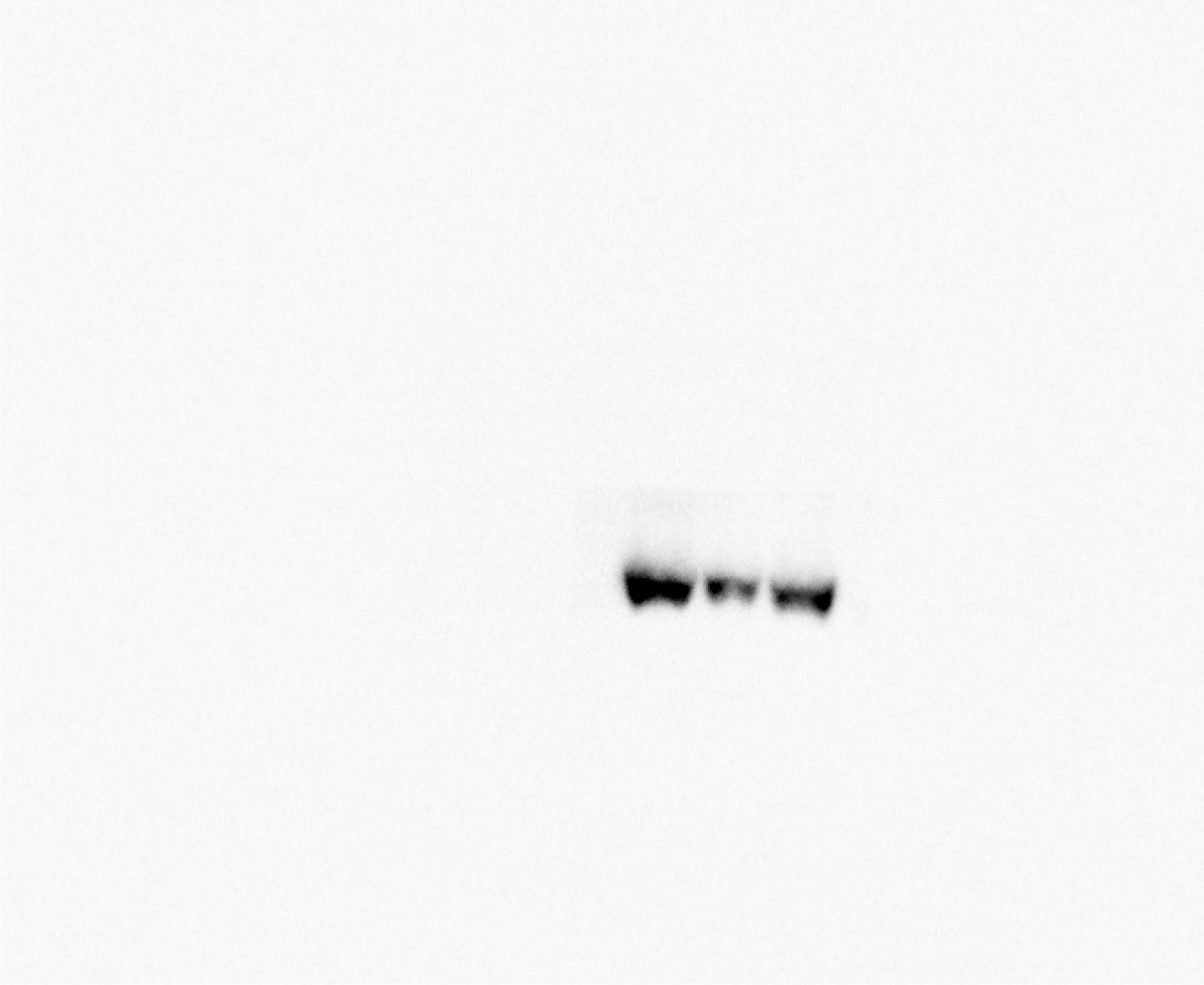

Figure S6 H3K UMUC3

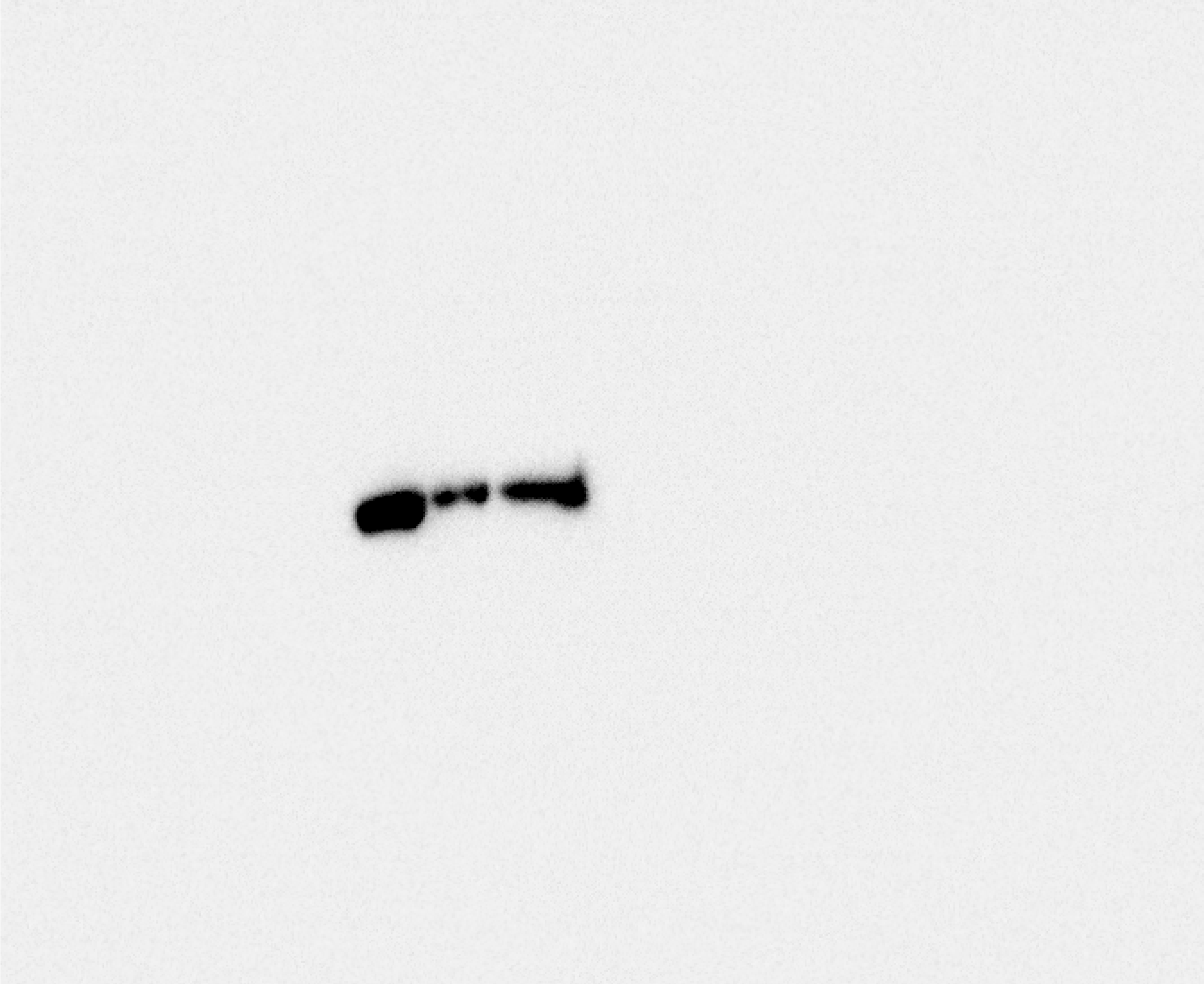

Figure S6 MAT2A T24

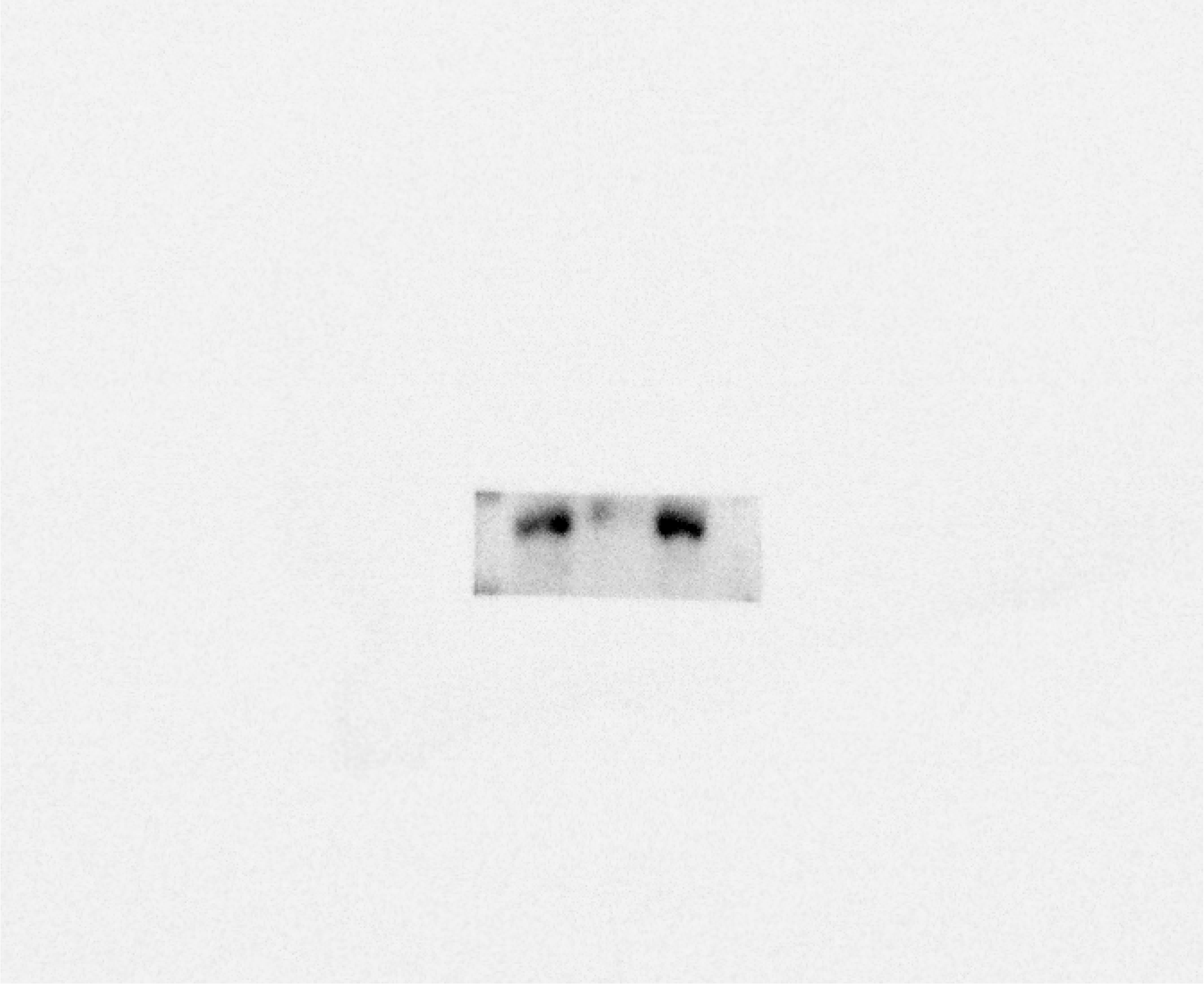

Figure-S8E-Total-H3

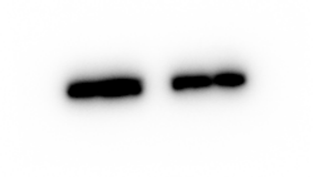

Figure-S8E-CD44

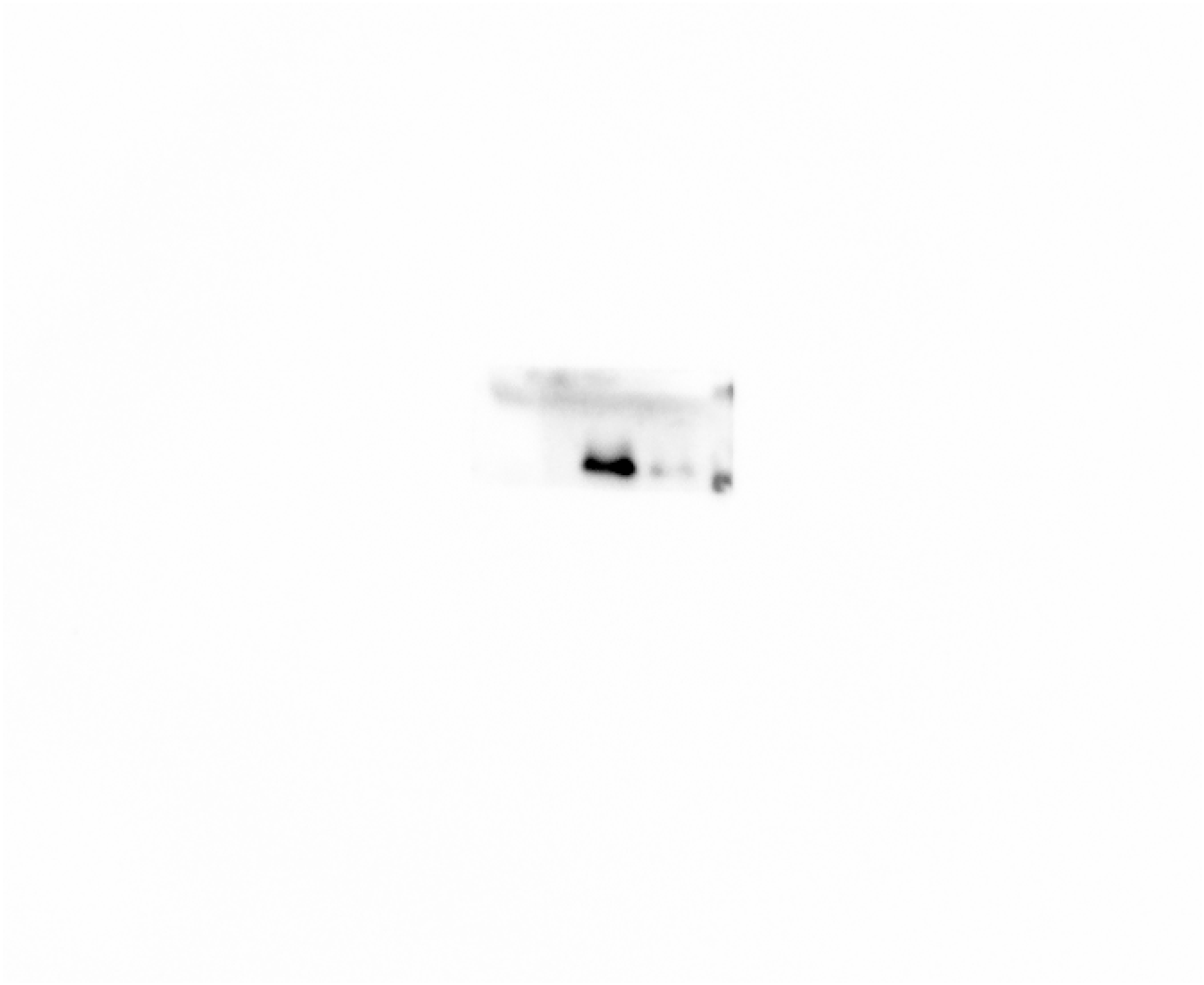

Figure-S8E-GAPDH

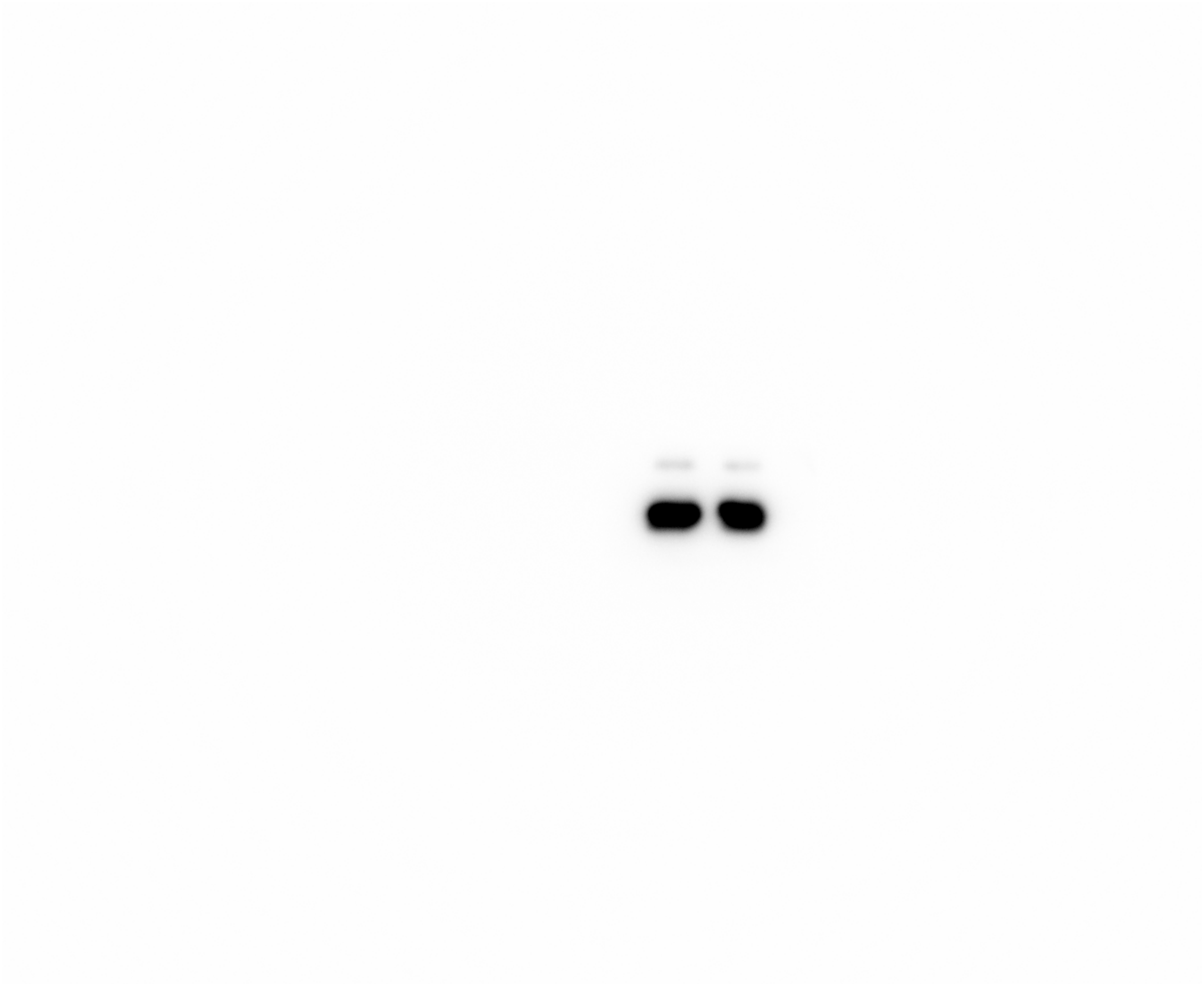

Figure-S8E-H3K

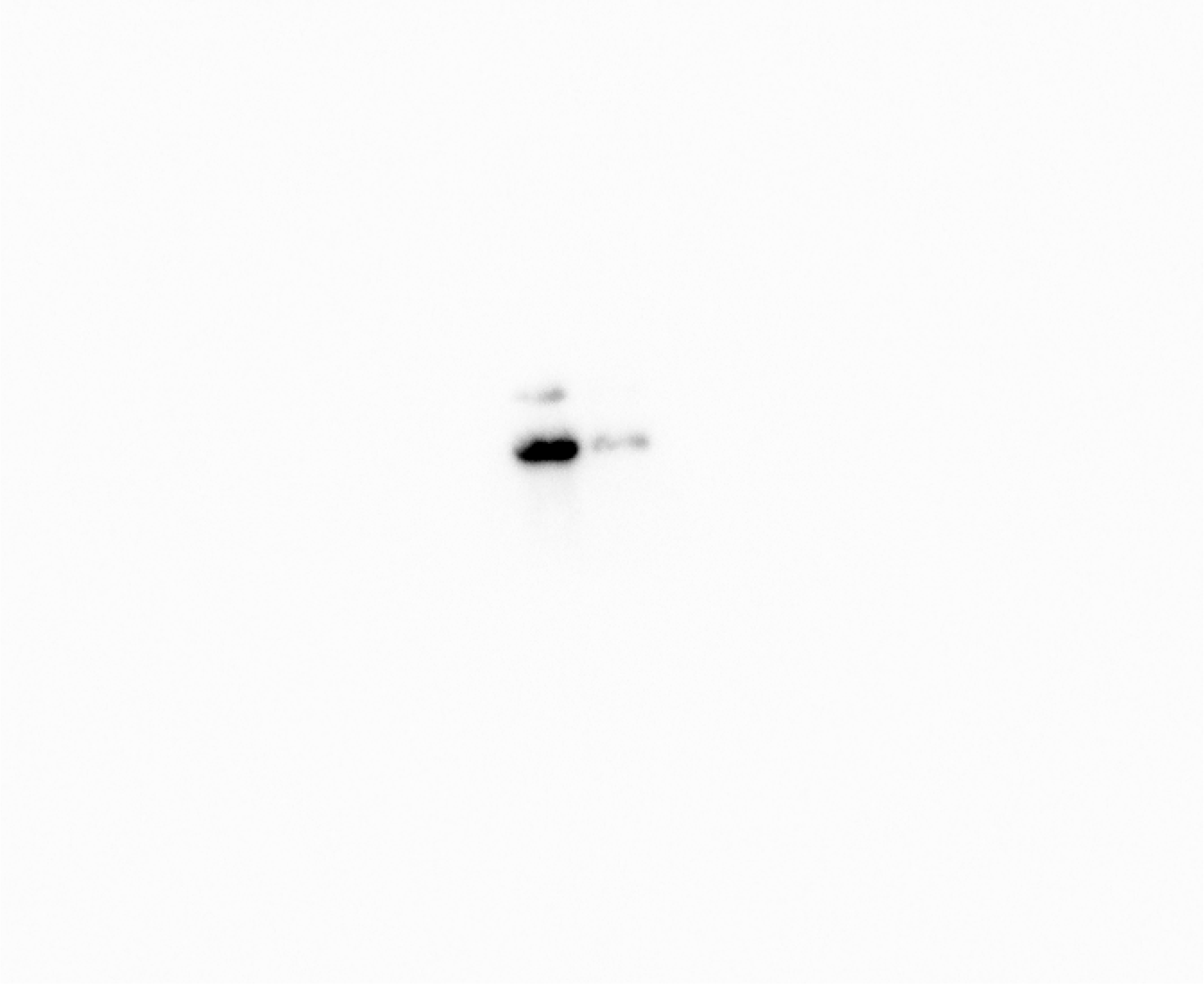

Figure-S8E-MAT2A

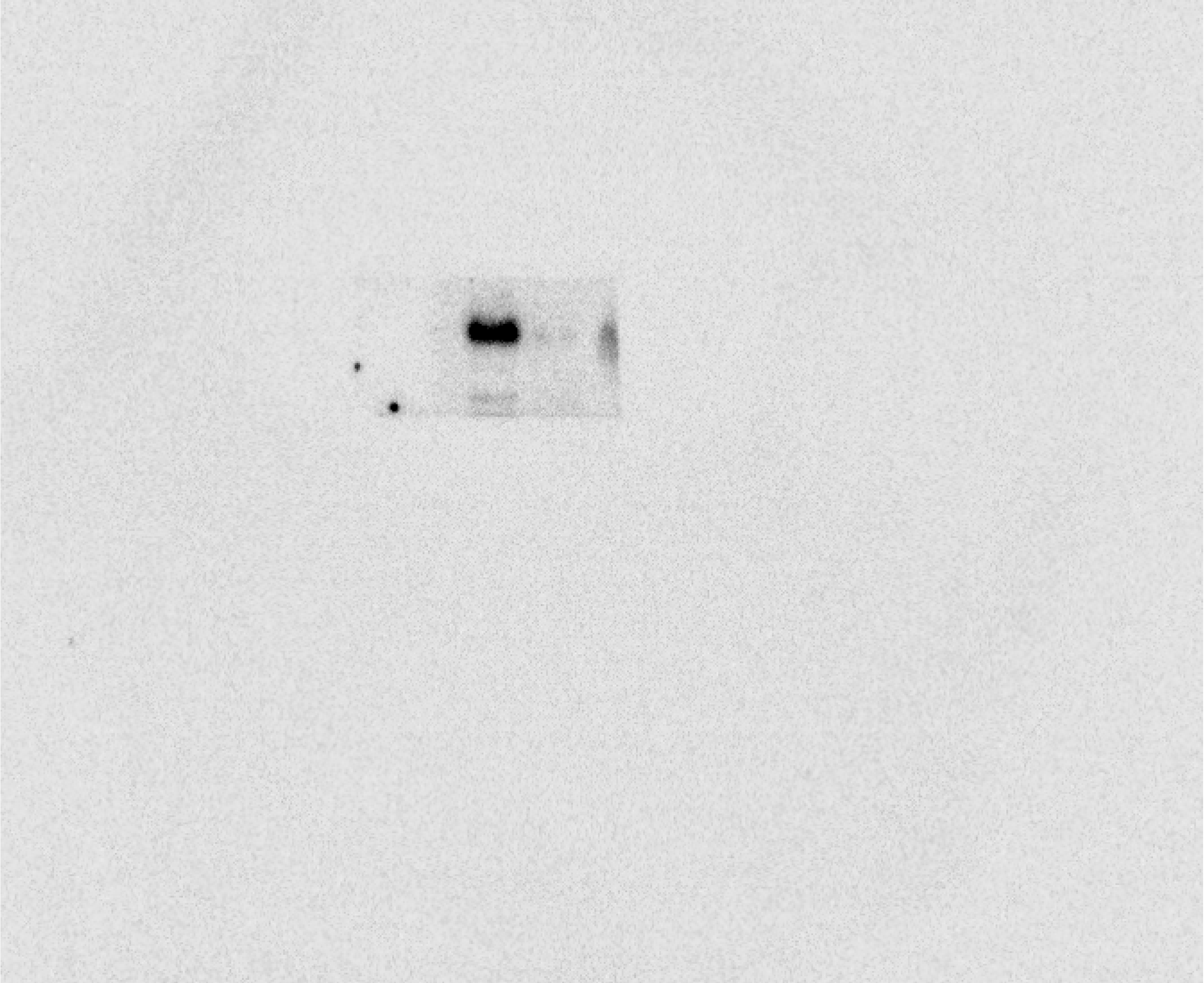

Figure-S8G-TRIM25

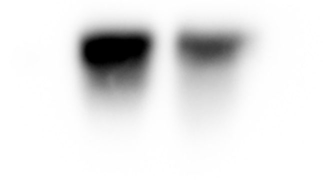

Figure-S8G-GAPDH

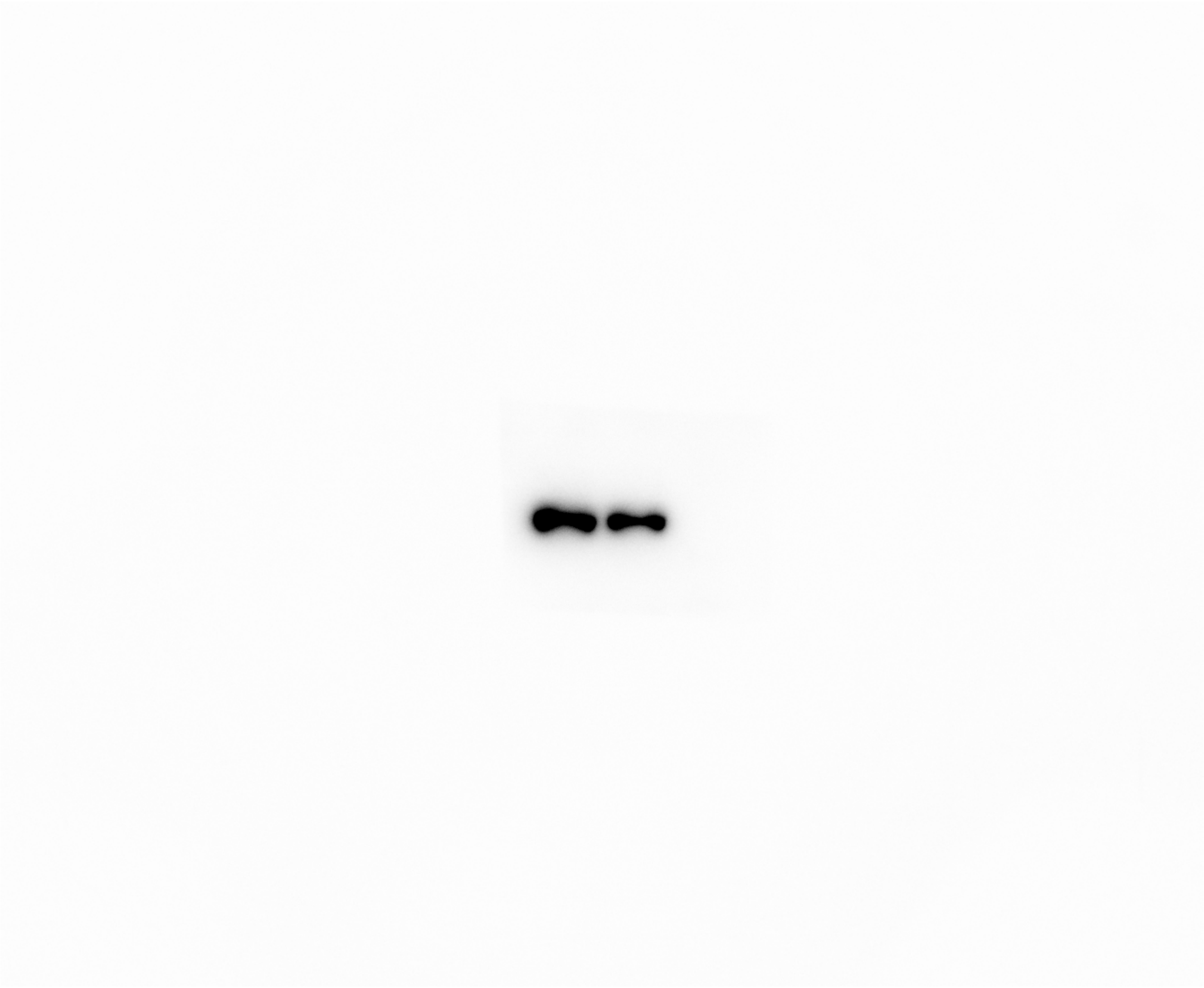

Figure-S8G-MAT2A

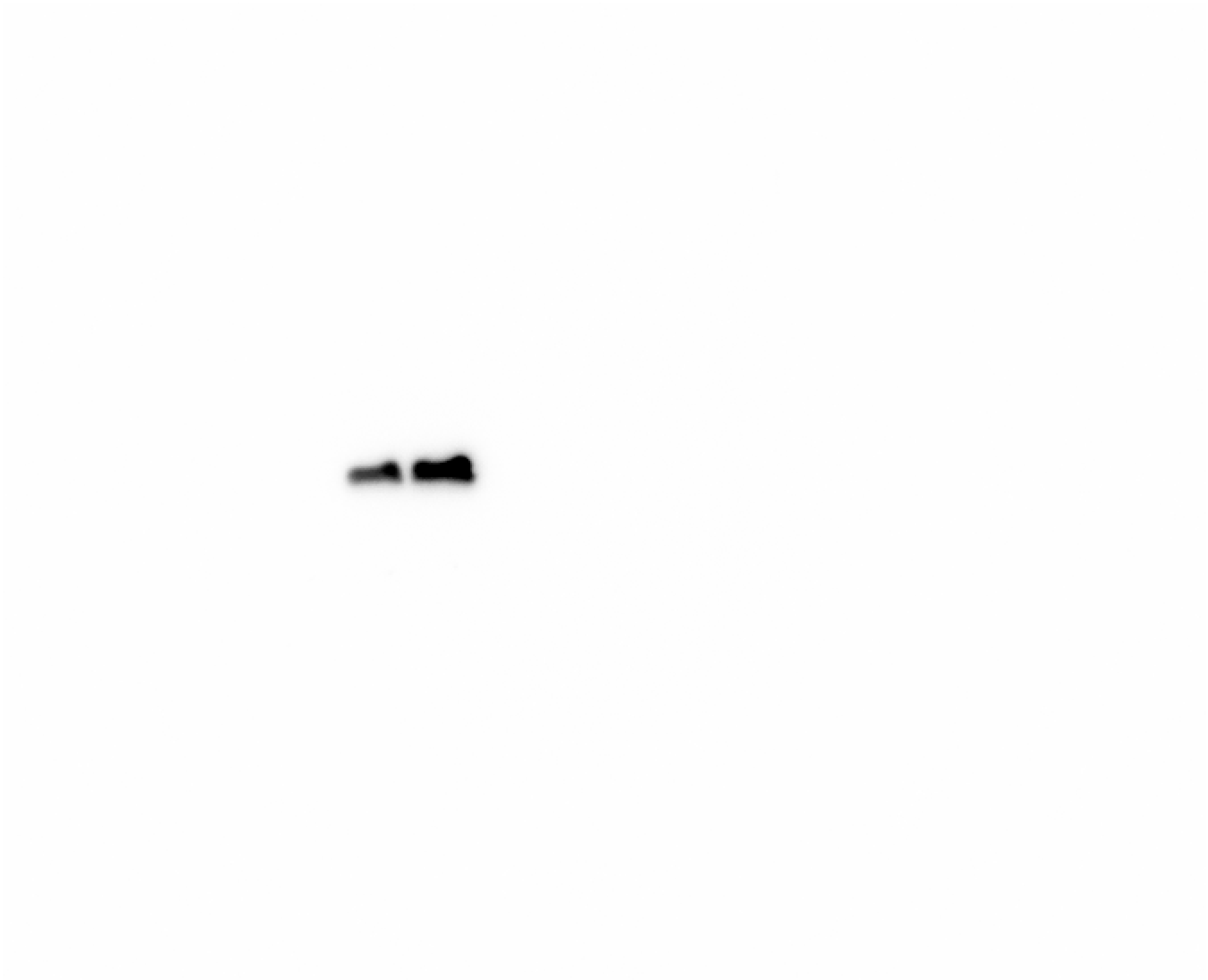

Figure-S8H-MAT2A

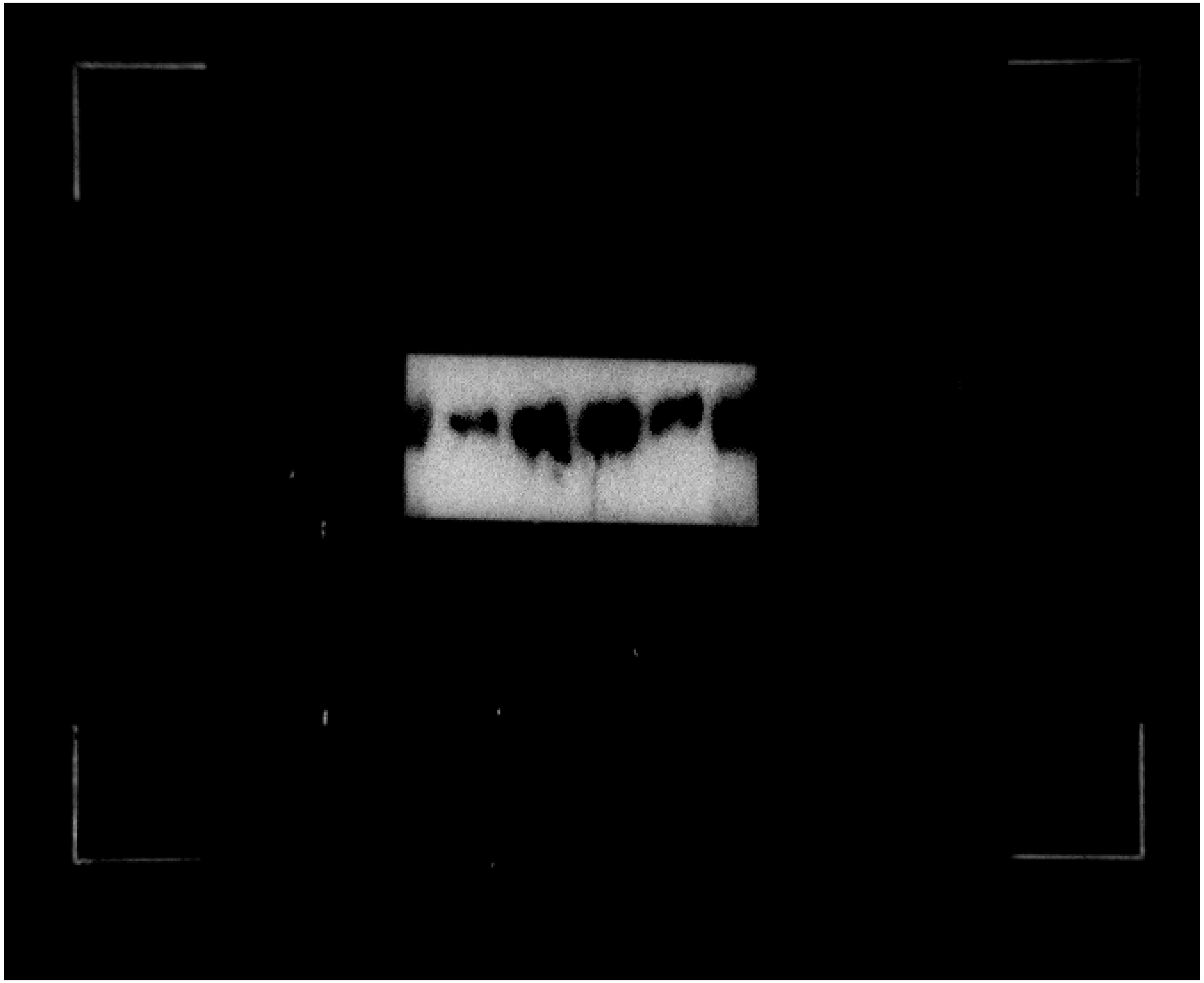

Figure-S8H-CD44

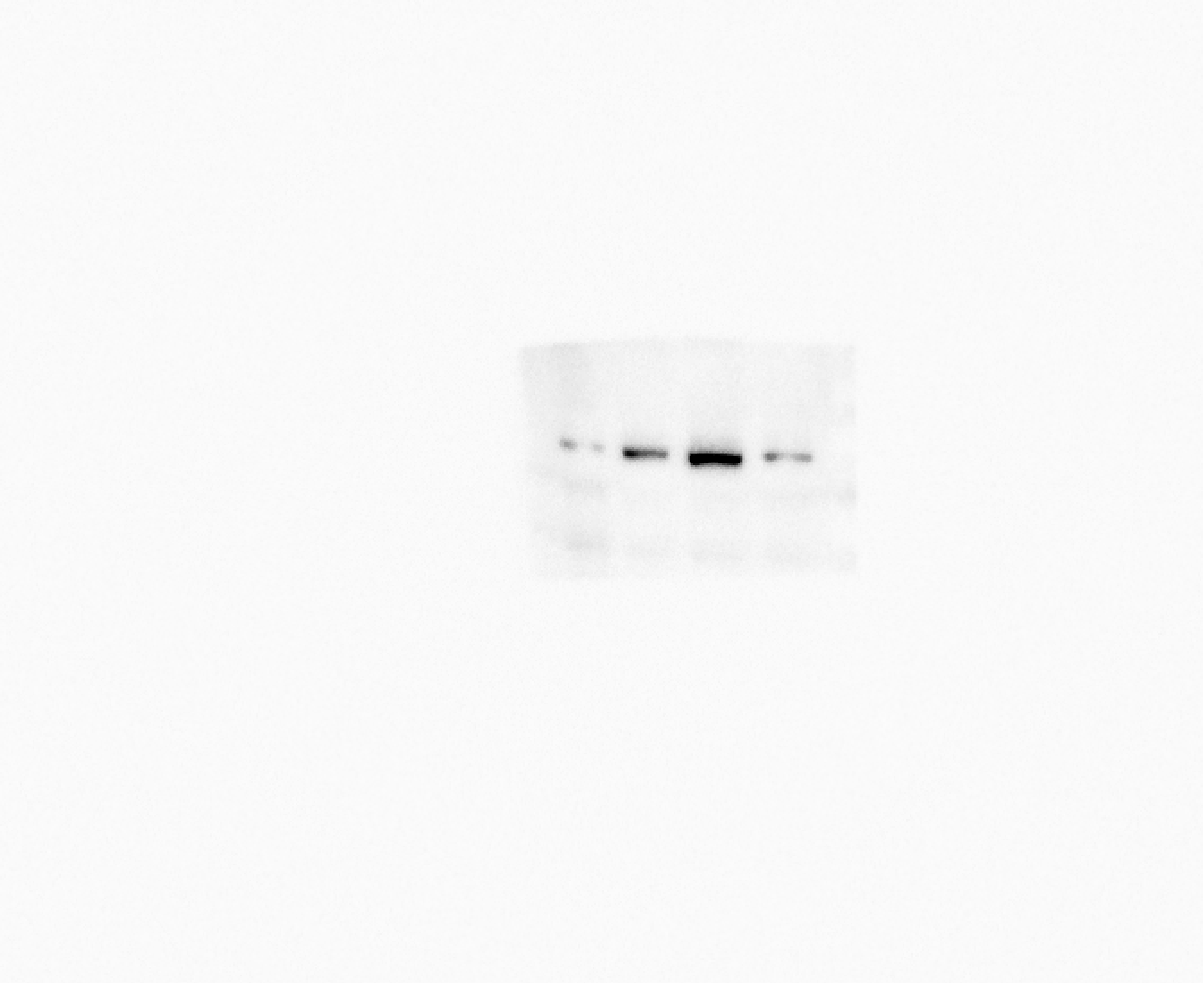

Figure-S8H-GAPDH

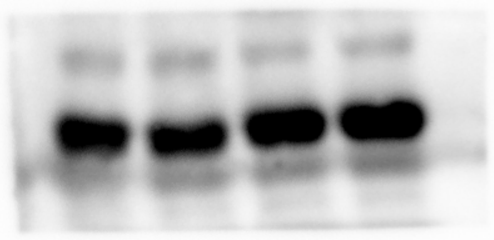

Figure-S8H-H3

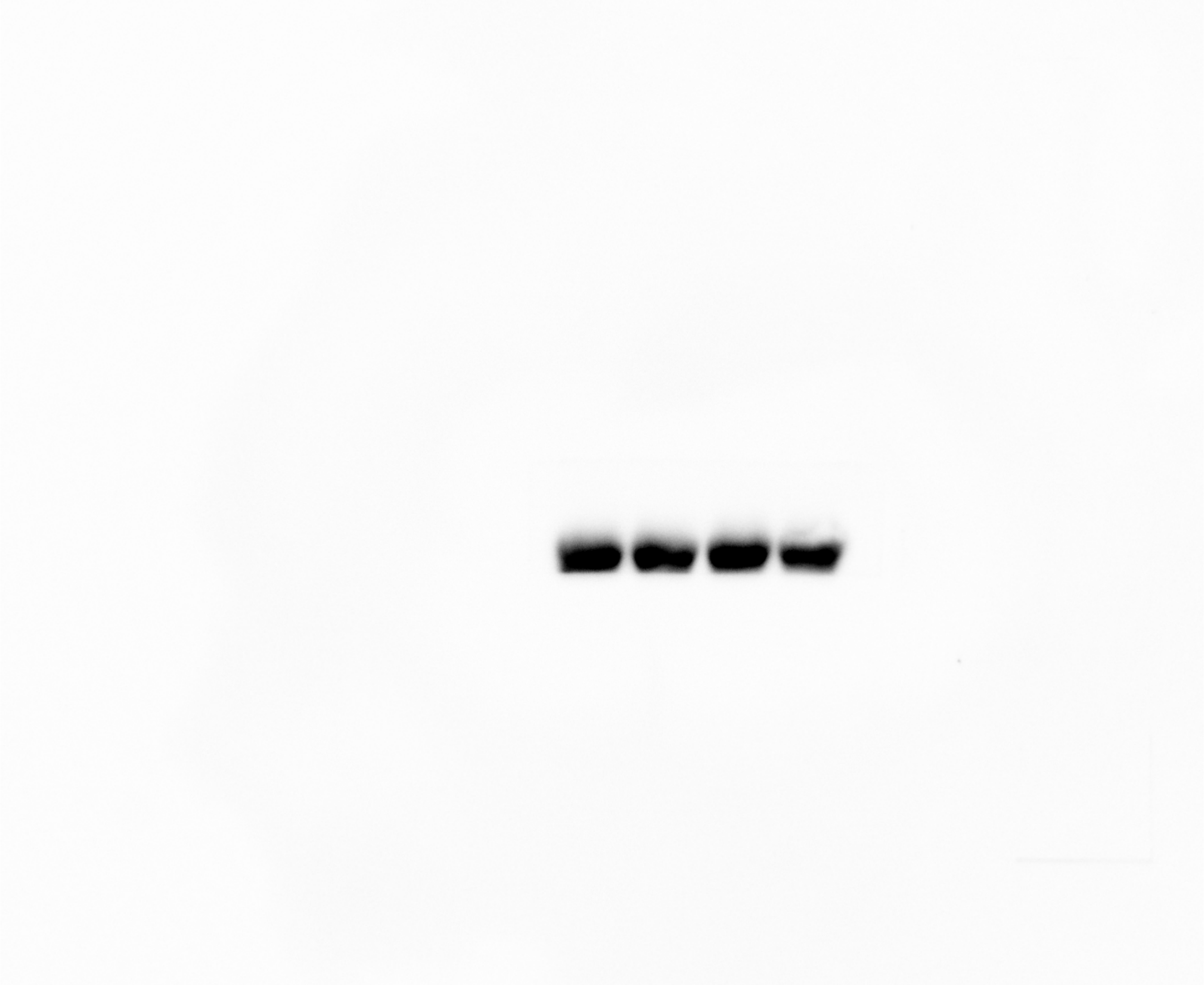

Figure-S8H-H3K

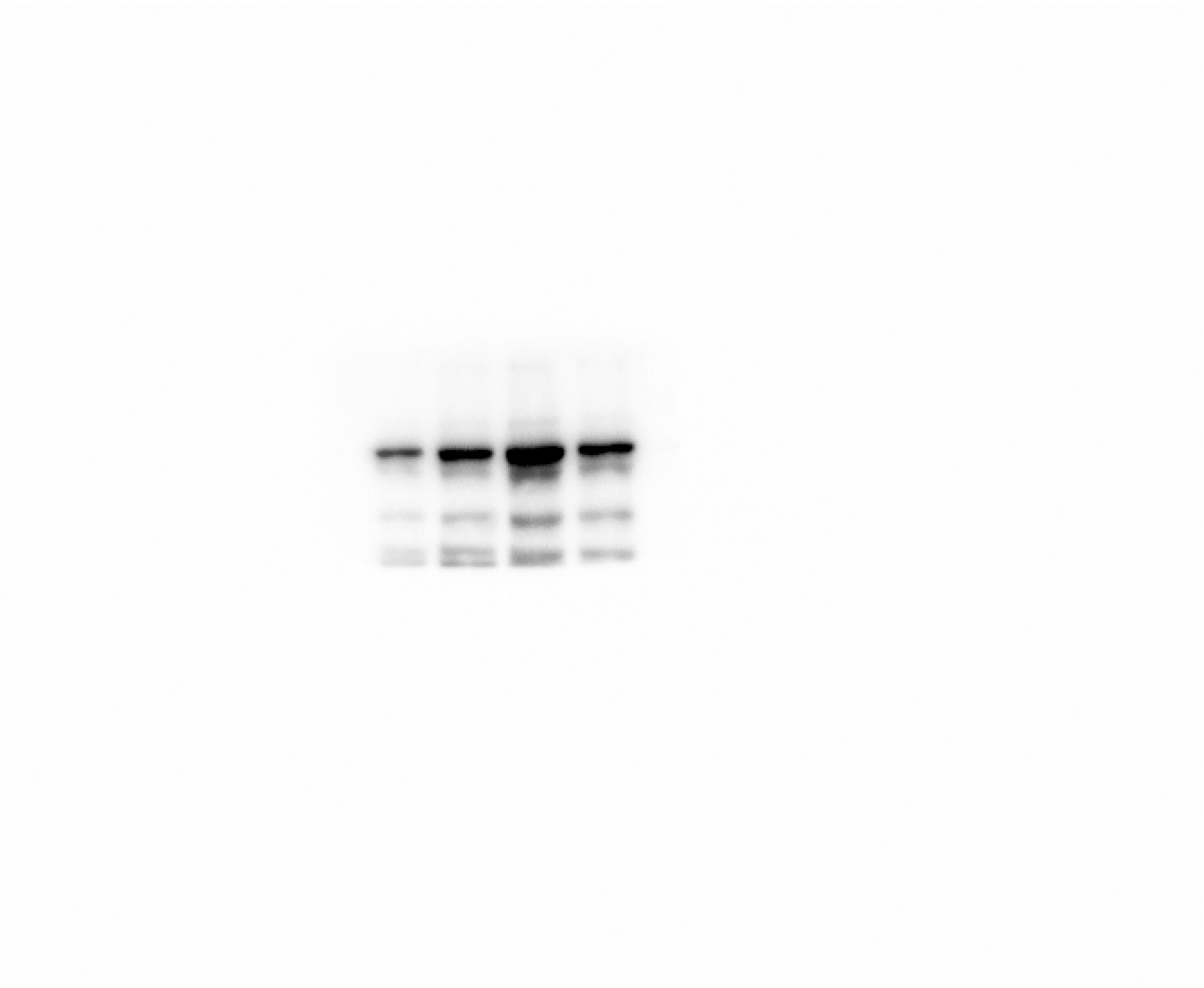

Figure-S8J-MAT2A

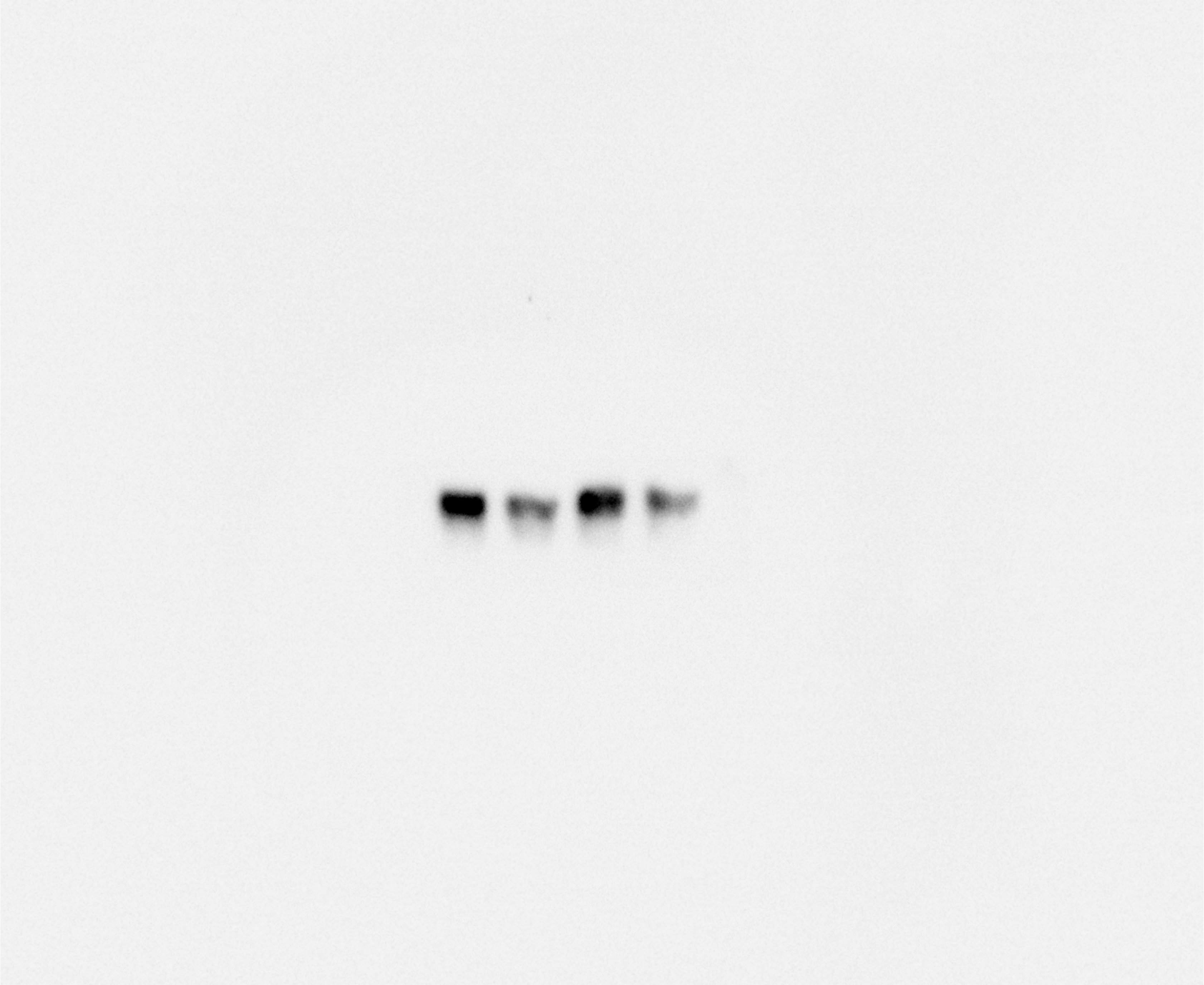

Figure-S8J-CD44

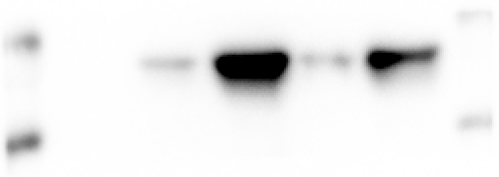

Figure-S8J-GAPDH

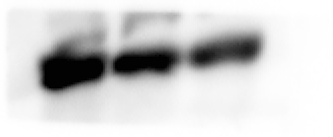

Figure-S8J-H3

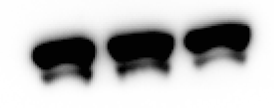

Figure-S8J-H3K

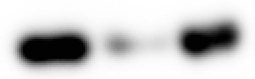

Figure-S8L-MAT2A

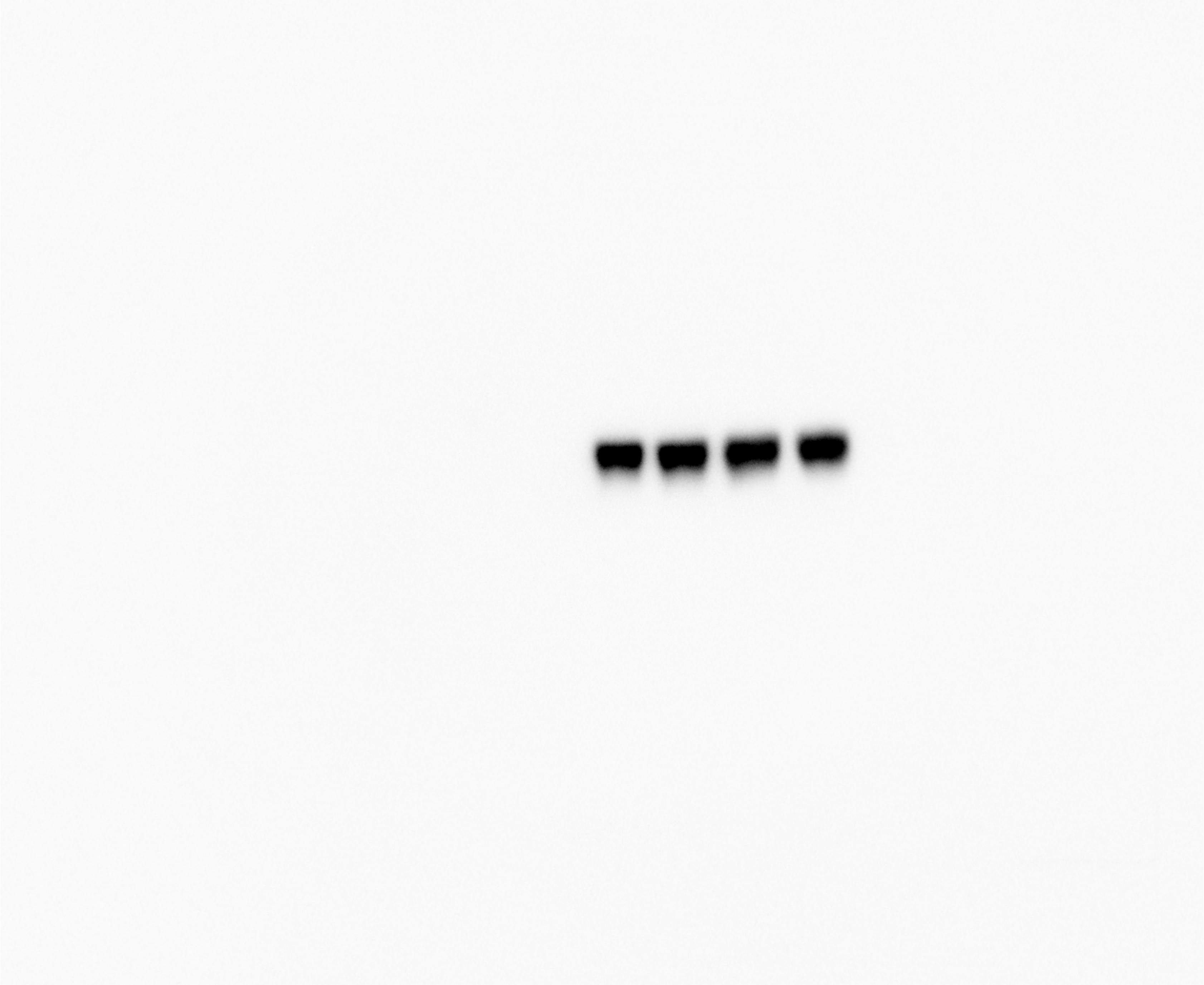

Figure-S8L-IP-HA

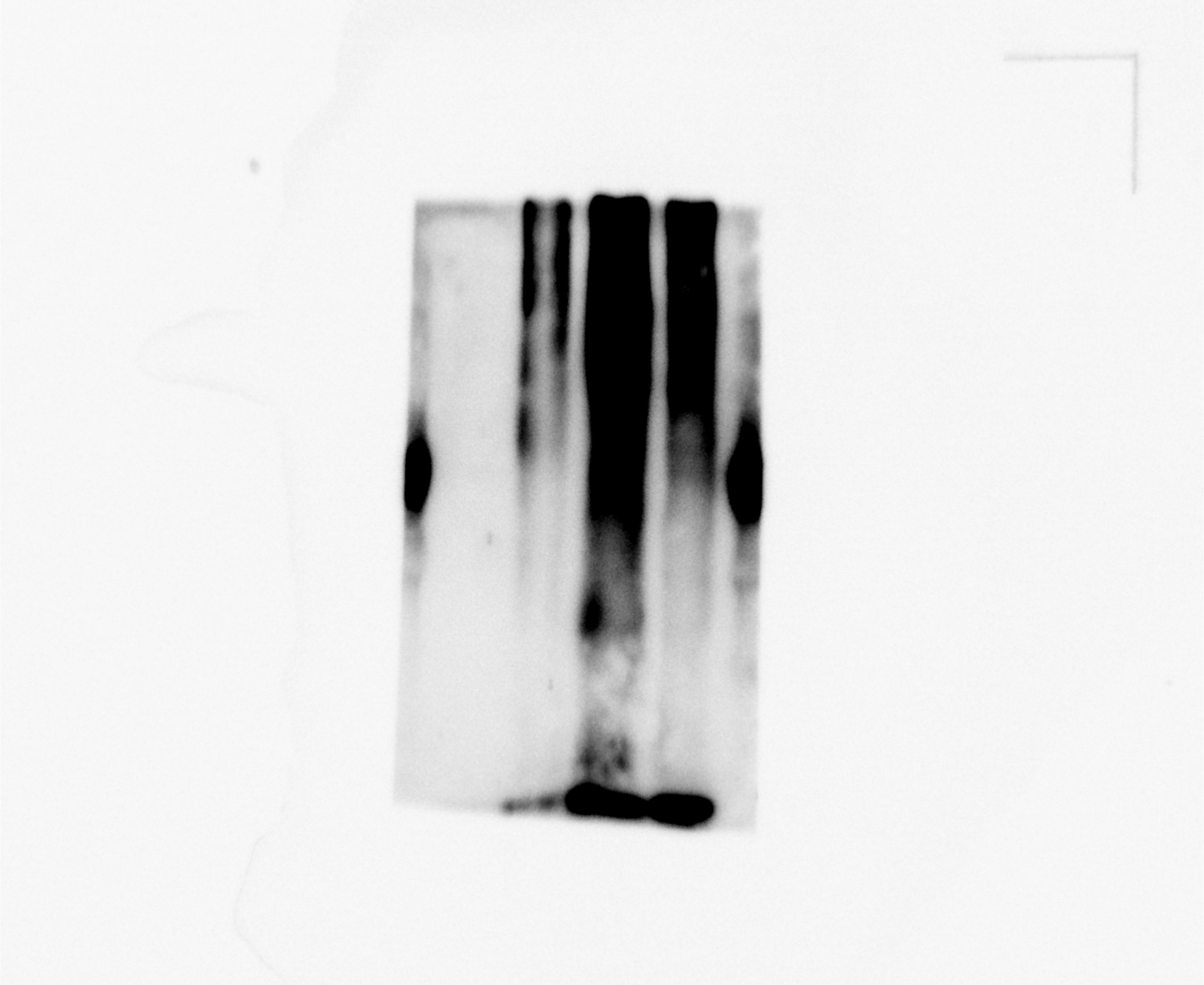

Figure S10 SLC7A6 T24

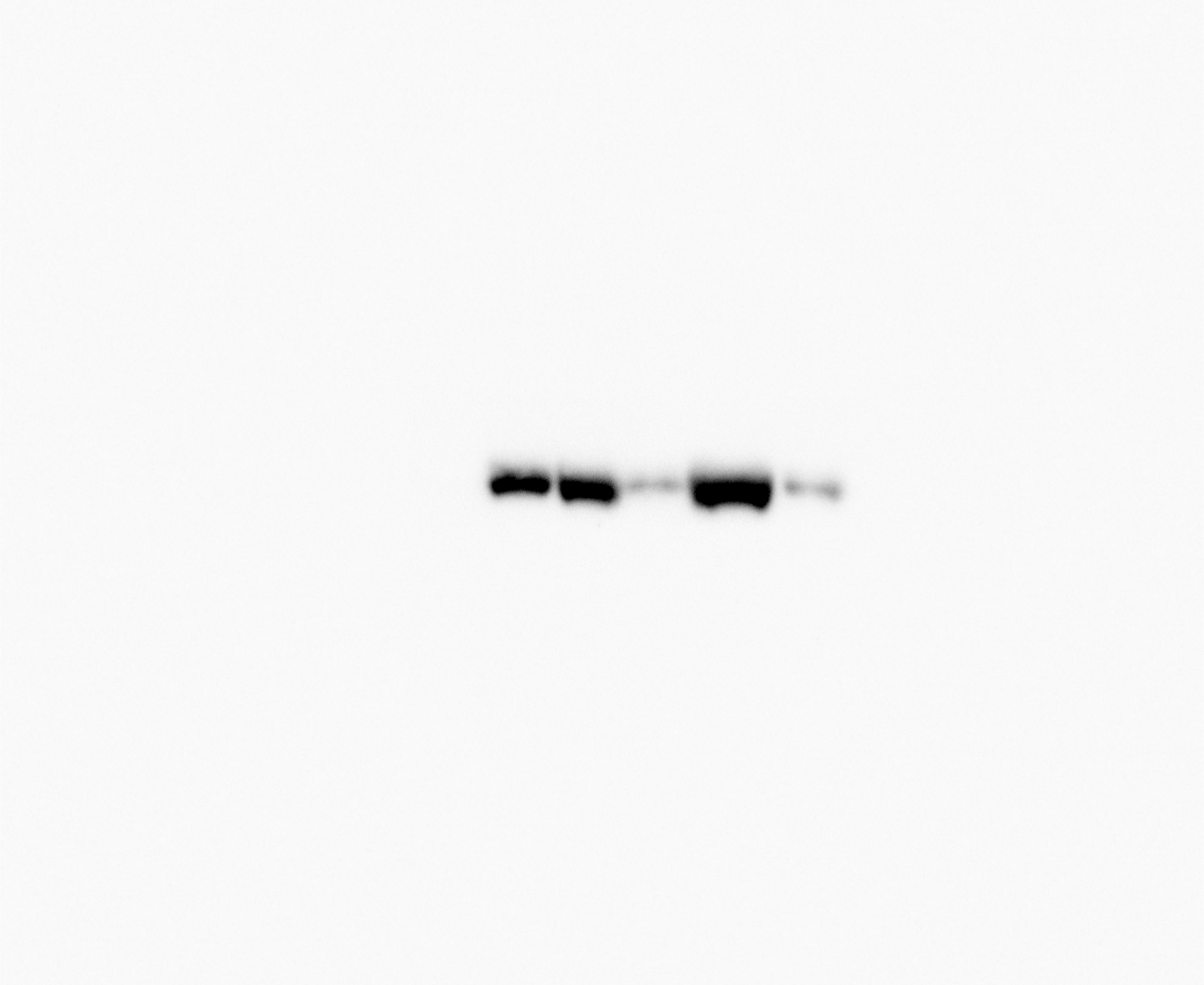

Figure S10 GAPDH MB49

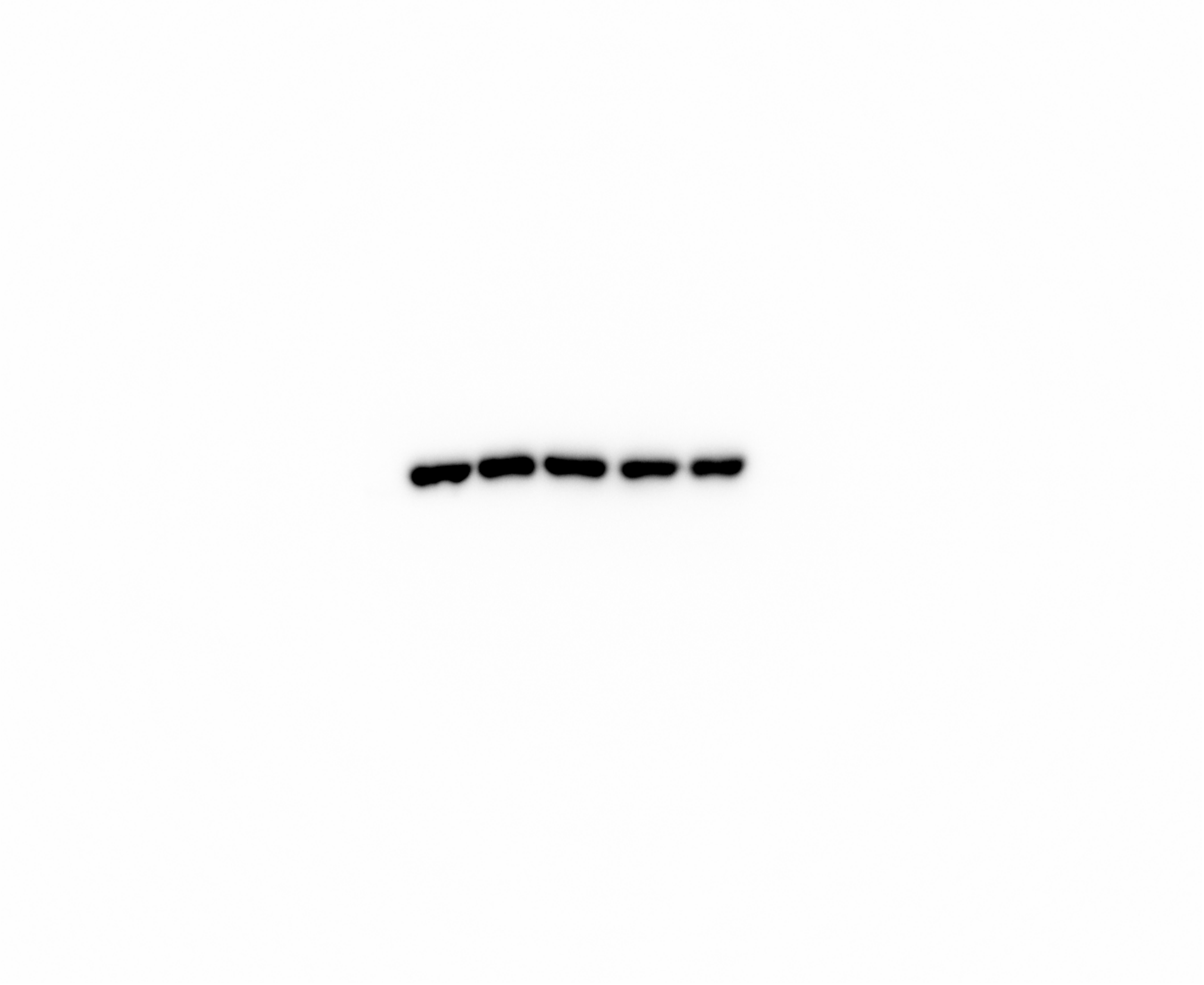

Figure S10 GAPDH T24

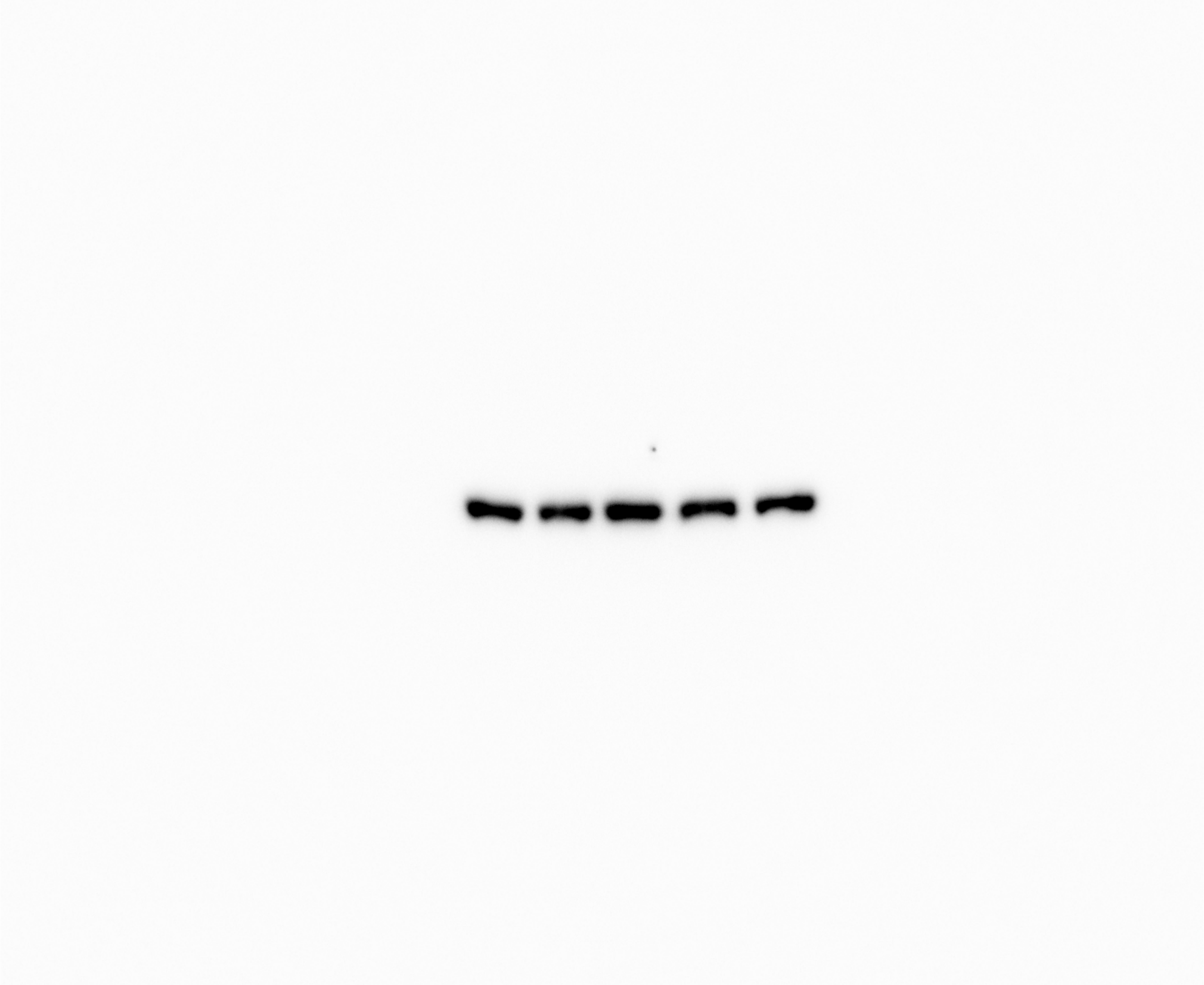

Figure S10 MAT2A MB49

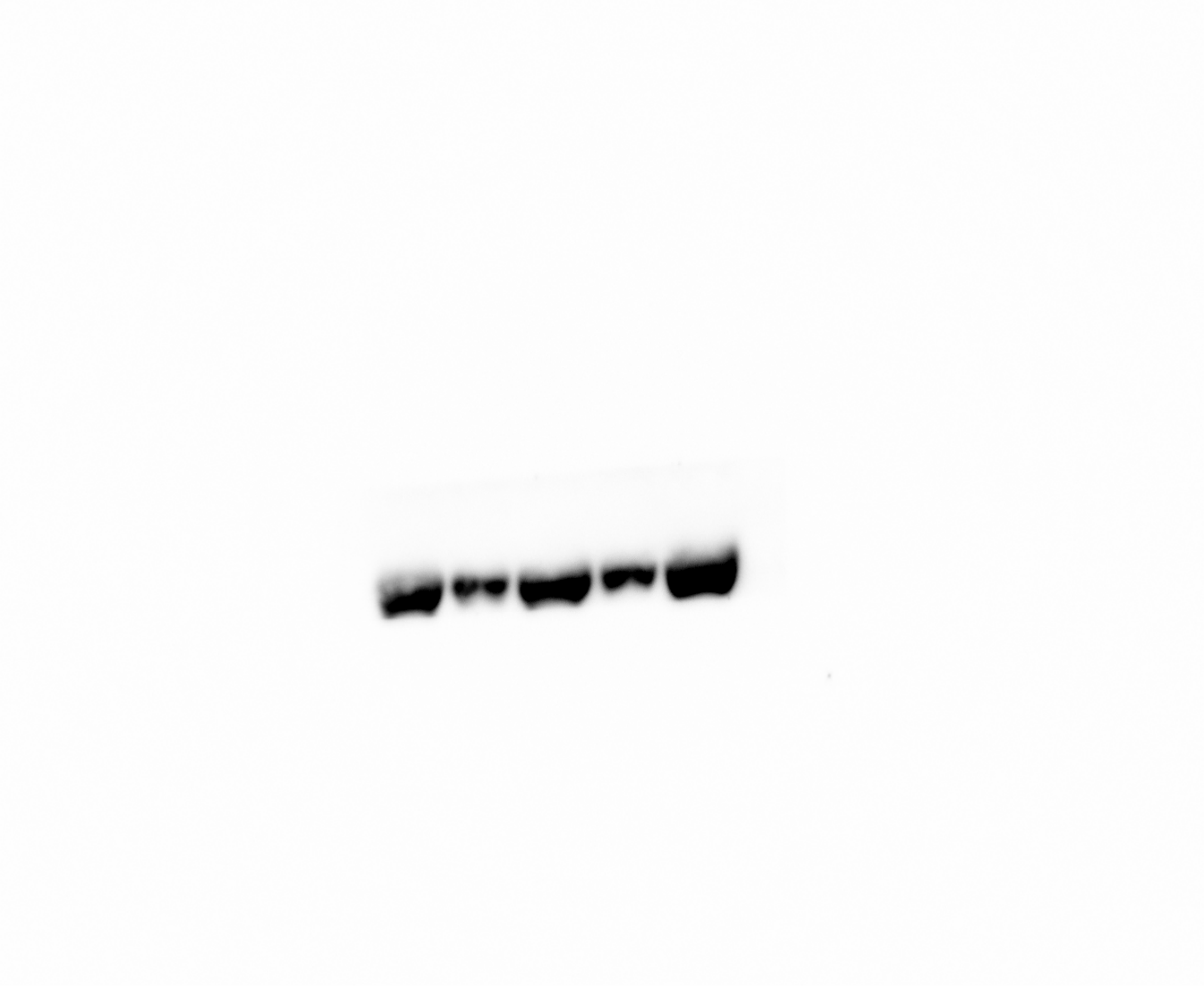

Figure S10 MAT2A T24

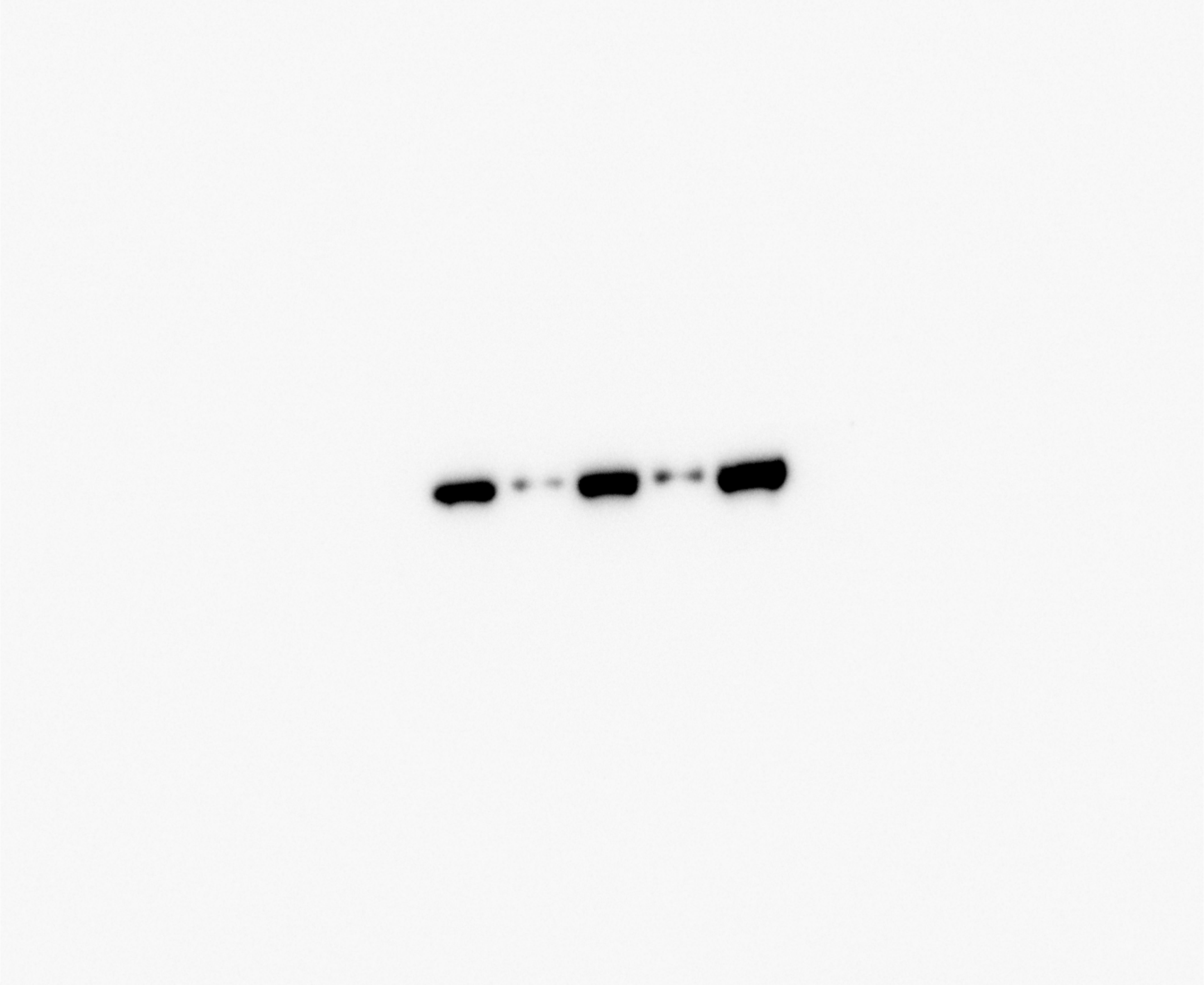

Figure S10 SLC7A6 MB49

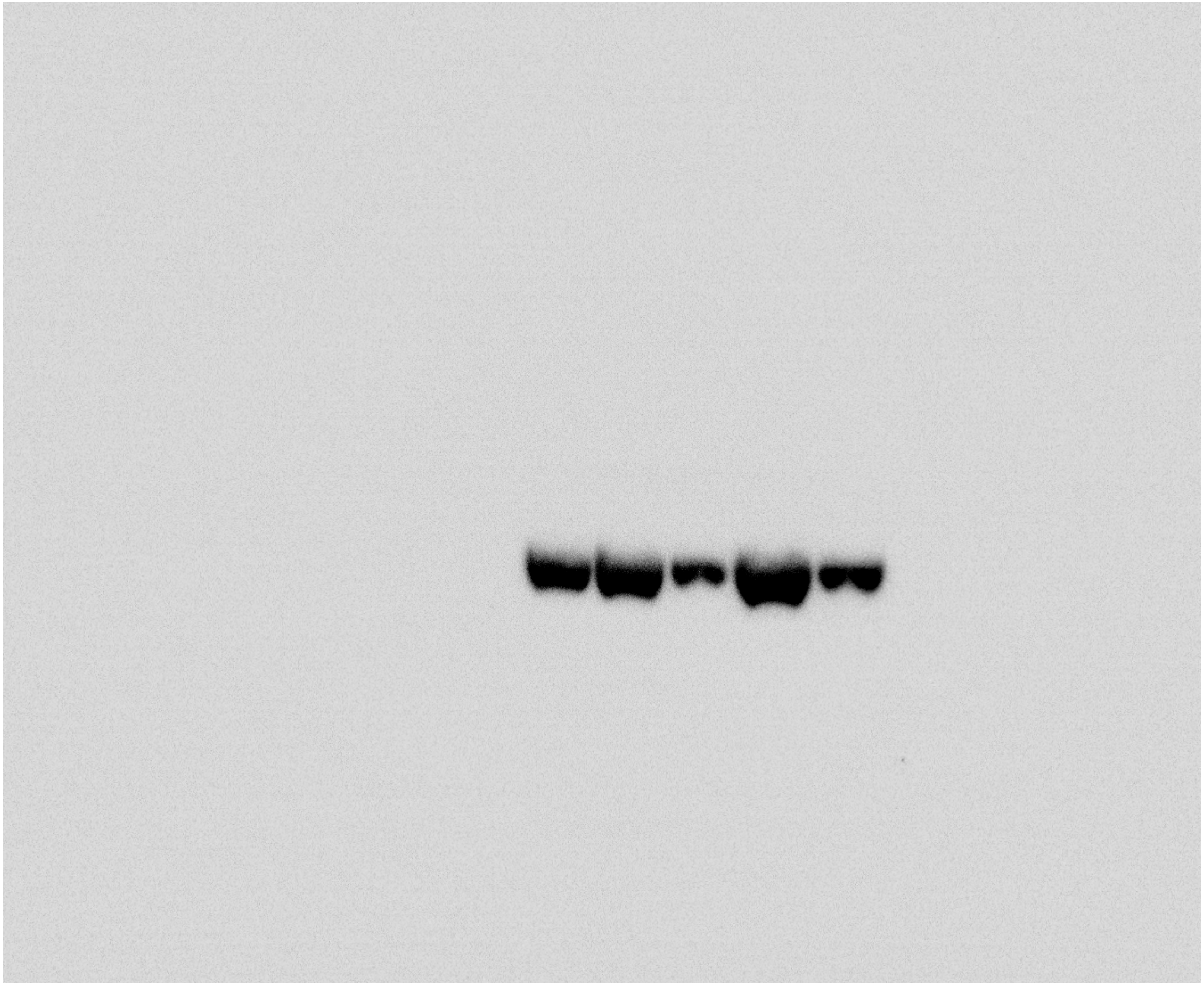

Revised Figure 6 p5 p-STAT5

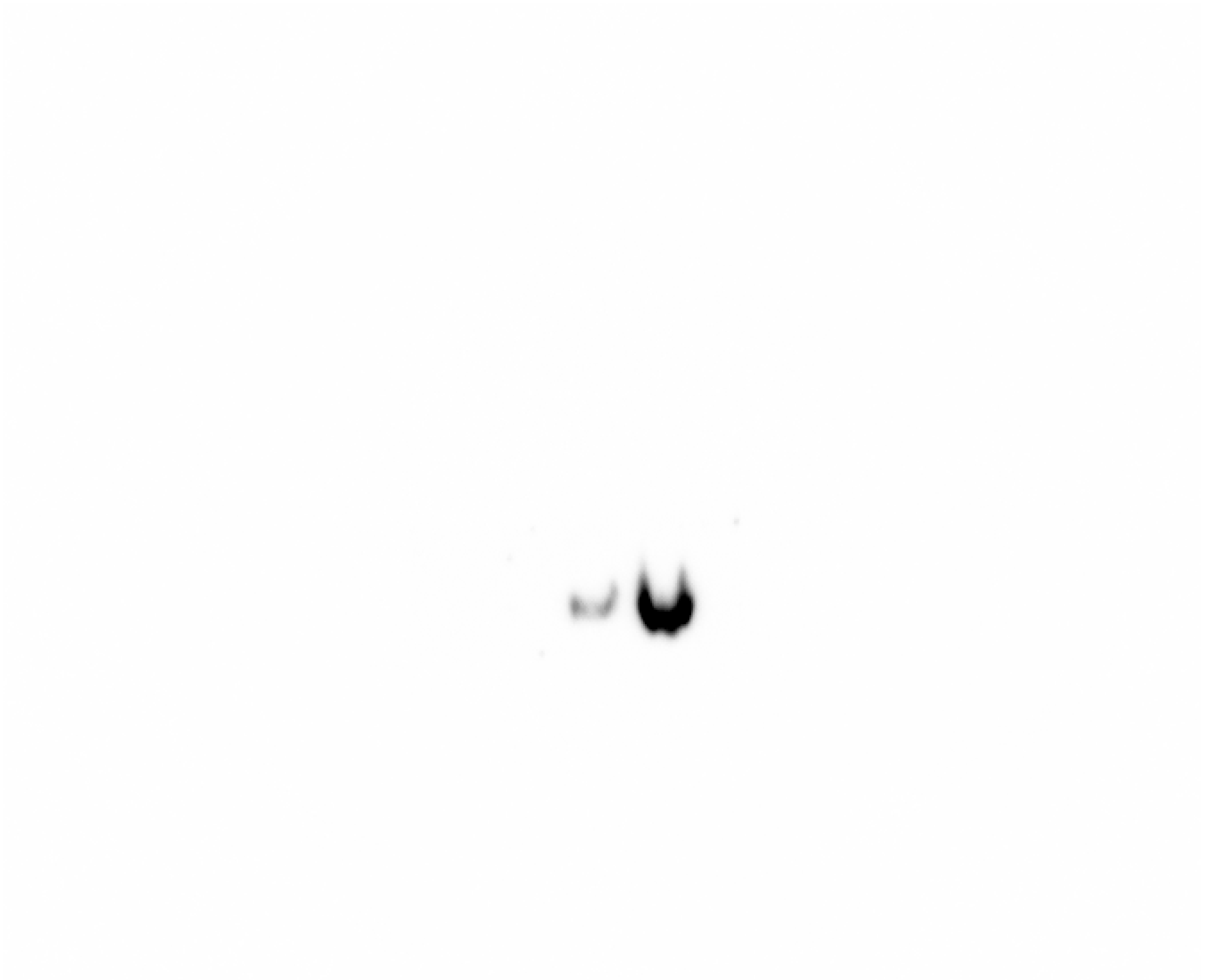

Revised Figure 6 6F p-STAT5

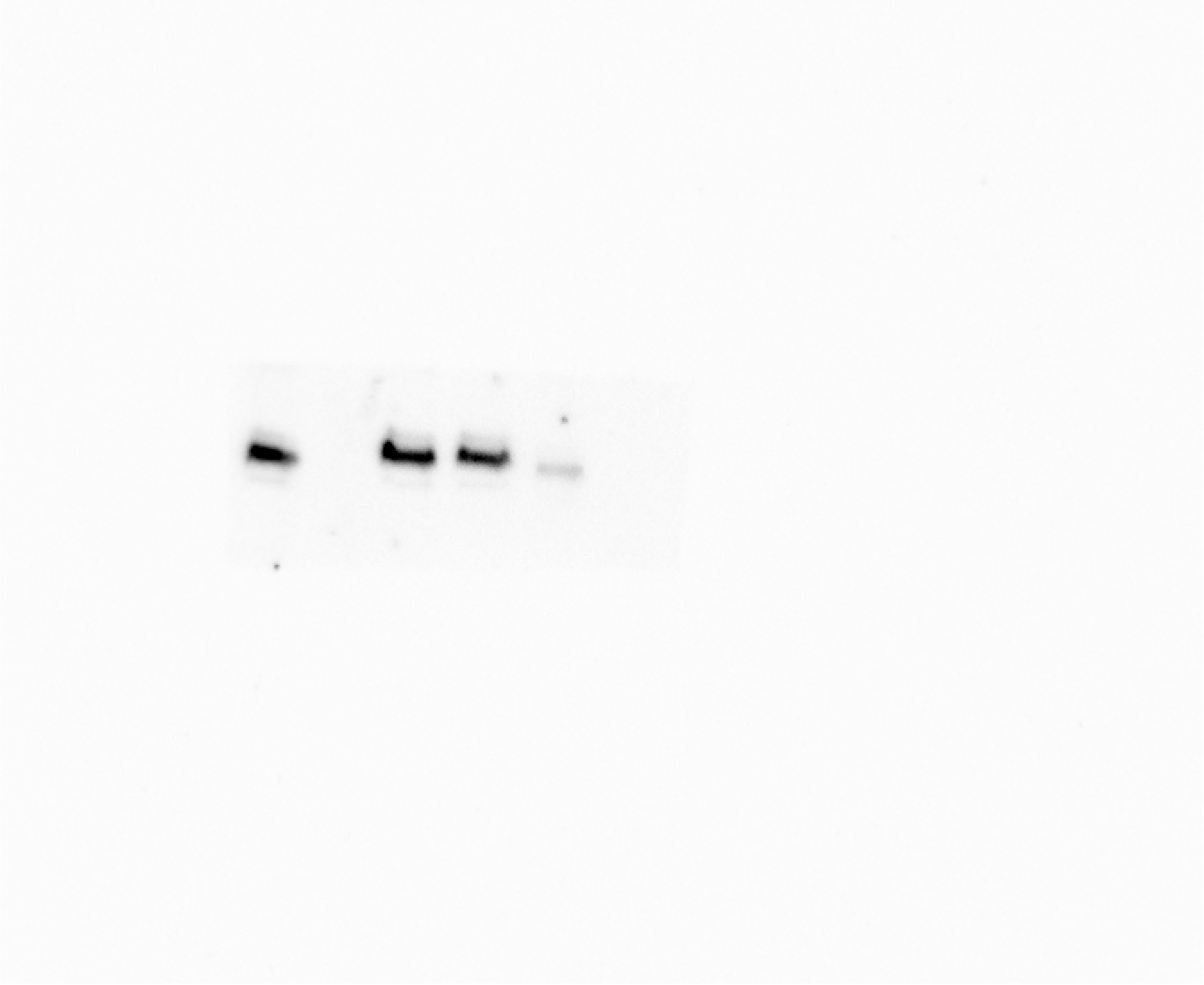

Revised Figure 6 6H p1 p-STAT5

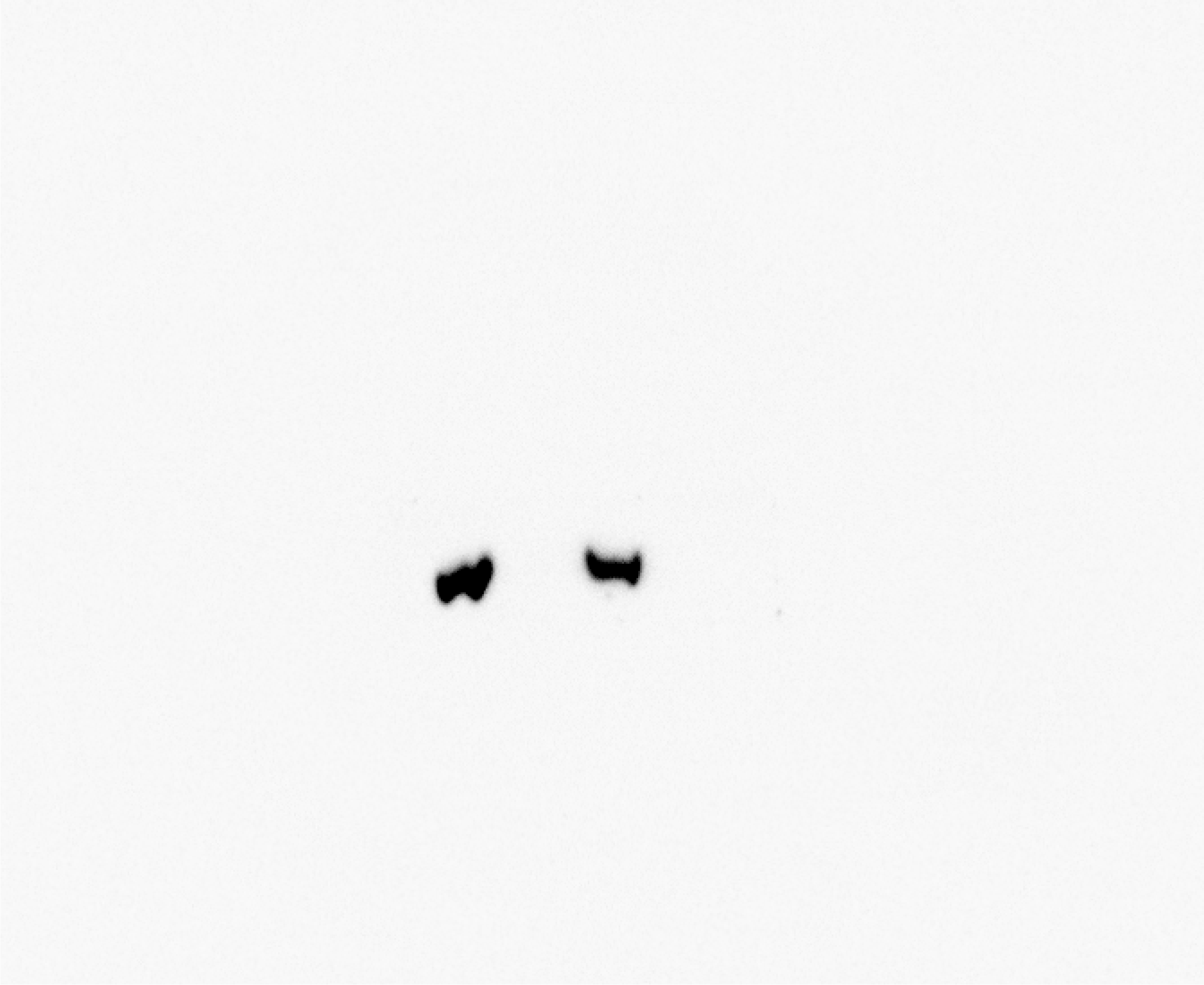

Revised Figure 6 6H p4 p-STAT5

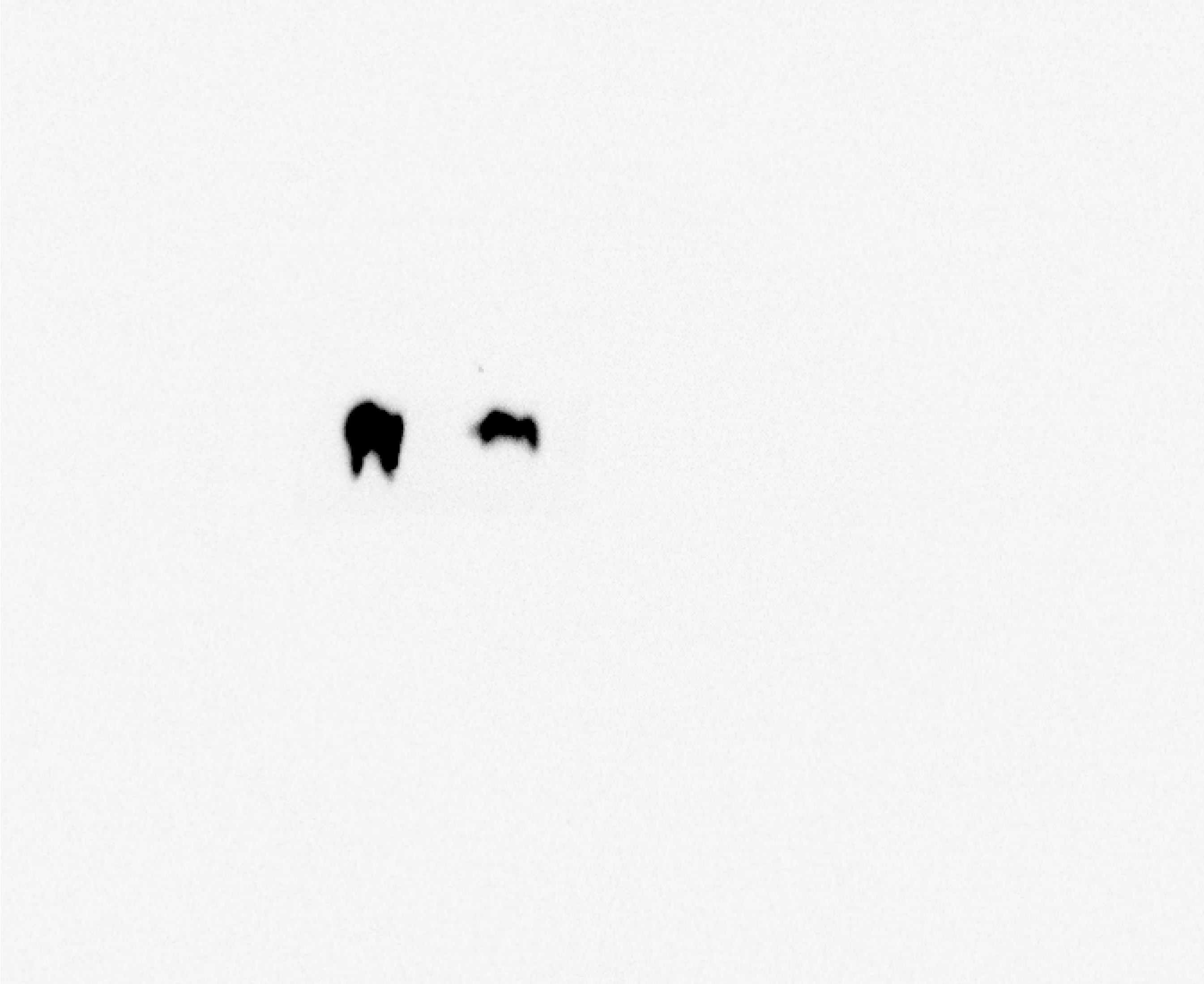

Revised Figure 6 p1 p-STAT5

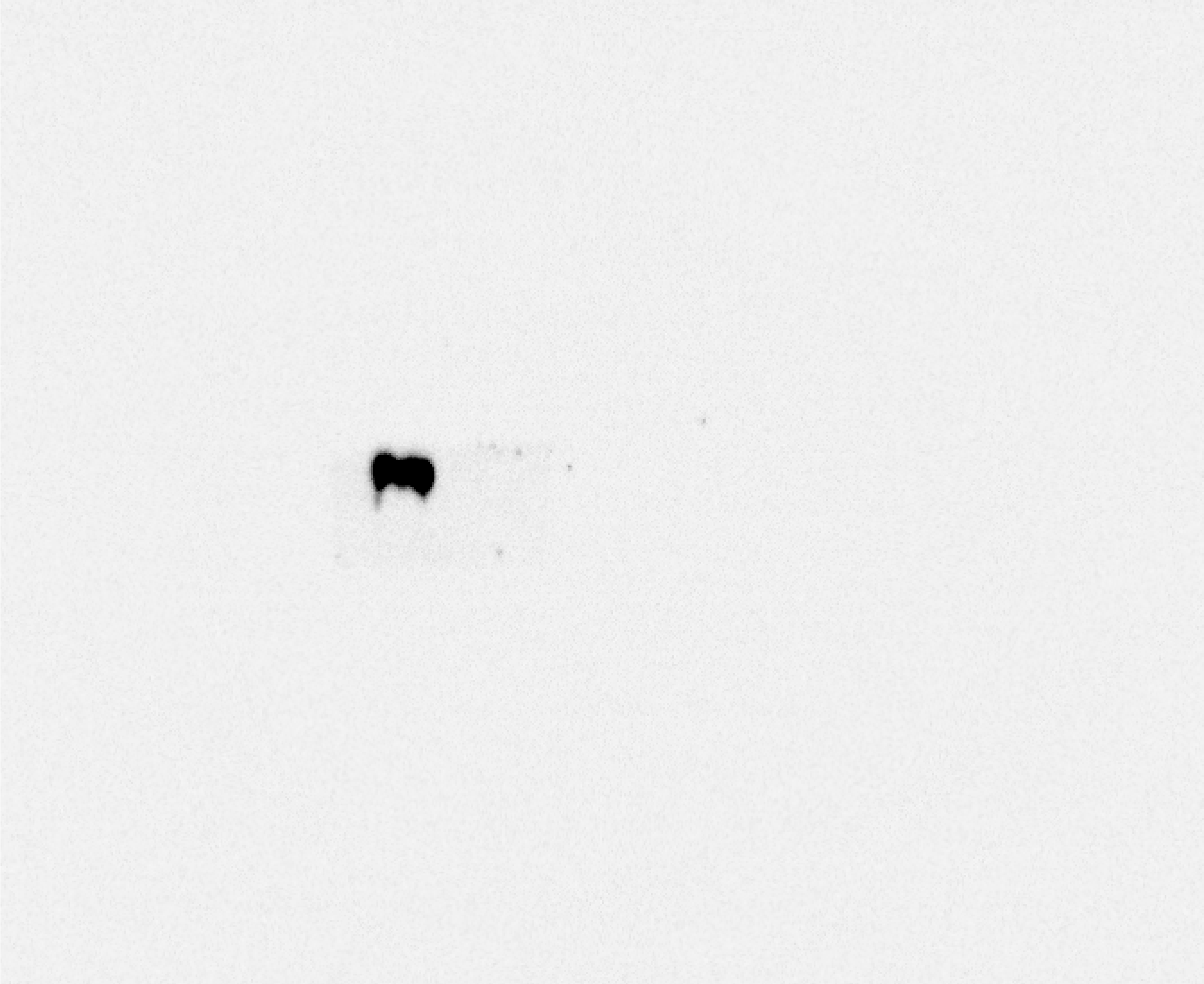

Revised Figure 6 p2 p-STAT5

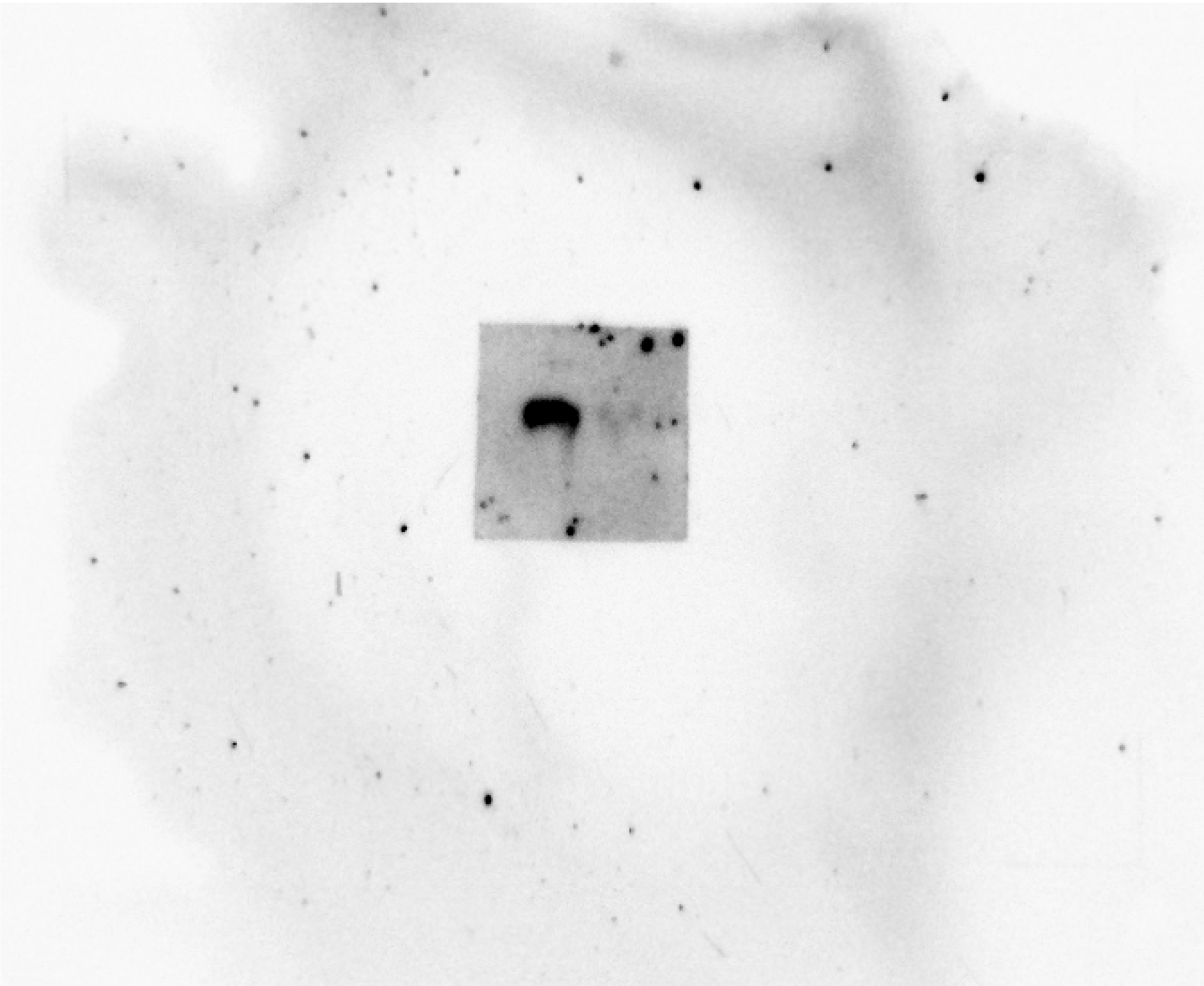

Revised Figure 6 p3 p-STAT5

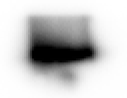

Revised Figure 6 p4 p-STAT5

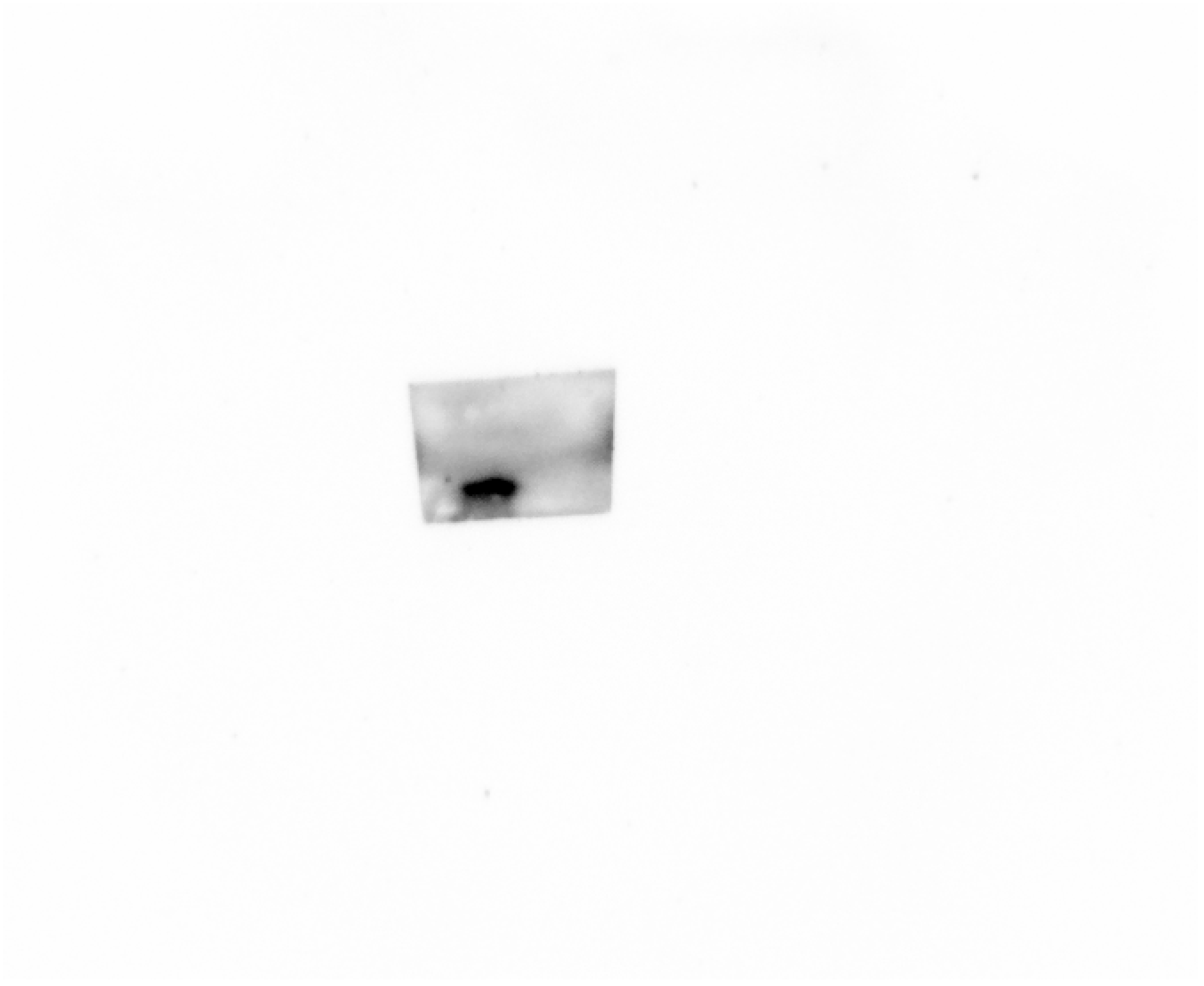

Revised Figure 7 p4 p-STAT5

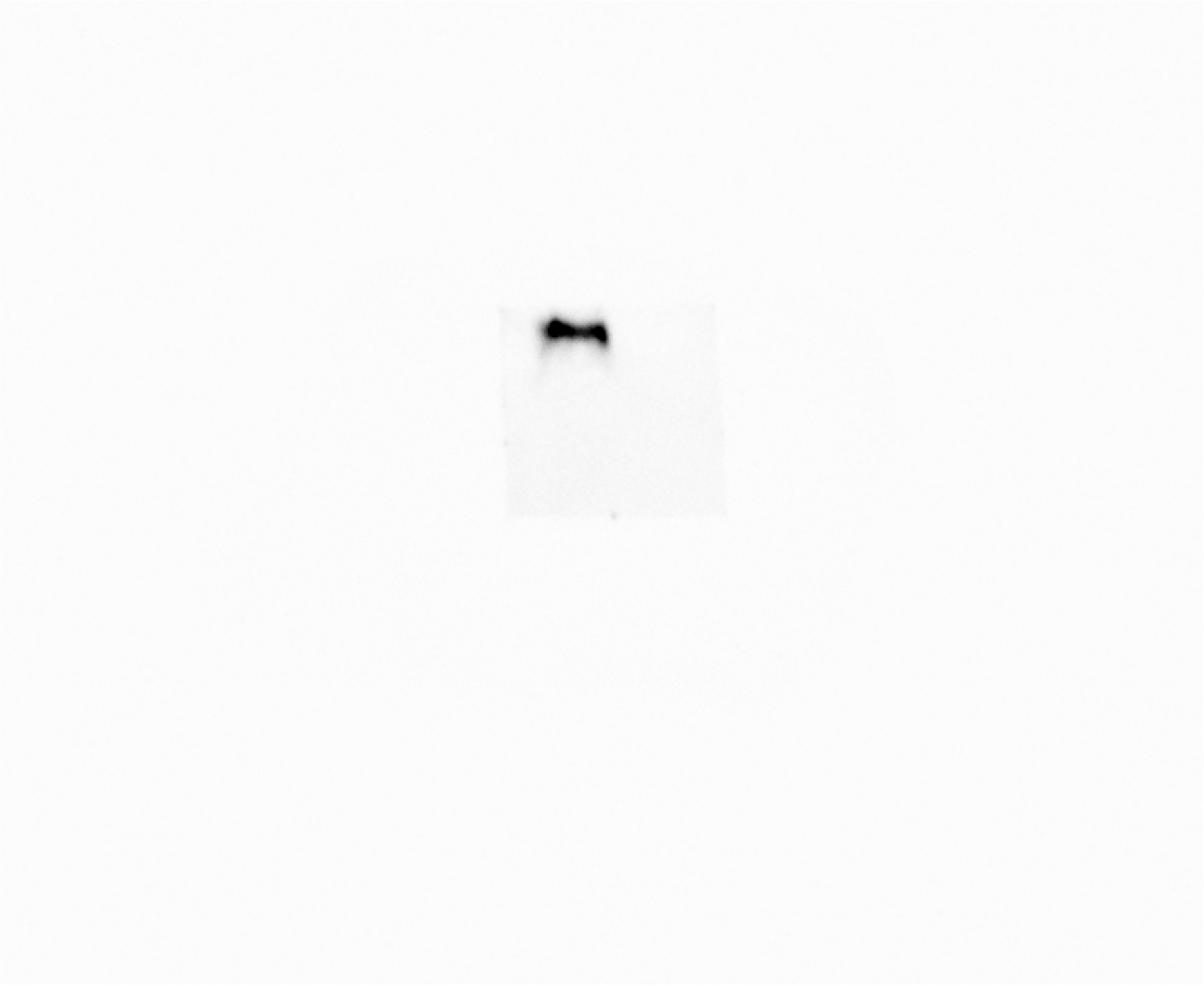

Revised Figure 7 p1 p-STAT5

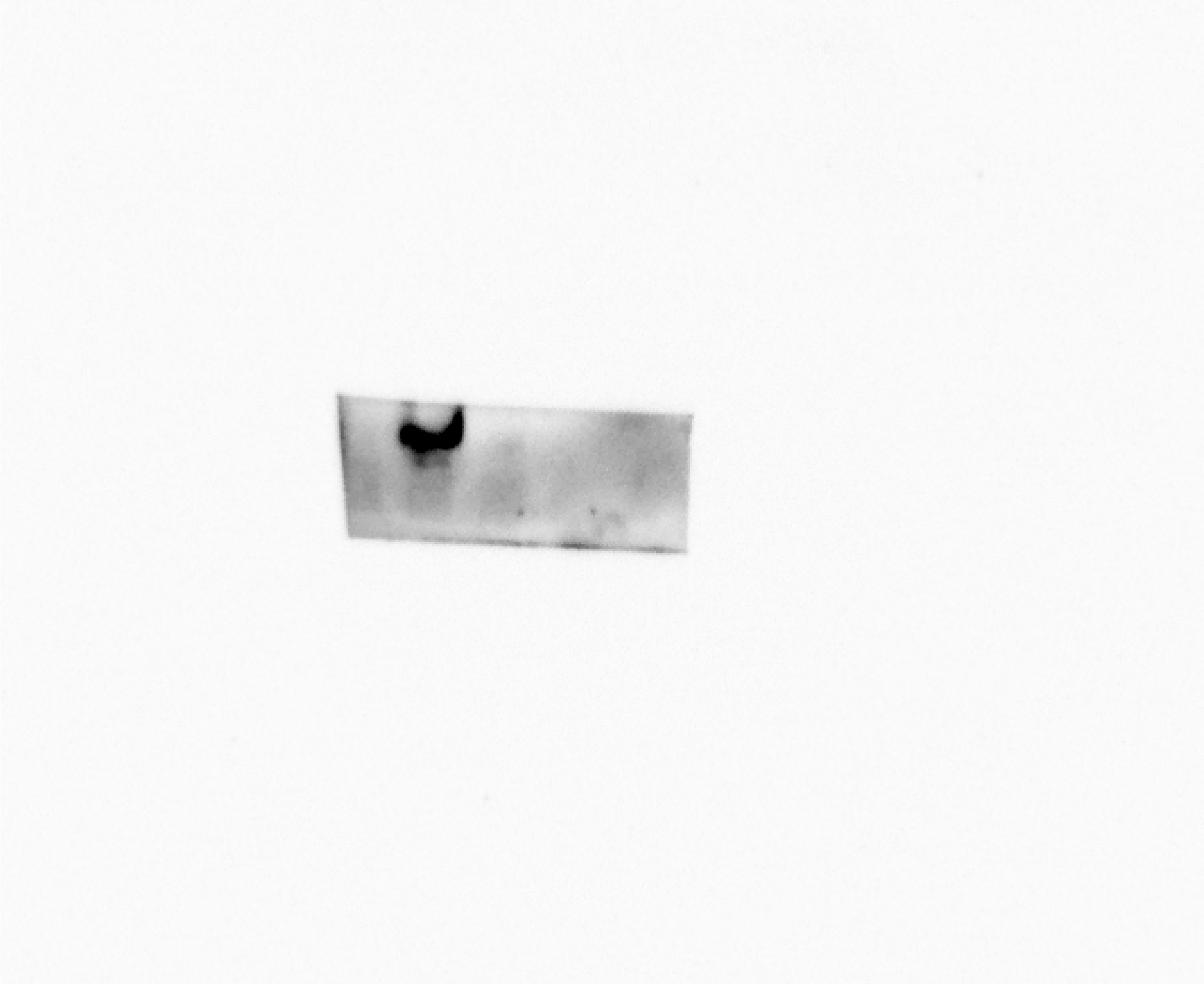

Revised Figure S7 total H3

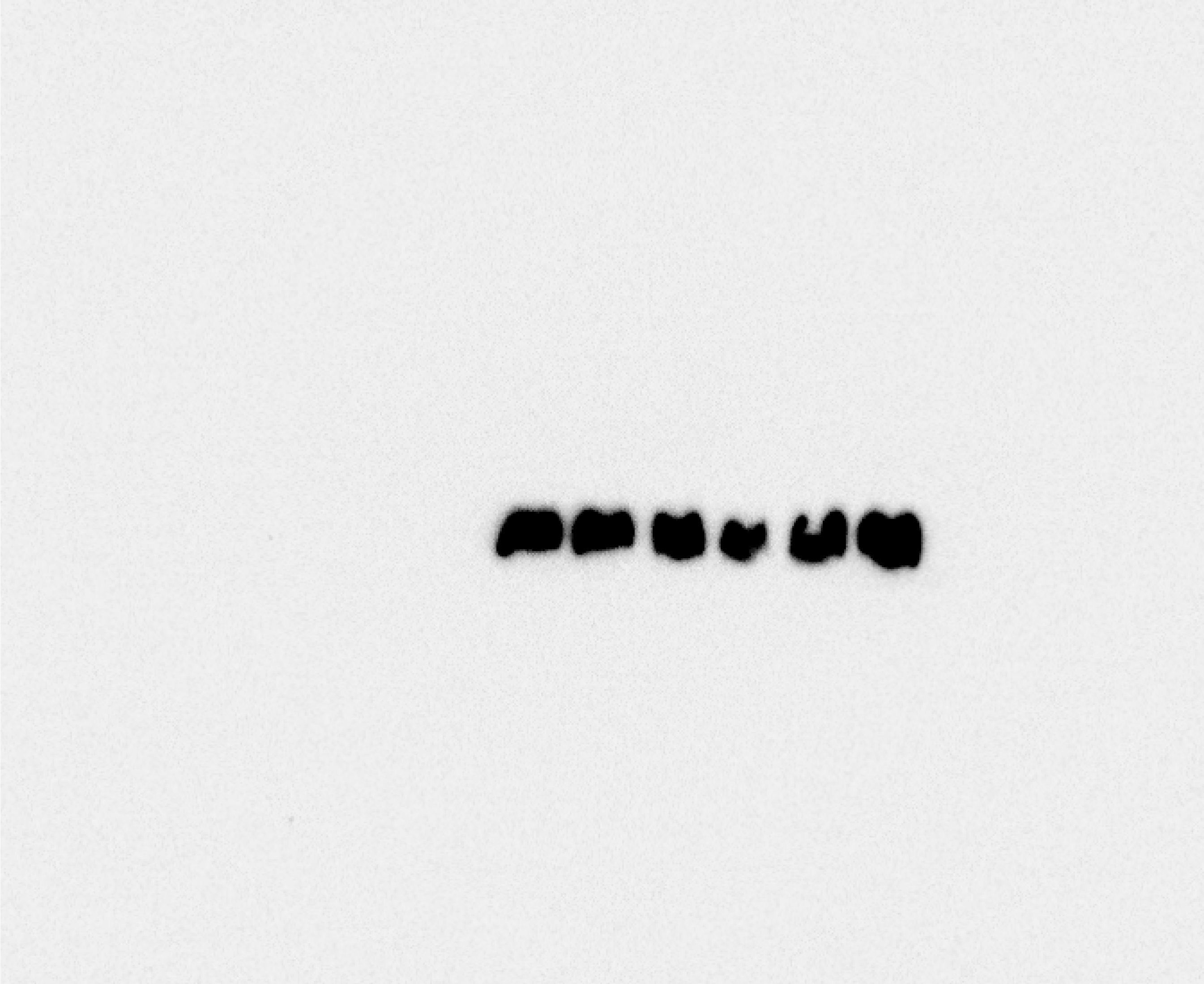

Revised Figure S7 GAPDH

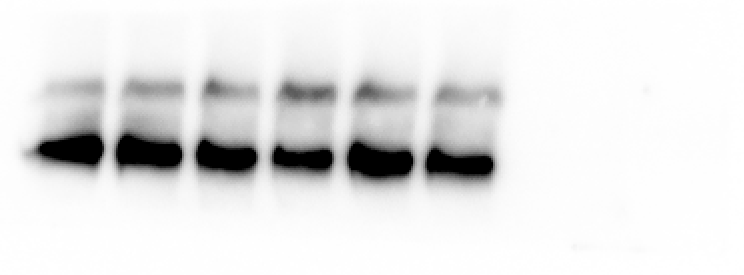

Revised Figure S7 H3K4me3

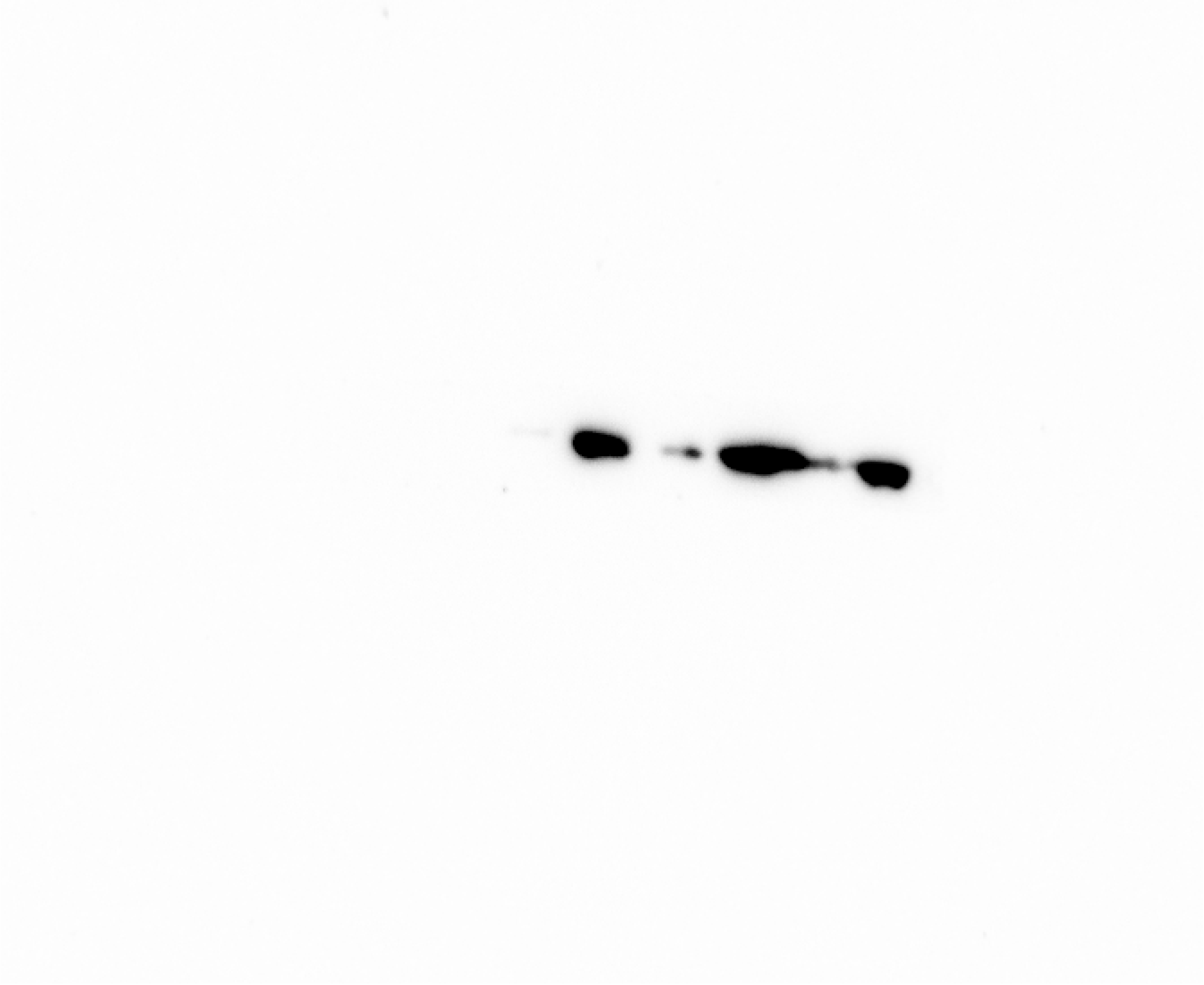

Revised Figure S7 H3K9me3

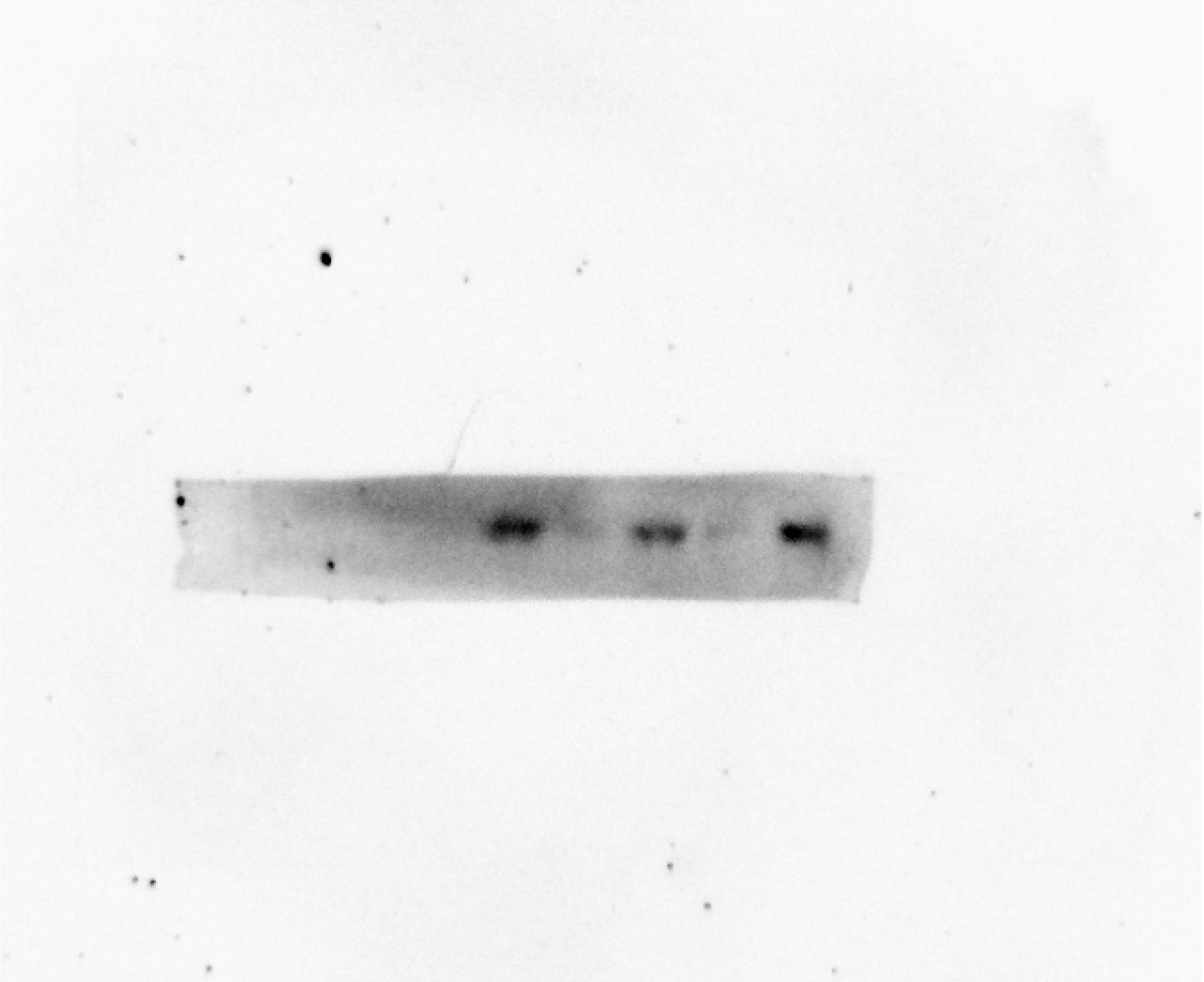

Revised Figure S7 H3K27me3

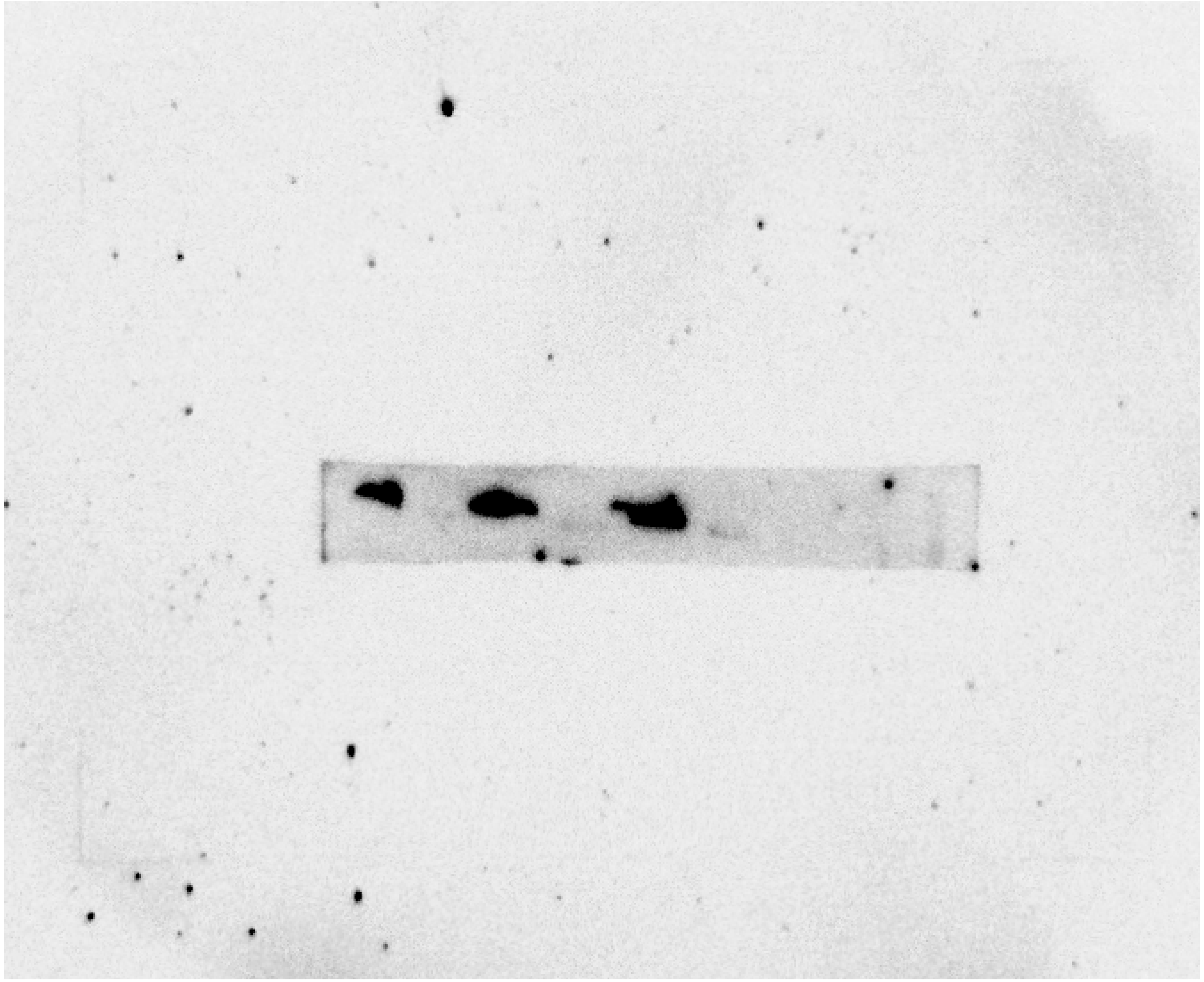

Revised Figure S7 H3K36me2

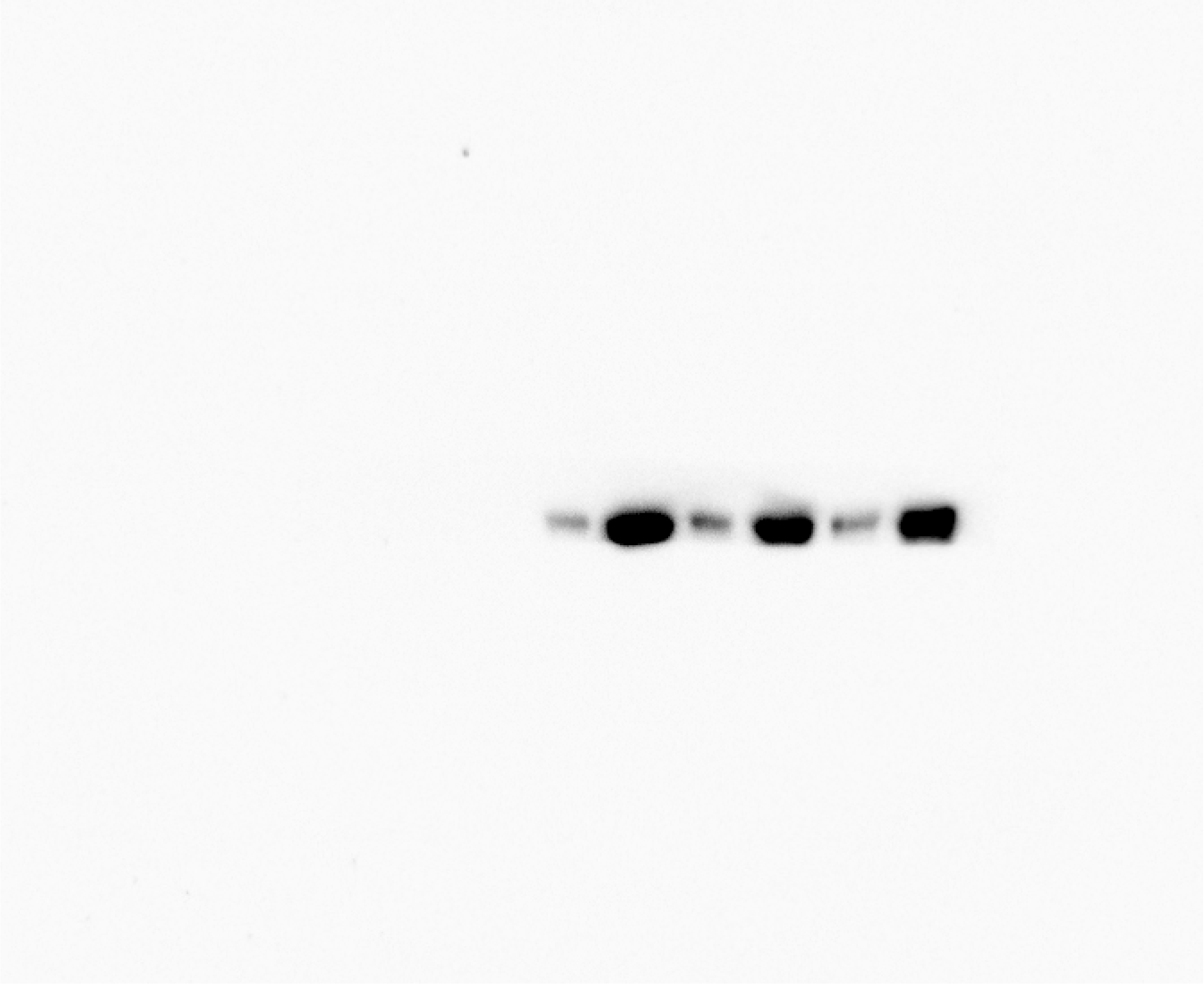

Revised Figure S7 H3K36me3

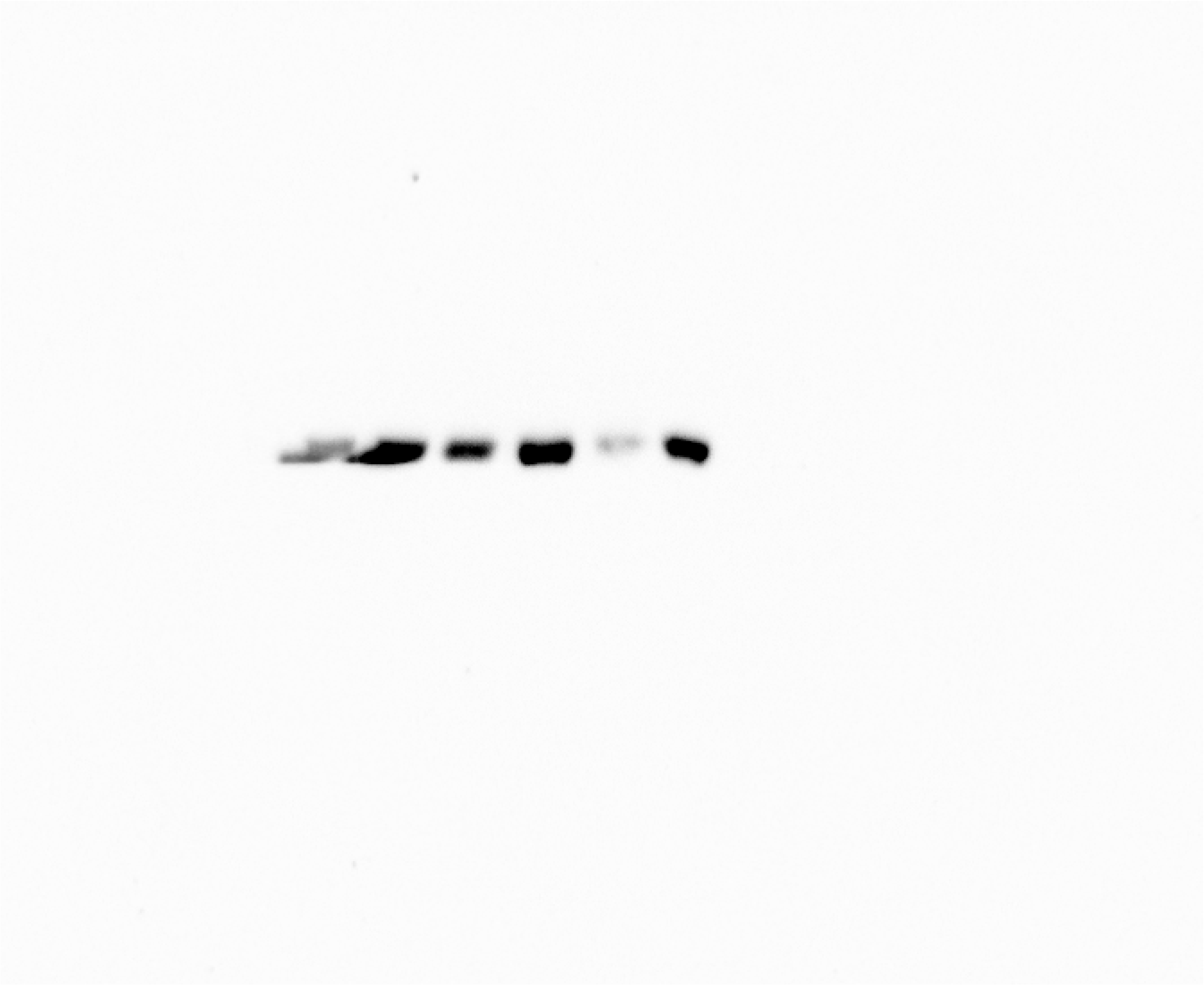

Revised Figure S7 H3K79me2

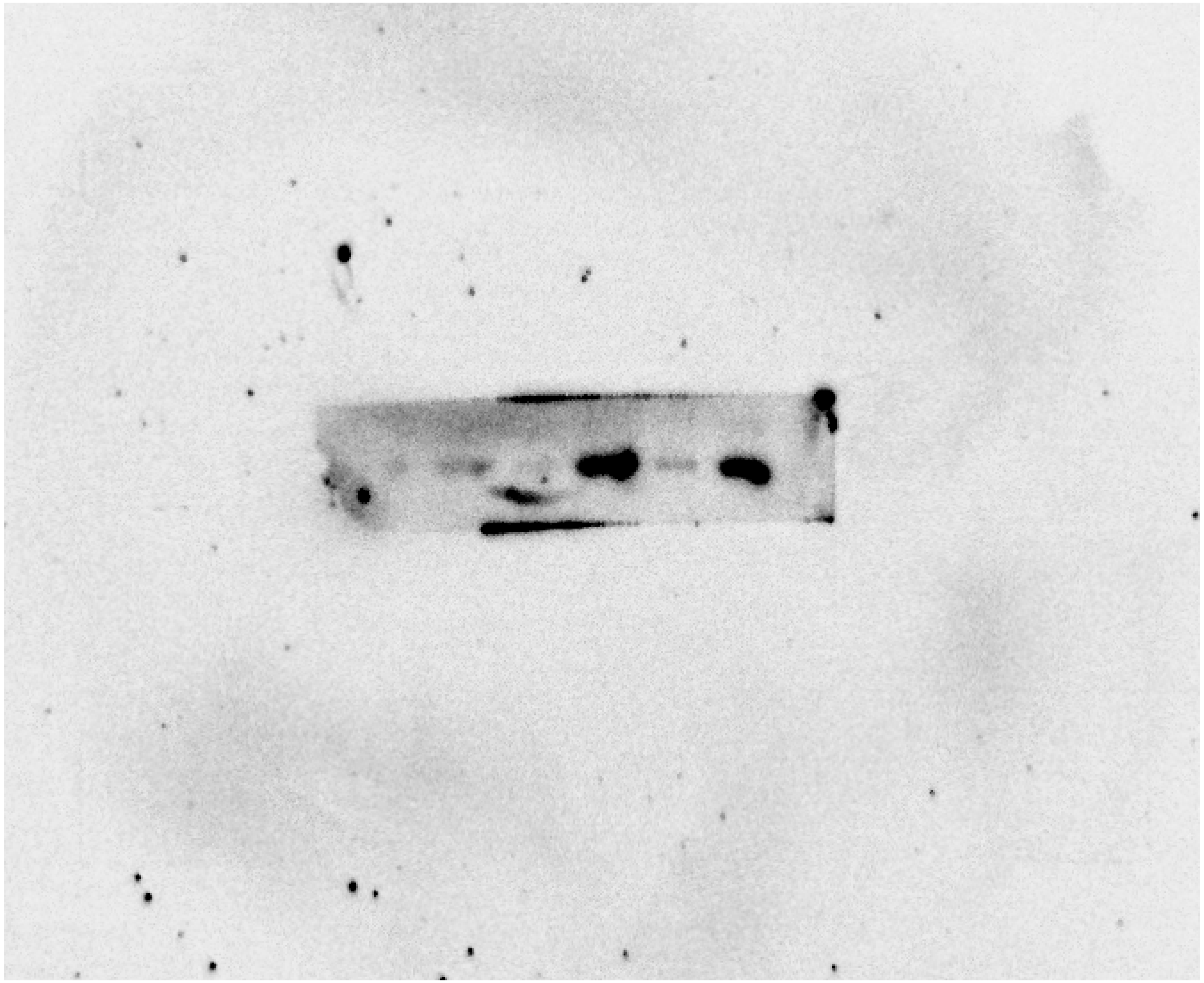

Revised Figure S7 H3K79me3

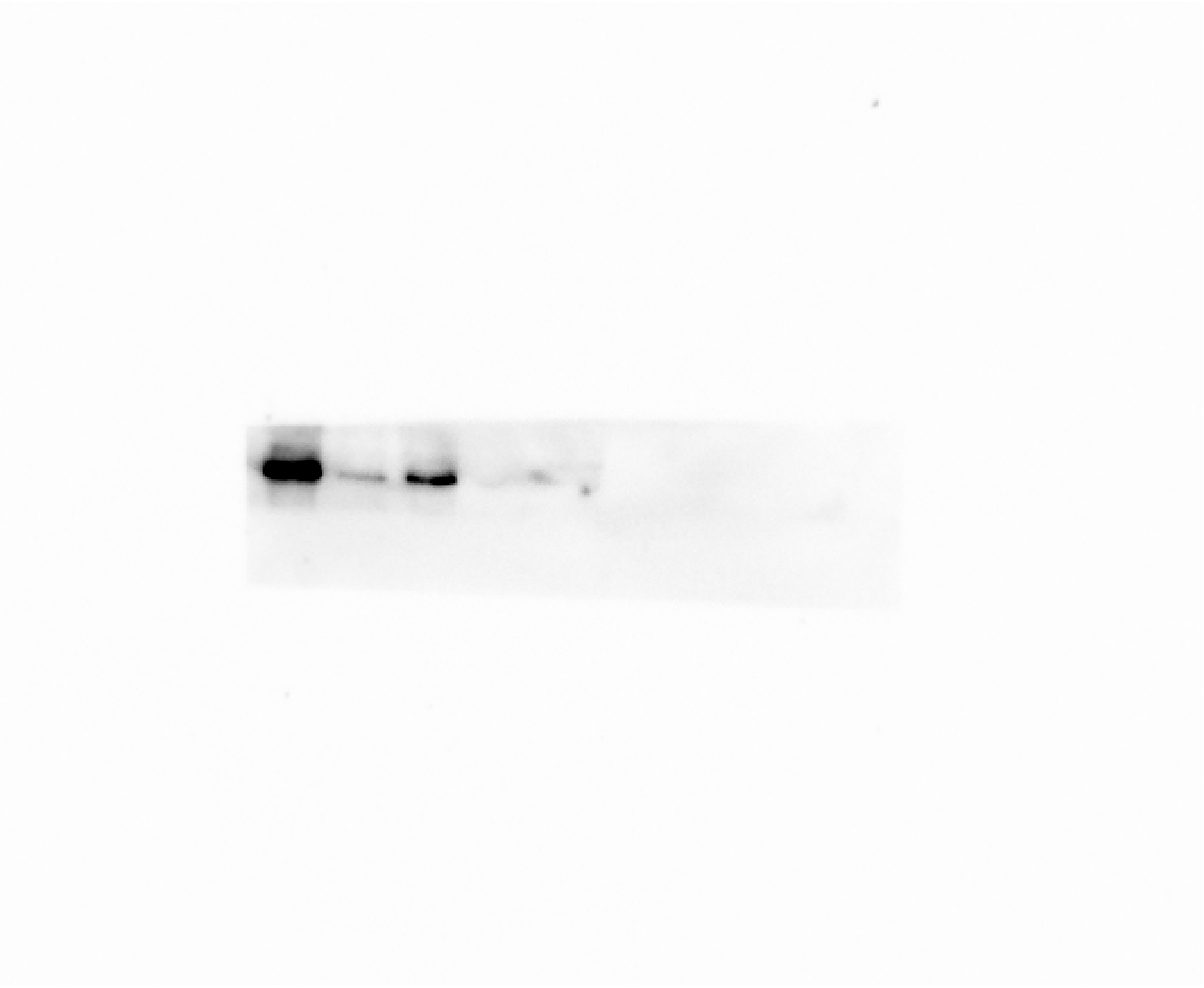

Revised Figure S7 MAT2A

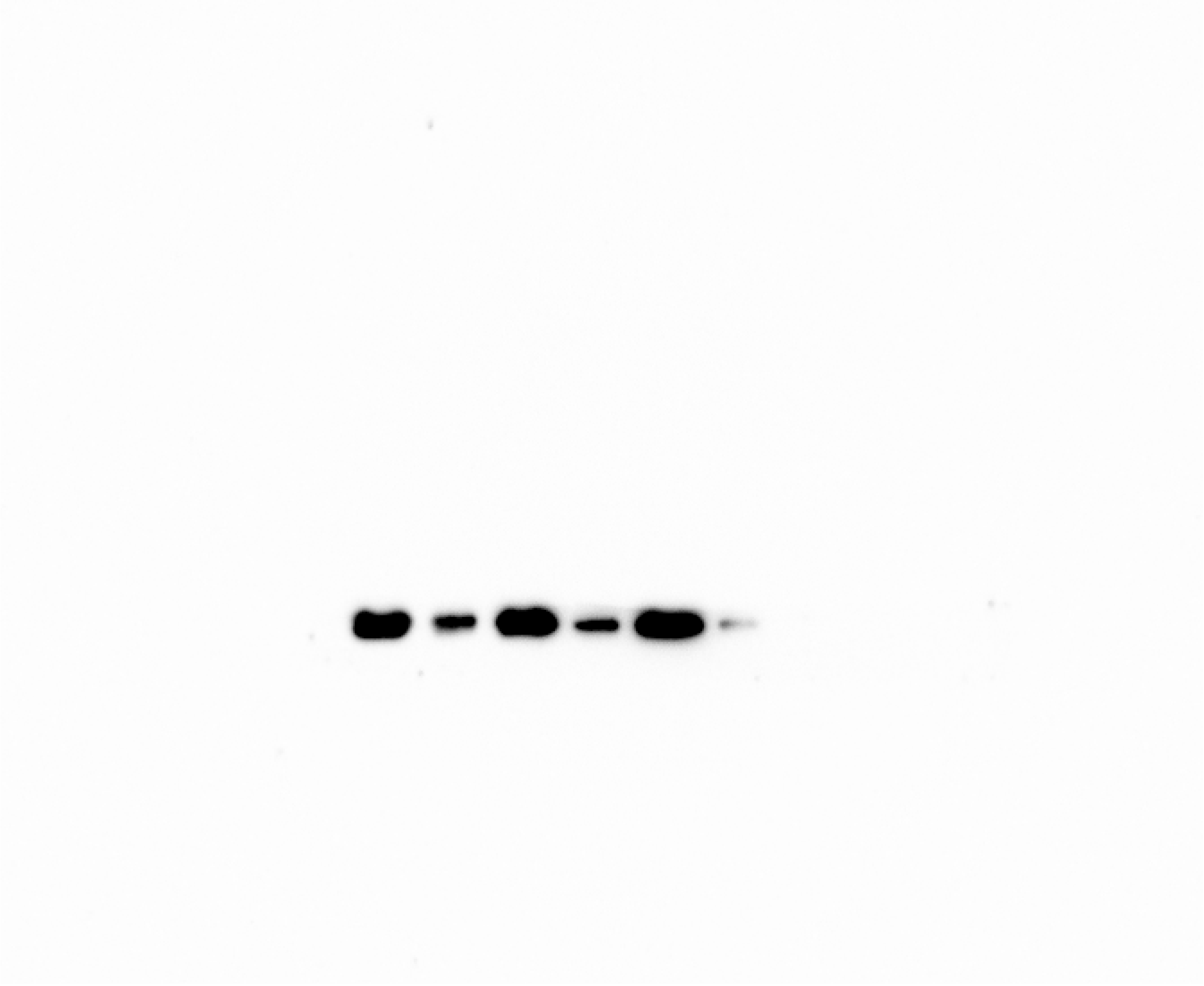

Supplement: Supplementary file 3 — Original Data File [file 41419_2023_6050_MOESM3_ESM.pdf]
