# Supplementary material for: Methionine orchestrates the metabolism vulnerability in cisplatin resistant bladder cancer microenvironment
Source: Cell Death Dis. 2023 Aug 15;14(8):525. doi: 10.1038/s41419-023-06050-1 (PMC10427658; doi:10.1038/s41419-023-06050-1)
Supplement: Supplementary file 4 — primers [file 41419_2023_6050_MOESM4_ESM.doc]

**Table S2 Primers and Probes**

**RT-qPCR primers:**

GAPDH Forward 5-CAAGGCTGAGAACGGGAAG-3

GAPDH Reverse 5-TGAAGACGCCAGTGGACTC-3

Gapdh Forward 5-AGGTCGGTGTGAACGGATTTG-3

Gapdh Reverse 5-GGGGTCGTTGATGGCAACA-3

ARHGAP10 Forward 5-CCCAGCGGAAGTTTGCTCAT-3

ARHGAP10 Reverse 5-ACAGCTCCAAGTTGCTCTTTTC-3

circARHGAP10 Forward 5-CAGATTGGGAAGTGAAGA-3

circARHGAP10 Reverse 5-TTTATACTGGCTGGAACA-3

Arhgap10 Forward 5-TCGCTCAGAGACTTCAAGTTCG-3

Arhgap10 Reverse 5-ATTCCCGTAAGGAGGCATCTAT-3

circArhgap10 Forward 5-CTTCATTGTTCCAGCCAGTA-3

circArhgap10 Reverse 5-GGACCTTTGAACTCACCC-3

hsa_circ_0087630 Forward 5-CATGGGTGGATGTGGAGT-3

hsa_circ_0087630 Reverse 5-CTGTCTCATAGGGTCGGTA-3

hsa_circ_0003885 Forward 5-TTATGAGCGGGAGGAAGT-3

hsa_circ_0003885 Reverse 5-TTCTTATGATGCCTTTGC-3

hsa_circ_0001833 Forward 5-GTGCTGCGCTGCTTCACT-3

hsa_circ_0001833 Reverse 5-AGATGTCGGTGGCAAAGG-3

hsa_circ_0058051 Forward 5-CCAGCATAAGGCATTGGT-3

hsa_circ_0058051 Reverse 5-GCTCACATCCTCCTAAACA-3

hsa_circ_0004849 Forward 5-AAGCTCACTCAGATTCCCTC-3

hsa_circ_0004849 Reverse 5-ATCAAATCGCCCAAACAC-3

hsa_circ_0001727 Forward 5-CAGGCTCCAGGAACTGAC-3

hsa_circ_0001727 Reverse 5-CTGAGATGCTCGCAAGGG-3

SLC7A5 Forward 5-CCGTGAACTGCTACAGCGT-3

SLC7A5 Reverse 5-CTTCCCGATCTGGACGAAGC-3

SLC7A6 Forward 5-CGTGTCCGCCGTAATCTCC-3

SLC7A6 Reverse 5-CTAACAACTGAAAGCCCGAGT-3

SLC7A7 Forward 5-CCCAAGGGTGTGCTCATATACA-3

SLC7A7 Reverse 5-CCAGTTCCGCATAACAAAGGG-3

SLC7A8 Forward 5-AGGCTGGAACTTTCTGAATTACG-3

SLC7A8 Reverse 5-ACATAAGCGACATTGGCAAAGA-3

SLC7A9 Forward 5-TGGGCACCATCATTGGCTC-3

SLC7A9 Reverse 5-GGCCTCCATCAGGTAGGGAT-3

SLC38A1 Forward 5-CAGCATCGTAGGGGTCAGTG-3

SLC38A1 Reverse 5-CATGAGCAGAAGACCAAGAGC-3

SLC38A2 Forward 5-ATGAGTTGCCTTTGGTGATCC-3

SLC38A2 Reverse 5-ACAGGACACGGAACCTGAAAT-3

SLC38A4 Forward 5-AGAAATTCCAAATACCCTGCCC-3

SLC38A4 Reverse 5-GAAGCGTGTTGTTGAATGACAG-3

SLC43A1 Forward 5-GGACGTGGAAGCTCTGTCTC-3

SLC43A1 Reverse 5-GCAGCGTGAGTGAAGTGAAC-3

SLC43A2 Forward 5-AGTCAGAGGGCTTTTACTCCTAC-3

SLC43A2 Reverse 5-GTCCATGACGATACCCAGGG-3

**Probes:**

circARHGAP10-Cy3: 5-CTTGGTCATTTATACTGGCTGGAACAATG-3

circARHGAP10-Bio: 5-CTTGGTCATTTATACTGGCTGGAACAATG-3

**Oligonucleotides:**

si-NC sense 5-UUCUCCGAACGUGUCACGUTT-3

si-NC antisense 5-ACGUGACACGUUCGGAGAATT-3

si-circARHGAP10 sense 5-CAUUGUUCCAGCCAGUAUATT-3

si-circARHGAP10 antisense 5-UAUACUGGCUGGAACAAUGTT-3
